# Weighted gene co-expression network analysis identifies important modules and hub genes involved in the regulation of breast muscle yield in broilers

**DOI:** 10.5713/ab.23.0548

**Published:** 2024-04-25

**Authors:** Xing Guo, Hao Wang, Meng Liu, Jin-Mei Xu, Ya-Nan Liu, Hong Zhang, Xin-Xin He, Jiang-Xian Wang, Wei Wei, Da-Long Ren, Run-Shen Jiang

**Affiliations:** 1 College of Animal Science and Technology, Anhui Agricultural University, Hefei 230036, China

**Keywords:** Breast Muscle Yield, Chicken, Hub Genes, Weighted Gene Co-expression Network Analysis (WGCNA)

## Abstract

**Objective:**

Increasing breast meat production is one of the primary goals of the broiler industry. Over the past few decades, tremendous progress has been made in genetic selection and the identification of candidate genes for improving the breast muscle mass. However, the molecular network contributing to muscle production traits in chickens still needs to be further illuminated.

**Methods:**

A total of 150 1-day-old male 817 broilers were reared in a floor litter system. At the market age of 50 d, eighteen healthy 817 broilers were slaughtered and the left *pectoralis major* muscle sample from each bird was collected for RNA-seq sequencing. The birds were then plucked and eviscerated and the whole breast muscle was removed and weighed. Breast muscle yield was calculated as the ratio of the breast muscle weight to the eviscerated weight. To identify the co-expression networks and hub genes contributing to breast muscle yield in chickens, we performed weighted gene co-expression network analysis (WGCNA) based on the 18 transcriptome datasets of *pectoralis major* muscle from eighteen 817 broilers.

**Results:**

The WGCNA analysis classified all co-expressed genes in the *pectoral muscle* of 817 broilers into 44 modules. Among these modules, the turquoise and skyblue3 modules were found to be most significantly positively (*r* = 0.78, p = 1e–04) and negatively (*r* = −0.57, p = 0.01) associated with breast meat yield, respectively. Further analysis identified several hub genes (e.g., *DLX3*, *SH3RF2*, *TPM1*, *CAV3*, *MYF6*, and *CFL2*) that involved in muscle structure and muscle development were identified as potential regulators of breast meat production.

**Conclusion:**

The present study has advanced our understanding of the molecular regulatory networks contributing to muscle growth and breast muscle production and will contribute to the molecular breeding of chickens in the future.

## INTRODUCTION

Chickens are one of the most important sources of high-quality animal protein for humans, accounting for ~35% of the meat consumed worldwide [1]. As a major component of edible tissue, breast muscle is the most valuable part of the chicken carcass [2]. To meet the rapidly growing demand for chicken meat, poultry breeders have made great progress in genetic selection to improve the growth rates and breast muscle mass [3]. After intensive artificial selection over the past few decades, the breast meat yield of chickens has increased substantially and now accounts for more than one-fifth of the live weight [4]. Muscle fibers are the functional units of skeletal muscle, and muscle mass is mainly determined by muscle fiber diameter and density [5]. Typically, the number of myofiber is determined at the embryo phase, and the increase in meat mass is the result of the lengthening and enlargement of the myofiber [6].

Genetics is the dominant factor controlling muscle development and growth in chickens [7]. Heritability estimates have shown that the heritability of breast muscle yield ranges from 0.32 to 0.57 [8,9], indicating that breast meat production in broilers can be increased through genetic selection [9]. As an important economic trait for poultry, it is essential to uncover the genetic basis contributing to breast meat production in order to implement effective genetic improvement programmes. In recent years, a larger number of quantitative trait loci (QTL) and candidate genes associated with the breast muscle production trait have been identified using different strategies such as marker-QTL linkage analysis and genome wide association study. In the chicken QTL database there are 56 QTLs associated with breast muscle yield. ( https://www.animalgenome.org/cgi-bin/QTLdb/GG/traitmap?trait_ID=2175 ). For example, genome-wide linkage analysis based on 269 microsatellite markers from 2 unique F2 resource populations derived from broiler-leghorn cross (n = 417) and broiler-fayoumi cross (n = 325) revealed that 5 QTL within GGA2, 8, 9, 12, Z and 4 QTL within GGA2, 7, 8, 9 were significantly associated with breast muscle yield in 2 F2 resource populations, respectively [10]. However, only 2 QTL on GGA17 and GGA18 were detected for breast meat yield in a slow-growing chicken breed based on the Illumina chicken single nucleotide polymorphism (SNP) 60 K beadchip dataset of 836 birds using 3 different methods [11]. Integrating of genome-wide association studies and selection signatures identified 13 genes, including forkhead box O1 (*FOXO1*) and karyopherin subunit alpha 3 (*KPNA3*), as candidates for pectoral muscle mass in an F2 chicken population derived from a cross between Arbor Acres broilers and Baier layers [12]. Based on 45,548 SNP markers from 724 Beijing-You chickens, genome-wide association analyses showed that 15 markers located within or near five genes, including tRNA methyltransferase 11 (*TRMT11*), fatty acid binding protein 7 (*FABP7*), and gap junction protein, alpha 1 (*GJA1*), were significantly associated with breast muscle yield [13]. These results suggest that the trait of breast meat production is affected by polygenic and regulated by a complex network. Although the above studies have advanced our understanding of the genetic basis of the trait of breast meat production, however, our knowledge of the molecular network involved in this trait remains largely unknown.

In China, three main meat-type genotypes of chicken including indigenous Chinese breeds, introduced commercial broilers, and crossbreds of broilers and layers, are widely reared [14]. Among the three main broiler genotypes, the crossbred broiler contributes to 30% of Chinese consumption [15]. The 817 broiler is bred by a cross between Arbor Acres broiler and Hyline brown [16]. Due to its higher growth rate and feed efficiency, the 817 broiler has become the dominant crossbred chicken in China [16]. Particularly, as a fast growth broiler, the 817 broilers exhibited a significantly higher breast muscle yield than that of Chinese indigenous breeds, providing an excellent model to study the specific molecular network contributing to the breast meat yield of broilers.

Weighted gene co-expression network analysis (WGCNA) is a powerful method for identifying the key co-expression gene networks and hub gene associations with phenotypic data based on sample RNA expression datasets [17]. WGCNA is efficient for the study of complex traits affected by multiple regulators. Using this strategy, many specific molecular processes underlying economically important traits in chickens have been illuminated. For example, using breast muscle transcriptome data from the Gushi chicken, WGCNA analyses identified six modules and six key regulators that contribute to fatty acid composition in breast muscle of chickens [18]. Based on a time course of liver transcriptomic data, WGCNA analyses identified four crucial modules and several hub genes are associated with chicken abdominal fat weight [19]. In this study, we measured the breast meat yield of 817 broilers and based on 18 *pectoralis major* muscle RNA-Seq datasets, WGCNA was used to identify gene modules and hub genes associated with the breast muscle production in broilers. Our results will improve our understanding of the molecular regulation of breast muscle production and will contribute to the molecular breeding of chickens in the future.

## MATERIALS AND METHODS

### Ethical approval of the study

The study protocol was approved by the Animal Care and Use Committee of Anhui Agricultural University (Heifei, China. Permit number: SYXK (WAN) 2021-009).

### Birds, sampling, and data collection

In total, 150 1-day-old male 817 broilers (a fast-growing white feather broiler) were purchased and reared under a floor litter system. All birds had free access to water and food throughout the experimental period. The temperature was maintained at 34°C to 35°C for 1 to 4 d and 32°C to 33°C for 5 to 7 d, then decreased by 2°C–3°C per week to 20°C–21°C and maintaining a constant temperature. All broilers were fed a fast-growing broiler diet containing 21.1% crude protein (CP) and 12.55 MJ/kg of metabolizable energy (ME) for 1 to 3 weeks, 20.99% CP and12.13 MJ/kg of ME for 4 to 5 weeks, and 19.57% CP with 13.13 MJ/kg of ME thereafter. At the market age of 50 d, there were 147 experimental birds remaining, and the body weight (BW) of each bird was recorded after 12 h of fasting. The BW of all experimental birds is presented in Supplementary Table S1. Eighteen healthy 817 broilers were randomly selected to measure breast muscle yield and the data are presented in Supplementary Table S2. The slaughtering procedure was similar to our previous study [20]. Briefly, the selected birds were stunned by electric shock followed by exsanguination. After euthanasia, samples of the left *pectoralis major* muscle were snap-frozen in liquid nitrogen and stored at −80°C until further analysis. The birds were then plucked and eviscerated, and the whole breast muscle was removed, weighed, and proportioned to the eviscerated weight.

### RNA extraction, library construction and RNA sequencing

Total RNA was extracted from 18 *pectoralis major* tissues of 817 broilers using trizol method according to the manufacturer’s instructions (Invitrogen, Carlsbad, CA, USA). The concentration and integrity of the total RNA were checked using a NanoDrop 2000 spectrophotometer (Termo Scientific, Wilmington, DE, USA) and by agarose gel electrophoresis. After quality assessment, the RNA was used for cDNA library construction as described in our previous study [20]. RNA-sequencing was performed on an Illumina NovaSeq 6000 platform and 150-bp paired-end reads were generated. The sequencing data have been deposited in the NCBI Sequence Read Archive (SRA) under Bioproject: PRJNA1033035.

### mRNA expression analysis

For the quality control of the sequencing data, adapter sequences and low-quality data were filtered using trim_galore with the parameters ‘-q 20 --phred33 --stringency 3’. After quality control, clean reads were aligned to the chicken genome reference (GRCg7b) using HISAT2 [21]. Sam files were converted to bam files, sorted and indexed using Samtools [22]. The htseq-count script [23] was designed to count the number of reads or read pairs attributable to different genes and transcripts with a count >1 were considered to be expressed. Normalisation of gene read counts was performed using the ‘varianceStabilizingTransformation’ function of DESeq2 [24], and genes with read counts <1 in more than 16 of the samples were excluded from the WGCNA analysis.

### Construction of weighted gene co expression network

A total of 18 RNA-seq datasets were used for the WGCNA analysis. WGCNA was performed using the WGCNA package (v1.7.1) [17] in R software. After checking, no outlier samples were found by clustering the samples, and all 18 samples were retained. The soft threshold power β (β = 1 to 20) was screened to form a scale-free network in both dataset matrices using the “pickSoftThreshold” function in the WGCNA software package. Gene coexpression networks were constructed using the cutreeDynamamic function in the WGCNA with parameters “power = 10, minModuleSize = 30, deepSplit = 2, pamRespectsDendro = F”, and use MEDissThres = 0.3 to merge these modules. The correlation between each module eigengene of the consensus modules and breast muscle yield was calculated using the correlation analysis function in the WGCNA package, and significant consensus modules were identified with the threshold (p≤ 0.05). For the selected modules, the top 150 genes ranked by the kME absolute value were used to gene interaction network analysis using Cytoscape software [25].

### Quantitative real-time polymerase chain reaction analysis

The Wannan chicken is an indigenous chicken breed in China that has a lower breast muscle yield than the 817 broilers, making it an excellent model to test the relationship between hub gene expression levels and breast muscle yield. To verify whether the hub genes are involved in breast muscle production, fifteen Wannan chickens (sampled at market age of 112 d) and fifteen 817 broiler breast muscle samples were selected for quantitative polymerase chain reaction (qPCR) analysis. An ABI Prism 7500 instrument was used for qPCR analysis (Applied Biosystems, Carlsbad, CA, USA). Specific primers for 6 selected genes are shown in Supplementary Table S3. Relative gene expression was measured using the 2^−ΔΔCt^ method with normalisation to the glyceraldehyde-3-phosphate dehydrogenase (*GAPDH*) gene [26].

### Functional enrichment analysis

Functional enrichment analysis for gene ontology (GO) and Kyoto encyclopedia of genes and genomes (KEGG) was performed using the online tool g: Profiler [27]. The significance threshold for GO and KEGG functional categories was defined as Benjamini-Hochberg false discovery rate (FDR) p-value <0.05.

### Statistical analysis

We performed t-tests using SPSS26 software (SPSS Inc., Chicago, IL, USA) with a significance threshold set at p< 0.05 to compare the breast muscle yield and qRT-PCR quantitative expression data between the 817 broilers, and Wannan chickens.

## RESULTS

### Summary of RNA-seq data

A total of 18 *pectoralis major* muscle RNA-seq data were analyzed in this study. The quality control parameters for the RNA-seq data are shown in Supplementary Table S4. The Q20 (phred score 20) and Q30 score were exceeded 97.16% and 92.75%, respectively (Supplementary Table S4). After mapping to the chicken reference genome (GRCg7b), the percentage of mapped reads ranged from 84.25% to 94.07% and the number of expressed genes ranged from 15,305 to 17,817 in each library (Supplementary Table S4).

### Co-expression network construction and modules detection

**Figure 1:**
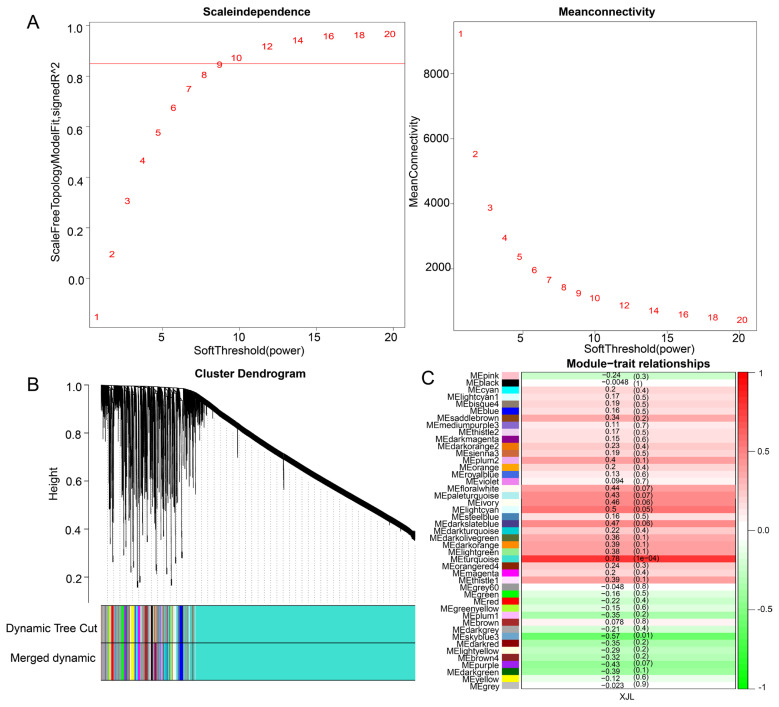
Weighted gene co-expression network analysis of the 18 *pectoralis major* muscle transcriptome datasets. (A) Scale-free topology model and mean connectivity. The fit index curve indicates that a soft threshold power above 10 meets scale–free topology above 0.85. (B) Clustering of co-expression modules. 44 modules, represented by colors, were found using a merging threshold of 0.30. (C) Heat map of module-trait relationships. Each cell contains the matching correlation and p-value. XJL, breast muscle yield.

Co-expression networks allow analysis of gene expression variation associated with multiple genetic traits. We performed a weighted gene co-expression network analysis with 22,148 genes identified from the transcriptomic data to integrate the module eigengene relationship with breast muscle yield. As the *R*^2^ of the scale-free network was greater than 0.85, a soft threshold of 10 was chosen (Figure 1A). After dynamic tree trimming, a total of 44 co-expression modules were detected (Figure 1B). Among these modules, the turquoise module is significantly positively (*r* = 0.78, p = 1e–04) associated with breast muscle yield, while the skyblue3 module is negatively (*r* = −0.57, p = 0.01) related to breast muscle yield of 817 broilers (Figure 1C). We performed enrichment analysis for genes in the turquoise, and skyblue3 modules. A total of 950 GO categories were enriched in the turquoise module, such as “muscle cell development”, “skeletal muscle tissue development” and “skeletal muscle fiber development” (Supplementary Table S5). There was only 1 GO category (Supplementary Table S6), named “motile cilium assembly” that was significantly enriched in the skyblue3 module.

### Identification of hub genes associated with breast muscle yield

**Figure 2:**
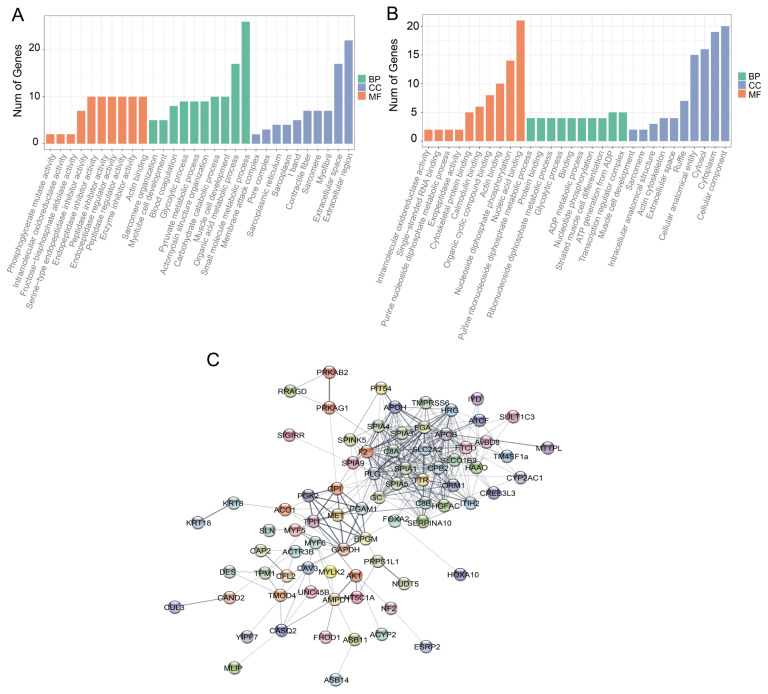
Gene interaction network and functional enrichment analysis diagram of the hub genes in the turquoise module. (A) The top 10 significantly enriched molecular function (MF), cellular component (CC) and biological process (BP) of the top 150 hub genes in the turquoise module. (B). The top 10 significantly enriched MF, CC, and BP of the top 30 hub genes in the turquoise module. (C) Network relationship diagram of the top 150 hub genes in the turquoise module.

The turquoise and skyblue3 modules were identified most significant positive and negative correlations with breast muscle production. We further quantified the kME values for each gene and the top 150 hub genes were collected by ranking the |kME| value in both turquoise and skyblue3 modules (Supplementary Table S7). The skyblue3 module contains 56 annotated genes, including distal-less homeobox 3 (*DLX3*) and SH3 domain containing ring finger 2 (*SH3RF2*) (Supplementary Table S8). The hub genes in the skyblue3 module were significantly enriched in two categories including “monoamine:proton antiporter activity” and “monoamine transmembrane transporter activity” (Supplementary Table S9). In total, 134 genes were annotated among the 150 hub genes in the turquoise module (Supplementary Table S10), and these hub genes were enriched in many categories (Figure 2A; Supplementary Table S11). For example, 10 genes including transmembrane protein 182 (*TMEM182*), tropomyosin 1 (*TPM1*), synaptophysin like 2 (*SYPL2*), calsequestrin 2 (*CASQ2*) and caveolin 3 (*CAV3*), are significantly enriched in the GO terms “muscle cell development” and “muscle cell differentiation”. Several genes including SH3 and cysteine rich domain 3 (*STAC3*), myogenic factor 6 (*MYF6*), MYF5, cofilin 2 (*CFL2*), myosin light chain kinase 2 (*MYLK2*), unc-45 myosin chaperone B (*UNC45B*) and bardet-biedl syndrome 5 (*BBS5*) are significantly enriched in “skeletal muscle tissue development” and “skeletal muscle fiber development” (Figure 2A; Supplementary Table S11). With further gene network analysis combined with the top 30 hub genes ranked by absolute value of kME (Supplementary Table S12), we identified 4 genes (*TPM1*, *CAV3*, *MYF6*, and *CFL2*) that are involved in muscle development and structure (Figure 2B; Supplementary Table S13) and may be crucial regulators contribute to breast muscle yield (Figure 2C).

### Verification the relationship between expression levels of hub genes and breast muscle yield

**Figure 3:**
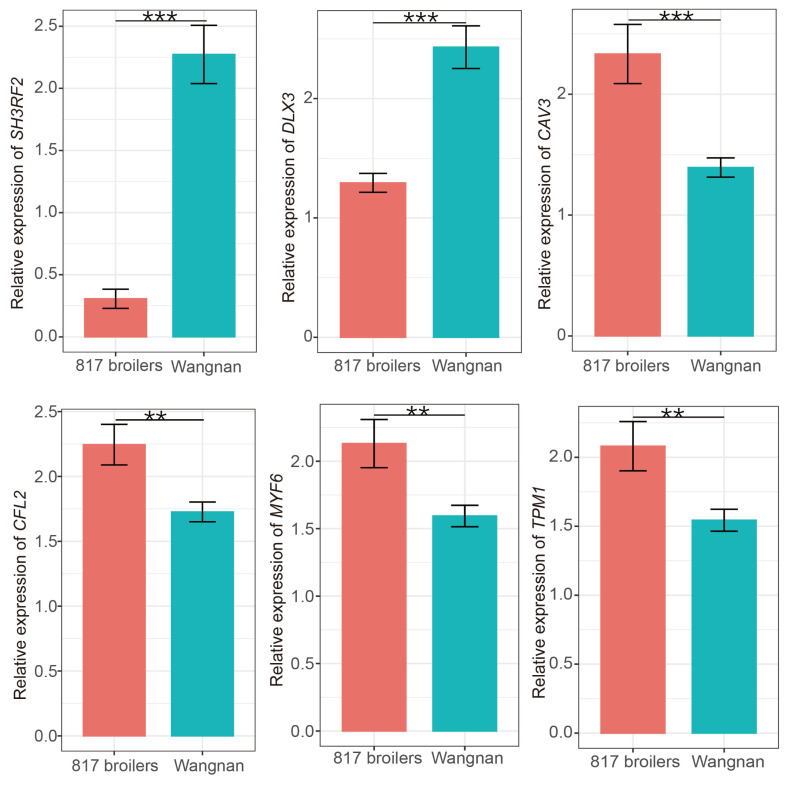
Quantitative polymerase chain reaction (qPCR) validation of gene expression patterns of six hub genes between the 817 broilers and Wannan chickens. Data analysis was performed using t-tests. *** p<0.001, ** p<0.01.

We selected 6 hub genes from the turquoise (*TPM1*, *CAV3*, *MYF6*, and *CFL2*) and from the skyblue3 module (*DLX3* and *SH3RF2*) and evaluated the mRNA levels of these hub genes in the *pectoralis major* of 817 broiler and Wannan chickens to verify the relationship between the expression levels of these hub genes and breast muscle yield. As a broiler breed, the breast muscle yield from 817 broilers was significantly higher than that of Wannan chickens (Supplementary Table S14). The mRNA levels of *TPM1*, *CAV3*, *MYF6*, and *CFL2* were significantly higher, whereas the mRNA levels of *DLX3* and *SH3RF2* were significantly lower in the *pectoralis major* muscle of 817 broilers than that of Wannan chickens (Figure 3). Further correlation analysis shows that the mRNA levels of *TPM1*, *CAV3*, *MYF6* and *CFL2* were significantly positively correlated (*R*^2^ range between 0.63 and 0.76; p<0.05 or p<0.01) and the mRNA levels of *DLX3* and *SH3RF2* were significantly negatively correlated (*R*^2^ = −0.71, p<0.01 and *R*^2^ = −0.62, p<0.05, respectively) with breast muscle yield of 817 broilers (Supplementary Table S15). These results suggest that these hub genes may affect the breast muscle yield of chickens.

## DISCUSSION

Skeletal muscle makes up most chicken meat production and is one of the most important tissues in maintaining locomotion and energy metabolism functions [28]. To meet the ever-increasing consumer demand for chicken meat, intensive genetic selection has been made by breeders in recent decades to improve skeletal muscle mass in broilers [3]. Breast muscle is the most valuable part of the chicken carcass [4]. Therefore, increasing breast meat production is one of the main objectives of the broiler industry. However, breast meat production is a quantitative trait that is polygenically influenced and regulated by a complex network and a comprehensive understanding of the genetic basis and regulatory network that contributing to breast meat yield is essential for poultry breeding. Muscle fibres, which develop from myogenic precursor cells called myoblasts, are the basic unit of muscle [29]. The myoblasts proliferation and differentiation to become myotubes and finally muscle fibres [30]. Skeletal muscle satellite cells, which fuse with existing fibres to cause muscle hypertrophy, are the main contributors to postnatal muscle growth in birds [30]. In this study, WGCNA analysis was performed on 18 *pectoralis major* muscle RNA-seq datasets from 817 broilers to uncover hub genes and network that involved in breast meat mass. The WGCNA analysis classified all co-expressed genes into 44 modules. Among these modules, the turquoise and skyblue3 modules were most significantly positively and negatively related to breast meat yield. Combined with hub genes and qPCR analysis, we identified several genes in the turquoise (*TPM1*, *CAV3*, *MYF6*, and *CFL2*) and in the skyblue3 modules (*DLX3* and *SH3RF2*) that may play a central role in breast muscle yield. However, the hub genes identified associated with breast muscle yield are unique to our study and didn’t overlap with previous studies [10–12]. This is understandable as breast muscle yield is a quantitative trait and is affected by multiple genes with minor individual effects, and it is common for previous studies to identify different candidate genes or QTL for breast muscle yield by using different genetic strategies and different resource populations [12,13].

Among the top 150 hub genes in the skyblue3 module, only 56 genes were annotated. We surveyed the published literature to categorise the function of the annotated hub genes in the skyblue3 module and found that two genes, including *DLX3* and *SH3RF2* may play an important role in muscle development. *DLX3* belongs to the Dlx family of homeodomain transcription factors, which plays important roles in the regulation of myogenesis [31]. In C2C12 myoblast cells, overexpression of *DLX3* markedly downregulated the expression levels of myogenic transcription factors such as *MYOD1*, *MYOG*, *MYF5*, and significantly reduced myotube formation [31]. Furthemore knockdown *DLX3* enhanced expression levels of *MYOD1*, *MYOG*, *MYF5*, and increased the number of myotubes and nuclei per myotube [31]. *SH3RF2* has been mapped to chromosome 13 and encodes the SH3 domain containing ring finger 2, which is expressed in brain and muscle [32]. *SH3RF2* is located within a QTL region for BW, and a deletion mutation in this gene was positively associated with BW and breast muscle weight [33]. We further performed qPCR analysis to verify whether the mRNA levels of *DLX3* and *SH3RF2* were related to breast muscle yield and found that the chickens with higher breast muscle yield had lower expression levels of both *DLX3* and *SH3RF2* genes. Our results suggest that both *DLX3* and *SH3RF2* may be negative regulators of myogenic activity.

We focussd on the turquoise module because many hub genes in the turquoise module were involved in muscle structure and muscle development. Remarkably, we identified 4 genes (*TPM1*, *CAV3*, *MYF6*, and *CFL2*) that may be crucial regulators contributing to breast muscle yield. Tropomyosin (TMs) are a family of highly conserved proteins, that are an integral part of most actin filaments in animals [34]. TMs play a critical role in binding to the troponin complex and control myosin head access to actin in a Ca^2+^-dependent manner [35]. *TPM1*, a member of tropomyosins family, is highly expressed in skeletal and cardiac muscles [36]. In birds, TPM1 plays an important role in the formation of myotubes and mature myofibrils, which is crucial for muscle development [36]. The caveolin gene family contains three subtypes, including *CAV1*, *CAV2*, and *CAV3*, which are the major components of caveolae. *CAV1* and *CAV2* are ubiquitously expressed, whereas *CAV3* is predominantly expressed in skeletal muscle [37]. *CAV3* contributes to the proper differentiation of myoblasts and the homeostasis of myofibers and its expression level increases during the development of muscle cells [38]. In C2C12 myoblasts, overexpression of *CAV3* inhibits of myostatin (*MSTN*) activity and increases cell diameter [39]. Moreover, knockdown of *CAV3* in myoblast cultures results in inhibition of myoblast differentiation [40]. CFL2 is a skeletal muscle-specific actin-binding protein that belongs to the actin depolymerizing factor (ADF)/cofilin family, which includes CFL1, CFL2, and ADF [29]. In C2C12 cells, *CFL2* knockdown markedly downregulated the protein expression of myogenic transcription factors and impaired the differentiation and myotube formation of C2C12 myoblasts [41]. In mice, *CLF2* knockout resulted in progressive disruption of the sarcomeric architecture and abnormal accumulation of F-actin in skeletal muscle [42]. These results suggest that *CFL2* is an essential mediator of myoblast proliferation and differentiation. Myogenic factors 6 (*MYF6*) belong to the family of myogenic regulatory factor (MRF) family, which also includes *MYF5*, *MYOD*, and *MYOG* (myogenin) [43]. MYF6 has been implicated in the differentiation of myotubes, the maturation of muscle fibres and the maintenance of their differentiated state [44]. MYF6 is the primary myogenic factor that remains expressed in the fully differentiated muscle fibre [45]. Compared with Wannan chickens, the mRNA levels of *TPM1*, *CAV3*, *MYF6*, *CFL2* were up-regulated in the *pectoralis major* muscles of 817 broilers, suggesting that these genes may contribute to higher breast muscle yield in 817 broilers.

In conclusion, based on the 18 transcriptome datasets of *pectoralis major* muscles from eighteen 817 broilers, the WGCNA analysis classified all co-expressed genes in the *pectoral muscle* of 817 broilers into 44 modules. Among these modules, the turquoise and skyblue3 modules were found to be most significantly positively and negatively associated with breast meat yield. In particular, several hub genes (e.g. *DLX3*, *SH3RF2*, *TPM1*, *CAV3*, *MYF6*, and *CFL2*) involved in muscle structure and development have been identified as putative regulators of breast meat production. These results will improve our understanding of muscle growth and development and will contribute to future genetic breeding for the improvement of chicken meat production.

## SUPPLEMENTARY MATERIAL

The Supplemental Tables S1–15 were deposited in Figshare doi:https://doi.org/10.6084/m9.figshare.24911187
