# Supplementary material for: Weighted gene co-expression network analysis identifies important modules and hub genes involved in the regulation of breast muscle yield in broilers
Source: Anim Biosci. 2024 Apr 25;37(10):1673–82. doi: 10.5713/ab.23.0548 (PMC11366510; doi:10.5713/ab.23.0548)
Supplement: Supplementary file 1 [file ab-23-0548-Supplementary-Table-1.pdf]

Table S1. Body weight for 147 experiment birds at the market age of 50 d.

Sample	Body weight (g)
Brolier 1	1756.6
Brolier 2	1882.4
Brolier 3	2025.1
Brolier 4	1940.5
Brolier 5	1827.3
Brolier 6	1947.0
Brolier 7	2066.4
Brolier 8	1933.5
Brolier 9	2087.1
Brolier 10	1712.8
Brolier 11	1729.3
Brolier 12	1968.2
Brolier 13	1766.3
Brolier 14	1951.8
Brolier 15	1970.7
Brolier 16	1870.5
Brolier 17	2019.6
Brolier 18	1818.9
Brolier 19	1922.9
Brolier 20	2069.3
Brolier 21	2082.5
Brolier 22	1821.7
Brolier 23	1820.4
Brolier 24	1922.9
Brolier 25	2078.6
Brolier 26	2081.6
Brolier 27	1886.9
Brolier 28	1968.2
Brolier 29	1837.7
Brolier 30	1814.8
Brolier 31	1937.4
Brolier 32	1920.7
Brolier 33	2092.5
Brolier 34	1760.5
Brolier 35	2060.0
Brolier 36	1835.4
Brolier 37	2067.8
Brolier 38	1988.7
Brolier 39	1916.1
Brolier 40	2083.4
Brolier 41	1902.9
Brolier 42	1886.0
Brolier 43	1701.6
Brolier 44	2004.0
Brolier 45	2026.8
Brolier 46	2078.7
Brolier 47	1808.7
Brolier 48	1998.8
Brolier 49	1902.9
Brolier 50	1813.6
Brolier 51	1918.8
Brolier 52	1926.6
Brolier 53	2044.4
Brolier 54	1805.1

Brolier 55	1968.9
Brolier 56	1787.2
Brolier 57	1995.6
Brolier 58	1722.8
Brolier 59	2029.8
Brolier 60	1961.9
Brolier 61	1982.5
Brolier 62	2049.5
Brolier 63	1844.5
Brolier 64	1972.1
Brolier 65	2006.9
Brolier 66	1899.7
Brolier 67	2028.6
Brolier 68	1813.9
Brolier 69	1729.0
Brolier 70	1839.9
Brolier 71	2092.9
Brolier 72	1761.1
Brolier 73	1799.2
Brolier 74	1846.9
Brolier 75	1969.1
Brolier 76	2049.4
Brolier 77	1826.6
Brolier 78	1835.6
Brolier 79	1798.8
Brolier 80	1859.5
Brolier 81	1948.2
Brolier 82	1995.6
Brolier 83	2053.5
Brolier 84	1884.2
Brolier 85	1933.4
Brolier 86	1795.1
Brolier 87	1781.8
Brolier 88	1879.2
Brolier 89	1847.7
Brolier 90	2065.0
Brolier 91	1758.9
Brolier 92	2087.1
Brolier 93	2000.0
Brolier 94	2028.0
Brolier 95	1809.0
Brolier 96	1869.4
Brolier 97	1727.1
Brolier 98	1800.0
Brolier 99	1989.4
Brolier 100	1890.1
Brolier 101	1824.6
Brolier 102	1892.6
Brolier 103	2061.2
Brolier 104	1904.4
Brolier 105	2011.6
Brolier 106	2043.9
Brolier 107	1944.5
Brolier 108	1688.6
Brolier 109	1985.1
Brolier 110	2083.5

Brolier 111	1874.6
Brolier 112	1718.5
Brolier 113	1810.1
Brolier 114	1985.2
Brolier 115	2100.1
Brolier 116	1894.7
Brolier 117	1720.0
Brolier 118	1902.9
Brolier 119	1769.2
Brolier 120	1926.1
Brolier 121	2004.5
Brolier 122	1798.2
Brolier 123	1843.5
Brolier 124	2018.6
Brolier 125	1770.1
Brolier 126	1765.4
Brolier 127	1788.1
Brolier 128	1898.7
Brolier 129	1974.3
Brolier 130	1761.3
Brolier 131	1827.7
Brolier 132	1765.3
Brolier 133	1757.5
Brolier 134	1849.4
Brolier 135	2075.8
Brolier 136	1886.8
Brolier 137	1739.5
Brolier 138	1719.9
Brolier 139	1746.5
Brolier 140	1844.4
Brolier 141	1759.8
Brolier 142	1750.7
Brolier 143	1966.5
Brolier 144	1764.5
Brolier 145	1780.9
Brolier 146	1827.4
Brolier 147	2027.1
