# Supplementary material for: Weighted gene co-expression network analysis identifies important modules and hub genes involved in the regulation of breast muscle yield in broilers
Source: Anim Biosci. 2024 Apr 25;37(10):1673–82. doi: 10.5713/ab.23.0548 (PMC11366510; doi:10.5713/ab.23.0548)
Supplement: Supplementary file 2 [file ab-23-0548-Supplementary-Table-2.pdf]

Table S2. The breast muscle yield and body weight for the 18 selected 817 broilers.

Sample ID	Breast muscle yield (%)	Body weight (g)
T01	20.52	2069.3
T02	18.75	1795.1
T03	19.93	1902.9
T04	18.33	1844.5
T05	19.28	1870.5
T06	19.15	1874.6
T07	18.55	1882.4
T08	20.28	2043.9
T09	17.74	1805.1
T10	19.72	1920.7
T17	18.81	1933.4
T18	19.11	1948.2
T19	18.35	1769.2
T20	19.94	1985.1
T21	18.71	1894.7
T22	19.14	2061.2
T23	18.97	1968.2
T24	20.77	2087.1
