# Supplementary material for: Weighted gene co-expression network analysis identifies important modules and hub genes involved in the regulation of breast muscle yield in broilers
Source: Anim Biosci. 2024 Apr 25;37(10):1673–82. doi: 10.5713/ab.23.0548 (PMC11366510; doi:10.5713/ab.23.0548)
Supplement: Supplementary file 3 [file ab-23-0548-Supplementary-Table-3.pdf]

Table S3. qPCR primers for the six hub genes.

Gene Name	Sequence of primer(5’-3’)	Product scize (bp)
TPM1	tgaggaagagttggatcggg	213
	gccacctcttcatacttgcg	
CAV3	accttcaccgtcagcaagta	246
	aaccctgacgttactgcaga	
MYF6	aagaggcggtggagaaatga	160
	ggtggtctgtgggtcaaaac	
CFL2	tgcatgaaatctcgcagctc	226
	caaaacatgtccccagtgca	
DLX3	ggaaacccaagaagatccgc	204
	cgccgttcttgtagagcttc	
SH3RF2	acttcgacctggctttctga	161
	agtccttgctgtgctctcat	
GAPDH	ctacacacggacacttcaag	244
	acaaacatgggggcatcag	
