# Supplementary material for: Weighted gene co-expression network analysis identifies important modules and hub genes involved in the regulation of breast muscle yield in broilers
Source: Anim Biosci. 2024 Apr 25;37(10):1673–82. doi: 10.5713/ab.23.0548 (PMC11366510; doi:10.5713/ab.23.0548)
Supplement: Supplementary file 4 [file ab-23-0548-Supplementary-Table-4.pdf]

Table S4. Summary of RNA-seq data.

Sample ID	Obtained Reads	OBtained Base(bp)	Q20(%)	Q30(%)	Mapped ratio	Expressed Gene Numbers
T01	22,398,081	6,590,708,822	99.23	96.95	0.8507	15447
T02	26,474,502	7,917,689,566	97.16	95.37	0.855	16316
T03	23,722,541	7,093,886,418	98.13	94.92	0.85	15754
T04	25,428,440	7,591,598,618	98.21	95.29	0.8692	16997
T05	23,348,002	6,983,154,724	98.3	95.5	0.8582	16136
T06	23,971,663	7,165,810,160	98.16	95.19	0.8498	16670
T07	24,846,611	7,429,548,126	98.29	95.48	0.8425	15305
T08	26,272,074	7,857,050,174	98.28	95.45	0.8566	16705
T09	25,759,409	7,688,277,382	98.37	95.58	0.8617	16635
T10	24,278,097	7,255,658,700	98.27	95.37	0.8628	16758
T17	19,782,100	5,905,567,802	98.04	94.41	0.93914372	17188
T18	23,840,259	7,131,208,736	97.36	92.75	0.93692517	17272
T19	24,932,030	7,455,643,004	97.76	93.85	0.93257957	17430
T20	27,508,474	8,221,256,254	97.68	93.75	0.93303511	17817
T21	22,378,894	6,685,088,132	97.55	93.37	0.93574809	17402
T22	27,522,043	8,200,374,676	97.7	93.83	0.93281403	17707
T23	22,676,648	6,789,175,820	97.85	94.01	0.94066911	17591
T24	20,904,521	6,245,592,412	97.38	92.94	0.93140144	17208
