# Supplementary material for: Weighted gene co-expression network analysis identifies important modules and hub genes involved in the regulation of breast muscle yield in broilers
Source: Anim Biosci. 2024 Apr 25;37(10):1673–82. doi: 10.5713/ab.23.0548 (PMC11366510; doi:10.5713/ab.23.0548)
Supplement: Supplementary file 5 [file ab-23-0548-Supplementary-Table-5.pdf]

**Table S5. Functional enrichment analysis of the genes in the turquoise module.**

| Category | Term description | Term ID | Adjusted p value |
|---|---|---|---|
| GO:MF | protein binding | GO:0005515 | 6.43E-48 |
| GO:MF | catalytic activity | GO:0003824 | 6.84E-33 |
| GO:MF | binding | GO:0005488 | 1.01E-30 |
| GO:MF | enzyme binding | GO:0019899 | 3.26E-17 |
| GO:MF | transferase activity | GO:0016740 | 6.49E-14 |
| GO:MF | nucleoside phosphate binding | GO:1901265 | 1.74E-12 |
| GO:MF | nucleotide binding | GO:0000166 | 1.74E-12 |
| GO:MF | identical protein binding | GO:0042802 | 3.86E-12 |
| GO:MF | ion binding | GO:0043167 | 1.03E-11 |
| GO:MF | anion binding | GO:0043168 | 2.78E-11 |
| GO:MF | carbohydrate derivative binding | GO:0097367 | 3.06E-11 |
| GO:MF | small molecule binding | GO:0036094 | 8.57E-11 |
| GO:MF | purine nucleotide binding | GO:0017076 | 8.57E-11 |
| GO:MF | ribonucleotide binding | GO:0032553 | 1.44E-09 |
| GO:MF | catalytic activity, acting on a protein | GO:0140096 | 1.63E-09 |
| GO:MF | purine ribonucleoside triphosphate binding | GO:0035639 | 1.85E-09 |
| GO:MF | adenyl nucleotide binding | GO:0030554 | 2.40E-09 |
| GO:MF | purine ribonucleotide binding | GO:0032555 | 4.59E-09 |
| GO:MF | phosphatase activity | GO:0016791 | 6.25E-08 |
| GO:MF | adenyl ribonucleotide binding | GO:0032559 | 1.23E-07 |
| GO:MF | ATP binding | GO:0005524 | 1.23E-07 |
| GO:MF | protein kinase binding | GO:0019901 | 1.99E-07 |
| GO:MF | oxidoreductase activity | GO:0016491 | 3.31E-07 |
| GO:MF | protein-containing complex binding | GO:0044877 | 4.90E-07 |
| GO:MF | kinase binding | GO:0019900 | 6.09E-07 |
| GO:MF | enzyme regulator activity | GO:0030234 | 9.94E-07 |
| GO:MF | phosphoprotein phosphatase activity | GO:0004721 | 1.92E-06 |
| GO:MF | cytoskeletal protein binding | GO:0008092 | 2.14E-06 |
| GO:MF | phosphoric ester hydrolase activity | GO:0042578 | 1.77E-05 |
| GO:MF | acyltransferase activity | GO:0016746 | 2.51E-05 |
| GO:MF | protein domain specific binding | GO:0019904 | 4.50E-05 |
| GO:MF | phosphotransferase activity, alcohol group as acceptor | GO:0016773 | 5.77E-05 |
| GO:MF | hydrolase activity, acting on acid anhydrides | GO:0016817 | 6.37E-05 |
| GO:MF | hydrolase activity, acting on acid anhydrides, in phosphorus-containing anhydrides | GO:0016818 | 6.90E-05 |
| GO:MF | protein homodimerization activity | GO:0042803 | 8.91E-05 |
| GO:MF | pyrophosphatase activity | GO:0016462 | 9.05E-05 |
| GO:MF | ribonucleoside triphosphate phosphatase activity | GO:0017111 | 0.0001422 |
| GO:MF | protein serine/threonine kinase activity | GO:0004674 | 0.0001684 |
| GO:MF | kinase activity | GO:0016301 | 0.0003395 |
| GO:MF | ATP-dependent activity | GO:0140657 | 0.0003755 |
| GO:MF | protein kinase activity | GO:0004672 | 0.0005199 |
| GO:MF | nucleoside-triphosphatase regulator activity | GO:0060589 | 0.0005346 |
| GO:MF | GTPase regulator activity | GO:0030695 | 0.0008505 |
| GO:MF | transferase activity, transferring phosphorus-containing groups | GO:0016772 | 0.0008505 |
| GO:MF | protein tyrosine/serine/threonine phosphatase activity | GO:0008138 | 0.0015209 |
| GO:MF | protein dimerization activity | GO:0046983 | 0.0018046 |
| GO:MF | aminoacyltransferase activity | GO:0016755 | 0.0018181 |
| GO:MF | histone binding | GO:0042393 | 0.0018889 |
| GO:MF | guanyl-nucleotide exchange factor activity | GO:0005085 | 0.0024827 |
| GO:MF | ubiquitin-like protein transferase activity | GO:0019787 | 0.0027675 |
| GO:MF | protein-macromolecule adaptor activity | GO:0030674 | 0.0037102 |

| | | | |
|---|---|---|---|
| GO:MF | cell adhesion molecule binding | GO:0050839 | 0.0037102 |
| GO:MF | phosphatase binding | GO:0019902 | 0.0037837 |
| GO:MF | translation regulator activity, nucleic acid binding | GO:0090079 | 0.0038054 |
| GO:MF | modification-dependent protein binding | GO:0140030 | 0.0038054 |
| GO:MF | oxidoreductase activity, acting on CH-OH group of donors | GO:0016614 | 0.0039328 |
| GO:MF | enzyme activator activity | GO:0008047 | 0.0040782 |
| GO:MF | transcription coregulator activity | GO:0003712 | 0.0045546 |
| GO:MF | magnesium ion binding | GO:0000287 | 0.0048959 |
| GO:MF | ATP hydrolysis activity | GO:0016887 | 0.0048959 |
| GO:MF | protein tyrosine phosphatase activity | GO:0004725 | 0.0051425 |
| GO:MF | transcription factor binding | GO:0008134 | 0.0064557 |
| GO:MF | histone modifying activity | GO:0140993 | 0.0088561 |
| GO:MF | ubiquitin-protein transferase activity | GO:0004842 | 0.0089203 |
| GO:MF | actin binding | GO:0003779 | 0.0098505 |
| GO:MF | flavin adenine dinucleotide binding | GO:0050660 | 0.0105634 |
| GO:MF | molecular adaptor activity | GO:0060090 | 0.0105634 |
| GO:MF | DNA-binding transcription factor binding | GO:0140297 | 0.0119819 |
| GO:MF | oxidoreductase activity, acting on the CH-OH group of donors, NAD or NADP as acceptor | GO:0016616 | 0.0140014 |
| GO:MF | hydrolase activity | GO:0016787 | 0.0229192 |
| GO:MF | translation regulator activity | GO:0045182 | 0.0236966 |
| GO:MF | cation binding | GO:0043169 | 0.0236966 |
| GO:MF | ubiquitin-like protein ligase activity | GO:0061659 | 0.0236966 |
| GO:MF | ubiquitin protein ligase activity | GO:0061630 | 0.0236966 |
| GO:MF | metal ion binding | GO:0046872 | 0.0236966 |
| GO:MF | translation factor activity, RNA binding | GO:0008135 | 0.0246867 |
| GO:MF | protease binding | GO:0002020 | 0.0308363 |
| GO:MF | primary active transmembrane transporter activity | GO:0015399 | 0.0332428 |
| GO:MF | protein heterodimerization activity | GO:0046982 | 0.0348961 |
| GO:MF | translation initiation factor activity | GO:0003743 | 0.0348961 |
| GO:MF | active transmembrane transporter activity | GO:0022804 | 0.0350347 |
| GO:MF | telomeric DNA binding | GO:0042162 | 0.0410808 |
| GO:MF | ribonucleoprotein complex binding | GO:0043021 | 0.0422236 |
| GO:MF | ubiquitin-like protein binding | GO:0032182 | 0.0468246 |
| GO:MF | protein serine/threonine phosphatase activity | GO:0004722 | 0.0468246 |
| GO:MF | iron-sulfur cluster binding | GO:0051536 | 0.0468246 |
| GO:MF | metal cluster binding | GO:0051540 | 0.0468246 |
| GO:MF | carbohydrate derivative transmembrane transporter activity | GO:1901505 | 0.0491267 |
| GO:BP | organonitrogen compound metabolic process | GO:1901564 | 4.50E-46 |
| GO:BP | protein metabolic process | GO:0019538 | 4.89E-39 |
| GO:BP | macromolecule modification | GO:0043412 | 6.09E-36 |
| GO:BP | protein modification process | GO:0036211 | 6.29E-32 |
| GO:BP | positive regulation of biological process | GO:0048518 | 1.11E-26 |
| GO:BP | response to stress | GO:0006950 | 4.82E-24 |
| GO:BP | catabolic process | GO:0009056 | 1.09E-21 |
| GO:BP | positive regulation of cellular process | GO:0048522 | 5.09E-21 |
| GO:BP | cellular localization | GO:0051641 | 2.63E-20 |
| GO:BP | regulation of protein metabolic process | GO:0051246 | 1.31E-19 |
| GO:BP | localization | GO:0051179 | 1.62E-19 |
| GO:BP | intracellular signal transduction | GO:0035556 | 2.14E-19 |
| GO:BP | regulation of response to stimulus | GO:0048583 | 2.92E-19 |
| GO:BP | phosphorus metabolic process | GO:0006793 | 1.08E-17 |
| GO:BP | phosphate-containing compound metabolic process | GO:0006796 | 1.74E-17 |
| GO:BP | organic substance catabolic process | GO:1901575 | 7.86E-17 |
| GO:BP | cellular response to stress | GO:0033554 | 1.22E-16 |
| GO:BP | macromolecule localization | GO:0033036 | 1.27E-16 |

| | | | |
|---|---|---|---|
| GO:BP | primary metabolic process | GO:0044238 | 1.98E-16 |
| GO:BP | regulation of cellular component organization | GO:0051128 | 4.41E-16 |
| GO:BP | cellular macromolecule localization | GO:0070727 | 5.26E-16 |
| GO:BP | cellular component organization or biogenesis | GO:0071840 | 6.61E-16 |
| GO:BP | protein localization | GO:0008104 | 8.61E-16 |
| GO:BP | amide metabolic process | GO:0043603 | 2.68E-15 |
| GO:BP | intracellular transport | GO:0046907 | 4.00E-15 |
| GO:BP | positive regulation of molecular function | GO:0044093 | 5.43E-15 |
| GO:BP | positive regulation of metabolic process | GO:0009893 | 6.35E-15 |
| GO:BP | establishment of localization | GO:0051234 | 1.57E-14 |
| GO:BP | regulation of molecular function | GO:0065009 | 2.26E-14 |
| GO:BP | transport | GO:0006810 | 2.65E-14 |
| GO:BP | macromolecule catabolic process | GO:0009057 | 6.16E-14 |
| GO:BP | nitrogen compound metabolic process | GO:0006807 | 7.16E-14 |
| GO:BP | organonitrogen compound catabolic process | GO:1901565 | 1.34E-13 |
| GO:BP | cellular component organization | GO:0016043 | 2.17E-13 |
| GO:BP | cellular response to chemical stimulus | GO:0070887 | 2.19E-13 |
| GO:BP | small molecule metabolic process | GO:0044281 | 3.42E-13 |
| GO:BP | establishment of localization in cell | GO:0051649 | 3.42E-13 |
| GO:BP | response to chemical | GO:0042221 | 4.74E-13 |
| GO:BP | regulation of primary metabolic process | GO:0080090 | 8.45E-13 |
| GO:BP | regulation of intracellular signal transduction | GO:1902531 | 9.34E-13 |
| GO:BP | organonitrogen compound biosynthetic process | GO:1901566 | 1.27E-12 |
| GO:BP | peptide metabolic process | GO:0006518 | 1.38E-12 |
| GO:BP | response to external stimulus | GO:0009605 | 1.79E-12 |
| GO:BP | amide biosynthetic process | GO:0043604 | 2.19E-12 |
| GO:BP | positive regulation of macromolecule metabolic process | GO:0010604 | 2.54E-12 |
| GO:BP | immune system process | GO:0002376 | 3.96E-12 |
| GO:BP | regulation of nitrogen compound metabolic process | GO:0051171 | 6.19E-12 |
| GO:BP | regulation of catalytic activity | GO:0050790 | 6.84E-12 |
| GO:BP | regulation of response to stress | GO:0080134 | 1.03E-11 |
| GO:BP | establishment of protein localization | GO:0045184 | 2.36E-11 |
| GO:BP | negative regulation of response to stimulus | GO:0048585 | 2.36E-11 |
| GO:BP | positive regulation of cellular metabolic process | GO:0031325 | 2.79E-11 |
| GO:BP | locomotion | GO:0040011 | 2.97E-11 |
| GO:BP | positive regulation of nitrogen compound metabolic process | GO:0051173 | 3.60E-11 |
| GO:BP | protein transport | GO:0015031 | 4.09E-11 |
| GO:BP | regulation of cell motility | GO:2000145 | 4.80E-11 |
| GO:BP | cellular catabolic process | GO:0044248 | 4.98E-11 |
| GO:BP | cell death | GO:0008219 | 5.23E-11 |
| GO:BP | protein catabolic process | GO:0030163 | 5.44E-11 |
| GO:BP | positive regulation of protein metabolic process | GO:0051247 | 5.46E-11 |
| GO:BP | programmed cell death | GO:0012501 | 5.57E-11 |
| GO:BP | regulation of signal transduction | GO:0009966 | 6.37E-11 |
| GO:BP | negative regulation of intracellular signal transduction | GO:1902532 | 6.73E-11 |
| GO:BP | regulation of immune system process | GO:0002682 | 6.73E-11 |
| GO:BP | positive regulation of response to stimulus | GO:0048584 | 6.73E-11 |
| GO:BP | positive regulation of catalytic activity | GO:0043085 | 6.73E-11 |
| GO:BP | response to organic substance | GO:0010033 | 7.35E-11 |
| GO:BP | phosphorylation | GO:0016310 | 7.36E-11 |
| GO:BP | regulation of cell migration | GO:0030334 | 1.00E-10 |
| GO:BP | peptide biosynthetic process | GO:0043043 | 1.20E-10 |
| GO:BP | regulation of protein modification process | GO:0031399 | 1.22E-10 |
| GO:BP | apoptotic process | GO:0006915 | 2.44E-10 |
| GO:BP | response to other organism | GO:0051707 | 2.44E-10 |
| GO:BP | response to biotic stimulus | GO:0009607 | 2.44E-10 |

| | | | |
|---|---|---|---|
| GO:BP | proteolysis involved in protein catabolic process | GO:0051603 | 2.46E-10 |
| GO:BP | biological process involved in interspecies interaction between organisms | GO:0044419 | 2.62E-10 |
| GO:BP | nitrogen compound transport | GO:0071705 | 2.79E-10 |
| GO:BP | protein phosphorylation | GO:0006468 | 3.24E-10 |
| GO:BP | organic substance transport | GO:0071702 | 3.38E-10 |
| GO:BP | response to external biotic stimulus | GO:0043207 | 3.94E-10 |
| GO:BP | post-translational protein modification | GO:0043687 | 4.05E-10 |
| GO:BP | translation | GO:0006412 | 4.14E-10 |
| GO:BP | cellular component biogenesis | GO:0044085 | 4.80E-10 |
| GO:BP | regulation of locomotion | GO:0040012 | 5.25E-10 |
| GO:BP | positive regulation of immune system process | GO:0002684 | 6.14E-10 |
| GO:BP | regulation of catabolic process | GO:0009894 | 6.22E-10 |
| GO:BP | metabolic process | GO:0008152 | 7.36E-10 |
| GO:BP | developmental process | GO:0032502 | 7.49E-10 |
| GO:BP | regulation of multicellular organismal process | GO:0051239 | 1.04E-09 |
| GO:BP | modification-dependent macromolecule catabolic process | GO:0043632 | 1.52E-09 |
| GO:BP | regulation of developmental process | GO:0050793 | 1.54E-09 |
| GO:BP | immune response | GO:0006955 | 1.78E-09 |
| GO:BP | modification-dependent protein catabolic process | GO:0019941 | 2.30E-09 |
| GO:BP | anatomical structure development | GO:0048856 | 2.82E-09 |
| GO:BP | regulation of response to external stimulus | GO:0032101 | 3.03E-09 |
| GO:BP | ubiquitin-dependent protein catabolic process | GO:0006511 | 3.03E-09 |
| GO:BP | organic acid metabolic process | GO:0006082 | 4.48E-09 |
| GO:BP | mitochondrion organization | GO:0007005 | 4.70E-09 |
| GO:BP | regulation of immune response | GO:0050776 | 4.79E-09 |
| GO:BP | regulation of cellular localization | GO:0060341 | 5.63E-09 |
| GO:BP | dephosphorylation | GO:0016311 | 5.68E-09 |
| GO:BP | oxoacid metabolic process | GO:0043436 | 6.35E-09 |
| GO:BP | cellular response to organic substance | GO:0071310 | 7.62E-09 |
| GO:BP | negative regulation of nitrogen compound metabolic process | GO:0051172 | 9.03E-09 |
| GO:BP | defense response to other organism | GO:0098542 | 9.29E-09 |
| GO:BP | negative regulation of signal transduction | GO:0009968 | 1.13E-08 |
| GO:BP | positive regulation of locomotion | GO:0040017 | 1.13E-08 |
| GO:BP | regulation of protein serine/threonine kinase activity | GO:0071900 | 1.26E-08 |
| GO:BP | regulation of signaling | GO:0023051 | 1.37E-08 |
| GO:BP | vesicle-mediated transport | GO:0016192 | 1.38E-08 |
| GO:BP | protein dephosphorylation | GO:0006470 | 1.45E-08 |
| GO:BP | carboxylic acid metabolic process | GO:0019752 | 1.45E-08 |
| GO:BP | positive regulation of cell migration | GO:0030335 | 2.04E-08 |
| GO:BP | positive regulation of cellular component organization | GO:0051130 | 2.06E-08 |
| GO:BP | cell motility | GO:0048870 | 2.22E-08 |
| GO:BP | regulation of cell communication | GO:0010646 | 2.47E-08 |
| GO:BP | cell migration | GO:0016477 | 2.72E-08 |
| GO:BP | positive regulation of cell motility | GO:2000147 | 3.07E-08 |
| GO:BP | negative regulation of protein metabolic process | GO:0051248 | 3.63E-08 |
| GO:BP | hemopoiesis | GO:0030097 | 4.01E-08 |
| GO:BP | negative regulation of biological process | GO:0048519 | 4.20E-08 |
| GO:BP | regulation of phosphorus metabolic process | GO:0051174 | 4.44E-08 |
| GO:BP | regulation of phosphate metabolic process | GO:0019220 | 4.93E-08 |
| GO:BP | regulation of localization | GO:0032879 | 5.83E-08 |
| GO:BP | protein modification by small protein conjugation or removal | GO:0070647 | 5.98E-08 |
| GO:BP | intracellular protein transport | GO:0006886 | 6.28E-08 |
| GO:BP | cell surface receptor signaling pathway | GO:0007166 | 7.70E-08 |
| GO:BP | protein localization to organelle | GO:0033365 | 1.11E-07 |
| GO:BP | regulation of organelle organization | GO:0033043 | 1.21E-07 |

| | | | |
|---|---|---|---|
| GO:BP | DNA damage response | GO:0006974 | 1.33E-07 |
| GO:BP | regulation of amide metabolic process | GO:0034248 | 1.34E-07 |
| GO:BP | regulation of protein localization | GO:0032880 | 1.34E-07 |
| GO:BP | negative regulation of signaling | GO:0023057 | 1.45E-07 |
| GO:BP | regulation of cellular component biogenesis | GO:0044087 | 1.53E-07 |
| GO:BP | cellular component assembly | GO:0022607 | 1.66E-07 |
| GO:BP | cell differentiation | GO:0030154 | 1.80E-07 |
| GO:BP | cellular developmental process | GO:0048869 | 1.80E-07 |
| GO:BP | organelle organization | GO:0006996 | 2.08E-07 |
| GO:BP | cell adhesion | GO:0007155 | 2.25E-07 |
| GO:BP | protein modification by small protein conjugation | GO:0032446 | 2.25E-07 |
| GO:BP | proteasomal protein catabolic process | GO:0010498 | 2.57E-07 |
| GO:BP | regulation of programmed cell death | GO:0043067 | 2.88E-07 |
| GO:BP | negative regulation of cell communication | GO:0010648 | 3.62E-07 |
| GO:BP | regulation of phosphorylation | GO:0042325 | 4.27E-07 |
| GO:BP | negative regulation of cellular process | GO:0048523 | 4.46E-07 |
| GO:BP | cellular lipid metabolic process | GO:0044255 | 4.46E-07 |
| GO:BP | cell activation | GO:0001775 | 5.14E-07 |
| GO:BP | actin filament-based process | GO:0030029 | 6.49E-07 |
| GO:BP | regulation of transferase activity | GO:0051338 | 7.00E-07 |
| GO:BP | positive regulation of hydrolase activity | GO:0051345 | 7.51E-07 |
| GO:BP | positive regulation of catabolic process | GO:0009896 | 7.89E-07 |
| GO:BP | positive regulation of response to external stimulus | GO:0032103 | 1.00E-06 |
| GO:BP | regulation of proteolysis | GO:0030162 | 1.04E-06 |
| GO:BP | positive regulation of immune response | GO:0050778 | 1.12E-06 |
| GO:BP | ameboidal-type cell migration | GO:0001667 | 1.14E-06 |
| GO:BP | immune response-regulating cell surface receptor signaling | GO:0002768 | 1.16E-06 |
| GO:BP | regulation of kinase activity | GO:0043549 | 1.22E-06 |
| GO:BP | response to oxygen-containing compound | GO:1901700 | 1.23E-06 |
| GO:BP | regulation of apoptotic process | GO:0042981 | 1.25E-06 |
| GO:BP | defense response | GO:0006952 | 1.34E-06 |
| GO:BP | carbohydrate metabolic process | GO:0005975 | 1.49E-06 |
| GO:BP | regulation of protein phosphorylation | GO:0001932 | 1.51E-06 |
| GO:BP | actin cytoskeleton organization | GO:0030036 | 1.63E-06 |
| GO:BP | activation of immune response | GO:0002253 | 1.69E-06 |
| GO:BP | proteasome-mediated ubiquitin-dependent protein catabolic process | GO:0043161 | 1.87E-06 |
| GO:BP | regulation of cell adhesion | GO:0030155 | 1.90E-06 |
| GO:BP | negative regulation of molecular function | GO:0044092 | 1.90E-06 |
| GO:BP | carbohydrate derivative metabolic process | GO:1901135 | 2.05E-06 |
| GO:BP | lipid metabolic process | GO:0006629 | 2.14E-06 |
| GO:BP | protein maturation | GO:0051604 | 2.37E-06 |
| GO:BP | epithelial cell migration | GO:0010631 | 2.40E-06 |
| GO:BP | immune response-activating cell surface receptor signaling pathway | GO:0002429 | 2.40E-06 |
| GO:BP | regulation of protein kinase activity | GO:0045859 | 2.74E-06 |
| GO:BP | regulation of hydrolase activity | GO:0051336 | 2.92E-06 |
| GO:BP | organic substance metabolic process | GO:0071704 | 2.93E-06 |
| GO:BP | regulation of cellular catabolic process | GO:0031329 | 3.07E-06 |
| GO:BP | regulation of MAPK cascade | GO:0043408 | 3.13E-06 |
| GO:BP | MAPK cascade | GO:0000165 | 3.55E-06 |
| GO:BP | leukocyte activation | GO:0045321 | 3.57E-06 |
| GO:BP | regulation of cellular response to stress | GO:0080135 | 3.74E-06 |
| GO:BP | generation of precursor metabolites and energy | GO:0006091 | 3.74E-06 |
| GO:BP | tissue migration | GO:0090130 | 3.86E-06 |
| GO:BP | cellular metabolic process | GO:0044237 | 4.01E-06 |
| GO:BP | regulation of biological quality | GO:0065008 | 4.49E-06 |
| GO:BP | protein ubiquitination | GO:0016567 | 4.49E-06 |

| | | | |
|---|---|---|---|
| GO:BP | myeloid cell differentiation | GO:0030099 | 4.62E-06 |
| GO:BP | regulation of intracellular transport | GO:0032386 | 4.62E-06 |
| GO:BP | regulation of defense response | GO:0031347 | 5.03E-06 |
| GO:BP | epithelium migration | GO:0090132 | 5.16E-06 |
| GO:BP | regulation of protein-containing complex assembly | GO:0043254 | 7.12E-06 |
| GO:BP | immune response-regulating signaling pathway | GO:0002764 | 7.15E-06 |
| GO:BP | positive regulation of protein serine/threonine kinase activity | GO:0071902 | 7.21E-06 |
| GO:BP | protein-DNA complex organization | GO:0071824 | 7.22E-06 |
| GO:BP | immune effector process | GO:0002252 | 7.67E-06 |
| GO:BP | response to cytokine | GO:0034097 | 8.50E-06 |
| GO:BP | cellular nitrogen compound biosynthetic process | GO:0044271 | 9.28E-06 |
| GO:BP | regulation of translation | GO:0006417 | 9.39E-06 |
| GO:BP | cellular response to oxygen-containing compound | GO:1901701 | 9.68E-06 |
| GO:BP | apoptotic signaling pathway | GO:0097190 | 9.72E-06 |
| GO:BP | regulation of cell differentiation | GO:0045595 | 1.07E-05 |
| GO:BP | chromatin organization | GO:0006325 | 1.08E-05 |
| GO:BP | positive regulation of biosynthetic process | GO:0009891 | 1.26E-05 |
| GO:BP | protein-containing complex organization | GO:0043933 | 1.52E-05 |
| GO:BP | cytokine production | GO:0001816 | 1.53E-05 |
| GO:BP | immune response-activating signaling pathway | GO:0002757 | 1.71E-05 |
| GO:BP | positive regulation of proteolysis | GO:0045862 | 1.73E-05 |
| GO:BP | multicellular organismal process | GO:0032501 | 2.04E-05 |
| GO:BP | positive regulation of cellular biosynthetic process | GO:0031328 | 2.11E-05 |
| GO:BP | endocytosis | GO:0006897 | 2.15E-05 |
| GO:BP | circulatory system development | GO:0072359 | 2.15E-05 |
| GO:BP | regulation of cell population proliferation | GO:0042127 | 2.25E-05 |
| GO:BP | actin filament organization | GO:0007015 | 2.38E-05 |
| GO:BP | response to lipid | GO:0033993 | 2.60E-05 |
| GO:BP | regulation of cytokine production | GO:0001817 | 2.72E-05 |
| GO:BP | positive regulation of cell adhesion | GO:0045785 | 2.74E-05 |
| GO:BP | positive regulation of protein modification process | GO:0031401 | 2.76E-05 |
| GO:BP | process utilizing autophagic mechanism | GO:0061919 | 2.76E-05 |
| GO:BP | autophagy | GO:0006914 | 2.76E-05 |
| GO:BP | cell cycle | GO:0007049 | 2.76E-05 |
| GO:BP | muscle structure development | GO:0061061 | 2.99E-05 |
| GO:BP | regulation of actin cytoskeleton organization | GO:0032956 | 3.05E-05 |
| GO:BP | muscle cell differentiation | GO:0042692 | 3.33E-05 |
| GO:BP | chromatin remodeling | GO:0006338 | 3.38E-05 |
| GO:BP | supramolecular fiber organization | GO:0097435 | 3.40E-05 |
| GO:BP | negative regulation of protein modification process | GO:0031400 | 3.45E-05 |
| GO:BP | small molecule catabolic process | GO:0044282 | 3.50E-05 |
| GO:BP | homeostatic process | GO:0042592 | 3.59E-05 |
| GO:BP | membrane organization | GO:0061024 | 3.80E-05 |
| GO:BP | enzyme-linked receptor protein signaling pathway | GO:0007167 | 3.93E-05 |
| GO:BP | alcohol metabolic process | GO:0006066 | 4.64E-05 |
| GO:BP | regulation of transport | GO:0051049 | 4.75E-05 |
| GO:BP | regulation of epithelial cell migration | GO:0010632 | 5.04E-05 |
| GO:BP | peptidyl-amino acid modification | GO:0018193 | 5.25E-05 |
| GO:BP | tissue development | GO:0009888 | 5.25E-05 |
| GO:BP | positive regulation of macromolecule biosynthetic process | GO:0010557 | 5.25E-05 |
| GO:BP | regulation of MAP kinase activity | GO:0043405 | 5.28E-05 |
| GO:BP | innate immune response | GO:0045087 | 5.30E-05 |
| GO:BP | regulation of actin filament-based process | GO:0032970 | 5.69E-05 |
| GO:BP | regulation of actin filament organization | GO:0110053 | 5.76E-05 |
| GO:BP | lipid biosynthetic process | GO:0008610 | 5.76E-05 |
| GO:BP | regulation of cell cycle | GO:0051726 | 5.76E-05 |

| | | | |
|---|---|---|---|
| GO:BP | regulation of cell activation | GO:0050865 | 5.78E-05 |
| GO:BP | response to oxidative stress | GO:0006979 | 5.78E-05 |
| GO:BP | ncRNA processing | GO:0034470 | 6.06E-05 |
| GO:BP | proteolysis | GO:0006508 | 6.58E-05 |
| GO:BP | nuclear transport | GO:0051169 | 6.86E-05 |
| GO:BP | monocarboxylic acid metabolic process | GO:0032787 | 7.27E-05 |
| GO:BP | positive regulation of gene expression | GO:0010628 | 7.38E-05 |
| GO:BP | nucleocytoplasmic transport | GO:0006913 | 7.90E-05 |
| GO:BP | regulation of metabolic process | GO:0019222 | 8.07E-05 |
| GO:BP | cell population proliferation | GO:0008283 | 8.20E-05 |
| GO:BP | oxidative phosphorylation | GO:0006119 | 8.52E-05 |
| GO:BP | regulation of multicellular organismal development | GO:2000026 | 8.87E-05 |
| GO:BP | response to abiotic stimulus | GO:0009628 | 9.24E-05 |
| GO:BP | vasculature development | GO:0001944 | 9.27E-05 |
| GO:BP | cellular response to cytokine stimulus | GO:0071345 | 9.47E-05 |
| GO:BP | lymphocyte activation | GO:0046649 | 9.65E-05 |
| GO:BP | aerobic respiration | GO:0009060 | 0.0001117 |
| GO:BP | multicellular organism development | GO:0007275 | 0.0001132 |
| GO:BP | cell development | GO:0048468 | 0.0001163 |
| GO:BP | ncRNA metabolic process | GO:0034660 | 0.0001202 |
| GO:BP | actomyosin structure organization | GO:0031032 | 0.0001279 |
| GO:BP | cellular response to biotic stimulus | GO:0071216 | 0.0001386 |
| GO:BP | positive regulation of phosphorus metabolic process | GO:0010562 | 0.0001453 |
| GO:BP | positive regulation of phosphate metabolic process | GO:0045937 | 0.0001453 |
| GO:BP | response to molecule of bacterial origin | GO:0002237 | 0.0001509 |
| GO:BP | cellular respiration | GO:0045333 | 0.0001522 |
| GO:BP | electron transport chain | GO:0022900 | 0.0001698 |
| GO:BP | regulation of supramolecular fiber organization | GO:1902903 | 0.0001805 |
| GO:BP | phagocytosis | GO:0006909 | 0.000182 |
| GO:BP | regulation of leukocyte activation | GO:0002694 | 0.0002033 |
| GO:BP | glycoprotein metabolic process | GO:0009100 | 0.0002072 |
| GO:BP | positive regulation of protein localization | GO:1903829 | 0.0002073 |
| GO:BP | regulation of lipid metabolic process | GO:0019216 | 0.0002073 |
| GO:BP | sulfur compound metabolic process | GO:0006790 | 0.0002148 |
| GO:BP | negative regulation of response to external stimulus | GO:0032102 | 0.000218 |
| GO:BP | production of molecular mediator of immune response | GO:0002440 | 0.000218 |
| GO:BP | signal transduction in response to DNA damage | GO:0042770 | 0.000218 |
| GO:BP | anatomical structure formation involved in morphogenesis | GO:0048646 | 0.0002342 |
| GO:BP | regulation of establishment of protein localization | GO:0070201 | 0.0002484 |
| GO:BP | myeloid leukocyte differentiation | GO:0002573 | 0.0002536 |
| GO:BP | negative regulation of MAPK cascade | GO:0043409 | 0.0002567 |
| GO:BP | establishment of protein localization to organelle | GO:0072594 | 0.0002721 |
| GO:BP | small molecule biosynthetic process | GO:0044283 | 0.0002839 |
| GO:BP | leukocyte differentiation | GO:0002521 | 0.0002844 |
| GO:BP | positive regulation of defense response | GO:0031349 | 0.0003004 |
| GO:BP | regulation of immune effector process | GO:0002697 | 0.0003031 |
| GO:BP | hematopoietic progenitor cell differentiation | GO:0002244 | 0.0003048 |
| GO:BP | negative regulation of multicellular organismal process | GO:0051241 | 0.0003149 |
| GO:BP | positive regulation of cellular catabolic process | GO:0031331 | 0.000317 |
| GO:BP | regulation of autophagy | GO:0010506 | 0.0003193 |
| GO:BP | blood vessel development | GO:0001568 | 0.0003357 |
| GO:BP | positive regulation of signal transduction | GO:0009967 | 0.000353 |
| GO:BP | positive regulation of intracellular signal transduction | GO:1902533 | 0.0003649 |
| GO:BP | positive regulation of transferase activity | GO:0051347 | 0.0003844 |
| GO:BP | response to lipopolysaccharide | GO:0032496 | 0.0003955 |
| GO:BP | growth | GO:0040007 | 0.0004033 |

| | | | |
|---|---|---|---|
| GO:BP | small GTPase mediated signal transduction | GO:0007264 | 0.0004615 |
| GO:BP | glycerophospholipid metabolic process | GO:0006650 | 0.0004622 |
| GO:BP | protein localization to nucleus | GO:0034504 | 0.0004693 |
| GO:BP | regulation of protein transport | GO:0051223 | 0.0004693 |
| GO:BP | cell-substrate adhesion | GO:0031589 | 0.0004693 |
| GO:BP | regulation of anatomical structure morphogenesis | GO:0022603 | 0.0004714 |
| GO:BP | positive regulation of cellular component biogenesis | GO:0044089 | 0.0004782 |
| GO:BP | angiogenesis | GO:0001525 | 0.0004782 |
| GO:BP | positive regulation of kinase activity | GO:0033674 | 0.000491 |
| GO:BP | positive regulation of transport | GO:0051050 | 0.0005144 |
| GO:BP | cell cycle process | GO:0022402 | 0.0005215 |
| GO:BP | cellular response to lipid | GO:0071396 | 0.0005611 |
| GO:BP | import into cell | GO:0098657 | 0.0005783 |
| GO:BP | response to endogenous stimulus | GO:0009719 | 0.0005879 |
| GO:BP | regulation of ERK1 and ERK2 cascade | GO:0070372 | 0.0005886 |
| GO:BP | response to bacterium | GO:0009617 | 0.0006016 |
| GO:BP | antigen receptor-mediated signaling pathway | GO:0050851 | 0.0006018 |
| GO:BP | endothelial cell migration | GO:0043542 | 0.0006117 |
| GO:BP | localization within membrane | GO:0051668 | 0.0006398 |
| GO:BP | positive regulation of cell population proliferation | GO:0008284 | 0.0006723 |
| GO:BP | positive regulation of cytokine production | GO:0001819 | 0.0007014 |
| GO:BP | blood vessel morphogenesis | GO:0048514 | 0.0007089 |
| GO:BP | regulation of production of molecular mediator of immune response | GO:0002700 | 0.0007144 |
| GO:BP | cellular response to endogenous stimulus | GO:0071495 | 0.0007847 |
| GO:BP | regulation of protein binding | GO:0043393 | 0.000813 |
| GO:BP | positive regulation of cell activation | GO:0050867 | 0.0008511 |
| GO:BP | negative regulation of cell motility | GO:2000146 | 0.0008511 |
| GO:BP | negative regulation of cellular component organization | GO:0051129 | 0.0008511 |
| GO:BP | energy derivation by oxidation of organic compounds | GO:0015980 | 0.0008762 |
| GO:BP | tube development | GO:0035295 | 0.0008762 |
| GO:BP | regulation of response to biotic stimulus | GO:0002831 | 0.0009324 |
| GO:BP | muscle cell development | GO:0055001 | 0.0009346 |
| GO:BP | regulation of binding | GO:0051098 | 0.0009669 |
| GO:BP | transmembrane receptor protein tyrosine kinase signaling pathway | GO:0007169 | 0.0009974 |
| GO:BP | positive regulation of phosphorylation | GO:0042327 | 0.0010027 |
| GO:BP | regulation of cell cycle process | GO:0010564 | 0.0010027 |
| GO:BP | myoblast differentiation | GO:0045445 | 0.0010583 |
| GO:BP | Ras protein signal transduction | GO:0007265 | 0.0011299 |
| GO:BP | negative regulation of locomotion | GO:0040013 | 0.0011329 |
| GO:BP | cellular response to molecule of bacterial origin | GO:0071219 | 0.0011678 |
| GO:BP | regulation of ubiquitin-dependent protein catabolic process | GO:2000058 | 0.0011771 |
| GO:BP | inflammatory response | GO:0006954 | 0.0012097 |
| GO:BP | DNA repair | GO:0006281 | 0.0012526 |
| GO:BP | cellular response to oxidative stress | GO:0034599 | 0.0012665 |
| GO:BP | organophosphate metabolic process | GO:0019637 | 0.0012665 |
| GO:BP | respiratory electron transport chain | GO:0022904 | 0.0012714 |
| GO:BP | regulation of protein catabolic process | GO:0042176 | 0.0012714 |
| GO:BP | ERK1 and ERK2 cascade | GO:0070371 | 0.0012724 |
| GO:BP | positive regulation of organelle organization | GO:0010638 | 0.001319 |
| GO:BP | ATP synthesis coupled electron transport | GO:0042773 | 0.001319 |
| GO:BP | negative regulation of programmed cell death | GO:0043069 | 0.0013929 |
| GO:BP | leukocyte mediated immunity | GO:0002443 | 0.0014069 |
| GO:BP | regulation of endothelial cell migration | GO:0010594 | 0.0014141 |
| GO:BP | fatty acid metabolic process | GO:0006631 | 0.0014219 |
| GO:BP | positive regulation of protein kinase activity | GO:0045860 | 0.0014262 |
| GO:BP | regulation of intracellular protein transport | GO:0033157 | 0.0014346 |

| | | | |
|---|---|---|---|
| GO:BP | positive regulation of cell-cell adhesion | GO:0022409 | 0.0014366 |
| GO:BP | striated muscle cell differentiation | GO:0051146 | 0.001479 |
| GO:BP | regulation of endopeptidase activity | GO:0052548 | 0.001479 |
| GO:BP | positive regulation of nucleobase-containing compound metabolic process | GO:0045935 | 0.0015721 |
| GO:BP | organophosphate ester transport | GO:0015748 | 0.0015759 |
| GO:BP | regulation of proteolysis involved in protein catabolic process | GO:1903050 | 0.0015951 |
| GO:BP | cellular response to lipopolysaccharide | GO:0071222 | 0.0016132 |
| GO:BP | negative regulation of developmental process | GO:0051093 | 0.0016265 |
| GO:BP | positive regulation of programmed cell death | GO:0043068 | 0.0016293 |
| GO:BP | regulation of cytoskeleton organization | GO:0051493 | 0.0018038 |
| GO:BP | macroautophagy | GO:0016236 | 0.0018038 |
| GO:BP | positive regulation of leukocyte activation | GO:0002696 | 0.0018054 |
| GO:BP | negative regulation of protein serine/threonine kinase activity | GO:0071901 | 0.0018054 |
| GO:BP | lymphocyte mediated immunity | GO:0002449 | 0.0018054 |
| GO:BP | response to endoplasmic reticulum stress | GO:0034976 | 0.0018054 |
| GO:BP | positive regulation of apoptotic process | GO:0043065 | 0.0018054 |
| GO:BP | glycosylation | GO:0070085 | 0.0018054 |
| GO:BP | leukocyte migration | GO:0050900 | 0.0018082 |
| GO:BP | glycoprotein biosynthetic process | GO:0009101 | 0.0018146 |
| GO:BP | negative regulation of protein-containing complex assembly | GO:0031333 | 0.0018964 |
| GO:BP | receptor metabolic process | GO:0043112 | 0.0019104 |
| GO:BP | mitochondrial ATP synthesis coupled electron transport | GO:0042775 | 0.0019104 |
| GO:BP | actin filament bundle organization | GO:0061572 | 0.0019775 |
| GO:BP | negative regulation of cell migration | GO:0030336 | 0.0020456 |
| GO:BP | carbohydrate derivative biosynthetic process | GO:1901137 | 0.0020667 |
| GO:BP | positive regulation of ERK1 and ERK2 cascade | GO:0070374 | 0.0021531 |
| GO:BP | tube morphogenesis | GO:0035239 | 0.0021868 |
| GO:BP | membrane protein proteolysis | GO:0033619 | 0.0022425 |
| GO:BP | activation of GTPase activity | GO:0090630 | 0.0022425 |
| GO:BP | response to UV | GO:0009411 | 0.0023059 |
| GO:BP | positive regulation of supramolecular fiber organization | GO:1902905 | 0.0023059 |
| GO:BP | regulation of protein dephosphorylation | GO:0035304 | 0.0023274 |
| GO:BP | ribosome biogenesis | GO:0042254 | 0.0023337 |
| GO:BP | positive regulation of multicellular organismal process | GO:0051240 | 0.0023574 |
| GO:BP | response to stimulus | GO:0050896 | 0.0023574 |
| GO:BP | positive regulation of protein phosphorylation | GO:0001934 | 0.002376 |
| GO:BP | regulation of cellular component size | GO:0032535 | 0.0023992 |
| GO:BP | methylation | GO:0032259 | 0.0024451 |
| GO:BP | regulation of growth | GO:0040008 | 0.0024451 |
| GO:BP | regulation of protein polymerization | GO:0032271 | 0.0024497 |
| GO:BP | adaptive immune response based on somatic recombination of immune receptors built from immunoglobulin superfamily domains | GO:0002460 | 0.0024614 |
| GO:BP | positive regulation of MAP kinase activity | GO:0043406 | 0.0024733 |
| GO:BP | negative regulation of apoptotic process | GO:0043066 | 0.0024942 |
| GO:BP | actin filament bundle assembly | GO:0051017 | 0.0026543 |
| GO:BP | negative regulation of phosphorus metabolic process | GO:0010563 | 0.0026802 |
| GO:BP | negative regulation of phosphate metabolic process | GO:0045936 | 0.0026802 |
| GO:BP | intrinsic apoptotic signaling pathway | GO:0097193 | 0.0026918 |
| GO:BP | regulation of angiogenesis | GO:0045765 | 0.0027361 |
| GO:BP | regulation of innate immune response | GO:0045088 | 0.0027361 |
| GO:BP | positive regulation of intracellular transport | GO:0032388 | 0.0027427 |
| GO:BP | negative regulation of nucleobase-containing compound metabolic process | GO:0045934 | 0.0027427 |
| GO:BP | positive regulation of endopeptidase activity | GO:0010950 | 0.0028542 |
| GO:BP | anatomical structure morphogenesis | GO:0009653 | 0.0028542 |

| | | | |
|---|---|---|---|
| GO:BP | positive regulation of innate immune response | GO:0045089 | 0.0028772 |
| GO:BP | positive regulation of developmental process | GO:0051094 | 0.002911 |
| GO:BP | T cell receptor signaling pathway | GO:0050852 | 0.002911 |
| GO:BP | phospholipid transport | GO:0015914 | 0.002911 |
| GO:BP | positive regulation of GTPase activity | GO:0043547 | 0.0029775 |
| GO:BP | aminoglycan metabolic process | GO:0006022 | 0.0029948 |
| GO:BP | protein glycosylation | GO:0006486 | 0.0030567 |
| GO:BP | macromolecule glycosylation | GO:0043413 | 0.0030567 |
| GO:BP | monosaccharide metabolic process | GO:0005996 | 0.0030567 |
| GO:BP | positive regulation of protein catabolic process | GO:0045732 | 0.0030583 |
| GO:BP | response to oxygen levels | GO:0070482 | 0.0030583 |
| GO:BP | regulation of cysteine-type endopeptidase activity | GO:2000116 | 0.0031369 |
| GO:BP | cell-matrix adhesion | GO:0007160 | 0.0031369 |
| GO:BP | positive regulation of immune effector process | GO:0002699 | 0.0031766 |
| GO:BP | RNA modification | GO:0009451 | 0.0031766 |
| GO:BP | glycerolipid metabolic process | GO:0046486 | 0.0032203 |
| GO:BP | mononuclear cell differentiation | GO:1903131 | 0.0032203 |
| GO:BP | signal transduction by p53 class mediator | GO:0072331 | 0.0032664 |
| GO:BP | negative regulation of defense response | GO:0031348 | 0.0033451 |
| GO:BP | regulation of hemopoiesis | GO:1903706 | 0.0034128 |
| GO:BP | macromolecule methylation | GO:0043414 | 0.0034321 |
| GO:BP | positive regulation of response to biotic stimulus | GO:0002833 | 0.0034443 |
| GO:BP | positive regulation of peptidase activity | GO:0010952 | 0.0034715 |
| GO:BP | regulation of cytokine production involved in immune response | GO:0002718 | 0.0035965 |
| GO:BP | cytokine production involved in immune response | GO:0002367 | 0.0035965 |
| GO:BP | positive regulation of establishment of protein localization | GO:1904951 | 0.003742 |
| GO:BP | positive regulation of protein transport | GO:0051222 | 0.0038696 |
| GO:BP | regulation of lymphocyte activation | GO:0051249 | 0.0039728 |
| GO:BP | cytoplasmic translation | GO:0002181 | 0.0040873 |
| GO:BP | mitotic cell cycle process | GO:1903047 | 0.0041397 |
| GO:BP | endosomal transport | GO:0016197 | 0.0041397 |
| GO:BP | regulation of vasculature development | GO:1901342 | 0.0041397 |
| GO:BP | regulation of dephosphorylation | GO:0035303 | 0.0041875 |
| GO:BP | wound healing | GO:0042060 | 0.0043561 |
| GO:BP | endomembrane system organization | GO:0010256 | 0.0044506 |
| GO:BP | response to wounding | GO:0009611 | 0.004624 |
| GO:BP | cellular homeostasis | GO:0019725 | 0.0048635 |
| GO:BP | retrograde transport, endosome to Golgi | GO:0042147 | 0.0048828 |
| GO:BP | positive regulation of epithelial cell migration | GO:0010634 | 0.0048942 |
| GO:BP | Rho protein signal transduction | GO:0007266 | 0.0049829 |
| GO:BP | maintenance of location | GO:0051235 | 0.0050512 |
| GO:BP | mitochondrial membrane organization | GO:0007006 | 0.0052537 |
| GO:BP | phospholipid metabolic process | GO:0006644 | 0.0053016 |
| GO:BP | positive regulation of signaling | GO:0023056 | 0.0054618 |
| GO:BP | regulation of actin polymerization or depolymerization | GO:0008064 | 0.0055061 |
| GO:BP | regulation of actin filament length | GO:0030832 | 0.0055061 |
| GO:BP | extracellular structure organization | GO:0043062 | 0.0056324 |
| GO:BP | actin polymerization or depolymerization | GO:0008154 | 0.0056324 |
| GO:BP | external encapsulating structure organization | GO:0045229 | 0.0056324 |
| GO:BP | translational initiation | GO:0006413 | 0.0056324 |
| GO:BP | regulation of chromosome organization | GO:0033044 | 0.0056742 |
| GO:BP | regulation of protein stability | GO:0031647 | 0.0057371 |
| GO:BP | mitotic cell cycle | GO:0000278 | 0.0059008 |
| GO:BP | cell growth | GO:0016049 | 0.0059008 |
| GO:BP | non-membrane-bounded organelle assembly | GO:0140694 | 0.0059008 |
| GO:BP | aerobic electron transport chain | GO:0019646 | 0.0059043 |

| | | | |
|---|---|---|---|
| GO:BP | protein polymerization | GO:0051258 | 0.0059521 |
| GO:BP | transcription by RNA polymerase III | GO:0006383 | 0.0061684 |
| GO:BP | positive regulation of dephosphorylation | GO:0035306 | 0.0061684 |
| GO:BP | apoptotic mitochondrial changes | GO:0008637 | 0.0062183 |
| GO:BP | extracellular matrix organization | GO:0030198 | 0.006252 |
| GO:BP | response to radiation | GO:0009314 | 0.0063581 |
| GO:BP | positive regulation of lymphocyte activation | GO:0051251 | 0.006366 |
| GO:BP | positive regulation of MAPK cascade | GO:0043410 | 0.006442 |
| GO:BP | negative regulation of immune system process | GO:0002683 | 0.0065108 |
| GO:BP | glycosaminoglycan metabolic process | GO:0030203 | 0.0065108 |
| GO:BP | chromosome organization | GO:0051276 | 0.0065108 |
| GO:BP | organelle localization | GO:0051640 | 0.0067539 |
| GO:BP | mucopolysaccharide metabolic process | GO:1903510 | 0.0067726 |
| GO:BP | negative regulation of catalytic activity | GO:0043086 | 0.0069053 |
| GO:BP | positive regulation of cell communication | GO:0010647 | 0.0070131 |
| GO:BP | multicellular organismal-level homeostasis | GO:0048871 | 0.0070204 |
| GO:BP | regulation of cell growth | GO:0001558 | 0.0070582 |
| GO:BP | skeletal muscle tissue development | GO:0007519 | 0.0071535 |
| GO:BP | protein autophosphorylation | GO:0046777 | 0.0071535 |
| GO:BP | regulation of proteasomal ubiquitin-dependent protein catabolic | GO:0032434 | 0.007229 |
| GO:BP | negative regulation of RNA biosynthetic process | GO:1902679 | 0.007229 |
| GO:BP | protein localization to membrane | GO:0072657 | 0.00725 |
| GO:BP | regulation of Ras protein signal transduction | GO:0046578 | 0.00725 |
| GO:BP | myeloid leukocyte migration | GO:0097529 | 0.00725 |
| GO:BP | negative regulation of amide metabolic process | GO:0034249 | 0.007518 |
| GO:BP | mitochondrial transport | GO:0006839 | 0.0075493 |
| GO:BP | protein processing | GO:0016485 | 0.0075871 |
| GO:BP | cell cycle phase transition | GO:0044770 | 0.0076886 |
| GO:BP | regulation of cellular metabolic process | GO:0031323 | 0.0077303 |
| GO:BP | cell chemotaxis | GO:0060326 | 0.0077303 |
| GO:BP | taxis | GO:0042330 | 0.0078981 |
| GO:BP | positive regulation of production of molecular mediator of immune response | GO:0002702 | 0.0078981 |
| GO:BP | regulation of cell-matrix adhesion | GO:0001952 | 0.0078981 |
| GO:BP | regulation of mitochondrion organization | GO:0010821 | 0.0080138 |
| GO:BP | positive regulation of autophagy | GO:0010508 | 0.0084001 |
| GO:BP | regulation of macromolecule metabolic process | GO:0060255 | 0.0084684 |
| GO:BP | regulation of proteasomal protein catabolic process | GO:0061136 | 0.0084914 |
| GO:BP | platelet-derived growth factor receptor signaling pathway | GO:0048008 | 0.0084914 |
| GO:BP | negative regulation of DNA-templated transcription | GO:0045892 | 0.0085924 |
| GO:BP | regulation of DNA metabolic process | GO:0051052 | 0.0087044 |
| GO:BP | B cell mediated immunity | GO:0019724 | 0.0087162 |
| GO:BP | phosphatidylinositol metabolic process | GO:0046488 | 0.0088969 |
| GO:BP | regulation of small GTPase mediated signal transduction | GO:0051056 | 0.0091103 |
| GO:BP | regulation of inflammatory response | GO:0050727 | 0.0091815 |
| GO:BP | sterol metabolic process | GO:0016125 | 0.0092948 |
| GO:BP | tRNA processing | GO:0008033 | 0.0095779 |
| GO:BP | carboxylic acid catabolic process | GO:0046395 | 0.0095779 |
| GO:BP | regulation of myeloid cell differentiation | GO:0045637 | 0.0095779 |
| GO:BP | chemotaxis | GO:0006935 | 0.0095779 |
| GO:BP | regulation of leukocyte mediated immunity | GO:0002703 | 0.0095779 |
| GO:BP | organic acid catabolic process | GO:0016054 | 0.0095779 |
| GO:BP | import into nucleus | GO:0051170 | 0.0095779 |
| GO:BP | cellular response to chemical stress | GO:0062197 | 0.009653 |
| GO:BP | negative regulation of catabolic process | GO:0009895 | 0.0096781 |
| GO:BP | skeletal muscle organ development | GO:0060538 | 0.0100578 |

| | | | |
|---|---|---|---|
| GO:BP | coagulation | GO:0050817 | 0.0100578 |
| GO:BP | mitochondrial respiratory chain complex I assembly | GO:0032981 | 0.0101597 |
| GO:BP | cellular response to oxygen levels | GO:0071453 | 0.0101597 |
| GO:BP | NADH dehydrogenase complex assembly | GO:0010257 | 0.0101597 |
| GO:BP | regulation of cell-cell adhesion | GO:0022407 | 0.0102096 |
| GO:BP | immunoglobulin mediated immune response | GO:0016064 | 0.0103014 |
| GO:BP | leukocyte chemotaxis | GO:0030595 | 0.0103896 |
| GO:BP | positive regulation of leukocyte cell-cell adhesion | GO:1903039 | 0.0103896 |
| GO:BP | nucleobase-containing compound transport | GO:0015931 | 0.0103896 |
| GO:BP | regulation of actin filament polymerization | GO:0030833 | 0.0106322 |
| GO:BP | positive regulation of intracellular protein transport | GO:0090316 | 0.0106322 |
| GO:BP | response to growth factor | GO:0070848 | 0.01066 |
| GO:BP | positive regulation of RNA metabolic process | GO:0051254 | 0.01066 |
| GO:BP | actin filament polymerization | GO:0030041 | 0.0108625 |
| GO:BP | myeloid leukocyte cytokine production | GO:0061082 | 0.011109 |
| GO:BP | membrane invagination | GO:0010324 | 0.011109 |
| GO:BP | activation of innate immune response | GO:0002218 | 0.0111935 |
| GO:BP | lipid modification | GO:0030258 | 0.0113057 |
| GO:BP | cellular response to growth factor stimulus | GO:0071363 | 0.0115053 |
| GO:BP | cellular response to external stimulus | GO:0071496 | 0.011576 |
| GO:BP | regulation of GTPase activity | GO:0043087 | 0.0116956 |
| GO:BP | negative regulation of RNA metabolic process | GO:0051253 | 0.0116956 |
| GO:BP | endothelium development | GO:0003158 | 0.0116956 |
| GO:BP | protein localization to plasma membrane | GO:0072659 | 0.0116956 |
| GO:BP | positive regulation of T cell proliferation | GO:0042102 | 0.0121473 |
| GO:BP | positive regulation of cytokine production involved in immune | GO:0002720 | 0.0121473 |
| GO:BP | positive regulation of cysteine-type endopeptidase activity | GO:2001056 | 0.0122487 |
| GO:BP | TOR signaling | GO:0031929 | 0.0124377 |
| GO:BP | cell cycle checkpoint signaling | GO:0000075 | 0.0128508 |
| GO:BP | regulation of peptidase activity | GO:0052547 | 0.01286 |
| GO:BP | negative regulation of protein phosphorylation | GO:0001933 | 0.0129959 |
| GO:BP | phospholipid biosynthetic process | GO:0008654 | 0.013013 |
| GO:BP | regulation of cell development | GO:0060284 | 0.0131008 |
| GO:BP | fat cell differentiation | GO:0045444 | 0.013387 |
| GO:BP | negative regulation of transferase activity | GO:0051348 | 0.013387 |
| GO:BP | regulation of cell-substrate adhesion | GO:0010810 | 0.0135317 |
| GO:BP | innate immune response activating cell surface receptor signaling pathway | GO:0002220 | 0.0135349 |
| GO:BP | regulation of phosphoprotein phosphatase activity | GO:0043666 | 0.0135349 |
| GO:BP | response to organonitrogen compound | GO:0010243 | 0.0135349 |
| GO:BP | membrane protein ectodomain proteolysis | GO:0006509 | 0.0135349 |
| GO:BP | phosphatidylinositol 3-kinase/protein kinase B signal transduction | GO:0043491 | 0.0136064 |
| GO:BP | response to decreased oxygen levels | GO:0036293 | 0.0136064 |
| GO:BP | response to organic cyclic compound | GO:0014070 | 0.0136842 |
| GO:BP | response to reactive oxygen species | GO:0000302 | 0.0137612 |
| GO:BP | myeloid leukocyte activation | GO:0002274 | 0.0137612 |
| GO:BP | protein import into nucleus | GO:0006606 | 0.0141277 |
| GO:BP | homeostasis of number of cells | GO:0048872 | 0.0142167 |
| GO:BP | blood coagulation | GO:0007596 | 0.0143945 |
| GO:BP | regulation of endocytosis | GO:0030100 | 0.0144203 |
| GO:BP | protein transmembrane transport | GO:0071806 | 0.0144891 |
| GO:BP | regulation of anatomical structure size | GO:0090066 | 0.0145386 |
| GO:BP | B cell activation involved in immune response | GO:0002312 | 0.0149741 |
| GO:BP | rRNA processing | GO:0006364 | 0.0150917 |
| GO:BP | cytoskeleton-dependent intracellular transport | GO:0030705 | 0.0150917 |
| GO:BP | negative regulation of phosphorylation | GO:0042326 | 0.0155454 |

| | | | |
|---|---|---|---|
| GO:BP | contractile actin filament bundle assembly | GO:0030038 | 0.0156057 |
| GO:BP | stress fiber assembly | GO:0043149 | 0.0156057 |
| GO:BP | positive regulation of endocytosis | GO:0045807 | 0.0156057 |
| GO:BP | positive regulation of amide metabolic process | GO:0034250 | 0.0156468 |
| GO:BP | positive regulation of T cell activation | GO:0050870 | 0.0156468 |
| GO:BP | regulation of cysteine-type endopeptidase activity involved in apoptotic process | GO:0043281 | 0.0162961 |
| GO:BP | regulation of cell cycle phase transition | GO:1901987 | 0.0164468 |
| GO:BP | endothelial cell differentiation | GO:0045446 | 0.0169839 |
| GO:BP | cholesterol metabolic process | GO:0008203 | 0.0169839 |
| GO:BP | organelle assembly | GO:0070925 | 0.0176201 |
| GO:BP | T cell activation | GO:0042110 | 0.0176321 |
| GO:BP | RNA methylation | GO:0001510 | 0.0176554 |
| GO:BP | DNA-templated transcription elongation | GO:0006354 | 0.0176554 |
| GO:BP | negative regulation of cell cycle | GO:0045786 | 0.0178257 |
| GO:BP | canonical NF-kappaB signal transduction | GO:0007249 | 0.0181072 |
| GO:BP | DNA damage checkpoint signaling | GO:0000077 | 0.0181881 |
| GO:BP | aromatic compound catabolic process | GO:0019439 | 0.0182619 |
| GO:BP | chemical homeostasis | GO:0048878 | 0.0182702 |
| GO:BP | cytokine-mediated signaling pathway | GO:0019221 | 0.018625 |
| GO:BP | response to salt | GO:1902074 | 0.0188038 |
| GO:BP | hexose metabolic process | GO:0019318 | 0.0190478 |
| GO:BP | positive regulation of cell differentiation | GO:0045597 | 0.0196321 |
| GO:BP | response to hypoxia | GO:0001666 | 0.019634 |
| GO:BP | regulation of protein ubiquitination | GO:0031396 | 0.0196755 |
| GO:BP | regulation of cell morphogenesis | GO:0022604 | 0.0196755 |
| GO:BP | cell surface pattern recognition receptor signaling pathway | GO:0002752 | 0.0204019 |
| GO:BP | cell surface toll-like receptor signaling pathway | GO:0140895 | 0.0204019 |
| GO:BP | rRNA metabolic process | GO:0016072 | 0.0206008 |
| GO:BP | sterol homeostasis | GO:0055092 | 0.020746 |
| GO:BP | in utero embryonic development | GO:0001701 | 0.020746 |
| GO:BP | cholesterol homeostasis | GO:0042632 | 0.020746 |
| GO:BP | regulation of leukocyte differentiation | GO:1902105 | 0.0207706 |
| GO:BP | cellular response to radiation | GO:0071478 | 0.0207706 |
| GO:BP | response to nitrogen compound | GO:1901698 | 0.0207706 |
| GO:BP | positive regulation of protein-containing complex assembly | GO:0031334 | 0.0207706 |
| GO:BP | organic hydroxy compound metabolic process | GO:1901615 | 0.0207706 |
| GO:BP | regulation of vesicle-mediated transport | GO:0060627 | 0.0214585 |
| GO:BP | response to ionizing radiation | GO:0010212 | 0.0214585 |
| GO:BP | regulation of viral process | GO:0050792 | 0.0214585 |
| GO:BP | protein-containing complex assembly | GO:0065003 | 0.0219012 |
| GO:BP | hemostasis | GO:0007599 | 0.0219517 |
| GO:BP | positive regulation of DNA-templated transcription | GO:0045893 | 0.0219783 |
| GO:BP | cellular nitrogen compound catabolic process | GO:0044270 | 0.0219945 |
| GO:BP | positive regulation of RNA biosynthetic process | GO:1902680 | 0.0220076 |
| GO:BP | response to inorganic substance | GO:0010035 | 0.0227859 |
| GO:BP | organic cyclic compound catabolic process | GO:1901361 | 0.0229164 |
| GO:BP | tRNA modification | GO:0006400 | 0.0229491 |
| GO:BP | leukocyte cell-cell adhesion | GO:0007159 | 0.0232859 |
| GO:BP | mesenchymal cell differentiation | GO:0048762 | 0.0232859 |
| GO:BP | Golgi vesicle transport | GO:0048193 | 0.0233483 |
| GO:BP | innate immune response-activating signaling pathway | GO:0002758 | 0.0237622 |
| GO:BP | activation of cysteine-type endopeptidase activity involved in apoptotic process | GO:0006919 | 0.0239033 |
| GO:BP | regulation of Rho protein signal transduction | GO:0035023 | 0.0239033 |
| GO:BP | response to type II interferon | GO:0034341 | 0.0241835 |

| | | | |
|---|---|---|---|
| GO:BP | blood vessel endothelial cell migration | GO:0043534 | 0.0245346 |
| GO:BP | positive regulation of phagocytosis | GO:0050766 | 0.0247357 |
| GO:BP | multicellular organismal movement | GO:0050879 | 0.0247357 |
| GO:BP | regulation of nucleobase-containing compound metabolic process | GO:0019219 | 0.0249094 |
| GO:BP | positive regulation of small GTPase mediated signal transduction | GO:0051057 | 0.0250739 |
| GO:BP | maintenance of location in cell | GO:0051651 | 0.0267406 |
| GO:BP | pattern recognition receptor signaling pathway | GO:0002221 | 0.0267406 |
| GO:BP | regulation of post-translational protein modification | GO:1901873 | 0.0274809 |
| GO:BP | positive regulation of cell cycle | GO:0045787 | 0.0278928 |
| GO:BP | cytosolic transport | GO:0016482 | 0.028 |
| GO:BP | sphingolipid biosynthetic process | GO:0030148 | 0.0282668 |
| GO:BP | regulation of macroautophagy | GO:0016241 | 0.0282779 |
| GO:BP | regulation of lipid biosynthetic process | GO:0046890 | 0.0282779 |
| GO:BP | regulation of leukocyte migration | GO:0002685 | 0.0282779 |
| GO:BP | DNA integrity checkpoint signaling | GO:0031570 | 0.0285345 |
| GO:BP | organic acid biosynthetic process | GO:0016053 | 0.0287324 |
| GO:BP | regulation of T cell activation | GO:0050863 | 0.0287557 |
| GO:BP | negative regulation of cell differentiation | GO:0045596 | 0.0289146 |
| GO:BP | glycerophospholipid biosynthetic process | GO:0046474 | 0.0289151 |
| GO:BP | organophosphate biosynthetic process | GO:0090407 | 0.0295922 |
| GO:BP | protein localization to cell periphery | GO:1990778 | 0.0297011 |
| GO:BP | regulation of myoblast differentiation | GO:0045661 | 0.0303423 |
| GO:BP | musculoskeletal movement | GO:0050881 | 0.0304273 |
| GO:BP | water-soluble vitamin metabolic process | GO:0006767 | 0.0304273 |
| GO:BP | positive regulation of reactive oxygen species metabolic process | GO:2000379 | 0.0304273 |
| GO:BP | nucleobase-containing small molecule metabolic process | GO:0055086 | 0.0306077 |
| GO:BP | system development | GO:0048731 | 0.030875 |
| GO:BP | negative regulation of gene expression, epigenetic | GO:0045814 | 0.030875 |
| GO:BP | regulation of viral life cycle | GO:1903900 | 0.030875 |
| GO:BP | regulation of protein localization to cell periphery | GO:1904375 | 0.030875 |
| GO:BP | alpha-beta T cell activation | GO:0046631 | 0.030875 |
| GO:BP | lipid homeostasis | GO:0055088 | 0.030875 |
| GO:BP | negative regulation of cytokine production | GO:0001818 | 0.030875 |
| GO:BP | cell activation involved in immune response | GO:0002263 | 0.030875 |
| GO:BP | negative regulation of translation | GO:0017148 | 0.0309518 |
| GO:BP | stress-activated protein kinase signaling cascade | GO:0031098 | 0.0310866 |
| GO:BP | T cell proliferation | GO:0042098 | 0.0315788 |
| GO:BP | negative regulation of inflammatory response | GO:0050728 | 0.0318931 |
| GO:BP | secondary alcohol metabolic process | GO:1902652 | 0.0319577 |
| GO:BP | negative regulation of protein kinase activity | GO:0006469 | 0.0319577 |
| GO:BP | negative regulation of post-translational protein modification | GO:1901874 | 0.0319577 |
| GO:BP | regulation of actin filament bundle assembly | GO:0032231 | 0.0319577 |
| GO:BP | mitochondrial respiratory chain complex assembly | GO:0033108 | 0.0319577 |
| GO:BP | regulation of cell shape | GO:0008360 | 0.0321971 |
| GO:BP | lymphocyte differentiation | GO:0030098 | 0.0326479 |
| GO:BP | heterochromatin organization | GO:0070828 | 0.0329025 |
| GO:BP | sphingolipid metabolic process | GO:0006665 | 0.0331025 |
| GO:BP | DNA damage response, signal transduction by p53 class mediator | GO:0030330 | 0.0331025 |
| GO:BP | protein N-linked glycosylation | GO:0006487 | 0.0331025 |
| GO:BP | cellular response to nitrogen compound | GO:1901699 | 0.033165 |
| GO:BP | extrinsic apoptotic signaling pathway | GO:0097191 | 0.0333282 |
| GO:BP | tRNA metabolic process | GO:0006399 | 0.0333282 |
| GO:BP | negative regulation of kinase activity | GO:0033673 | 0.0333282 |
| GO:BP | regulation of mitotic cell cycle | GO:0007346 | 0.0333317 |
| GO:BP | chromosome segregation | GO:0007059 | 0.033675 |
| GO:BP | heterocycle catabolic process | GO:0046700 | 0.0338867 |

| | | | |
|---|---|---|---|
| GO:BP | regulation of protein modification by small protein conjugation or removal | GO:1903320 | 0.0342947 |
| GO:BP | T cell differentiation | GO:0030217 | 0.0342947 |
| GO:BP | protein targeting | GO:0006605 | 0.0343047 |
| GO:BP | positive regulation of protein binding | GO:0032092 | 0.0354923 |
| GO:BP | receptor recycling | GO:0001881 | 0.0354923 |
| GO:BP | intracellular protein transmembrane transport | GO:0065002 | 0.0354923 |
| GO:BP | positive regulation of proteolysis involved in protein catabolic | GO:1903052 | 0.0360095 |
| GO:BP | xenobiotic metabolic process | GO:0006805 | 0.036389 |
| GO:BP | plasma membrane invagination | GO:0099024 | 0.036389 |
| GO:BP | toll-like receptor 4 signaling pathway | GO:0034142 | 0.036389 |
| GO:BP | positive regulation of protein dephosphorylation | GO:0035307 | 0.036389 |
| GO:BP | regulation of lymphocyte mediated immunity | GO:0002706 | 0.0364895 |
| GO:BP | positive regulation of ubiquitin-dependent protein catabolic process | GO:2000060 | 0.0364895 |
| GO:BP | positive regulation of cysteine-type endopeptidase activity involved in apoptotic process | GO:0043280 | 0.0365184 |
| GO:BP | nucleobase-containing compound catabolic process | GO:0034655 | 0.0371623 |
| GO:BP | carbohydrate catabolic process | GO:0016052 | 0.0372169 |
| GO:BP | vesicle-mediated transport to the plasma membrane | GO:0098876 | 0.0372169 |
| GO:BP | regulation of leukocyte cell-cell adhesion | GO:1903037 | 0.0372719 |
| GO:BP | carboxylic acid biosynthetic process | GO:0046394 | 0.0372719 |
| GO:BP | leukocyte activation involved in immune response | GO:0002366 | 0.0372719 |
| GO:BP | response to transforming growth factor beta | GO:0071559 | 0.0375079 |
| GO:BP | response to heat | GO:0009408 | 0.0379019 |
| GO:BP | positive regulation of binding | GO:0051099 | 0.0382011 |
| GO:BP | cell-cell adhesion | GO:0098609 | 0.0384128 |
| GO:BP | regulation of phagocytosis | GO:0050764 | 0.0385468 |
| GO:BP | negative regulation of autophagy | GO:0010507 | 0.0385468 |
| GO:BP | cardiocyte differentiation | GO:0035051 | 0.0390325 |
| GO:BP | epigenetic regulation of gene expression | GO:0040029 | 0.0393272 |
| GO:BP | cellular ketone metabolic process | GO:0042180 | 0.0403469 |
| GO:BP | regulation of DNA-binding transcription factor activity | GO:0051090 | 0.0405447 |
| GO:BP | mitotic nuclear division | GO:0140014 | 0.0406588 |
| GO:BP | positive regulation of NF-kappaB transcription factor activity | GO:0051092 | 0.0406699 |
| GO:BP | T cell differentiation in thymus | GO:0033077 | 0.0407115 |
| GO:BP | stem cell differentiation | GO:0048863 | 0.0412155 |
| GO:BP | epithelium development | GO:0060429 | 0.0412155 |
| GO:BP | receptor-mediated endocytosis | GO:0006898 | 0.0412155 |
| GO:BP | steroid metabolic process | GO:0008202 | 0.0412155 |
| GO:BP | membrane lipid metabolic process | GO:0006643 | 0.0414236 |
| GO:BP | regulation of actomyosin structure organization | GO:0110020 | 0.0416795 |
| GO:BP | transport along microtubule | GO:0010970 | 0.0417543 |
| GO:BP | endothelial cell development | GO:0001885 | 0.0418599 |
| GO:BP | mitochondrial transmembrane transport | GO:1990542 | 0.0418599 |
| GO:BP | cellular response to organic cyclic compound | GO:0071407 | 0.0426185 |
| GO:BP | regulation of stress-activated protein kinase signaling cascade | GO:0070302 | 0.0439196 |
| GO:BP | skeletal muscle fiber development | GO:0048741 | 0.0440378 |
| GO:BP | semaphorin-plexin signaling pathway | GO:0071526 | 0.0440378 |
| GO:BP | mitochondrial electron transport, NADH to ubiquinone | GO:0006120 | 0.0440378 |
| GO:BP | lymphocyte migration | GO:0072676 | 0.044052 |
| GO:BP | nuclear-transcribed mRNA catabolic process | GO:0000956 | 0.044052 |
| GO:BP | mRNA catabolic process | GO:0006402 | 0.0454611 |
| GO:BP | RNA catabolic process | GO:0006401 | 0.0454611 |
| GO:BP | epithelial cell development | GO:0002064 | 0.0456334 |
| GO:BP | cellular response to nutrient levels | GO:0031669 | 0.0456334 |
| GO:BP | amino acid metabolic process | GO:0006520 | 0.0463839 |

| | | | |
|---|---|---|---|
| GO:BP | developmental growth | GO:0048589 | 0.047493 |
| GO:BP | positive regulation of proteasomal protein catabolic process | GO:1901800 | 0.0475984 |
| GO:BP | regulation of nucleocytoplasmic transport | GO:0046822 | 0.0475984 |
| GO:BP | B cell activation | GO:0042113 | 0.0498112 |
| GO:BP | double-strand break repair | GO:0006302 | 0.0499808 |
| GO:CC | cytoplasm | GO:0005737 | 1.42E-142 |
| GO:CC | intracellular anatomical structure | GO:0005622 | 6.42E-67 |
| GO:CC | intracellular membrane-bounded organelle | GO:0043231 | 2.01E-65 |
| GO:CC | membrane-bounded organelle | GO:0043227 | 1.42E-64 |
| GO:CC | cytosol | GO:0005829 | 2.17E-39 |
| GO:CC | intracellular organelle | GO:0043229 | 5.79E-38 |
| GO:CC | organelle | GO:0043226 | 9.50E-35 |
| GO:CC | nucleoplasm | GO:0005654 | 9.02E-33 |
| GO:CC | cellular anatomical entity | GO:0110165 | 1.02E-31 |
| GO:CC | endomembrane system | GO:0012505 | 2.28E-31 |
| GO:CC | membrane-enclosed lumen | GO:0031974 | 3.21E-26 |
| GO:CC | organelle lumen | GO:0043233 | 3.21E-26 |
| GO:CC | intracellular organelle lumen | GO:0070013 | 3.21E-26 |
| GO:CC | catalytic complex | GO:1902494 | 3.97E-25 |
| GO:CC | organelle membrane | GO:0031090 | 1.33E-22 |
| GO:CC | nuclear lumen | GO:0031981 | 2.68E-21 |
| GO:CC | mitochondrion | GO:0005739 | 3.84E-19 |
| GO:CC | Golgi apparatus | GO:0005794 | 3.78E-18 |
| GO:CC | endoplasmic reticulum | GO:0005783 | 3.41E-14 |
| GO:CC | intracellular protein-containing complex | GO:0140535 | 4.13E-13 |
| GO:CC | protein-containing complex | GO:0032991 | 5.78E-13 |
| GO:CC | organelle envelope | GO:0031967 | 7.43E-13 |
| GO:CC | envelope | GO:0031975 | 7.43E-13 |
| GO:CC | nucleus | GO:0005634 | 1.28E-12 |
| GO:CC | mitochondrial envelope | GO:0005740 | 2.70E-11 |
| GO:CC | mitochondrial membrane | GO:0031966 | 2.51E-10 |
| GO:CC | organelle subcompartment | GO:0031984 | 2.63E-10 |
| GO:CC | actin cytoskeleton | GO:0015629 | 1.42E-09 |
| GO:CC | mitochondrial protein-containing complex | GO:0098798 | 6.36E-09 |
| GO:CC | transferase complex | GO:1990234 | 3.29E-08 |
| GO:CC | mitochondrial inner membrane | GO:0005743 | 4.45E-08 |
| GO:CC | bounding membrane of organelle | GO:0098588 | 6.46E-08 |
| GO:CC | endoplasmic reticulum subcompartment | GO:0098827 | 8.08E-08 |
| GO:CC | nuclear outer membrane-endoplasmic reticulum membrane network | GO:0042175 | 8.17E-08 |
| GO:CC | endoplasmic reticulum membrane | GO:0005789 | 8.17E-08 |
| GO:CC | vesicle | GO:0031982 | 4.41E-07 |
| GO:CC | organelle inner membrane | GO:0019866 | 9.55E-07 |
| GO:CC | nuclear body | GO:0016604 | 1.49E-06 |
| GO:CC | endosome | GO:0005768 | 2.12E-06 |
| GO:CC | Golgi membrane | GO:0000139 | 2.14E-06 |
| GO:CC | cytoplasmic vesicle | GO:0031410 | 2.14E-06 |
| GO:CC | plasma membrane | GO:0005886 | 2.14E-06 |
| GO:CC | cell periphery | GO:0071944 | 2.48E-06 |
| GO:CC | intracellular vesicle | GO:0097708 | 3.45E-06 |
| GO:CC | inner mitochondrial membrane protein complex | GO:0098800 | 5.87E-06 |
| GO:CC | sarcomere | GO:0030017 | 1.00E-05 |
| GO:CC | ribosomal subunit | GO:0044391 | 1.37E-05 |
| GO:CC | chromosome | GO:0005694 | 1.69E-05 |
| GO:CC | perinuclear region of cytoplasm | GO:0048471 | 2.29E-05 |
| GO:CC | nuclear speck | GO:0016607 | 2.64E-05 |
| GO:CC | contractile fiber | GO:0043292 | 5.83E-05 |

| | | | |
|---|---|---|---|
| GO:CC | membrane protein complex | GO:0098796 | 5.83E-05 |
| GO:CC | ubiquitin ligase complex | GO:0000151 | 7.44E-05 |
| GO:CC | cell surface | GO:0009986 | 8.42E-05 |
| GO:CC | myofibril | GO:0030016 | 0.0001166 |
| GO:CC | spliceosomal complex | GO:0005681 | 0.0001295 |
| GO:CC | vacuole | GO:0005773 | 0.0001419 |
| GO:CC | side of membrane | GO:0098552 | 0.0002207 |
| GO:CC | lysosome | GO:0005764 | 0.0003539 |
| GO:CC | microtubule cytoskeleton | GO:0015630 | 0.0003539 |
| GO:CC | lytic vacuole | GO:0000323 | 0.0003539 |
| GO:CC | mitochondrial matrix | GO:0005759 | 0.0004625 |
| GO:CC | chromatin | GO:0000785 | 0.0005243 |
| GO:CC | I band | GO:0031674 | 0.0005596 |
| GO:CC | endopeptidase complex | GO:1905369 | 0.0005621 |
| GO:CC | cytosolic ribosome | GO:0022626 | 0.0006768 |
| GO:CC | protein-DNA complex | GO:0032993 | 0.0006773 |
| GO:CC | small ribosomal subunit | GO:0015935 | 0.0007625 |
| GO:CC | peptidase complex | GO:1905368 | 0.0010415 |
| GO:CC | nuclear membrane | GO:0031965 | 0.0010415 |
| GO:CC | cytoplasmic ribonucleoprotein granule | GO:0036464 | 0.0010449 |
| GO:CC | ribonucleoprotein granule | GO:0035770 | 0.001124 |
| GO:CC | microtubule organizing center | GO:0005815 | 0.001174 |
| GO:CC | focal adhesion | GO:0005925 | 0.0013426 |
| GO:CC | cell-substrate junction | GO:0030055 | 0.0013696 |
| GO:CC | centrosome | GO:0005813 | 0.0013738 |
| GO:CC | Z disc | GO:0030018 | 0.00252 |
| GO:CC | endosome membrane | GO:0010008 | 0.0025539 |
| GO:CC | nuclear envelope | GO:0005635 | 0.0030185 |
| GO:CC | membrane raft | GO:0045121 | 0.0032503 |
| GO:CC | membrane microdomain | GO:0098857 | 0.0032503 |
| GO:CC | U2-type spliceosomal complex | GO:0005684 | 0.0039857 |
| GO:CC | respiratory chain complex | GO:0098803 | 0.0039857 |
| GO:CC | early endosome | GO:0005769 | 0.0040337 |
| GO:CC | proteasome complex | GO:0000502 | 0.0040337 |
| GO:CC | oxidoreductase complex | GO:1990204 | 0.0040934 |
| GO:CC | peroxisome | GO:0005777 | 0.0056656 |
| GO:CC | microbody | GO:0042579 | 0.0056656 |
| GO:CC | external side of plasma membrane | GO:0009897 | 0.006335 |
| GO:CC | respirasome | GO:0070469 | 0.0078965 |
| GO:CC | Golgi apparatus subcompartment | GO:0098791 | 0.0086171 |
| GO:CC | lamellipodium | GO:0030027 | 0.0105525 |
| GO:CC | large ribosomal subunit | GO:0015934 | 0.0126157 |
| GO:CC | cortical actin cytoskeleton | GO:0030864 | 0.0133284 |
| GO:CC | organellar ribosome | GO:0000313 | 0.0133284 |
| GO:CC | mitochondrial ribosome | GO:0005761 | 0.0133284 |
| GO:CC | spindle | GO:0005819 | 0.0133284 |
| GO:CC | ATPase complex | GO:1904949 | 0.0148187 |
| GO:CC | catalytic step 2 spliceosome | GO:0071013 | 0.0148187 |
| GO:CC | mitochondrial respirasome | GO:0005746 | 0.0148187 |
| GO:CC | cell leading edge | GO:0031252 | 0.015122 |
| GO:CC | myosin complex | GO:0016459 | 0.0223729 |
| GO:CC | cytoplasmic vesicle membrane | GO:0030659 | 0.0233471 |
| GO:CC | cullin-RING ubiquitin ligase complex | GO:0031461 | 0.0233603 |
| GO:CC | Cul4-RING E3 ubiquitin ligase complex | GO:0080008 | 0.0234965 |
| GO:CC | mitotic spindle | GO:0072686 | 0.0240015 |
| GO:CC | sarcolemma | GO:0042383 | 0.0240015 |

| | | | |
|---|---|---|---|
| GO:CC | respiratory chain complex I | GO:0045271 | 0.0276796 |
| GO:CC | NADH dehydrogenase complex | GO:0030964 | 0.0276796 |
| GO:CC | mitochondrial respiratory chain complex I | GO:0005747 | 0.0276796 |
| GO:CC | vesicle membrane | GO:0012506 | 0.0334816 |
| GO:CC | mitochondrial outer membrane | GO:0005741 | 0.0337262 |
| GO:CC | anchoring junction | GO:0070161 | 0.0339589 |
| GO:CC | endoplasmic reticulum-Golgi intermediate compartment | GO:0005793 | 0.0348282 |
| GO:CC | cytosolic small ribosomal subunit | GO:0022627 | 0.0348282 |
| GO:CC | recycling endosome | GO:0055037 | 0.0348282 |
| GO:CC | chromosomal region | GO:0098687 | 0.0420025 |
| GO:CC | SWI/SNF superfamily-type complex | GO:0070603 | 0.0445528 |
| GO:CC | outer membrane | GO:0019867 | 0.0466654 |
| GO:CC | organelle outer membrane | GO:0031968 | 0.0466654 |
| GO:CC | heterochromatin | GO:0000792 | 0.0480823 |
