# Supplementary material for: Weighted gene co-expression network analysis identifies important modules and hub genes involved in the regulation of breast muscle yield in broilers
Source: Anim Biosci. 2024 Apr 25;37(10):1673–82. doi: 10.5713/ab.23.0548 (PMC11366510; doi:10.5713/ab.23.0548)
Supplement: Supplementary file 6 [file ab-23-0548-Supplementary-Table-6.pdf]

Table S6. Functional enrichment analysis of the genes in the skyblue3 module.

Category	Term description	Term ID	Adjusted p value	Genes
GO:BP	motile cilium assembly	GO:0044458	0.015767706	ENSGALG00010004386,ENSGALG00010007861,ENSGALG00010007862
