# Supplementary material for: Weighted gene co-expression network analysis identifies important modules and hub genes involved in the regulation of breast muscle yield in broilers
Source: Anim Biosci. 2024 Apr 25;37(10):1673–82. doi: 10.5713/ab.23.0548 (PMC11366510; doi:10.5713/ab.23.0548)
Supplement: Supplementary file 7 [file ab-23-0548-Supplementary-Table-7.pdf]

Table S7.The |KME| value of each gene in the turquoise and skyblue3 modules.

Gene ID	kME_MM.turquoise	kME_MM.skyblue3
ENSGALG00010000003	0.67713786	0.268869005
ENSGALG00010000005	0.182435892	0.111675254
ENSGALG00010000007	0.701844933	0.157349005
ENSGALG00010000008	0.684526008	0.242677865
ENSGALG00010000009	0.803826811	0.238213257
ENSGALG00010000011	0.648049638	0.125438727
ENSGALG00010000012	0.172212679	0.138142629
ENSGALG00010000013	0.121598278	0.093596252
ENSGALG00010000014	0.240397531	0.022084896
ENSGALG00010000015	0.066079265	0.176921541
ENSGALG00010000016	0.745184821	0.194745892
ENSGALG00010000017	0.643016394	0.079652908
ENSGALG00010000018	0.682282424	0.12175719
ENSGALG00010000019	0.224779305	0.192921885
ENSGALG00010000020	0.692641058	0.120689171
ENSGALG00010000022	0.57900545	0.110834363
ENSGALG00010000023	0.842137933	0.178854668
ENSGALG00010000024	0.83613675	0.206106559
ENSGALG00010000026	0.834068365	0.209176272
ENSGALG00010000028	0.757054671	0.140089859
ENSGALG00010000029	0.775204873	0.136692049
ENSGALG00010000033	0.804706882	0.178964005
ENSGALG00010000034	0.806636092	0.202310047
ENSGALG00010000035	0.675810485	0.003512569
ENSGALG00010000036	0.864362727	0.210032607
ENSGALG00010000037	0.646262723	0.254353035
ENSGALG00010000038	0.800739856	0.273352761
ENSGALG00010000039	0.946311036	0.292453163
ENSGALG00010000042	0.782584922	0.248525703
ENSGALG00010000043	0.538937469	0.101483349
ENSGALG00010000044	0.983924335	0.315610705
ENSGALG00010000045	0.73303003	0.093595803
ENSGALG00010000046	0.097915723	0.015123708
ENSGALG00010000047	0.901331555	0.20643188
ENSGALG00010000048	0.993780774	0.351032165
ENSGALG00010000049	0.961209591	0.391094849
ENSGALG00010000050	0.478907053	0.08575067
ENSGALG00010000051	0.341385833	0.036099822
ENSGALG00010000052	0.79627951	0.393446169
ENSGALG00010000053	0.494773155	0.269681888
ENSGALG00010000054	0.934613678	0.347151254
ENSGALG00010000055	0.348137151	0.079483386
ENSGALG00010000056	0.862541337	0.15130898
ENSGALG00010000058	0.393916642	0.109133222
ENSGALG00010000059	0.892840085	0.21548811
ENSGALG00010000060	0.939446462	0.303469055
ENSGALG00010000061	0.938597236	0.432841745
ENSGALG00010000062	0.182883847	0.160858452
ENSGALG00010000063	0.900819619	0.310191486
ENSGALG00010000065	0.506539912	0.043309882
ENSGALG00010000066	0.586929648	0.232480561
ENSGALG00010000068	0.371201294	0.154460096
ENSGALG00010000069	0.985074172	0.314422848
ENSGALG00010000070	0.460865763	0.02165537

ENSGALG00010000071	0.318121149	0.063067284
ENSGALG00010000072	0.840375586	0.098305482
ENSGALG00010000073	0.082576983	0.333392741
ENSGALG00010000074	0.482188149	0.036709511
ENSGALG00010000075	0.304017794	0.009211689
ENSGALG00010000076	0.680266948	0.217810446
ENSGALG00010000078	0.62186167	0.337567599
ENSGALG00010000081	0.99737335	0.312977789
ENSGALG00010000082	0.867423816	0.424618736
ENSGALG00010000084	0.299215241	0.118879997
ENSGALG00010000085	0.585306333	0.033479025
ENSGALG00010000086	0.059605451	0.275867318
ENSGALG00010000087	0.327901033	0.065172461
ENSGALG00010000088	0.805560626	0.209291854
ENSGALG00010000089	0.813731633	0.573335529
ENSGALG00010000090	0.088282936	0.168883153
ENSGALG00010000091	0.338822945	0.131496438
ENSGALG00010000092	0.969156581	0.303084956
ENSGALG00010000093	0.671240355	0.212128494
ENSGALG00010000094	0.201400243	0.317586472
ENSGALG00010000095	0.244019886	0.18555773
ENSGALG00010000096	0.218808132	0.266049458
ENSGALG00010000098	0.062106651	0.249684857
ENSGALG00010000099	0.022812732	0.098563748
ENSGALG00010000100	0.279607186	0.137322857
ENSGALG00010000101	0.280441633	0.389997873
ENSGALG00010000102	0.046953014	0.356333913
ENSGALG00010000103	0.621730248	0.342123731
ENSGALG00010000104	0.066574794	0.150135049
ENSGALG00010000105	0.792226181	0.027901748
ENSGALG00010000106	0.39454732	0.069859099
ENSGALG00010000107	0.098724135	0.186773211
ENSGALG00010000108	0.383538676	0.331753084
ENSGALG00010000109	0.175825467	0.099421526
ENSGALG00010000110	0.379524334	0.186886972
ENSGALG00010000111	0.397706705	0.359896341
ENSGALG00010000112	0.264486095	0.170965971
ENSGALG00010000113	0.19148267	0.031974627
ENSGALG00010000114	0.283168602	0.205384802
ENSGALG00010000116	0.534486748	0.534472143
ENSGALG00010000117	0.090280663	0.190361202
ENSGALG00010000119	0.1981871	0.0876879
ENSGALG00010000120	0.086362913	0.230559941
ENSGALG00010000121	0.554522606	0.222465351
ENSGALG00010000122	0.201958335	0.258758598
ENSGALG00010000123	0.470005901	0.319750703
ENSGALG00010000124	0.302843886	0.39866435
ENSGALG00010000125	0.588613125	0.393640326
ENSGALG00010000126	0.950876764	0.314298462
ENSGALG00010000127	0.935269127	0.407157222
ENSGALG00010000129	0.61017249	0.176261391
ENSGALG00010000130	0.044822307	0.068184874
ENSGALG00010000132	0.385848762	0.189101471
ENSGALG00010000133	0.947554712	0.350858375
ENSGALG00010000134	0.968246621	0.282973321
ENSGALG00010000135	0.959211403	0.315325082

ENSGALG00010000137	0.842033829	0.561701762
ENSGALG00010000138	0.779177303	0.183853187
ENSGALG00010000139	0.020868334	0.340711942
ENSGALG00010000140	0.730189687	0.179209526
ENSGALG00010000141	0.890773417	0.332502139
ENSGALG00010000142	0.549070007	0.106267265
ENSGALG00010000143	0.876868821	0.381793433
ENSGALG00010000145	0.841891243	0.284419506
ENSGALG00010000146	0.073990821	0.033900297
ENSGALG00010000147	0.621842521	0.246890789
ENSGALG00010000148	0.749289356	0.464781896
ENSGALG00010000149	0.89723681	0.253136365
ENSGALG00010000150	0.981303167	0.317835606
ENSGALG00010000151	0.952695574	0.31473792
ENSGALG00010000152	0.724232064	0.148590177
ENSGALG00010000153	0.998344957	0.33341863
ENSGALG00010000155	0.916616961	0.433283083
ENSGALG00010000156	0.219928655	0.14384675
ENSGALG00010000160	0.394327047	0.133628127
ENSGALG00010000161	0.267602661	0.383260926
ENSGALG00010000163	0.409436406	0.157934013
ENSGALG00010000165	0.177578803	0.344296519
ENSGALG00010000166	0.947942718	0.320109688
ENSGALG00010000168	0.016918137	0.111182187
ENSGALG00010000169	0.72526171	0.268993078
ENSGALG00010000170	0.633549698	0.078623057
ENSGALG00010000171	0.944412875	0.218348315
ENSGALG00010000173	0.98710556	0.291826512
ENSGALG00010000174	0.823200439	0.163955708
ENSGALG00010000175	0.282754916	0.323313762
ENSGALG00010000176	0.345519434	0.151812649
ENSGALG00010000177	0.894020103	0.219313866
ENSGALG00010000178	0.38813597	0.156768404
ENSGALG00010000179	0.900929472	0.248380947
ENSGALG00010000180	0.193820404	0.329577812
ENSGALG00010000181	0.120830886	0.22671206
ENSGALG00010000183	0.562325151	0.27845489
ENSGALG00010000185	0.265558089	0.028821141
ENSGALG00010000186	0.975063698	0.342381411
ENSGALG00010000187	0.181784172	0.269566066
ENSGALG00010000189	0.500124249	0.384696218
ENSGALG00010000191	0.654347541	0.346957861
ENSGALG00010000192	0.032664121	0.12367773
ENSGALG00010000193	0.332381325	0.155868563
ENSGALG00010000194	0.847600602	0.3646619
ENSGALG00010000195	0.85905845	0.343014695
ENSGALG00010000197	0.025104256	0.066609323
ENSGALG00010000198	0.271315151	0.244987657
ENSGALG00010000199	0.426881079	0.044800078
ENSGALG00010000200	0.178760711	0.066310067
ENSGALG00010000201	0.277566769	0.107857127
ENSGALG00010000202	0.975167672	0.237285493
ENSGALG00010000205	0.541129824	0.152683563
ENSGALG00010000206	0.159298216	0.098116394
ENSGALG00010000210	0.740110011	0.066156881
ENSGALG00010000215	0.533229281	0.314863703

ENSGALG00010000216	0.008963608	0.336983718
ENSGALG00010000217	0.216693802	0.023288188
ENSGALG00010000218	0.292519347	0.251059971
ENSGALG00010000219	0.357310469	0.002025275
ENSGALG00010000220	0.311429086	0.017150986
ENSGALG00010000221	0.902789763	0.335253742
ENSGALG00010000222	0.474234478	0.079151133
ENSGALG00010000223	0.625245231	0.202124994
ENSGALG00010000224	0.986808581	0.320707628
ENSGALG00010000225	0.208208723	0.078478438
ENSGALG00010000226	0.890126749	0.35918507
ENSGALG00010000227	0.872729957	0.321594645
ENSGALG00010000228	0.007149054	0.442043105
ENSGALG00010000229	0.738339183	0.287860154
ENSGALG00010000230	0.130352148	0.101811912
ENSGALG00010000231	0.906263036	0.326996238
ENSGALG00010000232	0.946182075	0.266611573
ENSGALG00010000233	0.957159178	0.34873544
ENSGALG00010000235	0.866956868	0.177786896
ENSGALG00010000236	0.800700993	0.321681539
ENSGALG00010000238	0.103188554	0.180603966
ENSGALG00010000241	0.154561313	0.365560538
ENSGALG00010000242	0.951333566	0.308757107
ENSGALG00010000244	0.586125024	0.199277277
ENSGALG00010000247	0.947766353	0.35586602
ENSGALG00010000248	0.383549736	0.180028334
ENSGALG00010000254	0.479323659	0.118157845
ENSGALG00010000255	0.988523079	0.323807244
ENSGALG00010000256	0.496903148	0.147876642
ENSGALG00010000258	0.285272007	0.109169955
ENSGALG00010000261	0.621305532	0.078798209
ENSGALG00010000263	0.869613551	0.127901101
ENSGALG00010000266	0.048522101	0.113076891
ENSGALG00010000267	0.947895777	0.268612047
ENSGALG00010000269	0.436114363	0.157360596
ENSGALG00010000271	0.607836476	0.446182268
ENSGALG00010000272	0.644490714	0.514098843
ENSGALG00010000273	0.302436477	0.152549183
ENSGALG00010000277	0.555340376	0.12165401
ENSGALG00010000279	0.642919184	0.306092605
ENSGALG00010000280	0.795596161	0.067331988
ENSGALG00010000281	0.93547088	0.351570104
ENSGALG00010000283	0.589405713	0.26145117
ENSGALG00010000284	0.750980331	0.317828426
ENSGALG00010000285	0.984818432	0.328978033
ENSGALG00010000286	0.917316202	0.257099718
ENSGALG00010000287	0.145814573	0.11618022
ENSGALG00010000288	0.556835512	0.005107816
ENSGALG00010000289	0.921269203	0.297905093
ENSGALG00010000291	0.795524011	0.290629895
ENSGALG00010000292	0.095740687	0.357179994
ENSGALG00010000293	0.744980138	0.166477753
ENSGALG00010000294	0.701978184	0.014254704
ENSGALG00010000295	0.913605083	0.340117613
ENSGALG00010000297	0.694260876	0.338915472
ENSGALG00010000298	0.906331397	0.38917595

ENSGALG00010000300	0.64029768	0.244040708
ENSGALG00010000301	0.978568254	0.350169096
ENSGALG00010000302	0.628657002	0.314299243
ENSGALG00010000303	0.763141582	0.154461705
ENSGALG00010000304	0.297090418	0.136289521
ENSGALG00010000305	0.520987419	0.162604164
ENSGALG00010000306	0.738765512	0.202968517
ENSGALG00010000307	0.917166395	0.314454344
ENSGALG00010000308	0.631444371	0.068901316
ENSGALG00010000310	0.636982726	0.319093774
ENSGALG00010000312	0.0178609	0.012529185
ENSGALG00010000314	0.531008016	0.230390244
ENSGALG00010000315	0.260516212	0.164924477
ENSGALG00010000316	0.51727901	0.272491674
ENSGALG00010000317	0.49081902	0.149174245
ENSGALG00010000320	0.406311723	0.009117756
ENSGALG00010000321	0.239043905	0.151562739
ENSGALG00010000323	0.848915605	0.223748466
ENSGALG00010000324	0.286582928	0.136904406
ENSGALG00010000325	0.104724503	0.169287249
ENSGALG00010000327	0.245700908	0.010899108
ENSGALG00010000328	0.149638706	0.188230914
ENSGALG00010000331	0.830320193	0.308847496
ENSGALG00010000333	0.267089704	0.089862198
ENSGALG00010000334	0.409436406	0.157934013
ENSGALG00010000335	0.30082842	0.188633163
ENSGALG00010000337	0.132246131	0.188493184
ENSGALG00010000338	0.679494568	0.206807442
ENSGALG00010000340	0.93490093	0.357239034
ENSGALG00010000341	0.331326123	0.219257781
ENSGALG00010000342	0.245700908	0.010899108
ENSGALG00010000344	0.074387989	0.096852877
ENSGALG00010000346	0.902883484	0.342295803
ENSGALG00010000347	0.383605605	0.025332023
ENSGALG00010000349	0.88785872	0.281688769
ENSGALG00010000350	0.909077042	0.275152935
ENSGALG00010000351	0.721190767	0.277840767
ENSGALG00010000353	0.973269824	0.28516072
ENSGALG00010000356	0.969525401	0.342239339
ENSGALG00010000357	0.458831118	0.263412792
ENSGALG00010000359	0.138718324	0.153188062
ENSGALG00010000360	0.210180491	0.104336086
ENSGALG00010000361	0.36853544	0.277846699
ENSGALG00010000362	0.893683345	0.142865183
ENSGALG00010000363	0.590613907	0.163920133
ENSGALG00010000364	0.217147198	0.137739361
ENSGALG00010000365	0.491926949	0.015334533
ENSGALG00010000366	0.802488844	0.21909053
ENSGALG00010000367	0.211037562	0.03617017
ENSGALG00010000368	0.503243231	0.114904165
ENSGALG00010000370	0.264239718	0.073427965
ENSGALG00010000371	0.700413249	0.371073562
ENSGALG00010000372	0.750875156	0.17447336
ENSGALG00010000373	0.194179602	0.152508466
ENSGALG00010000374	0.145483169	0.072714909
ENSGALG00010000375	0.196287929	0.062576308

ENSGALG00010000376	0.819868533	0.451972218
ENSGALG00010000377	0.69685578	0.126793557
ENSGALG00010000378	0.435073584	0.297152464
ENSGALG00010000381	0.283316707	0.15918372
ENSGALG00010000382	0.02585932	0.368605526
ENSGALG00010000383	0.394927857	0.119113854
ENSGALG00010000384	0.003386147	0.100344536
ENSGALG00010000386	0.37760366	0.406892287
ENSGALG00010000387	0.336071976	0.150227555
ENSGALG00010000388	0.936179	0.231019826
ENSGALG00010000389	0.327120709	0.228008616
ENSGALG00010000390	0.124380843	0.190094924
ENSGALG00010000391	0.221145033	0.188575242
ENSGALG00010000392	0.105124449	0.249309416
ENSGALG00010000393	0.181816966	0.434706795
ENSGALG00010000395	0.926451303	0.238654121
ENSGALG00010000396	0.129117615	0.180464336
ENSGALG00010000397	0.120945868	0.062169713
ENSGALG00010000398	0.170088117	0.205267558
ENSGALG00010000399	0.310605471	0.070195065
ENSGALG00010000400	0.934884133	0.233501688
ENSGALG00010000401	0.166483129	0.280094128
ENSGALG00010000402	0.579422546	0.139894527
ENSGALG00010000403	0.326732486	0.465221416
ENSGALG00010000404	0.392961713	0.112028159
ENSGALG00010000405	0.330907906	0.058726588
ENSGALG00010000406	0.862346384	0.298607036
ENSGALG00010000407	0.981577317	0.351764558
ENSGALG00010000408	0.994157083	0.357701998
ENSGALG00010000409	0.979343569	0.417914862
ENSGALG00010000410	0.982203399	0.419672132
ENSGALG00010000411	0.37194389	0.094802596
ENSGALG00010000412	0.295395629	0.203024862
ENSGALG00010000413	0.579554629	0.139138574
ENSGALG00010000414	0.21207558	0.182396415
ENSGALG00010000415	0.093638077	0.188510707
ENSGALG00010000416	0.247065276	0.177173488
ENSGALG00010000417	0.394271748	0.144488011
ENSGALG00010000418	0.262023223	0.070344809
ENSGALG00010000419	0.020037043	0.234563583
ENSGALG00010000420	0.257730745	0.073942076
ENSGALG00010000421	0.25876306	0.010094137
ENSGALG00010000422	0.299300116	0.20482601
ENSGALG00010000423	0.947251556	0.42230018
ENSGALG00010000424	0.055997904	0.195716958
ENSGALG00010000426	0.407111757	0.409525857
ENSGALG00010000427	0.734063942	0.178410706
ENSGALG00010000428	0.826874641	0.155150376
ENSGALG00010000429	0.608997345	0.112876731
ENSGALG00010000431	0.768778609	0.256134465
ENSGALG00010000432	0.976031569	0.292008297
ENSGALG00010000433	0.02076408	0.146491622
ENSGALG00010000434	0.929227567	0.286441968
ENSGALG00010000435	0.020579242	0.106743297
ENSGALG00010000436	0.904642271	0.525530322
ENSGALG00010000440	0.083780209	0.084706185

ENSGALG00010000441	0.086400909	0.181501363
ENSGALG00010000442	0.587983253	0.04387958
ENSGALG00010000443	0.955297529	0.266418482
ENSGALG00010000444	0.841062506	0.178553184
ENSGALG00010000445	0.981660531	0.330554344
ENSGALG00010000446	0.549665779	0.171936703
ENSGALG00010000447	0.994611335	0.336856003
ENSGALG00010000448	0.873148724	0.291868435
ENSGALG00010000449	0.990953737	0.2879844
ENSGALG00010000450	0.45035876	0.008357521
ENSGALG00010000451	0.675363589	0.215639766
ENSGALG00010000452	0.992784145	0.32360379
ENSGALG00010000453	0.988518243	0.309959801
ENSGALG00010000454	0.106855453	0.091596959
ENSGALG00010000456	0.157698299	0.38525341
ENSGALG00010000457	0.812853405	0.100064124
ENSGALG00010000458	0.872786117	0.239301454
ENSGALG00010000460	0.104853295	0.446721456
ENSGALG00010000462	0.304885028	0.17446583
ENSGALG00010000463	0.266137114	0.107784983
ENSGALG00010000465	0.966901785	0.306579129
ENSGALG00010000466	0.655902536	0.297707628
ENSGALG00010000467	0.802220433	0.20054285
ENSGALG00010000468	0.935655336	0.280926199
ENSGALG00010000469	0.963861279	0.305313985
ENSGALG00010000470	0.42337818	0.012651876
ENSGALG00010000471	0.031643804	0.167181631
ENSGALG00010000472	0.481888728	0.178304379
ENSGALG00010000473	0.081664875	0.26323161
ENSGALG00010000474	0.594153712	0.200501789
ENSGALG00010000475	0.345897105	0.036045218
ENSGALG00010000476	0.996625865	0.33136723
ENSGALG00010000477	0.091350892	0.530184218
ENSGALG00010000478	0.587188482	0.373477545
ENSGALG00010000479	0.974714037	0.316770077
ENSGALG00010000480	0.448350069	0.332222985
ENSGALG00010000481	0.388181886	0.168275567
ENSGALG00010000483	0.862150391	0.115535851
ENSGALG00010000484	0.854753159	0.354059398
ENSGALG00010000485	0.950650414	0.327233782
ENSGALG00010000486	0.610919343	0.023020787
ENSGALG00010000491	0.91990148	0.297954191
ENSGALG00010000495	0.231623601	0.005768907
ENSGALG00010000496	0.382977183	0.103780214
ENSGALG00010000498	0.77724314	0.257618414
ENSGALG00010000501	0.963680723	0.260893718
ENSGALG00010000505	0.654906861	0.226263439
ENSGALG00010000506	0.966460017	0.382171684
ENSGALG00010000507	0.888058054	0.330235254
ENSGALG00010000508	0.687477304	0.003186649
ENSGALG00010000510	0.142384954	0.051077075
ENSGALG00010000511	0.842492527	0.406411063
ENSGALG00010000516	0.578343939	0.29409458
ENSGALG00010000517	0.319320077	0.024051854
ENSGALG00010000521	0.245167	0.188148786
ENSGALG00010000522	0.172310245	0.136743136

ENSGALG00010000523	0.400778541	0.133797302
ENSGALG00010000525	0.432580607	0.042291542
ENSGALG00010000526	0.444346554	0.082214018
ENSGALG00010000527	0.134995584	0.230697158
ENSGALG00010000529	0.942965842	0.227664188
ENSGALG00010000532	0.899563695	0.277536084
ENSGALG00010000535	0.076380867	0.061149184
ENSGALG00010000539	0.181706745	0.341650625
ENSGALG00010000541	0.402821949	0.052686556
ENSGALG00010000542	0.38788653	0.126003537
ENSGALG00010000543	0.468895336	0.175379239
ENSGALG00010000544	0.645202228	0.147673453
ENSGALG00010000546	0.36047432	0.318888467
ENSGALG00010000547	0.022250526	0.036379144
ENSGALG00010000548	0.646170342	0.255506712
ENSGALG00010000549	0.247124405	0.247622785
ENSGALG00010000551	0.828964063	0.302895954
ENSGALG00010000552	0.373994933	0.108284034
ENSGALG00010000553	0.579206564	0.312995359
ENSGALG00010000554	0.02302955	0.390011841
ENSGALG00010000555	0.96316624	0.29587699
ENSGALG00010000556	0.537607893	0.079681093
ENSGALG00010000558	0.116253797	0.491843798
ENSGALG00010000559	0.318187606	0.085406555
ENSGALG00010000560	0.154632309	0.150717431
ENSGALG00010000561	0.183612595	0.133653163
ENSGALG00010000562	0.920879064	0.347307606
ENSGALG00010000563	0.271882331	0.119653794
ENSGALG00010000564	0.847269166	0.254778181
ENSGALG00010000565	0.995454723	0.336726983
ENSGALG00010000566	0.156792161	0.045135206
ENSGALG00010000567	0.627687297	0.307967408
ENSGALG00010000569	0.762342016	0.455683707
ENSGALG00010000570	0.845653022	0.469071604
ENSGALG00010000571	0.996197235	0.335893911
ENSGALG00010000573	0.048271294	0.126898884
ENSGALG00010000574	0.987751774	0.318694309
ENSGALG00010000575	0.68788406	0.48094575
ENSGALG00010000576	0.91562357	0.35018049
ENSGALG00010000577	0.952236147	0.417337059
ENSGALG00010000578	0.978639218	0.375669165
ENSGALG00010000579	0.338966875	0.12816707
ENSGALG00010000580	0.584384538	0.399805396
ENSGALG00010000581	0.959991064	0.342699077
ENSGALG00010000582	0.368780246	0.322100778
ENSGALG00010000583	0.699233973	0.270054963
ENSGALG00010000584	0.774122689	0.271761379
ENSGALG00010000585	0.627414789	0.093589425
ENSGALG00010000586	0.978122079	0.315587741
ENSGALG00010000587	0.419870392	0.128064503
ENSGALG00010000588	0.453020425	0.011538766
ENSGALG00010000589	0.967576976	0.384485686
ENSGALG00010000590	0.345147794	0.041702059
ENSGALG00010000591	0.988126694	0.325331186
ENSGALG00010000592	0.238895258	0.16534526
ENSGALG00010000593	0.366069181	0.002907495

ENSGALG00010000595	0.98332239	0.306634277
ENSGALG00010000596	0.69236614	0.184504914
ENSGALG00010000598	0.520253554	0.153938583
ENSGALG00010000599	0.936747728	0.289864132
ENSGALG00010000601	0.737347657	0.289061616
ENSGALG00010000602	0.172936343	0.218353208
ENSGALG00010000603	0.927067408	0.370554232
ENSGALG00010000604	0.568660448	0.208043299
ENSGALG00010000605	0.394820712	0.156611869
ENSGALG00010000606	0.972092281	0.359653839
ENSGALG00010000607	0.469617399	0.231538221
ENSGALG00010000608	0.38169401	0.076457517
ENSGALG00010000610	0.91907321	0.368014744
ENSGALG00010000612	0.699501891	0.009452877
ENSGALG00010000613	0.952384794	0.286378448
ENSGALG00010000614	0.962570492	0.33738805
ENSGALG00010000615	0.520826681	0.140755019
ENSGALG00010000616	0.947548247	0.283580689
ENSGALG00010000618	0.61067623	0.283350381
ENSGALG00010000619	0.633441073	0.0489143
ENSGALG00010000620	0.161040694	0.079968341
ENSGALG00010000621	0.238320357	0.103958319
ENSGALG00010000622	0.277566769	0.107857127
ENSGALG00010000623	0.681903637	0.203417202
ENSGALG00010000625	0.605768311	0.322652733
ENSGALG00010000626	0.147937611	0.156140405
ENSGALG00010000627	0.363762424	0.166941682
ENSGALG00010000628	0.609129013	0.204497595
ENSGALG00010000629	0.104271551	0.170685281
ENSGALG00010000630	0.461217267	0.267499101
ENSGALG00010000632	0.48488446	0.171836785
ENSGALG00010000633	0.090903431	0.151998548
ENSGALG00010000634	0.055432623	0.187407906
ENSGALG00010000635	0.550509161	0.62058635
ENSGALG00010000636	0.848234789	0.106807088
ENSGALG00010000637	0.264239718	0.073427965
ENSGALG00010000638	0.362492797	0.140212263
ENSGALG00010000639	0.9921548	0.341279475
ENSGALG00010000641	0.645531399	0.358268489
ENSGALG00010000642	0.394927857	0.119113854
ENSGALG00010000643	0.810898687	0.323100391
ENSGALG00010000645	0.812790402	0.337156886
ENSGALG00010000646	0.506904213	0.179461729
ENSGALG00010000647	0.246020753	0.082628377
ENSGALG00010000648	0.06499139	0.167344773
ENSGALG00010000649	0.66200691	0.187107001
ENSGALG00010000650	0.954937182	0.272482388
ENSGALG00010000651	0.194016078	0.270879469
ENSGALG00010000652	0.907867796	0.261489786
ENSGALG00010000653	0.709300923	0.184755225
ENSGALG00010000654	0.123086793	0.031111915
ENSGALG00010000655	0.635952182	0.2006274
ENSGALG00010000656	0.36303177	0.201320994
ENSGALG00010000657	0.010732765	0.321159117
ENSGALG00010000658	0.392973346	0.181481667
ENSGALG00010000659	0.124919692	0.361760437

ENSGALG00010000660	0.886873885	0.228665282
ENSGALG00010000661	0.377563732	0.136077675
ENSGALG00010000662	0.041905319	0.297804681
ENSGALG00010000663	0.833589651	0.216340671
ENSGALG00010000665	0.503056376	0.168109625
ENSGALG00010000666	0.907833861	0.545986759
ENSGALG00010000668	0.456082258	0.383423111
ENSGALG00010000670	0.683297727	0.223694371
ENSGALG00010000672	0.691711392	0.12319543
ENSGALG00010000673	0.925187651	0.282651358
ENSGALG00010000674	0.3363146	0.047049133
ENSGALG00010000675	0.265558089	0.028821141
ENSGALG00010000676	0.474010947	0.158763591
ENSGALG00010000677	0.070583826	0.069304593
ENSGALG00010000680	0.335041244	0.010885792
ENSGALG00010000681	0.603034965	0.234890225
ENSGALG00010000682	0.200570274	0.091667991
ENSGALG00010000683	0.366667574	0.096374266
ENSGALG00010000684	0.895010567	0.307481518
ENSGALG00010000685	0.423755986	0.0644898
ENSGALG00010000686	0.401391482	0.190857678
ENSGALG00010000687	0.952854261	0.182320509
ENSGALG00010000688	0.098229179	0.16563049
ENSGALG00010000689	0.489118905	0.163768703
ENSGALG00010000691	0.00263811	0.1342553
ENSGALG00010000692	0.392414199	0.075936062
ENSGALG00010000693	0.279930061	0.000456945
ENSGALG00010000694	0.653508364	0.261197252
ENSGALG00010000695	0.235007016	0.520920843
ENSGALG00010000696	0.650081836	0.429237283
ENSGALG00010000697	0.001993906	0.209044184
ENSGALG00010000698	0.794320313	0.014161132
ENSGALG00010000699	0.693939385	0.191447831
ENSGALG00010000700	0.374802591	0.002966083
ENSGALG00010000701	0.953655601	0.324813406
ENSGALG00010000702	0.980102167	0.370022222
ENSGALG00010000703	0.050698842	0.088610378
ENSGALG00010000704	0.864352457	0.221195396
ENSGALG00010000705	0.509223733	0.351645918
ENSGALG00010000706	0.830040398	0.514864209
ENSGALG00010000707	0.839849843	0.375525844
ENSGALG00010000708	0.736365326	0.25891245
ENSGALG00010000709	0.591457269	0.165820057
ENSGALG00010000710	0.381323895	0.041653817
ENSGALG00010000711	0.916667487	0.409667527
ENSGALG00010000712	0.423159897	0.191787992
ENSGALG00010000713	0.09124281	0.217585266
ENSGALG00010000714	0.841964859	0.256076579
ENSGALG00010000715	0.939832413	0.293353973
ENSGALG00010000716	0.323935392	0.212341937
ENSGALG00010000717	0.895809952	0.237590858
ENSGALG00010000718	0.934240912	0.342755097
ENSGALG00010000719	0.327987687	0.332338387
ENSGALG00010000720	0.215414603	0.451410557
ENSGALG00010000721	0.771005388	0.227462791
ENSGALG00010000722	0.878191403	0.328095899

ENSGALG00010000724	0.683246317	0.3846186
ENSGALG00010000725	0.096642349	0.170010693
ENSGALG00010000726	0.394352978	0.05219725
ENSGALG00010000727	0.18791545	0.20047248
ENSGALG00010000728	0.748904135	0.01633287
ENSGALG00010000729	0.988579983	0.295415102
ENSGALG00010000730	0.98262122	0.319541835
ENSGALG00010000731	0.413156623	0.241050245
ENSGALG00010000732	0.971065157	0.240358523
ENSGALG00010000733	0.28037105	0.288808031
ENSGALG00010000734	0.002467781	0.131919472
ENSGALG00010000735	0.183058837	0.21641214
ENSGALG00010000736	0.216529159	0.327121921
ENSGALG00010000737	0.55909867	0.071277594
ENSGALG00010000738	0.474047136	0.223221435
ENSGALG00010000739	0.090150977	0.176974358
ENSGALG00010000740	0.001499613	0.020129532
ENSGALG00010000741	0.918487194	0.21398698
ENSGALG00010000742	0.228746555	0.269680954
ENSGALG00010000743	0.105284843	0.087359016
ENSGALG00010000747	0.119904406	0.120665854
ENSGALG00010000749	0.267969515	0.064590254
ENSGALG00010000752	0.565140368	0.394231307
ENSGALG00010000753	0.935042601	0.334738667
ENSGALG00010000754	0.436859213	0.199712895
ENSGALG00010000757	0.687047095	0.266755815
ENSGALG00010000760	0.164483416	0.131728253
ENSGALG00010000761	0.385573219	0.076032555
ENSGALG00010000762	0.571882506	0.23817288
ENSGALG00010000763	0.752228981	0.272323458
ENSGALG00010000764	0.333467635	0.061370204
ENSGALG00010000765	0.357418515	0.3009244
ENSGALG00010000766	0.270369431	0.254012424
ENSGALG00010000767	0.021016026	0.035635705
ENSGALG00010000768	0.342776031	0.19828406
ENSGALG00010000769	0.187514554	0.108479243
ENSGALG00010000770	0.448184476	0.318943786
ENSGALG00010000771	0.218242443	0.279314896
ENSGALG00010000772	0.41855963	0.337359746
ENSGALG00010000773	0.270451009	0.090541667
ENSGALG00010000775	0.080735793	0.202761572
ENSGALG00010000776	0.11902281	0.267794419
ENSGALG00010000777	0.443230845	0.225509934
ENSGALG00010000778	0.395713962	0.527203642
ENSGALG00010000779	0.181629153	0.250726705
ENSGALG00010000780	0.694181203	0.238394251
ENSGALG00010000782	0.013327512	0.129304962
ENSGALG00010000783	0.985088462	0.307624023
ENSGALG00010000784	0.267583265	0.007934867
ENSGALG00010000785	0.957077405	0.2943191
ENSGALG00010000786	0.253334176	0.063564968
ENSGALG00010000787	0.738086028	0.211845171
ENSGALG00010000788	0.978999678	0.288883258
ENSGALG00010000789	0.589056809	0.166178418
ENSGALG00010000790	0.61776749	0.066831929
ENSGALG00010000791	0.948156337	0.36479823

ENSGALG00010000792	0.058386062	0.157650156
ENSGALG00010000794	0.962668202	0.317810165
ENSGALG00010000795	0.383069479	0.028024677
ENSGALG00010000796	0.08412472	0.338155568
ENSGALG00010000797	0.914767631	0.315076671
ENSGALG00010000798	0.83534366	0.330258398
ENSGALG00010000799	0.887222562	0.14227093
ENSGALG00010000800	0.635587301	0.175884052
ENSGALG00010000802	0.751996763	0.214813174
ENSGALG00010000803	0.85590894	0.236252842
ENSGALG00010000805	0.809138616	0.352042082
ENSGALG00010000806	0.84383152	0.27422129
ENSGALG00010000807	0.213840248	0.093335179
ENSGALG00010000808	0.391148148	0.524511369
ENSGALG00010000810	0.259533627	0.028380018
ENSGALG00010000811	0.399285785	0.689270568
ENSGALG00010000812	0.513031935	0.256367961
ENSGALG00010000813	0.677221529	0.379329006
ENSGALG00010000814	0.709874932	0.187981738
ENSGALG00010000815	0.599776341	0.336749576
ENSGALG00010000816	0.973445917	0.274384755
ENSGALG00010000817	0.929520217	0.333808601
ENSGALG00010000818	0.887661321	0.402140917
ENSGALG00010000819	0.685234164	0.065584393
ENSGALG00010000820	0.960827382	0.32180376
ENSGALG00010000821	0.930974551	0.400783587
ENSGALG00010000822	0.976024795	0.32163849
ENSGALG00010000823	0.050286675	0.151020351
ENSGALG00010000829	0.277566769	0.107857127
ENSGALG00010000835	0.381352663	0.078374659
ENSGALG00010000841	0.208201679	0.079142709
ENSGALG00010000843	0.94501108	0.379866654
ENSGALG00010000853	0.85802413	0.048866103
ENSGALG00010000855	0.306689429	0.364030294
ENSGALG00010000857	0.969540988	0.204086831
ENSGALG00010000858	0.433078375	0.306178743
ENSGALG00010000859	0.47819073	0.311726557
ENSGALG00010000861	0.246497798	0.036590263
ENSGALG00010000863	0.986492888	0.341331791
ENSGALG00010000864	0.925402156	0.30812881
ENSGALG00010000865	0.344387678	0.20026329
ENSGALG00010000867	0.162997499	0.208257899
ENSGALG00010000868	0.557336628	0.180668759
ENSGALG00010000869	0.947530632	0.370796365
ENSGALG00010000870	0.851128126	0.159591544
ENSGALG00010000872	0.352609914	0.106490996
ENSGALG00010000873	0.61122215	0.089160859
ENSGALG00010000874	0.39155441	0.07460765
ENSGALG00010000880	0.796328458	0.249156219
ENSGALG00010000885	0.768051054	0.23400839
ENSGALG00010000887	0.648259535	0.169506222
ENSGALG00010000888	0.500902519	0.568238169
ENSGALG00010000896	0.38480368	0.180523942
ENSGALG00010000897	0.748632226	0.08912014
ENSGALG00010000901	0.251162536	0.205519984
ENSGALG00010000902	0.264239718	0.073427965

ENSGALG00010000909	0.350862304	0.103149209
ENSGALG00010000911	0.231348001	0.150820468
ENSGALG00010000913	0.671374452	0.272693857
ENSGALG00010000914	0.113066118	0.064825726
ENSGALG00010000916	0.758940101	0.235566598
ENSGALG00010000923	0.082559758	0.13783377
ENSGALG00010000925	0.203991683	0.058221925
ENSGALG00010000926	0.563723727	0.482133449
ENSGALG00010000935	0.388773945	0.124549697
ENSGALG00010000936	0.297449338	0.622836754
ENSGALG00010000939	0.520878278	0.20346952
ENSGALG00010000941	0.491335246	0.181035858
ENSGALG00010000945	0.577297802	0.200688425
ENSGALG00010000949	0.738186813	0.250919862
ENSGALG00010000950	0.667960762	0.235327929
ENSGALG00010000952	0.271701261	0.072417625
ENSGALG00010000953	0.591738803	0.1859785
ENSGALG00010000955	0.012059079	0.093716972
ENSGALG00010000959	0.440461385	0.381737937
ENSGALG00010000962	0.005772544	0.38822656
ENSGALG00010000963	0.167414966	0.046468263
ENSGALG00010000966	0.249038452	0.000989663
ENSGALG00010000971	0.269800455	0.283735606
ENSGALG00010000985	0.189240572	0.04996112
ENSGALG00010000987	0.493098418	0.178919939
ENSGALG00010000988	0.396343306	0.152518913
ENSGALG00010000990	0.189240572	0.04996112
ENSGALG00010001001	0.190478359	0.148106019
ENSGALG00010001005	0.386311561	0.13402198
ENSGALG00010001013	0.44902746	0.081626457
ENSGALG00010001027	0.231046558	0.260485545
ENSGALG00010001032	0.586685326	0.215239956
ENSGALG00010001033	0.184279383	0.116835035
ENSGALG00010001049	0.964957502	0.320023008
ENSGALG00010001050	0.928214275	0.301063528
ENSGALG00010001051	0.917612333	0.282314381
ENSGALG00010001053	0.968750363	0.381529902
ENSGALG00010001055	0.583232869	0.362586675
ENSGALG00010001056	0.261823381	0.37057578
ENSGALG00010001057	0.737446774	0.179382874
ENSGALG00010001058	0.733811594	0.184568221
ENSGALG00010001059	0.175265693	0.184386624
ENSGALG00010001060	0.545321161	0.160014083
ENSGALG00010001061	0.716206608	0.272267913
ENSGALG00010001064	0.127700027	0.11783144
ENSGALG00010001065	0.801903936	0.503358997
ENSGALG00010001066	0.991502638	0.282822076
ENSGALG00010001067	0.223002124	0.106656109
ENSGALG00010001069	0.435970087	0.291087418
ENSGALG00010001070	0.294089512	0.008747479
ENSGALG00010001074	0.787700821	0.291581586
ENSGALG00010001075	0.109616876	0.103395309
ENSGALG00010001080	0.297090418	0.136289521
ENSGALG00010001081	0.687101185	0.371520664
ENSGALG00010001082	0.990239058	0.279211778
ENSGALG00010001083	0.009812001	0.5166245

ENSGALG00010001085	0.972513256	0.412092035
ENSGALG00010001086	0.219386918	0.079388121
ENSGALG00010001088	0.233289764	0.110817619
ENSGALG00010001098	0.026681103	0.120260695
ENSGALG00010001101	0.120169729	0.149706176
ENSGALG00010001104	0.984698301	0.301299835
ENSGALG00010001111	0.389170958	0.354003212
ENSGALG00010001112	0.813303316	0.41847683
ENSGALG00010001113	0.271659883	0.230327378
ENSGALG00010001114	0.02590484	0.138054293
ENSGALG00010001115	0.275961505	0.301638601
ENSGALG00010001116	0.948769618	0.300248965
ENSGALG00010001117	0.919682693	0.299868713
ENSGALG00010001118	0.922378407	0.134939957
ENSGALG00010001119	0.076976416	0.286338669
ENSGALG00010001120	0.051606007	0.145348651
ENSGALG00010001121	0.943740601	0.273213395
ENSGALG00010001122	0.482236926	0.097115734
ENSGALG00010001123	0.967295573	0.353427806
ENSGALG00010001124	0.687048326	0.281688812
ENSGALG00010001125	0.935719244	0.26379196
ENSGALG00010001126	0.175696376	0.006120429
ENSGALG00010001127	0.784856492	0.30519224
ENSGALG00010001128	0.520193903	0.118130497
ENSGALG00010001129	0.992061614	0.316685058
ENSGALG00010001130	0.495892281	0.20079274
ENSGALG00010001131	0.914622952	0.30794593
ENSGALG00010001132	0.951861344	0.291800479
ENSGALG00010001133	0.521356491	0.170676861
ENSGALG00010001134	0.622284656	0.403731657
ENSGALG00010001135	0.991581021	0.312692434
ENSGALG00010001136	0.925553306	0.324176354
ENSGALG00010001138	0.66307778	0.209719503
ENSGALG00010001140	0.97684698	0.254646761
ENSGALG00010001141	0.398911711	0.183310111
ENSGALG00010001142	0.919772349	0.234360435
ENSGALG00010001143	0.884412236	0.19602128
ENSGALG00010001144	0.972399666	0.342414652
ENSGALG00010001145	0.946759492	0.383228112
ENSGALG00010001146	0.923377753	0.293317784
ENSGALG00010001147	0.472042878	0.207071835
ENSGALG00010001148	0.357613792	0.16703301
ENSGALG00010001149	0.984192496	0.293131814
ENSGALG00010001150	0.957904527	0.172242387
ENSGALG00010001151	0.934982943	0.366997894
ENSGALG00010001152	0.949067357	0.318923599
ENSGALG00010001153	0.879765785	0.381299079
ENSGALG00010001154	0.81530522	0.338921279
ENSGALG00010001155	0.989072425	0.313753156
ENSGALG00010001156	0.865912654	0.445440567
ENSGALG00010001157	0.796191704	0.300757405
ENSGALG00010001158	0.837310052	0.30043804
ENSGALG00010001159	0.898002633	0.074854126
ENSGALG00010001160	0.645521039	0.356239991
ENSGALG00010001161	0.970128527	0.287721437
ENSGALG00010001162	0.616859806	0.535510285

ENSGALG00010001163	0.149813559	0.047275671
ENSGALG00010001164	0.984780592	0.299141616
ENSGALG00010001165	0.051337923	0.170540381
ENSGALG00010001166	0.77299726	0.351863144
ENSGALG00010001167	0.731530095	0.285063838
ENSGALG00010001168	0.206156519	0.119397962
ENSGALG00010001169	0.095978937	0.110042855
ENSGALG00010001170	0.428211648	0.32117406
ENSGALG00010001171	0.900067449	0.276225049
ENSGALG00010001172	0.881246719	0.43027212
ENSGALG00010001173	0.179652839	0.133643202
ENSGALG00010001174	0.808875597	0.222068899
ENSGALG00010001176	0.46947767	0.201570798
ENSGALG00010001177	0.927056218	0.448417871
ENSGALG00010001178	0.933129166	0.296842992
ENSGALG00010001179	0.929988047	0.233337476
ENSGALG00010001180	0.785641965	0.478364753
ENSGALG00010001181	0.878230584	0.340578104
ENSGALG00010001182	0.394044966	0.350180127
ENSGALG00010001183	0.978500661	0.293623614
ENSGALG00010001185	0.944866864	0.322406173
ENSGALG00010001187	0.168153459	0.063314642
ENSGALG00010001188	0.517374795	0.018986273
ENSGALG00010001190	0.505222578	0.245276513
ENSGALG00010001191	0.932180426	0.300261367
ENSGALG00010001192	0.990768009	0.288191265
ENSGALG00010001193	0.832068688	0.314031777
ENSGALG00010001194	0.748544357	0.509322413
ENSGALG00010001195	0.910082858	0.234165412
ENSGALG00010001196	0.992926457	0.328846159
ENSGALG00010001197	0.647607752	0.184440023
ENSGALG00010001198	0.704130601	0.258710142
ENSGALG00010001199	0.914571589	0.266624582
ENSGALG00010001200	0.984622378	0.304188605
ENSGALG00010001201	0.538280579	0.247935763
ENSGALG00010001202	0.71454387	0.209812007
ENSGALG00010001203	0.838745523	0.360706925
ENSGALG00010001204	0.828688091	0.429624782
ENSGALG00010001205	0.922323525	0.349362271
ENSGALG00010001206	0.981154768	0.258110575
ENSGALG00010001208	0.857665541	0.276486299
ENSGALG00010001209	0.302736334	0.117713433
ENSGALG00010001210	0.188953919	0.226739868
ENSGALG00010001212	0.134103264	0.157215272
ENSGALG00010001213	0.781688004	0.317418976
ENSGALG00010001214	0.456594099	0.21572423
ENSGALG00010001216	0.350713899	0.089775552
ENSGALG00010001218	0.846562874	0.344173492
ENSGALG00010001219	0.985792121	0.269022672
ENSGALG00010001220	0.959152049	0.383048598
ENSGALG00010001221	0.904407802	0.217282962
ENSGALG00010001222	0.879958089	0.505924045
ENSGALG00010001224	0.629624	0.351573056
ENSGALG00010001225	0.663540002	0.354328783
ENSGALG00010001226	0.98257608	0.241769331
ENSGALG00010001228	0.327558125	0.093845166

ENSGALG00010001229	0.138201654	0.2107437
ENSGALG00010001230	0.718794654	0.207387275
ENSGALG00010001231	0.779570166	0.181427586
ENSGALG00010001232	0.994750348	0.330891367
ENSGALG00010001233	0.66877584	0.174349239
ENSGALG00010001234	0.124155238	0.206242682
ENSGALG00010001235	0.578022969	0.186966325
ENSGALG00010001236	0.089535689	0.238912045
ENSGALG00010001237	0.636415415	0.183588997
ENSGALG00010001238	0.991766889	0.30523938
ENSGALG00010001239	0.698597704	0.095188817
ENSGALG00010001242	0.495248153	0.172088977
ENSGALG00010001244	0.096102445	0.254986452
ENSGALG00010001245	0.800180114	0.166228461
ENSGALG00010001248	0.385849847	0.039478853
ENSGALG00010001249	0.734534758	0.118691464
ENSGALG00010001251	0.702543597	0.430212373
ENSGALG00010001252	0.972945002	0.337721692
ENSGALG00010001253	0.398890409	0.187679725
ENSGALG00010001254	0.946694623	0.271518618
ENSGALG00010001258	0.544803247	0.003101316
ENSGALG00010001262	0.970369423	0.371720378
ENSGALG00010001264	0.937252582	0.15030668
ENSGALG00010001265	0.885396882	0.297019726
ENSGALG00010001267	0.926123366	0.30904842
ENSGALG00010001268	0.389213719	0.196872086
ENSGALG00010001270	0.985331603	0.313435589
ENSGALG00010001271	0.977539417	0.312090724
ENSGALG00010001273	0.381702061	0.247063428
ENSGALG00010001274	0.097086638	0.340317564
ENSGALG00010001276	0.335584327	0.130874902
ENSGALG00010001277	0.869303144	0.266645962
ENSGALG00010001279	0.982528745	0.300940182
ENSGALG00010001281	0.59905298	0.161305222
ENSGALG00010001283	0.916802651	0.35288319
ENSGALG00010001285	0.920085173	0.307635774
ENSGALG00010001286	0.005805532	0.058084496
ENSGALG00010001287	0.441547902	0.047460264
ENSGALG00010001288	0.886113925	0.296275427
ENSGALG00010001289	0.731831344	0.173624593
ENSGALG00010001291	0.97366064	0.319977699
ENSGALG00010001292	0.818457597	0.417622859
ENSGALG00010001294	0.010206714	0.093552912
ENSGALG00010001295	0.117267781	0.261824861
ENSGALG00010001297	0.285828431	0.622258824
ENSGALG00010001298	0.828083783	0.617238526
ENSGALG00010001300	0.801717418	0.027042427
ENSGALG00010001301	0.982051018	0.273447279
ENSGALG00010001302	0.270451009	0.090541667
ENSGALG00010001304	0.188246224	0.038506068
ENSGALG00010001305	0.732573064	0.332200565
ENSGALG00010001306	0.164830975	0.437922329
ENSGALG00010001307	0.662098907	0.008618864
ENSGALG00010001308	0.980799884	0.292671151
ENSGALG00010001309	0.823920396	0.444695425
ENSGALG00010001310	0.890005788	0.260834519

ENSGALG00010001311	0.480772329	0.183615671
ENSGALG00010001313	0.553111895	0.352845955
ENSGALG00010001318	0.487599556	0.252036288
ENSGALG00010001319	0.231178716	0.152554667
ENSGALG00010001320	0.930349368	0.256774094
ENSGALG00010001321	0.498819913	0.248903727
ENSGALG00010001322	0.830183703	0.398343795
ENSGALG00010001323	0.65984337	0.200693038
ENSGALG00010001325	0.625907938	0.218461345
ENSGALG00010001327	0.925473388	0.263999823
ENSGALG00010001328	0.909973808	0.329331783
ENSGALG00010001330	0.718194095	0.22608778
ENSGALG00010001331	0.800243761	0.312317286
ENSGALG00010001333	0.410004785	0.039327832
ENSGALG00010001334	0.967086644	0.395370252
ENSGALG00010001335	0.341050152	0.388294636
ENSGALG00010001338	0.256828349	0.119064955
ENSGALG00010001340	0.366810591	0.293049106
ENSGALG00010001341	0.465301817	0.194135365
ENSGALG00010001342	0.023279831	0.134374301
ENSGALG00010001343	0.636788244	0.187733018
ENSGALG00010001345	0.388181886	0.168275567
ENSGALG00010001346	0.324607775	0.333271277
ENSGALG00010001347	0.993282669	0.342037897
ENSGALG00010001349	0.638479388	0.429419475
ENSGALG00010001350	0.716773391	0.094512372
ENSGALG00010001351	0.996806728	0.329945704
ENSGALG00010001352	0.782925301	0.203341705
ENSGALG00010001353	0.979720931	0.30880498
ENSGALG00010001355	0.374500718	0.179369714
ENSGALG00010001356	0.277566769	0.107857127
ENSGALG00010001357	0.718275528	0.237906344
ENSGALG00010001358	0.062582014	0.03532597
ENSGALG00010001359	0.284539265	0.23921182
ENSGALG00010001360	0.578677231	0.222015005
ENSGALG00010001361	0.654232866	0.229730979
ENSGALG00010001363	0.919719389	0.32436915
ENSGALG00010001364	0.923156164	0.309833899
ENSGALG00010001365	0.440319304	0.047504119
ENSGALG00010001366	0.307350977	0.089221936
ENSGALG00010001367	0.253296461	0.15039664
ENSGALG00010001368	0.802456047	0.196400888
ENSGALG00010001369	0.547426952	0.40627332
ENSGALG00010001371	0.436203673	0.194694662
ENSGALG00010001372	0.738431394	0.730462124
ENSGALG00010001373	0.98415855	0.381033985
ENSGALG00010001374	0.265558089	0.028821141
ENSGALG00010001375	0.703548476	0.449087711
ENSGALG00010001376	0.707302773	0.061365446
ENSGALG00010001377	0.28880012	0.418789177
ENSGALG00010001378	0.659867928	0.022906883
ENSGALG00010001380	0.766937672	0.280414555
ENSGALG00010001381	0.341280867	0.461612355
ENSGALG00010001382	0.932050963	0.354779087
ENSGALG00010001384	0.422989801	0.19893951
ENSGALG00010001385	0.951012258	0.336988458

ENSGALG00010001386	0.056923469	0.063015847
ENSGALG00010001387	0.035158038	0.119871525
ENSGALG00010001388	0.713874085	0.401948637
ENSGALG00010001390	0.903862835	0.409230518
ENSGALG00010001395	0.820475903	0.331735209
ENSGALG00010001397	0.016651787	0.116556206
ENSGALG00010001398	0.950289177	0.38445702
ENSGALG00010001400	0.93933928	0.223920235
ENSGALG00010001401	0.262922434	0.138601464
ENSGALG00010001402	0.956883142	0.245549636
ENSGALG00010001404	0.021308589	0.164876077
ENSGALG00010001406	0.423518038	0.111172615
ENSGALG00010001407	0.984126133	0.324823941
ENSGALG00010001410	0.40135901	0.158038591
ENSGALG00010001411	0.942478125	0.294988164
ENSGALG00010001412	0.501234631	0.063163522
ENSGALG00010001414	0.666347626	0.443521705
ENSGALG00010001415	0.972938853	0.240063339
ENSGALG00010001416	0.248995672	0.13018674
ENSGALG00010001418	0.846371061	0.419319896
ENSGALG00010001419	0.225118777	0.607085928
ENSGALG00010001420	0.490141705	0.21869336
ENSGALG00010001422	0.914809266	0.159317897
ENSGALG00010001423	0.944134983	0.270737734
ENSGALG00010001425	0.677356602	0.04046161
ENSGALG00010001426	0.96613022	0.243951491
ENSGALG00010001427	0.997103776	0.320865225
ENSGALG00010001429	0.949815082	0.3912454
ENSGALG00010001432	0.42236827	0.123421679
ENSGALG00010001435	0.10546932	0.463318278
ENSGALG00010001436	0.987530324	0.265391071
ENSGALG00010001437	0.647799221	0.250577497
ENSGALG00010001440	0.977680392	0.375539164
ENSGALG00010001441	0.937396715	0.310301761
ENSGALG00010001442	0.939767268	0.303763435
ENSGALG00010001443	0.128324049	0.114818801
ENSGALG00010001445	0.393302098	0.14073727
ENSGALG00010001447	0.550217361	0.243867474
ENSGALG00010001448	0.282305083	0.450438657
ENSGALG00010001450	0.465585317	0.380861732
ENSGALG00010001451	0.399452143	0.144662594
ENSGALG00010001453	0.239238081	0.080377374
ENSGALG00010001454	0.936983561	0.308704061
ENSGALG00010001456	0.218666623	0.042118554
ENSGALG00010001457	0.868109943	0.430085416
ENSGALG00010001458	0.833577834	0.413733418
ENSGALG00010001460	0.951218696	0.298060856
ENSGALG00010001461	0.973075746	0.327390069
ENSGALG00010001462	0.830949376	0.180358492
ENSGALG00010001463	0.379228237	0.241303646
ENSGALG00010001465	0.981201705	0.29451684
ENSGALG00010001466	0.895565745	0.39630577
ENSGALG00010001468	0.956028122	0.386065616
ENSGALG00010001469	0.966142136	0.169205395
ENSGALG00010001470	0.185453987	0.140651822
ENSGALG00010001471	0.843460539	0.406809018

ENSGALG00010001475	0.290506567	0.103490682
ENSGALG00010001476	0.436601883	0.293204036
ENSGALG00010001477	0.694033419	0.420663464
ENSGALG00010001479	0.937190815	0.313391446
ENSGALG00010001480	0.403848943	0.500569498
ENSGALG00010001482	0.905495585	0.390399175
ENSGALG00010001483	0.77288074	0.28072602
ENSGALG00010001486	0.320368116	0.565662666
ENSGALG00010001487	0.700081473	0.342546251
ENSGALG00010001488	0.298217676	0.010575877
ENSGALG00010001491	0.967793297	0.347921802
ENSGALG00010001492	0.150508738	0.088614097
ENSGALG00010001493	0.002319943	0.47043011
ENSGALG00010001495	0.95650391	0.424737896
ENSGALG00010001496	0.980469216	0.332062559
ENSGALG00010001497	0.920410126	0.418410465
ENSGALG00010001499	0.598022336	0.232241162
ENSGALG00010001501	0.97743339	0.286296564
ENSGALG00010001504	0.14369534	0.072015335
ENSGALG00010001505	0.876580963	0.302289175
ENSGALG00010001506	0.533176119	0.090786783
ENSGALG00010001507	0.865638367	0.159465725
ENSGALG00010001508	0.037941496	0.119350675
ENSGALG00010001510	0.600551781	0.137480743
ENSGALG00010001511	0.582080219	0.174219582
ENSGALG00010001512	0.97350601	0.300087721
ENSGALG00010001513	0.551966622	0.181572207
ENSGALG00010001514	0.996378251	0.326298221
ENSGALG00010001516	0.640524801	0.048921745
ENSGALG00010001517	0.974767214	0.35562217
ENSGALG00010001518	0.965400208	0.332704703
ENSGALG00010001519	0.324368703	0.149815344
ENSGALG00010001520	0.153460003	0.294851523
ENSGALG00010001521	0.899899607	0.311711377
ENSGALG00010001524	0.984055507	0.33961461
ENSGALG00010001525	0.909570625	0.225743789
ENSGALG00010001526	0.989343726	0.327832331
ENSGALG00010001527	0.443102846	0.18234284
ENSGALG00010001528	0.335807076	0.117362214
ENSGALG00010001529	0.910854377	0.295168809
ENSGALG00010001531	0.411821802	0.235910741
ENSGALG00010001532	0.915059177	0.23622365
ENSGALG00010001533	0.668797069	0.320495311
ENSGALG00010001534	0.843200893	0.417165944
ENSGALG00010001535	0.116542343	0.132380464
ENSGALG00010001536	0.076005904	0.271008244
ENSGALG00010001537	0.86772009	0.204589202
ENSGALG00010001538	0.120761508	0.175560081
ENSGALG00010001540	0.934490807	0.267615286
ENSGALG00010001541	0.584861539	0.203315769
ENSGALG00010001542	0.948103249	0.243272543
ENSGALG00010001543	0.964750735	0.362011687
ENSGALG00010001545	0.583103108	0.191823962
ENSGALG00010001546	0.915532377	0.39636128
ENSGALG00010001547	0.963460076	0.33578262
ENSGALG00010001548	0.352313449	0.307693974

ENSGALG00010001551	0.988946626	0.336486925
ENSGALG00010001552	0.028270611	0.072037719
ENSGALG00010001553	0.252661814	0.074787931
ENSGALG00010001554	0.519587055	0.236739008
ENSGALG00010001556	0.673586903	0.232079701
ENSGALG00010001557	0.986750629	0.305576355
ENSGALG00010001558	0.344496224	0.336183711
ENSGALG00010001559	0.950416345	0.35954184
ENSGALG00010001560	0.462436136	0.24773472
ENSGALG00010001561	0.703258159	0.254663502
ENSGALG00010001563	0.387473629	0.117811347
ENSGALG00010001565	0.349705454	0.096072153
ENSGALG00010001567	0.540232484	0.245057796
ENSGALG00010001570	0.551027924	0.276687238
ENSGALG00010001573	0.48775796	0.189318879
ENSGALG00010001574	0.1147801	0.220113731
ENSGALG00010001575	0.858340807	0.271859779
ENSGALG00010001576	0.392894344	0.133607003
ENSGALG00010001577	0.295661329	0.120963357
ENSGALG00010001578	0.968720989	0.340570977
ENSGALG00010001579	0.374872911	0.136540542
ENSGALG00010001580	0.288795251	0.418569519
ENSGALG00010001581	0.062239627	0.209982906
ENSGALG00010001582	0.277566769	0.107857127
ENSGALG00010001583	0.022148785	0.104742673
ENSGALG00010001584	0.82482813	0.129304612
ENSGALG00010001585	0.661841721	0.017064297
ENSGALG00010001586	0.59501695	0.415542455
ENSGALG00010001587	0.100271802	0.218393156
ENSGALG00010001588	0.603726241	0.120235869
ENSGALG00010001590	0.527596017	0.10475464
ENSGALG00010001592	0.135367345	0.125686785
ENSGALG00010001593	0.937578499	0.464484255
ENSGALG00010001594	0.728516285	0.466525655
ENSGALG00010001595	0.682827949	0.390350763
ENSGALG00010001597	0.973219567	0.376141658
ENSGALG00010001598	0.143204296	0.159562468
ENSGALG00010001599	0.851379433	0.245586073
ENSGALG00010001600	0.279119344	0.061522979
ENSGALG00010001604	0.982650827	0.303196378
ENSGALG00010001605	0.359400241	0.191093242
ENSGALG00010001606	0.431195439	0.220837769
ENSGALG00010001607	0.379394869	0.170237467
ENSGALG00010001609	0.38485223	0.180377061
ENSGALG00010001610	0.479828286	0.188515705
ENSGALG00010001611	0.764324375	0.021834869
ENSGALG00010001612	0.992457233	0.341522387
ENSGALG00010001614	0.974457935	0.328911337
ENSGALG00010001616	0.886163884	0.316312033
ENSGALG00010001617	0.757975382	0.582841337
ENSGALG00010001618	0.926583983	0.25451735
ENSGALG00010001619	0.723661709	0.22067569
ENSGALG00010001620	0.862107596	0.436447512
ENSGALG00010001621	0.503281868	0.463210458
ENSGALG00010001622	0.438124912	0.15068347
ENSGALG00010001624	0.307131788	0.322787596

ENSGALG00010001625	0.475781087	0.149245505
ENSGALG00010001627	0.362859878	0.378888076
ENSGALG00010001628	0.94006089	0.205803618
ENSGALG00010001631	0.360541296	0.175664672
ENSGALG00010001632	0.07617994	0.020712389
ENSGALG00010001633	0.800513891	0.380052578
ENSGALG00010001635	0.828194899	0.249673442
ENSGALG00010001639	0.776995608	0.146484686
ENSGALG00010001640	0.890955123	0.251690966
ENSGALG00010001642	0.912000165	0.393563357
ENSGALG00010001647	0.810441135	0.256062754
ENSGALG00010001648	0.871939945	0.274022325
ENSGALG00010001649	0.692090679	0.001025962
ENSGALG00010001652	0.852191898	0.275451212
ENSGALG00010001653	0.36238761	0.287206834
ENSGALG00010001654	0.939415852	0.324840722
ENSGALG00010001657	0.940079655	0.42791466
ENSGALG00010001660	0.193165979	0.245970706
ENSGALG00010001661	0.825576724	0.251015792
ENSGALG00010001662	0.416210059	0.33118846
ENSGALG00010001665	0.745571439	0.341027931
ENSGALG00010001666	0.515759654	0.229055281
ENSGALG00010001667	0.285272007	0.109169955
ENSGALG00010001668	0.635424353	0.089695518
ENSGALG00010001670	0.805296009	0.409494313
ENSGALG00010001671	0.251051216	0.208832212
ENSGALG00010001672	0.837539475	0.392119044
ENSGALG00010001673	0.648023922	0.193727678
ENSGALG00010001674	0.841272945	0.341116862
ENSGALG00010001675	0.302191238	0.175836158
ENSGALG00010001677	0.941041319	0.182213826
ENSGALG00010001678	0.932075194	0.227871096
ENSGALG00010001679	0.485498341	0.186576831
ENSGALG00010001680	0.708369168	0.2761317
ENSGALG00010001681	0.446890226	0.274959567
ENSGALG00010001682	0.150765673	0.087775298
ENSGALG00010001686	0.912578149	0.354163694
ENSGALG00010001688	0.495218022	0.330308758
ENSGALG00010001689	0.910508735	0.394436243
ENSGALG00010001691	0.671928996	0.151950439
ENSGALG00010001692	0.979615338	0.313388132
ENSGALG00010001693	0.952768298	0.360028852
ENSGALG00010001694	0.734375216	0.283745957
ENSGALG00010001695	0.88867006	0.309414059
ENSGALG00010001696	0.722939931	0.322783248
ENSGALG00010001697	0.354771856	0.344581578
ENSGALG00010001698	0.943465414	0.349919751
ENSGALG00010001699	0.993535924	0.317787881
ENSGALG00010001700	0.673997719	0.125152105
ENSGALG00010001701	0.964644636	0.401155254
ENSGALG00010001702	0.861956278	0.133756866
ENSGALG00010001704	0.549089704	0.364964556
ENSGALG00010001705	0.856256999	0.13039973
ENSGALG00010001706	0.707221888	0.13986217
ENSGALG00010001707	0.964718515	0.304769688
ENSGALG00010001708	0.972037516	0.364517096

ENSGALG00010001709	0.405797923	0.13200104
ENSGALG00010001710	0.323348566	0.152618716
ENSGALG00010001711	0.909743307	0.26401307
ENSGALG00010001712	0.378005361	0.094833287
ENSGALG00010001713	0.808420242	0.21748494
ENSGALG00010001714	0.214067262	0.182518297
ENSGALG00010001715	0.33593534	0.034995438
ENSGALG00010001716	0.974845051	0.401855212
ENSGALG00010001717	0.363526723	0.21641949
ENSGALG00010001721	0.026180415	0.285752017
ENSGALG00010001725	0.896404801	0.305936477
ENSGALG00010001726	0.241229294	0.225366445
ENSGALG00010001727	0.010813399	0.280237107
ENSGALG00010001728	0.870943999	0.440839015
ENSGALG00010001729	0.82124044	0.344783346
ENSGALG00010001730	0.37615561	0.112727925
ENSGALG00010001731	0.961785686	0.229114516
ENSGALG00010001732	0.971071441	0.351708641
ENSGALG00010001737	0.311656077	0.221682868
ENSGALG00010001738	0.01352735	0.075448972
ENSGALG00010001741	0.963582256	0.356998252
ENSGALG00010001743	0.409436406	0.157934013
ENSGALG00010001744	0.088450995	0.244414017
ENSGALG00010001745	0.770875414	0.081221572
ENSGALG00010001746	0.374556039	0.35122871
ENSGALG00010001749	0.981221649	0.29927475
ENSGALG00010001750	0.487891197	0.181703046
ENSGALG00010001751	0.291720498	0.524744722
ENSGALG00010001752	0.680094122	0.172491979
ENSGALG00010001753	0.18307392	0.158560056
ENSGALG00010001754	0.501120352	0.469491098
ENSGALG00010001756	0.550202827	0.227020267
ENSGALG00010001758	0.275935626	0.291669028
ENSGALG00010001759	0.972237252	0.27605888
ENSGALG00010001760	0.854979333	0.315243439
ENSGALG00010001761	0.984237129	0.32799955
ENSGALG00010001763	0.769133784	0.401167732
ENSGALG00010001764	0.635288662	0.483721836
ENSGALG00010001766	0.16589826	0.352067461
ENSGALG00010001767	0.922869859	0.295352968
ENSGALG00010001770	0.4312115	0.482861159
ENSGALG00010001771	0.478884847	0.1712237
ENSGALG00010001773	0.58584331	0.36400428
ENSGALG00010001774	0.90801579	0.263089581
ENSGALG00010001776	0.103343486	0.250529151
ENSGALG00010001778	0.821416762	0.259999518
ENSGALG00010001779	0.404737955	0.305913177
ENSGALG00010001780	0.891664271	0.426914304
ENSGALG00010001783	0.564502616	0.289504862
ENSGALG00010001784	0.886486237	0.333825804
ENSGALG00010001785	0.78822063	0.319292664
ENSGALG00010001786	0.940612577	0.409160378
ENSGALG00010001788	0.577839928	0.068787042
ENSGALG00010001790	0.544042159	0.159165608
ENSGALG00010001793	0.385138438	0.009820524
ENSGALG00010001795	0.503202112	0.014344819

ENSGALG00010001796	0.872331453	0.289622813
ENSGALG00010001797	0.82389983	0.114835583
ENSGALG00010001798	0.955149841	0.327488856
ENSGALG00010001800	0.745877095	0.03402381
ENSGALG00010001801	0.893646174	0.302276342
ENSGALG00010001803	0.541415505	0.293197477
ENSGALG00010001805	0.781475409	0.31255792
ENSGALG00010001807	0.908742489	0.350500704
ENSGALG00010001808	0.172683158	0.184448338
ENSGALG00010001810	0.551973915	0.149221827
ENSGALG00010001811	0.087945553	0.216678174
ENSGALG00010001813	0.954711468	0.280990883
ENSGALG00010001816	0.236644512	0.207965177
ENSGALG00010001817	0.942321868	0.245376179
ENSGALG00010001819	0.977462427	0.360767814
ENSGALG00010001820	0.980082317	0.361287591
ENSGALG00010001822	0.575462371	0.392747094
ENSGALG00010001823	0.786144707	0.150797324
ENSGALG00010001825	0.980921806	0.305017738
ENSGALG00010001826	0.986686707	0.337345562
ENSGALG00010001827	0.408991391	0.672076699
ENSGALG00010001828	0.029558715	0.175619474
ENSGALG00010001829	0.936881613	0.2689309
ENSGALG00010001831	0.9659007	0.387049342
ENSGALG00010001832	0.825871495	0.326009906
ENSGALG00010001834	0.928915895	0.231797775
ENSGALG00010001835	0.965812898	0.344837357
ENSGALG00010001836	0.937403621	0.387642225
ENSGALG00010001837	0.854152913	0.417501817
ENSGALG00010001838	0.668381074	0.272484221
ENSGALG00010001839	0.90167706	0.245486679
ENSGALG00010001840	0.988388444	0.347292261
ENSGALG00010001841	0.373495264	0.061911944
ENSGALG00010001843	0.657803866	0.043446347
ENSGALG00010001844	0.997502673	0.317290205
ENSGALG00010001845	0.08717748	0.048138178
ENSGALG00010001847	0.836220923	0.297835668
ENSGALG00010001848	0.984842721	0.33762884
ENSGALG00010001849	0.847502995	0.34513911
ENSGALG00010001850	0.983403019	0.326427979
ENSGALG00010001852	0.845528298	0.452423048
ENSGALG00010001853	0.975342956	0.28807058
ENSGALG00010001854	0.007468921	0.055443459
ENSGALG00010001856	0.836956887	0.362685347
ENSGALG00010001857	0.968045577	0.260525733
ENSGALG00010001858	0.960473038	0.39879441
ENSGALG00010001860	0.620160965	0.449254878
ENSGALG00010001861	0.304038276	0.266354292
ENSGALG00010001862	0.100071103	0.311755764
ENSGALG00010001863	0.285272007	0.109169955
ENSGALG00010001864	0.661088705	0.014476613
ENSGALG00010001865	0.974707685	0.242396243
ENSGALG00010001866	0.487863067	0.025362473
ENSGALG00010001868	0.961852445	0.300861672
ENSGALG00010001869	0.911395835	0.246146443
ENSGALG00010001870	0.967441795	0.380176804

ENSGALG00010001871	0.100221071	0.412892211
ENSGALG00010001873	0.861763995	0.23517105
ENSGALG00010001874	0.264239718	0.073427965
ENSGALG00010001875	0.164245645	0.108932726
ENSGALG00010001877	0.605460878	0.333927968
ENSGALG00010001878	0.94486034	0.441580577
ENSGALG00010001879	0.921923207	0.394595507
ENSGALG00010001880	0.990681278	0.324144034
ENSGALG00010001882	0.903959733	0.220689775
ENSGALG00010001883	0.878064936	0.277692422
ENSGALG00010001885	0.98458861	0.336543573
ENSGALG00010001886	0.822447073	0.486037751
ENSGALG00010001888	0.750800179	0.220743785
ENSGALG00010001889	0.898150811	0.077434428
ENSGALG00010001890	0.992707465	0.328531774
ENSGALG00010001892	0.993166635	0.285330988
ENSGALG00010001895	0.926931098	0.324491785
ENSGALG00010001896	0.755778039	0.223682771
ENSGALG00010001897	0.917448919	0.348090338
ENSGALG00010001898	0.318961828	0.271158231
ENSGALG00010001899	0.129676067	0.379278408
ENSGALG00010001901	0.986940333	0.305065123
ENSGALG00010001902	0.807440863	0.269679152
ENSGALG00010001903	0.00850065	0.008840407
ENSGALG00010001905	0.474453227	0.175126822
ENSGALG00010001906	0.973321884	0.238608264
ENSGALG00010001907	0.033649171	0.058721699
ENSGALG00010001908	0.561309596	0.161144458
ENSGALG00010001910	0.601743001	0.109459527
ENSGALG00010001911	0.757177548	0.462132434
ENSGALG00010001912	0.965539185	0.311766398
ENSGALG00010001914	0.282061563	0.23673287
ENSGALG00010001915	0.381917902	0.392874494
ENSGALG00010001916	0.200570274	0.091667991
ENSGALG00010001918	0.984103032	0.325585941
ENSGALG00010001921	0.158695787	0.17776753
ENSGALG00010001923	0.087051516	0.293845625
ENSGALG00010001925	0.231437858	0.180950134
ENSGALG00010001928	0.95913538	0.40318912
ENSGALG00010001929	0.51789724	0.195712684
ENSGALG00010001932	0.270451009	0.090541667
ENSGALG00010001933	0.269161007	0.003152763
ENSGALG00010001934	0.656286915	0.222562807
ENSGALG00010001935	0.408724021	0.189326644
ENSGALG00010001936	0.564772984	0.130483595
ENSGALG00010001937	0.447022056	0.214365905
ENSGALG00010001938	0.908835778	0.510353715
ENSGALG00010001940	0.743566916	0.315618966
ENSGALG00010001941	0.583501426	0.242446184
ENSGALG00010001947	0.326544472	0.228845279
ENSGALG00010001948	0.633787179	0.394036387
ENSGALG00010001952	0.937218546	0.374932901
ENSGALG00010001957	0.955927125	0.324326143
ENSGALG00010001958	0.750575146	0.195324776
ENSGALG00010001961	0.794714643	0.313849589
ENSGALG00010001966	0.296387604	0.201163796

ENSGALG00010001967	0.569846666	0.268256926
ENSGALG00010001969	0.886698255	0.304745097
ENSGALG00010001971	0.996073752	0.314944997
ENSGALG00010001972	0.687468578	0.261006717
ENSGALG00010001974	0.595074433	0.242627304
ENSGALG00010001976	0.960203016	0.237115596
ENSGALG00010001982	0.962599659	0.363708127
ENSGALG00010001983	0.420589752	0.19582821
ENSGALG00010001985	0.399453749	0.144668892
ENSGALG00010001987	0.918058442	0.334574514
ENSGALG00010001991	0.978315368	0.322462291
ENSGALG00010001994	0.024265609	0.001469158
ENSGALG00010001995	0.608952549	0.112868405
ENSGALG00010001997	0.874045216	0.468522623
ENSGALG00010001998	0.391612505	0.15490595
ENSGALG00010001999	0.287998705	0.327203319
ENSGALG00010002001	0.466434729	0.173009248
ENSGALG00010002002	0.576113547	0.127580867
ENSGALG00010002003	0.371720349	0.297047526
ENSGALG00010002005	0.64813848	0.139257113
ENSGALG00010002006	0.927659011	0.247504842
ENSGALG00010002011	0.015799617	0.33126237
ENSGALG00010002012	0.119609481	0.128738958
ENSGALG00010002013	0.98891557	0.347238662
ENSGALG00010002015	0.977479543	0.30286779
ENSGALG00010002016	0.256828349	0.119064955
ENSGALG00010002017	0.787248049	0.229353503
ENSGALG00010002019	0.179605246	0.330783199
ENSGALG00010002022	0.001482199	0.141923802
ENSGALG00010002023	0.961856308	0.30679047
ENSGALG00010002024	0.984007534	0.325094127
ENSGALG00010002027	0.988449513	0.339751757
ENSGALG00010002031	0.123809512	0.225781273
ENSGALG00010002032	0.266137114	0.107784983
ENSGALG00010002033	0.750300665	0.372604994
ENSGALG00010002034	0.601758652	0.109944628
ENSGALG00010002035	0.502118462	0.332717279
ENSGALG00010002036	0.639658693	0.412252286
ENSGALG00010002039	0.920364582	0.366939472
ENSGALG00010002040	0.262922434	0.138601464
ENSGALG00010002041	0.990963849	0.353009736
ENSGALG00010002043	0.262922434	0.138601464
ENSGALG00010002045	0.596787529	0.130584084
ENSGALG00010002046	0.958424938	0.244257041
ENSGALG00010002053	0.270451009	0.090541667
ENSGALG00010002054	0.870869653	0.336132441
ENSGALG00010002056	0.195381024	0.080851447
ENSGALG00010002058	0.36047204	0.134977804
ENSGALG00010002060	0.920184343	0.294891757
ENSGALG00010002062	0.924010136	0.362654966
ENSGALG00010002063	0.193734992	0.177459015
ENSGALG00010002065	0.41048686	0.009984217
ENSGALG00010002067	0.364871524	0.123582396
ENSGALG00010002068	0.235630588	0.132393411
ENSGALG00010002072	0.911476469	0.262402167
ENSGALG00010002077	0.286555462	0.078819245

ENSGALG00010002079	0.998279143	0.336234355
ENSGALG00010002084	0.974199586	0.32731906
ENSGALG00010002087	0.178616917	0.180857114
ENSGALG00010002088	0.535201006	0.247188657
ENSGALG00010002090	0.903262855	0.425614721
ENSGALG00010002091	0.285272007	0.109169955
ENSGALG00010002092	0.034019991	0.100937458
ENSGALG00010002096	0.349930392	0.50024929
ENSGALG00010002097	0.688904084	0.548530308
ENSGALG00010002098	0.833964872	0.261780622
ENSGALG00010002100	0.977459802	0.311983929
ENSGALG00010002101	0.155022075	0.203417013
ENSGALG00010002102	0.855267399	0.291276986
ENSGALG00010002105	0.317817399	0.302086383
ENSGALG00010002106	0.26027575	0.046300471
ENSGALG00010002107	0.820836269	0.334211338
ENSGALG00010002111	0.558566182	0.190569919
ENSGALG00010002112	0.807907065	0.266517914
ENSGALG00010002115	0.412926743	0.131655581
ENSGALG00010002116	0.66841915	0.277631832
ENSGALG00010002117	0.221294806	0.149665226
ENSGALG00010002118	0.79492547	0.343593845
ENSGALG00010002122	0.312470885	0.570353048
ENSGALG00010002126	0.963133114	0.357270411
ENSGALG00010002129	0.399426914	0.178230835
ENSGALG00010002131	0.250946664	0.19344305
ENSGALG00010002134	0.924202636	0.225355732
ENSGALG00010002135	0.980372099	0.280412341
ENSGALG00010002136	0.017490827	0.103523428
ENSGALG00010002137	0.920415746	0.26491614
ENSGALG00010002138	0.935809807	0.197887315
ENSGALG00010002139	0.387818129	0.081671798
ENSGALG00010002140	0.300082404	0.333976218
ENSGALG00010002142	0.840976609	0.291194717
ENSGALG00010002143	0.417426328	0.086497625
ENSGALG00010002145	0.379083402	0.207745045
ENSGALG00010002146	0.937506555	0.234066966
ENSGALG00010002148	0.963240937	0.294170194
ENSGALG00010002149	0.841892451	0.368264097
ENSGALG00010002150	0.974137068	0.299355199
ENSGALG00010002153	0.331971497	0.045379539
ENSGALG00010002154	0.448057352	0.358463963
ENSGALG00010002155	0.32196029	0.050029141
ENSGALG00010002156	0.893453353	0.200582769
ENSGALG00010002157	0.243306597	0.037543053
ENSGALG00010002158	0.910665853	0.353970553
ENSGALG00010002159	0.913825897	0.296802372
ENSGALG00010002160	0.796113835	0.268415382
ENSGALG00010002161	0.552718574	0.177327382
ENSGALG00010002162	0.487268559	0.180261519
ENSGALG00010002163	0.050617734	0.096533567
ENSGALG00010002165	0.815355117	0.389309432
ENSGALG00010002166	0.004293511	0.153198801
ENSGALG00010002167	0.265558089	0.028821141
ENSGALG00010002171	0.284475392	0.383854323
ENSGALG00010002173	0.35061144	0.226205545

ENSGALG00010002174	0.631184483	0.291887293
ENSGALG00010002175	0.036009037	0.278514501
ENSGALG00010002176	0.663505605	0.376624786
ENSGALG00010002177	0.83957327	0.415685877
ENSGALG00010002179	0.11121658	0.144637997
ENSGALG00010002181	0.594785611	0.158473488
ENSGALG00010002185	0.206080138	0.254112363
ENSGALG00010002186	0.965078447	0.266865386
ENSGALG00010002187	0.684292802	0.275619159
ENSGALG00010002188	0.832477341	0.28742483
ENSGALG00010002191	0.131862928	0.144483832
ENSGALG00010002192	0.977970476	0.389163571
ENSGALG00010002193	0.013536418	0.091863354
ENSGALG00010002194	0.976054342	0.275593113
ENSGALG00010002196	0.746921051	0.010823814
ENSGALG00010002197	0.967678454	0.320388359
ENSGALG00010002199	0.40785333	0.157127562
ENSGALG00010002200	0.960697932	0.271426871
ENSGALG00010002202	0.897395274	0.187043309
ENSGALG00010002203	0.235630588	0.132393411
ENSGALG00010002204	0.359249748	0.523045056
ENSGALG00010002205	0.378848707	0.137590461
ENSGALG00010002206	0.915008195	0.154977911
ENSGALG00010002207	0.522750984	0.288998508
ENSGALG00010002208	0.956191986	0.319042847
ENSGALG00010002211	0.975853164	0.313823341
ENSGALG00010002213	0.264239718	0.073427965
ENSGALG00010002215	0.05248968	0.311555691
ENSGALG00010002219	0.332441466	0.000632524
ENSGALG00010002220	0.392749996	0.129686126
ENSGALG00010002225	0.972774838	0.262065753
ENSGALG00010002226	0.545906426	0.115022971
ENSGALG00010002227	0.372048872	0.145150327
ENSGALG00010002229	0.334462638	0.171008572
ENSGALG00010002231	0.968389065	0.296951638
ENSGALG00010002232	0.201512115	0.188537209
ENSGALG00010002235	0.966381803	0.360506061
ENSGALG00010002237	0.871457344	0.266855614
ENSGALG00010002238	0.234648381	0.205315189
ENSGALG00010002239	0.988688185	0.335082269
ENSGALG00010002242	0.729204817	0.22567151
ENSGALG00010002243	0.61833817	0.140594786
ENSGALG00010002247	0.963281966	0.282022851
ENSGALG00010002249	0.064441818	0.209223326
ENSGALG00010002250	0.490643133	0.120773411
ENSGALG00010002251	0.968472082	0.195980758
ENSGALG00010002252	0.308758304	0.100827364
ENSGALG00010002253	0.981863495	0.315641213
ENSGALG00010002254	0.929203839	0.326197059
ENSGALG00010002255	0.404167701	0.144831306
ENSGALG00010002257	0.401639624	0.101384476
ENSGALG00010002259	0.841979688	0.115649868
ENSGALG00010002260	0.829235643	0.461410663
ENSGALG00010002261	0.981291432	0.358477627
ENSGALG00010002263	0.083875835	0.089201779
ENSGALG00010002269	0.482041472	0.068104602

ENSGALG00010002270	0.432089303	0.112386283
ENSGALG00010002271	0.953948707	0.366179693
ENSGALG00010002272	0.013536418	0.091863354
ENSGALG00010002274	0.781782082	0.150063433
ENSGALG00010002275	0.460756806	0.123465737
ENSGALG00010002277	0.771558499	0.126067366
ENSGALG00010002278	0.791631678	0.347553326
ENSGALG00010002280	0.854528827	0.240573047
ENSGALG00010002281	0.523785584	0.37428227
ENSGALG00010002282	0.878326268	0.273846912
ENSGALG00010002284	0.207029721	0.157390351
ENSGALG00010002285	0.981283397	0.376850882
ENSGALG00010002286	0.410453886	0.411345105
ENSGALG00010002288	0.881235023	0.410091685
ENSGALG00010002290	0.922039554	0.428214604
ENSGALG00010002291	0.79817682	0.220025111
ENSGALG00010002292	0.266137114	0.107784983
ENSGALG00010002293	0.20322794	0.04483825
ENSGALG00010002294	0.410372274	0.054570367
ENSGALG00010002295	0.420623441	0.236251635
ENSGALG00010002297	0.329906659	0.052658091
ENSGALG00010002298	0.319246163	0.029179991
ENSGALG00010002299	0.470913161	0.146489572
ENSGALG00010002303	0.269007545	0.141697266
ENSGALG00010002304	0.55470103	0.21617295
ENSGALG00010002307	0.089145506	0.104599052
ENSGALG00010002308	0.81350012	0.200696867
ENSGALG00010002309	0.984130856	0.363286487
ENSGALG00010002310	0.82405586	0.314913743
ENSGALG00010002311	0.068507877	0.183989296
ENSGALG00010002312	0.814999364	0.413627874
ENSGALG00010002314	0.265558089	0.028821141
ENSGALG00010002315	0.419325205	0.386495332
ENSGALG00010002318	0.195642099	0.201239282
ENSGALG00010002322	0.404293546	0.305645222
ENSGALG00010002324	0.007420952	0.120965845
ENSGALG00010002328	0.808165789	0.126141729
ENSGALG00010002329	0.376641041	0.343763671
ENSGALG00010002330	0.648195409	0.41241721
ENSGALG00010002331	0.116533458	0.349735986
ENSGALG00010002332	0.405979644	0.432394487
ENSGALG00010002333	0.83870193	0.284339411
ENSGALG00010002334	0.870389854	0.185179678
ENSGALG00010002335	0.987730943	0.400027166
ENSGALG00010002336	0.312883485	0.180249285
ENSGALG00010002337	0.037470672	0.184159142
ENSGALG00010002338	0.982019403	0.408089306
ENSGALG00010002339	0.546813749	0.329813676
ENSGALG00010002340	0.464885443	0.178568748
ENSGALG00010002341	0.970848915	0.283811486
ENSGALG00010002342	0.992994184	0.314984031
ENSGALG00010002343	0.946602163	0.23496604
ENSGALG00010002344	0.271701261	0.072417625
ENSGALG00010002345	0.6568966	0.196937671
ENSGALG00010002346	0.497430348	0.269146535
ENSGALG00010002347	0.881412428	0.433580416

ENSGALG00010002348	0.54918003	0.340428897
ENSGALG00010002349	0.66265891	0.076203495
ENSGALG00010002350	0.007673762	0.024982282
ENSGALG00010002351	0.236432957	0.223877779
ENSGALG00010002352	0.622179368	0.264594319
ENSGALG00010002353	0.526467769	0.288638189
ENSGALG00010002354	0.20150931	0.117569843
ENSGALG00010002355	0.935521746	0.251158205
ENSGALG00010002356	0.757756127	0.2999707
ENSGALG00010002357	0.937962693	0.260327998
ENSGALG00010002358	0.733257125	0.26569355
ENSGALG00010002359	0.915087417	0.276591313
ENSGALG00010002360	0.845872958	0.37995274
ENSGALG00010002361	0.939857001	0.369748718
ENSGALG00010002362	0.146806367	0.489290217
ENSGALG00010002364	0.989998402	0.38272385
ENSGALG00010002365	0.987267893	0.35276476
ENSGALG00010002366	0.806419697	0.092386917
ENSGALG00010002368	0.922752508	0.365251738
ENSGALG00010002369	0.72116223	0.081001222
ENSGALG00010002370	0.029378696	0.074475345
ENSGALG00010002371	0.994751126	0.319619551
ENSGALG00010002372	0.37567732	0.265265869
ENSGALG00010002373	0.289500909	0.136137638
ENSGALG00010002374	0.979462808	0.324138768
ENSGALG00010002375	0.541434491	0.784661634
ENSGALG00010002376	0.878989566	0.335994526
ENSGALG00010002377	0.921964238	0.394114084
ENSGALG00010002378	0.629554137	0.009240489
ENSGALG00010002379	0.00111261	0.287462778
ENSGALG00010002380	0.94048042	0.444354167
ENSGALG00010002381	0.962015934	0.239305225
ENSGALG00010002382	0.435769736	0.009700538
ENSGALG00010002383	0.682525544	0.455637758
ENSGALG00010002384	0.351344784	0.143255986
ENSGALG00010002385	0.962191819	0.295832921
ENSGALG00010002386	0.258099818	0.199697
ENSGALG00010002387	0.849461369	0.082731173
ENSGALG00010002388	0.903419374	0.157467723
ENSGALG00010002389	0.139907236	0.0013802
ENSGALG00010002391	0.852551265	0.351981951
ENSGALG00010002392	0.950756384	0.409657786
ENSGALG00010002393	0.986132545	0.3319394
ENSGALG00010002395	0.984517438	0.329817898
ENSGALG00010002396	0.101370942	0.065971534
ENSGALG00010002397	0.556270843	0.259490072
ENSGALG00010002398	0.075277146	0.41885664
ENSGALG00010002399	0.279300361	0.104446866
ENSGALG00010002400	0.327339808	0.258875108
ENSGALG00010002401	0.873381245	0.134993275
ENSGALG00010002402	0.165747177	0.147615287
ENSGALG00010002403	0.817550119	0.138408616
ENSGALG00010002404	0.927240254	0.231391258
ENSGALG00010002405	0.818190503	0.431149568
ENSGALG00010002406	0.330385536	0.265643889
ENSGALG00010002407	0.109582889	0.331216082

ENSGALG00010002408	0.399639503	0.273436609
ENSGALG00010002409	0.858670336	0.348960999
ENSGALG00010002410	0.87470675	0.405178391
ENSGALG00010002411	0.544494716	0.016119427
ENSGALG00010002413	0.939530492	0.241914034
ENSGALG00010002414	0.347516663	0.024277913
ENSGALG00010002416	0.966197547	0.380983443
ENSGALG00010002417	0.268039933	0.265604527
ENSGALG00010002418	0.553929708	0.045602183
ENSGALG00010002419	0.94792226	0.386722065
ENSGALG00010002420	0.271701261	0.072417625
ENSGALG00010002421	0.377217408	0.077348101
ENSGALG00010002422	0.673520653	0.258768313
ENSGALG00010002423	0.26221985	0.169429033
ENSGALG00010002424	0.944718644	0.32217825
ENSGALG00010002425	0.975269595	0.376700932
ENSGALG00010002426	0.048532643	0.007975711
ENSGALG00010002427	0.568091387	0.301122608
ENSGALG00010002428	0.800928272	0.341502302
ENSGALG00010002429	0.562464518	0.203919215
ENSGALG00010002430	0.984187021	0.303509453
ENSGALG00010002431	0.894497211	0.20551621
ENSGALG00010002432	0.379870409	0.007626282
ENSGALG00010002433	0.854188547	0.276192734
ENSGALG00010002434	0.977066946	0.295676463
ENSGALG00010002435	0.993944509	0.340708021
ENSGALG00010002436	0.22972462	0.267744745
ENSGALG00010002437	0.273637011	0.154650996
ENSGALG00010002438	0.569636624	0.091692577
ENSGALG00010002439	0.849959777	0.198065964
ENSGALG00010002440	0.951119588	0.308529672
ENSGALG00010002441	0.244131471	0.249991568
ENSGALG00010002442	0.296802117	0.049266998
ENSGALG00010002443	0.746706235	0.185213419
ENSGALG00010002444	0.726320069	0.078836282
ENSGALG00010002445	0.955524188	0.372202695
ENSGALG00010002446	0.638384901	0.001965816
ENSGALG00010002447	0.765710835	0.012769636
ENSGALG00010002448	0.346743652	0.121805224
ENSGALG00010002449	0.87390924	0.174633544
ENSGALG00010002450	0.001253957	0.226044501
ENSGALG00010002451	0.211604673	0.150221027
ENSGALG00010002453	0.441743725	0.521469948
ENSGALG00010002454	0.86642296	0.409841965
ENSGALG00010002455	0.796606791	0.141745318
ENSGALG00010002456	0.388899421	0.120062276
ENSGALG00010002457	0.093327267	0.035470014
ENSGALG00010002458	0.986168127	0.288264235
ENSGALG00010002459	0.513826869	0.146371947
ENSGALG00010002460	0.990649136	0.336642969
ENSGALG00010002461	0.967977958	0.328576842
ENSGALG00010002462	0.804243719	0.169200784
ENSGALG00010002463	0.519849624	0.088950182
ENSGALG00010002464	0.525941386	0.092177103
ENSGALG00010002465	0.971630941	0.344893196
ENSGALG00010002466	0.971232383	0.282005213

ENSGALG00010002467	0.826683508	0.163189772
ENSGALG00010002468	0.109265555	0.056672294
ENSGALG00010002470	0.761699184	0.312249899
ENSGALG00010002471	0.127500065	0.038712491
ENSGALG00010002472	0.980961522	0.305522222
ENSGALG00010002473	0.321159034	0.382612349
ENSGALG00010002474	0.926733333	0.300303624
ENSGALG00010002475	0.746926439	0.074301467
ENSGALG00010002476	0.396723331	0.068539048
ENSGALG00010002478	0.215333569	0.131764311
ENSGALG00010002481	0.243549737	0.139892922
ENSGALG00010002482	0.360647091	0.259347684
ENSGALG00010002483	0.886229716	0.305511434
ENSGALG00010002484	0.924855759	0.40111544
ENSGALG00010002485	0.944885358	0.389285791
ENSGALG00010002487	0.812958172	0.103583696
ENSGALG00010002491	0.301310019	0.334117489
ENSGALG00010002492	0.901679399	0.306371713
ENSGALG00010002493	0.587695198	0.121793753
ENSGALG00010002494	0.013254443	0.189524565
ENSGALG00010002495	0.373446581	0.117589412
ENSGALG00010002496	0.505026024	0.219416157
ENSGALG00010002497	0.452161949	0.056599887
ENSGALG00010002498	0.264239718	0.073427965
ENSGALG00010002500	0.837498197	0.397635092
ENSGALG00010002501	0.941076251	0.233095693
ENSGALG00010002503	0.525432077	0.328586149
ENSGALG00010002504	0.947816851	0.272717015
ENSGALG00010002507	0.514385928	0.068384281
ENSGALG00010002511	0.507605255	0.000207829
ENSGALG00010002512	0.954727106	0.28627579
ENSGALG00010002513	0.987629156	0.385431264
ENSGALG00010002518	0.817655668	0.416578256
ENSGALG00010002520	0.7256483	0.102078649
ENSGALG00010002522	0.943366769	0.394613358
ENSGALG00010002523	0.983262658	0.348233078
ENSGALG00010002525	0.623949504	0.305339076
ENSGALG00010002526	0.976744457	0.331648369
ENSGALG00010002527	0.906565876	0.331794818
ENSGALG00010002528	0.910976106	0.301691782
ENSGALG00010002529	0.438779362	0.196437292
ENSGALG00010002530	0.610628981	0.303084537
ENSGALG00010002531	0.382713001	0.71925729
ENSGALG00010002532	0.292362493	0.105019933
ENSGALG00010002533	0.402134739	0.158601814
ENSGALG00010002535	0.216433634	0.680598293
ENSGALG00010002536	0.785873165	0.213352519
ENSGALG00010002537	0.290365752	0.580915876
ENSGALG00010002539	0.82283447	0.225386718
ENSGALG00010002540	0.586480106	0.2100934
ENSGALG00010002541	0.843507467	0.144176543
ENSGALG00010002543	0.982549855	0.343026867
ENSGALG00010002544	0.271311783	0.10889103
ENSGALG00010002546	0.96515111	0.316880546
ENSGALG00010002547	0.761726917	0.037184173
ENSGALG00010002550	0.409801916	0.308826204

ENSGALG00010002551	0.559085956	0.500134161
ENSGALG00010002553	0.607736483	0.516102329
ENSGALG00010002554	0.978666747	0.376230245
ENSGALG00010002556	0.883766133	0.36095683
ENSGALG00010002557	0.4854601	0.302175003
ENSGALG00010002559	0.471333085	0.16661648
ENSGALG00010002561	0.810365224	0.249143162
ENSGALG00010002563	0.713249995	0.125533509
ENSGALG00010002565	0.952554929	0.220290927
ENSGALG00010002566	0.864815577	0.185604576
ENSGALG00010002567	0.24148808	0.516815743
ENSGALG00010002569	0.053398513	0.653005504
ENSGALG00010002570	0.92074244	0.227887537
ENSGALG00010002571	0.745830978	0.211749186
ENSGALG00010002572	0.591517896	0.522424853
ENSGALG00010002573	0.816745632	0.142673975
ENSGALG00010002575	0.785573964	0.311367318
ENSGALG00010002576	0.348083767	0.433107477
ENSGALG00010002577	0.134335152	0.013225701
ENSGALG00010002578	0.426258376	0.053454122
ENSGALG00010002579	0.622043971	0.195421409
ENSGALG00010002580	0.052478779	0.171591946
ENSGALG00010002581	0.06666648	0.306647492
ENSGALG00010002582	0.99660158	0.34273536
ENSGALG00010002584	0.687510332	0.437407259
ENSGALG00010002585	0.593073102	0.287060952
ENSGALG00010002586	0.775283986	0.294009529
ENSGALG00010002587	0.842438936	0.08544142
ENSGALG00010002588	0.864803051	0.253398894
ENSGALG00010002589	0.348658701	0.351293902
ENSGALG00010002590	0.834407264	0.045623602
ENSGALG00010002591	0.770610577	0.117572362
ENSGALG00010002592	0.308514388	0.237056647
ENSGALG00010002595	0.264239718	0.073427965
ENSGALG00010002596	0.370777081	0.136486549
ENSGALG00010002597	0.761883842	0.226463848
ENSGALG00010002598	0.849081232	0.325485809
ENSGALG00010002599	0.132526512	0.141072167
ENSGALG00010002600	0.786218138	0.29346218
ENSGALG00010002601	0.987507296	0.339380013
ENSGALG00010002603	0.592872182	0.000555661
ENSGALG00010002604	0.468156893	0.024688016
ENSGALG00010002605	0.897861522	0.142743458
ENSGALG00010002606	0.591244658	0.256270192
ENSGALG00010002608	0.168760106	0.013448821
ENSGALG00010002609	0.938421906	0.261421442
ENSGALG00010002611	0.252875981	0.081393995
ENSGALG00010002612	0.994398855	0.316285402
ENSGALG00010002613	0.832557548	0.42056262
ENSGALG00010002614	0.226604935	0.047815008
ENSGALG00010002616	0.600821256	0.129690324
ENSGALG00010002617	0.834801906	0.378750325
ENSGALG00010002618	0.766394885	0.189504672
ENSGALG00010002619	0.832297565	0.19961566
ENSGALG00010002621	0.832799435	0.310171368
ENSGALG00010002622	0.860134182	0.411581648

ENSGALG00010002623	0.988168784	0.34738914
ENSGALG00010002624	0.798170236	0.127850966
ENSGALG00010002625	0.867022457	0.407691782
ENSGALG00010002626	0.021573164	0.027978749
ENSGALG00010002627	0.969006675	0.343020729
ENSGALG00010002628	0.961988424	0.365979351
ENSGALG00010002629	0.976540787	0.383142424
ENSGALG00010002630	0.200570274	0.091667991
ENSGALG00010002631	0.396365709	0.157137627
ENSGALG00010002632	0.975789749	0.300951433
ENSGALG00010002633	0.914201219	0.391265466
ENSGALG00010002634	0.94412954	0.40166593
ENSGALG00010002635	0.584985124	0.125300598
ENSGALG00010002636	0.784076848	0.286908227
ENSGALG00010002637	0.128456369	0.294809833
ENSGALG00010002638	0.09567427	0.090373318
ENSGALG00010002640	0.208550923	0.676301294
ENSGALG00010002641	0.367503232	0.093310735
ENSGALG00010002643	0.38913966	0.311292364
ENSGALG00010002644	0.734858781	0.049428576
ENSGALG00010002646	0.542147947	0.023183849
ENSGALG00010002647	0.981785971	0.272291201
ENSGALG00010002649	0.662011727	0.25769865
ENSGALG00010002650	0.1051832	0.08542629
ENSGALG00010002651	0.058197908	0.203222062
ENSGALG00010002652	0.251163168	0.06689371
ENSGALG00010002653	0.940120982	0.115733884
ENSGALG00010002654	0.27428151	0.347212613
ENSGALG00010002655	0.948305061	0.338631972
ENSGALG00010002656	0.210161188	0.078991138
ENSGALG00010002657	0.235630588	0.132393411
ENSGALG00010002658	0.97805512	0.333123565
ENSGALG00010002659	0.40135901	0.158038591
ENSGALG00010002661	0.892018324	0.401479067
ENSGALG00010002662	0.188404905	0.00721247
ENSGALG00010002663	0.158426212	0.147582862
ENSGALG00010002664	0.908910103	0.151799814
ENSGALG00010002666	0.844113614	0.262859143
ENSGALG00010002667	0.995221774	0.335999421
ENSGALG00010002668	0.018485516	0.005043303
ENSGALG00010002669	0.899584903	0.246510372
ENSGALG00010002671	0.917528577	0.16190009
ENSGALG00010002673	0.948304103	0.432005085
ENSGALG00010002676	0.503222906	0.103611778
ENSGALG00010002677	0.515294759	0.296742286
ENSGALG00010002678	0.984389628	0.321496412
ENSGALG00010002679	0.711348852	0.238315284
ENSGALG00010002680	0.689010494	0.046739699
ENSGALG00010002681	0.645875683	0.181812107
ENSGALG00010002682	0.935281818	0.284039443
ENSGALG00010002685	0.895649539	0.304949716
ENSGALG00010002688	0.385889159	0.436525088
ENSGALG00010002689	0.909515111	0.387601969
ENSGALG00010002693	0.927987726	0.367371735
ENSGALG00010002694	0.089670069	0.175722502
ENSGALG00010002695	0.693152292	0.192497857

ENSGALG00010002700	0.907690254	0.401399344
ENSGALG00010002701	0.013536418	0.091863354
ENSGALG00010002702	0.514763036	0.546902705
ENSGALG00010002703	0.97936662	0.284018576
ENSGALG00010002704	0.988398549	0.371435111
ENSGALG00010002705	0.969325867	0.398920039
ENSGALG00010002706	0.978999387	0.301042744
ENSGALG00010002707	0.512479956	0.002186191
ENSGALG00010002708	0.698499931	0.563935604
ENSGALG00010002709	0.616577629	0.266600516
ENSGALG00010002711	0.665471944	0.209041478
ENSGALG00010002712	0.941779316	0.424316691
ENSGALG00010002713	0.980798879	0.29252487
ENSGALG00010002714	0.942342294	0.277452427
ENSGALG00010002716	0.917246188	0.245395192
ENSGALG00010002718	0.806663257	0.25075505
ENSGALG00010002722	0.942800182	0.318929477
ENSGALG00010002723	0.081960713	0.054317503
ENSGALG00010002724	0.265558089	0.028821141
ENSGALG00010002725	0.961884516	0.315741108
ENSGALG00010002727	0.405710703	0.256827649
ENSGALG00010002728	0.963633881	0.266322551
ENSGALG00010002729	0.050115024	0.155140157
ENSGALG00010002730	0.961373189	0.224244191
ENSGALG00010002732	0.061812619	0.144281832
ENSGALG00010002733	0.664116795	0.064144743
ENSGALG00010002737	0.384831073	0.205747021
ENSGALG00010002738	0.553196658	0.142295553
ENSGALG00010002739	0.284227058	0.103920777
ENSGALG00010002740	0.597098814	0.038813846
ENSGALG00010002741	0.188503634	0.036622052
ENSGALG00010002742	0.079922276	0.124331003
ENSGALG00010002743	0.321362185	0.385493124
ENSGALG00010002746	0.097253698	0.391639402
ENSGALG00010002747	0.218604483	0.59786529
ENSGALG00010002750	0.817627166	0.130611257
ENSGALG00010002752	0.972027803	0.294590017
ENSGALG00010002753	0.79032313	0.624994439
ENSGALG00010002754	0.423548628	0.274867707
ENSGALG00010002756	0.979643725	0.333733693
ENSGALG00010002758	0.489921907	0.173810043
ENSGALG00010002760	0.908423175	0.156626554
ENSGALG00010002762	0.646585239	0.354940791
ENSGALG00010002763	0.985776839	0.319294611
ENSGALG00010002765	0.987072451	0.365734253
ENSGALG00010002766	0.914860215	0.2082568
ENSGALG00010002767	0.958757267	0.399259527
ENSGALG00010002768	0.787171397	0.538873536
ENSGALG00010002774	0.990232382	0.354145301
ENSGALG00010002775	0.129381518	0.340333232
ENSGALG00010002777	0.226728902	0.299204319
ENSGALG00010002778	0.015410241	0.296645041
ENSGALG00010002779	0.974641964	0.380419443
ENSGALG00010002781	0.935081051	0.293169562
ENSGALG00010002782	0.905563743	0.28963167
ENSGALG00010002783	0.547459666	0.147704667

ENSGALG00010002784	0.817501527	0.309279627
ENSGALG00010002785	0.445272576	0.129016816
ENSGALG00010002786	0.497040156	0.153304647
ENSGALG00010002787	0.808403104	0.383330073
ENSGALG00010002788	0.989858915	0.313439297
ENSGALG00010002789	0.536497008	0.270657586
ENSGALG00010002790	0.717602907	0.039521565
ENSGALG00010002792	0.924073034	0.337488594
ENSGALG00010002795	0.813272739	0.382047568
ENSGALG00010002796	0.64519392	0.342000698
ENSGALG00010002798	0.738267709	0.11789362
ENSGALG00010002800	0.672318269	0.371935094
ENSGALG00010002801	0.123399186	0.153791139
ENSGALG00010002803	0.160453813	0.049889016
ENSGALG00010002805	0.781338966	0.259011695
ENSGALG00010002807	0.468137186	0.074174055
ENSGALG00010002808	0.679381855	0.141389924
ENSGALG00010002809	0.651000185	0.177137896
ENSGALG00010002810	0.273136127	0.096156459
ENSGALG00010002812	0.077726789	0.139500592
ENSGALG00010002814	0.807851342	0.471808017
ENSGALG00010002816	0.3740819	0.203837355
ENSGALG00010002817	0.977707071	0.302488857
ENSGALG00010002819	0.637887274	0.096111047
ENSGALG00010002821	0.948606912	0.333173773
ENSGALG00010002822	0.559695398	0.225424885
ENSGALG00010002825	0.955981058	0.428515922
ENSGALG00010002827	0.802380691	0.238740324
ENSGALG00010002830	0.737181229	0.237131313
ENSGALG00010002832	0.089586703	0.079373167
ENSGALG00010002833	0.532342495	0.19563974
ENSGALG00010002834	0.617521292	0.118097216
ENSGALG00010002835	0.974440928	0.303915591
ENSGALG00010002837	0.887372354	0.245444565
ENSGALG00010002838	0.966231797	0.363958387
ENSGALG00010002840	0.921029481	0.2903661
ENSGALG00010002841	0.034342762	0.247942561
ENSGALG00010002842	0.368847025	0.001815083
ENSGALG00010002844	0.18653258	0.177497954
ENSGALG00010002846	0.067693333	0.482957797
ENSGALG00010002847	0.018301635	0.519548151
ENSGALG00010002848	0.963487809	0.327363036
ENSGALG00010002849	0.215564187	0.055310278
ENSGALG00010002850	0.484546415	0.13932145
ENSGALG00010002852	0.363332073	0.116301156
ENSGALG00010002853	0.299914809	0.159203077
ENSGALG00010002855	0.934951684	0.313402512
ENSGALG00010002856	0.583360324	0.010464357
ENSGALG00010002857	0.907740721	0.121127844
ENSGALG00010002858	0.090929485	0.127903745
ENSGALG00010002860	0.909438875	0.382931244
ENSGALG00010002862	0.034784375	0.127082974
ENSGALG00010002863	0.634105514	0.067200761
ENSGALG00010002864	0.543976329	0.101285266
ENSGALG00010002865	0.319320077	0.024051854
ENSGALG00010002866	0.949012625	0.342950128

ENSGALG00010002867	0.336495107	0.02005805
ENSGALG00010002868	0.154749196	0.053544849
ENSGALG00010002870	0.988771347	0.372066371
ENSGALG00010002871	0.972456741	0.281995985
ENSGALG00010002872	0.02545792	0.169929005
ENSGALG00010002874	0.321350164	0.307712971
ENSGALG00010002875	0.599752179	0.216695548
ENSGALG00010002876	0.399453749	0.144668892
ENSGALG00010002878	0.990041938	0.34967244
ENSGALG00010002879	0.08290626	0.315697545
ENSGALG00010002880	0.713284886	0.276050439
ENSGALG00010002881	0.984528091	0.333212714
ENSGALG00010002882	0.328403153	0.064477768
ENSGALG00010002883	0.865969641	0.20661609
ENSGALG00010002884	0.944603563	0.313006779
ENSGALG00010002885	0.776917659	0.289278976
ENSGALG00010002886	0.962354179	0.331749342
ENSGALG00010002888	0.872640908	0.387452838
ENSGALG00010002891	0.488583769	0.130802663
ENSGALG00010002894	0.780387628	0.097965825
ENSGALG00010002895	0.541729153	0.26832461
ENSGALG00010002896	0.299137823	0.17686552
ENSGALG00010002897	0.931414189	0.421330955
ENSGALG00010002898	0.459182356	0.057010776
ENSGALG00010002899	0.54500619	0.237306784
ENSGALG00010002900	0.215989685	0.133491664
ENSGALG00010002902	0.417686966	0.030069739
ENSGALG00010002903	0.374329203	0.223226042
ENSGALG00010002904	0.867695087	0.307694231
ENSGALG00010002906	0.535734581	0.256996225
ENSGALG00010002907	0.036463635	0.400420927
ENSGALG00010002908	0.951212916	0.322436714
ENSGALG00010002909	0.867748891	0.284006757
ENSGALG00010002910	0.007866985	0.178290013
ENSGALG00010002911	0.707555847	0.029984673
ENSGALG00010002912	0.609874666	0.305357225
ENSGALG00010002913	0.9147753	0.287018165
ENSGALG00010002914	0.961857327	0.303227608
ENSGALG00010002915	0.561986621	0.201759229
ENSGALG00010002916	0.937706168	0.294043772
ENSGALG00010002917	0.873729623	0.274242787
ENSGALG00010002918	0.964797923	0.249936301
ENSGALG00010002919	0.921404978	0.23887909
ENSGALG00010002920	0.713222425	0.199940403
ENSGALG00010002921	0.942949372	0.441491969
ENSGALG00010002922	0.977456033	0.293173964
ENSGALG00010002923	0.757976084	0.366250395
ENSGALG00010002924	0.534017986	0.220747753
ENSGALG00010002925	0.959037204	0.421181984
ENSGALG00010002926	0.254111924	0.543691091
ENSGALG00010002927	0.302352116	0.144221977
ENSGALG00010002928	0.990082963	0.332064354
ENSGALG00010002929	0.782079651	0.264437072
ENSGALG00010002930	0.094420733	0.114438224
ENSGALG00010002931	0.400778541	0.133797302
ENSGALG00010002934	0.264239718	0.073427965

ENSGALG00010002935	0.753183599	0.250138541
ENSGALG00010002937	0.832117103	0.118128938
ENSGALG00010002939	0.319804591	0.225079114
ENSGALG00010002940	0.898236788	0.370087183
ENSGALG00010002941	0.052836869	0.101827534
ENSGALG00010002942	0.321208885	0.180627958
ENSGALG00010002943	0.953350271	0.269412889
ENSGALG00010002946	0.949813977	0.31152286
ENSGALG00010002947	0.159309019	0.102716807
ENSGALG00010002949	0.0093646	0.159193652
ENSGALG00010002950	0.819412691	0.123162726
ENSGALG00010002951	0.350516363	0.140397923
ENSGALG00010002952	0.671851489	0.007396482
ENSGALG00010002953	0.996900958	0.340224508
ENSGALG00010002954	0.545711454	0.129888083
ENSGALG00010002955	0.936569118	0.255610602
ENSGALG00010002956	0.596206285	0.315116623
ENSGALG00010002957	0.71589222	0.112283395
ENSGALG00010002958	0.729192722	0.516375274
ENSGALG00010002960	0.358360697	0.034853537
ENSGALG00010002962	0.895310695	0.170926509
ENSGALG00010002963	0.21177891	0.172575124
ENSGALG00010002964	0.484974795	0.209787014
ENSGALG00010002965	0.967378968	0.398331504
ENSGALG00010002966	0.451442752	0.192821405
ENSGALG00010002967	0.745841062	0.087621869
ENSGALG00010002968	0.399665645	0.210283168
ENSGALG00010002969	0.724204835	0.243191962
ENSGALG00010002970	0.255071857	0.134385033
ENSGALG00010002972	0.860730098	0.22872842
ENSGALG00010002978	0.92335593	0.305091371
ENSGALG00010002979	0.185245082	0.005210771
ENSGALG00010002981	0.160561999	0.124937733
ENSGALG00010002982	0.864825664	0.259042923
ENSGALG00010002983	0.945615056	0.250106254
ENSGALG00010002984	0.532832538	0.603240541
ENSGALG00010002985	0.323600332	0.046971225
ENSGALG00010002986	0.26745999	0.014614436
ENSGALG00010002987	0.598501561	0.054000852
ENSGALG00010002988	0.037762284	0.090313054
ENSGALG00010002991	0.456794173	0.004444862
ENSGALG00010002992	0.922965896	0.167925537
ENSGALG00010002994	0.792162307	0.340634752
ENSGALG00010002995	0.891087512	0.159067386
ENSGALG00010002998	0.537793187	0.161957436
ENSGALG00010002999	0.941766916	0.226596194
ENSGALG00010003000	0.38718504	0.101838932
ENSGALG00010003001	0.242149378	0.155084306
ENSGALG00010003002	0.991133936	0.373243097
ENSGALG00010003004	0.288049102	0.27865616
ENSGALG00010003006	0.975831192	0.377226411
ENSGALG00010003007	0.069815925	0.44175828
ENSGALG00010003009	0.943620939	0.179341028
ENSGALG00010003011	0.37552597	0.101692059
ENSGALG00010003017	0.813954693	0.222560812
ENSGALG00010003019	0.412897535	0.161739506

ENSGALG00010003022	0.183416587	0.000803411
ENSGALG00010003023	0.110231669	0.044134076
ENSGALG00010003024	0.983960408	0.308732372
ENSGALG00010003025	0.989741908	0.317412066
ENSGALG00010003026	0.50022725	0.538831074
ENSGALG00010003027	0.961367841	0.202644343
ENSGALG00010003030	0.937048066	0.371432484
ENSGALG00010003032	0.271701261	0.072417625
ENSGALG00010003033	0.813832316	0.595639535
ENSGALG00010003034	0.191738216	0.124332771
ENSGALG00010003035	0.921086254	0.291902831
ENSGALG00010003038	0.219386918	0.079388121
ENSGALG00010003039	0.303566744	0.121227958
ENSGALG00010003041	0.691381674	0.153691098
ENSGALG00010003042	0.688538247	0.022173363
ENSGALG00010003043	0.009429422	0.134618735
ENSGALG00010003046	0.504215335	0.238231603
ENSGALG00010003047	0.720528183	0.116255236
ENSGALG00010003048	0.682490442	0.054721849
ENSGALG00010003050	0.285272007	0.109169955
ENSGALG00010003051	0.57153765	0.230500456
ENSGALG00010003052	0.815654755	0.204954565
ENSGALG00010003053	0.101700264	0.526058573
ENSGALG00010003055	0.099618978	0.16833127
ENSGALG00010003056	0.18041281	0.185292443
ENSGALG00010003057	0.974545804	0.34135965
ENSGALG00010003058	0.676282879	0.072104425
ENSGALG00010003060	0.372757682	0.112441993
ENSGALG00010003061	0.72534336	0.267283627
ENSGALG00010003062	0.266137114	0.107784983
ENSGALG00010003063	0.990080096	0.322621179
ENSGALG00010003064	0.932020919	0.249173441
ENSGALG00010003065	0.586577347	0.171538282
ENSGALG00010003066	0.933131159	0.347543741
ENSGALG00010003068	0.847771351	0.312947041
ENSGALG00010003072	0.960972546	0.379418149
ENSGALG00010003074	0.96483777	0.359845559
ENSGALG00010003076	0.780930904	0.154271074
ENSGALG00010003077	0.817815656	0.237338784
ENSGALG00010003081	0.900450899	0.417253874
ENSGALG00010003085	0.219191757	0.127418001
ENSGALG00010003086	0.767182415	0.542373845
ENSGALG00010003087	0.912045536	0.304668672
ENSGALG00010003088	0.270451009	0.090541667
ENSGALG00010003090	0.755446653	0.231492813
ENSGALG00010003092	0.689563715	0.075413306
ENSGALG00010003094	0.471665946	0.176475399
ENSGALG00010003095	0.380790704	0.180335707
ENSGALG00010003096	0.676111699	0.174305163
ENSGALG00010003097	0.951481504	0.366024207
ENSGALG00010003098	0.264239718	0.073427965
ENSGALG00010003099	0.661315985	0.179947302
ENSGALG00010003101	0.906775458	0.39687163
ENSGALG00010003103	0.952992579	0.245502991
ENSGALG00010003106	0.349255737	0.188021623
ENSGALG00010003107	0.952353317	0.328629512

ENSGALG00010003108	0.7805579	0.365261529
ENSGALG00010003109	0.913006122	0.277664833
ENSGALG00010003110	0.489750186	0.263946287
ENSGALG00010003112	0.10155689	0.50610192
ENSGALG00010003113	0.865000985	0.201608268
ENSGALG00010003114	0.800912961	0.305090895
ENSGALG00010003115	0.77160143	0.435012841
ENSGALG00010003116	0.020861923	0.205362832
ENSGALG00010003117	0.749331414	0.167521098
ENSGALG00010003118	0.434455961	0.132085964
ENSGALG00010003119	0.511408405	0.012979867
ENSGALG00010003120	0.547247472	0.165556652
ENSGALG00010003121	0.728974195	0.031258108
ENSGALG00010003123	0.85554152	0.465113482
ENSGALG00010003125	0.923319886	0.274722881
ENSGALG00010003126	0.490923073	0.051478983
ENSGALG00010003127	0.365045337	0.128150973
ENSGALG00010003129	0.215173257	0.178900514
ENSGALG00010003131	0.873993448	0.379844842
ENSGALG00010003132	0.241134268	0.06465488
ENSGALG00010003133	0.88983497	0.335762871
ENSGALG00010003135	0.265558089	0.028821141
ENSGALG00010003136	0.316258174	0.031049574
ENSGALG00010003137	0.187514554	0.108479243
ENSGALG00010003138	0.006224473	0.058042069
ENSGALG00010003143	0.046413968	0.122175326
ENSGALG00010003149	0.747415456	0.023654901
ENSGALG00010003151	0.266137114	0.107784983
ENSGALG00010003152	0.636062063	0.156426084
ENSGALG00010003154	0.97547725	0.284058693
ENSGALG00010003155	0.777949766	0.36334837
ENSGALG00010003156	0.545913933	0.06323652
ENSGALG00010003157	0.085533546	0.570832887
ENSGALG00010003158	0.907973109	0.249789549
ENSGALG00010003159	0.737387895	0.498559729
ENSGALG00010003160	0.967125728	0.429513891
ENSGALG00010003162	0.863118987	0.449258859
ENSGALG00010003163	0.208550923	0.676301294
ENSGALG00010003164	0.993088402	0.318836368
ENSGALG00010003165	0.923705558	0.346973918
ENSGALG00010003166	0.978616624	0.351780888
ENSGALG00010003167	0.037255754	0.308963048
ENSGALG00010003168	0.97569587	0.362390606
ENSGALG00010003169	0.957341758	0.244315185
ENSGALG00010003170	0.9935769	0.352003003
ENSGALG00010003171	0.91697749	0.251240507
ENSGALG00010003174	0.861355018	0.386942891
ENSGALG00010003176	0.640322856	0.30207015
ENSGALG00010003177	0.640215088	0.389449192
ENSGALG00010003178	0.106650467	0.0179704
ENSGALG00010003179	0.266137114	0.107784983
ENSGALG00010003180	0.086603746	0.007818119
ENSGALG00010003182	0.208711821	0.162017834
ENSGALG00010003183	0.990440602	0.277214332
ENSGALG00010003184	0.779254915	0.300873577
ENSGALG00010003186	0.295532074	0.393206655

ENSGALG00010003187	0.918674657	0.311547716
ENSGALG00010003188	0.329744371	0.371370966
ENSGALG00010003189	0.625370326	0.072954359
ENSGALG00010003190	0.691174322	0.229633288
ENSGALG00010003191	0.481659976	0.058084051
ENSGALG00010003192	0.926310782	0.230371501
ENSGALG00010003193	0.909146878	0.325098423
ENSGALG00010003195	0.938550513	0.396098473
ENSGALG00010003197	0.351308294	0.32074392
ENSGALG00010003198	0.407585146	0.214208593
ENSGALG00010003199	0.100912843	0.096197224
ENSGALG00010003201	0.948193452	0.309002621
ENSGALG00010003202	0.954735225	0.319530713
ENSGALG00010003204	0.904232232	0.394148731
ENSGALG00010003205	0.956721201	0.354657318
ENSGALG00010003206	0.996779326	0.33513556
ENSGALG00010003207	0.211482089	0.284909679
ENSGALG00010003208	0.441756036	0.067921577
ENSGALG00010003210	0.967543608	0.307940798
ENSGALG00010003211	0.351196368	0.200359655
ENSGALG00010003212	0.858522707	0.122304598
ENSGALG00010003214	0.951121673	0.275677421
ENSGALG00010003215	0.083169178	0.101477029
ENSGALG00010003217	0.723269845	0.43280934
ENSGALG00010003218	0.986736667	0.264253779
ENSGALG00010003219	0.371352104	0.142693329
ENSGALG00010003220	0.960592322	0.376491187
ENSGALG00010003222	0.858514907	0.094675802
ENSGALG00010003223	0.951964032	0.306792046
ENSGALG00010003225	0.955572748	0.268416834
ENSGALG00010003226	0.378529261	0.203754877
ENSGALG00010003227	0.779604253	0.022854952
ENSGALG00010003228	0.388919998	0.28043621
ENSGALG00010003229	0.905181014	0.355437018
ENSGALG00010003230	0.535583716	0.241999674
ENSGALG00010003231	0.77202336	0.300389658
ENSGALG00010003234	0.539557773	0.044261676
ENSGALG00010003237	0.834154027	0.289944839
ENSGALG00010003238	0.529678578	0.216292576
ENSGALG00010003239	0.89506619	0.272042447
ENSGALG00010003240	0.90368163	0.347748439
ENSGALG00010003241	0.608592208	0.092638622
ENSGALG00010003242	0.860571815	0.486502047
ENSGALG00010003243	0.943521832	0.314453042
ENSGALG00010003245	0.422096609	0.146671079
ENSGALG00010003246	0.66345725	0.175709534
ENSGALG00010003247	0.729456255	0.287735768
ENSGALG00010003248	0.965505788	0.295513128
ENSGALG00010003249	0.134903132	0.266293164
ENSGALG00010003250	0.635798482	0.167682344
ENSGALG00010003251	0.930619498	0.349340272
ENSGALG00010003252	0.09088815	0.213622299
ENSGALG00010003253	0.783877527	0.418191224
ENSGALG00010003254	0.517575552	0.102288255
ENSGALG00010003255	0.940824884	0.375258765
ENSGALG00010003259	0.861804702	0.19253104

ENSGALG00010003260	0.745703627	0.043894129
ENSGALG00010003261	0.851141409	0.300300292
ENSGALG00010003262	0.452004334	0.318131111
ENSGALG00010003264	0.018022442	0.059184271
ENSGALG00010003266	0.208550923	0.676301294
ENSGALG00010003268	0.144281563	0.451596598
ENSGALG00010003269	0.17090271	0.105936578
ENSGALG00010003271	0.899297913	0.291269664
ENSGALG00010003272	0.407989348	0.226340472
ENSGALG00010003273	0.951067143	0.385954433
ENSGALG00010003274	0.413555737	0.082037797
ENSGALG00010003275	0.825045118	0.341005756
ENSGALG00010003276	0.877871796	0.33285196
ENSGALG00010003278	0.988797014	0.339081359
ENSGALG00010003279	0.928969476	0.386660928
ENSGALG00010003280	0.93259041	0.216102722
ENSGALG00010003283	0.28441977	0.17808526
ENSGALG00010003287	0.088975161	0.146877788
ENSGALG00010003288	0.184512425	0.164683615
ENSGALG00010003289	0.879225924	0.365397017
ENSGALG00010003290	0.726501539	0.240442693
ENSGALG00010003291	0.625052083	0.345113977
ENSGALG00010003292	0.991586612	0.328544888
ENSGALG00010003295	0.92267673	0.273614234
ENSGALG00010003296	0.398908642	0.182257139
ENSGALG00010003297	0.265481862	0.322207991
ENSGALG00010003298	0.55108881	0.524673942
ENSGALG00010003299	0.992049793	0.324825389
ENSGALG00010003300	0.976806815	0.327583062
ENSGALG00010003302	0.710524716	0.386123802
ENSGALG00010003303	0.114550871	0.411449492
ENSGALG00010003304	0.267959468	0.18114852
ENSGALG00010003305	0.7706991	0.266550858
ENSGALG00010003306	0.44601038	0.147757387
ENSGALG00010003307	0.939943216	0.415976004
ENSGALG00010003308	0.928996765	0.389153541
ENSGALG00010003309	0.40289019	0.168989184
ENSGALG00010003310	0.116964672	0.301144361
ENSGALG00010003312	0.619369934	0.464396294
ENSGALG00010003315	0.341344272	0.335570742
ENSGALG00010003317	0.900717649	0.2723442
ENSGALG00010003319	0.014423969	0.094604939
ENSGALG00010003320	0.189240572	0.04996112
ENSGALG00010003321	0.530572456	0.079071089
ENSGALG00010003322	0.927113283	0.286709003
ENSGALG00010003323	0.74873922	0.117733026
ENSGALG00010003325	0.333291256	0.229057711
ENSGALG00010003326	0.262922434	0.138601464
ENSGALG00010003330	0.967186807	0.392194328
ENSGALG00010003331	0.9581695	0.37832599
ENSGALG00010003339	0.299741029	0.074474613
ENSGALG00010003342	0.555518849	0.233941357
ENSGALG00010003343	0.64373379	0.198888235
ENSGALG00010003344	0.560978829	0.242248637
ENSGALG00010003345	0.90743012	0.334058611
ENSGALG00010003347	0.620793401	0.13696321

ENSGALG00010003348	0.801453869	0.084057909
ENSGALG00010003350	0.835666955	0.317206634
ENSGALG00010003352	0.931579292	0.328480555
ENSGALG00010003353	0.498442246	0.209516228
ENSGALG00010003355	0.384865276	0.180497163
ENSGALG00010003358	0.866124314	0.392408015
ENSGALG00010003363	0.248210599	0.171476859
ENSGALG00010003364	0.622918014	0.150976809
ENSGALG00010003366	0.395752072	0.195088037
ENSGALG00010003367	0.364661545	0.023442498
ENSGALG00010003368	0.679893291	0.327883078
ENSGALG00010003369	0.73486278	0.176490025
ENSGALG00010003370	0.485030426	0.2000668
ENSGALG00010003371	0.222929393	0.121601676
ENSGALG00010003372	0.108149065	0.085747997
ENSGALG00010003373	0.498400664	0.50680211
ENSGALG00010003374	0.583259982	0.585376227
ENSGALG00010003375	0.895998636	0.327739174
ENSGALG00010003378	0.291530067	0.097635261
ENSGALG00010003379	0.077669352	0.09831719
ENSGALG00010003382	0.95165418	0.225206141
ENSGALG00010003383	0.915095327	0.369716856
ENSGALG00010003384	0.162255913	0.050717563
ENSGALG00010003386	0.298505865	0.422939515
ENSGALG00010003388	0.731703062	0.433402021
ENSGALG00010003389	0.355837172	0.200776487
ENSGALG00010003391	0.386299037	0.252195982
ENSGALG00010003392	0.357358991	0.136196478
ENSGALG00010003393	0.870511781	0.056146624
ENSGALG00010003394	0.978619318	0.265392973
ENSGALG00010003397	0.568771776	0.282422625
ENSGALG00010003400	0.984612528	0.31267959
ENSGALG00010003402	0.569177905	0.260291683
ENSGALG00010003403	0.869640651	0.246512396
ENSGALG00010003404	0.284225216	0.103866786
ENSGALG00010003405	0.271522808	0.294558867
ENSGALG00010003406	0.971467796	0.398709394
ENSGALG00010003407	0.218042719	0.172896263
ENSGALG00010003408	0.026966462	0.158284821
ENSGALG00010003412	0.901750372	0.229371015
ENSGALG00010003413	0.565562602	0.199452658
ENSGALG00010003415	0.280762285	0.221260328
ENSGALG00010003416	0.277566769	0.107857127
ENSGALG00010003419	0.179259662	0.209057242
ENSGALG00010003421	0.264239718	0.073427965
ENSGALG00010003422	0.386050996	0.14466628
ENSGALG00010003423	0.282976025	0.145658897
ENSGALG00010003428	0.595406142	0.197447668
ENSGALG00010003429	0.345957381	0.377989906
ENSGALG00010003431	0.684365232	0.05876826
ENSGALG00010003432	0.935106485	0.15690481
ENSGALG00010003434	0.243326925	0.091719824
ENSGALG00010003437	0.666786482	0.072034928
ENSGALG00010003438	0.433619983	0.397964641
ENSGALG00010003439	0.979751919	0.390219881
ENSGALG00010003441	0.743633063	0.300548019

ENSGALG00010003442	0.161824623	0.050157963
ENSGALG00010003444	0.567820428	0.173537088
ENSGALG00010003445	0.75078083	0.221899468
ENSGALG00010003446	0.435556311	0.320315436
ENSGALG00010003447	0.895715995	0.278352729
ENSGALG00010003448	0.973007341	0.337147778
ENSGALG00010003449	0.77221778	0.140201459
ENSGALG00010003451	0.884230452	0.273144268
ENSGALG00010003452	0.669829352	0.02350625
ENSGALG00010003454	0.921512683	0.253925153
ENSGALG00010003455	0.877570749	0.370238298
ENSGALG00010003456	0.968249193	0.26905128
ENSGALG00010003457	0.340035978	0.184426891
ENSGALG00010003458	0.258128488	0.262877821
ENSGALG00010003459	0.530374864	0.12556435
ENSGALG00010003461	0.882311852	0.330385353
ENSGALG00010003462	0.526680875	0.329544002
ENSGALG00010003463	0.982686196	0.304429578
ENSGALG00010003464	0.612438115	0.091226236
ENSGALG00010003466	0.643705295	0.065322629
ENSGALG00010003468	0.095284118	0.469054078
ENSGALG00010003469	0.035158038	0.119871525
ENSGALG00010003470	0.980133249	0.281862488
ENSGALG00010003471	0.612651951	0.328358215
ENSGALG00010003472	0.906749147	0.260175874
ENSGALG00010003473	0.938662212	0.1653475
ENSGALG00010003474	0.63341263	0.242974946
ENSGALG00010003475	0.398518281	0.225017969
ENSGALG00010003476	0.349184898	0.351999142
ENSGALG00010003477	0.410090651	0.015209216
ENSGALG00010003478	0.989054802	0.315843295
ENSGALG00010003479	0.191718042	0.209764603
ENSGALG00010003481	0.42029357	0.05696234
ENSGALG00010003482	0.414089516	0.075029542
ENSGALG00010003483	0.227513041	0.26806072
ENSGALG00010003484	0.3637525	0.159288563
ENSGALG00010003485	0.840067072	0.413811957
ENSGALG00010003486	0.271701261	0.072417625
ENSGALG00010003487	0.308758304	0.100827364
ENSGALG00010003489	0.70946041	0.5603584
ENSGALG00010003493	0.943278967	0.342946143
ENSGALG00010003494	0.271701261	0.072417625
ENSGALG00010003495	0.562927339	0.083208267
ENSGALG00010003496	0.366667574	0.096374266
ENSGALG00010003498	0.545077606	0.000787856
ENSGALG00010003499	0.845903973	0.289650688
ENSGALG00010003500	0.474087893	0.045382483
ENSGALG00010003503	0.480347077	0.208770509
ENSGALG00010003505	0.767492577	0.103815419
ENSGALG00010003506	0.724055013	0.072856124
ENSGALG00010003507	0.890913526	0.302434554
ENSGALG00010003509	0.792208591	0.575786037
ENSGALG00010003511	0.772697729	0.035620265
ENSGALG00010003512	0.856535228	0.367339157
ENSGALG00010003513	0.416448651	0.410231292
ENSGALG00010003515	0.553818657	0.306975281

ENSGALG00010003516	0.677344975	0.297625833
ENSGALG00010003517	0.866134698	0.184801696
ENSGALG00010003518	0.967693306	0.334376198
ENSGALG00010003519	0.54623158	0.193385731
ENSGALG00010003520	0.476448648	0.301148952
ENSGALG00010003522	0.905514678	0.392048707
ENSGALG00010003523	0.990113085	0.300859823
ENSGALG00010003525	0.607408374	0.239024614
ENSGALG00010003528	0.069237868	0.091618427
ENSGALG00010003531	0.030464363	0.054912662
ENSGALG00010003535	0.442790485	0.317590288
ENSGALG00010003539	0.725594986	0.271940912
ENSGALG00010003541	0.540698392	0.068927106
ENSGALG00010003542	0.495183892	0.066100648
ENSGALG00010003543	0.9337097	0.328157923
ENSGALG00010003547	0.601195446	0.297188712
ENSGALG00010003550	0.836547624	0.161741682
ENSGALG00010003551	0.474173002	0.19716725
ENSGALG00010003552	0.044378794	0.339771304
ENSGALG00010003555	0.308301889	0.170345784
ENSGALG00010003556	0.539889122	0.103003819
ENSGALG00010003558	0.985262493	0.338918116
ENSGALG00010003559	0.009812001	0.5166245
ENSGALG00010003560	0.76727397	0.26541017
ENSGALG00010003564	0.289526293	0.199376796
ENSGALG00010003567	0.03285381	0.446229691
ENSGALG00010003569	0.235630588	0.132393411
ENSGALG00010003571	0.885703583	0.263787864
ENSGALG00010003573	0.336296695	0.157126423
ENSGALG00010003577	0.395556858	0.102965232
ENSGALG00010003581	0.053679082	0.152566612
ENSGALG00010003582	0.991907943	0.285945047
ENSGALG00010003583	0.86778449	0.456869931
ENSGALG00010003585	0.643075543	0.232657338
ENSGALG00010003586	0.187514554	0.108479243
ENSGALG00010003589	0.883523569	0.411295598
ENSGALG00010003591	0.587353234	0.226044568
ENSGALG00010003595	0.117694755	0.383805242
ENSGALG00010003596	0.908052265	0.44227001
ENSGALG00010003597	0.58239358	0.240887491
ENSGALG00010003600	0.315496541	0.220943394
ENSGALG00010003601	0.404216238	0.144864446
ENSGALG00010003603	0.381415582	0.158761082
ENSGALG00010003604	0.334308133	0.197241689
ENSGALG00010003606	0.689838987	0.096135982
ENSGALG00010003610	0.496123135	0.059822433
ENSGALG00010003611	0.075427953	0.252942273
ENSGALG00010003615	0.373811017	0.102306018
ENSGALG00010003616	0.844209099	0.191912409
ENSGALG00010003617	0.82877473	0.428538338
ENSGALG00010003618	0.849325621	0.236493937
ENSGALG00010003619	0.99263052	0.372167721
ENSGALG00010003627	0.418853559	0.255119119
ENSGALG00010003629	0.662322138	0.093784319
ENSGALG00010003631	0.285272007	0.109169955
ENSGALG00010003632	0.472893374	0.124519532

ENSGALG00010003633	0.874628978	0.049126022
ENSGALG00010003634	0.961751717	0.454254215
ENSGALG00010003639	0.421908167	0.090398445
ENSGALG00010003641	0.265338374	0.113300876
ENSGALG00010003642	0.794658173	0.012865267
ENSGALG00010003646	0.345622816	0.144937494
ENSGALG00010003647	0.663877396	0.284968529
ENSGALG00010003652	0.832309419	0.29497811
ENSGALG00010003656	0.955212678	0.160723665
ENSGALG00010003659	0.467248351	0.003156925
ENSGALG00010003660	0.601717212	0.323463603
ENSGALG00010003662	0.305732779	0.28673228
ENSGALG00010003663	0.936007033	0.105639193
ENSGALG00010003664	0.325327998	0.037399662
ENSGALG00010003665	0.970020923	0.262880879
ENSGALG00010003666	0.984071952	0.376305979
ENSGALG00010003668	0.745910226	0.212745565
ENSGALG00010003669	0.333095414	0.134815547
ENSGALG00010003670	0.203991683	0.058221925
ENSGALG00010003671	0.546775682	0.073560151
ENSGALG00010003674	0.031609767	0.078395419
ENSGALG00010003676	0.980343157	0.349226762
ENSGALG00010003677	0.264239718	0.073427965
ENSGALG00010003678	0.337896669	0.259230612
ENSGALG00010003679	0.109342752	0.277812353
ENSGALG00010003680	0.928092902	0.32832635
ENSGALG00010003681	0.802117468	0.288164114
ENSGALG00010003682	0.397516991	0.125566479
ENSGALG00010003683	0.613259095	0.20316246
ENSGALG00010003684	0.216433634	0.680598293
ENSGALG00010003686	0.1806966	0.412338801
ENSGALG00010003688	0.058767	0.066578089
ENSGALG00010003689	0.364871524	0.123582396
ENSGALG00010003691	0.403879276	0.057889159
ENSGALG00010003692	0.050334666	0.261114476
ENSGALG00010003693	0.151745883	0.013042855
ENSGALG00010003695	0.963794281	0.314590462
ENSGALG00010003698	0.740711035	0.327199718
ENSGALG00010003700	0.534871678	0.341380531
ENSGALG00010003701	0.845611439	0.254967113
ENSGALG00010003702	0.112003557	0.251178953
ENSGALG00010003703	0.590175	0.335121021
ENSGALG00010003704	0.938494263	0.421116966
ENSGALG00010003705	0.940155994	0.371214651
ENSGALG00010003706	0.623359778	0.17818241
ENSGALG00010003707	0.482768793	0.331455444
ENSGALG00010003712	0.800238416	0.337330986
ENSGALG00010003713	0.957916368	0.418772429
ENSGALG00010003714	0.449308097	0.005700852
ENSGALG00010003715	0.513720473	0.212256414
ENSGALG00010003716	0.118666793	0.432641873
ENSGALG00010003717	0.930211006	0.288941534
ENSGALG00010003718	0.720805487	0.323692607
ENSGALG00010003719	0.057573501	0.14967922
ENSGALG00010003720	0.180551543	0.226519768
ENSGALG00010003721	0.091660648	0.263774691

ENSGALG00010003722	0.41016831	0.221865089
ENSGALG00010003723	0.661457835	0.272298077
ENSGALG00010003724	0.904703366	0.308172809
ENSGALG00010003725	0.606644321	0.004445315
ENSGALG00010003726	0.989556861	0.333102143
ENSGALG00010003727	0.93218123	0.232380256
ENSGALG00010003728	0.635167172	0.286355746
ENSGALG00010003729	0.767558412	0.25193485
ENSGALG00010003730	0.517678123	0.1475315
ENSGALG00010003731	0.055330196	0.271475439
ENSGALG00010003732	0.543769497	0.24197464
ENSGALG00010003733	0.221289906	0.258940391
ENSGALG00010003734	0.917206458	0.154695552
ENSGALG00010003736	0.377046878	0.298393455
ENSGALG00010003737	0.470861304	0.051290929
ENSGALG00010003738	0.215461258	0.191543784
ENSGALG00010003739	0.52593984	0.155196339
ENSGALG00010003740	0.679093913	0.158265774
ENSGALG00010003742	0.651452626	0.226706596
ENSGALG00010003743	0.796172359	0.083345939
ENSGALG00010003744	0.291391904	0.136533702
ENSGALG00010003745	0.407193203	0.187860213
ENSGALG00010003746	0.704846956	0.14275699
ENSGALG00010003748	0.270451009	0.090541667
ENSGALG00010003749	0.75704988	0.57139259
ENSGALG00010003750	0.997426663	0.33614887
ENSGALG00010003751	0.39155441	0.07460765
ENSGALG00010003753	0.965372443	0.306983052
ENSGALG00010003754	0.972848047	0.416219372
ENSGALG00010003755	0.871187049	0.220924192
ENSGALG00010003756	0.474268348	0.43463797
ENSGALG00010003757	0.545743575	0.172584057
ENSGALG00010003758	0.948464723	0.150552026
ENSGALG00010003759	0.264239718	0.073427965
ENSGALG00010003760	0.249651824	0.095925253
ENSGALG00010003761	0.975613863	0.246535295
ENSGALG00010003762	0.980762475	0.337611014
ENSGALG00010003763	0.056816994	0.074976849
ENSGALG00010003764	0.930531628	0.331936806
ENSGALG00010003765	0.917650638	0.198631761
ENSGALG00010003766	0.781510113	0.318030064
ENSGALG00010003767	0.261145848	0.037777787
ENSGALG00010003768	0.546358234	0.136643049
ENSGALG00010003769	0.562958499	0.028674529
ENSGALG00010003770	0.147647715	0.061176691
ENSGALG00010003771	0.044367189	0.300494749
ENSGALG00010003772	0.380946785	0.181642936
ENSGALG00010003773	0.098617306	0.195747229
ENSGALG00010003774	0.90450863	0.285568639
ENSGALG00010003775	0.861392241	0.231321253
ENSGALG00010003776	0.922070107	0.281517367
ENSGALG00010003777	0.394367059	0.123226926
ENSGALG00010003778	0.274903919	0.104782153
ENSGALG00010003779	0.008043983	0.110635211
ENSGALG00010003781	0.449716953	0.054005331
ENSGALG00010003783	0.519706477	0.040584863

ENSGALG00010003784	0.895282301	0.209866027
ENSGALG00010003785	0.193201469	0.214500937
ENSGALG00010003786	0.118498134	0.255318368
ENSGALG00010003787	0.920466763	0.277765167
ENSGALG00010003788	0.307782724	0.120262487
ENSGALG00010003789	0.383975425	0.311146784
ENSGALG00010003790	0.989781018	0.300986304
ENSGALG00010003791	0.777754481	0.093726757
ENSGALG00010003792	0.631794336	0.225727293
ENSGALG00010003793	0.604029498	0.302561221
ENSGALG00010003794	0.230413829	0.265058991
ENSGALG00010003795	0.988215125	0.303431202
ENSGALG00010003796	0.177336903	0.109240256
ENSGALG00010003797	0.97964055	0.325554325
ENSGALG00010003798	0.396668506	0.181671381
ENSGALG00010003799	0.199172908	0.188012133
ENSGALG00010003800	0.583204113	0.200288107
ENSGALG00010003801	0.798079253	0.281626084
ENSGALG00010003803	0.847319142	0.338104071
ENSGALG00010003804	0.713060647	0.403592649
ENSGALG00010003806	0.974965209	0.340084703
ENSGALG00010003807	0.79901806	0.406597191
ENSGALG00010003808	0.873063608	0.246724875
ENSGALG00010003809	0.723141333	0.2315194
ENSGALG00010003810	0.989865776	0.297555757
ENSGALG00010003811	0.996054664	0.33456763
ENSGALG00010003812	0.612489052	0.426648321
ENSGALG00010003813	0.18428844	0.34935688
ENSGALG00010003814	0.592826421	0.462936843
ENSGALG00010003815	0.890536073	0.456834226
ENSGALG00010003816	0.878667234	0.493541792
ENSGALG00010003817	0.554842987	0.330429702
ENSGALG00010003818	0.837723911	0.367287562
ENSGALG00010003819	0.699905242	0.107513339
ENSGALG00010003820	0.435733688	0.052192253
ENSGALG00010003821	0.179192807	0.011314274
ENSGALG00010003822	0.672276415	0.441193988
ENSGALG00010003823	0.935567815	0.283806526
ENSGALG00010003824	0.5872904	0.383302272
ENSGALG00010003825	0.604721454	0.205554982
ENSGALG00010003826	0.627594356	0.053571447
ENSGALG00010003827	0.497941643	0.295494368
ENSGALG00010003828	0.633188691	0.367388327
ENSGALG00010003829	0.844393352	0.328961286
ENSGALG00010003830	0.347224266	0.058576525
ENSGALG00010003831	0.911689961	0.252679173
ENSGALG00010003832	0.963463941	0.211376003
ENSGALG00010003833	0.030970995	0.234027066
ENSGALG00010003834	0.176855442	0.203885196
ENSGALG00010003835	0.962467558	0.265241084
ENSGALG00010003836	0.8997595	0.270241643
ENSGALG00010003837	0.129945308	0.012445902
ENSGALG00010003838	0.08008411	0.183424084
ENSGALG00010003839	0.947170988	0.299618485
ENSGALG00010003841	0.948585486	0.339566238
ENSGALG00010003842	0.688847734	0.124493083

ENSGALG00010003843	0.132479459	0.195113064
ENSGALG00010003844	0.788254132	0.424731953
ENSGALG00010003845	0.985758225	0.314697236
ENSGALG00010003847	0.956098346	0.333772581
ENSGALG00010003848	0.677611343	0.23432597
ENSGALG00010003849	0.235630588	0.132393411
ENSGALG00010003850	0.915384716	0.180784202
ENSGALG00010003851	0.742281021	0.134307293
ENSGALG00010003852	0.868133069	0.340568128
ENSGALG00010003853	0.656991224	0.125946268
ENSGALG00010003854	0.892396902	0.361507009
ENSGALG00010003855	0.839479689	0.46890711
ENSGALG00010003856	0.771136536	0.289018104
ENSGALG00010003858	0.991669304	0.344768352
ENSGALG00010003860	0.881212834	0.238643819
ENSGALG00010003861	0.939946764	0.338994599
ENSGALG00010003862	0.983996694	0.334826707
ENSGALG00010003863	0.1074886	0.261268043
ENSGALG00010003864	0.325830509	0.254423033
ENSGALG00010003865	0.943448117	0.314515995
ENSGALG00010003868	0.138168917	0.103922961
ENSGALG00010003869	0.750258674	0.043346671
ENSGALG00010003870	0.633827119	0.066062913
ENSGALG00010003871	0.722946434	0.167617145
ENSGALG00010003872	0.222387642	0.257917365
ENSGALG00010003874	0.981804526	0.378524019
ENSGALG00010003875	0.778328958	0.089619346
ENSGALG00010003877	0.23788527	0.002817365
ENSGALG00010003878	0.83377193	0.27262198
ENSGALG00010003879	0.932164517	0.151749942
ENSGALG00010003882	0.885255909	0.07160147
ENSGALG00010003883	0.059020822	0.180345661
ENSGALG00010003884	0.453005608	0.1015344
ENSGALG00010003887	0.84160079	0.117422789
ENSGALG00010003889	0.877934909	0.160939603
ENSGALG00010003890	0.742491293	0.247659545
ENSGALG00010003893	0.465092004	0.15227717
ENSGALG00010003894	0.982112202	0.271533705
ENSGALG00010003895	0.865390641	0.040395729
ENSGALG00010003896	0.949813013	0.283777756
ENSGALG00010003897	0.817520907	0.212586914
ENSGALG00010003898	0.884639865	0.267782455
ENSGALG00010003899	0.081094107	0.149632189
ENSGALG00010003900	0.912480796	0.252478551
ENSGALG00010003902	0.953737407	0.364543375
ENSGALG00010003903	0.986133114	0.36452548
ENSGALG00010003909	0.557727677	0.08580001
ENSGALG00010003910	0.911399123	0.349760719
ENSGALG00010003913	0.965178233	0.295662895
ENSGALG00010003914	0.915951999	0.262287165
ENSGALG00010003915	0.623832624	0.104322821
ENSGALG00010003918	0.534759157	0.65242646
ENSGALG00010003920	0.804037518	0.34928034
ENSGALG00010003921	0.406978523	0.109363757
ENSGALG00010003922	0.245700908	0.010899108
ENSGALG00010003923	0.654813399	0.194817717

ENSGALG00010003924	0.150803076	0.13717728
ENSGALG00010003925	0.899993121	0.176827636
ENSGALG00010003928	0.960732877	0.297270655
ENSGALG00010003929	0.520559197	0.207634232
ENSGALG00010003932	0.453713345	0.091608454
ENSGALG00010003933	0.972079829	0.404884564
ENSGALG00010003935	0.521491133	0.242330835
ENSGALG00010003937	0.900350235	0.397562657
ENSGALG00010003939	0.335553126	0.154693383
ENSGALG00010003940	0.778920323	0.345226129
ENSGALG00010003941	0.929827556	0.37439592
ENSGALG00010003943	0.946158543	0.316220288
ENSGALG00010003944	0.698163581	0.293680279
ENSGALG00010003945	0.023281626	0.151646943
ENSGALG00010003948	0.206871925	0.248714914
ENSGALG00010003950	0.877692685	0.293741007
ENSGALG00010003951	0.475975319	0.223012701
ENSGALG00010003953	0.80126755	0.144447442
ENSGALG00010003956	0.275073634	0.137563663
ENSGALG00010003958	0.979470773	0.219741293
ENSGALG00010003959	0.40407931	0.24317184
ENSGALG00010003960	0.957374533	0.270754325
ENSGALG00010003961	0.141302448	0.094724793
ENSGALG00010003962	0.893912084	0.192900538
ENSGALG00010003963	0.110625341	0.160302958
ENSGALG00010003965	0.657173864	0.21254842
ENSGALG00010003968	0.935094158	0.382582172
ENSGALG00010003972	0.991868279	0.348348043
ENSGALG00010003977	0.971992644	0.239696401
ENSGALG00010003978	0.981950885	0.343187228
ENSGALG00010003980	0.965461805	0.284195894
ENSGALG00010003981	0.989987189	0.327497146
ENSGALG00010003982	0.958162658	0.322308687
ENSGALG00010003985	0.92769978	0.272875568
ENSGALG00010003986	0.892062425	0.422349681
ENSGALG00010003987	0.050201373	0.155932479
ENSGALG00010003988	0.27861604	0.105617638
ENSGALG00010003990	0.253183748	0.134355641
ENSGALG00010003993	0.968800742	0.279965467
ENSGALG00010003994	0.990056966	0.35181895
ENSGALG00010003995	0.63034915	0.550089121
ENSGALG00010004000	0.889140674	0.237009667
ENSGALG00010004001	0.95616087	0.254235744
ENSGALG00010004002	0.918378169	0.334004687
ENSGALG00010004003	0.115651573	0.417972781
ENSGALG00010004006	0.322445922	0.072805369
ENSGALG00010004007	0.642708686	0.32576823
ENSGALG00010004012	0.700122013	0.409428423
ENSGALG00010004013	0.814699905	0.268479074
ENSGALG00010004015	0.492523084	0.200387707
ENSGALG00010004016	0.312353223	0.206595394
ENSGALG00010004018	0.904016202	0.217971197
ENSGALG00010004023	0.404886942	0.037874908
ENSGALG00010004024	0.33836361	0.307557996
ENSGALG00010004029	0.89457251	0.240099769
ENSGALG00010004033	0.646046785	0.199598976

ENSGALG00010004034	0.959816009	0.268626716
ENSGALG00010004035	0.941853178	0.145220012
ENSGALG00010004037	0.100664681	0.185461778
ENSGALG00010004038	0.951539002	0.247116423
ENSGALG00010004039	0.889312088	0.249666283
ENSGALG00010004040	0.906108257	0.356570558
ENSGALG00010004041	0.613586786	0.490913623
ENSGALG00010004042	0.003319633	0.247171187
ENSGALG00010004043	0.874683009	0.283537396
ENSGALG00010004044	0.957979406	0.392582104
ENSGALG00010004045	0.099916924	0.144054513
ENSGALG00010004046	0.853286345	0.322695462
ENSGALG00010004047	0.620044465	0.585199781
ENSGALG00010004049	0.94936813	0.308259442
ENSGALG00010004050	0.782674801	0.245504622
ENSGALG00010004053	0.832676558	0.100459625
ENSGALG00010004054	0.683421265	0.158627862
ENSGALG00010004055	0.979486664	0.317777577
ENSGALG00010004056	0.565771866	0.245462375
ENSGALG00010004057	0.566698674	0.003569204
ENSGALG00010004058	0.980009722	0.275506464
ENSGALG00010004060	0.8928937	0.466827156
ENSGALG00010004061	0.288492687	0.199597344
ENSGALG00010004063	0.834049516	0.3997365
ENSGALG00010004064	0.882132116	0.136183696
ENSGALG00010004065	0.380773522	0.245009732
ENSGALG00010004066	0.159710197	0.212441481
ENSGALG00010004067	0.05558615	0.124683193
ENSGALG00010004069	0.277566769	0.107857127
ENSGALG00010004070	0.993079359	0.277628816
ENSGALG00010004071	0.186300686	0.087818217
ENSGALG00010004072	0.984515914	0.277000683
ENSGALG00010004073	0.085497345	0.472198778
ENSGALG00010004074	0.288966659	0.013641522
ENSGALG00010004076	0.969229377	0.255829973
ENSGALG00010004077	0.845716191	0.413583645
ENSGALG00010004079	0.964674124	0.364424618
ENSGALG00010004080	0.045361648	0.071024779
ENSGALG00010004081	0.952912584	0.331259905
ENSGALG00010004082	0.531046089	0.375217916
ENSGALG00010004083	0.868968726	0.271345486
ENSGALG00010004085	0.203724337	0.054897037
ENSGALG00010004086	0.67034786	0.254645971
ENSGALG00010004087	0.96440984	0.292846054
ENSGALG00010004088	0.85424739	0.334690658
ENSGALG00010004089	0.380163374	0.082113198
ENSGALG00010004090	0.381744995	0.351846763
ENSGALG00010004091	0.345111395	0.412515077
ENSGALG00010004092	0.977789549	0.238396099
ENSGALG00010004093	0.845566009	0.196639482
ENSGALG00010004094	0.322075766	0.335418656
ENSGALG00010004095	0.817714286	0.401848322
ENSGALG00010004096	0.776546685	0.228552278
ENSGALG00010004097	0.12300267	0.12002686
ENSGALG00010004098	0.697318972	0.131723542
ENSGALG00010004099	0.952723909	0.307352854

ENSGALG00010004100	0.005753495	0.103741412
ENSGALG00010004101	0.856858643	0.207748003
ENSGALG00010004102	0.90839725	0.248821285
ENSGALG00010004103	0.739067945	0.393355781
ENSGALG00010004104	0.89227608	0.354853732
ENSGALG00010004105	0.869073256	0.154611531
ENSGALG00010004106	0.091979775	0.34528311
ENSGALG00010004107	0.917406213	0.181274347
ENSGALG00010004108	0.060018443	0.226151612
ENSGALG00010004109	0.925540569	0.188499205
ENSGALG00010004110	0.604139568	0.273819251
ENSGALG00010004111	0.928218748	0.224689437
ENSGALG00010004112	0.386067924	0.133174077
ENSGALG00010004113	0.940473626	0.492324477
ENSGALG00010004114	0.211714813	0.010861468
ENSGALG00010004116	0.929838842	0.48625635
ENSGALG00010004117	0.975311557	0.354035778
ENSGALG00010004118	0.388404098	0.148641621
ENSGALG00010004119	0.976091634	0.304503233
ENSGALG00010004120	0.561728293	0.147037025
ENSGALG00010004121	0.405797923	0.13200104
ENSGALG00010004122	0.98939801	0.335212139
ENSGALG00010004123	0.689283107	0.451525122
ENSGALG00010004125	0.441086231	0.184475956
ENSGALG00010004127	0.256116925	0.391633556
ENSGALG00010004128	0.838732592	0.34842756
ENSGALG00010004130	0.372796048	0.167811456
ENSGALG00010004131	0.974705074	0.37361409
ENSGALG00010004132	0.378986826	0.110289096
ENSGALG00010004133	0.474701096	0.332010266
ENSGALG00010004135	0.730954181	0.450018784
ENSGALG00010004137	0.971912178	0.410492033
ENSGALG00010004138	0.758870699	0.24764207
ENSGALG00010004139	0.874899766	0.318465679
ENSGALG00010004140	0.0712284	0.18890371
ENSGALG00010004141	0.993861479	0.312699508
ENSGALG00010004142	0.950221625	0.413538937
ENSGALG00010004143	0.246513322	0.52174039
ENSGALG00010004144	0.643017503	0.266201275
ENSGALG00010004145	0.222104581	0.140461329
ENSGALG00010004146	0.806816675	0.12756736
ENSGALG00010004147	0.645584134	0.140144021
ENSGALG00010004148	0.983287391	0.379693709
ENSGALG00010004149	0.836699109	0.115060666
ENSGALG00010004150	0.415966549	0.08338041
ENSGALG00010004153	0.395556858	0.102965232
ENSGALG00010004154	0.991926992	0.365790842
ENSGALG00010004158	0.867850541	0.298852548
ENSGALG00010004164	0.146555911	0.157925269
ENSGALG00010004172	0.959698094	0.377436985
ENSGALG00010004173	0.347516458	0.258228165
ENSGALG00010004175	0.624742806	0.253038781
ENSGALG00010004177	0.090916672	0.240216732
ENSGALG00010004178	0.358543526	0.309448517
ENSGALG00010004181	0.266170488	0.124364713
ENSGALG00010004187	0.536295729	0.072845589

ENSGALG00010004188	0.958609039	0.440565962
ENSGALG00010004191	0.946977769	0.238202463
ENSGALG00010004194	0.685856161	0.217612417
ENSGALG00010004195	0.024689244	0.135282994
ENSGALG00010004196	0.231926346	0.072157719
ENSGALG00010004198	0.63360744	0.200830109
ENSGALG00010004200	0.991102315	0.294805624
ENSGALG00010004205	0.789702425	0.297026419
ENSGALG00010004207	0.940258786	0.317716632
ENSGALG00010004210	0.973344992	0.339058211
ENSGALG00010004211	0.623714681	0.234046778
ENSGALG00010004212	0.916960579	0.318002425
ENSGALG00010004214	0.92495405	0.286657307
ENSGALG00010004215	0.587757021	0.290944479
ENSGALG00010004216	0.013621403	0.107361756
ENSGALG00010004217	0.725075443	0.123624506
ENSGALG00010004218	0.308758304	0.100827364
ENSGALG00010004219	0.977569792	0.271626546
ENSGALG00010004220	0.744265555	0.312222766
ENSGALG00010004222	0.933003449	0.302459091
ENSGALG00010004224	0.837916944	0.083674994
ENSGALG00010004225	0.786181435	0.174719434
ENSGALG00010004226	0.815440663	0.275570708
ENSGALG00010004227	0.38718504	0.101838932
ENSGALG00010004228	0.775502472	0.277690292
ENSGALG00010004229	0.298140893	0.421788492
ENSGALG00010004230	0.753540761	0.141641531
ENSGALG00010004231	0.032786322	0.295792709
ENSGALG00010004233	0.503873384	0.044646797
ENSGALG00010004234	0.185981752	0.184718158
ENSGALG00010004235	0.475012278	0.125191971
ENSGALG00010004236	0.771418329	0.545654507
ENSGALG00010004237	0.960635256	0.294780671
ENSGALG00010004238	0.945484797	0.421568336
ENSGALG00010004240	0.951113432	0.364599428
ENSGALG00010004241	0.686027652	0.005468117
ENSGALG00010004242	0.532441947	0.022821239
ENSGALG00010004243	0.894650645	0.334089358
ENSGALG00010004244	0.931366373	0.378108825
ENSGALG00010004245	0.583833838	0.056791646
ENSGALG00010004246	0.014014488	0.111917686
ENSGALG00010004247	0.975507486	0.36260265
ENSGALG00010004248	0.932005912	0.304848007
ENSGALG00010004249	0.955776692	0.28174915
ENSGALG00010004250	0.855854409	0.425399177
ENSGALG00010004251	0.124678997	0.242349635
ENSGALG00010004252	0.486367749	0.198114684
ENSGALG00010004253	0.65128621	0.199550355
ENSGALG00010004254	0.192356465	0.148868047
ENSGALG00010004255	0.918763608	0.305593632
ENSGALG00010004256	0.052213134	0.383781291
ENSGALG00010004257	0.213884327	0.139873272
ENSGALG00010004258	0.029558715	0.175619474
ENSGALG00010004259	0.129682451	0.227988131
ENSGALG00010004262	0.866284219	0.332098216
ENSGALG00010004263	0.050115024	0.155140157

ENSGALG00010004264	0.873793944	0.291929929
ENSGALG00010004265	0.177104338	0.168490618
ENSGALG00010004266	0.479020464	0.125675697
ENSGALG00010004267	0.687868856	0.215664403
ENSGALG00010004273	0.235630588	0.132393411
ENSGALG00010004274	0.175678469	0.139522726
ENSGALG00010004276	0.37526789	0.118093036
ENSGALG00010004277	0.269042686	0.038441503
ENSGALG00010004278	0.265558089	0.028821141
ENSGALG00010004281	0.992190474	0.381087979
ENSGALG00010004282	0.326265175	0.058327135
ENSGALG00010004283	0.125090114	0.111434688
ENSGALG00010004284	0.256828349	0.119064955
ENSGALG00010004285	0.665769453	0.688691875
ENSGALG00010004286	0.685218381	0.010397705
ENSGALG00010004289	0.907283266	0.319906363
ENSGALG00010004290	0.109305411	0.156099278
ENSGALG00010004291	0.130760297	0.361243107
ENSGALG00010004292	0.364530168	0.264515588
ENSGALG00010004294	0.385953062	0.133261917
ENSGALG00010004295	0.811814499	0.021035032
ENSGALG00010004297	0.896032109	0.291607287
ENSGALG00010004298	0.275822561	0.000152595
ENSGALG00010004299	0.652384329	0.239956491
ENSGALG00010004300	0.953642571	0.421090256
ENSGALG00010004302	0.90738798	0.231358393
ENSGALG00010004303	0.041713203	0.225464311
ENSGALG00010004306	0.4319912	0.400197792
ENSGALG00010004307	0.954101002	0.333036028
ENSGALG00010004308	0.981835463	0.294971641
ENSGALG00010004309	0.738306623	0.150597065
ENSGALG00010004310	0.32399311	0.10716461
ENSGALG00010004311	0.386956996	0.157865948
ENSGALG00010004312	0.993918594	0.326295671
ENSGALG00010004315	0.974322193	0.340622356
ENSGALG00010004319	0.948981794	0.455189355
ENSGALG00010004320	0.208550923	0.676301294
ENSGALG00010004321	0.663508677	0.074227487
ENSGALG00010004323	0.348621627	0.000326195
ENSGALG00010004325	0.975591933	0.372880167
ENSGALG00010004327	0.374184405	0.202787171
ENSGALG00010004329	0.967397923	0.387209943
ENSGALG00010004330	0.893223227	0.294491502
ENSGALG00010004332	0.241128174	0.111231046
ENSGALG00010004333	0.806789083	0.342495903
ENSGALG00010004334	0.665685852	0.015438297
ENSGALG00010004336	0.427652329	0.27389309
ENSGALG00010004337	0.231780554	0.428571759
ENSGALG00010004338	0.996794393	0.341971921
ENSGALG00010004339	0.961988949	0.317465135
ENSGALG00010004340	0.354441585	0.107132351
ENSGALG00010004341	0.957586307	0.353601483
ENSGALG00010004342	0.884798979	0.290451455
ENSGALG00010004343	0.271701261	0.072417625
ENSGALG00010004344	0.36547348	0.429694444
ENSGALG00010004345	0.036941359	0.214921564

ENSGALG00010004346	0.809192697	0.096092871
ENSGALG00010004347	0.381608622	0.094262957
ENSGALG00010004350	0.985498437	0.362886719
ENSGALG00010004352	0.960062853	0.360268974
ENSGALG00010004355	0.826357033	0.196928215
ENSGALG00010004356	0.914800735	0.39309395
ENSGALG00010004358	0.997439569	0.336634972
ENSGALG00010004359	0.462738032	0.210802015
ENSGALG00010004360	0.973607436	0.25832629
ENSGALG00010004361	0.969675962	0.313782975
ENSGALG00010004362	0.965388485	0.295113479
ENSGALG00010004365	0.900172878	0.135317697
ENSGALG00010004366	0.951142732	0.189801964
ENSGALG00010004367	0.890987101	0.220065815
ENSGALG00010004368	0.544336427	0.433881138
ENSGALG00010004369	0.985632176	0.31850648
ENSGALG00010004370	0.500456633	0.427121612
ENSGALG00010004371	0.271701261	0.072417625
ENSGALG00010004372	0.901896318	0.228600778
ENSGALG00010004373	0.703869739	0.25001071
ENSGALG00010004374	0.994008927	0.289436661
ENSGALG00010004375	0.9259514	0.344312065
ENSGALG00010004377	0.142229601	0.306073777
ENSGALG00010004378	0.908570601	0.306849998
ENSGALG00010004379	0.936118221	0.434606086
ENSGALG00010004380	0.743544755	0.265340948
ENSGALG00010004381	0.579504438	0.216516835
ENSGALG00010004383	0.101554226	0.031780779
ENSGALG00010004384	0.732875385	0.401540658
ENSGALG00010004385	0.950181238	0.272340606
ENSGALG00010004386	0.215016821	0.656254954
ENSGALG00010004389	0.662000453	0.484069336
ENSGALG00010004390	0.707753611	0.332523576
ENSGALG00010004391	0.797315779	0.310061245
ENSGALG00010004393	0.965917185	0.290459572
ENSGALG00010004394	0.97704112	0.267523228
ENSGALG00010004395	0.088124561	0.110100763
ENSGALG00010004396	0.155528963	0.172652865
ENSGALG00010004397	0.011764112	0.292420853
ENSGALG00010004398	0.375877828	0.164310454
ENSGALG00010004399	0.429516227	0.258559703
ENSGALG00010004401	0.928767383	0.30737365
ENSGALG00010004402	0.529797453	0.211877086
ENSGALG00010004403	0.346799595	0.356949556
ENSGALG00010004404	0.08439784	0.025665108
ENSGALG00010004405	0.317823624	0.156280464
ENSGALG00010004406	0.401175792	0.16717388
ENSGALG00010004408	0.642027213	0.216126921
ENSGALG00010004409	0.96660642	0.287229646
ENSGALG00010004410	0.30914455	0.175530498
ENSGALG00010004411	0.08503538	0.178477399
ENSGALG00010004412	0.615674699	0.439391383
ENSGALG00010004414	0.886318323	0.226148055
ENSGALG00010004415	0.317468071	0.452961925
ENSGALG00010004416	0.950106756	0.258467902
ENSGALG00010004420	0.98878789	0.366265351

ENSGALG00010004421	0.386697888	0.064543587
ENSGALG00010004423	0.419908233	0.231814228
ENSGALG00010004424	0.947095633	0.273672457
ENSGALG00010004425	0.535882048	0.255153776
ENSGALG00010004429	0.270477661	0.284383882
ENSGALG00010004430	0.96386548	0.13126068
ENSGALG00010004431	0.278069745	0.045734197
ENSGALG00010004433	0.988913185	0.287386645
ENSGALG00010004434	0.471178857	0.26205905
ENSGALG00010004435	0.49355053	0.315912394
ENSGALG00010004436	0.912428814	0.25294061
ENSGALG00010004440	0.754555321	0.198634895
ENSGALG00010004442	0.672425042	0.315775436
ENSGALG00010004443	0.878329548	0.382883831
ENSGALG00010004445	0.308758304	0.100827364
ENSGALG00010004446	0.970559955	0.330375727
ENSGALG00010004448	0.498047487	0.145770051
ENSGALG00010004450	0.724757197	0.262841037
ENSGALG00010004451	0.715604329	0.132001737
ENSGALG00010004454	0.937125838	0.2013426
ENSGALG00010004457	0.967012251	0.304240096
ENSGALG00010004458	0.432712051	0.240393627
ENSGALG00010004459	0.3514537	0.11239814
ENSGALG00010004460	0.423188015	0.175642895
ENSGALG00010004461	0.311997177	0.424590371
ENSGALG00010004464	0.011199134	0.012807924
ENSGALG00010004468	0.572158844	0.115115362
ENSGALG00010004469	0.497323542	0.331011367
ENSGALG00010004471	0.958136266	0.265598891
ENSGALG00010004472	0.816900919	0.434255754
ENSGALG00010004473	0.080894863	0.117718561
ENSGALG00010004475	0.37304904	0.131841775
ENSGALG00010004476	0.830552503	0.292184903
ENSGALG00010004477	0.757163865	0.287950576
ENSGALG00010004479	0.981413323	0.34374178
ENSGALG00010004480	0.81581572	0.380788717
ENSGALG00010004481	0.952626584	0.388552216
ENSGALG00010004482	0.381415582	0.158761082
ENSGALG00010004483	0.971449555	0.32802348
ENSGALG00010004484	0.658641828	0.228999741
ENSGALG00010004485	0.520017927	0.21386933
ENSGALG00010004487	0.038549738	0.188924109
ENSGALG00010004489	0.290585849	0.181199568
ENSGALG00010004490	0.462840333	0.205813513
ENSGALG00010004491	0.463754168	0.204317474
ENSGALG00010004493	0.763710142	0.316121718
ENSGALG00010004494	0.847790247	0.262638451
ENSGALG00010004495	0.00340322	0.129953815
ENSGALG00010004500	0.73526713	0.416206303
ENSGALG00010004501	0.068247438	0.000551906
ENSGALG00010004502	0.092853389	0.297573914
ENSGALG00010004503	0.878576843	0.360954336
ENSGALG00010004504	0.102433888	0.23864989
ENSGALG00010004505	0.744371734	0.379945891
ENSGALG00010004507	0.846411908	0.200597657
ENSGALG00010004515	0.882002823	0.211912416

ENSGALG00010004516	0.051606007	0.145348651
ENSGALG00010004517	0.576319221	0.158794173
ENSGALG00010004518	0.271701261	0.072417625
ENSGALG00010004519	0.660194314	0.194854869
ENSGALG00010004520	0.924795988	0.365815313
ENSGALG00010004522	0.567535793	0.413092912
ENSGALG00010004523	0.223970335	0.243417213
ENSGALG00010004526	0.400778541	0.133797302
ENSGALG00010004529	0.040094268	0.172408119
ENSGALG00010004531	0.34252533	0.15605685
ENSGALG00010004532	0.516018781	0.228055565
ENSGALG00010004533	0.870264494	0.128340214
ENSGALG00010004534	0.983786091	0.34739939
ENSGALG00010004535	0.702419035	0.158415985
ENSGALG00010004540	0.363991468	0.139814047
ENSGALG00010004541	0.441865379	0.009139954
ENSGALG00010004542	0.723909742	0.248490033
ENSGALG00010004545	0.899488356	0.255914027
ENSGALG00010004553	0.818839796	0.57464036
ENSGALG00010004557	0.11282631	0.084691498
ENSGALG00010004560	0.654108892	0.354796412
ENSGALG00010004565	0.510606625	0.153783548
ENSGALG00010004568	0.927489998	0.320566179
ENSGALG00010004569	0.904652302	0.335606483
ENSGALG00010004570	0.226056695	0.179342739
ENSGALG00010004571	0.872857878	0.397698183
ENSGALG00010004573	0.356238393	0.010999432
ENSGALG00010004576	0.748200524	0.452451694
ENSGALG00010004577	0.280209824	0.199653512
ENSGALG00010004580	0.935431558	0.143575214
ENSGALG00010004581	0.856636139	0.269821069
ENSGALG00010004583	0.73602374	0.358662131
ENSGALG00010004584	0.921060058	0.2420016
ENSGALG00010004586	0.923099229	0.302798848
ENSGALG00010004588	0.85405856	0.21417799
ENSGALG00010004590	0.977412567	0.327874391
ENSGALG00010004591	0.923463919	0.381841782
ENSGALG00010004592	0.708962086	0.252221856
ENSGALG00010004596	0.088955713	0.448530809
ENSGALG00010004598	0.87041214	0.287145573
ENSGALG00010004601	0.929564558	0.355721044
ENSGALG00010004602	0.505952072	0.18883251
ENSGALG00010004609	0.661980792	0.10617632
ENSGALG00010004610	0.517646171	0.313291294
ENSGALG00010004616	0.900276977	0.289150828
ENSGALG00010004618	0.043111913	0.249018414
ENSGALG00010004621	0.264239718	0.073427965
ENSGALG00010004622	0.993587118	0.324655808
ENSGALG00010004623	0.070284017	0.040336485
ENSGALG00010004624	0.890595953	0.320988067
ENSGALG00010004626	0.475051411	0.142786434
ENSGALG00010004630	0.994566457	0.317846355
ENSGALG00010004633	0.364245733	0.168369964
ENSGALG00010004634	0.399453749	0.144668892
ENSGALG00010004635	0.208500176	0.144881495
ENSGALG00010004636	0.277566769	0.107857127

ENSGALG00010004637	0.530443077	0.465483656
ENSGALG00010004638	0.151405028	0.085694576
ENSGALG00010004639	0.909827413	0.291663546
ENSGALG00010004641	0.709929504	0.403922975
ENSGALG00010004642	0.368270834	0.170752567
ENSGALG00010004646	0.271701261	0.072417625
ENSGALG00010004647	0.339444113	0.045741665
ENSGALG00010004648	0.262545058	0.073484319
ENSGALG00010004649	0.387155025	0.291685884
ENSGALG00010004650	0.897418666	0.361547126
ENSGALG00010004652	0.181138151	0.171220136
ENSGALG00010004654	0.264239718	0.073427965
ENSGALG00010004657	0.185549637	0.123776305
ENSGALG00010004659	0.364245733	0.168369964
ENSGALG00010004660	0.088650565	0.468351546
ENSGALG00010004662	0.75630823	0.360073678
ENSGALG00010004663	0.675189524	0.084247767
ENSGALG00010004664	0.038961094	0.119686046
ENSGALG00010004665	0.401569976	0.281035686
ENSGALG00010004666	0.780343713	0.170786351
ENSGALG00010004668	0.049695518	0.521447482
ENSGALG00010004669	0.118694066	0.37588779
ENSGALG00010004671	0.742903863	0.433905727
ENSGALG00010004673	0.637585412	0.197218434
ENSGALG00010004680	0.395418784	0.135316935
ENSGALG00010004683	0.277566769	0.107857127
ENSGALG00010004684	0.575627026	0.199190278
ENSGALG00010004685	0.285272007	0.109169955
ENSGALG00010004689	0.908668209	0.263675772
ENSGALG00010004692	0.950152517	0.403361498
ENSGALG00010004697	0.618548594	0.22482835
ENSGALG00010004699	0.745933174	0.0392723
ENSGALG00010004701	0.02879719	0.282610319
ENSGALG00010004703	0.375545622	0.141397116
ENSGALG00010004705	0.833285377	0.367239529
ENSGALG00010004707	0.859795954	0.363470565
ENSGALG00010004708	0.935404948	0.309928764
ENSGALG00010004709	0.386114539	0.074004299
ENSGALG00010004710	0.941084566	0.33822774
ENSGALG00010004712	0.856258556	0.383672845
ENSGALG00010004713	0.81967241	0.277528982
ENSGALG00010004715	0.275093156	0.383411042
ENSGALG00010004716	0.982442006	0.325474449
ENSGALG00010004717	0.596983259	0.052639989
ENSGALG00010004718	0.081670579	0.231183566
ENSGALG00010004720	0.605417913	0.333161908
ENSGALG00010004721	0.939600532	0.34819623
ENSGALG00010004724	0.739666223	0.514459896
ENSGALG00010004726	0.367899555	0.330436197
ENSGALG00010004728	0.507856316	0.163760728
ENSGALG00010004729	0.913758773	0.239412297
ENSGALG00010004730	0.967938833	0.414685036
ENSGALG00010004732	0.189240572	0.04996112
ENSGALG00010004733	0.355639478	0.141537604
ENSGALG00010004734	0.533644827	0.034888331
ENSGALG00010004735	0.86252429	0.214727333

ENSGALG00010004736	0.991355538	0.329084154
ENSGALG00010004739	0.983990465	0.345829694
ENSGALG00010004740	0.569092242	0.181950755
ENSGALG00010004742	0.09934493	0.325628778
ENSGALG00010004743	0.393763142	0.088137251
ENSGALG00010004746	0.992049528	0.270596109
ENSGALG00010004748	0.475501392	0.010269959
ENSGALG00010004750	0.983113144	0.310827114
ENSGALG00010004751	0.382874261	0.352083209
ENSGALG00010004754	0.969405063	0.302954927
ENSGALG00010004755	0.753529858	0.299875871
ENSGALG00010004757	0.96877833	0.392038425
ENSGALG00010004758	0.939174809	0.406465948
ENSGALG00010004760	0.634381475	0.042774462
ENSGALG00010004761	0.844352294	0.288641855
ENSGALG00010004762	0.911782835	0.310370462
ENSGALG00010004763	0.378612439	0.180485529
ENSGALG00010004765	0.988773662	0.318576119
ENSGALG00010004767	0.386307259	0.413341954
ENSGALG00010004769	0.762325169	0.575760665
ENSGALG00010004774	0.393096753	0.36166642
ENSGALG00010004776	0.911177055	0.309104183
ENSGALG00010004777	0.645578878	0.378101968
ENSGALG00010004778	0.491856686	0.179680942
ENSGALG00010004780	0.029461136	0.132198366
ENSGALG00010004781	0.57929413	0.249388221
ENSGALG00010004783	0.937137434	0.294641788
ENSGALG00010004784	0.002187182	0.311210729
ENSGALG00010004787	0.774395014	0.185759952
ENSGALG00010004788	0.954068916	0.27465051
ENSGALG00010004789	0.310254517	0.364263923
ENSGALG00010004791	0.643299644	0.21790878
ENSGALG00010004792	0.915650246	0.263526142
ENSGALG00010004793	0.816913162	0.367312251
ENSGALG00010004795	0.122274331	0.457072726
ENSGALG00010004798	0.973372856	0.364484527
ENSGALG00010004800	0.932177074	0.334059763
ENSGALG00010004803	0.921414892	0.411337426
ENSGALG00010004808	0.937753373	0.232201223
ENSGALG00010004809	0.603384276	0.269293483
ENSGALG00010004813	0.208550923	0.676301294
ENSGALG00010004814	0.957656159	0.32259993
ENSGALG00010004815	0.245771723	0.02202522
ENSGALG00010004816	0.560372776	0.165713112
ENSGALG00010004818	0.92775089	0.305666183
ENSGALG00010004820	0.822944788	0.266446326
ENSGALG00010004824	0.910859896	0.329287482
ENSGALG00010004828	0.975535688	0.344298272
ENSGALG00010004829	0.267742878	0.132359092
ENSGALG00010004831	0.415822078	0.214975165
ENSGALG00010004832	0.760803066	0.251351027
ENSGALG00010004833	0.736122136	0.251358793
ENSGALG00010004834	0.379796638	0.33436092
ENSGALG00010004835	0.965680842	0.430444218
ENSGALG00010004836	0.856075252	0.288784831
ENSGALG00010004837	0.855041461	0.184331768

ENSGALG00010004839	0.931237283	0.384158608
ENSGALG00010004841	0.894670559	0.43901988
ENSGALG00010004842	0.858967155	0.285108319
ENSGALG00010004843	0.558290571	0.053294879
ENSGALG00010004844	0.863534948	0.178735436
ENSGALG00010004846	0.238704914	0.066047917
ENSGALG00010004847	0.761399226	0.248579497
ENSGALG00010004851	0.37671603	0.150307264
ENSGALG00010004852	0.989418546	0.3373045
ENSGALG00010004854	0.840682177	0.274339409
ENSGALG00010004855	0.740127227	0.295417344
ENSGALG00010004856	0.831553397	0.258450872
ENSGALG00010004857	0.374383337	0.346057116
ENSGALG00010004858	0.050564	0.120729262
ENSGALG00010004859	0.808393394	0.181571408
ENSGALG00010004860	0.311930858	0.122131344
ENSGALG00010004861	0.237451044	0.160483973
ENSGALG00010004862	0.074340167	0.008928453
ENSGALG00010004863	0.3167141	0.302101562
ENSGALG00010004866	0.930371337	0.329722789
ENSGALG00010004868	0.05361597	0.266519371
ENSGALG00010004869	0.153528447	0.240772581
ENSGALG00010004871	0.717240925	0.301798695
ENSGALG00010004872	0.925222232	0.292633125
ENSGALG00010004873	0.482970746	0.244510928
ENSGALG00010004874	0.229904968	0.381618699
ENSGALG00010004875	0.271701261	0.072417625
ENSGALG00010004876	0.258739835	0.239614589
ENSGALG00010004878	0.311995916	0.42508406
ENSGALG00010004879	0.234714087	0.219627023
ENSGALG00010004880	0.890792008	0.352935255
ENSGALG00010004881	0.939244184	0.284123868
ENSGALG00010004883	0.653093499	0.304242418
ENSGALG00010004885	0.203991683	0.058221925
ENSGALG00010004887	0.93586907	0.52519552
ENSGALG00010004888	0.997037599	0.336646308
ENSGALG00010004890	0.906185018	0.30707695
ENSGALG00010004891	0.067227222	0.000474485
ENSGALG00010004892	0.358494397	0.321372695
ENSGALG00010004893	0.126006572	0.365736207
ENSGALG00010004896	0.577076245	0.263203216
ENSGALG00010004898	0.003906436	0.050339809
ENSGALG00010004899	0.566172846	0.49794575
ENSGALG00010004901	0.916078023	0.251222185
ENSGALG00010004902	0.856245945	0.186019443
ENSGALG00010004903	0.846923093	0.346510892
ENSGALG00010004906	0.271113979	0.203747319
ENSGALG00010004907	0.327852322	0.09993466
ENSGALG00010004908	0.529627058	0.254444218
ENSGALG00010004909	0.216838333	0.540217534
ENSGALG00010004911	0.399452143	0.144662594
ENSGALG00010004915	0.24652834	0.309853164
ENSGALG00010004917	0.787656112	0.437959878
ENSGALG00010004919	0.394927857	0.119113854
ENSGALG00010004920	0.264239718	0.073427965
ENSGALG00010004921	0.996196805	0.305145258

ENSGALG00010004924	0.642980539	0.389350874
ENSGALG00010004925	0.271701261	0.072417625
ENSGALG00010004926	0.271701261	0.072417625
ENSGALG00010004927	0.60024376	0.074081407
ENSGALG00010004928	0.933657176	0.345674375
ENSGALG00010004929	0.951776428	0.309618052
ENSGALG00010004931	0.385129618	0.120226607
ENSGALG00010004932	0.032597739	0.058748633
ENSGALG00010004933	0.944024122	0.419793051
ENSGALG00010004934	0.818248647	0.22639127
ENSGALG00010004935	0.908226595	0.26366675
ENSGALG00010004936	0.018241221	0.116736502
ENSGALG00010004938	0.861185719	0.452206362
ENSGALG00010004939	0.443606845	0.247535095
ENSGALG00010004942	0.073376936	0.164024345
ENSGALG00010004943	0.118295041	0.169720193
ENSGALG00010004944	0.908882761	0.20738007
ENSGALG00010004946	0.828447396	0.241713177
ENSGALG00010004948	0.105093336	0.322143658
ENSGALG00010004949	0.571762237	0.305823579
ENSGALG00010004950	0.922611571	0.251471642
ENSGALG00010004951	0.973126459	0.242330548
ENSGALG00010004952	0.686365482	0.430508287
ENSGALG00010004955	0.843102097	0.281963975
ENSGALG00010004956	0.842077321	0.397341619
ENSGALG00010004957	0.031419822	0.159167307
ENSGALG00010004958	0.293752397	0.302284688
ENSGALG00010004962	0.986346974	0.330662035
ENSGALG00010004963	0.523545303	0.124025053
ENSGALG00010004964	0.81670734	0.237078319
ENSGALG00010004965	0.239731232	0.049282697
ENSGALG00010004966	0.595934187	0.436428829
ENSGALG00010004967	0.810743814	0.206359339
ENSGALG00010004968	0.787025525	0.05344297
ENSGALG00010004969	0.92261686	0.467579159
ENSGALG00010004970	0.361049494	0.315369367
ENSGALG00010004971	0.796308999	0.229562668
ENSGALG00010004972	0.53673469	0.060369424
ENSGALG00010004973	0.996181356	0.333693407
ENSGALG00010004974	0.757864363	0.368526941
ENSGALG00010004975	0.513938491	0.007833986
ENSGALG00010004976	0.97148374	0.301343358
ENSGALG00010004977	0.412486142	0.321031476
ENSGALG00010004978	0.196680351	0.261371719
ENSGALG00010004979	0.489227462	0.026969251
ENSGALG00010004980	0.735529463	0.420582325
ENSGALG00010004981	0.982863234	0.26959325
ENSGALG00010004983	0.071781537	0.333510976
ENSGALG00010004984	0.383211517	0.152712488
ENSGALG00010004985	0.992020783	0.333477536
ENSGALG00010004987	0.961333202	0.319989039
ENSGALG00010004988	0.709040204	0.366823496
ENSGALG00010004990	0.997217498	0.336662203
ENSGALG00010004991	0.883963596	0.050849823
ENSGALG00010004992	0.061980386	0.117694959
ENSGALG00010004993	0.954988449	0.40018935

ENSGALG00010004994	0.89238695	0.440526844
ENSGALG00010004995	0.99607337	0.338921295
ENSGALG00010004996	0.766602281	0.454341536
ENSGALG00010004997	0.655034237	0.042143801
ENSGALG00010004998	0.538004959	0.596853774
ENSGALG00010004999	0.772772995	0.057488562
ENSGALG00010005000	0.311289807	0.13851383
ENSGALG00010005001	0.281356228	0.317815845
ENSGALG00010005003	0.993738475	0.354357431
ENSGALG00010005004	0.953793137	0.263312174
ENSGALG00010005005	0.924803138	0.433028778
ENSGALG00010005007	0.988024161	0.335906241
ENSGALG00010005008	0.991070715	0.331391362
ENSGALG00010005010	0.681734411	0.106947156
ENSGALG00010005011	0.992811915	0.334879594
ENSGALG00010005012	0.935840413	0.485688418
ENSGALG00010005014	0.885498839	0.277619359
ENSGALG00010005015	0.496115786	0.179724563
ENSGALG00010005016	0.64812744	0.359031893
ENSGALG00010005017	0.97367585	0.311226714
ENSGALG00010005023	0.894642931	0.374615869
ENSGALG00010005025	0.934824696	0.211328396
ENSGALG00010005026	0.097303109	0.158797063
ENSGALG00010005027	0.919409889	0.122358973
ENSGALG00010005029	0.538324164	0.069879147
ENSGALG00010005030	0.389066621	0.118811063
ENSGALG00010005033	0.935742131	0.28400458
ENSGALG00010005035	0.834628519	0.137963931
ENSGALG00010005037	0.900098572	0.296460547
ENSGALG00010005038	0.64505398	0.386211765
ENSGALG00010005039	0.218741569	0.190475163
ENSGALG00010005042	0.948730135	0.406132192
ENSGALG00010005043	0.281734348	0.241069156
ENSGALG00010005044	0.314454044	0.050770401
ENSGALG00010005045	0.912342748	0.284645539
ENSGALG00010005047	0.368754444	0.215037581
ENSGALG00010005048	0.989044477	0.299126591
ENSGALG00010005049	0.729577131	0.462289025
ENSGALG00010005054	0.36961805	0.141256136
ENSGALG00010005055	0.309655755	0.987979031
ENSGALG00010005056	0.461412531	0.359038832
ENSGALG00010005057	0.965453324	0.217362051
ENSGALG00010005059	0.318125278	0.24495276
ENSGALG00010005060	0.945844433	0.256429957
ENSGALG00010005065	0.288981502	0.129643301
ENSGALG00010005066	0.208550923	0.676301294
ENSGALG00010005067	0.879798351	0.411142898
ENSGALG00010005068	0.03905123	0.19025897
ENSGALG00010005070	0.993638264	0.340949363
ENSGALG00010005071	0.474937178	0.127997944
ENSGALG00010005074	0.232556738	0.155960133
ENSGALG00010005075	0.992582522	0.329129347
ENSGALG00010005077	0.942846699	0.231466575
ENSGALG00010005078	0.725649145	0.03548861
ENSGALG00010005081	0.968865038	0.313976076
ENSGALG00010005082	0.623533981	0.141022664

ENSGALG00010005087	0.816709826	0.361366931
ENSGALG00010005089	0.469200733	0.033817945
ENSGALG00010005091	0.377042884	0.132366957
ENSGALG00010005092	0.972842589	0.298999635
ENSGALG00010005094	0.92050264	0.3524121
ENSGALG00010005098	0.051606007	0.145348651
ENSGALG00010005102	0.080299204	0.172274854
ENSGALG00010005104	0.965263158	0.291604322
ENSGALG00010005105	0.763720975	0.239294538
ENSGALG00010005107	0.858849377	0.239827177
ENSGALG00010005109	0.944184347	0.330036581
ENSGALG00010005110	0.466796891	0.167433006
ENSGALG00010005111	0.949626526	0.471235323
ENSGALG00010005114	0.972829538	0.310834753
ENSGALG00010005116	0.505026024	0.219416157
ENSGALG00010005118	0.922385647	0.212460167
ENSGALG00010005119	0.865117782	0.376592824
ENSGALG00010005120	0.485483307	0.218572055
ENSGALG00010005122	0.431214145	0.153450729
ENSGALG00010005123	0.981835078	0.329742962
ENSGALG00010005124	0.389047419	0.16978754
ENSGALG00010005125	0.445399567	0.012857239
ENSGALG00010005126	0.886881923	0.395489898
ENSGALG00010005127	0.976861834	0.283390849
ENSGALG00010005128	0.39144912	0.115895902
ENSGALG00010005130	0.522993441	0.138952057
ENSGALG00010005131	0.972573222	0.260859511
ENSGALG00010005132	0.704071035	0.263464689
ENSGALG00010005140	0.901394401	0.297012513
ENSGALG00010005141	0.172449586	0.077272984
ENSGALG00010005143	0.762131146	0.027792577
ENSGALG00010005145	0.984470688	0.328458763
ENSGALG00010005146	0.959895761	0.291419781
ENSGALG00010005148	0.82917662	0.523740647
ENSGALG00010005149	0.003921305	0.249095559
ENSGALG00010005150	0.566867179	0.335817644
ENSGALG00010005151	0.378022522	0.12438851
ENSGALG00010005152	0.251259112	0.234738059
ENSGALG00010005154	0.667675483	0.125359552
ENSGALG00010005157	0.767918663	0.320605349
ENSGALG00010005158	0.944981329	0.328198901
ENSGALG00010005159	0.937971762	0.283172079
ENSGALG00010005160	0.431245048	0.315691019
ENSGALG00010005162	0.125880024	0.205619727
ENSGALG00010005163	0.373234341	0.177459231
ENSGALG00010005164	0.218496484	0.2357475
ENSGALG00010005165	0.980902774	0.286360141
ENSGALG00010005166	0.79650625	0.082726199
ENSGALG00010005167	0.081651617	0.049902913
ENSGALG00010005170	0.676268157	0.194057163
ENSGALG00010005171	0.699696558	0.42205719
ENSGALG00010005172	0.928540405	0.331446335
ENSGALG00010005173	0.073404375	0.575118526
ENSGALG00010005174	0.971184689	0.313782843
ENSGALG00010005176	0.982036056	0.423942711
ENSGALG00010005177	0.903555854	0.302517082

ENSGALG00010005178	0.051894818	0.262579457
ENSGALG00010005179	0.562488715	0.211240742
ENSGALG00010005180	0.81879065	0.24982751
ENSGALG00010005181	0.685979028	0.109933597
ENSGALG00010005182	0.341478699	0.209601186
ENSGALG00010005184	0.642196575	0.24997015
ENSGALG00010005185	0.843889951	0.236207376
ENSGALG00010005186	0.955902659	0.323862955
ENSGALG00010005187	0.307713	0.294125534
ENSGALG00010005188	0.612726709	0.048169163
ENSGALG00010005189	0.643537803	0.096199799
ENSGALG00010005190	0.737928588	0.126967201
ENSGALG00010005191	0.835825494	0.349596692
ENSGALG00010005192	0.78300019	0.112414139
ENSGALG00010005193	0.619077496	0.389424888
ENSGALG00010005194	0.573746996	0.195578938
ENSGALG00010005195	0.676568007	0.265320462
ENSGALG00010005197	0.59406642	0.166454434
ENSGALG00010005198	0.353738159	0.15397171
ENSGALG00010005199	0.997260081	0.336351736
ENSGALG00010005200	0.744629188	0.235307353
ENSGALG00010005201	0.489069206	0.169905043
ENSGALG00010005202	0.243417503	0.31053392
ENSGALG00010005203	0.912606687	0.228682395
ENSGALG00010005204	0.660464968	0.51833889
ENSGALG00010005206	0.370622167	0.730060946
ENSGALG00010005208	0.881508178	0.175951884
ENSGALG00010005209	0.621326583	0.326420628
ENSGALG00010005210	0.719828229	0.29939544
ENSGALG00010005211	0.4463046	0.152148195
ENSGALG00010005212	0.725691292	0.080657886
ENSGALG00010005213	0.784772795	0.324730227
ENSGALG00010005214	0.269310992	0.099788613
ENSGALG00010005215	0.911095544	0.215972216
ENSGALG00010005216	0.564738715	0.028410167
ENSGALG00010005217	0.459333435	0.263238616
ENSGALG00010005218	0.566408275	0.321741097
ENSGALG00010005219	0.647911413	0.350599433
ENSGALG00010005220	0.597544665	0.108515763
ENSGALG00010005221	0.941561195	0.311428757
ENSGALG00010005222	0.989530915	0.336109853
ENSGALG00010005223	0.580108305	0.040586496
ENSGALG00010005224	0.678782611	0.227370099
ENSGALG00010005226	0.895802339	0.31647642
ENSGALG00010005227	0.878703395	0.34964241
ENSGALG00010005228	0.647482362	0.424029727
ENSGALG00010005229	0.560170443	0.187211213
ENSGALG00010005231	0.679628435	0.036683545
ENSGALG00010005232	0.819522866	0.37312732
ENSGALG00010005233	0.419623546	0.557771527
ENSGALG00010005236	0.799222648	0.391965621
ENSGALG00010005237	0.762981243	0.184856224
ENSGALG00010005238	0.930915504	0.311877557
ENSGALG00010005239	0.928115552	0.32192057
ENSGALG00010005240	0.113349306	0.226412826
ENSGALG00010005241	0.955611001	0.268814401

ENSGALG00010005242	0.947210674	0.394864653
ENSGALG00010005243	0.030798317	0.351380107
ENSGALG00010005244	0.63863652	0.477720478
ENSGALG00010005245	0.948181378	0.362094619
ENSGALG00010005246	0.858208548	0.314941576
ENSGALG00010005247	0.773946538	0.349851281
ENSGALG00010005248	0.860618002	0.2798435
ENSGALG00010005249	0.961227447	0.369544553
ENSGALG00010005250	0.958499348	0.42244381
ENSGALG00010005251	0.052545293	0.138312166
ENSGALG00010005252	0.941272003	0.213445491
ENSGALG00010005253	0.475243611	0.358195905
ENSGALG00010005254	0.594998295	0.306261579
ENSGALG00010005255	0.996534916	0.301095703
ENSGALG00010005256	0.744100566	0.243016325
ENSGALG00010005257	0.753907094	0.215494126
ENSGALG00010005258	0.444724743	0.060021854
ENSGALG00010005259	0.979417708	0.357896985
ENSGALG00010005260	0.974748175	0.250636201
ENSGALG00010005261	0.348799145	0.040260592
ENSGALG00010005262	0.953514751	0.418598734
ENSGALG00010005263	0.674947555	0.275621772
ENSGALG00010005264	0.99561496	0.32699766
ENSGALG00010005265	0.699630301	0.27445769
ENSGALG00010005266	0.781489041	0.264598873
ENSGALG00010005267	0.949138691	0.306101448
ENSGALG00010005268	0.983724059	0.344396328
ENSGALG00010005269	0.996334993	0.341659489
ENSGALG00010005270	0.961580168	0.265958762
ENSGALG00010005271	0.647720279	0.198313355
ENSGALG00010005272	0.956988143	0.324367265
ENSGALG00010005273	0.767290434	0.170026813
ENSGALG00010005274	0.804109369	0.304150692
ENSGALG00010005275	0.630601896	0.21667994
ENSGALG00010005276	0.396668506	0.181671381
ENSGALG00010005277	0.916223179	0.263610609
ENSGALG00010005278	0.551769667	0.120913483
ENSGALG00010005279	0.792590588	0.229760271
ENSGALG00010005280	0.050564	0.120729262
ENSGALG00010005281	0.876759014	0.428036709
ENSGALG00010005282	0.614730899	0.052177339
ENSGALG00010005283	0.856541634	0.266545848
ENSGALG00010005284	0.534976018	0.296709269
ENSGALG00010005285	0.908139504	0.246570428
ENSGALG00010005286	0.333361666	0.242868776
ENSGALG00010005287	0.068703172	0.161634517
ENSGALG00010005288	0.078489461	0.081967578
ENSGALG00010005290	0.669366594	0.392789746
ENSGALG00010005291	0.981904398	0.320088319
ENSGALG00010005292	0.921388277	0.420503334
ENSGALG00010005293	0.950998526	0.247491043
ENSGALG00010005294	0.054343211	0.202346398
ENSGALG00010005295	0.633236512	0.265609694
ENSGALG00010005296	0.891858939	0.379584369
ENSGALG00010005297	0.914904933	0.341837359
ENSGALG00010005298	0.82433745	0.316038466

ENSGALG00010005299	0.285513734	0.24313624
ENSGALG00010005300	0.385376427	0.119512257
ENSGALG00010005301	0.441573517	0.414688391
ENSGALG00010005302	0.793341391	0.308676516
ENSGALG00010005303	0.766288009	0.151639653
ENSGALG00010005304	0.681188867	0.121580011
ENSGALG00010005305	0.892107251	0.331564228
ENSGALG00010005306	0.885512372	0.24357376
ENSGALG00010005307	0.45545821	0.216724458
ENSGALG00010005308	0.655908444	0.258819716
ENSGALG00010005309	0.768453274	0.329109875
ENSGALG00010005310	0.830484504	0.261220371
ENSGALG00010005311	0.855544503	0.364903643
ENSGALG00010005312	0.49646521	0.048859621
ENSGALG00010005313	0.906665914	0.522767783
ENSGALG00010005314	0.992004286	0.299727743
ENSGALG00010005315	0.72050549	0.166964879
ENSGALG00010005316	0.845287337	0.405042769
ENSGALG00010005317	0.914706738	0.324900577
ENSGALG00010005318	0.414147668	0.085536883
ENSGALG00010005319	0.381608622	0.094262957
ENSGALG00010005320	0.852350785	0.091455654
ENSGALG00010005321	0.750643739	0.626231772
ENSGALG00010005322	0.369879257	0.006047186
ENSGALG00010005323	0.598021733	0.249182215
ENSGALG00010005324	0.454000726	0.009048199
ENSGALG00010005326	0.478051733	0.062151187
ENSGALG00010005328	0.457160252	0.114267555
ENSGALG00010005329	0.873624921	0.353719528
ENSGALG00010005330	0.839230805	0.39981102
ENSGALG00010005332	0.653383458	0.249659553
ENSGALG00010005333	0.846732755	0.282913344
ENSGALG00010005334	0.631049184	0.034362807
ENSGALG00010005335	0.956871316	0.277770451
ENSGALG00010005336	0.940518553	0.41164813
ENSGALG00010005338	0.721732976	0.267652543
ENSGALG00010005340	0.279834744	0.382907809
ENSGALG00010005341	0.869385879	0.465907346
ENSGALG00010005342	0.855617827	0.294717196
ENSGALG00010005344	0.441581065	0.208199336
ENSGALG00010005345	0.337616669	0.113534786
ENSGALG00010005346	0.894016229	0.174856124
ENSGALG00010005347	0.919114727	0.32595725
ENSGALG00010005348	0.832455513	0.443649825
ENSGALG00010005349	0.894050226	0.306967766
ENSGALG00010005350	0.112822493	0.03666474
ENSGALG00010005351	0.986892437	0.297766597
ENSGALG00010005352	0.876543908	0.440225401
ENSGALG00010005353	0.968204022	0.273931206
ENSGALG00010005354	0.861474633	0.317371034
ENSGALG00010005355	0.961812664	0.422227829
ENSGALG00010005356	0.779054561	0.325371069
ENSGALG00010005357	0.881650056	0.327814332
ENSGALG00010005358	0.894923444	0.318861496
ENSGALG00010005359	0.840184367	0.143961305
ENSGALG00010005360	0.937167497	0.399707634

ENSGALG00010005361	0.765104514	0.552504583
ENSGALG00010005362	0.019418185	0.206183982
ENSGALG00010005363	0.875522342	0.253147535
ENSGALG00010005364	0.981900039	0.345690909
ENSGALG00010005365	0.054289971	0.14989198
ENSGALG00010005366	0.24149414	0.076962543
ENSGALG00010005367	0.920938299	0.273347911
ENSGALG00010005368	0.913822824	0.172389073
ENSGALG00010005369	0.825296099	0.424952485
ENSGALG00010005370	0.594066093	0.31349955
ENSGALG00010005372	0.602122235	0.116553506
ENSGALG00010005373	0.285188268	0.121935808
ENSGALG00010005374	0.492325412	0.18165357
ENSGALG00010005375	0.127856628	0.013601694
ENSGALG00010005376	0.853684118	0.276268618
ENSGALG00010005377	0.953679061	0.375437072
ENSGALG00010005378	0.880732589	0.367747057
ENSGALG00010005379	0.20055635	0.015073068
ENSGALG00010005380	0.926718729	0.332908538
ENSGALG00010005381	0.077417792	0.027564508
ENSGALG00010005382	0.953697392	0.271720169
ENSGALG00010005383	0.978642357	0.310747449
ENSGALG00010005384	0.98261663	0.402154258
ENSGALG00010005385	0.080413327	0.10220662
ENSGALG00010005386	0.673744982	0.237127041
ENSGALG00010005387	0.625680907	0.24351295
ENSGALG00010005388	0.870967245	0.276923233
ENSGALG00010005389	0.848039329	0.215761186
ENSGALG00010005390	0.229451174	0.308991893
ENSGALG00010005391	0.788065329	0.557269037
ENSGALG00010005392	0.960989041	0.264017683
ENSGALG00010005393	0.927796522	0.279519461
ENSGALG00010005394	0.323923084	0.022782276
ENSGALG00010005395	0.941404249	0.359003395
ENSGALG00010005396	0.8630785	0.123276309
ENSGALG00010005397	0.708686073	0.386139568
ENSGALG00010005398	0.888093781	0.146752821
ENSGALG00010005399	0.354198248	0.300816078
ENSGALG00010005400	0.524634497	0.016092084
ENSGALG00010005401	0.941704533	0.322012905
ENSGALG00010005402	0.765029162	0.289179171
ENSGALG00010005403	0.199110738	0.004108869
ENSGALG00010005404	0.819340698	0.328530426
ENSGALG00010005405	0.599200738	0.209242185
ENSGALG00010005406	0.114406287	0.312344548
ENSGALG00010005407	0.694680469	0.254915639
ENSGALG00010005408	0.931338129	0.29521449
ENSGALG00010005409	0.983547607	0.303646315
ENSGALG00010005410	0.234180613	0.13494038
ENSGALG00010005411	0.139352043	0.321824924
ENSGALG00010005412	0.300241627	0.570926791
ENSGALG00010005413	0.25387471	0.14445058
ENSGALG00010005414	0.272006816	0.288372799
ENSGALG00010005415	0.850650345	0.5203502
ENSGALG00010005416	0.98399732	0.310612635
ENSGALG00010005417	0.902896069	0.36694735

ENSGALG00010005418	0.609409188	0.150921349
ENSGALG00010005419	0.936029666	0.278059122
ENSGALG00010005420	0.864434114	0.322774607
ENSGALG00010005421	0.844892436	0.315570748
ENSGALG00010005422	0.676478816	0.361322719
ENSGALG00010005423	0.982996126	0.350330527
ENSGALG00010005424	0.969344418	0.312622577
ENSGALG00010005425	0.925133561	0.385702329
ENSGALG00010005426	0.85564399	0.172033778
ENSGALG00010005427	0.956104639	0.354091958
ENSGALG00010005428	0.22285664	0.257372557
ENSGALG00010005429	0.887142738	0.315445961
ENSGALG00010005430	0.116807251	0.060277794
ENSGALG00010005431	0.078311903	0.375121149
ENSGALG00010005432	0.137667049	0.280594582
ENSGALG00010005433	0.910170746	0.16410947
ENSGALG00010005434	0.217386736	0.147437101
ENSGALG00010005435	0.495297326	0.006843784
ENSGALG00010005436	0.795639965	0.468247134
ENSGALG00010005437	0.981702318	0.370809791
ENSGALG00010005438	0.786925702	0.284773268
ENSGALG00010005439	0.525297352	0.067449141
ENSGALG00010005440	0.717565814	0.1253265
ENSGALG00010005441	0.973369404	0.261769605
ENSGALG00010005442	0.806316823	0.202879775
ENSGALG00010005443	0.732865412	0.138269862
ENSGALG00010005444	0.98318568	0.376944435
ENSGALG00010005445	0.990758295	0.345408511
ENSGALG00010005446	0.423517453	0.213001248
ENSGALG00010005447	0.535739628	0.241621526
ENSGALG00010005448	0.971474995	0.31394628
ENSGALG00010005449	0.905801918	0.360906373
ENSGALG00010005450	0.852142719	0.221568861
ENSGALG00010005452	0.876784176	0.354899468
ENSGALG00010005453	0.796328743	0.051801114
ENSGALG00010005454	0.236364906	0.543169529
ENSGALG00010005455	0.879640251	0.340822712
ENSGALG00010005456	0.706047603	0.352091944
ENSGALG00010005457	0.234124422	0.241621045
ENSGALG00010005458	0.158747102	0.01655308
ENSGALG00010005459	0.398334376	0.151074837
ENSGALG00010005460	0.978540703	0.367333778
ENSGALG00010005461	0.217013836	0.193799607
ENSGALG00010005462	0.466491776	0.001914512
ENSGALG00010005463	0.713743737	0.321493611
ENSGALG00010005464	0.98901332	0.302295735
ENSGALG00010005465	0.031969973	0.155291033
ENSGALG00010005466	0.793765562	0.290956327
ENSGALG00010005467	0.964745725	0.280006094
ENSGALG00010005468	0.883418534	0.441278993
ENSGALG00010005469	0.039849486	0.174398509
ENSGALG00010005470	0.846518338	0.523180354
ENSGALG00010005471	0.647474012	0.24304038
ENSGALG00010005472	0.272510887	0.024748777
ENSGALG00010005473	0.292462411	0.020335823
ENSGALG00010005474	0.190972861	0.412756245

ENSGALG00010005475	0.981885698	0.292488591
ENSGALG00010005476	0.098088661	0.454318358
ENSGALG00010005477	0.888870221	0.183366834
ENSGALG00010005478	0.979019383	0.37345942
ENSGALG00010005479	0.55481494	0.17434458
ENSGALG00010005480	0.647295531	0.285801076
ENSGALG00010005481	0.257102694	0.137180099
ENSGALG00010005482	0.247350771	0.241123882
ENSGALG00010005483	0.219563083	0.229706133
ENSGALG00010005484	0.963460137	0.360807821
ENSGALG00010005485	0.467903358	0.338853674
ENSGALG00010005486	0.896831293	0.349982471
ENSGALG00010005487	0.862410947	0.291739761
ENSGALG00010005488	0.856927229	0.464019617
ENSGALG00010005489	0.813184718	0.061795886
ENSGALG00010005490	0.823787534	0.259539648
ENSGALG00010005491	0.949287619	0.290355404
ENSGALG00010005492	0.987128089	0.339043122
ENSGALG00010005493	0.301742166	0.414259336
ENSGALG00010005494	0.986056045	0.353384917
ENSGALG00010005495	0.94758736	0.206134736
ENSGALG00010005496	0.341056803	0.114817382
ENSGALG00010005497	0.85425295	0.31110155
ENSGALG00010005498	0.982103366	0.321211675
ENSGALG00010005499	0.640081355	0.369907805
ENSGALG00010005500	0.540057705	0.49526768
ENSGALG00010005501	0.979858615	0.334628691
ENSGALG00010005502	0.824283975	0.446624232
ENSGALG00010005503	0.659752926	0.090135322
ENSGALG00010005504	0.959111281	0.36047214
ENSGALG00010005505	0.247363671	0.263819475
ENSGALG00010005506	0.662169108	0.286248679
ENSGALG00010005507	0.836163239	0.337545776
ENSGALG00010005508	0.966090153	0.231421068
ENSGALG00010005509	0.990189568	0.330153652
ENSGALG00010005510	0.976162688	0.385754984
ENSGALG00010005511	0.96491049	0.411775748
ENSGALG00010005512	0.89490161	0.473798038
ENSGALG00010005513	0.920781244	0.302982245
ENSGALG00010005514	0.986660468	0.303227959
ENSGALG00010005515	0.884298313	0.255381127
ENSGALG00010005516	0.453602912	0.017072357
ENSGALG00010005517	0.723669342	0.321387166
ENSGALG00010005518	0.985095918	0.354962488
ENSGALG00010005519	0.439976527	0.070533118
ENSGALG00010005520	0.284227058	0.103920777
ENSGALG00010005521	0.929300458	0.426899277
ENSGALG00010005522	0.989935771	0.340853549
ENSGALG00010005523	0.683096062	0.202273709
ENSGALG00010005524	0.954468375	0.280698448
ENSGALG00010005526	0.966626135	0.297100482
ENSGALG00010005527	0.674385302	0.191755791
ENSGALG00010005529	0.667530828	0.321133204
ENSGALG00010005530	0.972071117	0.326374182
ENSGALG00010005531	0.959654298	0.290067221
ENSGALG00010005532	0.787053903	0.16101926

ENSGALG00010005533	0.699468249	0.313055403
ENSGALG00010005534	0.965827276	0.292507209
ENSGALG00010005535	0.91545416	0.240681666
ENSGALG00010005536	0.339465031	0.216660977
ENSGALG00010005537	0.857813753	0.251285682
ENSGALG00010005538	0.586719176	0.271806719
ENSGALG00010005539	0.521720198	0.252281033
ENSGALG00010005540	0.994047896	0.323695065
ENSGALG00010005541	0.654372905	0.156421285
ENSGALG00010005542	0.651459421	0.236593159
ENSGALG00010005543	0.926196199	0.267846421
ENSGALG00010005544	0.989405268	0.326828763
ENSGALG00010005545	0.92172622	0.252897434
ENSGALG00010005546	0.983459132	0.311159888
ENSGALG00010005547	0.889939873	0.469711805
ENSGALG00010005548	0.555682581	0.022625516
ENSGALG00010005549	0.955379287	0.326579382
ENSGALG00010005550	0.734158342	0.247671716
ENSGALG00010005551	0.16811112	0.346045403
ENSGALG00010005552	0.844417309	0.415210988
ENSGALG00010005553	0.990682039	0.301857327
ENSGALG00010005554	0.097545227	0.139654523
ENSGALG00010005555	0.42035714	0.209949832
ENSGALG00010005556	0.995990248	0.336475553
ENSGALG00010005557	0.754736875	0.1401165
ENSGALG00010005558	0.84816026	0.178077538
ENSGALG00010005559	0.961477111	0.285647053
ENSGALG00010005560	0.773531776	0.286528187
ENSGALG00010005561	0.622339285	0.065100742
ENSGALG00010005562	0.737330584	0.180875506
ENSGALG00010005564	0.983493747	0.36175527
ENSGALG00010005565	0.977183384	0.251694196
ENSGALG00010005566	0.015922281	0.12649853
ENSGALG00010005567	0.6819521	0.548693021
ENSGALG00010005568	0.632701716	0.02523601
ENSGALG00010005571	0.265540773	0.258640441
ENSGALG00010005572	0.986283282	0.348933301
ENSGALG00010005573	0.927120836	0.319532236
ENSGALG00010005574	0.991745728	0.333868878
ENSGALG00010005575	0.597415573	0.128687172
ENSGALG00010005578	0.064553866	0.046527695
ENSGALG00010005581	0.044418695	0.451480948
ENSGALG00010005582	0.214288333	0.164706724
ENSGALG00010005583	0.989729831	0.329375614
ENSGALG00010005585	0.880899382	0.334435138
ENSGALG00010005587	0.475576833	0.295967887
ENSGALG00010005588	0.008178884	0.225908097
ENSGALG00010005590	0.30132941	0.077999125
ENSGALG00010005593	0.708508783	0.233166817
ENSGALG00010005594	0.966825767	0.273739547
ENSGALG00010005596	0.946734838	0.284558538
ENSGALG00010005597	0.712836903	0.23293836
ENSGALG00010005600	0.901147643	0.349391302
ENSGALG00010005601	0.348997163	0.18871309
ENSGALG00010005602	0.299722474	0.126577591
ENSGALG00010005604	0.865182059	0.317793863

ENSGALG00010005605	0.180499089	0.190324653
ENSGALG00010005608	0.461005186	0.187426455
ENSGALG00010005609	0.781481179	0.348314678
ENSGALG00010005610	0.046603161	0.319673736
ENSGALG00010005612	0.591651591	0.197429027
ENSGALG00010005613	0.651805195	0.251364429
ENSGALG00010005617	0.092581875	0.049231578
ENSGALG00010005618	0.08305758	0.029370107
ENSGALG00010005619	0.85327717	0.231830808
ENSGALG00010005621	0.758270424	0.025062405
ENSGALG00010005624	0.292709036	0.327963953
ENSGALG00010005629	0.148955122	0.296952365
ENSGALG00010005630	0.936438436	0.245193128
ENSGALG00010005631	0.59648293	0.195715183
ENSGALG00010005632	0.671437704	0.407591201
ENSGALG00010005634	0.913011927	0.350166478
ENSGALG00010005636	0.554110885	0.40856543
ENSGALG00010005637	0.437129415	0.239904049
ENSGALG00010005639	0.266137114	0.107784983
ENSGALG00010005641	0.734242176	0.104776545
ENSGALG00010005645	0.967793081	0.236081198
ENSGALG00010005649	0.595875915	0.389124502
ENSGALG00010005650	0.577256167	0.173504072
ENSGALG00010005651	0.817842349	0.053112203
ENSGALG00010005656	0.976985141	0.298853917
ENSGALG00010005658	0.009812001	0.5166245
ENSGALG00010005659	0.578772447	0.155042686
ENSGALG00010005660	0.951857356	0.342680626
ENSGALG00010005664	0.653197339	0.136806212
ENSGALG00010005665	0.896024454	0.32372595
ENSGALG00010005666	0.207235825	0.237121127
ENSGALG00010005667	0.277566769	0.107857127
ENSGALG00010005668	0.451280892	0.147355642
ENSGALG00010005670	0.932077759	0.286716332
ENSGALG00010005672	0.788797428	0.213787969
ENSGALG00010005673	0.779723691	0.284200727
ENSGALG00010005674	0.818724459	0.387027169
ENSGALG00010005675	0.798991511	0.038998737
ENSGALG00010005676	0.65325985	0.181296045
ENSGALG00010005677	0.472681523	0.144293074
ENSGALG00010005678	0.855081866	0.282532408
ENSGALG00010005679	0.51461622	0.355074395
ENSGALG00010005680	0.67000677	0.260718086
ENSGALG00010005681	0.013239249	0.160953236
ENSGALG00010005682	0.48171383	0.64825227
ENSGALG00010005683	0.632843377	0.132522461
ENSGALG00010005684	0.37984072	0.108385122
ENSGALG00010005686	0.814753173	0.272769261
ENSGALG00010005688	0.832749871	0.221790294
ENSGALG00010005689	0.876158238	0.41684929
ENSGALG00010005691	0.59008763	0.190354068
ENSGALG00010005692	0.16571413	0.064643912
ENSGALG00010005693	0.931565742	0.26463072
ENSGALG00010005694	0.940765493	0.107131484
ENSGALG00010005695	0.554864574	0.136202294
ENSGALG00010005696	0.379349827	0.099230569

ENSGALG00010005698	0.382985366	0.1291087
ENSGALG00010005699	0.907930069	0.323307578
ENSGALG00010005700	0.48904219	0.192561417
ENSGALG00010005701	0.064253469	0.10917019
ENSGALG00010005702	0.024374485	0.442743073
ENSGALG00010005704	0.990383142	0.365144562
ENSGALG00010005705	0.12917798	0.468639813
ENSGALG00010005706	0.203991683	0.058221925
ENSGALG00010005708	0.950518469	0.362359193
ENSGALG00010005711	0.598892571	0.174457186
ENSGALG00010005712	0.940024552	0.34013903
ENSGALG00010005714	0.771432784	0.341442311
ENSGALG00010005716	0.845555136	0.403237465
ENSGALG00010005718	0.423838815	0.20565106
ENSGALG00010005720	0.606315696	0.188399197
ENSGALG00010005724	0.861306783	0.286217845
ENSGALG00010005725	0.140920444	0.15530806
ENSGALG00010005726	0.672460482	0.187654448
ENSGALG00010005735	0.100771311	0.166487866
ENSGALG00010005737	0.921446862	0.302608552
ENSGALG00010005739	0.986093867	0.326282035
ENSGALG00010005740	0.873716518	0.173006372
ENSGALG00010005741	0.929592794	0.295114434
ENSGALG00010005742	0.869219613	0.192508054
ENSGALG00010005743	0.339894371	0.217479104
ENSGALG00010005744	0.677640935	0.312680608
ENSGALG00010005746	0.928729915	0.296595248
ENSGALG00010005748	0.961467009	0.216287015
ENSGALG00010005749	0.484242833	0.130180432
ENSGALG00010005752	0.716453694	0.52926132
ENSGALG00010005754	0.633552207	0.362373486
ENSGALG00010005755	0.928077045	0.350938284
ENSGALG00010005756	0.289194983	0.292805799
ENSGALG00010005757	0.919351839	0.307679221
ENSGALG00010005758	0.682249881	0.292503445
ENSGALG00010005759	0.423134054	0.242702369
ENSGALG00010005760	0.527293542	0.201548719
ENSGALG00010005762	0.295081293	0.447565639
ENSGALG00010005763	0.956278607	0.313158551
ENSGALG00010005765	0.528378417	0.039281071
ENSGALG00010005766	0.541033981	0.011729272
ENSGALG00010005767	0.829925237	0.223645557
ENSGALG00010005768	0.49600362	0.262272631
ENSGALG00010005769	0.071572107	0.054231037
ENSGALG00010005771	0.503056376	0.168109625
ENSGALG00010005772	0.944213696	0.374201224
ENSGALG00010005777	0.244713592	0.309089016
ENSGALG00010005778	0.714500037	0.00986036
ENSGALG00010005779	0.667420574	0.253459447
ENSGALG00010005780	0.399574883	0.154317703
ENSGALG00010005781	0.024209848	0.019981473
ENSGALG00010005782	0.238107881	0.178001475
ENSGALG00010005784	0.344430818	0.006237626
ENSGALG00010005785	0.659049579	0.187770345
ENSGALG00010005786	0.66874296	0.277565524
ENSGALG00010005787	0.989273536	0.339824662

ENSGALG00010005789	0.980148535	0.351521197
ENSGALG00010005790	0.754160541	0.161254493
ENSGALG00010005792	0.912702362	0.143679564
ENSGALG00010005793	0.585176285	0.235767836
ENSGALG00010005794	0.569043619	0.197196766
ENSGALG00010005795	0.115460095	0.045732271
ENSGALG00010005796	0.37916021	0.151571387
ENSGALG00010005799	0.894026247	0.257163544
ENSGALG00010005800	0.216080084	0.472879241
ENSGALG00010005802	0.420576684	0.263256979
ENSGALG00010005803	0.188649845	0.076399714
ENSGALG00010005805	0.319990883	0.456214528
ENSGALG00010005809	0.108245516	0.21902547
ENSGALG00010005811	0.426464256	0.079191256
ENSGALG00010005813	0.131752732	0.499944108
ENSGALG00010005814	0.676285181	0.163262051
ENSGALG00010005816	0.238151512	0.247098185
ENSGALG00010005817	0.931803184	0.209652948
ENSGALG00010005818	0.308387909	0.133528019
ENSGALG00010005819	0.190896405	0.512354093
ENSGALG00010005820	0.990723176	0.289806661
ENSGALG00010005821	0.570715239	0.087545795
ENSGALG00010005822	0.805585507	0.403140035
ENSGALG00010005823	0.377716751	0.312152712
ENSGALG00010005824	0.265558089	0.028821141
ENSGALG00010005825	0.40703223	0.350801379
ENSGALG00010005826	0.680073204	0.203942758
ENSGALG00010005827	0.693275535	0.361090318
ENSGALG00010005828	0.851773559	0.23708242
ENSGALG00010005829	0.512737105	0.370984544
ENSGALG00010005830	0.892864122	0.294050845
ENSGALG00010005831	0.972373005	0.354386248
ENSGALG00010005834	0.853027688	0.200174171
ENSGALG00010005836	0.925598423	0.222723
ENSGALG00010005837	0.821191807	0.444551831
ENSGALG00010005839	0.830834531	0.159503504
ENSGALG00010005840	0.557265658	0.188164937
ENSGALG00010005841	0.884221923	0.259364066
ENSGALG00010005842	0.956824147	0.297068198
ENSGALG00010005843	0.141090778	0.013014091
ENSGALG00010005844	0.404167701	0.144831306
ENSGALG00010005845	0.940894715	0.303441459
ENSGALG00010005847	0.761451455	0.274873202
ENSGALG00010005848	0.707092674	0.228027262
ENSGALG00010005849	0.858612926	0.100441505
ENSGALG00010005851	0.762962736	0.289888078
ENSGALG00010005852	0.285188268	0.121935808
ENSGALG00010005853	0.294001538	0.323163917
ENSGALG00010005854	0.208275758	0.224105925
ENSGALG00010005856	0.471475819	0.113760518
ENSGALG00010005858	0.819101925	0.168676385
ENSGALG00010005859	0.688478738	0.193113386
ENSGALG00010005860	0.941139838	0.378225471
ENSGALG00010005861	0.915564172	0.314304004
ENSGALG00010005862	0.914096123	0.291947724
ENSGALG00010005864	0.295794928	0.173016847

ENSGALG00010005865	0.311651474	0.016715479
ENSGALG00010005866	0.956555599	0.253132689
ENSGALG00010005870	0.578958841	0.140949939
ENSGALG00010005871	0.982719147	0.288943979
ENSGALG00010005872	0.75453221	0.251282546
ENSGALG00010005875	0.220746454	0.571592208
ENSGALG00010005876	0.594079286	0.107156912
ENSGALG00010005877	0.043685735	0.101872934
ENSGALG00010005879	0.9750451	0.340478003
ENSGALG00010005880	0.980621404	0.362810447
ENSGALG00010005881	0.974038139	0.30538087
ENSGALG00010005883	0.863154128	0.423966568
ENSGALG00010005884	0.855853209	0.265245142
ENSGALG00010005885	0.073555782	0.186105945
ENSGALG00010005886	0.381023651	0.141101774
ENSGALG00010005887	0.652254434	0.025563928
ENSGALG00010005888	0.066854889	0.147160136
ENSGALG00010005890	0.122938888	0.273125449
ENSGALG00010005891	0.621804415	0.284390924
ENSGALG00010005892	0.507017302	0.107731729
ENSGALG00010005893	0.048870901	0.129966141
ENSGALG00010005895	0.584794524	0.51925059
ENSGALG00010005899	0.033674058	0.198328249
ENSGALG00010005900	0.92997003	0.314837078
ENSGALG00010005901	0.346419837	0.145755448
ENSGALG00010005902	0.774475864	0.115919604
ENSGALG00010005903	0.162806185	0.177050262
ENSGALG00010005904	0.752229308	0.220485996
ENSGALG00010005905	0.292042451	0.213684571
ENSGALG00010005909	0.45846296	0.005801845
ENSGALG00010005910	0.770883105	0.26109568
ENSGALG00010005911	0.785196434	0.533541505
ENSGALG00010005912	0.760306361	0.38477747
ENSGALG00010005913	0.470643018	0.085559462
ENSGALG00010005914	0.866628916	0.28055381
ENSGALG00010005915	0.898927197	0.148522342
ENSGALG00010005916	0.055219348	0.608449815
ENSGALG00010005918	0.000893823	0.500599188
ENSGALG00010005919	0.008134371	0.208550203
ENSGALG00010005920	0.781833653	0.436791605
ENSGALG00010005921	0.362898454	0.02098693
ENSGALG00010005922	0.433960528	0.15553562
ENSGALG00010005923	0.412100413	0.15218604
ENSGALG00010005924	0.454091535	0.393546415
ENSGALG00010005925	0.019583988	0.039177109
ENSGALG00010005926	0.996479738	0.352155392
ENSGALG00010005927	0.285272007	0.109169955
ENSGALG00010005928	0.319989291	0.182147595
ENSGALG00010005929	0.476200491	0.172173364
ENSGALG00010005932	0.587653963	0.187631755
ENSGALG00010005934	0.989309466	0.341185558
ENSGALG00010005935	0.543346011	0.070822976
ENSGALG00010005936	0.219386918	0.079388121
ENSGALG00010005937	0.429529526	0.208598056
ENSGALG00010005938	0.580510709	0.382332307
ENSGALG00010005939	0.342060521	0.10352521

ENSGALG00010005941	0.986477812	0.319062924
ENSGALG00010005942	0.39957569	0.107171429
ENSGALG00010005943	0.245455492	0.198023473
ENSGALG00010005944	0.581051259	0.219875702
ENSGALG00010005949	0.828194759	0.451780898
ENSGALG00010005956	0.856173921	0.518791985
ENSGALG00010005959	0.089765396	0.095692089
ENSGALG00010005961	0.567062391	0.201274118
ENSGALG00010005963	0.459491949	0.098918206
ENSGALG00010005964	0.929876928	0.322379336
ENSGALG00010005965	0.060762054	0.1387881
ENSGALG00010005966	0.94341221	0.359411078
ENSGALG00010005967	0.94098615	0.344283517
ENSGALG00010005968	0.277566769	0.107857127
ENSGALG00010005969	0.901126367	0.185988843
ENSGALG00010005970	0.453857431	0.010409917
ENSGALG00010005972	0.167624301	0.217536576
ENSGALG00010005973	0.018301635	0.519548151
ENSGALG00010005975	0.911773979	0.321989626
ENSGALG00010005977	0.538573827	0.221027742
ENSGALG00010005978	0.093261333	0.117949127
ENSGALG00010005979	0.060595164	0.192953689
ENSGALG00010005980	0.14258792	0.245291929
ENSGALG00010005981	0.848003939	0.180994447
ENSGALG00010005982	0.127989044	0.245086416
ENSGALG00010005983	0.324373729	0.188688025
ENSGALG00010005984	0.989871678	0.337798542
ENSGALG00010005985	0.511502767	0.147233642
ENSGALG00010005988	0.630192272	0.012286493
ENSGALG00010005989	0.460020626	0.15575305
ENSGALG00010005990	0.979636383	0.324029467
ENSGALG00010005991	0.543196991	0.375907256
ENSGALG00010005994	0.58031066	0.206650939
ENSGALG00010005995	0.379004946	0.117491907
ENSGALG00010005997	0.95632506	0.290358581
ENSGALG00010005998	0.932082461	0.208618854
ENSGALG00010005999	0.962153944	0.343873801
ENSGALG00010006000	0.302495553	0.210142421
ENSGALG00010006001	0.933318244	0.331196091
ENSGALG00010006002	0.36703823	0.115008047
ENSGALG00010006003	0.458279308	0.217237622
ENSGALG00010006004	0.556388942	0.331129438
ENSGALG00010006005	0.689168208	0.448696931
ENSGALG00010006006	0.715346457	0.348329841
ENSGALG00010006007	0.153280279	0.127393813
ENSGALG00010006008	0.425892345	0.140604944
ENSGALG00010006009	0.972038005	0.332178038
ENSGALG00010006010	0.92283497	0.200221562
ENSGALG00010006011	0.982586621	0.348724642
ENSGALG00010006012	0.379952917	0.147982463
ENSGALG00010006013	0.65693844	0.13221887
ENSGALG00010006015	0.931414466	0.214015258
ENSGALG00010006016	0.07078608	0.18958579
ENSGALG00010006018	0.053029702	0.24059774
ENSGALG00010006020	0.395956512	0.135331957
ENSGALG00010006021	0.48238223	0.243950173

ENSGALG00010006022	0.625173288	0.06150088
ENSGALG00010006023	0.626647294	0.243991815
ENSGALG00010006024	0.811714262	0.265716933
ENSGALG00010006025	0.901505435	0.170882239
ENSGALG00010006026	0.075982956	0.067327113
ENSGALG00010006028	0.903292815	0.325403485
ENSGALG00010006029	0.874904782	0.227276245
ENSGALG00010006031	0.597016068	0.058703589
ENSGALG00010006032	0.410093186	0.167867886
ENSGALG00010006033	0.222163819	0.072940602
ENSGALG00010006035	0.989838668	0.35473096
ENSGALG00010006036	0.284102763	0.000618082
ENSGALG00010006037	0.973465552	0.399915132
ENSGALG00010006038	0.563375165	0.053772731
ENSGALG00010006039	0.784924959	0.348830946
ENSGALG00010006040	0.07211826	0.164320234
ENSGALG00010006041	0.882826269	0.245720303
ENSGALG00010006042	0.432123421	0.320526545
ENSGALG00010006045	0.192115082	0.095815865
ENSGALG00010006046	0.581450214	0.212540732
ENSGALG00010006047	0.718123403	0.414274534
ENSGALG00010006048	0.734206861	0.108559915
ENSGALG00010006049	0.875979049	0.251749221
ENSGALG00010006051	0.981981389	0.285884229
ENSGALG00010006052	0.883509874	0.380085223
ENSGALG00010006053	0.932135849	0.249846693
ENSGALG00010006054	0.376469181	0.114887751
ENSGALG00010006055	0.966584843	0.311195149
ENSGALG00010006057	0.912361721	0.27414705
ENSGALG00010006058	0.05197934	0.14495833
ENSGALG00010006059	0.933873767	0.26229376
ENSGALG00010006060	0.837492065	0.112310867
ENSGALG00010006061	0.852315618	0.324072573
ENSGALG00010006062	0.29617809	0.309362499
ENSGALG00010006063	0.892162555	0.385585867
ENSGALG00010006064	0.790188568	0.29008171
ENSGALG00010006065	0.3207622	0.38418892
ENSGALG00010006066	0.921584259	0.280825751
ENSGALG00010006068	0.422264561	0.132048224
ENSGALG00010006069	0.997542704	0.348863768
ENSGALG00010006070	0.252962569	0.019963585
ENSGALG00010006071	0.244081578	0.450511409
ENSGALG00010006072	0.971292547	0.274038599
ENSGALG00010006073	0.679682664	0.383144518
ENSGALG00010006075	0.70145155	0.249031403
ENSGALG00010006076	0.887357578	0.327338188
ENSGALG00010006077	0.938357376	0.298858279
ENSGALG00010006078	0.971519779	0.229409651
ENSGALG00010006079	0.408876452	0.029651334
ENSGALG00010006080	0.077464899	0.000275581
ENSGALG00010006081	0.4222328	0.091712051
ENSGALG00010006082	0.430056618	0.437962011
ENSGALG00010006083	0.255375373	0.086379041
ENSGALG00010006084	0.973612281	0.231251811
ENSGALG00010006085	0.018218104	0.162247183
ENSGALG00010006086	0.035357136	0.162462443

ENSGALG00010006087	0.525705259	0.192672484
ENSGALG00010006091	0.984527966	0.408064393
ENSGALG00010006096	0.917920374	0.486044926
ENSGALG00010006098	0.322445469	0.12071103
ENSGALG00010006100	0.68856425	0.052976648
ENSGALG00010006101	0.982660858	0.308233025
ENSGALG00010006102	0.518451772	0.210095435
ENSGALG00010006103	0.948596717	0.313633337
ENSGALG00010006104	0.097308482	0.337550935
ENSGALG00010006106	0.701855338	0.278817153
ENSGALG00010006107	0.973093096	0.358568261
ENSGALG00010006108	0.546957716	0.522632281
ENSGALG00010006110	0.363113293	0.145190058
ENSGALG00010006111	0.985801713	0.349804154
ENSGALG00010006113	0.973026111	0.362182026
ENSGALG00010006117	0.367251011	0.280386942
ENSGALG00010006118	0.666471822	0.02168643
ENSGALG00010006119	0.734537844	0.257177138
ENSGALG00010006120	0.809665393	0.230515288
ENSGALG00010006121	0.910138562	0.32462986
ENSGALG00010006122	0.501684793	0.152725632
ENSGALG00010006123	0.946170543	0.317504971
ENSGALG00010006124	0.861752126	0.297745524
ENSGALG00010006126	0.452027473	0.03835589
ENSGALG00010006128	0.983665854	0.354708027
ENSGALG00010006131	0.626359497	0.350571837
ENSGALG00010006132	0.927854834	0.255152491
ENSGALG00010006134	0.982151905	0.346170676
ENSGALG00010006135	0.378825597	0.199138949
ENSGALG00010006136	0.37802745	0.171540105
ENSGALG00010006138	0.329744371	0.371370966
ENSGALG00010006139	0.669409648	0.157450027
ENSGALG00010006145	0.38718504	0.101838932
ENSGALG00010006146	0.770581638	0.186259254
ENSGALG00010006147	0.179173248	0.216519043
ENSGALG00010006151	0.549732411	0.192427333
ENSGALG00010006152	0.027851816	0.14441112
ENSGALG00010006154	0.306269874	0.488490028
ENSGALG00010006155	0.802355923	0.153012866
ENSGALG00010006156	0.954067412	0.303819687
ENSGALG00010006157	0.976211681	0.335931666
ENSGALG00010006159	0.489626513	0.217953993
ENSGALG00010006163	0.725129725	0.441440434
ENSGALG00010006169	0.264239718	0.073427965
ENSGALG00010006170	0.45943766	0.392894758
ENSGALG00010006171	0.167446935	0.233672052
ENSGALG00010006172	0.863819985	0.408571505
ENSGALG00010006173	0.937078963	0.235012712
ENSGALG00010006175	0.946681949	0.192048686
ENSGALG00010006176	0.201764926	0.136964694
ENSGALG00010006177	0.942812675	0.25096924
ENSGALG00010006178	0.925193491	0.329209528
ENSGALG00010006179	0.200570274	0.091667991
ENSGALG00010006180	0.789611754	0.377681757
ENSGALG00010006183	0.825667834	0.095875451
ENSGALG00010006184	0.575783925	0.168575477

ENSGALG00010006186	0.583801249	0.336223585
ENSGALG00010006187	0.967807656	0.369316332
ENSGALG00010006188	0.113062198	0.076772557
ENSGALG00010006193	0.192808433	0.004211551
ENSGALG00010006197	0.84027474	0.346016251
ENSGALG00010006198	0.906107698	0.419370856
ENSGALG00010006199	0.42782925	0.112968953
ENSGALG00010006200	0.579381643	0.322081479
ENSGALG00010006201	0.976708947	0.323658713
ENSGALG00010006202	0.883940364	0.207191882
ENSGALG00010006203	0.985412275	0.279747168
ENSGALG00010006205	0.50427348	0.088484936
ENSGALG00010006207	0.773178421	0.352680899
ENSGALG00010006208	0.02211005	0.18206397
ENSGALG00010006210	0.011730111	0.196288363
ENSGALG00010006212	0.054397003	0.280234233
ENSGALG00010006213	0.762297002	0.002439843
ENSGALG00010006214	0.348638671	0.161958735
ENSGALG00010006217	0.941212107	0.43410535
ENSGALG00010006219	0.903873125	0.261407673
ENSGALG00010006220	0.989422047	0.331227848
ENSGALG00010006222	0.690229393	0.206028377
ENSGALG00010006223	0.571935146	0.259077516
ENSGALG00010006224	0.193612503	0.242140732
ENSGALG00010006227	0.960655778	0.351318098
ENSGALG00010006228	0.117135557	0.146182479
ENSGALG00010006229	0.87526	0.336284605
ENSGALG00010006230	0.992606046	0.338211481
ENSGALG00010006231	0.47432246	0.174095382
ENSGALG00010006232	0.814653726	0.064496912
ENSGALG00010006236	0.849045547	0.238632831
ENSGALG00010006237	0.897882767	0.359963676
ENSGALG00010006238	0.562567503	0.171952828
ENSGALG00010006239	0.992021918	0.319495048
ENSGALG00010006240	0.808990582	0.302500066
ENSGALG00010006241	0.985222607	0.289845737
ENSGALG00010006242	0.536573278	0.312854387
ENSGALG00010006243	0.803938684	0.452372466
ENSGALG00010006244	0.928905529	0.339375783
ENSGALG00010006245	0.637808467	0.023715333
ENSGALG00010006246	0.234402806	0.047342383
ENSGALG00010006247	0.085845322	0.129897524
ENSGALG00010006249	0.773245359	0.18091734
ENSGALG00010006250	0.560319604	0.08110139
ENSGALG00010006251	0.668946577	0.328948311
ENSGALG00010006252	0.973340557	0.332479535
ENSGALG00010006254	0.99299954	0.33394205
ENSGALG00010006258	0.012184093	0.540210871
ENSGALG00010006259	0.556547417	0.14778579
ENSGALG00010006260	0.651043613	0.398147539
ENSGALG00010006263	0.209775994	0.090518384
ENSGALG00010006266	0.440372721	0.318117424
ENSGALG00010006269	0.986805542	0.347552717
ENSGALG00010006270	0.363646358	0.142448516
ENSGALG00010006271	0.413222587	0.086530061
ENSGALG00010006272	0.475612077	0.164386681

ENSGALG00010006273	0.125246052	0.156619152
ENSGALG00010006274	0.553960346	0.231282983
ENSGALG00010006275	0.323006951	0.337030561
ENSGALG00010006277	0.218889418	0.114210848
ENSGALG00010006279	0.995835471	0.323848854
ENSGALG00010006280	0.546691943	0.235199091
ENSGALG00010006281	0.117672892	0.229118214
ENSGALG00010006283	0.194040703	0.297377193
ENSGALG00010006285	0.940602528	0.427810112
ENSGALG00010006287	0.96114406	0.268771899
ENSGALG00010006288	0.639644138	0.083739025
ENSGALG00010006289	0.364581264	0.057592898
ENSGALG00010006291	0.940546633	0.389265532
ENSGALG00010006292	0.090770829	0.281931965
ENSGALG00010006293	0.988084473	0.319556455
ENSGALG00010006294	0.915557786	0.362133506
ENSGALG00010006296	0.709421275	0.406000396
ENSGALG00010006297	0.723362365	0.2031375
ENSGALG00010006298	0.952748241	0.284224222
ENSGALG00010006300	0.22781856	0.157831454
ENSGALG00010006301	0.705156473	0.37047712
ENSGALG00010006308	0.515176991	0.260165925
ENSGALG00010006310	0.944567268	0.417438997
ENSGALG00010006311	0.956421826	0.362662377
ENSGALG00010006315	0.125119703	0.200269423
ENSGALG00010006318	0.960809734	0.229295094
ENSGALG00010006323	0.844551723	0.206648108
ENSGALG00010006326	0.914200967	0.486854517
ENSGALG00010006327	0.922786813	0.193709399
ENSGALG00010006328	0.36919582	0.393584224
ENSGALG00010006329	0.753080343	0.36175247
ENSGALG00010006330	0.046551646	0.053640801
ENSGALG00010006331	0.900188704	0.160421627
ENSGALG00010006335	0.463235888	0.207880444
ENSGALG00010006336	0.993948623	0.346454884
ENSGALG00010006339	0.970333212	0.243354116
ENSGALG00010006341	0.80312312	0.255774418
ENSGALG00010006342	0.897688677	0.177188641
ENSGALG00010006343	0.530934554	0.276567787
ENSGALG00010006344	0.474073154	0.33762903
ENSGALG00010006345	0.814859505	0.281294295
ENSGALG00010006346	0.334770131	0.107550766
ENSGALG00010006347	0.976557384	0.288380018
ENSGALG00010006348	0.959503241	0.270546543
ENSGALG00010006349	0.123552071	0.292330233
ENSGALG00010006350	0.870997354	0.349048161
ENSGALG00010006351	0.850470041	0.191333303
ENSGALG00010006352	0.964384362	0.273146769
ENSGALG00010006353	0.091642254	0.195973548
ENSGALG00010006354	0.345563967	0.539322521
ENSGALG00010006355	0.985089359	0.351522991
ENSGALG00010006357	0.441770554	0.140145291
ENSGALG00010006359	0.593486424	0.293746924
ENSGALG00010006360	0.052836869	0.101827534
ENSGALG00010006361	0.967825629	0.312489216
ENSGALG00010006363	0.850674577	0.443833421

ENSGALG00010006371	0.581190394	0.413109814
ENSGALG00010006372	0.744540409	0.310859578
ENSGALG00010006373	0.302872319	0.228723642
ENSGALG00010006377	0.600496229	0.130052872
ENSGALG00010006378	0.919986547	0.302233338
ENSGALG00010006379	0.289519971	0.23105599
ENSGALG00010006381	0.945460164	0.286199426
ENSGALG00010006382	0.586472643	0.037449223
ENSGALG00010006385	0.493878369	0.356494833
ENSGALG00010006387	0.341035137	0.229219755
ENSGALG00010006388	0.262922434	0.138601464
ENSGALG00010006389	0.82106016	0.465483387
ENSGALG00010006391	0.834523804	0.407644406
ENSGALG00010006393	0.799655762	0.440977244
ENSGALG00010006396	0.559173738	0.234928664
ENSGALG00010006397	0.5862764	0.100752261
ENSGALG00010006398	0.144773091	0.033673205
ENSGALG00010006401	0.059174359	0.219786842
ENSGALG00010006402	0.368476583	0.099553507
ENSGALG00010006404	0.281600925	0.171317723
ENSGALG00010006405	0.492883351	0.193792026
ENSGALG00010006408	0.866367625	0.371157487
ENSGALG00010006410	0.566518005	0.375384983
ENSGALG00010006411	0.007131848	0.533865704
ENSGALG00010006416	0.89872696	0.472287801
ENSGALG00010006419	0.679894642	0.460628567
ENSGALG00010006421	0.871470033	0.236546075
ENSGALG00010006423	0.301206853	0.404439853
ENSGALG00010006430	0.592815116	0.208011855
ENSGALG00010006431	0.95312199	0.33940522
ENSGALG00010006433	0.442258235	0.117686659
ENSGALG00010006434	0.900988374	0.302656362
ENSGALG00010006435	0.467745341	0.297729333
ENSGALG00010006436	0.787594863	0.243156604
ENSGALG00010006437	0.427448022	0.102041997
ENSGALG00010006438	0.803413245	0.237798277
ENSGALG00010006440	0.704575566	0.106614288
ENSGALG00010006441	0.905553789	0.325648752
ENSGALG00010006443	0.946790994	0.356558638
ENSGALG00010006445	0.541009733	0.016858784
ENSGALG00010006446	0.639538262	0.388187335
ENSGALG00010006447	0.005451243	0.223294019
ENSGALG00010006449	0.299725593	0.050338716
ENSGALG00010006451	0.817564681	0.295685925
ENSGALG00010006453	0.850698908	0.309083045
ENSGALG00010006454	0.317218683	0.027551815
ENSGALG00010006455	0.036215381	0.132705637
ENSGALG00010006457	0.619469084	0.049976524
ENSGALG00010006458	0.501925815	0.02411461
ENSGALG00010006463	0.227172267	0.227644594
ENSGALG00010006465	0.75552041	0.306238184
ENSGALG00010006466	0.666431755	0.283848077
ENSGALG00010006469	0.430731489	0.123241269
ENSGALG00010006470	0.52256903	0.163003117
ENSGALG00010006471	0.288666205	0.121620874
ENSGALG00010006472	0.195389286	0.178128866

ENSGALG00010006474	0.187514554	0.108479243
ENSGALG00010006478	0.965995376	0.352423143
ENSGALG00010006479	0.285272007	0.109169955
ENSGALG00010006480	0.137074409	0.209396109
ENSGALG00010006481	0.38344303	0.176088516
ENSGALG00010006483	0.495892281	0.20079274
ENSGALG00010006485	0.384884589	0.078217936
ENSGALG00010006486	0.935027304	0.296960953
ENSGALG00010006487	0.996043841	0.349078073
ENSGALG00010006488	0.318788652	0.201270103
ENSGALG00010006490	0.290124509	0.166136778
ENSGALG00010006492	0.474988409	0.367642333
ENSGALG00010006493	0.20977128	0.144828218
ENSGALG00010006494	0.025223182	0.049142008
ENSGALG00010006496	0.765726763	0.263082422
ENSGALG00010006497	0.257638506	0.173160424
ENSGALG00010006499	0.854137837	0.348260869
ENSGALG00010006503	0.232813515	0.176922988
ENSGALG00010006506	0.863526828	0.365022095
ENSGALG00010006508	0.264239718	0.073427965
ENSGALG00010006510	0.777179959	0.226812406
ENSGALG00010006514	0.822136083	0.334118032
ENSGALG00010006515	0.942875963	0.2862626
ENSGALG00010006516	0.956325282	0.306884444
ENSGALG00010006517	0.824344797	0.265301984
ENSGALG00010006519	0.593599655	0.176400255
ENSGALG00010006521	0.794501667	0.276649332
ENSGALG00010006523	0.330870409	0.017225032
ENSGALG00010006524	0.44688589	0.354016979
ENSGALG00010006525	0.917037558	0.366152884
ENSGALG00010006526	0.909617503	0.278716837
ENSGALG00010006527	0.873128888	0.361559822
ENSGALG00010006528	0.736312387	0.368569049
ENSGALG00010006529	0.868702521	0.314055075
ENSGALG00010006530	0.656780958	0.248036687
ENSGALG00010006531	0.402883264	0.115342988
ENSGALG00010006532	0.989262999	0.318505758
ENSGALG00010006533	0.292704713	0.009723639
ENSGALG00010006535	0.30101212	0.182183218
ENSGALG00010006537	0.868323755	0.239970449
ENSGALG00010006538	0.976426143	0.266898289
ENSGALG00010006540	0.531373855	0.099889385
ENSGALG00010006541	0.266137114	0.107784983
ENSGALG00010006542	0.425434948	0.144503696
ENSGALG00010006544	0.966321224	0.31979076
ENSGALG00010006546	0.087947134	0.259594956
ENSGALG00010006547	0.171346878	0.18671556
ENSGALG00010006548	0.0000973	0.245380759
ENSGALG00010006549	0.272652018	0.281906251
ENSGALG00010006550	0.226141252	0.067152688
ENSGALG00010006551	0.252996222	0.123283049
ENSGALG00010006553	0.674310757	0.247203213
ENSGALG00010006555	0.384513156	0.050700089
ENSGALG00010006556	0.712019294	0.146329266
ENSGALG00010006557	0.759531909	0.303913217
ENSGALG00010006558	0.146442103	0.070711242

ENSGALG00010006559	0.102332061	0.177954201
ENSGALG00010006561	0.687243203	0.188259654
ENSGALG00010006564	0.916095994	0.519356845
ENSGALG00010006565	0.642577806	0.015823771
ENSGALG00010006567	0.980245168	0.327867299
ENSGALG00010006568	0.953177388	0.406828232
ENSGALG00010006569	0.727062223	0.248761022
ENSGALG00010006570	0.997179075	0.335960784
ENSGALG00010006573	0.651733519	0.179489358
ENSGALG00010006575	0.733824754	0.329463759
ENSGALG00010006576	0.630639467	0.014168728
ENSGALG00010006577	0.559741545	0.272653337
ENSGALG00010006578	0.381415582	0.158761082
ENSGALG00010006579	0.204484783	0.111640026
ENSGALG00010006580	0.855073604	0.369620512
ENSGALG00010006581	0.804421305	0.365260826
ENSGALG00010006583	0.390174796	0.089394357
ENSGALG00010006584	0.899612476	0.433428655
ENSGALG00010006585	0.596384477	0.485018009
ENSGALG00010006586	0.684253287	0.286050663
ENSGALG00010006587	0.236089331	0.101960766
ENSGALG00010006588	0.835552788	0.373716868
ENSGALG00010006589	0.676038014	0.284531658
ENSGALG00010006590	0.38238129	0.047731314
ENSGALG00010006592	0.814799135	0.256731023
ENSGALG00010006593	0.363541789	0.477615452
ENSGALG00010006594	0.524948949	0.016554301
ENSGALG00010006596	0.688791927	0.029976401
ENSGALG00010006597	0.491271376	0.147715189
ENSGALG00010006598	0.136035637	0.157163419
ENSGALG00010006599	0.437628005	0.184427623
ENSGALG00010006601	0.478966979	0.276152886
ENSGALG00010006602	0.905948592	0.420943341
ENSGALG00010006603	0.869657892	0.326208681
ENSGALG00010006604	0.802099001	0.30993875
ENSGALG00010006605	0.534109888	0.498183749
ENSGALG00010006606	0.988381069	0.348122652
ENSGALG00010006608	0.784102435	0.350323685
ENSGALG00010006609	0.020656918	0.064774402
ENSGALG00010006610	0.464421117	0.176201614
ENSGALG00010006612	0.911397495	0.308185445
ENSGALG00010006614	0.910350045	0.291849781
ENSGALG00010006615	0.922887921	0.480454544
ENSGALG00010006616	0.776620738	0.036060155
ENSGALG00010006617	0.705343505	0.30932745
ENSGALG00010006618	0.903676314	0.436474824
ENSGALG00010006619	0.243241982	0.141488467
ENSGALG00010006620	0.388695239	0.167312355
ENSGALG00010006621	0.648717053	0.421292768
ENSGALG00010006623	0.02695674	0.195877388
ENSGALG00010006625	0.243802365	0.451193702
ENSGALG00010006626	0.703002556	0.271258975
ENSGALG00010006627	0.979798427	0.313410297
ENSGALG00010006628	0.637296104	0.54193683
ENSGALG00010006629	0.851951351	0.434380037
ENSGALG00010006631	0.446546561	0.180252848

ENSGALG00010006632	0.797131802	0.339679194
ENSGALG00010006633	0.961729121	0.409004962
ENSGALG00010006634	0.933259342	0.239014143
ENSGALG00010006635	0.555681323	0.240324984
ENSGALG00010006639	0.747048447	0.263916337
ENSGALG00010006640	0.663605216	0.218038354
ENSGALG00010006641	0.359764388	0.291712476
ENSGALG00010006643	0.55155225	0.470772151
ENSGALG00010006644	0.224673059	0.394837105
ENSGALG00010006646	0.603766575	0.158236572
ENSGALG00010006649	0.264239718	0.073427965
ENSGALG00010006651	0.929597132	0.336986122
ENSGALG00010006652	0.873546391	0.317159956
ENSGALG00010006653	0.997431688	0.311906827
ENSGALG00010006657	0.423584977	0.183248772
ENSGALG00010006659	0.851912781	0.300917142
ENSGALG00010006662	0.002346192	0.176799405
ENSGALG00010006663	0.646107629	0.194502589
ENSGALG00010006664	0.38718504	0.101838932
ENSGALG00010006667	0.265558089	0.028821141
ENSGALG00010006668	0.701816149	0.222982998
ENSGALG00010006669	0.521930895	0.258285298
ENSGALG00010006670	0.504317729	0.271197824
ENSGALG00010006671	0.440281419	0.080881678
ENSGALG00010006672	0.782520466	0.223731147
ENSGALG00010006673	0.963900587	0.349000171
ENSGALG00010006674	0.18447255	0.321216244
ENSGALG00010006676	0.030637844	0.185580366
ENSGALG00010006678	0.833424225	0.356199328
ENSGALG00010006679	0.104362205	0.069579145
ENSGALG00010006681	0.310234322	0.059859784
ENSGALG00010006682	0.150194346	0.222485047
ENSGALG00010006683	0.297090418	0.136289521
ENSGALG00010006684	0.390466756	0.106163828
ENSGALG00010006685	0.901285311	0.347547572
ENSGALG00010006686	0.594364474	0.225021311
ENSGALG00010006687	0.571664577	0.19672089
ENSGALG00010006691	0.110755281	0.399241662
ENSGALG00010006692	0.009429422	0.134618735
ENSGALG00010006693	0.393293574	0.180300648
ENSGALG00010006694	0.511990851	0.470205549
ENSGALG00010006698	0.071132652	0.475126547
ENSGALG00010006699	0.596878346	0.287670893
ENSGALG00010006702	0.41879472	0.14401035
ENSGALG00010006705	0.600683349	0.215693269
ENSGALG00010006706	0.031329216	0.144699932
ENSGALG00010006707	0.66803062	0.261745818
ENSGALG00010006708	0.249895964	0.10495343
ENSGALG00010006710	0.562885938	0.13207895
ENSGALG00010006713	0.298140893	0.421788492
ENSGALG00010006715	0.977007214	0.309844255
ENSGALG00010006716	0.985696935	0.31266727
ENSGALG00010006717	0.378386997	0.131934372
ENSGALG00010006718	0.888094379	0.329403991
ENSGALG00010006720	0.964451638	0.33171247
ENSGALG00010006721	0.30622539	0.12456887

ENSGALG00010006722	0.422845567	0.249434515
ENSGALG00010006723	0.065916238	0.156310701
ENSGALG00010006724	0.271701261	0.072417625
ENSGALG00010006725	0.118562097	0.25015439
ENSGALG00010006726	0.092059195	0.088327562
ENSGALG00010006728	0.981700154	0.247979895
ENSGALG00010006729	0.285272007	0.109169955
ENSGALG00010006730	0.038199932	0.114027168
ENSGALG00010006731	0.421743348	0.025884428
ENSGALG00010006735	0.539098195	0.565518189
ENSGALG00010006736	0.315858663	0.645614603
ENSGALG00010006738	0.438218602	0.08510368
ENSGALG00010006739	0.331263383	0.32986735
ENSGALG00010006740	0.27278291	0.05676752
ENSGALG00010006741	0.208353503	0.26439411
ENSGALG00010006744	0.042343157	0.133098028
ENSGALG00010006745	0.043544722	0.088274573
ENSGALG00010006746	0.424169595	0.262793288
ENSGALG00010006748	0.968757293	0.343172523
ENSGALG00010006750	0.775943759	0.211619145
ENSGALG00010006752	0.954827593	0.242789735
ENSGALG00010006759	0.657067604	0.030786243
ENSGALG00010006760	0.73565268	0.32729598
ENSGALG00010006761	0.95520805	0.33617771
ENSGALG00010006762	0.265558089	0.028821141
ENSGALG00010006766	0.074962637	0.299647683
ENSGALG00010006767	0.987679643	0.314547023
ENSGALG00010006769	0.544429863	0.194115562
ENSGALG00010006770	0.125310258	0.260060126
ENSGALG00010006774	0.483072825	0.484151838
ENSGALG00010006777	0.501749778	0.227205256
ENSGALG00010006779	0.548925716	0.176108321
ENSGALG00010006780	0.488988474	0.092908732
ENSGALG00010006783	0.171913555	0.242423517
ENSGALG00010006784	0.6677448	0.326050973
ENSGALG00010006785	0.869150634	0.451783971
ENSGALG00010006786	0.386627722	0.084137494
ENSGALG00010006789	0.420781679	0.470790778
ENSGALG00010006794	0.257246662	0.109487779
ENSGALG00010006795	0.043616815	0.215810579
ENSGALG00010006799	0.261865986	0.070958853
ENSGALG00010006803	0.876159321	0.234572118
ENSGALG00010006804	0.875381081	0.313623026
ENSGALG00010006805	0.168321586	0.146624045
ENSGALG00010006806	0.316899359	0.255164935
ENSGALG00010006808	0.297716184	0.001645542
ENSGALG00010006814	0.270775198	0.031145073
ENSGALG00010006816	0.97139617	0.255555089
ENSGALG00010006819	0.903130502	0.088585546
ENSGALG00010006820	0.704709969	0.290075427
ENSGALG00010006821	0.723707208	0.156812182
ENSGALG00010006823	0.001907955	0.10870884
ENSGALG00010006824	0.655229343	0.262735098
ENSGALG00010006828	0.464880837	0.012361113
ENSGALG00010006829	0.044640162	0.167754086
ENSGALG00010006831	0.373446581	0.117589412

ENSGALG00010006832	0.760691721	0.026127363
ENSGALG00010006833	0.594930662	0.062208807
ENSGALG00010006835	0.920548212	0.34231951
ENSGALG00010006836	0.485794128	0.262733795
ENSGALG00010006838	0.890089395	0.400643463
ENSGALG00010006839	0.550027728	0.383241221
ENSGALG00010006840	0.626470738	0.014543399
ENSGALG00010006842	0.396302421	0.165008599
ENSGALG00010006843	0.690341122	0.022715897
ENSGALG00010006845	0.73465908	0.257093044
ENSGALG00010006847	0.975693063	0.324028907
ENSGALG00010006848	0.341117049	0.184223483
ENSGALG00010006849	0.700663166	0.205713868
ENSGALG00010006850	0.844454567	0.227171292
ENSGALG00010006851	0.737669242	0.409776131
ENSGALG00010006853	0.134962965	0.004503123
ENSGALG00010006855	0.954016945	0.338324265
ENSGALG00010006865	0.705969566	0.260639524
ENSGALG00010006869	0.860745396	0.190254528
ENSGALG00010006870	0.921063213	0.19793942
ENSGALG00010006875	0.98815265	0.332935897
ENSGALG00010006877	0.97277083	0.265111425
ENSGALG00010006878	0.39190877	0.131701825
ENSGALG00010006881	0.573979212	0.426769409
ENSGALG00010006883	0.972106142	0.436010452
ENSGALG00010006884	0.534294507	0.048716621
ENSGALG00010006885	0.525671214	0.017132207
ENSGALG00010006886	0.393617809	0.024107571
ENSGALG00010006888	0.400890859	0.096271928
ENSGALG00010006893	0.187514554	0.108479243
ENSGALG00010006894	0.376027077	0.160418854
ENSGALG00010006895	0.143865107	0.471109384
ENSGALG00010006896	0.635952182	0.2006274
ENSGALG00010006898	0.344512287	0.234480692
ENSGALG00010006899	0.265558089	0.028821141
ENSGALG00010006900	0.02931309	0.104495807
ENSGALG00010006901	0.976478349	0.333201982
ENSGALG00010006903	0.932355121	0.183651234
ENSGALG00010006904	0.305017618	0.066195483
ENSGALG00010006908	0.169302787	0.144152203
ENSGALG00010006912	0.441047919	0.147337308
ENSGALG00010006913	0.850183162	0.42784386
ENSGALG00010006914	0.533200786	0.164492814
ENSGALG00010006915	0.454748752	0.389021691
ENSGALG00010006917	0.572249098	0.076642541
ENSGALG00010006918	0.373495264	0.061911944
ENSGALG00010006920	0.433169918	0.196540535
ENSGALG00010006922	0.187033479	0.016763766
ENSGALG00010006923	0.051631887	0.074634259
ENSGALG00010006925	0.486038373	0.330004745
ENSGALG00010006926	0.217193344	0.10154739
ENSGALG00010006928	0.560838931	0.22298192
ENSGALG00010006930	0.290122505	0.464993379
ENSGALG00010006932	0.246886976	0.171410319
ENSGALG00010006936	0.298399403	0.094963927
ENSGALG00010006937	0.495855034	0.326982799

ENSGALG00010006938	0.436251647	0.460841142
ENSGALG00010006939	0.967835818	0.322899413
ENSGALG00010006941	0.227985125	0.179615573
ENSGALG00010006942	0.497900812	0.238110765
ENSGALG00010006943	0.96543235	0.334733275
ENSGALG00010006944	0.953667989	0.368400438
ENSGALG00010006945	0.189240572	0.04996112
ENSGALG00010006946	0.013079389	0.166634288
ENSGALG00010006947	0.395705092	0.355839569
ENSGALG00010006948	0.203991683	0.058221925
ENSGALG00010006950	0.874550022	0.442975418
ENSGALG00010006953	0.98176337	0.30900893
ENSGALG00010006957	0.852845884	0.426240577
ENSGALG00010006958	0.422932345	0.320061084
ENSGALG00010006960	0.902317308	0.429181911
ENSGALG00010006961	0.944579272	0.247774955
ENSGALG00010006963	0.304394235	0.105991836
ENSGALG00010006964	0.392206929	0.143464443
ENSGALG00010006966	0.192228799	0.244713287
ENSGALG00010006967	0.526941029	0.442458428
ENSGALG00010006970	0.791432723	0.231986573
ENSGALG00010006971	0.122234441	0.1635518
ENSGALG00010006972	0.914943806	0.40344649
ENSGALG00010006973	0.129344242	0.012875835
ENSGALG00010006974	0.858121995	0.531424868
ENSGALG00010006975	0.940415126	0.301851362
ENSGALG00010006976	0.937738685	0.310265475
ENSGALG00010006977	0.294226904	0.099361029
ENSGALG00010006978	0.380291529	0.371093026
ENSGALG00010006979	0.56568017	0.048995438
ENSGALG00010006980	0.009429422	0.134618735
ENSGALG00010006982	0.185362508	0.114656568
ENSGALG00010006983	0.853967371	0.250465093
ENSGALG00010006984	0.175070675	0.102419576
ENSGALG00010006985	0.131572695	0.06618527
ENSGALG00010006986	0.376061206	0.219717698
ENSGALG00010006987	0.590507546	0.072211601
ENSGALG00010006988	0.901664439	0.355174614
ENSGALG00010006989	0.189240572	0.04996112
ENSGALG00010006990	0.360182321	0.109111334
ENSGALG00010006991	0.920275504	0.41257906
ENSGALG00010006992	0.637273366	0.341610027
ENSGALG00010006993	0.280412698	0.192242784
ENSGALG00010006995	0.055072366	0.269090805
ENSGALG00010006998	0.903125462	0.353149842
ENSGALG00010006999	0.995165295	0.334439664
ENSGALG00010007000	0.843551294	0.169008763
ENSGALG00010007001	0.845467046	0.280715512
ENSGALG00010007002	0.826896902	0.296615688
ENSGALG00010007004	0.035248149	0.150556591
ENSGALG00010007005	0.318687505	0.288388919
ENSGALG00010007007	0.974874705	0.29310442
ENSGALG00010007010	0.238798511	0.049658162
ENSGALG00010007011	0.855468672	0.263459461
ENSGALG00010007012	0.911938931	0.360626669
ENSGALG00010007014	0.057689882	0.461357923

ENSGALG00010007017	0.964703129	0.295081849
ENSGALG00010007018	0.17304218	0.03557151
ENSGALG00010007020	0.282754916	0.323313762
ENSGALG00010007021	0.113952352	0.294776878
ENSGALG00010007022	0.539708078	0.220276126
ENSGALG00010007023	0.150541675	0.404144522
ENSGALG00010007026	0.607259018	0.160727156
ENSGALG00010007027	0.386613367	0.019266881
ENSGALG00010007028	0.365769806	0.550992416
ENSGALG00010007031	0.527077587	0.189594978
ENSGALG00010007033	0.931952795	0.2798311
ENSGALG00010007034	0.66471661	0.215343526
ENSGALG00010007035	0.336981112	0.183312475
ENSGALG00010007037	0.394927857	0.119113854
ENSGALG00010007038	0.94620361	0.28504413
ENSGALG00010007040	0.278765262	0.561539442
ENSGALG00010007041	0.889063878	0.120189813
ENSGALG00010007042	0.264239718	0.073427965
ENSGALG00010007044	0.298147409	0.947793135
ENSGALG00010007045	0.232169808	0.218282188
ENSGALG00010007049	0.266390355	0.110076851
ENSGALG00010007050	0.360211434	0.130556139
ENSGALG00010007051	0.721156718	0.075394243
ENSGALG00010007052	0.669165296	0.077258689
ENSGALG00010007054	0.932229037	0.284898285
ENSGALG00010007055	0.387690559	0.167570115
ENSGALG00010007056	0.381476961	0.077101777
ENSGALG00010007058	0.730630771	0.175553945
ENSGALG00010007065	0.285272007	0.109169955
ENSGALG00010007066	0.460467165	0.0327956
ENSGALG00010007067	0.849682209	0.3365614
ENSGALG00010007069	0.779316773	0.257010877
ENSGALG00010007070	0.295729583	0.090714348
ENSGALG00010007072	0.744551927	0.256000716
ENSGALG00010007073	0.565502868	0.198302349
ENSGALG00010007074	0.024759706	0.290387463
ENSGALG00010007075	0.159142406	0.35223769
ENSGALG00010007080	0.810424878	0.212731765
ENSGALG00010007082	0.925027279	0.309231899
ENSGALG00010007083	0.073376936	0.164024345
ENSGALG00010007085	0.943677657	0.429474577
ENSGALG00010007087	0.184559753	0.020588946
ENSGALG00010007088	0.009428035	0.119453901
ENSGALG00010007090	0.924498686	0.215399106
ENSGALG00010007091	0.081969882	0.163235971
ENSGALG00010007092	0.874770887	0.299461136
ENSGALG00010007093	0.185370537	0.156674538
ENSGALG00010007096	0.039587847	0.080998562
ENSGALG00010007097	0.838882809	0.289566229
ENSGALG00010007098	0.605356431	0.133569922
ENSGALG00010007100	0.563110044	0.063989487
ENSGALG00010007102	0.860278425	0.119805086
ENSGALG00010007103	0.661856036	0.283120062
ENSGALG00010007104	0.47401914	0.141095213
ENSGALG00010007106	0.758794301	0.270107801
ENSGALG00010007108	0.474022162	0.034109303

ENSGALG00010007110	0.948096228	0.268036836
ENSGALG00010007114	0.869354534	0.532056439
ENSGALG00010007115	0.025123994	0.221855222
ENSGALG00010007116	0.497179646	0.132176742
ENSGALG00010007118	0.14174918	0.140251904
ENSGALG00010007119	0.202647901	0.447600028
ENSGALG00010007121	0.50240768	0.251798554
ENSGALG00010007123	0.228986695	0.131712485
ENSGALG00010007124	0.684676642	0.278308303
ENSGALG00010007126	0.736939629	0.290465261
ENSGALG00010007128	0.901850434	0.200997384
ENSGALG00010007130	0.245947144	0.019015193
ENSGALG00010007131	0.08475151	0.169540469
ENSGALG00010007133	0.53137906	0.5740837
ENSGALG00010007134	0.18070123	0.308411526
ENSGALG00010007135	0.878629034	0.427505188
ENSGALG00010007136	0.274586994	0.115798603
ENSGALG00010007137	0.002510958	0.264896934
ENSGALG00010007138	0.154219825	0.139547935
ENSGALG00010007139	0.220014762	0.027996214
ENSGALG00010007140	0.043255806	0.121677865
ENSGALG00010007141	0.94215225	0.376231909
ENSGALG00010007142	0.734203869	0.352981702
ENSGALG00010007143	0.988030876	0.339006321
ENSGALG00010007144	0.128456369	0.294809833
ENSGALG00010007146	0.797789457	0.119494865
ENSGALG00010007149	0.396366346	0.15714058
ENSGALG00010007150	0.534919744	0.20594822
ENSGALG00010007152	0.326697265	0.053245649
ENSGALG00010007153	0.425174823	0.220759366
ENSGALG00010007154	0.548236938	0.189832536
ENSGALG00010007155	0.271701261	0.072417625
ENSGALG00010007156	0.584305091	0.27009239
ENSGALG00010007157	0.185115883	0.084746236
ENSGALG00010007158	0.119278234	0.236527989
ENSGALG00010007159	0.310434552	0.241198621
ENSGALG00010007160	0.099459382	0.298674243
ENSGALG00010007162	0.926912191	0.37994148
ENSGALG00010007163	0.16098583	0.1577073
ENSGALG00010007164	0.883332474	0.440454392
ENSGALG00010007165	0.065828993	0.213972347
ENSGALG00010007166	0.708490131	0.283021731
ENSGALG00010007168	0.103699928	0.001651728
ENSGALG00010007169	0.857240935	0.426464659
ENSGALG00010007172	0.623812849	0.445679214
ENSGALG00010007173	0.643270835	0.11725624
ENSGALG00010007174	0.639229045	0.338907413
ENSGALG00010007175	0.24732621	0.426241881
ENSGALG00010007176	0.265558089	0.028821141
ENSGALG00010007178	0.3068634	0.08143621
ENSGALG00010007181	0.788994694	0.317943452
ENSGALG00010007182	0.123442368	0.132272207
ENSGALG00010007183	0.899549924	0.366875753
ENSGALG00010007184	0.980011175	0.328288273
ENSGALG00010007185	0.444032982	0.117142645
ENSGALG00010007187	0.649977794	0.381762681

ENSGALG00010007188	0.8971429	0.424593626
ENSGALG00010007189	0.845094599	0.453932757
ENSGALG00010007190	0.790816221	0.160691569
ENSGALG00010007191	0.378022522	0.12438851
ENSGALG00010007193	0.833304069	0.277517645
ENSGALG00010007197	0.716248457	0.221228334
ENSGALG00010007200	0.929627939	0.331752641
ENSGALG00010007201	0.875394586	0.246104711
ENSGALG00010007203	0.779487117	0.129904517
ENSGALG00010007204	0.784794931	0.313074684
ENSGALG00010007205	0.830538848	0.29331661
ENSGALG00010007206	0.362041241	0.114713121
ENSGALG00010007207	0.974934661	0.359724723
ENSGALG00010007208	0.352811971	0.25549689
ENSGALG00010007209	0.105689866	0.047456933
ENSGALG00010007210	0.794719727	0.289503137
ENSGALG00010007211	0.212592176	0.199636973
ENSGALG00010007212	0.138008045	0.171652802
ENSGALG00010007214	0.927932233	0.21878155
ENSGALG00010007215	0.299135455	0.121432985
ENSGALG00010007216	0.217104944	0.154584426
ENSGALG00010007217	0.521111236	0.280540404
ENSGALG00010007218	0.428654392	0.093790074
ENSGALG00010007220	0.427721754	0.350441719
ENSGALG00010007221	0.44909469	0.197877763
ENSGALG00010007222	0.34032496	0.20641554
ENSGALG00010007223	0.855423868	0.338948079
ENSGALG00010007224	0.982478969	0.374310061
ENSGALG00010007226	0.997289194	0.336947085
ENSGALG00010007228	0.382985366	0.1291087
ENSGALG00010007230	0.056080156	0.021833681
ENSGALG00010007231	0.375545622	0.141397116
ENSGALG00010007232	0.689729548	0.000862053
ENSGALG00010007236	0.972703669	0.338210224
ENSGALG00010007237	0.2896197	0.603980439
ENSGALG00010007238	0.766915399	0.366000751
ENSGALG00010007243	0.525899293	0.133069236
ENSGALG00010007245	0.84016783	0.416467623
ENSGALG00010007251	0.924814674	0.311822157
ENSGALG00010007252	0.988344708	0.333242824
ENSGALG00010007253	0.133032851	0.123652409
ENSGALG00010007254	0.009595786	0.236128949
ENSGALG00010007258	0.94121763	0.310849317
ENSGALG00010007259	0.827148541	0.316573097
ENSGALG00010007261	0.443301663	0.074818784
ENSGALG00010007263	0.399682938	0.156656358
ENSGALG00010007264	0.67218625	0.240719816
ENSGALG00010007265	0.609004298	0.167946281
ENSGALG00010007266	0.668503666	0.291438766
ENSGALG00010007267	0.881448678	0.119048043
ENSGALG00010007268	0.51235953	0.196324531
ENSGALG00010007269	0.450214564	0.288590152
ENSGALG00010007270	0.961335445	0.275093297
ENSGALG00010007271	0.354205285	0.12892787
ENSGALG00010007274	0.449894881	0.686553568
ENSGALG00010007275	0.902132958	0.276184411

ENSGALG00010007277	0.375082889	0.152666944
ENSGALG00010007278	0.684148611	0.228871417
ENSGALG00010007283	0.663064315	0.336405115
ENSGALG00010007284	0.884964124	0.216619401
ENSGALG00010007285	0.731126764	0.137092297
ENSGALG00010007287	0.232210118	0.286057892
ENSGALG00010007294	0.145268336	0.046523532
ENSGALG00010007296	0.362952673	0.224034773
ENSGALG00010007298	0.194041025	0.048796483
ENSGALG00010007300	0.794426335	0.09864259
ENSGALG00010007302	0.784992664	0.353682572
ENSGALG00010007304	0.99090756	0.332997765
ENSGALG00010007305	0.134339302	0.20703022
ENSGALG00010007315	0.909879437	0.124744727
ENSGALG00010007318	0.951420169	0.437323692
ENSGALG00010007319	0.954013845	0.392693588
ENSGALG00010007320	0.564512377	0.232194632
ENSGALG00010007321	0.837402437	0.366588829
ENSGALG00010007322	0.934633182	0.27047194
ENSGALG00010007323	0.805782543	0.536724401
ENSGALG00010007324	0.879543664	0.365990544
ENSGALG00010007325	0.793609897	0.34397382
ENSGALG00010007326	0.965864904	0.341215821
ENSGALG00010007327	0.033048351	0.011248539
ENSGALG00010007328	0.528159509	0.425225024
ENSGALG00010007330	0.328195648	0.193703918
ENSGALG00010007331	0.495247221	0.182126244
ENSGALG00010007333	0.927791366	0.251819375
ENSGALG00010007334	0.979467636	0.339804352
ENSGALG00010007337	0.363883361	0.007169681
ENSGALG00010007339	0.006531738	0.006476291
ENSGALG00010007342	0.009913564	0.319435298
ENSGALG00010007343	0.239466507	0.184011583
ENSGALG00010007344	0.484354048	0.003397795
ENSGALG00010007345	0.825556214	0.2772057
ENSGALG00010007348	0.196038127	0.155571026
ENSGALG00010007349	0.401261446	0.464525907
ENSGALG00010007350	0.327548727	0.376586909
ENSGALG00010007353	0.335718076	0.175391907
ENSGALG00010007355	0.075083243	0.08983048
ENSGALG00010007356	0.194960233	0.044643819
ENSGALG00010007357	0.317617038	0.150602043
ENSGALG00010007360	0.898372999	0.267915997
ENSGALG00010007365	0.994992225	0.336144713
ENSGALG00010007369	0.081634227	0.061371023
ENSGALG00010007371	0.769115183	0.221830478
ENSGALG00010007372	0.170425698	0.272333705
ENSGALG00010007373	0.405074429	0.156360385
ENSGALG00010007374	0.964312712	0.251533255
ENSGALG00010007375	0.946031151	0.269151808
ENSGALG00010007376	0.929149704	0.323915951
ENSGALG00010007379	0.309655755	0.987979031
ENSGALG00010007380	0.300546372	0.207185976
ENSGALG00010007381	0.992373247	0.309187359
ENSGALG00010007382	0.912801621	0.284387967
ENSGALG00010007386	0.265558089	0.028821141

ENSGALG00010007387	0.024796536	0.298082747
ENSGALG00010007388	0.445403753	0.02466208
ENSGALG00010007389	0.420011507	0.183671385
ENSGALG00010007390	0.695188148	0.151610415
ENSGALG00010007391	0.939671023	0.296345146
ENSGALG00010007392	0.555188264	0.211726358
ENSGALG00010007393	0.970024395	0.290513109
ENSGALG00010007394	0.066937894	0.156633736
ENSGALG00010007395	0.48578899	0.177061229
ENSGALG00010007396	0.388398154	0.156498862
ENSGALG00010007399	0.966272338	0.294717134
ENSGALG00010007400	0.964975273	0.32049917
ENSGALG00010007401	0.904769227	0.315191231
ENSGALG00010007403	0.948433304	0.267411542
ENSGALG00010007404	0.778626679	0.25202255
ENSGALG00010007405	0.257225351	0.161505109
ENSGALG00010007406	0.206520221	0.238812947
ENSGALG00010007408	0.931305423	0.29302291
ENSGALG00010007410	0.394894278	0.058875926
ENSGALG00010007412	0.022158214	0.185570774
ENSGALG00010007413	0.382801765	0.160449449
ENSGALG00010007414	0.833644433	0.214502491
ENSGALG00010007416	0.937028923	0.249096456
ENSGALG00010007417	0.45607147	0.220265817
ENSGALG00010007418	0.378542153	0.20653271
ENSGALG00010007419	0.230808479	0.070365202
ENSGALG00010007422	0.225584105	0.236913864
ENSGALG00010007423	0.858505801	0.094635143
ENSGALG00010007425	0.745790494	0.021350167
ENSGALG00010007427	0.013740372	0.09360751
ENSGALG00010007428	0.15879916	0.126551358
ENSGALG00010007429	0.625120296	0.233051803
ENSGALG00010007430	0.935455263	0.256900742
ENSGALG00010007432	0.962144066	0.338702081
ENSGALG00010007433	0.267158904	0.061604748
ENSGALG00010007434	0.265558089	0.028821141
ENSGALG00010007436	0.901944567	0.253455442
ENSGALG00010007437	0.909359089	0.327006006
ENSGALG00010007439	0.716658035	0.333194765
ENSGALG00010007440	0.277188858	0.521691462
ENSGALG00010007441	0.928501548	0.323365578
ENSGALG00010007442	0.616127716	0.130183702
ENSGALG00010007444	0.364718386	0.141909851
ENSGALG00010007445	0.072571977	0.110244959
ENSGALG00010007447	0.212871068	0.20313617
ENSGALG00010007448	0.588577619	0.16213138
ENSGALG00010007452	0.245700908	0.010899108
ENSGALG00010007453	0.391651102	0.170523959
ENSGALG00010007455	0.211398908	0.124336124
ENSGALG00010007457	0.601638543	0.246294479
ENSGALG00010007459	0.831573534	0.274556599
ENSGALG00010007461	0.068501264	0.199097315
ENSGALG00010007462	0.554045354	0.324672115
ENSGALG00010007464	0.951878837	0.331018919
ENSGALG00010007465	0.930352403	0.412781429
ENSGALG00010007467	0.990873288	0.320484436

ENSGALG00010007472	0.030198314	0.147519421
ENSGALG00010007473	0.274687811	0.538498988
ENSGALG00010007474	0.285272007	0.109169955
ENSGALG00010007477	0.066462015	0.269260472
ENSGALG00010007479	0.930044612	0.217572939
ENSGALG00010007480	0.967939359	0.346514694
ENSGALG00010007485	0.466828826	0.147376003
ENSGALG00010007490	0.266137114	0.107784983
ENSGALG00010007494	0.673629099	0.281454579
ENSGALG00010007495	0.982715308	0.290661235
ENSGALG00010007496	0.944825312	0.405660866
ENSGALG00010007497	0.8244456	0.317706725
ENSGALG00010007500	0.50495002	0.142878481
ENSGALG00010007501	0.394494248	0.128304162
ENSGALG00010007502	0.393291196	0.091976827
ENSGALG00010007503	0.138339906	0.466345147
ENSGALG00010007505	0.748424683	0.1779889
ENSGALG00010007506	0.088429427	0.031370352
ENSGALG00010007510	0.319058084	0.254756161
ENSGALG00010007513	0.929772326	0.311238852
ENSGALG00010007514	0.712358833	0.250462655
ENSGALG00010007515	0.705543674	0.434611332
ENSGALG00010007517	0.292362493	0.105019933
ENSGALG00010007519	0.506348141	0.39842206
ENSGALG00010007520	0.216433634	0.680598293
ENSGALG00010007521	0.986442907	0.331371573
ENSGALG00010007523	0.12185701	0.189684155
ENSGALG00010007525	0.205739277	0.090549788
ENSGALG00010007528	0.394927857	0.119113854
ENSGALG00010007529	0.983630295	0.371358382
ENSGALG00010007532	0.900524743	0.358624888
ENSGALG00010007536	0.068505756	0.078327733
ENSGALG00010007537	0.308309528	0.074072042
ENSGALG00010007539	0.717353879	0.280887
ENSGALG00010007541	0.048532643	0.007975711
ENSGALG00010007542	0.814400347	0.265908891
ENSGALG00010007544	0.46578975	0.385327486
ENSGALG00010007546	0.545878007	0.150610418
ENSGALG00010007547	0.8639403	0.356091594
ENSGALG00010007548	0.74544362	0.054050581
ENSGALG00010007549	0.982172462	0.250211317
ENSGALG00010007550	0.41485031	0.207418775
ENSGALG00010007551	0.943522539	0.287811132
ENSGALG00010007555	0.975719098	0.336356738
ENSGALG00010007563	0.34081212	0.34730063
ENSGALG00010007564	0.286231124	0.564253946
ENSGALG00010007565	0.554076183	0.377735479
ENSGALG00010007566	0.881693378	0.320253539
ENSGALG00010007567	0.895564775	0.342666723
ENSGALG00010007569	0.220728113	0.125579138
ENSGALG00010007570	0.570740473	0.163320661
ENSGALG00010007571	0.487434544	0.155085468
ENSGALG00010007572	0.799086525	0.264718347
ENSGALG00010007573	0.336850924	0.316322825
ENSGALG00010007575	0.322714299	0.521218221
ENSGALG00010007576	0.577035972	0.248778849

ENSGALG00010007577	0.958958797	0.387233724
ENSGALG00010007578	0.13263007	0.193629681
ENSGALG00010007579	0.857043365	0.245531199
ENSGALG00010007580	0.943340721	0.172740367
ENSGALG00010007581	0.979665193	0.254278351
ENSGALG00010007582	0.265558089	0.028821141
ENSGALG00010007584	0.679736064	0.4191066
ENSGALG00010007585	0.275115332	0.306639425
ENSGALG00010007586	0.885478567	0.323677491
ENSGALG00010007588	0.516281553	0.387705629
ENSGALG00010007589	0.046309987	0.171647964
ENSGALG00010007590	0.711035833	0.185678435
ENSGALG00010007592	0.262641434	0.175697698
ENSGALG00010007593	0.87609114	0.249293366
ENSGALG00010007594	0.677745834	0.219606546
ENSGALG00010007595	0.754101961	0.323137102
ENSGALG00010007596	0.768045365	0.268048115
ENSGALG00010007597	0.161208566	0.124999242
ENSGALG00010007601	0.700368389	0.303977012
ENSGALG00010007603	0.936771296	0.2992622
ENSGALG00010007604	0.513417396	0.286379319
ENSGALG00010007605	0.547076368	0.194424191
ENSGALG00010007607	0.296272339	0.491563762
ENSGALG00010007609	0.386863047	0.139229068
ENSGALG00010007610	0.493098486	0.110165594
ENSGALG00010007612	0.391097471	0.144336359
ENSGALG00010007613	0.680602791	0.222715696
ENSGALG00010007614	0.623981713	0.432081756
ENSGALG00010007615	0.707719723	0.22643292
ENSGALG00010007616	0.67142957	0.26194615
ENSGALG00010007619	0.579986427	0.083908469
ENSGALG00010007620	0.254205923	0.228062898
ENSGALG00010007621	0.015345754	0.043558471
ENSGALG00010007622	0.665854192	0.586523538
ENSGALG00010007624	0.826307616	0.144272508
ENSGALG00010007625	0.968904746	0.272200465
ENSGALG00010007626	0.611566647	0.161696034
ENSGALG00010007627	0.943050153	0.269103403
ENSGALG00010007628	0.842596956	0.344046049
ENSGALG00010007630	0.362381627	0.024884518
ENSGALG00010007631	0.526828396	0.2550782
ENSGALG00010007632	0.569684023	0.281034047
ENSGALG00010007633	0.490624959	0.021021549
ENSGALG00010007634	0.949667091	0.318423859
ENSGALG00010007635	0.003243725	0.177812847
ENSGALG00010007636	0.384670529	0.365016543
ENSGALG00010007637	0.33777847	0.133444889
ENSGALG00010007638	0.698645085	0.167277175
ENSGALG00010007640	0.988477989	0.376218188
ENSGALG00010007642	0.300109973	0.174683106
ENSGALG00010007643	0.5399183	0.07839718
ENSGALG00010007645	0.174736002	0.221328073
ENSGALG00010007649	0.345702106	0.06199354
ENSGALG00010007650	0.599617272	0.079931667
ENSGALG00010007651	0.944343661	0.18231755
ENSGALG00010007653	0.579983744	0.146492439

ENSGALG00010007654	0.720031381	0.243640669
ENSGALG00010007655	0.217976006	0.301193524
ENSGALG00010007656	0.944609554	0.307742679
ENSGALG00010007658	0.649964919	0.305111406
ENSGALG00010007659	0.88693596	0.028649057
ENSGALG00010007660	0.987513541	0.371947408
ENSGALG00010007661	0.287562285	0.365719592
ENSGALG00010007662	0.867467113	0.02271483
ENSGALG00010007663	0.927914832	0.345808753
ENSGALG00010007664	0.956840244	0.338317343
ENSGALG00010007666	0.757930359	0.253399301
ENSGALG00010007668	0.312802863	0.269991217
ENSGALG00010007669	0.385953062	0.133261917
ENSGALG00010007671	0.94357154	0.262725039
ENSGALG00010007672	0.950413146	0.476645303
ENSGALG00010007673	0.558546297	0.115329526
ENSGALG00010007678	0.867431274	0.36731114
ENSGALG00010007679	0.952795276	0.333062097
ENSGALG00010007680	0.228761856	0.415214082
ENSGALG00010007683	0.313956645	0.130146002
ENSGALG00010007686	0.388534291	0.330847295
ENSGALG00010007688	0.262922434	0.138601464
ENSGALG00010007691	0.134692884	0.311757208
ENSGALG00010007692	0.844854404	0.214077082
ENSGALG00010007695	0.347737515	0.045178685
ENSGALG00010007696	0.576224561	0.243582763
ENSGALG00010007697	0.533197418	0.156567823
ENSGALG00010007698	0.272389393	0.046079649
ENSGALG00010007699	0.577742306	0.053508435
ENSGALG00010007700	0.479262553	0.144177318
ENSGALG00010007701	0.472731913	0.10040291
ENSGALG00010007702	0.200994087	0.16470463
ENSGALG00010007703	0.033179125	0.179639579
ENSGALG00010007704	0.354984028	0.356984247
ENSGALG00010007705	0.617338842	0.147469657
ENSGALG00010007707	0.95486257	0.330762801
ENSGALG00010007708	0.977750608	0.332934537
ENSGALG00010007709	0.644816436	0.028774268
ENSGALG00010007710	0.826761421	0.190941712
ENSGALG00010007712	0.908920926	0.265894522
ENSGALG00010007713	0.912488079	0.252184891
ENSGALG00010007718	0.408177236	0.304504012
ENSGALG00010007719	0.41227023	0.031837949
ENSGALG00010007721	0.904909975	0.587363463
ENSGALG00010007723	0.448953241	0.357398828
ENSGALG00010007724	0.844597411	0.092890715
ENSGALG00010007727	0.685609513	0.118631971
ENSGALG00010007730	0.934218209	0.430836262
ENSGALG00010007731	0.730227506	0.328015971
ENSGALG00010007734	0.145065728	0.186475454
ENSGALG00010007739	0.4576733	0.097565236
ENSGALG00010007741	0.957497381	0.4234278
ENSGALG00010007748	0.567184382	0.224913536
ENSGALG00010007751	0.972897459	0.37193886
ENSGALG00010007752	0.700348977	0.096576503
ENSGALG00010007753	0.950375978	0.382757593

ENSGALG00010007755	0.031885131	0.218976498
ENSGALG00010007756	0.10756773	0.15878395
ENSGALG00010007758	0.953785424	0.263858327
ENSGALG00010007759	0.994977544	0.335475957
ENSGALG00010007760	0.016813969	0.1840436
ENSGALG00010007761	0.758500163	0.028567891
ENSGALG00010007762	0.874289691	0.11892508
ENSGALG00010007763	0.927860449	0.235758552
ENSGALG00010007764	0.11758128	0.23184693
ENSGALG00010007765	0.297956472	0.220467392
ENSGALG00010007766	0.693118207	0.100222836
ENSGALG00010007767	0.308371994	0.339084058
ENSGALG00010007768	0.87330332	0.232818846
ENSGALG00010007769	0.939951218	0.275696321
ENSGALG00010007770	0.662639212	0.272920127
ENSGALG00010007771	0.293907584	0.147174991
ENSGALG00010007774	0.197106861	0.158968576
ENSGALG00010007775	0.508410985	0.359662118
ENSGALG00010007776	0.943612342	0.341883032
ENSGALG00010007777	0.938426125	0.370450974
ENSGALG00010007779	0.702918403	0.213239038
ENSGALG00010007780	0.934503608	0.375806982
ENSGALG00010007781	0.434469745	0.16547294
ENSGALG00010007782	0.456399251	0.11287433
ENSGALG00010007783	0.798761598	0.221880023
ENSGALG00010007784	0.686080886	0.332361056
ENSGALG00010007786	0.964564926	0.43817223
ENSGALG00010007787	0.39269743	0.258005655
ENSGALG00010007788	0.705548599	0.310979481
ENSGALG00010007789	0.265667322	0.157928229
ENSGALG00010007792	0.872834051	0.378283408
ENSGALG00010007793	0.340226296	0.222832747
ENSGALG00010007795	0.74179348	0.472229222
ENSGALG00010007796	0.913892237	0.184349769
ENSGALG00010007797	0.481688014	0.023718441
ENSGALG00010007798	0.931138147	0.275297559
ENSGALG00010007799	0.728270292	0.300567617
ENSGALG00010007800	0.934727316	0.260294159
ENSGALG00010007801	0.962361894	0.267931154
ENSGALG00010007802	0.591654779	0.503879988
ENSGALG00010007803	0.98869251	0.299068521
ENSGALG00010007804	0.208550923	0.676301294
ENSGALG00010007805	0.353514384	0.42379427
ENSGALG00010007806	0.868898801	0.466404897
ENSGALG00010007807	0.364065872	0.140159288
ENSGALG00010007809	0.871452564	0.327284946
ENSGALG00010007811	0.531957233	0.081190198
ENSGALG00010007812	0.155064993	0.10498163
ENSGALG00010007817	0.878532007	0.332057285
ENSGALG00010007818	0.647851328	0.253208857
ENSGALG00010007819	0.162650696	0.01114347
ENSGALG00010007821	0.350044542	0.037157583
ENSGALG00010007824	0.386114539	0.074004299
ENSGALG00010007826	0.378577426	0.095614252
ENSGALG00010007830	0.967662892	0.342984326
ENSGALG00010007831	0.386940806	0.06873218

ENSGALG00010007835	0.9260776	0.353289968
ENSGALG00010007841	0.394927857	0.119113854
ENSGALG00010007846	0.33314852	0.227716081
ENSGALG00010007847	0.958861052	0.256866082
ENSGALG00010007849	0.991859484	0.398030145
ENSGALG00010007850	0.947373001	0.201134699
ENSGALG00010007851	0.946765999	0.305405911
ENSGALG00010007852	0.453340525	0.019342708
ENSGALG00010007853	0.968724859	0.338091806
ENSGALG00010007854	0.954539022	0.331731752
ENSGALG00010007856	0.934613832	0.182450305
ENSGALG00010007857	0.71768638	0.631858088
ENSGALG00010007859	0.472827009	0.029892722
ENSGALG00010007860	0.938982954	0.299610818
ENSGALG00010007861	0.217461377	0.487387806
ENSGALG00010007862	0.175817499	0.599094588
ENSGALG00010007863	0.888452867	0.300680998
ENSGALG00010007865	0.502597554	0.114547379
ENSGALG00010007866	0.514515547	0.017351798
ENSGALG00010007867	0.555423871	0.166992143
ENSGALG00010007868	0.944379275	0.348065378
ENSGALG00010007869	0.974057804	0.293475662
ENSGALG00010007870	0.631074877	0.181900586
ENSGALG00010007871	0.951036189	0.351858671
ENSGALG00010007872	0.470786851	0.189329773
ENSGALG00010007873	0.482053965	0.273772015
ENSGALG00010007874	0.845420879	0.442479237
ENSGALG00010007875	0.917532997	0.285152167
ENSGALG00010007876	0.498838275	0.165355112
ENSGALG00010007877	0.978863444	0.336457957
ENSGALG00010007878	0.953175319	0.379819983
ENSGALG00010007879	0.589229123	0.055242411
ENSGALG00010007880	0.964304542	0.424755087
ENSGALG00010007881	0.0923187	0.150253598
ENSGALG00010007882	0.90257023	0.483163305
ENSGALG00010007883	0.626435054	0.380797566
ENSGALG00010007884	0.138272065	0.007471603
ENSGALG00010007885	0.523092574	0.223206692
ENSGALG00010007887	0.286043662	0.197540989
ENSGALG00010007888	0.886555818	0.24240996
ENSGALG00010007889	0.988941148	0.29246925
ENSGALG00010007890	0.701467535	0.05979234
ENSGALG00010007891	0.127140164	0.010514772
ENSGALG00010007892	0.235570677	0.194409353
ENSGALG00010007893	0.512306622	0.20130008
ENSGALG00010007894	0.983402027	0.352945098
ENSGALG00010007895	0.980292093	0.327598235
ENSGALG00010007896	0.985372471	0.305966455
ENSGALG00010007897	0.835534792	0.530104656
ENSGALG00010007898	0.922441845	0.118024111
ENSGALG00010007899	0.069228061	0.054699326
ENSGALG00010007900	0.93199602	0.293193994
ENSGALG00010007901	0.393516822	0.238074982
ENSGALG00010007902	0.994490482	0.322020426
ENSGALG00010007903	0.205584867	0.026776481
ENSGALG00010007904	0.355840211	0.126688245

ENSGALG00010007905	0.082092945	0.021344404
ENSGALG00010007906	0.082689054	0.100862593
ENSGALG00010007908	0.285179533	0.121954912
ENSGALG00010007909	0.569662204	0.243063501
ENSGALG00010007910	0.034335122	0.18357704
ENSGALG00010007911	0.942387648	0.414808864
ENSGALG00010007912	0.409974878	0.096295837
ENSGALG00010007913	0.335707684	0.194178786
ENSGALG00010007914	0.900194964	0.143202046
ENSGALG00010007915	0.739360325	0.267564144
ENSGALG00010007916	0.676122784	0.092118264
ENSGALG00010007917	0.975357039	0.26893358
ENSGALG00010007919	0.085050712	0.180185983
ENSGALG00010007920	0.835242699	0.180127099
ENSGALG00010007921	0.957536498	0.257128654
ENSGALG00010007922	0.495590222	0.058264282
ENSGALG00010007923	0.110933706	0.181726464
ENSGALG00010007924	0.953882627	0.2979934
ENSGALG00010007925	0.916962531	0.342078323
ENSGALG00010007926	0.718339996	0.156774384
ENSGALG00010007927	0.976493687	0.303431176
ENSGALG00010007928	0.387591403	0.030397751
ENSGALG00010007929	0.993011415	0.328943794
ENSGALG00010007930	0.718391575	0.456914213
ENSGALG00010007931	0.645434467	0.202836639
ENSGALG00010007932	0.579959408	0.231826899
ENSGALG00010007934	0.961144566	0.2215051
ENSGALG00010007935	0.989375796	0.331005162
ENSGALG00010007936	0.977360184	0.355057737
ENSGALG00010007937	0.559220734	0.078746946
ENSGALG00010007938	0.943234149	0.303839227
ENSGALG00010007940	0.76700228	0.450380127
ENSGALG00010007941	0.861335459	0.172332103
ENSGALG00010007942	0.925854828	0.280086521
ENSGALG00010007943	0.958355056	0.195522072
ENSGALG00010007944	0.817674445	0.218078499
ENSGALG00010007945	0.552551209	0.15040742
ENSGALG00010007946	0.808278069	0.211618139
ENSGALG00010007948	0.103699037	0.06317715
ENSGALG00010007950	0.282976025	0.145658897
ENSGALG00010007951	0.922237957	0.305888117
ENSGALG00010007952	0.077320274	0.040909199
ENSGALG00010007953	0.231025261	0.327597124
ENSGALG00010007954	0.718640168	0.280585663
ENSGALG00010007955	0.85376497	0.342812293
ENSGALG00010007956	0.956715928	0.302999403
ENSGALG00010007957	0.996418431	0.30879497
ENSGALG00010007958	0.840851273	0.221573786
ENSGALG00010007960	0.560135843	0.303166045
ENSGALG00010007962	0.246621768	0.122491293
ENSGALG00010007963	0.982987279	0.2564181
ENSGALG00010007964	0.523090662	0.09479471
ENSGALG00010007965	0.982920076	0.310123579
ENSGALG00010007966	0.947344342	0.474868781
ENSGALG00010007968	0.953594979	0.255393854
ENSGALG00010007970	0.838575625	0.328862035

ENSGALG00010007972	0.847742722	0.344480772
ENSGALG00010007973	0.675138493	0.089834363
ENSGALG00010007975	0.196308861	0.046809973
ENSGALG00010007976	0.373998094	0.048098552
ENSGALG00010007977	0.996916911	0.335268476
ENSGALG00010007978	0.171443105	0.112276211
ENSGALG00010007979	0.927996583	0.315564424
ENSGALG00010007980	0.960135983	0.331574839
ENSGALG00010007981	0.517288297	0.192881547
ENSGALG00010007982	0.966168549	0.412280989
ENSGALG00010007983	0.984852798	0.378179405
ENSGALG00010007984	0.79010193	0.214775302
ENSGALG00010007985	0.981553379	0.310797718
ENSGALG00010007986	0.673766976	0.368800279
ENSGALG00010007987	0.812138897	0.212049248
ENSGALG00010007988	0.547581046	0.21697919
ENSGALG00010007989	0.969509281	0.297403936
ENSGALG00010007991	0.748959513	0.356632033
ENSGALG00010007992	0.681213399	0.19021192
ENSGALG00010007993	0.884072302	0.33632199
ENSGALG00010007994	0.916581196	0.318902284
ENSGALG00010007995	0.938463819	0.218889877
ENSGALG00010007996	0.923592134	0.197857825
ENSGALG00010007997	0.557689643	0.158494812
ENSGALG00010007998	0.824942024	0.323705514
ENSGALG00010007999	0.940667894	0.220049837
ENSGALG00010008000	0.619381692	0.479152553
ENSGALG00010008001	0.959523829	0.29783018
ENSGALG00010008002	0.017380207	0.502070766
ENSGALG00010008004	0.906373859	0.272728196
ENSGALG00010008005	0.480826355	0.16307731
ENSGALG00010008006	0.100232018	0.227182986
ENSGALG00010008007	0.756068546	0.275079408
ENSGALG00010008008	0.979749315	0.307932664
ENSGALG00010008009	0.988481138	0.313159242
ENSGALG00010008010	0.95827044	0.416740099
ENSGALG00010008011	0.885263024	0.202031389
ENSGALG00010008012	0.942132791	0.299700129
ENSGALG00010008013	0.813447456	0.112294134
ENSGALG00010008014	0.893638692	0.239817821
ENSGALG00010008015	0.934649061	0.339729984
ENSGALG00010008016	0.68150244	0.048914642
ENSGALG00010008017	0.017988535	0.066149537
ENSGALG00010008018	0.380435244	0.217241544
ENSGALG00010008019	0.829924485	0.409079627
ENSGALG00010008020	0.933652727	0.221575928
ENSGALG00010008021	0.325694796	0.462134608
ENSGALG00010008022	0.122995325	0.116113119
ENSGALG00010008023	0.774592867	0.246427652
ENSGALG00010008024	0.628103151	0.318016884
ENSGALG00010008025	0.114168306	0.141162836
ENSGALG00010008026	0.112560104	0.038188023
ENSGALG00010008027	0.543074262	0.255837184
ENSGALG00010008028	0.990704541	0.30867794
ENSGALG00010008029	0.96600417	0.385073859
ENSGALG00010008030	0.229289636	0.405972883

ENSGALG00010008031	0.934961396	0.361369297
ENSGALG00010008032	0.969093366	0.419183663
ENSGALG00010008033	0.933081321	0.364556291
ENSGALG00010008034	0.803134471	0.220160219
ENSGALG00010008035	0.765997687	0.443436968
ENSGALG00010008036	0.830686302	0.359099661
ENSGALG00010008037	0.49944091	0.29268137
ENSGALG00010008038	0.954191533	0.347680037
ENSGALG00010008039	0.26730295	0.360220415
ENSGALG00010008041	0.924503447	0.61249834
ENSGALG00010008042	0.360144738	0.298645978
ENSGALG00010008043	0.569938556	0.102353498
ENSGALG00010008044	0.789793176	0.241333694
ENSGALG00010008045	0.984828669	0.248695123
ENSGALG00010008046	0.928292478	0.21199164
ENSGALG00010008047	0.925966407	0.201217457
ENSGALG00010008048	0.317181506	0.084381918
ENSGALG00010008049	0.194621834	0.096222222
ENSGALG00010008050	0.11798188	0.021789302
ENSGALG00010008051	0.4679859	0.581900856
ENSGALG00010008052	0.753301532	0.173506995
ENSGALG00010008053	0.563541407	0.235649438
ENSGALG00010008054	0.869099346	0.225878896
ENSGALG00010008055	0.11900953	0.213026117
ENSGALG00010008056	0.741944976	0.384192001
ENSGALG00010008057	0.072275069	0.346198869
ENSGALG00010008058	0.928089368	0.257135382
ENSGALG00010008059	0.401306216	0.061753914
ENSGALG00010008061	0.93448505	0.302536181
ENSGALG00010008062	0.711794651	0.060349899
ENSGALG00010008063	0.787562713	0.485253003
ENSGALG00010008064	0.986230876	0.332340912
ENSGALG00010008065	0.76883502	0.058471932
ENSGALG00010008066	0.815810845	0.339561476
ENSGALG00010008067	0.609943352	0.246661062
ENSGALG00010008069	0.149231952	0.208920393
ENSGALG00010008071	0.222517999	0.582064403
ENSGALG00010008072	0.89131846	0.193462676
ENSGALG00010008073	0.320958891	0.014191627
ENSGALG00010008074	0.800956965	0.226670497
ENSGALG00010008075	0.904964062	0.433358402
ENSGALG00010008076	0.085869065	0.29826344
ENSGALG00010008078	0.856688645	0.257379185
ENSGALG00010008079	0.663317193	0.138160511
ENSGALG00010008081	0.903220057	0.331071557
ENSGALG00010008083	0.895692314	0.209269642
ENSGALG00010008084	0.996918731	0.341829159
ENSGALG00010008086	0.976042589	0.291835038
ENSGALG00010008087	0.95323892	0.24138535
ENSGALG00010008088	0.945026079	0.369981222
ENSGALG00010008091	0.878904909	0.162526972
ENSGALG00010008092	0.914751144	0.385941518
ENSGALG00010008093	0.964229193	0.36318022
ENSGALG00010008094	0.949852973	0.320410973
ENSGALG00010008095	0.381415582	0.158761082
ENSGALG00010008096	0.620966285	0.049823672

ENSGALG00010008097	0.613023567	0.206644295
ENSGALG00010008098	0.720861043	0.041646975
ENSGALG00010008099	0.989748971	0.37033063
ENSGALG00010008100	0.648083365	0.044677035
ENSGALG00010008101	0.961016041	0.1926886
ENSGALG00010008102	0.19832926	0.209269862
ENSGALG00010008103	0.80855567	0.314798265
ENSGALG00010008104	0.913990446	0.232272779
ENSGALG00010008105	0.946819693	0.324109876
ENSGALG00010008106	0.366466356	0.098299268
ENSGALG00010008107	0.04722783	0.19753533
ENSGALG00010008108	0.644716018	0.211194579
ENSGALG00010008109	0.58695361	0.198097752
ENSGALG00010008110	0.947356851	0.325439143
ENSGALG00010008111	0.175044389	0.2492851
ENSGALG00010008112	0.001230292	0.202444066
ENSGALG00010008113	0.087243134	0.141035895
ENSGALG00010008115	0.861401	0.321469818
ENSGALG00010008116	0.980980674	0.246404518
ENSGALG00010008117	0.721824354	0.306198203
ENSGALG00010008118	0.628324832	0.162348059
ENSGALG00010008119	0.150003191	0.313578112
ENSGALG00010008120	0.8660574	0.183854439
ENSGALG00010008121	0.951002206	0.321973637
ENSGALG00010008123	0.03423594	0.010419442
ENSGALG00010008124	0.423469538	0.042895477
ENSGALG00010008126	0.888223469	0.520094273
ENSGALG00010008127	0.776540077	0.2198111
ENSGALG00010008128	0.46872673	0.315694117
ENSGALG00010008129	0.880400894	0.396964398
ENSGALG00010008130	0.339154281	0.129365616
ENSGALG00010008132	0.994204331	0.324748356
ENSGALG00010008133	0.365799671	0.267454281
ENSGALG00010008134	0.957849159	0.359229269
ENSGALG00010008135	0.864550625	0.466704023
ENSGALG00010008136	0.831677555	0.120067434
ENSGALG00010008139	0.415539217	0.230683478
ENSGALG00010008140	0.097445731	0.211366078
ENSGALG00010008141	0.859750721	0.223145314
ENSGALG00010008142	0.884937484	0.454248241
ENSGALG00010008143	0.937769144	0.34721326
ENSGALG00010008144	0.835272174	0.276886669
ENSGALG00010008145	0.885608416	0.163036046
ENSGALG00010008146	0.942066127	0.320495735
ENSGALG00010008147	0.919169603	0.173828395
ENSGALG00010008148	0.948058797	0.255292056
ENSGALG00010008149	0.814067472	0.427237571
ENSGALG00010008150	0.316378889	0.052481458
ENSGALG00010008151	0.97171438	0.321243789
ENSGALG00010008152	0.215954767	0.274766132
ENSGALG00010008153	0.877774516	0.30111448
ENSGALG00010008154	0.070187365	0.101549555
ENSGALG00010008155	0.621438502	0.358198644
ENSGALG00010008156	0.445871714	0.218926265
ENSGALG00010008157	0.981894068	0.307797012
ENSGALG00010008158	0.951006099	0.302805749

ENSGALG00010008159	0.917802425	0.168178083
ENSGALG00010008160	0.484687057	0.021172625
ENSGALG00010008161	0.917631966	0.327796026
ENSGALG00010008162	0.893692958	0.173066277
ENSGALG00010008163	0.03392138	0.257055131
ENSGALG00010008164	0.583837899	0.516108946
ENSGALG00010008165	0.923343531	0.190232353
ENSGALG00010008166	0.677366662	0.034375979
ENSGALG00010008167	0.926908644	0.324935974
ENSGALG00010008168	0.996702936	0.330104367
ENSGALG00010008169	0.763347889	0.085534867
ENSGALG00010008170	0.794634063	0.304187265
ENSGALG00010008171	0.817519967	0.321988652
ENSGALG00010008172	0.418800386	0.231080396
ENSGALG00010008173	0.620520442	0.109527361
ENSGALG00010008174	0.96610658	0.367793222
ENSGALG00010008175	0.398699747	0.271679994
ENSGALG00010008176	0.955817672	0.228518051
ENSGALG00010008177	0.988961812	0.291113443
ENSGALG00010008178	0.066984897	0.274979709
ENSGALG00010008179	0.897158359	0.372648371
ENSGALG00010008180	0.376801876	0.329852003
ENSGALG00010008181	0.938077753	0.2087234
ENSGALG00010008182	0.97688494	0.351835392
ENSGALG00010008183	0.434211084	0.217478478
ENSGALG00010008184	0.93984524	0.15916154
ENSGALG00010008185	0.859971788	0.472865835
ENSGALG00010008186	0.958990417	0.3458583
ENSGALG00010008187	0.956912213	0.359922879
ENSGALG00010008188	0.977908816	0.337091858
ENSGALG00010008189	0.744444587	0.257031937
ENSGALG00010008190	0.801537573	0.2022804
ENSGALG00010008191	0.519777458	0.341465257
ENSGALG00010008192	0.740271281	0.007636016
ENSGALG00010008193	0.018494712	0.235260517
ENSGALG00010008194	0.922615934	0.394382295
ENSGALG00010008195	0.974323552	0.311724078
ENSGALG00010008196	0.600149308	0.255428276
ENSGALG00010008197	0.976395196	0.198997965
ENSGALG00010008198	0.605071765	0.17325594
ENSGALG00010008199	0.930747561	0.294265056
ENSGALG00010008200	0.619569472	0.418679056
ENSGALG00010008201	0.903354669	0.292520273
ENSGALG00010008202	0.944465429	0.243882182
ENSGALG00010008203	0.314792918	0.170923543
ENSGALG00010008204	0.805851629	0.257609165
ENSGALG00010008205	0.942892151	0.311763203
ENSGALG00010008206	0.542795175	0.196252614
ENSGALG00010008207	0.902137206	0.299066708
ENSGALG00010008208	0.979488803	0.27306084
ENSGALG00010008209	0.85459685	0.274721908
ENSGALG00010008210	0.669513857	0.603098273
ENSGALG00010008211	0.926207378	0.407129842
ENSGALG00010008212	0.672666981	0.044021808
ENSGALG00010008213	0.844161189	0.194918499
ENSGALG00010008214	0.403982221	0.184759805

ENSGALG00010008215	0.992978013	0.346353725
ENSGALG00010008216	0.776925541	0.407115339
ENSGALG00010008217	0.984427245	0.33577768
ENSGALG00010008218	0.948889631	0.291327865
ENSGALG00010008219	0.722832283	0.204003692
ENSGALG00010008220	0.488510741	0.323093055
ENSGALG00010008221	0.920071191	0.328179595
ENSGALG00010008222	0.993619718	0.317856782
ENSGALG00010008223	0.632136322	0.133234149
ENSGALG00010008224	0.964721336	0.340312709
ENSGALG00010008225	0.229617119	0.080150254
ENSGALG00010008226	0.208502343	0.311558725
ENSGALG00010008227	0.512013325	0.051712807
ENSGALG00010008228	0.412278842	0.06058923
ENSGALG00010008229	0.715692051	0.205977941
ENSGALG00010008230	0.486771277	0.41275408
ENSGALG00010008231	0.926188396	0.372952434
ENSGALG00010008232	0.974308149	0.324742612
ENSGALG00010008233	0.89160385	0.462705008
ENSGALG00010008234	0.415133953	0.397863136
ENSGALG00010008235	0.752343428	0.276836265
ENSGALG00010008236	0.800476776	0.335882182
ENSGALG00010008237	0.385044915	0.110616185
ENSGALG00010008238	0.163185093	0.230509085
ENSGALG00010008240	0.369918977	0.172515236
ENSGALG00010008241	0.51037783	0.279665214
ENSGALG00010008242	0.980609096	0.327625001
ENSGALG00010008243	0.966167084	0.285816576
ENSGALG00010008245	0.896644774	0.291889431
ENSGALG00010008246	0.484378003	0.222042391
ENSGALG00010008247	0.860958583	0.246226162
ENSGALG00010008248	0.363373896	0.082875218
ENSGALG00010008250	0.973222613	0.388775962
ENSGALG00010008251	0.855250406	0.353147207
ENSGALG00010008252	0.97226252	0.273371462
ENSGALG00010008253	0.297188989	0.027987018
ENSGALG00010008255	0.905899179	0.29476589
ENSGALG00010008257	0.922413824	0.161917489
ENSGALG00010008258	0.917595419	0.28432191
ENSGALG00010008259	0.682758806	0.052794751
ENSGALG00010008260	0.388899421	0.120062276
ENSGALG00010008261	0.727968721	0.466534204
ENSGALG00010008262	0.945633403	0.462239708
ENSGALG00010008263	0.460167639	0.558332402
ENSGALG00010008264	0.550689173	0.033142756
ENSGALG00010008265	0.943033014	0.261857716
ENSGALG00010008266	0.961526481	0.335714467
ENSGALG00010008267	0.457418933	0.016998845
ENSGALG00010008268	0.866830249	0.345961138
ENSGALG00010008270	0.593956601	0.113216994
ENSGALG00010008272	0.925661861	0.441171468
ENSGALG00010008273	0.825738269	0.284794084
ENSGALG00010008274	0.925482577	0.27962784
ENSGALG00010008275	0.955916778	0.361607487
ENSGALG00010008276	0.968097341	0.323792686
ENSGALG00010008277	0.325928283	0.532659218

ENSGALG00010008278	0.953962855	0.26517295
ENSGALG00010008279	0.631128581	0.311633578
ENSGALG00010008282	0.917303379	0.276902991
ENSGALG00010008283	0.98841633	0.315707621
ENSGALG00010008284	0.873231244	0.327745253
ENSGALG00010008285	0.34842739	0.16036542
ENSGALG00010008286	0.939386589	0.342366624
ENSGALG00010008287	0.461569218	0.026252786
ENSGALG00010008288	0.021308589	0.164876077
ENSGALG00010008289	0.666536235	0.216043203
ENSGALG00010008290	0.69054164	0.311378124
ENSGALG00010008291	0.442435039	0.377660189
ENSGALG00010008292	0.974380384	0.422076998
ENSGALG00010008293	0.542870646	0.253230077
ENSGALG00010008294	0.938683222	0.294979925
ENSGALG00010008295	0.854109521	0.413933906
ENSGALG00010008296	0.96680995	0.296812422
ENSGALG00010008298	0.876607282	0.279022946
ENSGALG00010008299	0.875800928	0.345914751
ENSGALG00010008300	0.233954101	0.286564822
ENSGALG00010008301	0.984555457	0.370136941
ENSGALG00010008302	0.368326215	0.209235812
ENSGALG00010008303	0.896101979	0.408051762
ENSGALG00010008305	0.979891642	0.380219282
ENSGALG00010008307	0.146595897	0.244256602
ENSGALG00010008308	0.983387751	0.291015689
ENSGALG00010008309	0.777742585	0.337125309
ENSGALG00010008310	0.818033603	0.178399392
ENSGALG00010008311	0.301521179	0.009556972
ENSGALG00010008312	0.990119266	0.31386291
ENSGALG00010008313	0.986025824	0.30918363
ENSGALG00010008314	0.428957726	0.035465533
ENSGALG00010008315	0.264239718	0.073427965
ENSGALG00010008316	0.822585372	0.364863333
ENSGALG00010008317	0.796695842	0.412169409
ENSGALG00010008318	0.9666354	0.304269917
ENSGALG00010008319	0.776077002	0.562607796
ENSGALG00010008320	0.854127113	0.259321354
ENSGALG00010008321	0.953126211	0.316516543
ENSGALG00010008323	0.855822354	0.298664502
ENSGALG00010008324	0.935400982	0.232267964
ENSGALG00010008325	0.847096059	0.299861816
ENSGALG00010008327	0.727643851	0.139153889
ENSGALG00010008330	0.842508598	0.195632559
ENSGALG00010008331	0.912353252	0.345026838
ENSGALG00010008332	0.131921507	0.128584882
ENSGALG00010008333	0.836052916	0.172108982
ENSGALG00010008334	0.009863359	0.489102725
ENSGALG00010008335	0.862881988	0.251584163
ENSGALG00010008336	0.959960144	0.317718981
ENSGALG00010008339	0.338686006	0.124826975
ENSGALG00010008340	0.973654735	0.305919237
ENSGALG00010008341	0.875917751	0.241280766
ENSGALG00010008342	0.291762351	0.51093649
ENSGALG00010008343	0.040239282	0.010765275
ENSGALG00010008344	0.471289152	0.173893333

ENSGALG00010008346	0.645845949	0.645467688
ENSGALG00010008347	0.75487337	0.23823989
ENSGALG00010008348	0.074612087	0.077950833
ENSGALG00010008349	0.478721866	0.148947923
ENSGALG00010008350	0.122486999	0.059919539
ENSGALG00010008351	0.925566309	0.378793717
ENSGALG00010008352	0.961296556	0.352736334
ENSGALG00010008354	0.663124921	0.270770704
ENSGALG00010008355	0.986357407	0.339851466
ENSGALG00010008356	0.536930426	0.182357667
ENSGALG00010008358	0.645644515	0.295496237
ENSGALG00010008360	0.330181657	0.352184146
ENSGALG00010008361	0.868169827	0.339694663
ENSGALG00010008362	0.962888587	0.28878513
ENSGALG00010008364	0.509263406	0.123243317
ENSGALG00010008366	0.264342283	0.237837915
ENSGALG00010008367	0.002399406	0.006663862
ENSGALG00010008368	0.562991205	0.256373294
ENSGALG00010008372	0.89583776	0.311616127
ENSGALG00010008373	0.337066473	0.036381618
ENSGALG00010008374	0.401887138	0.178938845
ENSGALG00010008375	0.953465271	0.22111572
ENSGALG00010008376	0.394846913	0.04886467
ENSGALG00010008377	0.987869507	0.283327839
ENSGALG00010008380	0.901823826	0.297871128
ENSGALG00010008381	0.938923608	0.227720262
ENSGALG00010008382	0.625257481	0.094324574
ENSGALG00010008383	0.245700908	0.010899108
ENSGALG00010008385	0.944053541	0.254327316
ENSGALG00010008386	0.951091045	0.249264132
ENSGALG00010008387	0.18719348	0.122453904
ENSGALG00010008388	0.931485782	0.238826966
ENSGALG00010008389	0.861553726	0.237239385
ENSGALG00010008390	0.023279831	0.134374301
ENSGALG00010008391	0.924937076	0.375112047
ENSGALG00010008392	0.966367628	0.261922602
ENSGALG00010008394	0.977350178	0.273637345
ENSGALG00010008395	0.881390685	0.440270077
ENSGALG00010008396	0.169812106	0.388906214
ENSGALG00010008397	0.47773829	0.324016024
ENSGALG00010008399	0.746780809	0.404540979
ENSGALG00010008400	0.775812717	0.260356913
ENSGALG00010008401	0.983856004	0.310910544
ENSGALG00010008403	0.358009916	0.179963489
ENSGALG00010008405	0.953405277	0.306832412
ENSGALG00010008407	0.472922076	0.338563902
ENSGALG00010008408	0.966893742	0.22234897
ENSGALG00010008409	0.978985031	0.275956514
ENSGALG00010008410	0.97506949	0.336081972
ENSGALG00010008412	0.139983943	0.294014143
ENSGALG00010008415	0.455566134	0.136190511
ENSGALG00010008416	0.04078883	0.20203698
ENSGALG00010008417	0.962048017	0.332709886
ENSGALG00010008418	0.942420598	0.327246771
ENSGALG00010008419	0.022881441	0.138844183
ENSGALG00010008422	0.624252897	0.045304355

ENSGALG00010008424	0.960839509	0.32223127
ENSGALG00010008425	0.667431839	0.419956753
ENSGALG00010008426	0.991980264	0.345358761
ENSGALG00010008428	0.762586514	0.090806779
ENSGALG00010008429	0.987551239	0.305099328
ENSGALG00010008431	0.364078712	0.17553523
ENSGALG00010008432	0.271701261	0.072417625
ENSGALG00010008433	0.759628596	0.213261292
ENSGALG00010008437	0.939320037	0.362078465
ENSGALG00010008438	0.884443558	0.157766845
ENSGALG00010008441	0.929071091	0.422642359
ENSGALG00010008442	0.905031888	0.32445949
ENSGALG00010008443	0.936619791	0.285541708
ENSGALG00010008444	0.697984877	0.027258693
ENSGALG00010008447	0.237241526	0.009594709
ENSGALG00010008448	0.923605013	0.450832881
ENSGALG00010008449	0.580666081	0.115480185
ENSGALG00010008450	0.822934834	0.054721465
ENSGALG00010008451	0.70911083	0.00080868
ENSGALG00010008453	0.986038047	0.358674312
ENSGALG00010008455	0.909912835	0.346280866
ENSGALG00010008456	0.950696397	0.272845987
ENSGALG00010008457	0.982915383	0.320013202
ENSGALG00010008459	0.982916279	0.373086646
ENSGALG00010008460	0.705608967	0.222457637
ENSGALG00010008464	0.004399529	0.086172557
ENSGALG00010008466	0.6536709	0.286818172
ENSGALG00010008467	0.189240572	0.04996112
ENSGALG00010008468	0.657209762	0.278627832
ENSGALG00010008469	0.868192503	0.330293214
ENSGALG00010008470	0.761000558	0.290112319
ENSGALG00010008473	0.390615778	0.13423307
ENSGALG00010008474	0.972509878	0.409510743
ENSGALG00010008475	0.639126502	0.38450568
ENSGALG00010008476	0.798824084	0.257808976
ENSGALG00010008479	0.581137707	0.711775215
ENSGALG00010008480	0.491519158	0.111732976
ENSGALG00010008483	0.438816874	0.253738372
ENSGALG00010008484	0.05197934	0.14495833
ENSGALG00010008485	0.230507588	0.31416683
ENSGALG00010008486	0.856918908	0.113965971
ENSGALG00010008488	0.916363785	0.243503343
ENSGALG00010008491	0.71703083	0.063872337
ENSGALG00010008492	0.949618058	0.307442068
ENSGALG00010008493	0.203991683	0.058221925
ENSGALG00010008494	0.295245823	0.130519728
ENSGALG00010008496	0.265558089	0.028821141
ENSGALG00010008497	0.989486678	0.296772997
ENSGALG00010008498	0.991976079	0.306064725
ENSGALG00010008500	0.886712724	0.236796556
ENSGALG00010008501	0.86092593	0.191702837
ENSGALG00010008504	0.632801987	0.273893536
ENSGALG00010008505	0.975628976	0.37891371
ENSGALG00010008509	0.915885973	0.28940429
ENSGALG00010008511	0.522446629	0.160523389
ENSGALG00010008514	0.961781787	0.356608659

ENSGALG00010008515	0.967320567	0.286666527
ENSGALG00010008516	0.206720924	0.053170894
ENSGALG00010008517	0.529255843	0.069610101
ENSGALG00010008518	0.534888567	0.379747017
ENSGALG00010008522	0.884924656	0.34755419
ENSGALG00010008524	0.379306892	0.160819793
ENSGALG00010008527	0.966532752	0.29279665
ENSGALG00010008529	0.496785806	0.096342124
ENSGALG00010008531	0.482497705	0.120500368
ENSGALG00010008532	0.265558089	0.028821141
ENSGALG00010008534	0.94472076	0.327825722
ENSGALG00010008539	0.671188156	0.501160398
ENSGALG00010008541	0.408304034	0.246578583
ENSGALG00010008542	0.884304796	0.315272437
ENSGALG00010008543	0.76634944	0.275111923
ENSGALG00010008545	0.266137114	0.107784983
ENSGALG00010008546	0.301015495	0.244106305
ENSGALG00010008547	0.042991981	0.309911724
ENSGALG00010008549	0.075305495	0.173273445
ENSGALG00010008551	0.295598961	0.069390962
ENSGALG00010008553	0.409436406	0.157934013
ENSGALG00010008554	0.302909646	0.324236094
ENSGALG00010008556	0.597100545	0.237620275
ENSGALG00010008558	0.198227915	0.250666602
ENSGALG00010008561	0.964374771	0.329278832
ENSGALG00010008562	0.753258371	0.056214588
ENSGALG00010008564	0.535461441	0.377569143
ENSGALG00010008566	0.546120817	0.196121746
ENSGALG00010008567	0.10532374	0.617624974
ENSGALG00010008568	0.590420055	0.297346176
ENSGALG00010008574	0.067084919	0.130961
ENSGALG00010008575	0.097600593	0.180811099
ENSGALG00010008578	0.266137114	0.107784983
ENSGALG00010008579	0.218648951	0.113340634
ENSGALG00010008580	0.861116071	0.396511411
ENSGALG00010008584	0.586815662	0.36998166
ENSGALG00010008586	0.163247233	0.166988212
ENSGALG00010008588	0.684098561	0.266296669
ENSGALG00010008592	0.673159528	0.209471113
ENSGALG00010008596	0.974064096	0.358661319
ENSGALG00010008597	0.0772958	0.130599182
ENSGALG00010008598	0.787791642	0.287949079
ENSGALG00010008600	0.342343757	0.168268007
ENSGALG00010008601	0.266137114	0.107784983
ENSGALG00010008604	0.433997862	0.100788281
ENSGALG00010008618	0.828137502	0.055486726
ENSGALG00010008619	0.279979046	0.096064068
ENSGALG00010008620	0.220110391	0.123050157
ENSGALG00010008622	0.189240572	0.04996112
ENSGALG00010008623	0.718761915	0.286178017
ENSGALG00010008627	0.401639624	0.101384476
ENSGALG00010008628	0.372815743	0.037819372
ENSGALG00010008634	0.151250299	0.401354686
ENSGALG00010008636	0.967129985	0.255196474
ENSGALG00010008640	0.490870766	0.189406675
ENSGALG00010008641	0.467661517	0.293794448

ENSGALG00010008648	0.270451009	0.090541667
ENSGALG00010008649	0.334482024	0.206505972
ENSGALG00010008652	0.861692707	0.343512516
ENSGALG00010008653	0.843990655	0.359422417
ENSGALG00010008656	0.96454596	0.260324841
ENSGALG00010008658	0.845538318	0.421679178
ENSGALG00010008661	0.387374433	0.109360451
ENSGALG00010008664	0.996886503	0.335458603
ENSGALG00010008665	0.493482049	0.177216653
ENSGALG00010008666	0.931322395	0.210963535
ENSGALG00010008668	0.385364039	0.072330531
ENSGALG00010008669	0.218289073	0.253940806
ENSGALG00010008671	0.277458611	0.137642943
ENSGALG00010008672	0.381238997	0.166698879
ENSGALG00010008674	0.839792709	0.195591719
ENSGALG00010008675	0.270451009	0.090541667
ENSGALG00010008676	0.907174091	0.178705703
ENSGALG00010008677	0.864577527	0.303004378
ENSGALG00010008678	0.173587358	0.223125021
ENSGALG00010008679	0.06217773	0.089667239
ENSGALG00010008680	0.390466756	0.106163828
ENSGALG00010008681	0.55543884	0.566168681
ENSGALG00010008682	0.262922434	0.138601464
ENSGALG00010008683	0.798621746	0.17043442
ENSGALG00010008685	0.390466756	0.106163828
ENSGALG00010008686	0.480679297	0.003136748
ENSGALG00010008688	0.017710855	0.378042089
ENSGALG00010008690	0.930423171	0.364917294
ENSGALG00010008691	0.511264752	0.043217369
ENSGALG00010008693	0.977674611	0.333987298
ENSGALG00010008694	0.475295296	0.169104794
ENSGALG00010008695	0.929377835	0.291520101
ENSGALG00010008696	0.848816876	0.213526723
ENSGALG00010008697	0.859270688	0.302904463
ENSGALG00010008698	0.283109645	0.065474856
ENSGALG00010008699	0.933925066	0.246175055
ENSGALG00010008700	0.925262766	0.159928901
ENSGALG00010008701	0.48290496	0.151973614
ENSGALG00010008702	0.72360016	0.333820157
ENSGALG00010008705	0.621539725	0.239924439
ENSGALG00010008706	0.826775	0.293550008
ENSGALG00010008708	0.91054215	0.253494434
ENSGALG00010008709	0.561463235	0.160924768
ENSGALG00010008710	0.25106244	0.22916411
ENSGALG00010008711	0.370314901	0.115441211
ENSGALG00010008712	0.37996605	0.103859099
ENSGALG00010008713	0.742390918	0.125751375
ENSGALG00010008716	0.915963183	0.32410085
ENSGALG00010008717	0.925647354	0.375634124
ENSGALG00010008718	0.863064187	0.393811237
ENSGALG00010008719	0.954679197	0.375407776
ENSGALG00010008721	0.97469113	0.305001045
ENSGALG00010008724	0.444938797	0.224439585
ENSGALG00010008726	0.398350651	0.147430316
ENSGALG00010008728	0.861890732	0.330187184
ENSGALG00010008729	0.986677444	0.285882018

ENSGALG00010008731	0.629755756	0.243694227
ENSGALG00010008732	0.356901954	0.14624352
ENSGALG00010008733	0.826181311	0.170253991
ENSGALG00010008737	0.180620012	0.198088429
ENSGALG00010008739	0.567432275	0.011159964
ENSGALG00010008741	0.086625756	0.174264531
ENSGALG00010008742	0.648936393	0.352559223
ENSGALG00010008743	0.131865323	0.089022932
ENSGALG00010008744	0.137923671	0.201947156
ENSGALG00010008745	0.965980011	0.274767935
ENSGALG00010008746	0.232768793	0.23416579
ENSGALG00010008749	0.409530736	0.203856835
ENSGALG00010008750	0.881439388	0.208500697
ENSGALG00010008751	0.574437747	0.202564113
ENSGALG00010008752	0.191188815	0.06387782
ENSGALG00010008754	0.187514554	0.108479243
ENSGALG00010008756	0.628062093	0.372757773
ENSGALG00010008758	0.295081293	0.447565639
ENSGALG00010008760	0.034060073	0.094660884
ENSGALG00010008761	0.301937873	0.006398703
ENSGALG00010008762	0.851993058	0.280342545
ENSGALG00010008764	0.875961958	0.359323727
ENSGALG00010008765	0.7012158	0.324237811
ENSGALG00010008767	0.739309775	0.478409677
ENSGALG00010008768	0.933139099	0.260790142
ENSGALG00010008770	0.628630064	0.172298878
ENSGALG00010008774	0.841998934	0.233580936
ENSGALG00010008775	0.127561638	0.277153751
ENSGALG00010008777	0.456332809	0.096071558
ENSGALG00010008778	0.580821958	0.26072042
ENSGALG00010008780	0.729392557	0.200964658
ENSGALG00010008782	0.685013059	0.056816472
ENSGALG00010008786	0.172500532	0.269492389
ENSGALG00010008790	0.908638502	0.254790708
ENSGALG00010008793	0.958955969	0.277153458
ENSGALG00010008796	0.751707923	0.246238804
ENSGALG00010008799	0.26643896	0.078371307
ENSGALG00010008801	0.292709036	0.327963953
ENSGALG00010008805	0.1626469	0.335039682
ENSGALG00010008807	0.86382439	0.486616667
ENSGALG00010008810	0.109906753	0.22849301
ENSGALG00010008811	0.93610474	0.351726517
ENSGALG00010008813	0.561916299	0.19614748
ENSGALG00010008814	0.91298317	0.235279898
ENSGALG00010008815	0.422928843	0.025570081
ENSGALG00010008816	0.599016573	0.365352256
ENSGALG00010008818	0.194830451	0.191307586
ENSGALG00010008820	0.219386918	0.079388121
ENSGALG00010008821	0.923477573	0.444181007
ENSGALG00010008822	0.630058538	0.293644221
ENSGALG00010008824	0.404159456	0.225719224
ENSGALG00010008825	0.71144556	0.289335128
ENSGALG00010008826	0.223229419	0.032312808
ENSGALG00010008829	0.522311319	0.096645298
ENSGALG00010008830	0.943511347	0.240748726
ENSGALG00010008831	0.194670287	0.019616138

ENSGALG00010008832	0.9851585	0.326463054
ENSGALG00010008836	0.696463752	0.014960071
ENSGALG00010008837	0.169116601	0.299384436
ENSGALG00010008838	0.383549736	0.180028334
ENSGALG00010008839	0.84375514	0.339189814
ENSGALG00010008842	0.325918446	0.235232335
ENSGALG00010008844	0.434525696	0.274241029
ENSGALG00010008845	0.006860602	0.03125996
ENSGALG00010008846	0.506663035	0.225484089
ENSGALG00010008847	0.198048631	0.177023802
ENSGALG00010008848	0.066222657	0.269018252
ENSGALG00010008850	0.580156999	0.34445604
ENSGALG00010008852	0.985249571	0.343470861
ENSGALG00010008854	0.754866523	0.231803077
ENSGALG00010008857	0.027569167	0.176120446
ENSGALG00010008859	0.078697228	0.192091108
ENSGALG00010008860	0.946978013	0.177925207
ENSGALG00010008861	0.381741179	0.07825054
ENSGALG00010008862	0.065331766	0.130038622
ENSGALG00010008864	0.204752133	0.034519469
ENSGALG00010008866	0.871116515	0.420928837
ENSGALG00010008867	0.682031616	0.452980769
ENSGALG00010008869	0.050564	0.120729262
ENSGALG00010008871	0.51381439	0.327606089
ENSGALG00010008872	0.235025311	0.029910414
ENSGALG00010008876	0.402974961	0.000449976
ENSGALG00010008877	0.59240741	0.038200881
ENSGALG00010008879	0.596360374	0.160708667
ENSGALG00010008880	0.285035043	0.405216513
ENSGALG00010008884	0.067933951	0.099990697
ENSGALG00010008886	0.265558089	0.028821141
ENSGALG00010008888	0.963397939	0.327662715
ENSGALG00010008892	0.338070107	0.207358644
ENSGALG00010008893	0.985940753	0.345245814
ENSGALG00010008894	0.507817099	0.166841031
ENSGALG00010008895	0.645459438	0.302482662
ENSGALG00010008896	0.534902397	0.215247488
ENSGALG00010008897	0.345273886	0.267769067
ENSGALG00010008899	0.159301598	0.292122312
ENSGALG00010008901	0.389933649	0.180873577
ENSGALG00010008902	0.953624467	0.336894746
ENSGALG00010008903	0.878491622	0.390871132
ENSGALG00010008904	0.386699547	0.186313031
ENSGALG00010008905	0.451358035	0.324446957
ENSGALG00010008906	0.939244053	0.403939409
ENSGALG00010008907	0.531495858	0.200002318
ENSGALG00010008909	0.653392824	0.141407499
ENSGALG00010008910	0.25522022	0.145054902
ENSGALG00010008918	0.78086175	0.537880896
ENSGALG00010008919	0.013910379	0.356198279
ENSGALG00010008921	0.948990824	0.318020957
ENSGALG00010008923	0.37663102	0.133050909
ENSGALG00010008926	0.393904719	0.246885565
ENSGALG00010008927	0.132042346	0.158277128
ENSGALG00010008928	0.499761322	0.155475493
ENSGALG00010008929	0.095432038	0.329020021

ENSGALG00010008931	0.939079412	0.412605286
ENSGALG00010008932	0.654411847	0.108278647
ENSGALG00010008934	0.265558089	0.028821141
ENSGALG00010008935	0.936261615	0.302728636
ENSGALG00010008936	0.43100393	0.04868432
ENSGALG00010008938	0.850964424	0.32979689
ENSGALG00010008939	0.966009453	0.245149281
ENSGALG00010008941	0.156062592	0.022622794
ENSGALG00010008942	0.697735063	0.28254601
ENSGALG00010008943	0.241181267	0.029233134
ENSGALG00010008944	0.216433634	0.680598293
ENSGALG00010008945	0.821684952	0.237522252
ENSGALG00010008946	0.476819387	0.095007482
ENSGALG00010008947	0.859537531	0.15540554
ENSGALG00010008952	0.056612317	0.337435458
ENSGALG00010008953	0.187514554	0.108479243
ENSGALG00010008955	0.961449505	0.251829358
ENSGALG00010008956	0.517378115	0.261071918
ENSGALG00010008960	0.992614623	0.320272381
ENSGALG00010008961	0.38718504	0.101838932
ENSGALG00010008964	0.195466331	0.209451297
ENSGALG00010008966	0.951084565	0.384360489
ENSGALG00010008967	0.271701261	0.072417625
ENSGALG00010008968	0.301227792	0.223805313
ENSGALG00010008969	0.890130545	0.328671239
ENSGALG00010008970	0.324228994	0.202759031
ENSGALG00010008971	0.955718125	0.350754127
ENSGALG00010008974	0.64747707	0.20221845
ENSGALG00010008979	0.277566769	0.107857127
ENSGALG00010008980	0.394494248	0.128304162
ENSGALG00010008983	0.477512283	0.45573071
ENSGALG00010008986	0.394327047	0.133628127
ENSGALG00010008988	0.928100116	0.325572553
ENSGALG00010008989	0.722362137	0.234846114
ENSGALG00010008991	0.963631514	0.300685077
ENSGALG00010008992	0.573828609	0.185476752
ENSGALG00010008994	0.367968282	0.418756987
ENSGALG00010008995	0.861135341	0.505714848
ENSGALG00010008996	0.816926461	0.415184754
ENSGALG00010008997	0.914672671	0.275723792
ENSGALG00010008999	0.955987808	0.357665693
ENSGALG00010009000	0.944748236	0.310027513
ENSGALG00010009002	0.992845076	0.370049387
ENSGALG00010009003	0.971093984	0.271071339
ENSGALG00010009007	0.842697283	0.442781531
ENSGALG00010009010	0.917282102	0.164716352
ENSGALG00010009011	0.921442449	0.303483954
ENSGALG00010009012	0.003409832	0.095139971
ENSGALG00010009013	0.156069065	0.101634428
ENSGALG00010009014	0.972721473	0.333187145
ENSGALG00010009015	0.955935637	0.367985862
ENSGALG00010009016	0.455729238	0.18353218
ENSGALG00010009018	0.526600511	0.214217539
ENSGALG00010009019	0.975854364	0.338020116
ENSGALG00010009020	0.915308388	0.252963571
ENSGALG00010009021	0.987653965	0.341757251

ENSGALG00010009022	0.386114539	0.074004299
ENSGALG00010009023	0.975146153	0.332876233
ENSGALG00010009024	0.939789413	0.322334208
ENSGALG00010009025	0.79614117	0.255274074
ENSGALG00010009026	0.932704201	0.318961842
ENSGALG00010009027	0.411944811	0.37913248
ENSGALG00010009028	0.925546383	0.308752592
ENSGALG00010009029	0.915081728	0.450558875
ENSGALG00010009030	0.95759564	0.227553543
ENSGALG00010009031	0.064235564	0.117198385
ENSGALG00010009034	0.975038043	0.306443296
ENSGALG00010009035	0.906879828	0.212988447
ENSGALG00010009036	0.917046085	0.422100016
ENSGALG00010009037	0.910905121	0.224987129
ENSGALG00010009038	0.889884659	0.350941074
ENSGALG00010009039	0.664558848	0.034026512
ENSGALG00010009040	0.943666876	0.279157443
ENSGALG00010009043	0.501538117	0.354228538
ENSGALG00010009045	0.529449658	0.252168504
ENSGALG00010009047	0.974297623	0.250239488
ENSGALG00010009049	0.68555979	0.266527462
ENSGALG00010009050	0.89886885	0.401999661
ENSGALG00010009051	0.531310299	0.082984895
ENSGALG00010009052	0.4703555	0.141400331
ENSGALG00010009053	0.266349267	0.252922358
ENSGALG00010009054	0.197837976	0.043386327
ENSGALG00010009056	0.111723115	0.155918956
ENSGALG00010009057	0.681859389	0.241438104
ENSGALG00010009059	0.898054066	0.373529387
ENSGALG00010009062	0.060762054	0.1387881
ENSGALG00010009063	0.844937446	0.027751253
ENSGALG00010009064	0.467670801	0.421953779
ENSGALG00010009065	0.976775537	0.293488498
ENSGALG00010009067	0.220662747	0.12282644
ENSGALG00010009068	0.969911747	0.356837062
ENSGALG00010009069	0.442331328	0.132428836
ENSGALG00010009073	0.862594433	0.069035557
ENSGALG00010009076	0.55523752	0.160971579
ENSGALG00010009078	0.532141184	0.242674197
ENSGALG00010009079	0.843061852	0.216187101
ENSGALG00010009080	0.835472331	0.371949262
ENSGALG00010009081	0.914756464	0.417714099
ENSGALG00010009083	0.963670507	0.314161714
ENSGALG00010009085	0.905506302	0.345526877
ENSGALG00010009086	0.979644086	0.284620049
ENSGALG00010009088	0.257551669	0.202839218
ENSGALG00010009089	0.623004254	0.279830405
ENSGALG00010009091	0.99037278	0.340714766
ENSGALG00010009092	0.693808657	0.302135973
ENSGALG00010009093	0.453879638	0.565438343
ENSGALG00010009094	0.983221	0.290175282
ENSGALG00010009096	0.782429726	0.01831362
ENSGALG00010009097	0.940072769	0.360019852
ENSGALG00010009098	0.083152552	0.067899087
ENSGALG00010009099	0.109021482	0.004454984
ENSGALG00010009100	0.894171846	0.545348722

ENSGALG00010009101	0.964876725	0.272956989
ENSGALG00010009102	0.84008617	0.431321768
ENSGALG00010009103	0.978858846	0.296462955
ENSGALG00010009104	0.9518256	0.340949326
ENSGALG00010009106	0.823792062	0.306600154
ENSGALG00010009109	0.331330803	0.249368958
ENSGALG00010009111	0.96456896	0.269097365
ENSGALG00010009112	0.992428646	0.35992109
ENSGALG00010009113	0.93165262	0.363513446
ENSGALG00010009114	0.970280423	0.311636373
ENSGALG00010009115	0.808630181	0.444899957
ENSGALG00010009116	0.939810328	0.402997441
ENSGALG00010009117	0.011388611	0.033513389
ENSGALG00010009118	0.985287302	0.318558799
ENSGALG00010009119	0.622373385	0.171707503
ENSGALG00010009120	0.946575071	0.241606145
ENSGALG00010009121	0.876425751	0.194805129
ENSGALG00010009123	0.950115389	0.322144986
ENSGALG00010009125	0.824996639	0.30121298
ENSGALG00010009126	0.981872172	0.338311151
ENSGALG00010009127	0.954136773	0.278580471
ENSGALG00010009129	0.984653819	0.378832714
ENSGALG00010009130	0.925466613	0.292337979
ENSGALG00010009131	0.861045423	0.160236298
ENSGALG00010009132	0.121011013	0.208763575
ENSGALG00010009133	0.820244759	0.145828
ENSGALG00010009135	0.68244371	0.367118157
ENSGALG00010009136	0.990254014	0.265510666
ENSGALG00010009137	0.961150753	0.354075354
ENSGALG00010009138	0.074622415	0.36021485
ENSGALG00010009139	0.249199688	0.207482223
ENSGALG00010009140	0.111131027	0.058732907
ENSGALG00010009141	0.951053537	0.315687889
ENSGALG00010009142	0.990663262	0.315580469
ENSGALG00010009143	0.500877653	0.023234526
ENSGALG00010009144	0.719174818	0.097008602
ENSGALG00010009145	0.386114539	0.074004299
ENSGALG00010009146	0.322633957	0.000124062
ENSGALG00010009147	0.984447271	0.353935568
ENSGALG00010009148	0.96943116	0.339120748
ENSGALG00010009149	0.877478808	0.107646593
ENSGALG00010009150	0.742864746	0.152871774
ENSGALG00010009151	0.180340613	0.076336052
ENSGALG00010009152	0.913105026	0.255890084
ENSGALG00010009153	0.876139769	0.292138235
ENSGALG00010009155	0.927164216	0.281907439
ENSGALG00010009157	0.462730488	0.128835678
ENSGALG00010009158	0.320729011	0.144028995
ENSGALG00010009159	0.270148847	0.180937283
ENSGALG00010009160	0.399453135	0.144666429
ENSGALG00010009162	0.290122505	0.464993379
ENSGALG00010009163	0.243454145	0.103758247
ENSGALG00010009164	0.347257554	0.217050453
ENSGALG00010009165	0.2935711	0.261798438
ENSGALG00010009166	0.878966288	0.2828647
ENSGALG00010009167	0.977424729	0.405188534

ENSGALG00010009168	0.932627227	0.35306971
ENSGALG00010009170	0.874613092	0.287828316
ENSGALG00010009171	0.064856166	0.171303517
ENSGALG00010009172	0.352715211	0.300980491
ENSGALG00010009173	0.584255144	0.124661154
ENSGALG00010009176	0.464198464	0.340291937
ENSGALG00010009180	0.963715185	0.312456496
ENSGALG00010009181	0.967969233	0.319114656
ENSGALG00010009183	0.469255613	0.111606247
ENSGALG00010009185	0.621674414	0.239352559
ENSGALG00010009186	0.431191163	0.106214945
ENSGALG00010009187	0.750453997	0.33968897
ENSGALG00010009188	0.622715334	0.291995876
ENSGALG00010009189	0.147903574	0.263058826
ENSGALG00010009190	0.821679594	0.447164632
ENSGALG00010009191	0.940808098	0.304632891
ENSGALG00010009192	0.816177408	0.265160119
ENSGALG00010009193	0.924379172	0.221043504
ENSGALG00010009194	0.883204148	0.425437341
ENSGALG00010009195	0.461499486	0.127107551
ENSGALG00010009196	0.390466756	0.106163828
ENSGALG00010009197	0.511829601	0.308466888
ENSGALG00010009198	0.67506301	0.380338521
ENSGALG00010009199	0.979439905	0.289819726
ENSGALG00010009200	0.809383461	0.204091307
ENSGALG00010009202	0.908526098	0.250210769
ENSGALG00010009204	0.551107417	0.277663202
ENSGALG00010009205	0.955908767	0.30026816
ENSGALG00010009206	0.265558089	0.028821141
ENSGALG00010009208	0.942578908	0.278909918
ENSGALG00010009210	0.283107207	0.256622444
ENSGALG00010009212	0.690742056	0.314145115
ENSGALG00010009213	0.924664807	0.375621987
ENSGALG00010009214	0.204270534	0.015385154
ENSGALG00010009216	0.949363234	0.287223072
ENSGALG00010009219	0.988102595	0.277677712
ENSGALG00010009220	0.552451851	0.247339038
ENSGALG00010009222	0.081102735	0.195874932
ENSGALG00010009223	0.984439957	0.282083306
ENSGALG00010009224	0.993449492	0.332112893
ENSGALG00010009225	0.88394196	0.135324968
ENSGALG00010009227	0.940768395	0.297679463
ENSGALG00010009228	0.836791816	0.435336129
ENSGALG00010009231	0.633043105	0.359376128
ENSGALG00010009232	0.928772901	0.323568315
ENSGALG00010009233	0.288602645	0.101844431
ENSGALG00010009234	0.481079393	0.212237748
ENSGALG00010009235	0.969345385	0.356978111
ENSGALG00010009236	0.233238615	0.176685012
ENSGALG00010009237	0.625610224	0.091709738
ENSGALG00010009238	0.502802413	0.125331407
ENSGALG00010009239	0.933668652	0.194712459
ENSGALG00010009240	0.918552142	0.295346134
ENSGALG00010009241	0.723499267	0.248073173
ENSGALG00010009243	0.50442438	0.243842813
ENSGALG00010009245	0.315224891	0.366601178

ENSGALG00010009246	0.967909224	0.304550194
ENSGALG00010009250	0.987342577	0.264556317
ENSGALG00010009252	0.59091888	0.147702636
ENSGALG00010009253	0.941050253	0.323081185
ENSGALG00010009254	0.599971609	0.26845767
ENSGALG00010009256	0.930477187	0.392007724
ENSGALG00010009257	0.973905107	0.335201618
ENSGALG00010009258	0.890605537	0.336197079
ENSGALG00010009259	0.650931426	0.276111283
ENSGALG00010009262	0.285272007	0.109169955
ENSGALG00010009263	0.984681791	0.310434326
ENSGALG00010009265	0.366667574	0.096374266
ENSGALG00010009266	0.786586086	0.328716985
ENSGALG00010009268	0.116008223	0.00077567
ENSGALG00010009270	0.391005801	0.038905436
ENSGALG00010009273	0.936752182	0.191796319
ENSGALG00010009275	0.652931963	0.201046922
ENSGALG00010009276	0.738791106	0.066803798
ENSGALG00010009277	0.883438233	0.205720054
ENSGALG00010009279	0.952877733	0.293984288
ENSGALG00010009280	0.969056423	0.326423002
ENSGALG00010009282	0.294127039	0.406617819
ENSGALG00010009283	0.000246074	0.091301674
ENSGALG00010009284	0.475833564	0.301277194
ENSGALG00010009285	0.589294659	0.040472659
ENSGALG00010009286	0.756606091	0.094409416
ENSGALG00010009287	0.034835075	0.173750304
ENSGALG00010009289	0.938875239	0.278413877
ENSGALG00010009291	0.266137114	0.107784983
ENSGALG00010009294	0.926007283	0.189326373
ENSGALG00010009297	0.976409118	0.33700196
ENSGALG00010009299	0.965345724	0.276909738
ENSGALG00010009303	0.960740608	0.272158944
ENSGALG00010009306	0.501565408	0.19157095
ENSGALG00010009307	0.838636063	0.174808097
ENSGALG00010009309	0.988426001	0.333080229
ENSGALG00010009311	0.457913492	0.463751825
ENSGALG00010009313	0.95479691	0.368548525
ENSGALG00010009315	0.498423932	0.168195647
ENSGALG00010009317	0.335344854	0.151757791
ENSGALG00010009320	0.348840432	0.204270277
ENSGALG00010009322	0.603037122	0.148478032
ENSGALG00010009323	0.974764427	0.312652535
ENSGALG00010009326	0.502206088	0.049577608
ENSGALG00010009327	0.366499402	0.304655994
ENSGALG00010009328	0.931508992	0.526057802
ENSGALG00010009329	0.370493859	0.13155368
ENSGALG00010009330	0.905691252	0.342406946
ENSGALG00010009332	0.512287777	0.323108314
ENSGALG00010009333	0.981021055	0.297915877
ENSGALG00010009334	0.975587451	0.32325165
ENSGALG00010009335	0.978498108	0.312735504
ENSGALG00010009336	0.948826582	0.448275905
ENSGALG00010009337	0.964602535	0.314636072
ENSGALG00010009338	0.980447156	0.394301107
ENSGALG00010009340	0.946679435	0.339048444

ENSGALG00010009343	0.058496737	0.160404795
ENSGALG00010009344	0.97286695	0.305544833
ENSGALG00010009345	0.192629209	0.268587133
ENSGALG00010009346	0.043134879	0.186784523
ENSGALG00010009347	0.988097936	0.326529337
ENSGALG00010009348	0.335055705	0.169278136
ENSGALG00010009349	0.954532933	0.346031051
ENSGALG00010009351	0.964948396	0.361960929
ENSGALG00010009352	0.947587485	0.244733778
ENSGALG00010009353	0.496202257	0.280253662
ENSGALG00010009354	0.226822397	0.05108955
ENSGALG00010009355	0.968583472	0.340225635
ENSGALG00010009357	0.961271066	0.226803844
ENSGALG00010009358	0.579641124	0.008298689
ENSGALG00010009359	0.977778112	0.344144641
ENSGALG00010009360	0.957480469	0.276748816
ENSGALG00010009361	0.01309043	0.509354819
ENSGALG00010009362	0.646613546	0.36113991
ENSGALG00010009363	0.347822576	0.222215904
ENSGALG00010009364	0.955650336	0.312549598
ENSGALG00010009365	0.981443672	0.291578103
ENSGALG00010009366	0.011589493	0.110944875
ENSGALG00010009367	0.833896727	0.313310841
ENSGALG00010009368	0.064024696	0.301669988
ENSGALG00010009369	0.052350997	0.305310891
ENSGALG00010009370	0.923780307	0.132538641
ENSGALG00010009371	0.961629554	0.269501968
ENSGALG00010009372	0.429966208	0.113367164
ENSGALG00010009373	0.95355176	0.212217504
ENSGALG00010009374	0.965040998	0.263874513
ENSGALG00010009375	0.236751829	0.036511106
ENSGALG00010009376	0.98467956	0.271440355
ENSGALG00010009377	0.203991683	0.058221925
ENSGALG00010009378	0.917278977	0.376395611
ENSGALG00010009379	0.997348216	0.329345462
ENSGALG00010009381	0.747523189	0.068480703
ENSGALG00010009382	0.348645811	0.106405353
ENSGALG00010009383	0.929993827	0.328891435
ENSGALG00010009384	0.833502142	0.096104326
ENSGALG00010009385	0.997556421	0.334252269
ENSGALG00010009386	0.14300637	0.036015413
ENSGALG00010009387	0.986823971	0.323408404
ENSGALG00010009388	0.978023265	0.314943503
ENSGALG00010009389	0.410791808	0.080663222
ENSGALG00010009390	0.732263091	0.224098365
ENSGALG00010009391	0.828192776	0.239506573
ENSGALG00010009392	0.98987624	0.292974104
ENSGALG00010009393	0.975288485	0.289932762
ENSGALG00010009394	0.221823731	0.062618599
ENSGALG00010009395	0.151025479	0.370594939
ENSGALG00010009396	0.868618933	0.373956457
ENSGALG00010009397	0.555229777	0.416065782
ENSGALG00010009398	0.76300181	0.057648165
ENSGALG00010009399	0.223262192	0.216458078
ENSGALG00010009400	0.79757726	0.269385928
ENSGALG00010009401	0.143484564	0.153820707

ENSGALG00010009403	0.294661357	0.109904448
ENSGALG00010009404	0.721888753	0.255998586
ENSGALG00010009407	0.955787645	0.240139833
ENSGALG00010009408	0.889652662	0.327018886
ENSGALG00010009409	0.34081212	0.34730063
ENSGALG00010009410	0.916874008	0.369021529
ENSGALG00010009411	0.944993721	0.365207044
ENSGALG00010009412	0.061759815	0.125212148
ENSGALG00010009414	0.471461019	0.20171668
ENSGALG00010009415	0.43991956	0.219830184
ENSGALG00010009416	0.851820072	0.021260842
ENSGALG00010009420	0.414379968	0.375196934
ENSGALG00010009425	0.45479502	0.427734319
ENSGALG00010009430	0.817821068	0.284921971
ENSGALG00010009431	0.975147618	0.333105203
ENSGALG00010009432	0.9025601	0.36213337
ENSGALG00010009433	0.502070782	0.231271286
ENSGALG00010009435	0.016162984	0.384203003
ENSGALG00010009437	0.901758299	0.40237393
ENSGALG00010009439	0.926368918	0.325591777
ENSGALG00010009440	0.027075211	0.143607493
ENSGALG00010009441	0.915012447	0.460468752
ENSGALG00010009444	0.560759228	0.375273652
ENSGALG00010009446	0.977621395	0.363006112
ENSGALG00010009447	0.428779355	0.024051302
ENSGALG00010009451	0.961492304	0.401870463
ENSGALG00010009452	0.94530732	0.285301616
ENSGALG00010009453	0.83970025	0.316616496
ENSGALG00010009457	0.588873966	0.109876895
ENSGALG00010009458	0.524817041	0.078034272
ENSGALG00010009461	0.215549513	0.01339
ENSGALG00010009462	0.977767264	0.29883253
ENSGALG00010009463	0.909229836	0.280382003
ENSGALG00010009464	0.960639337	0.406882263
ENSGALG00010009465	0.315049808	0.216957599
ENSGALG00010009466	0.703089043	0.386298991
ENSGALG00010009467	0.851793542	0.378202687
ENSGALG00010009468	0.770064431	0.290023954
ENSGALG00010009469	0.985671194	0.321358723
ENSGALG00010009470	0.274542993	0.332601871
ENSGALG00010009471	0.748800997	0.683286473
ENSGALG00010009472	0.96779857	0.282503036
ENSGALG00010009474	0.601424526	0.078414603
ENSGALG00010009477	0.366027373	0.203034917
ENSGALG00010009479	0.842525175	0.231298957
ENSGALG00010009482	0.709250917	0.254844038
ENSGALG00010009484	0.401925722	0.281494051
ENSGALG00010009486	0.98770343	0.339835369
ENSGALG00010009489	0.819325845	0.399093297
ENSGALG00010009490	0.976752552	0.210410214
ENSGALG00010009491	0.80225921	0.216904046
ENSGALG00010009492	0.939018123	0.219497618
ENSGALG00010009493	0.890494181	0.205397652
ENSGALG00010009494	0.643673225	0.416982099
ENSGALG00010009497	0.516495709	0.011547102
ENSGALG00010009498	0.789230541	0.05527468

ENSGALG00010009499	0.036506864	0.150507451
ENSGALG00010009500	0.42431614	0.006155289
ENSGALG00010009501	0.908855063	0.266715463
ENSGALG00010009502	0.961824761	0.311446225
ENSGALG00010009503	0.926150899	0.197492332
ENSGALG00010009506	0.337143935	0.420377906
ENSGALG00010009507	0.156611519	0.264062351
ENSGALG00010009508	0.855954248	0.38256078
ENSGALG00010009509	0.097291621	0.364606668
ENSGALG00010009511	0.97357764	0.365332471
ENSGALG00010009514	0.942429676	0.193145684
ENSGALG00010009515	0.864021965	0.188713304
ENSGALG00010009516	0.095653271	0.072578384
ENSGALG00010009517	0.824988897	0.483362515
ENSGALG00010009518	0.92466331	0.210118272
ENSGALG00010009520	0.975276483	0.269666801
ENSGALG00010009522	0.942677174	0.311692928
ENSGALG00010009524	0.920847991	0.300687004
ENSGALG00010009525	0.968142895	0.191319949
ENSGALG00010009526	0.608837082	0.055839094
ENSGALG00010009527	0.977107223	0.202925595
ENSGALG00010009530	0.329860191	0.061550364
ENSGALG00010009531	0.664476018	0.387177837
ENSGALG00010009533	0.265558089	0.028821141
ENSGALG00010009534	0.813313413	0.224089747
ENSGALG00010009535	0.462035818	0.100622993
ENSGALG00010009537	0.386086572	0.495102699
ENSGALG00010009538	0.839824718	0.222069209
ENSGALG00010009540	0.992620029	0.299946617
ENSGALG00010009541	0.097927457	0.3097471
ENSGALG00010009542	0.588178733	0.102538619
ENSGALG00010009543	0.992748982	0.327172093
ENSGALG00010009546	0.250534534	0.233169304
ENSGALG00010009548	0.864038151	0.259937966
ENSGALG00010009551	0.807607032	0.27527286
ENSGALG00010009554	0.281141827	0.142705631
ENSGALG00010009555	0.927352837	0.416091197
ENSGALG00010009557	0.969136466	0.303369289
ENSGALG00010009558	0.73121741	0.303941832
ENSGALG00010009559	0.006836752	0.210729843
ENSGALG00010009560	0.486128815	0.38436723
ENSGALG00010009562	0.921107239	0.284151895
ENSGALG00010009565	0.89368959	0.476678314
ENSGALG00010009566	0.282151895	0.179552023
ENSGALG00010009567	0.317168503	0.672001423
ENSGALG00010009569	0.855551684	0.153648181
ENSGALG00010009570	0.979950277	0.285793666
ENSGALG00010009571	0.985483577	0.365352761
ENSGALG00010009573	0.956343808	0.297851799
ENSGALG00010009574	0.490756073	0.15354372
ENSGALG00010009575	0.975128184	0.316163878
ENSGALG00010009576	0.940140454	0.320303575
ENSGALG00010009577	0.011589493	0.110944875
ENSGALG00010009578	0.451890746	0.298515228
ENSGALG00010009579	0.641792502	0.099534966
ENSGALG00010009580	0.440508737	0.322120722

ENSGALG00010009581	0.662036174	0.260784922
ENSGALG00010009582	0.440263521	0.028350116
ENSGALG00010009583	0.155752593	0.296074878
ENSGALG00010009587	0.912330454	0.152983271
ENSGALG00010009588	0.606085042	0.09331025
ENSGALG00010009589	0.947527893	0.413817134
ENSGALG00010009591	0.160205246	0.528740221
ENSGALG00010009592	0.558135531	0.064790871
ENSGALG00010009593	0.737900796	0.312022488
ENSGALG00010009594	0.22385336	0.228458282
ENSGALG00010009595	0.989018571	0.316786148
ENSGALG00010009596	0.745006126	0.265874097
ENSGALG00010009597	0.683617121	0.188583908
ENSGALG00010009599	0.203991683	0.058221925
ENSGALG00010009600	0.874935606	0.40374331
ENSGALG00010009601	0.597897514	0.343050332
ENSGALG00010009602	0.856120821	0.402773784
ENSGALG00010009603	0.987659787	0.353262211
ENSGALG00010009604	0.377008934	0.178767925
ENSGALG00010009605	0.863653139	0.526477573
ENSGALG00010009606	0.307575601	0.433875535
ENSGALG00010009608	0.409904383	0.224854342
ENSGALG00010009609	0.962140108	0.329167312
ENSGALG00010009612	0.972034569	0.313651745
ENSGALG00010009615	0.867696112	0.440618142
ENSGALG00010009616	0.358653964	0.083018769
ENSGALG00010009617	0.449411521	0.254524285
ENSGALG00010009618	0.095103607	0.010456941
ENSGALG00010009619	0.986690161	0.33598594
ENSGALG00010009620	0.970933976	0.358962906
ENSGALG00010009621	0.49020544	0.213524861
ENSGALG00010009623	0.388812529	0.236752812
ENSGALG00010009624	0.277566769	0.107857127
ENSGALG00010009625	0.571176978	0.16837699
ENSGALG00010009627	0.233163402	0.119740103
ENSGALG00010009629	0.92676478	0.342054006
ENSGALG00010009631	0.264239718	0.073427965
ENSGALG00010009634	0.863737372	0.316858944
ENSGALG00010009635	0.964609497	0.278599368
ENSGALG00010009637	0.640446143	0.018760199
ENSGALG00010009638	0.497406396	0.242271483
ENSGALG00010009640	0.95190811	0.31257053
ENSGALG00010009643	0.986641309	0.33829349
ENSGALG00010009644	0.482632386	0.22150328
ENSGALG00010009645	0.468179601	0.20442508
ENSGALG00010009646	0.225011329	0.066660175
ENSGALG00010009647	0.655064719	0.081205866
ENSGALG00010009649	0.934405624	0.254374783
ENSGALG00010009650	0.987947045	0.352239836
ENSGALG00010009652	0.563438833	0.194682084
ENSGALG00010009653	0.978629194	0.335553124
ENSGALG00010009654	0.9734121	0.357437583
ENSGALG00010009655	0.102173558	0.134877586
ENSGALG00010009657	0.675228119	0.184303943
ENSGALG00010009658	0.765858214	0.060355387
ENSGALG00010009659	0.629241105	0.004876131

ENSGALG00010009663	0.977809962	0.32807451
ENSGALG00010009665	0.963638586	0.525499182
ENSGALG00010009666	0.810271303	0.293482743
ENSGALG00010009669	0.048954231	0.160736934
ENSGALG00010009671	0.335553126	0.154693383
ENSGALG00010009672	0.846377223	0.45918451
ENSGALG00010009673	0.230116839	0.265243756
ENSGALG00010009676	0.996300368	0.325584879
ENSGALG00010009677	0.958075593	0.34300424
ENSGALG00010009678	0.92072931	0.35154227
ENSGALG00010009679	0.925877045	0.284256071
ENSGALG00010009683	0.866395414	0.377464342
ENSGALG00010009685	0.398190249	0.125967147
ENSGALG00010009686	0.964114384	0.314866797
ENSGALG00010009689	0.977111406	0.141948623
ENSGALG00010009690	0.905292703	0.380295865
ENSGALG00010009691	0.274489239	0.148680386
ENSGALG00010009695	0.98039601	0.323624969
ENSGALG00010009697	0.092968	0.000815592
ENSGALG00010009699	0.476244897	0.234332884
ENSGALG00010009700	0.964304484	0.19358275
ENSGALG00010009702	0.711456327	0.419900663
ENSGALG00010009703	0.677048397	0.003025202
ENSGALG00010009704	0.773393201	0.030485318
ENSGALG00010009705	0.765020317	0.114577576
ENSGALG00010009707	0.285272007	0.109169955
ENSGALG00010009708	0.278960356	0.202096482
ENSGALG00010009710	0.479996964	0.324408793
ENSGALG00010009712	0.99130254	0.335895774
ENSGALG00010009715	0.030615595	0.191807048
ENSGALG00010009717	0.885259381	0.433963748
ENSGALG00010009718	0.364509854	0.168471296
ENSGALG00010009719	0.842573772	0.271554948
ENSGALG00010009720	0.979350338	0.329682958
ENSGALG00010009721	0.9721776	0.355822864
ENSGALG00010009722	0.605612245	0.182607879
ENSGALG00010009724	0.584200353	0.255218888
ENSGALG00010009725	0.126019257	0.032418075
ENSGALG00010009726	0.511220422	0.048854876
ENSGALG00010009727	0.94246361	0.407593405
ENSGALG00010009728	0.588457653	0.184545201
ENSGALG00010009730	0.356087325	0.159041782
ENSGALG00010009731	0.905962154	0.393063213
ENSGALG00010009732	0.729876145	0.26714909
ENSGALG00010009736	0.265668698	0.09683288
ENSGALG00010009738	0.484299427	0.282459952
ENSGALG00010009740	0.25229655	0.15721546
ENSGALG00010009741	0.880183615	0.295214871
ENSGALG00010009743	0.9355053	0.260672818
ENSGALG00010009744	0.759903103	0.323836285
ENSGALG00010009746	0.384966644	0.120247113
ENSGALG00010009749	0.950222904	0.239915819
ENSGALG00010009750	0.3638061	0.160273458
ENSGALG00010009751	0.162317265	0.409793822
ENSGALG00010009752	0.885394118	0.269665063
ENSGALG00010009753	0.984377799	0.326377063

ENSGALG00010009754	0.759962375	0.044752084
ENSGALG00010009755	0.499884119	0.186219421
ENSGALG00010009756	0.366824439	0.481171868
ENSGALG00010009759	0.484177998	0.010642839
ENSGALG00010009760	0.511774065	0.058201506
ENSGALG00010009761	0.908598863	0.2747089
ENSGALG00010009762	0.978073768	0.336441397
ENSGALG00010009763	0.658780791	0.262348004
ENSGALG00010009765	0.903404977	0.412018859
ENSGALG00010009766	0.373762061	0.177956487
ENSGALG00010009769	0.826625408	0.290337067
ENSGALG00010009772	0.292583563	0.339182213
ENSGALG00010009773	0.189240572	0.04996112
ENSGALG00010009774	0.918760457	0.156238238
ENSGALG00010009775	0.146131158	0.328767982
ENSGALG00010009776	0.815334565	0.389566982
ENSGALG00010009777	0.970338357	0.379993849
ENSGALG00010009778	0.840668347	0.313979659
ENSGALG00010009779	0.778148658	0.069008811
ENSGALG00010009780	0.656676942	0.362741865
ENSGALG00010009783	0.442020646	0.772771322
ENSGALG00010009784	0.450885553	0.213573826
ENSGALG00010009785	0.685256275	0.143604398
ENSGALG00010009786	0.63472222	0.371159432
ENSGALG00010009787	0.560931708	0.223293307
ENSGALG00010009788	0.975685356	0.338499425
ENSGALG00010009789	0.391468422	0.257347124
ENSGALG00010009791	0.531708629	0.243822988
ENSGALG00010009793	0.889482788	0.418936054
ENSGALG00010009795	0.943307525	0.411305036
ENSGALG00010009796	0.548920747	0.157002707
ENSGALG00010009797	0.030911843	0.059998647
ENSGALG00010009798	0.599124168	0.055964695
ENSGALG00010009801	0.067462641	0.227437614
ENSGALG00010009802	0.378999746	0.377668043
ENSGALG00010009803	0.035158038	0.119871525
ENSGALG00010009804	0.818989881	0.23851854
ENSGALG00010009805	0.743518269	0.245184294
ENSGALG00010009806	0.895372594	0.413421495
ENSGALG00010009807	0.471274576	0.056405625
ENSGALG00010009809	0.974047175	0.349545621
ENSGALG00010009810	0.372077039	0.061157367
ENSGALG00010009811	0.955400806	0.305035436
ENSGALG00010009813	0.015376535	0.08458412
ENSGALG00010009815	0.914377006	0.404967173
ENSGALG00010009819	0.490939355	0.689956703
ENSGALG00010009822	0.47747502	0.249114104
ENSGALG00010009829	0.303449328	0.076304882
ENSGALG00010009830	0.963570738	0.342154873
ENSGALG00010009831	0.868644744	0.337454957
ENSGALG00010009833	0.767357131	0.323276072
ENSGALG00010009834	0.789301261	0.348318938
ENSGALG00010009839	0.91474749	0.300888923
ENSGALG00010009840	0.093471257	0.169939488
ENSGALG00010009841	0.682902721	0.296582717
ENSGALG00010009842	0.973347387	0.380359263

ENSGALG00010009843	0.012433676	0.134633327
ENSGALG00010009844	0.070813117	0.240543186
ENSGALG00010009847	0.057003729	0.153215582
ENSGALG00010009848	0.109540589	0.06117983
ENSGALG00010009849	0.103735514	0.176217522
ENSGALG00010009850	0.873013659	0.332650298
ENSGALG00010009851	0.987967066	0.328866037
ENSGALG00010009852	0.708384984	0.55834405
ENSGALG00010009854	0.99376886	0.345609755
ENSGALG00010009857	0.649929258	0.486772894
ENSGALG00010009858	0.986252222	0.268260451
ENSGALG00010009864	0.188612019	0.394446161
ENSGALG00010009865	0.324020854	0.340314887
ENSGALG00010009866	0.973633792	0.302155469
ENSGALG00010009868	0.936799344	0.402958992
ENSGALG00010009869	0.421101213	0.283636776
ENSGALG00010009870	0.873424389	0.238916902
ENSGALG00010009871	0.104705059	0.204789516
ENSGALG00010009872	0.336405255	0.013337989
ENSGALG00010009873	0.9100757	0.418241258
ENSGALG00010009874	0.937106355	0.37514417
ENSGALG00010009876	0.985334786	0.317768199
ENSGALG00010009877	0.785956934	0.038099166
ENSGALG00010009878	0.491333139	0.214742062
ENSGALG00010009880	0.044640162	0.167754086
ENSGALG00010009881	0.64403313	0.243753009
ENSGALG00010009882	0.265558089	0.028821141
ENSGALG00010009883	0.061092695	0.039742892
ENSGALG00010009884	0.031686609	0.300007471
ENSGALG00010009888	0.215798964	0.284793648
ENSGALG00010009902	0.950911785	0.260523702
ENSGALG00010009905	0.49695585	0.240488329
ENSGALG00010009910	0.445601919	0.178660484
ENSGALG00010009914	0.692013264	0.406326878
ENSGALG00010009915	0.515059117	0.519145618
ENSGALG00010009922	0.4659562	0.134605374
ENSGALG00010009931	0.270451009	0.090541667
ENSGALG00010009932	0.305117634	0.208649402
ENSGALG00010009938	0.730696177	0.267885515
ENSGALG00010009940	0.390477114	0.143486823
ENSGALG00010009941	0.81897072	0.420076106
ENSGALG00010009943	0.913788886	0.35670485
ENSGALG00010009948	0.969738013	0.381179466
ENSGALG00010009954	0.111639917	0.19645113
ENSGALG00010009957	0.264239718	0.073427965
ENSGALG00010009959	0.399453749	0.144668892
ENSGALG00010009961	0.345957381	0.377989906
ENSGALG00010009962	0.531051992	0.08858496
ENSGALG00010009967	0.083085855	0.05492063
ENSGALG00010009970	0.422132218	0.074365263
ENSGALG00010009971	0.927306009	0.141964572
ENSGALG00010009972	0.974794931	0.293816426
ENSGALG00010009975	0.475532602	0.130065709
ENSGALG00010009978	0.747846086	0.245055183
ENSGALG00010009979	0.338467499	0.109387283
ENSGALG00010009982	0.467260259	0.10848689

ENSGALG00010009985	0.967267476	0.292697704
ENSGALG00010009989	0.395366848	0.202193964
ENSGALG00010009990	0.029558715	0.175619474
ENSGALG00010009991	0.102909754	0.079672438
ENSGALG00010009992	0.635183038	0.020400828
ENSGALG00010009993	0.468170667	0.207143226
ENSGALG00010009995	0.431064742	0.102329685
ENSGALG00010009998	0.760783024	0.238559423
ENSGALG00010009999	0.059990911	0.036361224
ENSGALG00010010000	0.10909606	0.093996928
ENSGALG00010010001	0.441582771	0.194293404
ENSGALG00010010002	0.400013143	0.107448404
ENSGALG00010010003	0.503621832	0.147992139
ENSGALG00010010005	0.648455666	0.252916926
ENSGALG00010010006	0.922057907	0.204520005
ENSGALG00010010007	0.96564965	0.334497676
ENSGALG00010010008	0.397843669	0.221800636
ENSGALG00010010009	0.718689834	0.289534957
ENSGALG00010010011	0.659534851	0.085162371
ENSGALG00010010012	0.140866473	0.113174349
ENSGALG00010010014	0.498236548	0.235624701
ENSGALG00010010017	0.74738394	0.372555998
ENSGALG00010010019	0.988612995	0.302037487
ENSGALG00010010021	0.573884494	0.200277229
ENSGALG00010010022	0.928830336	0.364577207
ENSGALG00010010025	0.956287568	0.119932951
ENSGALG00010010030	0.321092151	0.305973755
ENSGALG00010010032	0.328574586	0.266567811
ENSGALG00010010035	0.839755983	0.330407041
ENSGALG00010010037	0.899425395	0.27792012
ENSGALG00010010038	0.810322271	0.264881561
ENSGALG00010010039	0.333759773	0.519918642
ENSGALG00010010040	0.208550923	0.676301294
ENSGALG00010010042	0.204269594	0.113864108
ENSGALG00010010044	0.265558089	0.028821141
ENSGALG00010010046	0.155834133	0.213121363
ENSGALG00010010047	0.530506168	0.364260746
ENSGALG00010010049	0.121549302	0.471566601
ENSGALG00010010051	0.207064507	0.058100135
ENSGALG00010010052	0.942899116	0.343823095
ENSGALG00010010053	0.934019782	0.30056276
ENSGALG00010010054	0.220380257	0.287338482
ENSGALG00010010056	0.848970522	0.178884709
ENSGALG00010010057	0.899444045	0.287506077
ENSGALG00010010058	0.271701261	0.072417625
ENSGALG00010010059	0.741840525	0.137963246
ENSGALG00010010060	0.031716562	0.057041277
ENSGALG00010010061	0.973900372	0.322501076
ENSGALG00010010062	0.188894633	0.116841825
ENSGALG00010010063	0.176677727	0.092693733
ENSGALG00010010064	0.801416667	0.175489273
ENSGALG00010010066	0.389501229	0.174238696
ENSGALG00010010068	0.132187787	0.179344
ENSGALG00010010070	0.60025556	0.17665402
ENSGALG00010010071	0.12066259	0.310632514
ENSGALG00010010074	0.285272007	0.109169955

ENSGALG00010010075	0.21893235	0.196486431
ENSGALG00010010076	0.93825124	0.348238333
ENSGALG00010010077	0.25875217	0.000638035
ENSGALG00010010080	0.99371772	0.334242988
ENSGALG00010010081	0.499268466	0.058931645
ENSGALG00010010082	0.58781186	0.387549919
ENSGALG00010010083	0.545063688	0.108129729
ENSGALG00010010084	0.645945062	0.040118824
ENSGALG00010010085	0.804441753	0.441967055
ENSGALG00010010088	0.461628388	0.201893452
ENSGALG00010010090	0.495600535	0.131676095
ENSGALG00010010092	0.49474283	0.15909416
ENSGALG00010010093	0.104691583	0.151088187
ENSGALG00010010094	0.989491252	0.361107675
ENSGALG00010010095	0.926637164	0.270032831
ENSGALG00010010096	0.200570274	0.091667991
ENSGALG00010010097	0.375287285	0.114121296
ENSGALG00010010099	0.222831216	0.180412963
ENSGALG00010010100	0.935892838	0.310811945
ENSGALG00010010101	0.90396031	0.332103188
ENSGALG00010010102	0.608853717	0.190098519
ENSGALG00010010103	0.957003037	0.322747939
ENSGALG00010010105	0.381314909	0.145557719
ENSGALG00010010106	0.148668272	0.280013144
ENSGALG00010010107	0.756290286	0.134690539
ENSGALG00010010108	0.480703662	0.130562937
ENSGALG00010010109	0.615837864	0.022509563
ENSGALG00010010110	0.432179255	0.118617507
ENSGALG00010010111	0.961152441	0.335195163
ENSGALG00010010112	0.370492735	0.206325946
ENSGALG00010010115	0.995915512	0.320157712
ENSGALG00010010116	0.526578179	0.13496119
ENSGALG00010010118	0.371261245	0.110780929
ENSGALG00010010119	0.325549722	0.228789892
ENSGALG00010010120	0.200045717	0.006774396
ENSGALG00010010122	0.656458728	0.496052869
ENSGALG00010010123	0.672273613	0.228352865
ENSGALG00010010124	0.907341166	0.362526778
ENSGALG00010010125	0.861589839	0.246409383
ENSGALG00010010126	0.077603677	0.146292989
ENSGALG00010010127	0.083349757	0.063704233
ENSGALG00010010129	0.46465774	0.185432666
ENSGALG00010010132	0.622439192	0.057527177
ENSGALG00010010133	0.897174546	0.271199514
ENSGALG00010010134	0.825005119	0.297910563
ENSGALG00010010136	0.960013795	0.467689433
ENSGALG00010010137	0.021520484	0.110470564
ENSGALG00010010139	0.330068334	0.11208283
ENSGALG00010010140	0.064991489	0.142576448
ENSGALG00010010141	0.270451009	0.090541667
ENSGALG00010010142	0.334801588	0.14615457
ENSGALG00010010144	0.627485497	0.358635038
ENSGALG00010010145	0.588780923	0.197935799
ENSGALG00010010146	0.93390512	0.386131463
ENSGALG00010010147	0.831948738	0.277404393
ENSGALG00010010148	0.062616764	0.025989509

ENSGALG00010010150	0.447267761	0.035535305
ENSGALG00010010151	0.452822474	0.311204231
ENSGALG00010010153	0.974746203	0.334838112
ENSGALG00010010154	0.799215469	0.300358157
ENSGALG00010010155	0.633139237	0.177684054
ENSGALG00010010156	0.973289077	0.315277365
ENSGALG00010010157	0.763392333	0.25398213
ENSGALG00010010159	0.52420736	0.027891208
ENSGALG00010010160	0.30027592	0.281739051
ENSGALG00010010161	0.887836477	0.284871424
ENSGALG00010010162	0.838336247	0.267889163
ENSGALG00010010164	0.967976116	0.206156454
ENSGALG00010010165	0.788048749	0.262322415
ENSGALG00010010167	0.473958231	0.29145015
ENSGALG00010010168	0.110714723	0.379244672
ENSGALG00010010170	0.487356268	0.202687677
ENSGALG00010010171	0.858141178	0.108929613
ENSGALG00010010175	0.894276233	0.347141037
ENSGALG00010010176	0.208550923	0.676301294
ENSGALG00010010177	0.270542518	0.179823095
ENSGALG00010010178	0.424854426	0.232233752
ENSGALG00010010179	0.75970069	0.235982887
ENSGALG00010010181	0.402628427	0.005149517
ENSGALG00010010183	0.885620908	0.296768766
ENSGALG00010010185	0.38335127	0.333419348
ENSGALG00010010186	0.879351092	0.330034269
ENSGALG00010010187	0.848655989	0.335509533
ENSGALG00010010188	0.977310893	0.288769402
ENSGALG00010010190	0.871753921	0.437906482
ENSGALG00010010191	0.402611391	0.240573049
ENSGALG00010010193	0.265558089	0.028821141
ENSGALG00010010195	0.972080356	0.368662327
ENSGALG00010010196	0.272381648	0.201609916
ENSGALG00010010197	0.992186632	0.313825112
ENSGALG00010010198	0.333298903	0.144818888
ENSGALG00010010201	0.644426007	0.329039512
ENSGALG00010010203	0.260711655	0.163637639
ENSGALG00010010205	0.962593803	0.212056088
ENSGALG00010010206	0.974143684	0.385205465
ENSGALG00010010213	0.115171987	0.264511384
ENSGALG00010010214	0.97800634	0.331408464
ENSGALG00010010215	0.082195976	0.006415488
ENSGALG00010010216	0.775520993	0.48478509
ENSGALG00010010218	0.557209524	0.237419516
ENSGALG00010010219	0.203991683	0.058221925
ENSGALG00010010220	0.264239718	0.073427965
ENSGALG00010010222	0.975649525	0.395992993
ENSGALG00010010223	0.843198081	0.188208144
ENSGALG00010010224	0.547650154	0.287696373
ENSGALG00010010225	0.382141826	0.113944492
ENSGALG00010010226	0.542647442	0.375289364
ENSGALG00010010227	0.208550923	0.676301294
ENSGALG00010010228	0.270192668	0.075397207
ENSGALG00010010231	0.799132784	0.434186817
ENSGALG00010010232	0.150267348	0.115512342
ENSGALG00010010233	0.581496718	0.199799195

ENSGALG00010010234	0.962678153	0.388906688
ENSGALG00010010239	0.339444113	0.045741665
ENSGALG00010010240	0.986252846	0.340166558
ENSGALG00010010241	0.842767094	0.3009802
ENSGALG00010010243	0.928880498	0.275649245
ENSGALG00010010244	0.490568407	0.142044933
ENSGALG00010010245	0.6070394	0.211842667
ENSGALG00010010246	0.974402486	0.371428418
ENSGALG00010010248	0.254896197	0.164745959
ENSGALG00010010249	0.8957164	0.316869961
ENSGALG00010010250	0.466282844	0.382935483
ENSGALG00010010251	0.272036314	0.190020678
ENSGALG00010010252	0.798300157	0.160941289
ENSGALG00010010253	0.955606639	0.252266645
ENSGALG00010010254	0.863810433	0.082835086
ENSGALG00010010255	0.909957295	0.437195285
ENSGALG00010010256	0.879438376	0.304536919
ENSGALG00010010257	0.814253463	0.288294583
ENSGALG00010010258	0.492471405	0.107463898
ENSGALG00010010259	0.953060639	0.387708267
ENSGALG00010010260	0.280792744	0.320044908
ENSGALG00010010261	0.325694796	0.462134608
ENSGALG00010010262	0.147302884	0.02861714
ENSGALG00010010263	0.902999789	0.26420622
ENSGALG00010010264	0.53209222	0.168828463
ENSGALG00010010266	0.343056976	0.099874359
ENSGALG00010010267	0.821416696	0.180775312
ENSGALG00010010268	0.38050694	0.099805136
ENSGALG00010010270	0.879038168	0.201211431
ENSGALG00010010271	0.610079168	0.021953707
ENSGALG00010010273	0.91420573	0.308801344
ENSGALG00010010274	0.842028475	0.027453206
ENSGALG00010010275	0.952683882	0.136993289
ENSGALG00010010276	0.73497581	0.182319894
ENSGALG00010010277	0.262922434	0.138601464
ENSGALG00010010278	0.484350639	0.398650629
ENSGALG00010010279	0.824599223	0.368033645
ENSGALG00010010280	0.921518735	0.303572303
ENSGALG00010010281	0.404167701	0.144831306
ENSGALG00010010282	0.083956046	0.072666279
ENSGALG00010010284	0.907567865	0.162851466
ENSGALG00010010285	0.968993282	0.215155932
ENSGALG00010010286	0.038961094	0.119686046
ENSGALG00010010287	0.972499735	0.372863183
ENSGALG00010010288	0.942963964	0.332108653
ENSGALG00010010289	0.660770539	0.218201639
ENSGALG00010010290	0.775061608	0.163967904
ENSGALG00010010291	0.313994451	0.071454208
ENSGALG00010010292	0.40785333	0.157127562
ENSGALG00010010293	0.643284367	0.345514057
ENSGALG00010010294	0.284144401	0.078500337
ENSGALG00010010295	0.988225539	0.329586732
ENSGALG00010010296	0.67851627	0.225729618
ENSGALG00010010297	0.397165244	0.108993322
ENSGALG00010010298	0.540668147	0.071592375
ENSGALG00010010299	0.77482881	0.168606611

ENSGALG00010010300	0.920336957	0.397912356
ENSGALG00010010301	0.704841285	0.158761891
ENSGALG00010010302	0.053435897	0.200042229
ENSGALG00010010303	0.97573173	0.286732021
ENSGALG00010010304	0.527403248	0.395275982
ENSGALG00010010305	0.982661346	0.263564983
ENSGALG00010010306	0.48382548	0.145996577
ENSGALG00010010307	0.320729011	0.144028995
ENSGALG00010010308	0.494377897	0.166144269
ENSGALG00010010309	0.81170232	0.404853121
ENSGALG00010010310	0.957182283	0.300244766
ENSGALG00010010311	0.922412239	0.389785751
ENSGALG00010010312	0.466243047	0.142226056
ENSGALG00010010313	0.804835157	0.18419454
ENSGALG00010010314	0.765645483	0.231097259
ENSGALG00010010315	0.421993023	0.219740936
ENSGALG00010010316	0.367202845	0.454724026
ENSGALG00010010317	0.460148782	0.2896749
ENSGALG00010010320	0.74876301	0.234544452
ENSGALG00010010321	0.759272841	0.184905069
ENSGALG00010010322	0.92807649	0.261486134
ENSGALG00010010323	0.992756385	0.32516058
ENSGALG00010010324	0.370787514	0.160871034
ENSGALG00010010325	0.313216017	0.204511642
ENSGALG00010010326	0.97896847	0.350637503
ENSGALG00010010327	0.988203436	0.275279402
ENSGALG00010010328	0.08548889	0.145662585
ENSGALG00010010330	0.929193865	0.304986006
ENSGALG00010010331	0.393915379	0.139646271
ENSGALG00010010333	0.809920472	0.249471472
ENSGALG00010010334	0.797066443	0.296878094
ENSGALG00010010335	0.842166879	0.249920826
ENSGALG00010010336	0.911159036	0.257005606
ENSGALG00010010337	0.921176741	0.354337892
ENSGALG00010010338	0.713946074	0.29789814
ENSGALG00010010339	0.390553776	0.106303683
ENSGALG00010010341	0.441737496	0.369076841
ENSGALG00010010342	0.748539199	0.361594409
ENSGALG00010010344	0.343342293	0.423010613
ENSGALG00010010345	0.945998276	0.178375286
ENSGALG00010010346	0.440873949	0.093501618
ENSGALG00010010347	0.904582681	0.205589308
ENSGALG00010010348	0.176956913	0.465439095
ENSGALG00010010349	0.631400194	0.122557308
ENSGALG00010010351	0.744936869	0.238792485
ENSGALG00010010352	0.464656865	0.468668024
ENSGALG00010010353	0.888449997	0.319380767
ENSGALG00010010355	0.466130064	0.167759686
ENSGALG00010010356	0.977602473	0.330486734
ENSGALG00010010357	0.835086621	0.297910523
ENSGALG00010010358	0.337333824	0.008880196
ENSGALG00010010360	0.433328733	0.337858113
ENSGALG00010010361	0.362967989	0.1879193
ENSGALG00010010365	0.068192757	0.007193302
ENSGALG00010010366	0.693991922	0.270793962
ENSGALG00010010367	0.067815929	0.164296756

ENSGALG00010010368	0.367549616	0.036322225
ENSGALG00010010369	0.835307142	0.674535491
ENSGALG00010010370	0.545041237	0.115317641
ENSGALG00010010371	0.84541585	0.327716732
ENSGALG00010010373	0.648134887	0.218056737
ENSGALG00010010374	0.967233056	0.33270684
ENSGALG00010010376	0.386681857	0.128510075
ENSGALG00010010378	0.467504183	0.264594505
ENSGALG00010010379	0.647684244	0.19267364
ENSGALG00010010381	0.944353341	0.214482528
ENSGALG00010010382	0.989547267	0.374853375
ENSGALG00010010383	0.43092684	0.38683132
ENSGALG00010010384	0.91732754	0.280389077
ENSGALG00010010385	0.741781307	0.492617039
ENSGALG00010010386	0.908883194	0.263230451
ENSGALG00010010388	0.483524641	0.406518875
ENSGALG00010010389	0.736039168	0.161888867
ENSGALG00010010390	0.782175755	0.445500326
ENSGALG00010010391	0.023242472	0.240119338
ENSGALG00010010392	0.390721088	0.058000471
ENSGALG00010010393	0.202140821	0.385294898
ENSGALG00010010394	0.867562318	0.358119735
ENSGALG00010010396	0.226564475	0.120476896
ENSGALG00010010397	0.853970782	0.185182525
ENSGALG00010010399	0.843618685	0.294623406
ENSGALG00010010401	0.070383912	0.185963327
ENSGALG00010010402	0.277566769	0.107857127
ENSGALG00010010404	0.937505984	0.400601285
ENSGALG00010010405	0.912641225	0.243159298
ENSGALG00010010406	0.305130081	0.10220604
ENSGALG00010010407	0.85315329	0.270158266
ENSGALG00010010408	0.442134896	0.064111828
ENSGALG00010010409	0.219386918	0.079388121
ENSGALG00010010410	0.988842782	0.339691355
ENSGALG00010010411	0.958142053	0.220452102
ENSGALG00010010415	0.459864997	0.246454025
ENSGALG00010010416	0.951612463	0.321323993
ENSGALG00010010417	0.641498561	0.22856668
ENSGALG00010010418	0.006923441	0.126535936
ENSGALG00010010419	0.144177663	0.516759034
ENSGALG00010010420	0.67701722	0.169625866
ENSGALG00010010421	0.968946288	0.314986531
ENSGALG00010010422	0.990009209	0.342948609
ENSGALG00010010423	0.911723553	0.263442434
ENSGALG00010010425	0.336367567	0.030172521
ENSGALG00010010430	0.726775044	0.274928554
ENSGALG00010010431	0.939485136	0.402666393
ENSGALG00010010434	0.950818722	0.295518623
ENSGALG00010010435	0.973095436	0.384187225
ENSGALG00010010438	0.988456934	0.322914307
ENSGALG00010010439	0.939190105	0.311982415
ENSGALG00010010444	0.477723379	0.270201543
ENSGALG00010010445	0.992822808	0.326837956
ENSGALG00010010446	0.030155467	0.225953914
ENSGALG00010010447	0.95126183	0.328118317
ENSGALG00010010448	0.076519665	0.042599145

ENSGALG00010010449	0.269272437	0.073182666
ENSGALG00010010450	0.51392991	0.257707667
ENSGALG00010010451	0.940516614	0.420617156
ENSGALG00010010452	0.496129098	0.136745135
ENSGALG00010010453	0.307179697	0.307925909
ENSGALG00010010456	0.215913373	0.29558641
ENSGALG00010010457	0.566072598	0.049290258
ENSGALG00010010460	0.980871397	0.346206056
ENSGALG00010010461	0.965577063	0.38182764
ENSGALG00010010462	0.966677536	0.441451174
ENSGALG00010010463	0.897994	0.295443713
ENSGALG00010010464	0.724147819	0.367546889
ENSGALG00010010465	0.672255105	0.579468498
ENSGALG00010010466	0.494125689	0.254787054
ENSGALG00010010467	0.899694175	0.187770982
ENSGALG00010010468	0.441476394	0.02844079
ENSGALG00010010469	0.255000842	0.231142866
ENSGALG00010010470	0.484436816	0.071658837
ENSGALG00010010471	0.718090778	0.450854931
ENSGALG00010010472	0.755051928	0.445740667
ENSGALG00010010473	0.861881829	0.228319641
ENSGALG00010010474	0.800208511	0.35834985
ENSGALG00010010475	0.134631022	0.264915811
ENSGALG00010010476	0.266137114	0.107784983
ENSGALG00010010477	0.910457955	0.245279862
ENSGALG00010010479	0.117583042	0.267532169
ENSGALG00010010480	0.187514554	0.108479243
ENSGALG00010010482	0.198700219	0.455786488
ENSGALG00010010483	0.586716586	0.060003899
ENSGALG00010010484	0.386902872	0.202119071
ENSGALG00010010486	0.51175427	0.022702561
ENSGALG00010010487	0.841327303	0.238285078
ENSGALG00010010488	0.251284199	0.12166773
ENSGALG00010010490	0.540886269	0.256059867
ENSGALG00010010491	0.36047241	0.115986332
ENSGALG00010010494	0.977415912	0.263657818
ENSGALG00010010495	0.867506041	0.331415844
ENSGALG00010010496	0.987454172	0.317333478
ENSGALG00010010499	0.782658317	0.272250105
ENSGALG00010010504	0.140956203	0.026059303
ENSGALG00010010505	0.856927641	0.428363001
ENSGALG00010010506	0.128434457	0.147990742
ENSGALG00010010511	0.38137981	0.201921904
ENSGALG00010010512	0.984164104	0.273914011
ENSGALG00010010515	0.955677823	0.356869396
ENSGALG00010010516	0.694378442	0.272160328
ENSGALG00010010517	0.208550923	0.676301294
ENSGALG00010010520	0.474616183	0.173292476
ENSGALG00010010521	0.924823984	0.135834933
ENSGALG00010010523	0.899112653	0.076261462
ENSGALG00010010524	0.373119456	0.207838807
ENSGALG00010010526	0.47552848	0.187758975
ENSGALG00010010528	0.989690058	0.354670728
ENSGALG00010010532	0.381608622	0.094262957
ENSGALG00010010534	0.871922375	0.314426771
ENSGALG00010010535	0.464193274	0.175432391

ENSGALG00010010536	0.000893823	0.500599188
ENSGALG00010010537	0.379394869	0.170237467
ENSGALG00010010540	0.21193208	0.506243928
ENSGALG00010010541	0.640135105	0.299555473
ENSGALG00010010542	0.523973097	0.275621888
ENSGALG00010010543	0.844850896	0.089227604
ENSGALG00010010544	0.8239731	0.404359798
ENSGALG00010010546	0.987858989	0.325193447
ENSGALG00010010547	0.931316379	0.206290396
ENSGALG00010010548	0.048532643	0.007975711
ENSGALG00010010549	0.174807695	0.132889743
ENSGALG00010010550	0.388060988	0.153227889
ENSGALG00010010551	0.678865142	0.357472548
ENSGALG00010010552	0.277566769	0.107857127
ENSGALG00010010553	0.569566071	0.180296093
ENSGALG00010010554	0.609203231	0.194366996
ENSGALG00010010555	0.494157879	0.195125042
ENSGALG00010010557	0.017246661	0.459476749
ENSGALG00010010558	0.65518014	0.017858217
ENSGALG00010010559	0.431646998	0.267777202
ENSGALG00010010561	0.923404622	0.196914684
ENSGALG00010010563	0.095474074	0.13183335
ENSGALG00010010564	0.56646333	0.445221062
ENSGALG00010010566	0.10738367	0.206840404
ENSGALG00010010567	0.029318055	0.146600019
ENSGALG00010010568	0.600774745	0.377683421
ENSGALG00010010569	0.580724372	0.194795499
ENSGALG00010010571	0.334984929	0.421560278
ENSGALG00010010572	0.605931924	0.273959359
ENSGALG00010010573	0.90243017	0.302373767
ENSGALG00010010575	0.072898767	0.415947721
ENSGALG00010010577	0.993037651	0.326659453
ENSGALG00010010578	0.975437533	0.308740799
ENSGALG00010010582	0.632946553	0.375081354
ENSGALG00010010583	0.36437197	0.115555231
ENSGALG00010010584	0.695334012	0.089498995
ENSGALG00010010585	0.451792579	0.299152714
ENSGALG00010010586	0.77958151	0.179483936
ENSGALG00010010587	0.872068773	0.367706934
ENSGALG00010010588	0.651601887	0.236319674
ENSGALG00010010589	0.583993759	0.216827313
ENSGALG00010010591	0.591493884	0.184688407
ENSGALG00010010592	0.078194497	0.171196888
ENSGALG00010010593	0.87993504	0.331117203
ENSGALG00010010595	0.556633076	0.241380052
ENSGALG00010010596	0.772482607	0.488509079
ENSGALG00010010597	0.385953062	0.133261917
ENSGALG00010010598	0.440528123	0.171424878
ENSGALG00010010599	0.984517352	0.264804649
ENSGALG00010010600	0.19264856	0.407560136
ENSGALG00010010601	0.073355415	0.028330384
ENSGALG00010010602	0.311915895	0.474009313
ENSGALG00010010603	0.123292688	0.248348235
ENSGALG00010010604	0.982058213	0.372295311
ENSGALG00010010605	0.626681621	0.035416091
ENSGALG00010010612	0.567993953	0.172709698

ENSGALG00010010614	0.401641365	0.107199432
ENSGALG00010010615	0.530128944	0.063158754
ENSGALG00010010618	0.94463132	0.342132724
ENSGALG00010010619	0.498651561	0.027016777
ENSGALG00010010620	0.925028383	0.268799123
ENSGALG00010010621	0.425321441	0.082218767
ENSGALG00010010622	0.136793605	0.094261048
ENSGALG00010010623	0.900469846	0.388864009
ENSGALG00010010624	0.10717702	0.143906514
ENSGALG00010010625	0.645377603	0.23498728
ENSGALG00010010626	0.716369083	0.12848447
ENSGALG00010010628	0.044601559	0.198257206
ENSGALG00010010629	0.110714723	0.379244672
ENSGALG00010010630	0.472394899	0.257481238
ENSGALG00010010631	0.462237741	0.444409777
ENSGALG00010010632	0.743726272	0.224438583
ENSGALG00010010633	0.992533498	0.347552159
ENSGALG00010010635	0.94216117	0.325772455
ENSGALG00010010638	0.157578276	0.240052996
ENSGALG00010010641	0.122858901	0.224023935
ENSGALG00010010642	0.627951597	0.232879898
ENSGALG00010010645	0.145203817	0.352497218
ENSGALG00010010646	0.924579814	0.378928039
ENSGALG00010010648	0.263394289	0.036129588
ENSGALG00010010650	0.304133466	0.450346192
ENSGALG00010010651	0.828952384	0.111123669
ENSGALG00010010653	0.877645913	0.158653242
ENSGALG00010010655	0.76310708	0.55706448
ENSGALG00010010657	0.570939742	0.338644284
ENSGALG00010010659	0.864154161	0.351611508
ENSGALG00010010660	0.778087112	0.301725836
ENSGALG00010010661	0.858317619	0.166548343
ENSGALG00010010662	0.918643981	0.336433881
ENSGALG00010010663	0.95338759	0.247694201
ENSGALG00010010666	0.767293643	0.241898791
ENSGALG00010010667	0.511041481	0.638841585
ENSGALG00010010671	0.827964961	0.207248657
ENSGALG00010010672	0.157487356	0.242311849
ENSGALG00010010673	0.11781451	0.122917091
ENSGALG00010010677	0.581375575	0.111169039
ENSGALG00010010678	0.78852219	0.329694244
ENSGALG00010010679	0.172415916	0.257085955
ENSGALG00010010680	0.260632516	0.277948498
ENSGALG00010010681	0.560700681	0.140712786
ENSGALG00010010682	0.960932038	0.371935943
ENSGALG00010010683	0.54322997	0.22551485
ENSGALG00010010685	0.942769847	0.268653976
ENSGALG00010010686	0.572414378	0.033218976
ENSGALG00010010688	0.993829661	0.313464295
ENSGALG00010010689	0.925826942	0.247248019
ENSGALG00010010690	0.97471422	0.318439425
ENSGALG00010010692	0.130515589	0.11260751
ENSGALG00010010693	0.954797545	0.263202919
ENSGALG00010010695	0.005068257	0.483321659
ENSGALG00010010696	0.47239257	0.121930621
ENSGALG00010010697	0.933689528	0.30047899

ENSGALG00010010698	0.490881081	0.249287919
ENSGALG00010010699	0.888430517	0.265200709
ENSGALG00010010702	0.266137114	0.107784983
ENSGALG00010010703	0.708149714	0.533898171
ENSGALG00010010704	0.755306552	0.182049347
ENSGALG00010010705	0.112954512	0.102150984
ENSGALG00010010706	0.20221497	0.144419279
ENSGALG00010010707	0.564599148	0.257323578
ENSGALG00010010708	0.184592678	0.041408745
ENSGALG00010010709	0.632817849	0.0444547
ENSGALG00010010710	0.898949752	0.232220125
ENSGALG00010010711	0.417836688	0.042675186
ENSGALG00010010712	0.702978446	0.256859938
ENSGALG00010010713	0.769746468	0.207952679
ENSGALG00010010714	0.283468811	0.33822564
ENSGALG00010010715	0.942330514	0.31865944
ENSGALG00010010716	0.021308589	0.164876077
ENSGALG00010010717	0.115957159	0.237961918
ENSGALG00010010718	0.042130527	0.126956878
ENSGALG00010010719	0.969222919	0.291886202
ENSGALG00010010720	0.578552359	0.409851544
ENSGALG00010010722	0.784989458	0.054382636
ENSGALG00010010724	0.079926982	0.497962761
ENSGALG00010010725	0.906168704	0.289213489
ENSGALG00010010728	0.992137039	0.347025217
ENSGALG00010010729	0.869587676	0.266041964
ENSGALG00010010730	0.991902511	0.329244513
ENSGALG00010010732	0.599378721	0.176728778
ENSGALG00010010733	0.784711074	0.205339708
ENSGALG00010010734	0.898534749	0.17903708
ENSGALG00010010735	0.110916976	0.224512995
ENSGALG00010010736	0.994254484	0.335670804
ENSGALG00010010740	0.922023783	0.295038879
ENSGALG00010010742	0.962681958	0.262251415
ENSGALG00010010743	0.188941272	0.300767939
ENSGALG00010010745	0.077170756	0.047187224
ENSGALG00010010748	0.927263744	0.388757951
ENSGALG00010010749	0.969140232	0.395162711
ENSGALG00010010750	0.492557799	0.141952549
ENSGALG00010010751	0.960892644	0.333547429
ENSGALG00010010752	0.338341761	0.318940424
ENSGALG00010010753	0.270515323	0.030303391
ENSGALG00010010754	0.514718255	0.107977883
ENSGALG00010010756	0.224230201	0.094815562
ENSGALG00010010759	0.354910093	0.305193946
ENSGALG00010010760	0.903634964	0.436157623
ENSGALG00010010761	0.952394607	0.207015151
ENSGALG00010010762	0.70012884	0.084377748
ENSGALG00010010763	0.794797956	0.129600855
ENSGALG00010010764	0.606101407	0.013803163
ENSGALG00010010765	0.99289244	0.329374474
ENSGALG00010010766	0.772793423	0.226607151
ENSGALG00010010767	0.363600201	0.059295317
ENSGALG00010010768	0.905799031	0.378185814
ENSGALG00010010769	0.969294847	0.304929662
ENSGALG00010010770	0.594017655	0.240067851

ENSGALG00010010771	0.892001671	0.214029377
ENSGALG00010010772	0.982277316	0.33764957
ENSGALG00010010773	0.863265974	0.35411474
ENSGALG00010010774	0.92741256	0.255831936
ENSGALG00010010775	0.270451009	0.090541667
ENSGALG00010010776	0.6711272	0.068698517
ENSGALG00010010777	0.863444655	0.149901445
ENSGALG00010010778	0.579907645	0.168644607
ENSGALG00010010779	0.972828284	0.258515286
ENSGALG00010010780	0.006740375	0.0038078
ENSGALG00010010781	0.910284706	0.279521415
ENSGALG00010010783	0.856776423	0.225125594
ENSGALG00010010784	0.71396034	0.349059276
ENSGALG00010010786	0.762974598	0.026027191
ENSGALG00010010787	0.383708263	0.151865425
ENSGALG00010010788	0.764426994	0.197117023
ENSGALG00010010789	0.314845355	0.218965371
ENSGALG00010010790	0.128609649	0.187071108
ENSGALG00010010791	0.396587792	0.01257238
ENSGALG00010010792	0.785786881	0.244294148
ENSGALG00010010796	0.930718531	0.448163143
ENSGALG00010010799	0.562474264	0.12893268
ENSGALG00010010800	0.927419074	0.256116575
ENSGALG00010010802	0.731358839	0.282648079
ENSGALG00010010803	0.781892521	0.015499224
ENSGALG00010010804	0.614196805	0.240945378
ENSGALG00010010805	0.711560292	0.238538958
ENSGALG00010010806	0.00853842	0.154655808
ENSGALG00010010807	0.543718243	0.147179149
ENSGALG00010010809	0.256657413	0.406164683
ENSGALG00010010810	0.811002889	0.2694747
ENSGALG00010010811	0.501362731	0.419745076
ENSGALG00010010812	0.46436837	0.184836279
ENSGALG00010010813	0.987611591	0.338868371
ENSGALG00010010814	0.358971653	0.072654586
ENSGALG00010010816	0.934302778	0.303142515
ENSGALG00010010817	0.48044056	0.138207522
ENSGALG00010010820	0.871534034	0.326732962
ENSGALG00010010821	0.867297337	0.255947293
ENSGALG00010010822	0.510538768	0.104119995
ENSGALG00010010823	0.89159243	0.415796756
ENSGALG00010010824	0.947633004	0.326360587
ENSGALG00010010825	0.797175966	0.192947366
ENSGALG00010010826	0.565757076	0.18615583
ENSGALG00010010827	0.459318714	0.096452807
ENSGALG00010010828	0.84666685	0.547300805
ENSGALG00010010829	0.799249775	0.190702763
ENSGALG00010010831	0.972912935	0.228946708
ENSGALG00010010832	0.678061371	0.118568669
ENSGALG00010010834	0.726345648	0.195630679
ENSGALG00010010836	0.938070344	0.311440048
ENSGALG00010010837	0.956370595	0.244161524
ENSGALG00010010838	0.41139081	0.248450937
ENSGALG00010010840	0.916204275	0.381291439
ENSGALG00010010841	0.955698019	0.408648677
ENSGALG00010010843	0.959656626	0.370295001

ENSGALG00010010844	0.817777449	0.211957456
ENSGALG00010010845	0.648746617	0.306172142
ENSGALG00010010846	0.154373696	0.138164639
ENSGALG00010010848	0.5570377	0.412482138
ENSGALG00010010849	0.896996054	0.13960585
ENSGALG00010010850	0.965247639	0.332054943
ENSGALG00010010851	0.898585394	0.456521892
ENSGALG00010010852	0.725570728	0.27774591
ENSGALG00010010853	0.547640017	0.150537391
ENSGALG00010010854	0.795960479	0.301727216
ENSGALG00010010855	0.947164504	0.353818398
ENSGALG00010010856	0.211761593	0.496217444
ENSGALG00010010857	0.90680715	0.370877877
ENSGALG00010010859	0.802578946	0.181459942
ENSGALG00010010860	0.761473925	0.36967377
ENSGALG00010010861	0.032306874	0.011769149
ENSGALG00010010862	0.522734381	0.048509883
ENSGALG00010010863	0.266137114	0.107784983
ENSGALG00010010864	0.392009975	0.054308127
ENSGALG00010010865	0.95587954	0.288199408
ENSGALG00010010866	0.883709269	0.316490873
ENSGALG00010010867	0.544727614	0.26619074
ENSGALG00010010868	0.870829181	0.201956712
ENSGALG00010010869	0.963919581	0.338000007
ENSGALG00010010870	0.197686952	0.23925456
ENSGALG00010010871	0.969044703	0.322929342
ENSGALG00010010872	0.311289807	0.13851383
ENSGALG00010010873	0.803628056	0.209743719
ENSGALG00010010874	0.910811549	0.174397516
ENSGALG00010010875	0.496122232	0.304116478
ENSGALG00010010876	0.938469825	0.34625905
ENSGALG00010010880	0.930686606	0.297346384
ENSGALG00010010882	0.923925237	0.265219753
ENSGALG00010010884	0.966001671	0.278592135
ENSGALG00010010885	0.888567213	0.193351495
ENSGALG00010010887	0.898871148	0.195269894
ENSGALG00010010888	0.978285802	0.340975082
ENSGALG00010010889	0.913990408	0.391141534
ENSGALG00010010890	0.131375389	0.074747141
ENSGALG00010010891	0.876765123	0.478438644
ENSGALG00010010892	0.082583701	0.247683759
ENSGALG00010010893	0.907303894	0.288428188
ENSGALG00010010894	0.749535867	0.545435675
ENSGALG00010010895	0.949346816	0.299773287
ENSGALG00010010896	0.940352519	0.23818032
ENSGALG00010010897	0.977766441	0.331062334
ENSGALG00010010900	0.942448025	0.305978252
ENSGALG00010010901	0.124312566	0.130525028
ENSGALG00010010902	0.783154159	0.394866688
ENSGALG00010010903	0.336037988	0.029536793
ENSGALG00010010904	0.991886872	0.310013785
ENSGALG00010010905	0.834114526	0.485415954
ENSGALG00010010906	0.748497843	0.074438699
ENSGALG00010010907	0.102557187	0.376152885
ENSGALG00010010908	0.878035151	0.141216361
ENSGALG00010010909	0.583914086	0.007883887

ENSGALG00010010910	0.94360391	0.290455337
ENSGALG00010010911	0.918228721	0.085825717
ENSGALG00010010912	0.90395677	0.490720861
ENSGALG00010010913	0.80378905	0.055190459
ENSGALG00010010914	0.480800697	0.116510583
ENSGALG00010010915	0.932582933	0.194012817
ENSGALG00010010916	0.370844335	0.111056801
ENSGALG00010010917	0.952176264	0.277145861
ENSGALG00010010918	0.741068786	0.118797156
ENSGALG00010010919	0.42909162	0.286061018
ENSGALG00010010920	0.794606715	0.176965983
ENSGALG00010010921	0.767115253	0.307197937
ENSGALG00010010922	0.965218179	0.274388645
ENSGALG00010010923	0.461161649	0.341414483
ENSGALG00010010924	0.709765942	0.15242107
ENSGALG00010010925	0.899957307	0.266619675
ENSGALG00010010926	0.429971141	0.081698076
ENSGALG00010010928	0.612868206	0.35438206
ENSGALG00010010929	0.785752995	0.330797131
ENSGALG00010010930	0.297192963	0.952693364
ENSGALG00010010931	0.746988909	0.428952942
ENSGALG00010010932	0.813637683	0.351928898
ENSGALG00010010933	0.858395293	0.049226803
ENSGALG00010010934	0.813801767	0.128719898
ENSGALG00010010935	0.893571242	0.436196953
ENSGALG00010010936	0.354532559	0.228848641
ENSGALG00010010937	0.14366844	0.015026333
ENSGALG00010010938	0.023594528	0.486075456
ENSGALG00010010939	0.712928212	0.314367435
ENSGALG00010010940	0.644161376	0.004376451
ENSGALG00010010941	0.991765688	0.341921979
ENSGALG00010010942	0.818256684	0.050106675
ENSGALG00010010943	0.886856856	0.308193089
ENSGALG00010010945	0.99107781	0.36711738
ENSGALG00010010946	0.571186892	0.241357489
ENSGALG00010010947	0.519607237	0.039196098
ENSGALG00010010948	0.740576262	0.339823605
ENSGALG00010010949	0.970961533	0.288225503
ENSGALG00010010950	0.542411648	0.284586448
ENSGALG00010010951	0.684205002	0.192766142
ENSGALG00010010952	0.549736085	0.088916962
ENSGALG00010010953	0.558021529	0.044062873
ENSGALG00010010954	0.799510222	0.239356481
ENSGALG00010010955	0.725974645	0.441812948
ENSGALG00010010956	0.055947878	0.337049294
ENSGALG00010010957	0.983702574	0.273520107
ENSGALG00010010958	0.721921798	0.264211415
ENSGALG00010010959	0.499524969	0.015914312
ENSGALG00010010960	0.635056119	0.193648766
ENSGALG00010010961	0.304134323	0.47839316
ENSGALG00010010962	0.273984384	0.050918496
ENSGALG00010010963	0.766239006	0.300526816
ENSGALG00010010964	0.062136651	0.466268865
ENSGALG00010010965	0.13727222	0.231536724
ENSGALG00010010966	0.877294119	0.216990649
ENSGALG00010010967	0.971012377	0.29804157

ENSGALG00010010968	0.526596581	0.143439885
ENSGALG00010010970	0.90104692	0.352304369
ENSGALG00010010971	0.799625401	0.612161516
ENSGALG00010010972	0.211807839	0.239083857
ENSGALG00010010973	0.940806445	0.343700565
ENSGALG00010010974	0.656911566	0.02030658
ENSGALG00010010975	0.890684342	0.33297226
ENSGALG00010010976	0.628364438	0.214757661
ENSGALG00010010977	0.595727105	0.369985287
ENSGALG00010010978	0.907222729	0.241673044
ENSGALG00010010979	0.185579348	0.155473945
ENSGALG00010010980	0.891717053	0.401629176
ENSGALG00010010981	0.889512196	0.406450452
ENSGALG00010010982	0.92820358	0.187004358
ENSGALG00010010983	0.945079373	0.291622437
ENSGALG00010010984	0.989011959	0.321621185
ENSGALG00010010985	0.63281873	0.283765074
ENSGALG00010010986	0.884105394	0.29796339
ENSGALG00010010987	0.014947342	0.518180536
ENSGALG00010010988	0.980302985	0.339437472
ENSGALG00010010989	0.674568216	0.004442804
ENSGALG00010010990	0.91369318	0.235216732
ENSGALG00010010991	0.918345529	0.13758421
ENSGALG00010010992	0.859150058	0.324882772
ENSGALG00010010993	0.987599205	0.316843946
ENSGALG00010010994	0.709412509	0.212847841
ENSGALG00010010995	0.981532583	0.269202662
ENSGALG00010010996	0.21010597	0.276127705
ENSGALG00010010997	0.789322557	0.062623142
ENSGALG00010010998	0.989366794	0.291684772
ENSGALG00010011000	0.677300404	0.220783306
ENSGALG00010011001	0.910543421	0.275459656
ENSGALG00010011003	0.686365343	0.330612125
ENSGALG00010011004	0.324322801	0.206306307
ENSGALG00010011006	0.420768286	0.039596267
ENSGALG00010011007	0.910211126	0.24967189
ENSGALG00010011008	0.992999801	0.332791159
ENSGALG00010011009	0.924501384	0.20171074
ENSGALG00010011010	0.979672322	0.347918006
ENSGALG00010011012	0.923731458	0.253514782
ENSGALG00010011013	0.550025861	0.367022777
ENSGALG00010011015	0.836334912	0.209846496
ENSGALG00010011016	0.90816844	0.259327813
ENSGALG00010011017	0.741292928	0.080177093
ENSGALG00010011018	0.982444603	0.321316594
ENSGALG00010011019	0.346137245	0.052068032
ENSGALG00010011020	0.975926427	0.315863802
ENSGALG00010011021	0.031902356	0.12595153
ENSGALG00010011022	0.161163382	0.016467566
ENSGALG00010011024	0.193367476	0.254267559
ENSGALG00010011025	0.899398025	0.195456873
ENSGALG00010011027	0.949519118	0.469678094
ENSGALG00010011028	0.952464848	0.425109713
ENSGALG00010011029	0.981693727	0.323009068
ENSGALG00010011030	0.348032803	0.146426442
ENSGALG00010011031	0.911287855	0.263287087

ENSGALG00010011032	0.938222115	0.220695677
ENSGALG00010011033	0.62160666	0.335949341
ENSGALG00010011034	0.189793615	0.232294369
ENSGALG00010011035	0.394377908	0.086868006
ENSGALG00010011036	0.138943243	0.107072059
ENSGALG00010011037	0.271701261	0.072417625
ENSGALG00010011038	0.763460949	0.144886362
ENSGALG00010011039	0.442444873	0.250720648
ENSGALG00010011040	0.611146259	0.240702414
ENSGALG00010011041	0.356325253	0.179172604
ENSGALG00010011042	0.295918706	0.116306489
ENSGALG00010011043	0.346105038	0.189054728
ENSGALG00010011044	0.994200281	0.363184862
ENSGALG00010011045	0.555473966	0.063745409
ENSGALG00010011046	0.447699804	0.390412082
ENSGALG00010011047	0.579597135	0.036460151
ENSGALG00010011049	0.074437696	0.206309501
ENSGALG00010011050	0.884827153	0.220505378
ENSGALG00010011051	0.93687624	0.30950914
ENSGALG00010011055	0.954935523	0.227427429
ENSGALG00010011056	0.220422002	0.326239399
ENSGALG00010011057	0.955229709	0.308795229
ENSGALG00010011059	0.112249285	0.111729914
ENSGALG00010011061	0.934871877	0.297554434
ENSGALG00010011062	0.778145825	0.269001044
ENSGALG00010011063	0.325669798	0.21699008
ENSGALG00010011064	0.729096974	0.378434106
ENSGALG00010011066	0.379952917	0.147982463
ENSGALG00010011067	0.607501121	0.356335331
ENSGALG00010011069	0.254890033	0.049705713
ENSGALG00010011070	0.277566769	0.107857127
ENSGALG00010011072	0.426065103	0.031702528
ENSGALG00010011074	0.400778541	0.133797302
ENSGALG00010011076	0.250807229	0.097768228
ENSGALG00010011077	0.427791392	0.043040161
ENSGALG00010011080	0.173794527	0.059177695
ENSGALG00010011082	0.503087719	0.174140093
ENSGALG00010011085	0.990146366	0.371535638
ENSGALG00010011086	0.991108077	0.309835848
ENSGALG00010011088	0.311438666	0.529761034
ENSGALG00010011089	0.573888108	0.181656699
ENSGALG00010011090	0.015348999	0.251892033
ENSGALG00010011091	0.074201562	0.108364953
ENSGALG00010011092	0.136190057	0.325015569
ENSGALG00010011093	0.094815613	0.084241463
ENSGALG00010011094	0.763637484	0.168826382
ENSGALG00010011095	0.271207923	0.39718463
ENSGALG00010011096	0.287641251	0.080513677
ENSGALG00010011097	0.116288353	0.426288055
ENSGALG00010011098	0.108780822	0.554743142
ENSGALG00010011099	0.91758024	0.227527927
ENSGALG00010011100	0.39190877	0.131701825
ENSGALG00010011101	0.139690722	0.020290881
ENSGALG00010011102	0.795983124	0.193421974
ENSGALG00010011103	0.042343157	0.133098028
ENSGALG00010011104	0.750684701	0.357108147

ENSGALG00010011106	0.966970386	0.29938174
ENSGALG00010011107	0.860255243	0.221744663
ENSGALG00010011109	0.216433634	0.680598293
ENSGALG00010011111	0.977588735	0.312149792
ENSGALG00010011113	0.15549669	0.176370565
ENSGALG00010011115	0.045918678	0.141100759
ENSGALG00010011116	0.971007189	0.375704136
ENSGALG00010011118	0.963963193	0.372435712
ENSGALG00010011119	0.380420727	0.197432745
ENSGALG00010011121	0.899504066	0.304095946
ENSGALG00010011123	0.744295501	0.239346488
ENSGALG00010011124	0.978747072	0.237210041
ENSGALG00010011125	0.498348919	0.153122678
ENSGALG00010011126	0.867413641	0.287211943
ENSGALG00010011128	0.951004772	0.370724604
ENSGALG00010011129	0.723156764	0.220522551
ENSGALG00010011130	0.318823904	0.190389365
ENSGALG00010011131	0.413731997	0.244509466
ENSGALG00010011133	0.835043203	0.263712153
ENSGALG00010011134	0.300683727	0.461762941
ENSGALG00010011136	0.180815449	0.385470115
ENSGALG00010011139	0.879786739	0.201400126
ENSGALG00010011141	0.022307196	0.119515171
ENSGALG00010011142	0.77195045	0.261841691
ENSGALG00010011143	0.932407794	0.321202208
ENSGALG00010011144	0.947624499	0.318900001
ENSGALG00010011145	0.037918858	0.097372492
ENSGALG00010011146	0.431788101	0.011844284
ENSGALG00010011148	0.845895216	0.33704306
ENSGALG00010011149	0.202485276	0.089313705
ENSGALG00010011150	0.963919674	0.245002676
ENSGALG00010011151	0.901740673	0.389049691
ENSGALG00010011152	0.373792563	0.171264927
ENSGALG00010011153	0.467450891	0.16866517
ENSGALG00010011155	0.673294141	0.240390357
ENSGALG00010011156	0.025124456	0.111798379
ENSGALG00010011157	0.256828349	0.119064955
ENSGALG00010011158	0.188771645	0.172631201
ENSGALG00010011159	0.253114587	0.289572198
ENSGALG00010011161	0.986970894	0.391576256
ENSGALG00010011166	0.69226945	0.275121833
ENSGALG00010011170	0.988283331	0.322022935
ENSGALG00010011171	0.895167849	0.32224306
ENSGALG00010011172	0.078690675	0.085030523
ENSGALG00010011173	0.097545881	0.09040332
ENSGALG00010011175	0.792074867	0.284766732
ENSGALG00010011176	0.57269461	0.218758253
ENSGALG00010011178	0.380187033	0.425708988
ENSGALG00010011181	0.501398143	0.296996655
ENSGALG00010011183	0.706840114	0.478949399
ENSGALG00010011184	0.868444242	0.304726103
ENSGALG00010011187	0.124337726	0.271559216
ENSGALG00010011189	0.270451009	0.090541667
ENSGALG00010011190	0.345699033	0.113595926
ENSGALG00010011191	0.235630588	0.132393411
ENSGALG00010011192	0.891147401	0.298635743

ENSGALG00010011193	0.564851479	0.063105465
ENSGALG00010011194	0.740281365	0.329591037
ENSGALG00010011196	0.984776388	0.319077174
ENSGALG00010011197	0.97671892	0.313526948
ENSGALG00010011198	0.387808463	0.383792675
ENSGALG00010011200	0.458665773	0.33864769
ENSGALG00010011202	0.721348301	0.07094435
ENSGALG00010011203	0.970326751	0.276581634
ENSGALG00010011204	0.977220787	0.290654019
ENSGALG00010011205	0.354882154	0.213412916
ENSGALG00010011206	0.219892668	0.15244855
ENSGALG00010011207	0.122042973	0.145658162
ENSGALG00010011208	0.165783327	0.016949743
ENSGALG00010011210	0.719165354	0.235406697
ENSGALG00010011211	0.699280058	0.23247242
ENSGALG00010011212	0.970183886	0.197158241
ENSGALG00010011213	0.184265256	0.186910607
ENSGALG00010011216	0.565116455	0.186719947
ENSGALG00010011218	0.037193636	0.239393609
ENSGALG00010011222	0.913506308	0.390763596
ENSGALG00010011226	0.286367932	0.089279855
ENSGALG00010011229	0.841348679	0.099312319
ENSGALG00010011231	0.961205224	0.273976316
ENSGALG00010011234	0.797117922	0.398264681
ENSGALG00010011235	0.455430788	0.216643181
ENSGALG00010011237	0.266392752	0.185541021
ENSGALG00010011239	0.956502635	0.433409538
ENSGALG00010011240	0.207654172	0.198718547
ENSGALG00010011244	0.828112575	0.339768268
ENSGALG00010011245	0.31277746	0.230133103
ENSGALG00010011246	0.984342045	0.360587381
ENSGALG00010011247	0.544286551	0.013609731
ENSGALG00010011249	0.05197934	0.14495833
ENSGALG00010011250	0.937065732	0.329973248
ENSGALG00010011251	0.501097353	0.162920055
ENSGALG00010011252	0.490296051	0.147652895
ENSGALG00010011253	0.9111842	0.493223686
ENSGALG00010011254	0.605504776	0.050825784
ENSGALG00010011255	0.688147388	0.22387638
ENSGALG00010011256	0.04146015	0.388741609
ENSGALG00010011259	0.216433634	0.680598293
ENSGALG00010011260	0.966857662	0.319666713
ENSGALG00010011261	0.079220401	0.115137619
ENSGALG00010011262	0.439410272	0.163247078
ENSGALG00010011263	0.504020497	0.141821405
ENSGALG00010011265	0.871000491	0.264805655
ENSGALG00010011267	0.769285353	0.28489452
ENSGALG00010011269	0.85925687	0.226965592
ENSGALG00010011270	0.049631859	0.180551362
ENSGALG00010011271	0.959949576	0.355103704
ENSGALG00010011272	0.325518582	0.03722157
ENSGALG00010011273	0.961211977	0.319178763
ENSGALG00010011274	0.085493117	0.062756127
ENSGALG00010011275	0.840117611	0.352701849
ENSGALG00010011277	0.262547348	0.213242632
ENSGALG00010011278	0.474191153	0.190199575

ENSGALG00010011280	0.919339897	0.451274859
ENSGALG00010011281	0.307293908	0.091015132
ENSGALG00010011282	0.874987912	0.1553329
ENSGALG00010011283	0.593471825	0.617293205
ENSGALG00010011284	0.603952078	0.495642047
ENSGALG00010011285	0.671583657	0.15297013
ENSGALG00010011287	0.800854394	0.13171559
ENSGALG00010011288	0.100359439	0.09323447
ENSGALG00010011290	0.343788084	0.038040212
ENSGALG00010011291	0.378404611	0.21288244
ENSGALG00010011293	0.968598143	0.26668281
ENSGALG00010011294	0.534385641	0.391317532
ENSGALG00010011295	0.511272699	0.334856458
ENSGALG00010011297	0.994456877	0.336051235
ENSGALG00010011299	0.838080534	0.388578998
ENSGALG00010011300	0.871826767	0.324635358
ENSGALG00010011302	0.232568609	0.171703899
ENSGALG00010011303	0.405797923	0.13200104
ENSGALG00010011304	0.934323675	0.297308457
ENSGALG00010011305	0.415324066	0.273122736
ENSGALG00010011306	0.982427014	0.306282425
ENSGALG00010011307	0.12038089	0.196457262
ENSGALG00010011308	0.318329187	0.289623907
ENSGALG00010011311	0.974112338	0.348534751
ENSGALG00010011312	0.851267972	0.268824
ENSGALG00010011313	0.879006223	0.240528159
ENSGALG00010011314	0.519188495	0.254090323
ENSGALG00010011315	0.469664944	0.130078439
ENSGALG00010011316	0.67385913	0.314254299
ENSGALG00010011317	0.829487921	0.293800299
ENSGALG00010011318	0.943167886	0.420494922
ENSGALG00010011321	0.265649194	0.140140023
ENSGALG00010011322	0.987028707	0.358098785
ENSGALG00010011323	0.961745156	0.315964645
ENSGALG00010011326	0.936880783	0.314944881
ENSGALG00010011328	0.367899555	0.330436197
ENSGALG00010011329	0.905030314	0.434606663
ENSGALG00010011331	0.327629778	0.233401589
ENSGALG00010011333	0.063013904	0.264467297
ENSGALG00010011334	0.990398761	0.285408503
ENSGALG00010011335	0.899951789	0.337761914
ENSGALG00010011336	0.67861888	0.179255534
ENSGALG00010011339	0.022736595	0.286672271
ENSGALG00010011340	0.589408393	0.262363724
ENSGALG00010011341	0.107758923	0.067805931
ENSGALG00010011342	0.364899705	0.192638711
ENSGALG00010011343	0.957116974	0.330323673
ENSGALG00010011345	0.398154902	0.043415756
ENSGALG00010011347	0.568263145	0.234085579
ENSGALG00010011348	0.803447815	0.461212062
ENSGALG00010011349	0.963576725	0.339411061
ENSGALG00010011351	0.976004636	0.251393794
ENSGALG00010011352	0.516863243	0.470714279
ENSGALG00010011353	0.468655384	0.159156276
ENSGALG00010011354	0.2609579	0.121460333
ENSGALG00010011355	0.393293574	0.180300648

ENSGALG00010011357	0.61917455	0.162555312
ENSGALG00010011359	0.797049584	0.324862954
ENSGALG00010011360	0.970779089	0.356848953
ENSGALG00010011361	0.895237184	0.23033387
ENSGALG00010011362	0.284638036	0.409296373
ENSGALG00010011363	0.290760043	0.143635487
ENSGALG00010011364	0.002742065	0.085392592
ENSGALG00010011365	0.854716394	0.293019413
ENSGALG00010011366	0.188173046	0.085789746
ENSGALG00010011367	0.706010112	0.235524277
ENSGALG00010011368	0.766842049	0.413512398
ENSGALG00010011370	0.754733248	0.26417962
ENSGALG00010011371	0.627743991	0.438944433
ENSGALG00010011372	0.405243404	0.56256372
ENSGALG00010011374	0.889896006	0.313704127
ENSGALG00010011375	0.935031411	0.244845602
ENSGALG00010011376	0.302561663	0.280513489
ENSGALG00010011377	0.967615985	0.313203645
ENSGALG00010011378	0.696277196	0.263628347
ENSGALG00010011379	0.53536342	0.168858382
ENSGALG00010011380	0.495439405	0.190109089
ENSGALG00010011381	0.533800056	0.212344766
ENSGALG00010011382	0.133876829	0.302372657
ENSGALG00010011383	0.862011997	0.322690071
ENSGALG00010011384	0.026237502	0.326247416
ENSGALG00010011385	0.248800348	0.340410378
ENSGALG00010011386	0.927931125	0.264113577
ENSGALG00010011387	0.6720406	0.164584777
ENSGALG00010011388	0.489972394	0.103067123
ENSGALG00010011389	0.528188102	0.173938649
ENSGALG00010011390	0.505826993	0.281626193
ENSGALG00010011391	0.180447557	0.247826354
ENSGALG00010011392	0.970097372	0.307465939
ENSGALG00010011394	0.991950945	0.374175533
ENSGALG00010011395	0.026681103	0.120260695
ENSGALG00010011396	0.02903322	0.143948599
ENSGALG00010011398	0.602363793	0.154391241
ENSGALG00010011399	0.806161077	0.366077809
ENSGALG00010011400	0.977574227	0.403479292
ENSGALG00010011401	0.605793013	0.141949771
ENSGALG00010011403	0.365421542	0.060491169
ENSGALG00010011406	0.981694795	0.325299246
ENSGALG00010011407	0.737167105	0.30749975
ENSGALG00010011410	0.098374077	0.086174569
ENSGALG00010011411	0.062572455	0.372325902
ENSGALG00010011412	0.717582607	0.484979213
ENSGALG00010011413	0.455617277	0.202988794
ENSGALG00010011414	0.212489801	0.044241246
ENSGALG00010011415	0.264239718	0.073427965
ENSGALG00010011416	0.238275933	0.304119854
ENSGALG00010011417	0.881641227	0.266689249
ENSGALG00010011418	0.938985144	0.401802042
ENSGALG00010011419	0.910391148	0.27669541
ENSGALG00010011420	0.532986199	0.018525502
ENSGALG00010011421	0.787900642	0.36380624
ENSGALG00010011422	0.278329322	0.179272119

ENSGALG00010011423	0.518479945	0.217771866
ENSGALG00010011424	0.937000683	0.249455383
ENSGALG00010011425	0.913794677	0.223919023
ENSGALG00010011426	0.366309794	0.065469425
ENSGALG00010011427	0.665376654	0.185626694
ENSGALG00010011428	0.855421164	0.333158994
ENSGALG00010011429	0.954314467	0.335860546
ENSGALG00010011430	0.02372619	0.01699834
ENSGALG00010011431	0.96371613	0.329202155
ENSGALG00010011432	0.254396306	0.393023371
ENSGALG00010011433	0.666652606	0.325341182
ENSGALG00010011434	0.972113074	0.400130837
ENSGALG00010011436	0.002137418	0.308333474
ENSGALG00010011437	0.414225449	0.170909261
ENSGALG00010011438	0.803556069	0.353195011
ENSGALG00010011439	0.292751436	0.222813632
ENSGALG00010011440	0.983405558	0.353712756
ENSGALG00010011441	0.953605026	0.351447811
ENSGALG00010011443	0.055972588	0.210561539
ENSGALG00010011444	0.208550923	0.676301294
ENSGALG00010011445	0.700072203	0.080657532
ENSGALG00010011446	0.222329967	0.148016346
ENSGALG00010011447	0.852246332	0.516831648
ENSGALG00010011448	0.995229035	0.333780318
ENSGALG00010011449	0.990628554	0.320354941
ENSGALG00010011450	0.948050842	0.378426828
ENSGALG00010011452	0.841795203	0.165952907
ENSGALG00010011453	0.863109897	0.287067338
ENSGALG00010011454	0.957420049	0.331376719
ENSGALG00010011455	0.995961471	0.335620201
ENSGALG00010011456	0.892822679	0.099138465
ENSGALG00010011457	0.623647925	0.338635305
ENSGALG00010011458	0.817847501	0.277402001
ENSGALG00010011459	0.266418373	0.070408048
ENSGALG00010011460	0.715974756	0.228179698
ENSGALG00010011461	0.951946241	0.39281477
ENSGALG00010011462	0.934714225	0.346610454
ENSGALG00010011463	0.938945082	0.305408938
ENSGALG00010011464	0.4702125	0.112418143
ENSGALG00010011465	0.855145738	0.388854297
ENSGALG00010011466	0.870159566	0.213001879
ENSGALG00010011468	0.658709788	0.340967446
ENSGALG00010011469	0.519537205	0.21164544
ENSGALG00010011470	0.076718666	0.300404048
ENSGALG00010011471	0.585406596	0.469027993
ENSGALG00010011473	0.747000364	0.219771237
ENSGALG00010011474	0.575833754	0.231040825
ENSGALG00010011475	0.363651178	0.082143312
ENSGALG00010011477	0.977915062	0.312666893
ENSGALG00010011478	0.62446273	0.41799487
ENSGALG00010011480	0.72996601	0.242562651
ENSGALG00010011481	0.04665175	0.059060748
ENSGALG00010011482	0.975103457	0.426537516
ENSGALG00010011484	0.821566056	0.374089855
ENSGALG00010011485	0.872853022	0.152588525
ENSGALG00010011486	0.342424858	0.319091317

ENSGALG00010011487	0.94803608	0.476475409
ENSGALG00010011491	0.264239718	0.073427965
ENSGALG00010011494	0.39155441	0.07460765
ENSGALG00010011496	0.745122609	0.517239823
ENSGALG00010011497	0.867602233	0.067982848
ENSGALG00010011498	0.812203624	0.124746846
ENSGALG00010011499	0.836536682	0.311889049
ENSGALG00010011500	0.154454077	0.244544221
ENSGALG00010011501	0.503967172	0.055172762
ENSGALG00010011502	0.729633653	0.234589189
ENSGALG00010011503	0.916521836	0.438682822
ENSGALG00010011504	0.667227149	0.482843876
ENSGALG00010011505	0.982118831	0.329000487
ENSGALG00010011507	0.027569167	0.176120446
ENSGALG00010011508	0.39155441	0.07460765
ENSGALG00010011509	0.078237959	0.360530349
ENSGALG00010011511	0.955008608	0.345273669
ENSGALG00010011514	0.39144912	0.115895902
ENSGALG00010011515	0.917782477	0.316880719
ENSGALG00010011520	0.749193947	0.254850138
ENSGALG00010011522	0.264747167	0.042883696
ENSGALG00010011523	0.560861721	0.041618508
ENSGALG00010011525	0.742762347	0.346398279
ENSGALG00010011526	0.584104383	0.219706795
ENSGALG00010011529	0.805602287	0.331605615
ENSGALG00010011530	0.970955743	0.230957624
ENSGALG00010011531	0.950083128	0.23009068
ENSGALG00010011533	0.854749397	0.083860235
ENSGALG00010011536	0.935727299	0.27357892
ENSGALG00010011538	0.718789258	0.077641061
ENSGALG00010011539	0.949247165	0.331968404
ENSGALG00010011544	0.93277678	0.275874598
ENSGALG00010011545	0.97475756	0.377679897
ENSGALG00010011546	0.661730417	0.045885929
ENSGALG00010011548	0.735187976	0.323119759
ENSGALG00010011549	0.803181436	0.374633305
ENSGALG00010011551	0.963787487	0.313881511
ENSGALG00010011552	0.367278486	0.580935072
ENSGALG00010011553	0.692826429	0.282077751
ENSGALG00010011554	0.736769851	0.189034053
ENSGALG00010011556	0.231333391	0.151628886
ENSGALG00010011558	0.96997016	0.248447038
ENSGALG00010011559	0.938163341	0.417696586
ENSGALG00010011560	0.875816189	0.403950133
ENSGALG00010011561	0.906376964	0.240094544
ENSGALG00010011563	0.954654216	0.307824037
ENSGALG00010011564	0.657364544	0.417821904
ENSGALG00010011565	0.958221882	0.308707203
ENSGALG00010011566	0.994823087	0.339407562
ENSGALG00010011567	0.745447002	0.299116844
ENSGALG00010011568	0.230134203	0.172217715
ENSGALG00010011570	0.887025618	0.081031623
ENSGALG00010011571	0.773352238	0.122553173
ENSGALG00010011572	0.945286098	0.330600157
ENSGALG00010011573	0.288021143	0.332904484
ENSGALG00010011574	0.709754066	0.030166308

ENSGALG00010011575	0.359043838	0.185207052
ENSGALG00010011576	0.177586498	0.09313515
ENSGALG00010011578	0.972748762	0.33562868
ENSGALG00010011579	0.539238239	0.058714089
ENSGALG00010011580	0.153179575	0.186651527
ENSGALG00010011581	0.004425675	0.43769974
ENSGALG00010011582	0.912903905	0.419624694
ENSGALG00010011586	0.736624659	0.007141803
ENSGALG00010011587	0.740796892	0.365872899
ENSGALG00010011588	0.898399519	0.182054992
ENSGALG00010011589	0.283858919	0.533375093
ENSGALG00010011590	0.581925546	0.005661406
ENSGALG00010011592	0.820105071	0.281256104
ENSGALG00010011593	0.922903004	0.2200487
ENSGALG00010011594	0.085654925	0.205522587
ENSGALG00010011595	0.075107975	0.094783114
ENSGALG00010011596	0.325327998	0.037399662
ENSGALG00010011597	0.995184444	0.336952506
ENSGALG00010011598	0.386764803	0.105517621
ENSGALG00010011601	0.51666588	0.034977854
ENSGALG00010011603	0.480552967	0.204744011
ENSGALG00010011605	0.985101689	0.343622058
ENSGALG00010011607	0.944075895	0.38308238
ENSGALG00010011609	0.985195857	0.330501614
ENSGALG00010011612	0.515530475	0.121206583
ENSGALG00010011613	0.285272007	0.109169955
ENSGALG00010011615	0.3619239	0.131870846
ENSGALG00010011616	0.784503462	0.362232699
ENSGALG00010011617	0.372332139	0.051846001
ENSGALG00010011619	0.974307243	0.319170192
ENSGALG00010011620	0.044919115	0.152705647
ENSGALG00010011621	0.845837819	0.167546509
ENSGALG00010011622	0.953160088	0.346765414
ENSGALG00010011625	0.975214302	0.337895991
ENSGALG00010011626	0.90762228	0.492018246
ENSGALG00010011627	0.401639624	0.101384476
ENSGALG00010011628	0.952642187	0.456350232
ENSGALG00010011629	0.767684733	0.231319694
ENSGALG00010011630	0.501545021	0.148613615
ENSGALG00010011633	0.475692575	0.108802559
ENSGALG00010011634	0.1484139	0.033754126
ENSGALG00010011635	0.560424703	0.142578772
ENSGALG00010011636	0.960401906	0.255353807
ENSGALG00010011637	0.882158467	0.239675088
ENSGALG00010011638	0.313656284	0.17886592
ENSGALG00010011639	0.368476583	0.099553507
ENSGALG00010011640	0.375927362	0.072713138
ENSGALG00010011641	0.128105416	0.123478558
ENSGALG00010011643	0.989387432	0.287617609
ENSGALG00010011644	0.367689286	0.134680195
ENSGALG00010011646	0.264239718	0.073427965
ENSGALG00010011647	0.103206346	0.309872442
ENSGALG00010011648	0.925024779	0.333894983
ENSGALG00010011649	0.54468247	0.063484501
ENSGALG00010011650	0.913329691	0.288362466
ENSGALG00010011651	0.151164128	0.165318892

ENSGALG00010011652	0.862039205	0.289394475
ENSGALG00010011653	0.906980664	0.387029802
ENSGALG00010011655	0.56644393	0.254312451
ENSGALG00010011657	0.980429735	0.27375894
ENSGALG00010011658	0.264082519	0.307815413
ENSGALG00010011659	0.849397349	0.147504273
ENSGALG00010011660	0.89947965	0.155295863
ENSGALG00010011661	0.828608245	0.285084371
ENSGALG00010011662	0.98336613	0.299413741
ENSGALG00010011663	0.376087585	0.210661075
ENSGALG00010011664	0.97718249	0.357300684
ENSGALG00010011667	0.264239718	0.073427965
ENSGALG00010011668	0.994583102	0.338966243
ENSGALG00010011669	0.302724629	0.96934886
ENSGALG00010011670	0.891165426	0.399865049
ENSGALG00010011671	0.982846522	0.287261285
ENSGALG00010011673	0.809115913	0.357220904
ENSGALG00010011674	0.867861056	0.448701207
ENSGALG00010011677	0.940155955	0.20773105
ENSGALG00010011680	0.448858621	0.208430775
ENSGALG00010011681	0.692782425	0.308070692
ENSGALG00010011682	0.953991271	0.195759296
ENSGALG00010011683	0.423373618	0.185270871
ENSGALG00010011685	0.964680589	0.323338585
ENSGALG00010011686	0.38480368	0.180523942
ENSGALG00010011688	0.375672498	0.060345555
ENSGALG00010011689	0.819581612	0.075713902
ENSGALG00010011691	0.399949686	0.216676978
ENSGALG00010011692	0.960666387	0.293543243
ENSGALG00010011695	0.223609937	0.312393154
ENSGALG00010011696	0.958260537	0.383033183
ENSGALG00010011699	0.911686283	0.138142869
ENSGALG00010011701	0.561015022	0.566552327
ENSGALG00010011702	0.318335977	0.238374845
ENSGALG00010011703	0.909201511	0.231432473
ENSGALG00010011705	0.451833186	0.212831663
ENSGALG00010011706	0.609259914	0.178414241
ENSGALG00010011707	0.765645754	0.302647548
ENSGALG00010011708	0.940543353	0.357770605
ENSGALG00010011710	0.867398957	0.531284217
ENSGALG00010011712	0.571504144	0.426932381
ENSGALG00010011713	0.601149828	0.278271277
ENSGALG00010011714	0.483410608	0.182168852
ENSGALG00010011715	0.997075595	0.337640176
ENSGALG00010011716	0.654167806	0.193457175
ENSGALG00010011720	0.687949682	0.208949982
ENSGALG00010011722	0.648679614	0.049441986
ENSGALG00010011723	0.681242389	0.354803105
ENSGALG00010011724	0.869022311	0.192744453
ENSGALG00010011726	0.531598818	0.129803326
ENSGALG00010011729	0.499690341	0.416471465
ENSGALG00010011734	0.618088514	0.186097831
ENSGALG00010011735	0.394320708	0.213793726
ENSGALG00010011738	0.778162611	0.373658368
ENSGALG00010011739	0.588746884	0.101505993
ENSGALG00010011740	0.603848307	0.440375365

ENSGALG00010011741	0.443859172	0.063996169
ENSGALG00010011742	0.876603303	0.242752787
ENSGALG00010011743	0.483711062	0.189371251
ENSGALG00010011744	0.57937556	0.15826361
ENSGALG00010011745	0.290159775	0.107663777
ENSGALG00010011746	0.887502068	0.339804595
ENSGALG00010011747	0.863204776	0.27649403
ENSGALG00010011748	0.971556108	0.312547108
ENSGALG00010011749	0.15230793	0.205138772
ENSGALG00010011750	0.474924759	0.108796887
ENSGALG00010011751	0.944057625	0.178526242
ENSGALG00010011752	0.099392904	0.353257386
ENSGALG00010011753	0.796249424	0.286732751
ENSGALG00010011754	0.552034619	0.191219759
ENSGALG00010011755	0.486638466	0.067679636
ENSGALG00010011757	0.472800605	0.103933459
ENSGALG00010011758	0.911200277	0.477828338
ENSGALG00010011759	0.461722673	0.0511847
ENSGALG00010011760	0.270406625	0.256214966
ENSGALG00010011764	0.674403459	0.224933011
ENSGALG00010011765	0.49558281	0.163180225
ENSGALG00010011766	0.064062843	0.117847258
ENSGALG00010011767	0.949230122	0.350487534
ENSGALG00010011770	0.928644633	0.227810661
ENSGALG00010011771	0.989420402	0.379561383
ENSGALG00010011772	0.737251832	0.336799036
ENSGALG00010011774	0.361335709	0.133815935
ENSGALG00010011775	0.503013931	0.25010058
ENSGALG00010011776	0.93437749	0.214419472
ENSGALG00010011777	0.837837169	0.293638615
ENSGALG00010011780	0.429951074	0.306496661
ENSGALG00010011782	0.034060073	0.094660884
ENSGALG00010011784	0.937342753	0.338718157
ENSGALG00010011786	0.678963135	0.253454805
ENSGALG00010011787	0.945037812	0.347951272
ENSGALG00010011789	0.940464395	0.197282516
ENSGALG00010011790	0.429490046	0.354890265
ENSGALG00010011792	0.898102192	0.338904605
ENSGALG00010011793	0.174561363	0.237002427
ENSGALG00010011795	0.849185773	0.100916082
ENSGALG00010011796	0.990741779	0.319426444
ENSGALG00010011797	0.07938051	0.016256037
ENSGALG00010011798	0.867683171	0.214129973
ENSGALG00010011802	0.064495596	0.127968052
ENSGALG00010011803	0.452417225	0.631318418
ENSGALG00010011808	0.918197304	0.239908201
ENSGALG00010011810	0.425884608	0.07018661
ENSGALG00010011814	0.770786772	0.427895146
ENSGALG00010011816	0.95468339	0.226694064
ENSGALG00010011817	0.659371996	0.217381088
ENSGALG00010011819	0.854327964	0.384400771
ENSGALG00010011821	0.383041837	0.148092965
ENSGALG00010011823	0.094263426	0.26522695
ENSGALG00010011825	0.755904978	0.011627432
ENSGALG00010011828	0.245700908	0.010899108
ENSGALG00010011830	0.332738249	0.166829217

ENSGALG00010011833	0.258407206	0.372577979
ENSGALG00010011834	0.266137114	0.107784983
ENSGALG00010011836	0.329463224	0.169694316
ENSGALG00010011837	0.904634287	0.29891858
ENSGALG00010011839	0.960161889	0.28633474
ENSGALG00010011840	0.580850905	0.270864958
ENSGALG00010011841	0.669848214	0.163837991
ENSGALG00010011843	0.760267004	0.42423808
ENSGALG00010011846	0.957826504	0.326297962
ENSGALG00010011850	0.334544584	0.022218866
ENSGALG00010011851	0.578839053	0.057038895
ENSGALG00010011853	0.986864288	0.353479068
ENSGALG00010011855	0.565170855	0.269738805
ENSGALG00010011856	0.499512398	0.099639666
ENSGALG00010011859	0.831402908	0.425401911
ENSGALG00010011860	0.706053538	0.243684049
ENSGALG00010011861	0.989585521	0.334267443
ENSGALG00010011864	0.884033578	0.373363226
ENSGALG00010011866	0.975796372	0.32112176
ENSGALG00010011867	0.17174986	0.08911126
ENSGALG00010011868	0.792445005	0.231908087
ENSGALG00010011870	0.736283991	0.209448625
ENSGALG00010011871	0.618237815	0.19579431
ENSGALG00010011872	0.57014937	0.217917445
ENSGALG00010011873	0.816559963	0.225059368
ENSGALG00010011874	0.291723759	0.342962866
ENSGALG00010011875	0.740570057	0.226810127
ENSGALG00010011876	0.987907908	0.321303494
ENSGALG00010011879	0.99319153	0.357715374
ENSGALG00010011880	0.708196373	0.035926503
ENSGALG00010011881	0.68335939	0.420511293
ENSGALG00010011883	0.074112911	0.220367631
ENSGALG00010011884	0.52969404	0.146627906
ENSGALG00010011885	0.961915024	0.167982694
ENSGALG00010011886	0.889742885	0.481910104
ENSGALG00010011887	0.986801942	0.309351941
ENSGALG00010011888	0.97657261	0.295322631
ENSGALG00010011889	0.007203266	0.48972541
ENSGALG00010011890	0.666271935	0.192961624
ENSGALG00010011891	0.989092815	0.270783935
ENSGALG00010011893	0.881251278	0.329922449
ENSGALG00010011894	0.174800004	0.038959758
ENSGALG00010011895	0.091627886	0.220538455
ENSGALG00010011896	0.695347517	0.3167596
ENSGALG00010011898	0.980427911	0.319931597
ENSGALG00010011899	0.982537146	0.277652721
ENSGALG00010011901	0.262944547	0.194104897
ENSGALG00010011902	0.833842246	0.400876325
ENSGALG00010011903	0.918503866	0.31517555
ENSGALG00010011904	0.341252174	0.242639671
ENSGALG00010011905	0.455389738	0.15089549
ENSGALG00010011907	0.82887286	0.131152285
ENSGALG00010011908	0.620862875	0.283114556
ENSGALG00010011909	0.327705463	0.244891426
ENSGALG00010011911	0.656720654	0.620875816
ENSGALG00010011912	0.525251759	0.278129137

ENSGALG00010011913	0.89456888	0.14488699
ENSGALG00010011914	0.2878541	0.085532585
ENSGALG00010011915	0.933732401	0.278660177
ENSGALG00010011916	0.442190906	0.358404691
ENSGALG00010011917	0.915999119	0.231855288
ENSGALG00010011918	0.31274393	0.056873263
ENSGALG00010011920	0.266137114	0.107784983
ENSGALG00010011921	0.993736945	0.344316825
ENSGALG00010011922	0.619537866	0.369467628
ENSGALG00010011923	0.727093087	0.301508952
ENSGALG00010011924	0.99377825	0.338508975
ENSGALG00010011925	0.588072892	0.357279496
ENSGALG00010011926	0.971441913	0.301947673
ENSGALG00010011927	0.913839681	0.375649536
ENSGALG00010011929	0.713631056	0.013620675
ENSGALG00010011930	0.855102839	0.141705898
ENSGALG00010011931	0.324470909	0.107018782
ENSGALG00010011932	0.981360163	0.381285972
ENSGALG00010011933	0.930521308	0.309319476
ENSGALG00010011934	0.247217504	0.210085781
ENSGALG00010011935	0.939921489	0.238762361
ENSGALG00010011936	0.611794619	0.019144574
ENSGALG00010011937	0.90573596	0.141259132
ENSGALG00010011938	0.170568071	0.886028531
ENSGALG00010011939	0.637892194	0.195401662
ENSGALG00010011941	0.945016225	0.23524561
ENSGALG00010011943	0.610363861	0.260221111
ENSGALG00010011944	0.950950936	0.137173984
ENSGALG00010011946	0.013905876	0.50828939
ENSGALG00010011947	0.954877159	0.285806587
ENSGALG00010011949	0.977628021	0.312237441
ENSGALG00010011950	0.985941029	0.334834001
ENSGALG00010011952	0.964674673	0.262786103
ENSGALG00010011953	0.42320212	0.045574073
ENSGALG00010011954	0.397287082	0.408721044
ENSGALG00010011957	0.9835734	0.309379608
ENSGALG00010011960	0.326428945	0.159254363
ENSGALG00010011961	0.950096011	0.383201963
ENSGALG00010011962	0.651432162	0.528785039
ENSGALG00010011963	0.931098837	0.241747607
ENSGALG00010011965	0.978199116	0.312508673
ENSGALG00010011966	0.593884663	0.049358268
ENSGALG00010011967	0.975570719	0.307075316
ENSGALG00010011968	0.934560417	0.315794939
ENSGALG00010011969	0.31380035	0.209059525
ENSGALG00010011971	0.46662339	0.0118048
ENSGALG00010011972	0.845426412	0.383640891
ENSGALG00010011973	0.95174429	0.453424844
ENSGALG00010011976	0.934472544	0.286581101
ENSGALG00010011977	0.949552501	0.250159871
ENSGALG00010011979	0.949173268	0.287006315
ENSGALG00010011980	0.995159797	0.335262489
ENSGALG00010011981	0.503351778	0.057829877
ENSGALG00010011982	0.95724726	0.207335472
ENSGALG00010011983	0.815281931	0.231449266
ENSGALG00010011984	0.914563452	0.189523645

ENSGALG00010011985	0.904456307	0.399371993
ENSGALG00010011987	0.923077087	0.207368346
ENSGALG00010011989	0.950598452	0.334017361
ENSGALG00010011991	0.736667596	0.060889772
ENSGALG00010011992	0.18883989	0.179765984
ENSGALG00010011993	0.960083837	0.288483659
ENSGALG00010011994	0.993373096	0.3355929
ENSGALG00010011996	0.890319642	0.239736064
ENSGALG00010011997	0.968362448	0.303690476
ENSGALG00010011998	0.850748023	0.293871339
ENSGALG00010012000	0.188292245	0.218406976
ENSGALG00010012001	0.255147627	0.000222436
ENSGALG00010012004	0.691352587	0.491785273
ENSGALG00010012007	0.20322517	0.162383673
ENSGALG00010012008	0.781506443	0.191133714
ENSGALG00010012010	0.517133108	0.019748633
ENSGALG00010012011	0.966339599	0.307510396
ENSGALG00010012012	0.277566769	0.107857127
ENSGALG00010012013	0.187514554	0.108479243
ENSGALG00010012015	0.319595365	0.129731762
ENSGALG00010012016	0.890787488	0.322206434
ENSGALG00010012017	0.796112282	0.309359231
ENSGALG00010012018	0.722336407	0.263574938
ENSGALG00010012019	0.983119706	0.322213935
ENSGALG00010012021	0.146592976	0.139930559
ENSGALG00010012022	0.244156167	0.153802059
ENSGALG00010012023	0.109443317	0.180132757
ENSGALG00010012024	0.459443939	0.34820086
ENSGALG00010012025	0.345248143	0.071509017
ENSGALG00010012026	0.112680991	0.204969795
ENSGALG00010012027	0.621562527	0.266502984
ENSGALG00010012028	0.510563674	0.122437857
ENSGALG00010012031	0.923203342	0.151832843
ENSGALG00010012032	0.971730812	0.290713268
ENSGALG00010012034	0.454965387	0.380086403
ENSGALG00010012035	0.061265043	0.137399214
ENSGALG00010012039	0.277566769	0.107857127
ENSGALG00010012040	0.721868544	0.214148708
ENSGALG00010012041	0.943586723	0.356991404
ENSGALG00010012042	0.839153021	0.246049954
ENSGALG00010012043	0.925644013	0.307923856
ENSGALG00010012044	0.584253952	0.232013386
ENSGALG00010012045	0.779722243	0.300717674
ENSGALG00010012046	0.813114291	0.376133263
ENSGALG00010012047	0.900435439	0.252548293
ENSGALG00010012048	0.431650221	0.394914953
ENSGALG00010012049	0.982863374	0.285703693
ENSGALG00010012051	0.273602451	0.568861216
ENSGALG00010012052	0.888799149	0.227788264
ENSGALG00010012053	0.687600894	0.048071332
ENSGALG00010012054	0.841388017	0.209259085
ENSGALG00010012055	0.821285067	0.188500103
ENSGALG00010012056	0.779676548	0.226805181
ENSGALG00010012058	0.372266996	0.37524055
ENSGALG00010012059	0.448112668	0.024750603
ENSGALG00010012060	0.987176451	0.297577412

ENSGALG00010012061	0.420759023	0.11967094
ENSGALG00010012062	0.030894139	0.178830973
ENSGALG00010012063	0.801100814	0.356654301
ENSGALG00010012064	0.770559589	0.06803018
ENSGALG00010012065	0.388181886	0.168275567
ENSGALG00010012066	0.991999008	0.370590736
ENSGALG00010012067	0.068506004	0.161511152
ENSGALG00010012068	0.05197934	0.14495833
ENSGALG00010012069	0.34081212	0.34730063
ENSGALG00010012070	0.201115968	0.31178457
ENSGALG00010012071	0.88689419	0.360104101
ENSGALG00010012072	0.954775546	0.418169704
ENSGALG00010012073	0.378770564	0.079283839
ENSGALG00010012074	0.935296588	0.249497986
ENSGALG00010012075	0.922682791	0.224820846
ENSGALG00010012076	0.325119108	0.20125316
ENSGALG00010012077	0.49605383	0.278749403
ENSGALG00010012078	0.804971557	0.278791065
ENSGALG00010012079	0.208116023	0.197338301
ENSGALG00010012080	0.163503711	0.101124089
ENSGALG00010012081	0.96115913	0.362722216
ENSGALG00010012082	0.623811183	0.421344119
ENSGALG00010012083	0.880000157	0.470814313
ENSGALG00010012084	0.969626111	0.266558966
ENSGALG00010012085	0.844192549	0.164257773
ENSGALG00010012086	0.434480888	0.64786167
ENSGALG00010012087	0.841677995	0.312116955
ENSGALG00010012088	0.737477467	0.367641895
ENSGALG00010012089	0.846798434	0.232122906
ENSGALG00010012090	0.936448028	0.356960054
ENSGALG00010012091	0.832135822	0.239102661
ENSGALG00010012092	0.049100097	0.306127958
ENSGALG00010012093	0.603564165	0.346718638
ENSGALG00010012094	0.290206454	0.07030665
ENSGALG00010012095	0.74555247	0.245732387
ENSGALG00010012096	0.936907578	0.223852324
ENSGALG00010012097	0.972205171	0.28617498
ENSGALG00010012098	0.144950617	0.223270414
ENSGALG00010012099	0.271944098	0.328468861
ENSGALG00010012100	0.985982323	0.32196462
ENSGALG00010012101	0.879458645	0.255637781
ENSGALG00010012102	0.096479429	0.317155548
ENSGALG00010012103	0.980024551	0.345109188
ENSGALG00010012105	0.668928366	0.435759193
ENSGALG00010012106	0.257084704	0.076724426
ENSGALG00010012107	0.608833794	0.490123655
ENSGALG00010012108	0.952914751	0.4059308
ENSGALG00010012110	0.989471616	0.37963336
ENSGALG00010012111	0.964595258	0.425906034
ENSGALG00010012112	0.951611737	0.31250283
ENSGALG00010012113	0.086151952	0.024534691
ENSGALG00010012114	0.98684992	0.386135468
ENSGALG00010012115	0.158787776	0.092763717
ENSGALG00010012116	0.967621033	0.19207874
ENSGALG00010012118	0.422309209	0.141811214
ENSGALG00010012119	0.940962094	0.410630933

ENSGALG00010012120	0.98699645	0.312132157
ENSGALG00010012121	0.848854534	0.225993065
ENSGALG00010012122	0.884259416	0.379169606
ENSGALG00010012123	0.729917612	0.314485819
ENSGALG00010012124	0.911540131	0.288374391
ENSGALG00010012125	0.769731312	0.113090665
ENSGALG00010012127	0.755922822	0.290125774
ENSGALG00010012128	0.491426386	0.646990655
ENSGALG00010012129	0.347153445	0.151918687
ENSGALG00010012130	0.109407067	0.065446484
ENSGALG00010012131	0.92979051	0.258177122
ENSGALG00010012133	0.77720716	0.136755704
ENSGALG00010012135	0.939155853	0.351956634
ENSGALG00010012136	0.599546708	0.02001782
ENSGALG00010012138	0.87362878	0.578334227
ENSGALG00010012140	0.714991007	0.313237339
ENSGALG00010012141	0.410323842	0.138736863
ENSGALG00010012142	0.683419221	0.292406822
ENSGALG00010012143	0.17356806	0.179677741
ENSGALG00010012144	0.910784534	0.278219283
ENSGALG00010012145	0.86355224	0.319480195
ENSGALG00010012147	0.973691443	0.340953937
ENSGALG00010012148	0.436051612	0.144836033
ENSGALG00010012149	0.929026616	0.328454134
ENSGALG00010012150	0.28986868	0.272918563
ENSGALG00010012151	0.500493317	0.166899124
ENSGALG00010012153	0.33777847	0.133444889
ENSGALG00010012154	0.574186831	0.415897645
ENSGALG00010012155	0.075650671	0.188849599
ENSGALG00010012156	0.583610171	0.185008823
ENSGALG00010012157	0.811736126	0.150739253
ENSGALG00010012158	0.034060073	0.094660884
ENSGALG00010012159	0.321038788	0.006470839
ENSGALG00010012160	0.36131855	0.079621968
ENSGALG00010012161	0.77344179	0.10121447
ENSGALG00010012162	0.267018706	0.028417775
ENSGALG00010012163	0.949925036	0.295542423
ENSGALG00010012164	0.264239718	0.073427965
ENSGALG00010012165	0.085128486	0.232424904
ENSGALG00010012166	0.715204142	0.286890639
ENSGALG00010012167	0.749773862	0.238289763
ENSGALG00010012168	0.368276332	0.248136926
ENSGALG00010012169	0.71144714	0.298558914
ENSGALG00010012171	0.996191249	0.33589749
ENSGALG00010012172	0.669260657	0.295007515
ENSGALG00010012173	0.014833316	0.547907885
ENSGALG00010012174	0.201512115	0.188537209
ENSGALG00010012175	0.988710067	0.344058402
ENSGALG00010012176	0.219386918	0.079388121
ENSGALG00010012177	0.755804957	0.336659918
ENSGALG00010012179	0.869207028	0.179215259
ENSGALG00010012180	0.656602307	0.031124264
ENSGALG00010012181	0.324020854	0.340314887
ENSGALG00010012182	0.870073325	0.368418056
ENSGALG00010012184	0.262922434	0.138601464
ENSGALG00010012186	0.959708321	0.290989836

ENSGALG00010012187	0.072443378	0.463646656
ENSGALG00010012188	0.700821337	0.200494281
ENSGALG00010012190	0.924087996	0.330572837
ENSGALG00010012191	0.98927153	0.309496276
ENSGALG00010012193	0.081766613	0.217982134
ENSGALG00010012194	0.564384142	0.171504076
ENSGALG00010012195	0.496926336	0.001311978
ENSGALG00010012196	0.104683574	0.172179805
ENSGALG00010012198	0.91843905	0.233977203
ENSGALG00010012199	0.290459155	0.052176187
ENSGALG00010012200	0.713575889	0.242621794
ENSGALG00010012202	0.119404885	0.095677849
ENSGALG00010012203	0.196562959	0.140074262
ENSGALG00010012205	0.797547215	0.256895358
ENSGALG00010012207	0.855576472	0.103765284
ENSGALG00010012208	0.716840183	0.348404724
ENSGALG00010012209	0.656127957	0.235073357
ENSGALG00010012210	0.255687341	0.370330683
ENSGALG00010012212	0.369285984	0.322826047
ENSGALG00010012213	0.754083844	0.502543717
ENSGALG00010012215	0.95680393	0.400379247
ENSGALG00010012216	0.120253183	0.187498856
ENSGALG00010012217	0.207114972	0.167559281
ENSGALG00010012218	0.012433676	0.134633327
ENSGALG00010012219	0.278069753	0.224084059
ENSGALG00010012224	0.376680122	0.171308365
ENSGALG00010012225	0.687195335	0.055826735
ENSGALG00010012226	0.509707519	0.329207768
ENSGALG00010012227	0.931918612	0.324371058
ENSGALG00010012228	0.599446594	0.11762545
ENSGALG00010012229	0.958633615	0.204993087
ENSGALG00010012230	0.494119398	0.071865626
ENSGALG00010012232	0.454790157	0.150866466
ENSGALG00010012233	0.264239718	0.073427965
ENSGALG00010012234	0.917590592	0.302878933
ENSGALG00010012235	0.907513172	0.282094238
ENSGALG00010012236	0.067733698	0.1621315
ENSGALG00010012237	0.669457956	0.182913933
ENSGALG00010012239	0.741467983	0.354508977
ENSGALG00010012240	0.889521265	0.322243263
ENSGALG00010012241	0.799115159	0.308690215
ENSGALG00010012242	0.073249016	0.149092301
ENSGALG00010012243	0.227300086	0.179854268
ENSGALG00010012244	0.957175565	0.269502689
ENSGALG00010012245	0.907804889	0.421788022
ENSGALG00010012246	0.617791301	0.292627085
ENSGALG00010012247	0.762173238	0.130812431
ENSGALG00010012248	0.903743359	0.360980448
ENSGALG00010012250	0.55835516	0.433183788
ENSGALG00010012252	0.456266076	0.096299349
ENSGALG00010012253	0.998232705	0.342775793
ENSGALG00010012258	0.50902694	0.470935475
ENSGALG00010012259	0.761603363	0.242021246
ENSGALG00010012260	0.534925549	0.072882227
ENSGALG00010012262	0.571775559	0.205677313
ENSGALG00010012263	0.737403879	0.040155689

ENSGALG00010012264	0.996433873	0.367514846
ENSGALG00010012266	0.463262327	0.091635488
ENSGALG00010012268	0.38089863	0.141066523
ENSGALG00010012271	0.690430351	0.283850997
ENSGALG00010012274	0.954091836	0.318785275
ENSGALG00010012275	0.797624537	0.523621078
ENSGALG00010012277	0.961918481	0.350411924
ENSGALG00010012282	0.318751865	0.188158991
ENSGALG00010012283	0.560044412	0.180483666
ENSGALG00010012285	0.977545297	0.259040801
ENSGALG00010012286	0.040734307	0.154016134
ENSGALG00010012288	0.98483695	0.314479719
ENSGALG00010012290	0.583257835	0.337519129
ENSGALG00010012291	0.981750062	0.223630472
ENSGALG00010012293	0.829965192	0.175506801
ENSGALG00010012294	0.009594157	0.270843457
ENSGALG00010012295	0.71344741	0.392629386
ENSGALG00010012297	0.863716215	0.322674661
ENSGALG00010012300	0.600866026	0.246190073
ENSGALG00010012303	0.938919015	0.267220006
ENSGALG00010012304	0.492536268	0.31590125
ENSGALG00010012307	0.928283601	0.259762964
ENSGALG00010012308	0.984910628	0.289410171
ENSGALG00010012309	0.932042276	0.363336613
ENSGALG00010012310	0.386940806	0.06873218
ENSGALG00010012311	0.989504321	0.275796974
ENSGALG00010012312	0.981446791	0.316299608
ENSGALG00010012314	0.496369668	0.341817516
ENSGALG00010012315	0.900098315	0.336324608
ENSGALG00010012317	0.227111494	0.478993975
ENSGALG00010012318	0.332220106	0.153703265
ENSGALG00010012320	0.923631494	0.155388383
ENSGALG00010012321	0.533598147	0.305978427
ENSGALG00010012322	0.954745728	0.221589221
ENSGALG00010012324	0.922309956	0.2753587
ENSGALG00010012325	0.973518758	0.238416592
ENSGALG00010012326	0.721461475	0.629639103
ENSGALG00010012327	0.593036553	0.148278906
ENSGALG00010012328	0.261809432	0.131901688
ENSGALG00010012329	0.842005794	0.370906455
ENSGALG00010012330	0.633564993	0.334007398
ENSGALG00010012331	0.864040535	0.326306194
ENSGALG00010012332	0.131918545	0.181841852
ENSGALG00010012334	0.811826751	0.18604391
ENSGALG00010012335	0.396365709	0.157137627
ENSGALG00010012337	0.982032159	0.363181737
ENSGALG00010012338	0.285027102	0.109308519
ENSGALG00010012339	0.95778692	0.201483129
ENSGALG00010012340	0.8194132	0.250772438
ENSGALG00010012341	0.786774319	0.51245832
ENSGALG00010012343	0.981151967	0.335326146
ENSGALG00010012344	0.445792306	0.32865935
ENSGALG00010012345	0.941577939	0.307717971
ENSGALG00010012346	0.910597868	0.360675962
ENSGALG00010012347	0.418197165	0.554176065
ENSGALG00010012348	0.058620363	0.471716419

ENSGALG00010012349	0.207523649	0.231661294
ENSGALG00010012350	0.909593537	0.27493151
ENSGALG00010012351	0.050427635	0.416779832
ENSGALG00010012352	0.974230883	0.311820961
ENSGALG00010012353	0.93377224	0.458906272
ENSGALG00010012354	0.757025665	0.129106577
ENSGALG00010012355	0.054289971	0.14989198
ENSGALG00010012356	0.636293872	0.271787957
ENSGALG00010012358	0.974529044	0.274091525
ENSGALG00010012360	0.894413549	0.145472276
ENSGALG00010012361	0.37532191	0.151592009
ENSGALG00010012362	0.941443896	0.405473476
ENSGALG00010012363	0.050115024	0.155140157
ENSGALG00010012364	0.708784202	0.306571811
ENSGALG00010012366	0.459976308	0.149302045
ENSGALG00010012367	0.967302054	0.238078739
ENSGALG00010012371	0.28803962	0.178648102
ENSGALG00010012372	0.091357271	0.008097583
ENSGALG00010012373	0.145804588	0.172052153
ENSGALG00010012374	0.1156088	0.565879625
ENSGALG00010012376	0.985108869	0.336097591
ENSGALG00010012377	0.152378064	0.375902785
ENSGALG00010012378	0.707581681	0.293466375
ENSGALG00010012382	0.981656642	0.302877008
ENSGALG00010012384	0.611102521	0.064932306
ENSGALG00010012385	0.571830253	0.224407096
ENSGALG00010012386	0.606850138	0.224565697
ENSGALG00010012387	0.90460781	0.361483177
ENSGALG00010012390	0.826267573	0.353913597
ENSGALG00010012391	0.923394045	0.2211967
ENSGALG00010012393	0.396668506	0.181671381
ENSGALG00010012394	0.304133466	0.450346192
ENSGALG00010012396	0.259919719	0.138129481
ENSGALG00010012398	0.071739017	0.123227448
ENSGALG00010012399	0.83433789	0.179111956
ENSGALG00010012401	0.837369367	0.169404126
ENSGALG00010012402	0.327556138	0.190619202
ENSGALG00010012404	0.885694347	0.270783967
ENSGALG00010012405	0.977455749	0.341258246
ENSGALG00010012406	0.995240169	0.34172099
ENSGALG00010012407	0.753757416	0.224533221
ENSGALG00010012408	0.498823559	0.228261749
ENSGALG00010012410	0.562414505	0.126282851
ENSGALG00010012412	0.349690836	0.193540413
ENSGALG00010012413	0.769546493	0.090789531
ENSGALG00010012414	0.99007082	0.314777542
ENSGALG00010012415	0.96839657	0.281924941
ENSGALG00010012416	0.184899013	0.310405855
ENSGALG00010012417	0.231139392	0.273128114
ENSGALG00010012418	0.487231014	0.183681527
ENSGALG00010012419	0.662050395	0.078680491
ENSGALG00010012420	0.645118714	0.147260876
ENSGALG00010012421	0.152833232	0.262330917
ENSGALG00010012423	0.303948957	0.198834601
ENSGALG00010012424	0.97646959	0.332828577
ENSGALG00010012425	0.425164887	0.172951222

ENSGALG00010012426	0.174563446	0.271427293
ENSGALG00010012427	0.935808208	0.215216328
ENSGALG00010012428	0.792193773	0.449381784
ENSGALG00010012429	0.811014252	0.4121805
ENSGALG00010012430	0.98385022	0.361496179
ENSGALG00010012431	0.574204409	0.334056756
ENSGALG00010012432	0.219386918	0.079388121
ENSGALG00010012433	0.336450291	0.253393944
ENSGALG00010012434	0.28826843	0.31329886
ENSGALG00010012435	0.201440626	0.168570114
ENSGALG00010012436	0.985533363	0.311238691
ENSGALG00010012438	0.160795421	0.445532741
ENSGALG00010012439	0.981614447	0.347223329
ENSGALG00010012440	0.763784697	0.239117837
ENSGALG00010012441	0.918476609	0.223956509
ENSGALG00010012442	0.490454122	0.282890368
ENSGALG00010012444	0.825053966	0.148927487
ENSGALG00010012445	0.987502646	0.325126312
ENSGALG00010012447	0.982199344	0.353876745
ENSGALG00010012448	0.271701261	0.072417625
ENSGALG00010012449	0.157606591	0.003198891
ENSGALG00010012450	0.923319574	0.320674382
ENSGALG00010012452	0.835014971	0.090886509
ENSGALG00010012454	0.789133545	0.291823727
ENSGALG00010012455	0.979295637	0.322715225
ENSGALG00010012456	0.256700942	0.029107591
ENSGALG00010012458	0.569213355	0.413228599
ENSGALG00010012459	0.039898928	0.722052861
ENSGALG00010012461	0.018104086	0.245325562
ENSGALG00010012463	0.0976492	0.374558104
ENSGALG00010012465	0.900518559	0.354712876
ENSGALG00010012466	0.951418686	0.401966527
ENSGALG00010012467	0.284702565	0.095129525
ENSGALG00010012468	0.044895337	0.187903904
ENSGALG00010012469	0.349430636	0.36405034
ENSGALG00010012470	0.182910978	0.260673537
ENSGALG00010012473	0.401704699	0.049652806
ENSGALG00010012474	0.452379072	0.184632839
ENSGALG00010012475	0.783296581	0.247292294
ENSGALG00010012476	0.207501166	0.223174188
ENSGALG00010012477	0.223187706	0.482797555
ENSGALG00010012478	0.627100959	0.454854599
ENSGALG00010012479	0.540436126	0.170669315
ENSGALG00010012480	0.926750888	0.449503943
ENSGALG00010012481	0.737748439	0.414034002
ENSGALG00010012482	0.147900396	0.135481317
ENSGALG00010012483	0.907415256	0.239804401
ENSGALG00010012484	0.258650604	0.15311761
ENSGALG00010012485	0.995054357	0.361456333
ENSGALG00010012486	0.307568048	0.311026784
ENSGALG00010012487	0.028771779	0.267362337
ENSGALG00010012490	0.366461586	0.435165612
ENSGALG00010012491	0.424053777	0.266333122
ENSGALG00010012492	0.891806398	0.500598385
ENSGALG00010012495	0.189240572	0.04996112
ENSGALG00010012496	0.743139776	0.355567883

ENSGALG00010012497	0.110503813	0.002612981
ENSGALG00010012498	0.968710579	0.488053318
ENSGALG00010012500	0.888112389	0.310832102
ENSGALG00010012501	0.480745159	0.185706431
ENSGALG00010012502	0.98644039	0.331556433
ENSGALG00010012503	0.845393165	0.267369649
ENSGALG00010012506	0.958930335	0.362595317
ENSGALG00010012507	0.10496232	0.284871669
ENSGALG00010012508	0.235532273	0.272683833
ENSGALG00010012509	0.934802528	0.2417222
ENSGALG00010012511	0.98139591	0.278234315
ENSGALG00010012515	0.779159997	0.251493389
ENSGALG00010012516	0.043079977	0.106820669
ENSGALG00010012517	0.876723213	0.261290763
ENSGALG00010012518	0.285272007	0.109169955
ENSGALG00010012519	0.016679716	0.125258416
ENSGALG00010012521	0.905091877	0.242040794
ENSGALG00010012522	0.096023914	0.145693557
ENSGALG00010012524	0.333055781	0.096253646
ENSGALG00010012526	0.102155	0.184542342
ENSGALG00010012528	0.440638897	0.196247805
ENSGALG00010012529	0.053111065	0.094060283
ENSGALG00010012530	0.923149155	0.299023111
ENSGALG00010012531	0.106706915	0.415508417
ENSGALG00010012532	0.978739864	0.310561329
ENSGALG00010012534	0.771293331	0.191823329
ENSGALG00010012535	0.816879584	0.191757171
ENSGALG00010012536	0.798749411	0.230799681
ENSGALG00010012538	0.914088602	0.251961309
ENSGALG00010012539	0.418335342	0.459088865
ENSGALG00010012541	0.484301047	0.333128977
ENSGALG00010012542	0.185560646	0.225434998
ENSGALG00010012543	0.143531397	0.220035291
ENSGALG00010012544	0.504678782	0.156838383
ENSGALG00010012545	0.436312113	0.104683013
ENSGALG00010012546	0.888142974	0.171358731
ENSGALG00010012547	0.494563401	0.059937476
ENSGALG00010012548	0.123221853	0.214803776
ENSGALG00010012550	0.920457036	0.317977553
ENSGALG00010012551	0.577283617	0.292634635
ENSGALG00010012552	0.528039363	0.002163392
ENSGALG00010012553	0.354417038	0.138796309
ENSGALG00010012555	0.894087157	0.197143339
ENSGALG00010012558	0.219386918	0.079388121
ENSGALG00010012561	0.850758163	0.089737487
ENSGALG00010012565	0.981065576	0.338952903
ENSGALG00010012569	0.969635858	0.287643891
ENSGALG00010012571	0.854796381	0.120363122
ENSGALG00010012573	0.377900487	0.132867801
ENSGALG00010012574	0.910281944	0.31206034
ENSGALG00010012575	0.089301885	0.168307621
ENSGALG00010012578	0.865189961	0.310834508
ENSGALG00010012581	0.265558089	0.028821141
ENSGALG00010012582	0.388067758	0.028544245
ENSGALG00010012583	0.86851384	0.449425037
ENSGALG00010012586	0.852724587	0.390174226

ENSGALG00010012587	0.93536289	0.19282432
ENSGALG00010012588	0.662958687	0.33842952
ENSGALG00010012593	0.279338442	0.135977135
ENSGALG00010012598	0.320167986	0.215348639
ENSGALG00010012599	0.939986021	0.290949926
ENSGALG00010012600	0.87913811	0.326023343
ENSGALG00010012601	0.953848335	0.300832653
ENSGALG00010012603	0.35306172	0.17929633
ENSGALG00010012606	0.533616741	0.359660376
ENSGALG00010012607	0.855594154	0.184372797
ENSGALG00010012609	0.33982727	0.065786191
ENSGALG00010012610	0.846923655	0.176340111
ENSGALG00010012614	0.390741705	0.105825194
ENSGALG00010012615	0.923837771	0.334933259
ENSGALG00010012616	0.954442436	0.347228457
ENSGALG00010012617	0.839693672	0.345336077
ENSGALG00010012618	0.592054958	0.077330337
ENSGALG00010012620	0.879690748	0.136711034
ENSGALG00010012624	0.856092572	0.35545908
ENSGALG00010012626	0.308758304	0.100827364
ENSGALG00010012627	0.478532683	0.276324085
ENSGALG00010012629	0.679938259	0.076307577
ENSGALG00010012630	0.691772697	0.265280889
ENSGALG00010012631	0.945439413	0.31158569
ENSGALG00010012632	0.180359236	0.196434749
ENSGALG00010012633	0.935259333	0.368220395
ENSGALG00010012634	0.892591301	0.335561263
ENSGALG00010012636	0.366176244	0.210393853
ENSGALG00010012637	0.256828349	0.119064955
ENSGALG00010012638	0.84655472	0.481527822
ENSGALG00010012639	0.077872215	0.13850113
ENSGALG00010012640	0.693970072	0.324606355
ENSGALG00010012648	0.467225506	0.037644195
ENSGALG00010012650	0.970034656	0.315875563
ENSGALG00010012651	0.320640563	0.165169259
ENSGALG00010012652	0.681666871	0.244221564
ENSGALG00010012653	0.33627712	0.221904457
ENSGALG00010012654	0.78902262	0.397924266
ENSGALG00010012655	0.39155441	0.07460765
ENSGALG00010012657	0.482258779	0.139453091
ENSGALG00010012658	0.369478107	0.160897087
ENSGALG00010012661	0.991034801	0.332083692
ENSGALG00010012662	0.967251236	0.207557067
ENSGALG00010012664	0.482218067	0.194074951
ENSGALG00010012665	0.954692102	0.303515451
ENSGALG00010012667	0.952349996	0.314853194
ENSGALG00010012669	0.931595467	0.194248009
ENSGALG00010012670	0.616467441	0.145209983
ENSGALG00010012671	0.918948978	0.298235527
ENSGALG00010012672	0.840755703	0.201415294
ENSGALG00010012673	0.784270131	0.11015394
ENSGALG00010012674	0.885978898	0.323641006
ENSGALG00010012675	0.428199258	0.187309964
ENSGALG00010012676	0.021423515	0.158182295
ENSGALG00010012677	0.907197965	0.367767083
ENSGALG00010012678	0.057360065	0.288973595

ENSGALG00010012680	0.131379375	0.266309757
ENSGALG00010012682	0.99061937	0.314919446
ENSGALG00010012683	0.253080239	0.071438614
ENSGALG00010012685	0.953581106	0.364167374
ENSGALG00010012687	0.895116529	0.426644725
ENSGALG00010012688	0.831551725	0.279505285
ENSGALG00010012690	0.972087552	0.3398272
ENSGALG00010012693	0.968293398	0.334332521
ENSGALG00010012694	0.687060737	0.105008607
ENSGALG00010012696	0.559941816	0.227309176
ENSGALG00010012697	0.71507851	0.415868115
ENSGALG00010012698	0.427728838	0.289863623
ENSGALG00010012699	0.964338203	0.369949645
ENSGALG00010012700	0.664802011	0.043279908
ENSGALG00010012701	0.827077973	0.51343655
ENSGALG00010012702	0.320093614	0.017032614
ENSGALG00010012704	0.487936173	0.069374768
ENSGALG00010012705	0.939684008	0.252055779
ENSGALG00010012706	0.748706375	0.418423461
ENSGALG00010012707	0.805256614	0.483978725
ENSGALG00010012708	0.989043066	0.378861101
ENSGALG00010012709	0.629620506	0.376962614
ENSGALG00010012710	0.760427351	0.274732079
ENSGALG00010012711	0.702566824	0.250353251
ENSGALG00010012712	0.924407065	0.270092708
ENSGALG00010012713	0.842351443	0.116818566
ENSGALG00010012714	0.61965133	0.333720481
ENSGALG00010012715	0.134457843	0.174600213
ENSGALG00010012717	0.696981676	0.288043
ENSGALG00010012718	0.006468973	0.227828661
ENSGALG00010012719	0.286700987	0.26294128
ENSGALG00010012720	0.9517374	0.287380978
ENSGALG00010012722	0.826700958	0.096699598
ENSGALG00010012723	0.200629457	0.015680852
ENSGALG00010012725	0.640587126	0.484704546
ENSGALG00010012726	0.955054757	0.235894375
ENSGALG00010012728	0.900440722	0.255363138
ENSGALG00010012730	0.995635201	0.334531297
ENSGALG00010012732	0.993927937	0.341635287
ENSGALG00010012734	0.980077645	0.443247542
ENSGALG00010012736	0.35371609	0.133957029
ENSGALG00010012738	0.824004493	0.191841019
ENSGALG00010012739	0.729129214	0.245361794
ENSGALG00010012741	0.634111492	0.121503578
ENSGALG00010012742	0.017725483	0.35627553
ENSGALG00010012743	0.913417352	0.498274914
ENSGALG00010012744	0.780666654	0.059672412
ENSGALG00010012745	0.380930144	0.086707133
ENSGALG00010012747	0.495134485	0.15931316
ENSGALG00010012749	0.947524198	0.250626802
ENSGALG00010012750	0.985494141	0.372589937
ENSGALG00010012751	0.382747803	0.311297893
ENSGALG00010012753	0.906052205	0.365129114
ENSGALG00010012754	0.870283988	0.085269952
ENSGALG00010012756	0.903604259	0.137741229
ENSGALG00010012757	0.038205268	0.175236885

ENSGALG00010012758	0.97095346	0.359161351
ENSGALG00010012759	0.989052133	0.359211312
ENSGALG00010012760	0.77409288	0.136832823
ENSGALG00010012761	0.95179082	0.39795743
ENSGALG00010012763	0.808974326	0.161374599
ENSGALG00010012764	0.07135513	0.010771078
ENSGALG00010012765	0.962934691	0.269581059
ENSGALG00010012767	0.793837951	0.267291135
ENSGALG00010012768	0.146831018	0.000510697
ENSGALG00010012769	0.662007748	0.33640812
ENSGALG00010012770	0.399510177	0.196523757
ENSGALG00010012771	0.907945243	0.252282549
ENSGALG00010012772	0.595497275	0.208160805
ENSGALG00010012773	0.347912442	0.498477449
ENSGALG00010012774	0.21646968	0.224197516
ENSGALG00010012775	0.96202123	0.332127496
ENSGALG00010012778	0.90025796	0.392769876
ENSGALG00010012780	0.940752835	0.366457481
ENSGALG00010012783	0.185154775	0.182180622
ENSGALG00010012786	0.921248732	0.259053193
ENSGALG00010012787	0.759281927	0.242570863
ENSGALG00010012788	0.916752731	0.280237842
ENSGALG00010012790	0.500575338	0.355176858
ENSGALG00010012791	0.507722517	0.294539081
ENSGALG00010012792	0.983583058	0.312096022
ENSGALG00010012793	0.395803147	0.099256728
ENSGALG00010012794	0.852638829	0.277132728
ENSGALG00010012795	0.489736563	0.24283089
ENSGALG00010012796	0.29209194	0.099161951
ENSGALG00010012797	0.583693577	0.098099878
ENSGALG00010012798	0.967040519	0.377354206
ENSGALG00010012800	0.391612505	0.15490595
ENSGALG00010012801	0.741376978	0.079203761
ENSGALG00010012803	0.69160482	0.532495057
ENSGALG00010012804	0.266137114	0.107784983
ENSGALG00010012806	0.237507215	0.033759299
ENSGALG00010012808	0.472235506	0.228108097
ENSGALG00010012809	0.881511393	0.245213881
ENSGALG00010012810	0.564422018	0.153298018
ENSGALG00010012811	0.375669891	0.092329224
ENSGALG00010012812	0.97780267	0.281859387
ENSGALG00010012813	0.375832149	0.020903857
ENSGALG00010012815	0.400855084	0.026171984
ENSGALG00010012817	0.235630588	0.132393411
ENSGALG00010012818	0.971199746	0.297327881
ENSGALG00010012819	0.583806038	0.036279357
ENSGALG00010012820	0.490568407	0.142044933
ENSGALG00010012821	0.557794485	0.314986758
ENSGALG00010012822	0.659131761	0.230764572
ENSGALG00010012823	0.115331852	0.198431602
ENSGALG00010012824	0.799724669	0.319612781
ENSGALG00010012827	0.285185205	0.344420234
ENSGALG00010012829	0.4696407	0.312849397
ENSGALG00010012830	0.872538587	0.263262315
ENSGALG00010012831	0.708032956	0.199106175
ENSGALG00010012832	0.072840675	0.489703365

ENSGALG00010012833	0.976783979	0.334550276
ENSGALG00010012837	0.228787754	0.341655482
ENSGALG00010012838	0.783800537	0.083616413
ENSGALG00010012839	0.853713832	0.146595111
ENSGALG00010012841	0.419664541	0.148824383
ENSGALG00010012842	0.61905265	0.420773165
ENSGALG00010012843	0.195138246	0.231482553
ENSGALG00010012845	0.108710153	0.051234388
ENSGALG00010012846	0.219386918	0.079388121
ENSGALG00010012847	0.606221342	0.295742364
ENSGALG00010012848	0.49698425	0.254645201
ENSGALG00010012849	0.988653324	0.315956341
ENSGALG00010012850	0.140427317	0.043703647
ENSGALG00010012851	0.44912152	0.175224723
ENSGALG00010012852	0.978920651	0.38760232
ENSGALG00010012853	0.517899914	0.299304996
ENSGALG00010012854	0.791724325	0.221416023
ENSGALG00010012856	0.142623147	0.224892044
ENSGALG00010012857	0.967215219	0.278068684
ENSGALG00010012858	0.435289332	0.284671205
ENSGALG00010012859	0.051066209	0.423098498
ENSGALG00010012861	0.426077687	0.046484676
ENSGALG00010012863	0.553129673	0.133932121
ENSGALG00010012864	0.798911201	0.333239032
ENSGALG00010012865	0.360928096	0.355037954
ENSGALG00010012867	0.947897154	0.311950861
ENSGALG00010012868	0.594361064	0.127159578
ENSGALG00010012869	0.441003917	0.00014656
ENSGALG00010012870	0.62979145	0.210554592
ENSGALG00010012871	0.931448409	0.337425464
ENSGALG00010012872	0.495402284	0.127956075
ENSGALG00010012873	0.956056924	0.321360548
ENSGALG00010012874	0.322438603	0.055248649
ENSGALG00010012875	0.556689828	0.103602014
ENSGALG00010012876	0.611661406	0.494775619
ENSGALG00010012877	0.874127723	0.279158968
ENSGALG00010012878	0.950008175	0.277731572
ENSGALG00010012879	0.759498468	0.285231398
ENSGALG00010012880	0.488416338	0.499905741
ENSGALG00010012882	0.068222048	0.118981567
ENSGALG00010012883	0.636877013	0.031293119
ENSGALG00010012885	0.431701014	0.015237304
ENSGALG00010012886	0.846731822	0.423761954
ENSGALG00010012887	0.547472316	0.435242124
ENSGALG00010012889	0.969796808	0.380097946
ENSGALG00010012890	0.439976036	0.070078374
ENSGALG00010012891	0.927867736	0.277819037
ENSGALG00010012892	0.844452562	0.140296981
ENSGALG00010012893	0.970288748	0.338434464
ENSGALG00010012894	0.925763108	0.186861865
ENSGALG00010012895	0.559355469	0.17881983
ENSGALG00010012896	0.796751659	0.213931251
ENSGALG00010012898	0.97468173	0.328796166
ENSGALG00010012899	0.507931946	0.42824933
ENSGALG00010012900	0.968076662	0.276091666
ENSGALG00010012901	0.560358098	0.092936969

ENSGALG00010012902	0.409026918	0.082023497
ENSGALG00010012903	0.942910984	0.404307597
ENSGALG00010012904	0.993004092	0.359003377
ENSGALG00010012905	0.107321708	0.10166927
ENSGALG00010012907	0.396343306	0.152518913
ENSGALG00010012908	0.953249642	0.245105459
ENSGALG00010012909	0.331118301	0.219019524
ENSGALG00010012910	0.917405148	0.294920562
ENSGALG00010012911	0.718870699	0.214919053
ENSGALG00010012912	0.734728052	0.156530679
ENSGALG00010012913	0.90404366	0.413757384
ENSGALG00010012914	0.285272007	0.109169955
ENSGALG00010012915	0.970984761	0.301824694
ENSGALG00010012917	0.850346273	0.350327126
ENSGALG00010012918	0.473579686	0.389254386
ENSGALG00010012920	0.518726358	0.25865655
ENSGALG00010012921	0.948071378	0.393805212
ENSGALG00010012922	0.814749192	0.418986845
ENSGALG00010012924	0.537735269	0.371708793
ENSGALG00010012925	0.115956742	0.449011231
ENSGALG00010012926	0.329515169	0.094740356
ENSGALG00010012928	0.061961029	0.225806868
ENSGALG00010012930	0.979250041	0.339994855
ENSGALG00010012932	0.790116206	0.441666641
ENSGALG00010012933	0.730453925	0.066477721
ENSGALG00010012934	0.970570105	0.338671444
ENSGALG00010012936	0.676940632	0.262331541
ENSGALG00010012938	0.967154071	0.345180807
ENSGALG00010012939	0.881873301	0.291313173
ENSGALG00010012940	0.947755458	0.381162726
ENSGALG00010012941	0.307432809	0.129569006
ENSGALG00010012942	0.594006965	0.057682925
ENSGALG00010012943	0.40134687	0.153836383
ENSGALG00010012944	0.869044407	0.215148288
ENSGALG00010012945	0.923547524	0.342557383
ENSGALG00010012946	0.672549599	0.224112568
ENSGALG00010012948	0.2105561	0.162872232
ENSGALG00010012949	0.268460109	0.19945332
ENSGALG00010012952	0.640339947	0.212828371
ENSGALG00010012954	0.937181892	0.231199679
ENSGALG00010012955	0.83642141	0.267069899
ENSGALG00010012956	0.974686216	0.336426532
ENSGALG00010012957	0.714136038	0.031180043
ENSGALG00010012958	0.782387758	0.428807278
ENSGALG00010012959	0.749533794	0.229108988
ENSGALG00010012961	0.552861553	0.077303129
ENSGALG00010012962	0.88256413	0.322308501
ENSGALG00010012963	0.861064218	0.3211267
ENSGALG00010012964	0.745105931	0.245959051
ENSGALG00010012965	0.458057322	0.365278772
ENSGALG00010012966	0.966740846	0.250119741
ENSGALG00010012967	0.912472311	0.362886702
ENSGALG00010012968	0.170159909	0.134031172
ENSGALG00010012969	0.867121995	0.337643634
ENSGALG00010012970	0.919847738	0.355828441
ENSGALG00010012971	0.937410793	0.19651895

ENSGALG00010012973	0.993956966	0.346106796
ENSGALG00010012974	0.85487209	0.367531899
ENSGALG00010012975	0.241449749	0.155703328
ENSGALG00010012976	0.981728147	0.313083312
ENSGALG00010012977	0.802844204	0.228419912
ENSGALG00010012978	0.957862714	0.357894685
ENSGALG00010012979	0.829424903	0.555206955
ENSGALG00010012980	0.91374047	0.408727491
ENSGALG00010012982	0.898517054	0.292105935
ENSGALG00010012985	0.491976616	0.335944318
ENSGALG00010012986	0.234659357	0.115936103
ENSGALG00010012988	0.787070652	0.259030077
ENSGALG00010012989	0.940310474	0.329299278
ENSGALG00010012990	0.009670608	0.189027234
ENSGALG00010012991	0.947946366	0.306567977
ENSGALG00010012992	0.502468036	0.526580726
ENSGALG00010012993	0.183571827	0.085549668
ENSGALG00010012994	0.270197877	0.12072243
ENSGALG00010012995	0.901276134	0.318071766
ENSGALG00010012996	0.553663145	0.223151606
ENSGALG00010012997	0.963370025	0.297926214
ENSGALG00010012998	0.253223789	0.307730743
ENSGALG00010012999	0.38742626	0.154147022
ENSGALG00010013000	0.436322255	0.266523534
ENSGALG00010013001	0.849160288	0.205370042
ENSGALG00010013002	0.817563199	0.22553925
ENSGALG00010013003	0.213460918	0.214911948
ENSGALG00010013004	0.519050546	0.179642471
ENSGALG00010013005	0.994108707	0.332750942
ENSGALG00010013006	0.265558089	0.028821141
ENSGALG00010013007	0.288631369	0.467279012
ENSGALG00010013008	0.621536748	0.145694015
ENSGALG00010013010	0.300391512	0.124478907
ENSGALG00010013012	0.997877074	0.331027671
ENSGALG00010013013	0.725814404	0.308959569
ENSGALG00010013015	0.57639275	0.259579482
ENSGALG00010013016	0.758045299	0.012940876
ENSGALG00010013017	0.966636404	0.373068717
ENSGALG00010013018	0.965182589	0.346374251
ENSGALG00010013019	0.994261955	0.348011998
ENSGALG00010013021	0.958534206	0.267805556
ENSGALG00010013022	0.544039905	0.313740514
ENSGALG00010013023	0.625843054	0.316717625
ENSGALG00010013024	0.693623004	0.566100671
ENSGALG00010013027	0.205317021	0.093923131
ENSGALG00010013029	0.995906744	0.335332852
ENSGALG00010013030	0.010672989	0.47373084
ENSGALG00010013032	0.630689551	0.483978479
ENSGALG00010013033	0.978460655	0.311685164
ENSGALG00010013035	0.311289807	0.13851383
ENSGALG00010013036	0.11416988	0.03314163
ENSGALG00010013037	0.868201588	0.320925007
ENSGALG00010013038	0.292351562	0.560458597
ENSGALG00010013040	0.980408905	0.366120724
ENSGALG00010013041	0.700531119	0.189755848
ENSGALG00010013042	0.014273076	0.035587719

ENSGALG00010013044	0.422397174	0.017509004
ENSGALG00010013045	0.927684134	0.27154013
ENSGALG00010013046	0.672269288	0.116974254
ENSGALG00010013047	0.959678841	0.34424319
ENSGALG00010013048	0.943974911	0.215139548
ENSGALG00010013050	0.511740883	0.003206151
ENSGALG00010013051	0.878139351	0.2086729
ENSGALG00010013052	0.115193317	0.000304681
ENSGALG00010013053	0.954398203	0.327928714
ENSGALG00010013054	0.183950837	0.323670436
ENSGALG00010013055	0.18067859	0.541007104
ENSGALG00010013056	0.711594416	0.001865934
ENSGALG00010013057	0.39144912	0.115895902
ENSGALG00010013059	0.084821793	0.146645445
ENSGALG00010013061	0.342359845	0.110452699
ENSGALG00010013062	0.929017926	0.406505118
ENSGALG00010013063	0.966192077	0.325715606
ENSGALG00010013064	0.382985366	0.1291087
ENSGALG00010013065	0.858419776	0.296573329
ENSGALG00010013066	0.046063829	0.220739248
ENSGALG00010013067	0.394752998	0.016143287
ENSGALG00010013068	0.128163573	0.21927169
ENSGALG00010013071	0.600813367	0.315438455
ENSGALG00010013072	0.739299988	0.108395845
ENSGALG00010013073	0.892602529	0.308174255
ENSGALG00010013074	0.499459571	0.152683706
ENSGALG00010013075	0.93303487	0.332848189
ENSGALG00010013076	0.003057108	0.276477933
ENSGALG00010013077	0.797443036	0.272307756
ENSGALG00010013078	0.875830461	0.445819675
ENSGALG00010013079	0.308547832	0.172637563
ENSGALG00010013080	0.74025737	0.222284886
ENSGALG00010013081	0.584770204	0.084517054
ENSGALG00010013082	0.076007605	0.054683997
ENSGALG00010013084	0.309286978	0.063036553
ENSGALG00010013085	0.904206818	0.257036731
ENSGALG00010013086	0.917451194	0.345050521
ENSGALG00010013087	0.371571672	0.199269701
ENSGALG00010013088	0.725372273	0.017422217
ENSGALG00010013089	0.944195551	0.436636004
ENSGALG00010013090	0.720035748	0.101905142
ENSGALG00010013093	0.614921729	0.646970853
ENSGALG00010013094	0.580623646	0.095274839
ENSGALG00010013095	0.220398798	0.00169847
ENSGALG00010013097	0.904049479	0.114567101
ENSGALG00010013098	0.632899202	0.307043835
ENSGALG00010013099	0.987174201	0.316707884
ENSGALG00010013100	0.851463163	0.139170999
ENSGALG00010013102	0.02951855	0.28924241
ENSGALG00010013103	0.168217376	0.037373782
ENSGALG00010013104	0.972739226	0.34140809
ENSGALG00010013105	0.914411447	0.310687498
ENSGALG00010013106	0.699591775	0.134683174
ENSGALG00010013107	0.622879379	0.244012617
ENSGALG00010013108	0.60640255	0.114394736
ENSGALG00010013109	0.38742626	0.154147022

ENSGALG00010013110	0.986998597	0.332530336
ENSGALG00010013111	0.602543109	0.691259317
ENSGALG00010013112	0.869399386	0.324034779
ENSGALG00010013114	0.124407669	0.194148845
ENSGALG00010013115	0.897951674	0.30085732
ENSGALG00010013116	0.814164756	0.273467996
ENSGALG00010013117	0.969189176	0.207147226
ENSGALG00010013119	0.868927623	0.404759824
ENSGALG00010013120	0.075263436	0.11005094
ENSGALG00010013121	0.004573262	0.036546283
ENSGALG00010013123	0.925665337	0.210545365
ENSGALG00010013125	0.112239646	0.237257893
ENSGALG00010013129	0.298939958	0.247157261
ENSGALG00010013130	0.515648159	0.221536523
ENSGALG00010013131	0.86877517	0.34194829
ENSGALG00010013132	0.887508691	0.103808911
ENSGALG00010013133	0.432649518	0.190750491
ENSGALG00010013135	0.958026295	0.407977501
ENSGALG00010013136	0.452135376	0.282059989
ENSGALG00010013137	0.208964901	0.069432382
ENSGALG00010013138	0.683247644	0.207785485
ENSGALG00010013139	0.601516764	0.280227367
ENSGALG00010013140	0.265558089	0.028821141
ENSGALG00010013141	0.158511397	0.254537799
ENSGALG00010013143	0.251949149	0.270716132
ENSGALG00010013144	0.791501955	0.068577514
ENSGALG00010013145	0.138801209	0.222746195
ENSGALG00010013146	0.407265606	0.386201405
ENSGALG00010013147	0.210713871	0.115110736
ENSGALG00010013149	0.987115303	0.348793321
ENSGALG00010013150	0.905970137	0.431645065
ENSGALG00010013151	0.694508057	0.251340183
ENSGALG00010013152	0.9858858	0.355944909
ENSGALG00010013153	0.984444728	0.351033634
ENSGALG00010013154	0.039738209	0.18370405
ENSGALG00010013155	0.421886629	0.000190121
ENSGALG00010013156	0.931049937	0.305664062
ENSGALG00010013157	0.1910296	0.287114534
ENSGALG00010013158	0.812590885	0.53724116
ENSGALG00010013159	0.125281441	0.118461904
ENSGALG00010013160	0.685051597	0.334884982
ENSGALG00010013161	0.270451009	0.090541667
ENSGALG00010013162	0.94359101	0.457438155
ENSGALG00010013163	0.284129909	0.290334841
ENSGALG00010013164	0.662118434	0.218933793
ENSGALG00010013165	0.714446419	0.416103436
ENSGALG00010013166	0.001199338	0.314578006
ENSGALG00010013167	0.140453364	0.019948235
ENSGALG00010013168	0.929333291	0.198956143
ENSGALG00010013170	0.922155003	0.545166182
ENSGALG00010013171	0.865357157	0.466686804
ENSGALG00010013172	0.878893704	0.256200193
ENSGALG00010013173	0.582627145	0.247109925
ENSGALG00010013174	0.198191448	0.329896384
ENSGALG00010013175	0.968998033	0.315365358
ENSGALG00010013176	0.787095582	0.30351993

ENSGALG00010013177	0.812332589	0.311775053
ENSGALG00010013179	0.943667915	0.228953818
ENSGALG00010013180	0.977884335	0.30196188
ENSGALG00010013181	0.946254185	0.23885685
ENSGALG00010013182	0.908422762	0.20536705
ENSGALG00010013183	0.978671824	0.318206693
ENSGALG00010013184	0.980971978	0.295313671
ENSGALG00010013185	0.235630588	0.132393411
ENSGALG00010013186	0.758100695	0.374579111
ENSGALG00010013187	0.96766936	0.394697522
ENSGALG00010013188	0.854212448	0.276834145
ENSGALG00010013190	0.377961669	0.085197514
ENSGALG00010013191	0.967986434	0.32332636
ENSGALG00010013192	0.94292625	0.22325484
ENSGALG00010013194	0.893537899	0.136317248
ENSGALG00010013196	0.883861953	0.293160496
ENSGALG00010013197	0.260964826	0.225732368
ENSGALG00010013198	0.720981377	0.397696241
ENSGALG00010013199	0.179605246	0.330783199
ENSGALG00010013200	0.488705491	0.213231432
ENSGALG00010013201	0.99443267	0.333383182
ENSGALG00010013203	0.977356503	0.35727055
ENSGALG00010013205	0.213052756	0.05934831
ENSGALG00010013206	0.867460006	0.26941595
ENSGALG00010013207	0.963837447	0.385649031
ENSGALG00010013209	0.630351515	0.095075307
ENSGALG00010013212	0.532936589	0.31342651
ENSGALG00010013213	0.88502559	0.219231208
ENSGALG00010013214	0.978925145	0.286120388
ENSGALG00010013215	0.828686031	0.227712533
ENSGALG00010013216	0.117823061	0.361091486
ENSGALG00010013217	0.729843171	0.172421047
ENSGALG00010013219	0.811680645	0.450293042
ENSGALG00010013220	0.59724041	0.302964513
ENSGALG00010013221	0.319654199	0.155938046
ENSGALG00010013222	0.496004799	0.24797181
ENSGALG00010013223	0.93554292	0.322431912
ENSGALG00010013224	0.073099841	0.111283213
ENSGALG00010013225	0.991738041	0.36318577
ENSGALG00010013226	0.907403298	0.275964468
ENSGALG00010013227	0.984963854	0.327578122
ENSGALG00010013228	0.893486209	0.271024401
ENSGALG00010013230	0.924765395	0.362815731
ENSGALG00010013231	0.827338693	0.262283987
ENSGALG00010013232	0.959520964	0.223183056
ENSGALG00010013233	0.409080899	0.210897434
ENSGALG00010013234	0.683350354	0.163485719
ENSGALG00010013235	0.993060623	0.348360901
ENSGALG00010013236	0.871626274	0.294063497
ENSGALG00010013237	0.311987459	0.064338115
ENSGALG00010013238	0.703590621	0.201134517
ENSGALG00010013241	0.756261918	0.204456111
ENSGALG00010013242	0.517531226	0.01408974
ENSGALG00010013243	0.511276417	0.122678557
ENSGALG00010013244	0.107716594	0.135876583
ENSGALG00010013245	0.350952883	0.063602806

ENSGALG00010013246	0.736051026	0.229124712
ENSGALG00010013247	0.278512197	0.029109271
ENSGALG00010013248	0.671507657	0.205539897
ENSGALG00010013249	0.928978543	0.231632983
ENSGALG00010013250	0.8314366	0.150307902
ENSGALG00010013251	0.770753794	0.055345886
ENSGALG00010013252	0.957483237	0.254779086
ENSGALG00010013253	0.659036718	0.266415328
ENSGALG00010013254	0.884643102	0.265866148
ENSGALG00010013256	0.462525453	0.417096582
ENSGALG00010013257	0.925700124	0.30578776
ENSGALG00010013258	0.969193198	0.250569655
ENSGALG00010013259	0.77384115	0.158539683
ENSGALG00010013260	0.781091693	0.296710153
ENSGALG00010013261	0.114038241	0.142274536
ENSGALG00010013262	0.564761658	0.100380263
ENSGALG00010013263	0.567138491	0.535368281
ENSGALG00010013264	0.73794176	0.280801012
ENSGALG00010013265	0.988173026	0.347186177
ENSGALG00010013266	0.714568125	0.300822113
ENSGALG00010013267	0.584338218	0.25877026
ENSGALG00010013268	0.269852975	0.090177272
ENSGALG00010013269	0.849234942	0.321730533
ENSGALG00010013271	0.971363874	0.332151975
ENSGALG00010013272	0.169840649	0.372819642
ENSGALG00010013273	0.573228346	0.009120614
ENSGALG00010013274	0.33880764	0.065819685
ENSGALG00010013275	0.958166082	0.316156038
ENSGALG00010013276	0.904213529	0.159264279
ENSGALG00010013277	0.419905985	0.356392381
ENSGALG00010013278	0.912734752	0.30448006
ENSGALG00010013280	0.838149021	0.37910399
ENSGALG00010013281	0.254372136	0.084986156
ENSGALG00010013282	0.90703278	0.430339494
ENSGALG00010013283	0.956925139	0.278585679
ENSGALG00010013284	0.746442053	0.248942741
ENSGALG00010013285	0.945265931	0.236006693
ENSGALG00010013286	0.98831562	0.322821924
ENSGALG00010013287	0.586062417	0.31224614
ENSGALG00010013288	0.947029882	0.203764447
ENSGALG00010013289	0.915322245	0.350249485
ENSGALG00010013290	0.967538657	0.314506179
ENSGALG00010013292	0.963250955	0.343767905
ENSGALG00010013293	0.793317709	0.349871775
ENSGALG00010013294	0.477279458	0.248887179
ENSGALG00010013295	0.919017107	0.275337699
ENSGALG00010013296	0.679209494	0.280387331
ENSGALG00010013297	0.952837126	0.32558232
ENSGALG00010013298	0.241101206	0.002785488
ENSGALG00010013299	0.814160164	0.389754697
ENSGALG00010013300	0.571992562	0.185082298
ENSGALG00010013301	0.174808906	0.29606967
ENSGALG00010013302	0.90271931	0.377168776
ENSGALG00010013303	0.805968631	0.334999426
ENSGALG00010013304	0.618388544	0.006844153
ENSGALG00010013305	0.960993185	0.385132691

ENSGALG00010013306	0.967879144	0.417436979
ENSGALG00010013307	0.943617214	0.217902823
ENSGALG00010013308	0.262922434	0.138601464
ENSGALG00010013309	0.984271661	0.33483391
ENSGALG00010013310	0.747083524	0.089864788
ENSGALG00010013311	0.633063259	0.380860974
ENSGALG00010013312	0.98023071	0.369412974
ENSGALG00010013313	0.9370202	0.21464178
ENSGALG00010013314	0.640319965	0.331231268
ENSGALG00010013315	0.8165783	0.387686241
ENSGALG00010013316	0.484243242	0.243431008
ENSGALG00010013317	0.858305245	0.185318596
ENSGALG00010013318	0.508940577	0.176445915
ENSGALG00010013319	0.240460951	0.020222045
ENSGALG00010013320	0.933278983	0.357473663
ENSGALG00010013321	0.008043983	0.110635211
ENSGALG00010013322	0.323910544	0.136128073
ENSGALG00010013323	0.606637975	0.022466053
ENSGALG00010013324	0.825567408	0.104693723
ENSGALG00010013325	0.969676045	0.337444786
ENSGALG00010013327	0.834952501	0.030856014
ENSGALG00010013328	0.868797633	0.305141863
ENSGALG00010013329	0.452202018	0.263432322
ENSGALG00010013330	0.844139814	0.163385685
ENSGALG00010013331	0.180502179	0.111455836
ENSGALG00010013332	0.820600463	0.475040776
ENSGALG00010013333	0.422768985	0.414231063
ENSGALG00010013334	0.193011454	0.300323358
ENSGALG00010013335	0.991734943	0.31438837
ENSGALG00010013336	0.399315634	0.170126231
ENSGALG00010013337	0.948818743	0.319830269
ENSGALG00010013339	0.241024924	0.087857425
ENSGALG00010013340	0.042950378	0.119158415
ENSGALG00010013341	0.222107324	0.165490218
ENSGALG00010013342	0.754957766	0.278207527
ENSGALG00010013343	0.76291645	0.114823774
ENSGALG00010013344	0.920234413	0.204346682
ENSGALG00010013345	0.438912388	0.163960374
ENSGALG00010013346	0.990636738	0.342778235
ENSGALG00010013347	0.597238867	0.016532654
ENSGALG00010013348	0.760544084	0.014365077
ENSGALG00010013349	0.842026211	0.220852909
ENSGALG00010013351	0.123591786	0.155530229
ENSGALG00010013352	0.832525453	0.341066876
ENSGALG00010013353	0.281311598	0.096832751
ENSGALG00010013354	0.978237876	0.29002466
ENSGALG00010013355	0.851115432	0.291705194
ENSGALG00010013356	0.236405177	0.364303255
ENSGALG00010013357	0.765435801	0.391412403
ENSGALG00010013358	0.820488445	0.274513987
ENSGALG00010013359	0.536669509	0.206493997
ENSGALG00010013360	0.423752321	0.012793047
ENSGALG00010013361	0.135893973	0.150450603
ENSGALG00010013362	0.802918176	0.422739897
ENSGALG00010013363	0.978985086	0.258542128
ENSGALG00010013364	0.453478428	0.066023984

ENSGALG00010013365	0.986071565	0.346767345
ENSGALG00010013367	0.351877136	0.23221238
ENSGALG00010013369	0.948862512	0.285634459
ENSGALG00010013370	0.931049935	0.231617044
ENSGALG00010013371	0.271769152	0.440076841
ENSGALG00010013372	0.69229792	0.130421389
ENSGALG00010013373	0.184698419	0.179519181
ENSGALG00010013374	0.945733669	0.28636718
ENSGALG00010013375	0.848784727	0.228988019
ENSGALG00010013376	0.694641595	0.010547628
ENSGALG00010013377	0.696164718	0.13701595
ENSGALG00010013378	0.388715732	0.4421999
ENSGALG00010013379	0.743670625	0.330704496
ENSGALG00010013380	0.986635857	0.327080832
ENSGALG00010013381	0.288870458	0.091189751
ENSGALG00010013382	0.985254247	0.315142748
ENSGALG00010013383	0.944075685	0.236386228
ENSGALG00010013385	0.969013936	0.309655748
ENSGALG00010013386	0.973773249	0.239973996
ENSGALG00010013387	0.178005602	0.341033526
ENSGALG00010013388	0.885025163	0.23914153
ENSGALG00010013389	0.434234727	0.003295498
ENSGALG00010013390	0.983944973	0.350576928
ENSGALG00010013391	0.650539331	0.040757933
ENSGALG00010013392	0.749805707	0.304630512
ENSGALG00010013393	0.815004174	0.208231805
ENSGALG00010013394	0.359598781	0.229280128
ENSGALG00010013395	0.875597493	0.305807856
ENSGALG00010013396	0.984118739	0.399402825
ENSGALG00010013397	0.539803971	0.062371104
ENSGALG00010013398	0.957002566	0.320185847
ENSGALG00010013399	0.961235149	0.34671248
ENSGALG00010013400	0.515204355	0.058332602
ENSGALG00010013401	0.134167114	0.295549581
ENSGALG00010013402	0.268088855	0.093096726
ENSGALG00010013403	0.649081346	0.512938285
ENSGALG00010013404	0.512032584	0.081947435
ENSGALG00010013405	0.696921871	0.241560726
ENSGALG00010013407	0.984338788	0.313215144
ENSGALG00010013408	0.588497287	0.07531384
ENSGALG00010013409	0.904060959	0.348244023
ENSGALG00010013410	0.639149559	0.04186644
ENSGALG00010013411	0.330792121	0.275586713
ENSGALG00010013412	0.18337826	0.122907007
ENSGALG00010013413	0.568198393	0.287191062
ENSGALG00010013414	0.981411796	0.331148379
ENSGALG00010013416	0.921530742	0.298133028
ENSGALG00010013417	0.976068247	0.260557553
ENSGALG00010013418	0.262922434	0.138601464
ENSGALG00010013419	0.936846776	0.207138513
ENSGALG00010013422	0.547294606	0.184886278
ENSGALG00010013423	0.623429205	0.299605744
ENSGALG00010013427	0.328311737	0.228937941
ENSGALG00010013428	0.996021046	0.330224944
ENSGALG00010013429	0.995317844	0.319782381
ENSGALG00010013431	0.90984141	0.141373613

ENSGALG00010013434	0.59290471	0.266170684
ENSGALG00010013435	0.923853845	0.404004899
ENSGALG00010013436	0.852231429	0.286562245
ENSGALG00010013438	0.873051806	0.288304988
ENSGALG00010013440	0.480195756	0.052808496
ENSGALG00010013442	0.578088603	0.011888647
ENSGALG00010013444	0.89304037	0.375313843
ENSGALG00010013445	0.738593172	0.204061191
ENSGALG00010013446	0.927150562	0.336992293
ENSGALG00010013449	0.544848113	0.349996674
ENSGALG00010013450	0.850190808	0.199788783
ENSGALG00010013451	0.995834288	0.336131194
ENSGALG00010013452	0.959237256	0.280105807
ENSGALG00010013453	0.400220827	0.320417892
ENSGALG00010013455	0.765383297	0.235972944
ENSGALG00010013457	0.991460687	0.327534002
ENSGALG00010013458	0.977243734	0.362476139
ENSGALG00010013459	0.990159943	0.351650065
ENSGALG00010013461	0.36652196	0.331420743
ENSGALG00010013463	0.975983671	0.348818988
ENSGALG00010013464	0.962303449	0.410786926
ENSGALG00010013465	0.768314592	0.109906416
ENSGALG00010013466	0.946667784	0.335019493
ENSGALG00010013468	0.590026697	0.080286296
ENSGALG00010013470	0.745791344	0.257552699
ENSGALG00010013471	0.055262695	0.13983149
ENSGALG00010013472	0.970626979	0.302258729
ENSGALG00010013476	0.916875846	0.473386762
ENSGALG00010013477	0.855125268	0.217483473
ENSGALG00010013480	0.193391989	0.193137629
ENSGALG00010013482	0.947885514	0.190420877
ENSGALG00010013483	0.211576192	0.519691433
ENSGALG00010013484	0.461984661	0.10011435
ENSGALG00010013486	0.930254891	0.271731727
ENSGALG00010013487	0.8393986	0.186419299
ENSGALG00010013488	0.505406403	0.087710092
ENSGALG00010013491	0.970050524	0.371412432
ENSGALG00010013492	0.965455549	0.457324921
ENSGALG00010013493	0.604951726	0.113584932
ENSGALG00010013494	0.983331714	0.323961738
ENSGALG00010013497	0.705611646	0.17102537
ENSGALG00010013498	0.922365608	0.202459475
ENSGALG00010013499	0.365840833	0.116281684
ENSGALG00010013501	0.593995261	0.574271617
ENSGALG00010013502	0.846919396	0.263461541
ENSGALG00010013504	0.992000952	0.334166044
ENSGALG00010013506	0.916016999	0.272863388
ENSGALG00010013508	0.687627232	0.399320342
ENSGALG00010013509	0.90075034	0.188918237
ENSGALG00010013510	0.905660754	0.272860862
ENSGALG00010013511	0.90741628	0.413301943
ENSGALG00010013514	0.950486658	0.197076667
ENSGALG00010013515	0.800312508	0.15112995
ENSGALG00010013516	0.032691858	0.028516063
ENSGALG00010013517	0.451246798	0.269628386
ENSGALG00010013520	0.708644999	0.258021366

ENSGALG00010013521	0.285272007	0.109169955
ENSGALG00010013522	0.956349943	0.182601946
ENSGALG00010013523	0.120992588	0.016455751
ENSGALG00010013524	0.735227674	0.28895601
ENSGALG00010013526	0.781746106	0.060019751
ENSGALG00010013527	0.087531075	0.288756603
ENSGALG00010013528	0.813399725	0.227770221
ENSGALG00010013529	0.75690815	0.334549675
ENSGALG00010013530	0.99187316	0.24800033
ENSGALG00010013531	0.117712394	0.565090935
ENSGALG00010013532	0.236275731	0.137738166
ENSGALG00010013533	0.595535074	0.060272568
ENSGALG00010013537	0.391101935	0.432129674
ENSGALG00010013538	0.624406287	0.195553325
ENSGALG00010013539	0.970454686	0.42436581
ENSGALG00010013541	0.31557269	0.39976646
ENSGALG00010013544	0.219643828	0.187072219
ENSGALG00010013546	0.96943687	0.379497766
ENSGALG00010013548	0.510602098	0.088101245
ENSGALG00010013549	0.938843967	0.277372067
ENSGALG00010013550	0.945007956	0.169768886
ENSGALG00010013551	0.957677762	0.291206566
ENSGALG00010013552	0.952046711	0.423527036
ENSGALG00010013553	0.847709701	0.072208535
ENSGALG00010013555	0.385267219	0.242462386
ENSGALG00010013556	0.578762219	0.220888407
ENSGALG00010013557	0.606880617	0.227656784
ENSGALG00010013558	0.542714762	0.060880498
ENSGALG00010013560	0.565739386	0.167874974
ENSGALG00010013561	0.960056687	0.326699093
ENSGALG00010013562	0.393080833	0.386838195
ENSGALG00010013563	0.980046352	0.372556471
ENSGALG00010013564	0.277566769	0.107857127
ENSGALG00010013566	0.332587279	0.522153151
ENSGALG00010013567	0.359887877	0.14396678
ENSGALG00010013568	0.09373728	0.650232439
ENSGALG00010013569	0.369613871	0.180196378
ENSGALG00010013570	0.019937434	0.451537925
ENSGALG00010013571	0.154945677	0.222990059
ENSGALG00010013572	0.964928935	0.430915467
ENSGALG00010013573	0.453898094	0.295305384
ENSGALG00010013574	0.961050579	0.292559254
ENSGALG00010013575	0.558432172	0.198548276
ENSGALG00010013576	0.985454928	0.348037623
ENSGALG00010013577	0.048041984	0.111727973
ENSGALG00010013578	0.258991126	0.176988502
ENSGALG00010013579	0.381549186	0.357971457
ENSGALG00010013580	0.417166272	0.023866057
ENSGALG00010013581	0.200570274	0.091667991
ENSGALG00010013582	0.946654513	0.271194803
ENSGALG00010013583	0.85545351	0.048127849
ENSGALG00010013584	0.744110623	0.11544466
ENSGALG00010013585	0.572771993	0.48662517
ENSGALG00010013586	0.218659563	0.138944896
ENSGALG00010013587	0.864659703	0.296139467
ENSGALG00010013588	0.026681103	0.120260695

ENSGALG00010013589	0.519837246	0.233559392
ENSGALG00010013592	0.546488069	0.058041499
ENSGALG00010013595	0.785802957	0.32745819
ENSGALG00010013596	0.962568532	0.421094246
ENSGALG00010013598	0.151503384	0.033138894
ENSGALG00010013601	0.061290856	0.4359714
ENSGALG00010013602	0.504507995	0.124417137
ENSGALG00010013603	0.395571797	0.097272276
ENSGALG00010013604	0.792623815	0.256852828
ENSGALG00010013609	0.958779395	0.37250109
ENSGALG00010013610	0.203991683	0.058221925
ENSGALG00010013612	0.15837725	0.349448279
ENSGALG00010013613	0.157707479	0.408306698
ENSGALG00010013614	0.846603534	0.320251118
ENSGALG00010013617	0.436787766	0.519669277
ENSGALG00010013619	0.914119021	0.287396991
ENSGALG00010013621	0.948690026	0.382211684
ENSGALG00010013622	0.647844934	0.06233081
ENSGALG00010013624	0.090435602	0.117900952
ENSGALG00010013625	0.95169563	0.309198981
ENSGALG00010013626	0.918693343	0.299812048
ENSGALG00010013627	0.945220291	0.254439643
ENSGALG00010013631	0.432025504	0.268195458
ENSGALG00010013632	0.273095654	0.612321109
ENSGALG00010013634	0.064519051	0.143619078
ENSGALG00010013635	0.87343009	0.406722416
ENSGALG00010013638	0.727237639	0.230064779
ENSGALG00010013639	0.822266772	0.224385324
ENSGALG00010013640	0.666595483	0.389589126
ENSGALG00010013644	0.386764803	0.105517621
ENSGALG00010013645	0.512927448	0.046425159
ENSGALG00010013646	0.97451788	0.332106901
ENSGALG00010013647	0.586270318	0.186080162
ENSGALG00010013648	0.894055385	0.323048644
ENSGALG00010013650	0.973997479	0.339463056
ENSGALG00010013651	0.930597696	0.365657356
ENSGALG00010013654	0.111111925	0.065530994
ENSGALG00010013657	0.984086515	0.283991738
ENSGALG00010013661	0.275924968	0.400777609
ENSGALG00010013662	0.739377655	0.229123623
ENSGALG00010013663	0.869597515	0.436606669
ENSGALG00010013664	0.917418216	0.357412538
ENSGALG00010013665	0.282754916	0.323313762
ENSGALG00010013666	0.909679047	0.313494703
ENSGALG00010013667	0.982121101	0.257409809
ENSGALG00010013668	0.160348444	0.041991657
ENSGALG00010013669	0.934496672	0.259433564
ENSGALG00010013670	0.295953409	0.537078435
ENSGALG00010013671	0.786263542	0.286987032
ENSGALG00010013672	0.990146904	0.326946331
ENSGALG00010013674	0.142435963	0.414251631
ENSGALG00010013675	0.390353249	0.133787383
ENSGALG00010013676	0.924016505	0.294041359
ENSGALG00010013677	0.372823977	0.113796518
ENSGALG00010013678	0.161017956	0.126018193
ENSGALG00010013679	0.941143983	0.343876204

ENSGALG00010013682	0.747741256	0.242634981
ENSGALG00010013683	0.21947649	0.06352501
ENSGALG00010013684	0.032435623	0.168967782
ENSGALG00010013686	0.957622955	0.310376202
ENSGALG00010013687	0.497605585	0.404760238
ENSGALG00010013690	0.949177965	0.26615348
ENSGALG00010013694	0.318869103	0.609620979
ENSGALG00010013696	0.879803256	0.105478536
ENSGALG00010013697	0.689537223	0.457441822
ENSGALG00010013698	0.009812001	0.5166245
ENSGALG00010013699	0.561563494	0.011603035
ENSGALG00010013701	0.083857975	0.104781578
ENSGALG00010013703	0.285272007	0.109169955
ENSGALG00010013704	0.925119803	0.414305052
ENSGALG00010013709	0.910994859	0.182564089
ENSGALG00010013715	0.991763449	0.341968453
ENSGALG00010013717	0.847450886	0.263346901
ENSGALG00010013719	0.726484678	0.017701755
ENSGALG00010013720	0.168879283	0.316894473
ENSGALG00010013726	0.395556858	0.102965232
ENSGALG00010013731	0.03285381	0.446229691
ENSGALG00010013732	0.913206924	0.234408802
ENSGALG00010013733	0.371974189	0.241517246
ENSGALG00010013735	0.380776272	0.137181361
ENSGALG00010013736	0.010611957	0.150966622
ENSGALG00010013737	0.457714007	0.21586017
ENSGALG00010013740	0.974772492	0.31441662
ENSGALG00010013743	0.299001104	0.0947825
ENSGALG00010013744	0.983602185	0.312783132
ENSGALG00010013746	0.95144781	0.395448056
ENSGALG00010013747	0.597790263	0.24194292
ENSGALG00010013748	0.38788653	0.126003537
ENSGALG00010013749	0.970831006	0.315107312
ENSGALG00010013750	0.348639952	0.546231234
ENSGALG00010013751	0.037121486	0.150390551
ENSGALG00010013752	0.77476991	0.257452719
ENSGALG00010013753	0.601284874	0.10793913
ENSGALG00010013755	0.838072383	0.298443643
ENSGALG00010013756	0.499060521	0.489254519
ENSGALG00010013757	0.467122895	0.162310228
ENSGALG00010013758	0.227348537	0.294045536
ENSGALG00010013760	0.77205514	0.429771904
ENSGALG00010013761	0.531532005	0.388828337
ENSGALG00010013762	0.385056955	0.359188973
ENSGALG00010013763	0.812442893	0.299046875
ENSGALG00010013764	0.643253246	0.22844101
ENSGALG00010013766	0.481973279	0.284703424
ENSGALG00010013767	0.996667246	0.331019589
ENSGALG00010013768	0.032543023	0.182103637
ENSGALG00010013769	0.285272007	0.109169955
ENSGALG00010013770	0.499571562	0.142812618
ENSGALG00010013771	0.90444041	0.35087352
ENSGALG00010013772	0.164111485	0.004667785
ENSGALG00010013774	0.299628493	0.000976957
ENSGALG00010013775	0.653901762	0.436746947
ENSGALG00010013776	0.285272007	0.109169955

ENSGALG00010013777	0.755569935	0.456634143
ENSGALG00010013779	0.786661964	0.461026277
ENSGALG00010013780	0.460544355	0.045077074
ENSGALG00010013782	0.198733304	0.184433
ENSGALG00010013783	0.743857499	0.238734394
ENSGALG00010013784	0.989626842	0.32435863
ENSGALG00010013785	0.271989419	0.229446651
ENSGALG00010013786	0.939409883	0.315949299
ENSGALG00010013787	0.622325889	0.020749161
ENSGALG00010013791	0.904010303	0.5425723
ENSGALG00010013792	0.667828704	0.016206113
ENSGALG00010013793	0.543574397	0.215933744
ENSGALG00010013794	0.809378863	0.218969749
ENSGALG00010013797	0.921293091	0.122685227
ENSGALG00010013798	0.029588435	0.156172859
ENSGALG00010013799	0.153015721	0.064444297
ENSGALG00010013802	0.913379685	0.266725828
ENSGALG00010013804	0.725703624	0.259769833
ENSGALG00010013805	0.965178541	0.251623384
ENSGALG00010013808	0.840526324	0.265187266
ENSGALG00010013809	0.905512889	0.394176216
ENSGALG00010013814	0.130389581	0.042150117
ENSGALG00010013815	0.698145499	0.045479984
ENSGALG00010013820	0.988069971	0.341796837
ENSGALG00010013821	0.468461797	0.141158848
ENSGALG00010013822	0.012433676	0.134633327
ENSGALG00010013823	0.698037015	0.380001634
ENSGALG00010013824	0.987777369	0.334402072
ENSGALG00010013825	0.320558359	0.423674672
ENSGALG00010013827	0.287464551	0.092296281
ENSGALG00010013828	0.605812928	0.126593548
ENSGALG00010013830	0.398160448	0.061610243
ENSGALG00010013831	0.618776683	0.041117296
ENSGALG00010013833	0.942442796	0.332560192
ENSGALG00010013836	0.894432428	0.468321096
ENSGALG00010013837	0.90277529	0.405154734
ENSGALG00010013840	0.373495264	0.061911944
ENSGALG00010013842	0.49337701	0.166302469
ENSGALG00010013845	0.086518717	0.120092976
ENSGALG00010013846	0.997557358	0.327833353
ENSGALG00010013848	0.144349412	0.069889765
ENSGALG00010013850	0.270451009	0.090541667
ENSGALG00010013857	0.840804776	0.240806656
ENSGALG00010013859	0.967550547	0.246937141
ENSGALG00010013861	0.977361302	0.327394368
ENSGALG00010013862	0.200570274	0.091667991
ENSGALG00010013865	0.520697792	0.375173109
ENSGALG00010013866	0.273085453	0.040064011
ENSGALG00010013867	0.187706647	0.090890223
ENSGALG00010013871	0.745827762	0.171008672
ENSGALG00010013872	0.880574865	0.410231229
ENSGALG00010013874	0.187514554	0.108479243
ENSGALG00010013878	0.721614541	0.267707382
ENSGALG00010013879	0.344386169	0.246957469
ENSGALG00010013880	0.026713547	0.440572191
ENSGALG00010013886	0.528210503	0.354331696

ENSGALG00010013889	0.943860404	0.188740659
ENSGALG00010013890	0.857091038	0.275592677
ENSGALG00010013891	0.187514554	0.108479243
ENSGALG00010013893	0.930194992	0.296163237
ENSGALG00010013894	0.265558089	0.028821141
ENSGALG00010013897	0.218454689	0.027994195
ENSGALG00010013898	0.722343072	0.363440446
ENSGALG00010013899	0.942734143	0.282773125
ENSGALG00010013900	0.238171268	0.174690323
ENSGALG00010013901	0.214623807	0.071091285
ENSGALG00010013905	0.93197314	0.211596303
ENSGALG00010013907	0.960423541	0.205321367
ENSGALG00010013908	0.951726467	0.341001395
ENSGALG00010013909	0.219386918	0.079388121
ENSGALG00010013910	0.883827048	0.564157862
ENSGALG00010013911	0.979812181	0.311409148
ENSGALG00010013912	0.689274242	0.123958412
ENSGALG00010013914	0.942132216	0.392583526
ENSGALG00010013915	0.706728676	0.300830668
ENSGALG00010013916	0.208032856	0.119983309
ENSGALG00010013917	0.785689838	0.266897263
ENSGALG00010013920	0.146908091	0.084521517
ENSGALG00010013921	0.687659961	0.450778591
ENSGALG00010013923	0.773479957	0.411694308
ENSGALG00010013925	0.373495264	0.061911944
ENSGALG00010013926	0.494010744	0.129594124
ENSGALG00010013928	0.827477335	0.439476647
ENSGALG00010013929	0.696108161	0.225134489
ENSGALG00010013930	0.369634494	0.073969895
ENSGALG00010013933	0.270451009	0.090541667
ENSGALG00010013934	0.859713736	0.342429904
ENSGALG00010013935	0.107987641	0.147761113
ENSGALG00010013936	0.358447787	0.13566212
ENSGALG00010013938	0.737489006	0.364490838
ENSGALG00010013940	0.796899393	0.018392452
ENSGALG00010013942	0.574880954	0.323891812
ENSGALG00010013943	0.087780444	0.120365176
ENSGALG00010013944	0.341871658	0.269119022
ENSGALG00010013945	0.866664831	0.478281357
ENSGALG00010013946	0.606983821	0.049321487
ENSGALG00010013947	0.931966258	0.20394417
ENSGALG00010013948	0.328685149	0.093645012
ENSGALG00010013949	0.672740672	0.024936331
ENSGALG00010013950	0.983298438	0.269302938
ENSGALG00010013952	0.353859546	0.637304418
ENSGALG00010013953	0.930707748	0.302447364
ENSGALG00010013954	0.136776981	0.248452392
ENSGALG00010013955	0.069382794	0.113339801
ENSGALG00010013956	0.421905141	0.198786386
ENSGALG00010013957	0.763395204	0.034348022
ENSGALG00010013958	0.836328304	0.218289668
ENSGALG00010013959	0.720195442	0.343279464
ENSGALG00010013961	0.708584806	0.197433803
ENSGALG00010013962	0.475630301	0.154026102
ENSGALG00010013964	0.853560193	0.233025825
ENSGALG00010013965	0.997368553	0.351183696

ENSGALG00010013966	0.278703818	0.125726279
ENSGALG00010013967	0.892296343	0.260098029
ENSGALG00010013971	0.451006588	0.564375683
ENSGALG00010013973	0.654825081	0.351075371
ENSGALG00010013974	0.83915247	0.240757106
ENSGALG00010013976	0.676238859	0.030912704
ENSGALG00010013977	0.932130163	0.236276642
ENSGALG00010013978	0.145055926	0.013843908
ENSGALG00010013980	0.930447073	0.334270985
ENSGALG00010013981	0.261110229	0.186982836
ENSGALG00010013983	0.399574883	0.154317703
ENSGALG00010013986	0.55440542	0.010500745
ENSGALG00010013987	0.304747817	0.1487839
ENSGALG00010013988	0.852396147	0.242072812
ENSGALG00010013989	0.989028633	0.36840103
ENSGALG00010013990	0.007835704	0.185941901
ENSGALG00010013994	0.996566369	0.334684829
ENSGALG00010013995	0.90130388	0.481342309
ENSGALG00010013997	0.481505139	0.158115463
ENSGALG00010013998	0.312583512	0.102254388
ENSGALG00010013999	0.588330132	0.229581084
ENSGALG00010014000	0.964317092	0.284558921
ENSGALG00010014001	0.480596437	0.174483453
ENSGALG00010014002	0.292940174	0.076530023
ENSGALG00010014006	0.342357985	0.068737211
ENSGALG00010014007	0.649616476	0.208647266
ENSGALG00010014008	0.65366567	0.191188874
ENSGALG00010014009	0.30413662	0.447810957
ENSGALG00010014010	0.731643439	0.195138168
ENSGALG00010014011	0.377890827	0.365773022
ENSGALG00010014012	0.270451009	0.090541667
ENSGALG00010014014	0.924671056	0.276923492
ENSGALG00010014018	0.193412258	0.369574011
ENSGALG00010014019	0.892205455	0.262692248
ENSGALG00010014020	0.747963378	0.285676854
ENSGALG00010014025	0.24274624	0.253601774
ENSGALG00010014026	0.891049452	0.198318882
ENSGALG00010014027	0.298143852	0.421988187
ENSGALG00010014029	0.82080503	0.284537061
ENSGALG00010014030	0.559736096	0.287396892
ENSGALG00010014031	0.837368865	0.258666511
ENSGALG00010014032	0.740852737	0.282989685
ENSGALG00010014033	0.969983245	0.304022841
ENSGALG00010014035	0.340139149	0.036533269
ENSGALG00010014036	0.468227249	0.169945428
ENSGALG00010014038	0.089464534	0.206635869
ENSGALG00010014039	0.812798795	0.340329734
ENSGALG00010014041	0.705519384	0.161407197
ENSGALG00010014042	0.09497156	0.359844284
ENSGALG00010014043	0.302402586	0.196026891
ENSGALG00010014044	0.119086826	0.279869788
ENSGALG00010014045	0.115923141	0.407160789
ENSGALG00010014047	0.407959512	0.105837954
ENSGALG00010014048	0.532040899	0.37115439
ENSGALG00010014049	0.340584432	0.170258254
ENSGALG00010014051	0.340670265	0.238948705

ENSGALG00010014054	0.041527275	0.149390608
ENSGALG00010014055	0.852780713	0.180072666
ENSGALG00010014056	0.965434976	0.289848244
ENSGALG00010014058	0.569107953	0.277350009
ENSGALG00010014059	0.994972309	0.324823455
ENSGALG00010014060	0.958831416	0.291571603
ENSGALG00010014062	0.735661139	0.370191547
ENSGALG00010014063	0.751774242	0.28796075
ENSGALG00010014065	0.328035799	0.206249319
ENSGALG00010014066	0.29320785	0.140482424
ENSGALG00010014068	0.262922434	0.138601464
ENSGALG00010014072	0.358544312	0.204272193
ENSGALG00010014075	0.937530483	0.21226278
ENSGALG00010014076	0.944414301	0.237912934
ENSGALG00010014077	0.912144876	0.424814771
ENSGALG00010014078	0.049403311	0.0583384
ENSGALG00010014080	0.964373872	0.322910535
ENSGALG00010014082	0.235422516	0.209705908
ENSGALG00010014083	0.979981812	0.297224745
ENSGALG00010014084	0.486568715	0.11129954
ENSGALG00010014087	0.082904719	0.393537098
ENSGALG00010014088	0.474269956	0.285754823
ENSGALG00010014089	0.687642386	0.183802503
ENSGALG00010014091	0.853032489	0.563795065
ENSGALG00010014093	0.961868741	0.29542091
ENSGALG00010014096	0.870770821	0.261078592
ENSGALG00010014097	0.745898831	0.397316154
ENSGALG00010014098	0.615773939	0.24853195
ENSGALG00010014099	0.791067711	0.262161769
ENSGALG00010014100	0.151370447	0.54048483
ENSGALG00010014101	0.324552403	0.130497611
ENSGALG00010014102	0.909653782	0.198977341
ENSGALG00010014103	0.566182883	0.14509737
ENSGALG00010014104	0.92087764	0.347416547
ENSGALG00010014105	0.920546662	0.200616127
ENSGALG00010014106	0.727569307	0.221382857
ENSGALG00010014107	0.954413084	0.330941588
ENSGALG00010014108	0.21070135	0.264960944
ENSGALG00010014109	0.814157166	0.032955816
ENSGALG00010014111	0.674965418	0.232913318
ENSGALG00010014113	0.168472759	0.276926583
ENSGALG00010014114	0.220304737	0.409433552
ENSGALG00010014116	0.266137114	0.107784983
ENSGALG00010014117	0.457016091	0.246692557
ENSGALG00010014118	0.146948216	0.14253776
ENSGALG00010014119	0.380566514	0.146304741
ENSGALG00010014120	0.938455334	0.264884559
ENSGALG00010014121	0.257273775	0.161918836
ENSGALG00010014122	0.980764263	0.399191633
ENSGALG00010014123	0.208550923	0.676301294
ENSGALG00010014124	0.451725267	0.106305896
ENSGALG00010014125	0.63957699	0.277376339
ENSGALG00010014126	0.189240572	0.04996112
ENSGALG00010014128	0.896116636	0.468248265
ENSGALG00010014129	0.84478614	0.388149137
ENSGALG00010014131	0.091145172	0.310694237

ENSGALG00010014134	0.617873611	0.026101576
ENSGALG00010014135	0.821590185	0.100826204
ENSGALG00010014138	0.557737279	0.097429904
ENSGALG00010014141	0.949419029	0.277179541
ENSGALG00010014142	0.522487296	0.192957245
ENSGALG00010014143	0.877109859	0.218609649
ENSGALG00010014144	0.340801247	0.000665337
ENSGALG00010014145	0.986724161	0.297727634
ENSGALG00010014146	0.584614581	0.193877131
ENSGALG00010014147	0.958821433	0.417788539
ENSGALG00010014148	0.08243109	0.117800123
ENSGALG00010014149	0.739038418	0.2799028
ENSGALG00010014151	0.108712892	0.093423115
ENSGALG00010014152	0.105365755	0.060065943
ENSGALG00010014153	0.968584081	0.259364173
ENSGALG00010014154	0.269247659	0.33106059
ENSGALG00010014156	0.964359668	0.419783935
ENSGALG00010014159	0.970808861	0.261693794
ENSGALG00010014160	0.576896426	0.190754842
ENSGALG00010014161	0.992778555	0.283577451
ENSGALG00010014162	0.661729179	0.535101888
ENSGALG00010014164	0.594777423	0.066226025
ENSGALG00010014165	0.88255285	0.055622578
ENSGALG00010014166	0.880793116	0.392428155
ENSGALG00010014167	0.881186804	0.399774057
ENSGALG00010014168	0.050564	0.120729262
ENSGALG00010014170	0.956623469	0.315571086
ENSGALG00010014171	0.370605838	0.107055239
ENSGALG00010014173	0.790019311	0.145714742
ENSGALG00010014174	0.447884878	0.204822895
ENSGALG00010014175	0.09541223	0.193385441
ENSGALG00010014176	0.737131464	0.296292271
ENSGALG00010014178	0.165105364	0.035305462
ENSGALG00010014179	0.943586691	0.43165578
ENSGALG00010014180	0.865670912	0.239276496
ENSGALG00010014183	0.512682274	0.14693857
ENSGALG00010014184	0.780980347	0.090639473
ENSGALG00010014185	0.187514554	0.108479243
ENSGALG00010014186	0.913151264	0.281820651
ENSGALG00010014187	0.844393789	0.284301402
ENSGALG00010014189	0.843055702	0.298346242
ENSGALG00010014190	0.51980741	0.137516637
ENSGALG00010014192	0.962181147	0.438443769
ENSGALG00010014193	0.925231103	0.315364637
ENSGALG00010014194	0.905870236	0.377915412
ENSGALG00010014197	0.400115622	0.5833757
ENSGALG00010014198	0.916736894	0.349009706
ENSGALG00010014199	0.549197148	0.230735307
ENSGALG00010014200	0.925070735	0.380054917
ENSGALG00010014201	0.37826817	0.194088878
ENSGALG00010014203	0.781193573	0.274152156
ENSGALG00010014204	0.936344789	0.328911778
ENSGALG00010014205	0.817195334	0.436565329
ENSGALG00010014207	0.013536418	0.091863354
ENSGALG00010014208	0.152894494	0.161114014
ENSGALG00010014209	0.933144695	0.268752591

ENSGALG00010014210	0.778199793	0.173762651
ENSGALG00010014211	0.000308962	0.089704914
ENSGALG00010014212	0.445249393	0.16502204
ENSGALG00010014213	0.013869109	0.267352086
ENSGALG00010014214	0.887792879	0.397875443
ENSGALG00010014217	0.951458977	0.346064008
ENSGALG00010014218	0.961446345	0.26918267
ENSGALG00010014219	0.769405642	0.395876376
ENSGALG00010014220	0.737659476	0.12372338
ENSGALG00010014221	0.977112101	0.300001146
ENSGALG00010014222	0.587330449	0.233110691
ENSGALG00010014223	0.021308589	0.164876077
ENSGALG00010014225	0.957325305	0.275758221
ENSGALG00010014226	0.717272205	0.229440539
ENSGALG00010014227	0.402335153	0.130211642
ENSGALG00010014228	0.770149738	0.546198356
ENSGALG00010014231	0.389527696	0.134775807
ENSGALG00010014234	0.109585598	0.034886346
ENSGALG00010014236	0.919941272	0.372958552
ENSGALG00010014238	0.907296712	0.221660232
ENSGALG00010014239	0.659651963	0.320015725
ENSGALG00010014240	0.834083122	0.335733246
ENSGALG00010014241	0.331557977	0.134316672
ENSGALG00010014242	0.33395213	0.127251842
ENSGALG00010014243	0.878712731	0.367873513
ENSGALG00010014244	0.164622994	0.009982049
ENSGALG00010014246	0.954666221	0.245965748
ENSGALG00010014247	0.635555926	0.584670794
ENSGALG00010014249	0.263659311	0.298696155
ENSGALG00010014250	0.987016593	0.337985079
ENSGALG00010014251	0.924176335	0.245894669
ENSGALG00010014252	0.303942447	0.152546132
ENSGALG00010014253	0.213479858	0.113179908
ENSGALG00010014254	0.627119917	0.171454281
ENSGALG00010014255	0.262922434	0.138601464
ENSGALG00010014256	0.383574131	0.156814206
ENSGALG00010014257	0.789860609	0.40825721
ENSGALG00010014258	0.899722258	0.402228758
ENSGALG00010014259	0.575725752	0.196837869
ENSGALG00010014260	0.904945394	0.400654841
ENSGALG00010014262	0.05369475	0.27839414
ENSGALG00010014263	0.856540233	0.303201577
ENSGALG00010014264	0.317468071	0.452961925
ENSGALG00010014265	0.947633081	0.391406068
ENSGALG00010014267	0.96931838	0.240713582
ENSGALG00010014268	0.274586994	0.115798603
ENSGALG00010014269	0.62992468	0.087690192
ENSGALG00010014272	0.954349378	0.398697284
ENSGALG00010014274	0.970370297	0.274766279
ENSGALG00010014275	0.379098883	0.108694718
ENSGALG00010014278	0.94688458	0.22475319
ENSGALG00010014279	0.216433634	0.680598293
ENSGALG00010014280	0.092255142	0.172283884
ENSGALG00010014281	0.265558089	0.028821141
ENSGALG00010014284	0.940677266	0.351176289
ENSGALG00010014288	0.106461845	0.069042996

ENSGALG00010014289	0.189240572	0.04996112
ENSGALG00010014291	0.353836011	0.142492185
ENSGALG00010014292	0.989748756	0.36309724
ENSGALG00010014293	0.730426667	0.25080918
ENSGALG00010014294	0.963037256	0.207074525
ENSGALG00010014295	0.648783981	0.135770435
ENSGALG00010014296	0.271701261	0.072417625
ENSGALG00010014298	0.984133585	0.346870309
ENSGALG00010014299	0.357294364	0.231549923
ENSGALG00010014300	0.201228432	0.47630071
ENSGALG00010014301	0.014909801	0.409709874
ENSGALG00010014302	0.914311986	0.345610592
ENSGALG00010014303	0.664158336	0.018751445
ENSGALG00010014305	0.927879008	0.365212205
ENSGALG00010014306	0.330774772	0.071450258
ENSGALG00010014307	0.475286975	0.718054333
ENSGALG00010014308	0.988357455	0.335865016
ENSGALG00010014309	0.186983517	0.258736575
ENSGALG00010014310	0.902787881	0.431506485
ENSGALG00010014312	0.869043945	0.299173039
ENSGALG00010014313	0.405253286	0.198417883
ENSGALG00010014314	0.612145091	0.102158057
ENSGALG00010014315	0.568431257	0.164095031
ENSGALG00010014317	0.949837322	0.252640046
ENSGALG00010014318	0.500756293	0.166557333
ENSGALG00010014319	0.088502897	0.082693322
ENSGALG00010014321	0.43802847	0.100203833
ENSGALG00010014322	0.575960811	0.16622708
ENSGALG00010014323	0.052476357	0.012009645
ENSGALG00010014324	0.93920864	0.436807918
ENSGALG00010014325	0.993698749	0.293952253
ENSGALG00010014328	0.003244989	0.058799645
ENSGALG00010014329	0.825905283	0.273980789
ENSGALG00010014330	0.191008478	0.35294258
ENSGALG00010014331	0.11820192	0.376084061
ENSGALG00010014332	0.488197751	0.067780538
ENSGALG00010014334	0.99460707	0.309437398
ENSGALG00010014338	0.901848899	0.295384369
ENSGALG00010014340	0.988121187	0.371161853
ENSGALG00010014344	0.764875859	0.001268942
ENSGALG00010014345	0.211938898	0.202044544
ENSGALG00010014346	0.951332065	0.252267748
ENSGALG00010014347	0.924332302	0.281181817
ENSGALG00010014349	0.851164938	0.495144126
ENSGALG00010014350	0.860346665	0.194272472
ENSGALG00010014353	0.053932482	0.023179231
ENSGALG00010014354	0.38867361	0.120775313
ENSGALG00010014355	0.889447152	0.279733017
ENSGALG00010014357	0.646546643	0.179753833
ENSGALG00010014358	0.841619722	0.266417516
ENSGALG00010014359	0.367039333	0.024038032
ENSGALG00010014360	0.275889746	0.27880894
ENSGALG00010014361	0.989573676	0.341553348
ENSGALG00010014362	0.86970055	0.26989594
ENSGALG00010014363	0.404461533	0.134346384
ENSGALG00010014365	0.011589493	0.110944875

ENSGALG00010014366	0.399597297	0.32533074
ENSGALG00010014367	0.67631596	0.401993513
ENSGALG00010014371	0.680958646	0.294541161
ENSGALG00010014372	0.178487054	0.312532566
ENSGALG00010014373	0.068328869	0.299916391
ENSGALG00010014374	0.544430491	0.223906011
ENSGALG00010014375	0.93703569	0.337447497
ENSGALG00010014376	0.104955345	0.220055655
ENSGALG00010014379	0.952410002	0.390769754
ENSGALG00010014380	0.988647081	0.306347345
ENSGALG00010014381	0.465409998	0.152535495
ENSGALG00010014385	0.003593231	0.494852331
ENSGALG00010014387	0.310769897	0.373922399
ENSGALG00010014388	0.849559483	0.182371452
ENSGALG00010014389	0.694398428	0.430816154
ENSGALG00010014390	0.946568283	0.402175398
ENSGALG00010014393	0.949943836	0.323147382
ENSGALG00010014394	0.476036995	0.156947873
ENSGALG00010014396	0.52898294	0.016771284
ENSGALG00010014397	0.973726576	0.3664003
ENSGALG00010014398	0.97540814	0.263116461
ENSGALG00010014399	0.772463038	0.37114427
ENSGALG00010014400	0.965038103	0.235683204
ENSGALG00010014401	0.980682636	0.265945461
ENSGALG00010014403	0.648110821	0.150923233
ENSGALG00010014406	0.9740495	0.269355597
ENSGALG00010014407	0.232924711	0.221149552
ENSGALG00010014408	0.984911821	0.29308878
ENSGALG00010014410	0.056449346	0.052133601
ENSGALG00010014411	0.970605298	0.311393509
ENSGALG00010014413	0.415779243	0.262119767
ENSGALG00010014414	0.324514485	0.031261924
ENSGALG00010014415	0.480086695	0.200222048
ENSGALG00010014416	0.770535743	0.372678013
ENSGALG00010014418	0.934766045	0.261910205
ENSGALG00010014419	0.382958892	0.167925233
ENSGALG00010014423	0.725029386	0.261962815
ENSGALG00010014424	0.020656918	0.064774402
ENSGALG00010014425	0.380102233	0.015133334
ENSGALG00010014426	0.753949693	0.313799331
ENSGALG00010014427	0.867019529	0.256904307
ENSGALG00010014428	0.634319867	0.218725164
ENSGALG00010014429	0.980394919	0.22999007
ENSGALG00010014430	0.656722601	0.376908199
ENSGALG00010014431	0.228162281	0.172242359
ENSGALG00010014432	0.702762265	0.138754123
ENSGALG00010014434	0.333124356	0.233734168
ENSGALG00010014436	0.387473629	0.117811347
ENSGALG00010014439	0.022074453	0.027483581
ENSGALG00010014441	0.176677727	0.092693733
ENSGALG00010014442	0.018301635	0.519548151
ENSGALG00010014444	0.122979657	0.005488898
ENSGALG00010014448	0.446580205	0.105074988
ENSGALG00010014449	0.884514376	0.186555269
ENSGALG00010014451	0.441900415	0.170592338
ENSGALG00010014453	0.993802687	0.294856495

ENSGALG00010014454	0.377140458	0.079224336
ENSGALG00010014455	0.473954511	0.248185324
ENSGALG00010014459	0.578128305	0.067072938
ENSGALG00010014462	0.611931155	0.180370296
ENSGALG00010014464	0.076380867	0.061149184
ENSGALG00010014465	0.979565558	0.305045343
ENSGALG00010014466	0.724960639	0.10519068
ENSGALG00010014468	0.036547844	0.303156115
ENSGALG00010014469	0.786807711	0.186043448
ENSGALG00010014470	0.981644336	0.311137128
ENSGALG00010014472	0.372162866	0.080568109
ENSGALG00010014474	0.985129785	0.363321054
ENSGALG00010014476	0.853918996	0.383621823
ENSGALG00010014477	0.980814425	0.293174186
ENSGALG00010014478	0.305953318	0.250597651
ENSGALG00010014480	0.991803269	0.292721305
ENSGALG00010014482	0.125247515	0.17037319
ENSGALG00010014484	0.96314534	0.262304787
ENSGALG00010014485	0.40135901	0.158038591
ENSGALG00010014487	0.959370084	0.324310041
ENSGALG00010014488	0.858419385	0.170752646
ENSGALG00010014490	0.358759145	0.183668006
ENSGALG00010014492	0.635700758	0.176160876
ENSGALG00010014493	0.521333825	0.343906194
ENSGALG00010014494	0.810522696	0.403035462
ENSGALG00010014495	0.872390426	0.393838422
ENSGALG00010014496	0.07518309	0.066451201
ENSGALG00010014497	0.050668086	0.013878872
ENSGALG00010014500	0.323738128	0.135328488
ENSGALG00010014504	0.86817014	0.26297612
ENSGALG00010014505	0.98070355	0.405706187
ENSGALG00010014507	0.313618903	0.186906495
ENSGALG00010014509	0.068903434	0.166940722
ENSGALG00010014512	0.870454241	0.242995584
ENSGALG00010014513	0.394494248	0.128304162
ENSGALG00010014515	0.960504606	0.313414703
ENSGALG00010014517	0.968734559	0.234333294
ENSGALG00010014522	0.810456986	0.316498115
ENSGALG00010014523	0.491102471	0.199717228
ENSGALG00010014525	0.282754916	0.323313762
ENSGALG00010014527	0.490281073	0.235545194
ENSGALG00010014529	0.535359982	0.074383053
ENSGALG00010014532	0.125498244	0.090447055
ENSGALG00010014533	0.442480559	0.182338764
ENSGALG00010014534	0.265558089	0.028821141
ENSGALG00010014536	0.363876531	0.1141785
ENSGALG00010014537	0.044012277	0.345497229
ENSGALG00010014538	0.37751878	0.23251672
ENSGALG00010014539	0.906364589	0.267330191
ENSGALG00010014540	0.996002559	0.336007033
ENSGALG00010014542	0.3910821	0.072487727
ENSGALG00010014543	0.037014007	0.170276634
ENSGALG00010014545	0.956731427	0.336394333
ENSGALG00010014547	0.97083018	0.294567664
ENSGALG00010014548	0.406469659	0.441870834
ENSGALG00010014550	0.500455472	0.188083547

ENSGALG00010014551	0.386254376	0.161451439
ENSGALG00010014553	0.641509342	0.083209536
ENSGALG00010014555	0.285115707	0.117813521
ENSGALG00010014558	0.824940193	0.225421532
ENSGALG00010014559	0.635308243	0.1357398
ENSGALG00010014562	0.878025086	0.250355146
ENSGALG00010014563	0.008565402	0.00863032
ENSGALG00010014564	0.77309401	0.284896773
ENSGALG00010014565	0.870073117	0.463884784
ENSGALG00010014567	0.683596862	0.27575507
ENSGALG00010014568	0.392964283	0.330603017
ENSGALG00010014569	0.746260639	0.213044024
ENSGALG00010014570	0.368756523	0.218214731
ENSGALG00010014571	0.954808176	0.367495287
ENSGALG00010014572	0.980364376	0.265303838
ENSGALG00010014573	0.536884584	0.228002413
ENSGALG00010014574	0.632003304	0.342206019
ENSGALG00010014575	0.365962212	0.234393377
ENSGALG00010014576	0.642168443	0.276985531
ENSGALG00010014577	0.346384801	0.248568751
ENSGALG00010014578	0.804972152	0.269744517
ENSGALG00010014579	0.944210848	0.266030825
ENSGALG00010014580	0.240341266	0.118990471
ENSGALG00010014582	0.659598408	0.617698859
ENSGALG00010014583	0.38813597	0.156768404
ENSGALG00010014585	0.981311199	0.310509023
ENSGALG00010014586	0.39259554	0.208198583
ENSGALG00010014587	0.756655543	0.221230863
ENSGALG00010014588	0.867848348	0.352834869
ENSGALG00010014591	0.929367777	0.315720909
ENSGALG00010014592	0.171875219	0.098010428
ENSGALG00010014598	0.798860406	0.281709276
ENSGALG00010014599	0.766258259	0.431818278
ENSGALG00010014600	0.745557987	0.210201081
ENSGALG00010014602	0.991149649	0.292550668
ENSGALG00010014603	0.596683684	0.270641028
ENSGALG00010014607	0.697111205	0.293577256
ENSGALG00010014611	0.703428802	0.429553039
ENSGALG00010014613	0.160873349	0.314690278
ENSGALG00010014614	0.932574482	0.316392247
ENSGALG00010014615	0.409486278	0.157951038
ENSGALG00010014617	0.746031738	0.101458967
ENSGALG00010014619	0.386050996	0.14466628
ENSGALG00010014623	0.531592418	0.005834432
ENSGALG00010014624	0.56839131	0.237265762
ENSGALG00010014625	0.891679722	0.409180736
ENSGALG00010014626	0.064286412	0.405761582
ENSGALG00010014627	0.285272007	0.109169955
ENSGALG00010014629	0.089765396	0.095692089
ENSGALG00010014630	0.202954004	0.527525171
ENSGALG00010014631	0.372997723	0.16116762
ENSGALG00010014632	0.524352281	0.266500789
ENSGALG00010014633	0.477518871	0.28890509
ENSGALG00010014634	0.989708925	0.281992927
ENSGALG00010014635	0.355008442	0.209211752
ENSGALG00010014636	0.025879769	0.000753611

ENSGALG00010014638	0.364055186	0.42690201
ENSGALG00010014639	0.265558089	0.028821141
ENSGALG00010014640	0.364233449	0.112130852
ENSGALG00010014641	0.367223028	0.229517479
ENSGALG00010014642	0.939422195	0.309370397
ENSGALG00010014644	0.540244583	0.116718164
ENSGALG00010014645	0.700920095	0.190841626
ENSGALG00010014647	0.369875003	0.291167377
ENSGALG00010014648	0.896159941	0.291825055
ENSGALG00010014649	0.222381971	0.242489937
ENSGALG00010014651	0.060053542	0.273736993
ENSGALG00010014652	0.938374603	0.300047237
ENSGALG00010014653	0.959462248	0.334524713
ENSGALG00010014654	0.783690044	0.269577391
ENSGALG00010014655	0.937047783	0.311774554
ENSGALG00010014656	0.732425882	0.310902332
ENSGALG00010014657	0.470349358	0.303602487
ENSGALG00010014658	0.990557478	0.294658185
ENSGALG00010014661	0.040462525	0.140214121
ENSGALG00010014664	0.082219616	0.123558789
ENSGALG00010014666	0.355404278	0.180504476
ENSGALG00010014667	0.94839342	0.220200887
ENSGALG00010014668	0.952748784	0.298690014
ENSGALG00010014669	0.390418234	0.123263498
ENSGALG00010014673	0.857823266	0.354289945
ENSGALG00010014674	0.358187506	0.245502378
ENSGALG00010014675	0.186893153	0.150171274
ENSGALG00010014676	0.906750296	0.205102349
ENSGALG00010014677	0.800049918	0.117111362
ENSGALG00010014680	0.429073832	0.284542873
ENSGALG00010014681	0.68609793	0.228534298
ENSGALG00010014682	0.846205277	0.277646267
ENSGALG00010014683	0.361470737	0.15237379
ENSGALG00010014684	0.442695998	0.373553176
ENSGALG00010014685	0.518521159	0.262309343
ENSGALG00010014687	0.938126398	0.279838827
ENSGALG00010014690	0.901426456	0.324620506
ENSGALG00010014691	0.383294577	0.171398002
ENSGALG00010014692	0.349203243	0.141617585
ENSGALG00010014693	0.203991683	0.058221925
ENSGALG00010014694	0.579295337	0.190118872
ENSGALG00010014696	0.996625715	0.350970003
ENSGALG00010014697	0.766217883	0.153747099
ENSGALG00010014698	0.166682034	0.069006121
ENSGALG00010014700	0.123027899	0.152780433
ENSGALG00010014702	0.678665924	0.065673069
ENSGALG00010014703	0.946703232	0.453126133
ENSGALG00010014704	0.130032832	0.019391731
ENSGALG00010014705	0.898767376	0.358440479
ENSGALG00010014707	0.949130167	0.259514767
ENSGALG00010014708	0.292709036	0.327963953
ENSGALG00010014710	0.993217893	0.354475705
ENSGALG00010014712	0.271701261	0.072417625
ENSGALG00010014713	0.368451542	0.026399899
ENSGALG00010014716	0.676994974	0.467247287
ENSGALG00010014717	0.475672252	0.381943347

ENSGALG00010014721	0.396808512	0.241687704
ENSGALG00010014722	0.960322897	0.2911526
ENSGALG00010014723	0.748674459	0.569598891
ENSGALG00010014724	0.74995786	0.260758407
ENSGALG00010014725	0.433660462	0.058736438
ENSGALG00010014726	0.937297448	0.329744898
ENSGALG00010014727	0.682750773	0.004726109
ENSGALG00010014728	0.986107727	0.307493755
ENSGALG00010014729	0.97636377	0.312472722
ENSGALG00010014730	0.38742626	0.154147022
ENSGALG00010014731	0.525034135	0.220204673
ENSGALG00010014732	0.411129067	0.215685987
ENSGALG00010014734	0.866335541	0.341798175
ENSGALG00010014735	0.909939041	0.340026117
ENSGALG00010014736	0.633488395	0.178445293
ENSGALG00010014737	0.722205298	0.428913169
ENSGALG00010014738	0.782507974	0.513294119
ENSGALG00010014739	0.961795642	0.324112287
ENSGALG00010014740	0.506400784	0.212008755
ENSGALG00010014742	0.988526627	0.383374962
ENSGALG00010014743	0.865350312	0.312409449
ENSGALG00010014744	0.933726083	0.246617469
ENSGALG00010014745	0.864870416	0.308539103
ENSGALG00010014746	0.389333388	0.090852028
ENSGALG00010014747	0.361814957	0.21853291
ENSGALG00010014748	0.96748513	0.322024065
ENSGALG00010014750	0.110726559	0.038810103
ENSGALG00010014751	0.287100517	0.099799561
ENSGALG00010014753	0.264857785	0.18590028
ENSGALG00010014754	0.694997882	0.291219849
ENSGALG00010014755	0.422849039	0.117723675
ENSGALG00010014756	0.478335955	0.142604859
ENSGALG00010014758	0.069545882	0.039778921
ENSGALG00010014759	0.628545439	0.256933148
ENSGALG00010014760	0.593780534	0.06959194
ENSGALG00010014762	0.702962182	0.018667778
ENSGALG00010014764	0.994966593	0.334373461
ENSGALG00010014766	0.974317611	0.298087474
ENSGALG00010014767	0.361229439	0.172843665
ENSGALG00010014768	0.36076785	0.482483507
ENSGALG00010014769	0.735203885	0.242562302
ENSGALG00010014771	0.551270423	0.285599955
ENSGALG00010014772	0.953272397	0.320081581
ENSGALG00010014773	0.574852109	0.185447775
ENSGALG00010014776	0.510225572	0.272066764
ENSGALG00010014777	0.086234425	0.374964954
ENSGALG00010014778	0.997059166	0.334911133
ENSGALG00010014780	0.556985377	0.242025362
ENSGALG00010014782	0.584445699	0.000870181
ENSGALG00010014783	0.932164807	0.138922071
ENSGALG00010014785	0.301600478	0.303177417
ENSGALG00010014788	0.407656186	0.076012416
ENSGALG00010014789	0.99232468	0.274988159
ENSGALG00010014792	0.904638107	0.319367002
ENSGALG00010014793	0.960997449	0.236372901
ENSGALG00010014795	0.386381878	0.531713305

ENSGALG00010014796	0.767164083	0.285157942
ENSGALG00010014797	0.954436584	0.365799348
ENSGALG00010014801	0.886505166	0.203811305
ENSGALG00010014802	0.787045903	0.274499616
ENSGALG00010014805	0.881333907	0.321961364
ENSGALG00010014806	0.995118453	0.320468635
ENSGALG00010014807	0.976343369	0.277548862
ENSGALG00010014809	0.400441604	0.007124268
ENSGALG00010014810	0.911448042	0.367908423
ENSGALG00010014811	0.914822073	0.505447103
ENSGALG00010014812	0.972465851	0.269125849
ENSGALG00010014813	0.912389297	0.2605677
ENSGALG00010014815	0.208550923	0.676301294
ENSGALG00010014816	0.909067689	0.311936006
ENSGALG00010014817	0.571550444	0.153396742
ENSGALG00010014818	0.005463639	0.442063768
ENSGALG00010014819	0.658282855	0.352245294
ENSGALG00010014820	0.453135293	0.022781597
ENSGALG00010014821	0.983551017	0.313339706
ENSGALG00010014822	0.573087024	0.304528055
ENSGALG00010014823	0.334408962	0.154015172
ENSGALG00010014824	0.829016668	0.347014892
ENSGALG00010014825	0.809143342	0.272761567
ENSGALG00010014826	0.838503321	0.204451304
ENSGALG00010014827	0.676583249	0.205369359
ENSGALG00010014828	0.055966411	0.152701962
ENSGALG00010014829	0.88259689	0.378641942
ENSGALG00010014830	0.549519592	0.596191278
ENSGALG00010014831	0.895886008	0.293947862
ENSGALG00010014832	0.218098117	0.159922573
ENSGALG00010014833	0.275160291	0.227691137
ENSGALG00010014834	0.712638295	0.17796094
ENSGALG00010014835	0.478320512	0.178306522
ENSGALG00010014836	0.691134718	0.031267806
ENSGALG00010014837	0.529681126	0.218384927
ENSGALG00010014838	0.417434606	0.118622464
ENSGALG00010014839	0.771769145	0.062292587
ENSGALG00010014840	0.944924304	0.184736901
ENSGALG00010014844	0.393807095	0.368308303
ENSGALG00010014845	0.14242522	0.328668018
ENSGALG00010014846	0.329744371	0.371370966
ENSGALG00010014847	0.980205916	0.341884035
ENSGALG00010014851	0.451440829	0.185296104
ENSGALG00010014852	0.171988536	0.357029771
ENSGALG00010014853	0.365421542	0.060491169
ENSGALG00010014854	0.265558089	0.028821141
ENSGALG00010014856	0.939968632	0.31502278
ENSGALG00010014857	0.4686264	0.113623448
ENSGALG00010014859	0.955315621	0.364230082
ENSGALG00010014860	0.287538582	0.122952094
ENSGALG00010014861	0.783064451	0.096771875
ENSGALG00010014862	0.026695066	0.171844461
ENSGALG00010014864	0.764318006	0.308794211
ENSGALG00010014865	0.981941307	0.311771154
ENSGALG00010014866	0.936418256	0.431416916
ENSGALG00010014867	0.720329554	0.171406193

ENSGALG00010014868	0.684745878	0.492883633
ENSGALG00010014870	0.409436406	0.157934013
ENSGALG00010014871	0.732123297	0.276792609
ENSGALG00010014873	0.720859809	0.334197431
ENSGALG00010014875	0.636912574	0.149124911
ENSGALG00010014876	0.234371858	0.022223252
ENSGALG00010014877	0.29921203	0.437704604
ENSGALG00010014878	0.765696645	0.274551503
ENSGALG00010014879	0.753945776	0.225548648
ENSGALG00010014880	0.681995219	0.530805037
ENSGALG00010014881	0.160800845	0.258228505
ENSGALG00010014882	0.972559554	0.284970602
ENSGALG00010014883	0.987185997	0.327756501
ENSGALG00010014886	0.897971933	0.263926436
ENSGALG00010014887	0.826017072	0.193828292
ENSGALG00010014888	0.512207956	0.2308252
ENSGALG00010014889	0.894622326	0.201599841
ENSGALG00010014890	0.088758412	0.165463429
ENSGALG00010014891	0.5700742	0.437182807
ENSGALG00010014892	0.769067582	0.196498335
ENSGALG00010014893	0.433571854	0.035498978
ENSGALG00010014894	0.193845926	0.218617081
ENSGALG00010014895	0.317468071	0.452961925
ENSGALG00010014897	0.877268957	0.42428726
ENSGALG00010014898	0.45869831	0.028895944
ENSGALG00010014899	0.14361461	0.484383905
ENSGALG00010014900	0.284249958	0.098679062
ENSGALG00010014902	0.808665621	0.239730207
ENSGALG00010014903	0.976451956	0.298361872
ENSGALG00010014904	0.200570274	0.091667991
ENSGALG00010014905	0.425508884	0.133407704
ENSGALG00010014906	0.193451095	0.215576658
ENSGALG00010014907	0.201321102	0.260528004
ENSGALG00010014908	0.933792214	0.301424288
ENSGALG00010014909	0.13637447	0.160729063
ENSGALG00010014910	0.450713864	0.178537055
ENSGALG00010014911	0.685962652	0.202788383
ENSGALG00010014912	0.689411816	0.588340984
ENSGALG00010014914	0.775695027	0.143254686
ENSGALG00010014915	0.086657171	0.03213558
ENSGALG00010014916	0.685046671	0.098189513
ENSGALG00010014917	0.947661054	0.30461301
ENSGALG00010014918	0.561513854	0.094786986
ENSGALG00010014919	0.364797438	0.061749059
ENSGALG00010014922	0.992825492	0.315495979
ENSGALG00010014923	0.049550984	0.122051033
ENSGALG00010014927	0.118658777	0.09508325
ENSGALG00010014928	0.846737357	0.306987923
ENSGALG00010014929	0.913162588	0.39148912
ENSGALG00010014930	0.378848707	0.137590461
ENSGALG00010014931	0.591131253	0.31169671
ENSGALG00010014934	0.93926461	0.319371445
ENSGALG00010014935	0.960622468	0.332105829
ENSGALG00010014936	0.196837525	0.086885519
ENSGALG00010014940	0.857150792	0.267563714
ENSGALG00010014941	0.584569341	0.047825298

ENSGALG00010014942	0.316934322	0.251382524
ENSGALG00010014943	0.80048352	0.334615837
ENSGALG00010014944	0.994471688	0.333204125
ENSGALG00010014945	0.488264621	0.167554431
ENSGALG00010014946	0.985343787	0.327120634
ENSGALG00010014948	0.973854048	0.326578763
ENSGALG00010014949	0.814144107	0.092991835
ENSGALG00010014951	0.015759578	0.118247465
ENSGALG00010014952	0.470755615	0.128493623
ENSGALG00010014953	0.886348015	0.411934238
ENSGALG00010014954	0.711872939	0.507681448
ENSGALG00010014956	0.994534064	0.345685138
ENSGALG00010014957	0.606068811	0.268934799
ENSGALG00010014958	0.381472652	0.117237179
ENSGALG00010014963	0.992160017	0.355097787
ENSGALG00010014965	0.907695434	0.463692032
ENSGALG00010014967	0.265786385	0.085111598
ENSGALG00010014970	0.982292107	0.278504743
ENSGALG00010014971	0.980473249	0.347698225
ENSGALG00010014972	0.996910991	0.338108252
ENSGALG00010014973	0.653599692	0.195715736
ENSGALG00010014974	0.871307973	0.404825033
ENSGALG00010014976	0.976840074	0.324437192
ENSGALG00010014977	0.285272007	0.109169955
ENSGALG00010014978	0.719379259	0.127026558
ENSGALG00010014979	0.285272007	0.109169955
ENSGALG00010014980	0.907780345	0.54788841
ENSGALG00010014981	0.363002822	0.184244517
ENSGALG00010014982	0.670821885	0.016583514
ENSGALG00010014983	0.065910046	0.322413415
ENSGALG00010014984	0.390182534	0.290651872
ENSGALG00010014985	0.882697671	0.318843855
ENSGALG00010014987	0.250376248	0.231572513
ENSGALG00010014988	0.13608142	0.124976599
ENSGALG00010014989	0.959275618	0.303438867
ENSGALG00010014990	0.463122908	0.240845612
ENSGALG00010014991	0.544530773	0.482167624
ENSGALG00010014992	0.451105651	0.458515103
ENSGALG00010014994	0.55468594	0.056413373
ENSGALG00010014995	0.972323552	0.253771933
ENSGALG00010014996	0.099823104	0.029850532
ENSGALG00010014997	0.753943112	0.34061151
ENSGALG00010014998	0.366289438	0.247197773
ENSGALG00010014999	0.211752083	0.171001832
ENSGALG00010015002	0.429269608	0.383107829
ENSGALG00010015006	0.991088041	0.331657563
ENSGALG00010015008	0.604623513	0.200749961
ENSGALG00010015009	0.634945195	0.259144782
ENSGALG00010015010	0.575190928	0.142105877
ENSGALG00010015013	0.895290816	0.267411827
ENSGALG00010015015	0.499546691	0.179313888
ENSGALG00010015016	0.253154897	0.262820722
ENSGALG00010015017	0.850461995	0.232972035
ENSGALG00010015018	0.31704587	0.011957074
ENSGALG00010015019	0.270451009	0.090541667
ENSGALG00010015020	0.991631851	0.349247228

ENSGALG00010015021	0.762971684	0.276424456
ENSGALG00010015023	0.60866963	0.004769025
ENSGALG00010015024	0.767317115	0.162894613
ENSGALG00010015025	0.250668281	0.147012492
ENSGALG00010015026	0.361476659	0.150924776
ENSGALG00010015027	0.604578943	0.241704694
ENSGALG00010015028	0.152480934	0.264102628
ENSGALG00010015029	0.594720883	0.023318381
ENSGALG00010015030	0.967578268	0.314785696
ENSGALG00010015031	0.871719333	0.217113998
ENSGALG00010015032	0.833412256	0.425099905
ENSGALG00010015033	0.827290992	0.234415656
ENSGALG00010015035	0.614295667	0.292719403
ENSGALG00010015036	0.023279831	0.134374301
ENSGALG00010015037	0.943777048	0.28469214
ENSGALG00010015038	0.943978515	0.276453786
ENSGALG00010015039	0.058960232	0.320684423
ENSGALG00010015040	0.916492438	0.207625843
ENSGALG00010015041	0.001517382	0.279031796
ENSGALG00010015042	0.871843592	0.358608798
ENSGALG00010015043	0.085229685	0.293188335
ENSGALG00010015044	0.896489595	0.336994443
ENSGALG00010015046	0.893715882	0.359476886
ENSGALG00010015047	0.759028937	0.441110989
ENSGALG00010015049	0.316889607	0.014597864
ENSGALG00010015050	0.804780119	0.317281061
ENSGALG00010015052	0.920941488	0.426124519
ENSGALG00010015053	0.405849911	0.06592907
ENSGALG00010015054	0.810320756	0.501783175
ENSGALG00010015055	0.97001458	0.302546961
ENSGALG00010015056	0.651604218	0.239977916
ENSGALG00010015057	0.665446766	0.270863834
ENSGALG00010015058	0.011120243	0.01000129
ENSGALG00010015059	0.932991112	0.316494702
ENSGALG00010015060	0.490749578	0.505630649
ENSGALG00010015061	0.900809662	0.200078186
ENSGALG00010015062	0.864862282	0.215287592
ENSGALG00010015063	0.129242349	0.039732684
ENSGALG00010015064	0.978220003	0.366319616
ENSGALG00010015065	0.992108546	0.35207722
ENSGALG00010015066	0.756448091	0.465331835
ENSGALG00010015067	0.090825598	0.315253713
ENSGALG00010015068	0.525362042	0.50022071
ENSGALG00010015069	0.942906105	0.337725872
ENSGALG00010015070	0.14591649	0.270351184
ENSGALG00010015071	0.525078838	0.109401122
ENSGALG00010015072	0.856090252	0.325129261
ENSGALG00010015074	0.423141104	0.265228141
ENSGALG00010015075	0.995716758	0.356573879
ENSGALG00010015076	0.951532495	0.16641196
ENSGALG00010015077	0.06817362	0.172942874
ENSGALG00010015078	0.482305655	0.182238968
ENSGALG00010015080	0.787598573	0.152383717
ENSGALG00010015082	0.811700897	0.336146163
ENSGALG00010015083	0.866159885	0.298175004
ENSGALG00010015084	0.8424844	0.437426213

ENSGALG00010015085	0.955377923	0.334851541
ENSGALG00010015086	0.071163159	0.150313734
ENSGALG00010015087	0.348012532	0.131070993
ENSGALG00010015088	0.357827325	0.065941713
ENSGALG00010015089	0.187152416	0.121744695
ENSGALG00010015090	0.921114132	0.433619482
ENSGALG00010015091	0.948774978	0.250793146
ENSGALG00010015092	0.914697433	0.305725747
ENSGALG00010015093	0.684200152	0.240927287
ENSGALG00010015094	0.985842875	0.333169352
ENSGALG00010015095	0.985779412	0.324684837
ENSGALG00010015096	0.214146421	0.153398195
ENSGALG00010015097	0.963726049	0.2899543
ENSGALG00010015098	0.978809265	0.301833075
ENSGALG00010015100	0.980538008	0.320813614
ENSGALG00010015101	0.979167486	0.292759928
ENSGALG00010015104	0.547280265	0.343843417
ENSGALG00010015105	0.919494834	0.218639077
ENSGALG00010015106	0.216433634	0.680598293
ENSGALG00010015107	0.78290024	0.27639434
ENSGALG00010015108	0.018598736	0.192380243
ENSGALG00010015109	0.972265856	0.248861484
ENSGALG00010015110	0.10139089	0.052099271
ENSGALG00010015111	0.703302092	0.073545321
ENSGALG00010015112	0.374058465	0.124202176
ENSGALG00010015113	0.195851107	0.167102021
ENSGALG00010015115	0.785135107	0.308803187
ENSGALG00010015116	0.271701261	0.072417625
ENSGALG00010015117	0.360323172	0.336213895
ENSGALG00010015118	0.920389506	0.27519638
ENSGALG00010015119	0.941021775	0.280157811
ENSGALG00010015120	0.24387788	0.217444781
ENSGALG00010015121	0.910821662	0.318296623
ENSGALG00010015122	0.684366221	0.273735386
ENSGALG00010015123	0.943454256	0.293358946
ENSGALG00010015124	0.863051687	0.400174918
ENSGALG00010015125	0.277566769	0.107857127
ENSGALG00010015126	0.507879496	0.126726054
ENSGALG00010015127	0.772938111	0.330304528
ENSGALG00010015128	0.966850799	0.305210072
ENSGALG00010015129	0.715695225	0.385794844
ENSGALG00010015130	0.383512035	0.158206748
ENSGALG00010015131	0.944804632	0.365184331
ENSGALG00010015132	0.948166348	0.320216676
ENSGALG00010015133	0.82617821	0.268090195
ENSGALG00010015134	0.138260956	0.331831533
ENSGALG00010015136	0.805823467	0.169141958
ENSGALG00010015137	0.949765228	0.304021482
ENSGALG00010015138	0.749603387	0.214119843
ENSGALG00010015140	0.512360269	0.084583234
ENSGALG00010015141	0.872600172	0.286037245
ENSGALG00010015142	0.826804852	0.263583169
ENSGALG00010015143	0.180034975	0.112791659
ENSGALG00010015144	0.139851858	0.111583845
ENSGALG00010015145	0.60642909	0.050360364
ENSGALG00010015146	0.406442211	0.009525154

ENSGALG00010015148	0.094583584	0.268771524
ENSGALG00010015149	0.666456187	0.032849515
ENSGALG00010015151	0.015454029	0.11304295
ENSGALG00010015152	0.987098677	0.29984816
ENSGALG00010015153	0.735358445	0.196725841
ENSGALG00010015154	0.024689244	0.135282994
ENSGALG00010015155	0.989771755	0.368811876
ENSGALG00010015156	0.927230125	0.42115168
ENSGALG00010015157	0.979578856	0.327895839
ENSGALG00010015158	0.914322564	0.301499668
ENSGALG00010015159	0.811691561	0.293670633
ENSGALG00010015160	0.714914258	0.222996691
ENSGALG00010015161	0.260122675	0.323257224
ENSGALG00010015162	0.059172591	0.157657381
ENSGALG00010015163	0.70679409	0.104544453
ENSGALG00010015164	0.622563869	0.148589226
ENSGALG00010015165	0.961854358	0.411437044
ENSGALG00010015166	0.954506812	0.328641606
ENSGALG00010015167	0.502071194	0.193675818
ENSGALG00010015169	0.032978828	0.093802223
ENSGALG00010015171	0.840844081	0.339305219
ENSGALG00010015172	0.418938362	0.096991001
ENSGALG00010015173	0.267450208	0.132160098
ENSGALG00010015174	0.614721021	0.369467682
ENSGALG00010015175	0.246821213	0.085267178
ENSGALG00010015177	0.776783554	0.319580895
ENSGALG00010015178	0.208550923	0.676301294
ENSGALG00010015179	0.387312444	0.236087824
ENSGALG00010015180	0.596665191	0.299384133
ENSGALG00010015181	0.959889878	0.414787783
ENSGALG00010015182	0.768907045	0.260135891
ENSGALG00010015183	0.208550923	0.676301294
ENSGALG00010015184	0.614619421	0.238684316
ENSGALG00010015185	0.431466585	0.01856818
ENSGALG00010015186	0.938772863	0.255269629
ENSGALG00010015188	0.996241272	0.336281327
ENSGALG00010015190	0.380267332	0.109092415
ENSGALG00010015191	0.993637404	0.303671611
ENSGALG00010015192	0.746201627	0.39724542
ENSGALG00010015193	0.498028662	0.484182772
ENSGALG00010015194	0.783174544	0.291772698
ENSGALG00010015196	0.952056362	0.347737949
ENSGALG00010015197	0.47648737	0.176555271
ENSGALG00010015198	0.087975919	0.13106331
ENSGALG00010015199	0.787999533	0.056576048
ENSGALG00010015200	0.371848469	0.232484125
ENSGALG00010015201	0.872991517	0.293635651
ENSGALG00010015203	0.93889693	0.472595738
ENSGALG00010015204	0.975897137	0.343894269
ENSGALG00010015205	0.791787263	0.300992741
ENSGALG00010015206	0.705780389	0.259793675
ENSGALG00010015207	0.774999488	0.425385759
ENSGALG00010015208	0.892121546	0.257018095
ENSGALG00010015209	0.220818482	0.205768094
ENSGALG00010015211	0.937919436	0.367982933
ENSGALG00010015212	0.774077153	0.273122689

ENSGALG00010015213	0.690551291	0.153074889
ENSGALG00010015216	0.418122213	0.062084349
ENSGALG00010015217	0.271701261	0.072417625
ENSGALG00010015218	0.672172247	0.252320172
ENSGALG00010015219	0.456104745	0.133499529
ENSGALG00010015220	0.69613153	0.205387576
ENSGALG00010015223	0.412788748	0.349800033
ENSGALG00010015225	0.558134002	0.002120534
ENSGALG00010015226	0.904703035	0.595359929
ENSGALG00010015227	0.931616772	0.250454336
ENSGALG00010015228	0.940762049	0.424675294
ENSGALG00010015232	0.558030867	0.222733875
ENSGALG00010015233	0.339354177	0.176126873
ENSGALG00010015234	0.936946488	0.385935199
ENSGALG00010015239	0.474116977	0.124314476
ENSGALG00010015241	0.039013067	0.082162305
ENSGALG00010015243	0.46508423	0.19455744
ENSGALG00010015245	0.463028763	0.087247183
ENSGALG00010015246	0.132029222	0.406358342
ENSGALG00010015248	0.849422031	0.269187756
ENSGALG00010015249	0.396617227	0.102705405
ENSGALG00010015250	0.048874854	0.177851658
ENSGALG00010015252	0.527983806	0.102422224
ENSGALG00010015253	0.771995446	0.291816026
ENSGALG00010015254	0.303239222	0.029970476
ENSGALG00010015255	0.957854233	0.240046612
ENSGALG00010015257	0.796790073	0.25885759
ENSGALG00010015258	0.666377935	0.102522258
ENSGALG00010015260	0.654594078	0.070193152
ENSGALG00010015261	0.50560779	0.252329751
ENSGALG00010015262	0.114044697	0.189520971
ENSGALG00010015263	0.951690253	0.241135525
ENSGALG00010015264	0.392206929	0.143464443
ENSGALG00010015266	0.440953629	0.143611041
ENSGALG00010015268	0.695754295	0.504310108
ENSGALG00010015272	0.233191245	0.230247574
ENSGALG00010015274	0.24776858	0.152050173
ENSGALG00010015278	0.270451009	0.090541667
ENSGALG00010015279	0.189240572	0.04996112
ENSGALG00010015282	0.340601308	0.064109408
ENSGALG00010015283	0.567861724	0.170361482
ENSGALG00010015285	0.663975443	0.115739647
ENSGALG00010015286	0.225024331	0.108181861
ENSGALG00010015291	0.332549612	0.140256647
ENSGALG00010015292	0.87862238	0.324265981
ENSGALG00010015293	0.287683915	0.813901871
ENSGALG00010015294	0.438006218	0.824299465
ENSGALG00010015295	0.904530018	0.307757536
ENSGALG00010015296	0.482308272	0.2943166
ENSGALG00010015298	0.92938457	0.303617333
ENSGALG00010015299	0.905502281	0.254850634
ENSGALG00010015300	0.937152272	0.30759334
ENSGALG00010015302	0.300479476	0.115860765
ENSGALG00010015303	0.288888201	0.045619874
ENSGALG00010015306	0.394428897	0.395496966
ENSGALG00010015310	0.98659564	0.311163284

ENSGALG00010015311	0.374867962	0.29811939
ENSGALG00010015312	0.445433687	0.060797523
ENSGALG00010015314	0.930388823	0.392231488
ENSGALG00010015315	0.234565587	0.016907675
ENSGALG00010015317	0.123663058	0.029069875
ENSGALG00010015318	0.928454843	0.348654534
ENSGALG00010015319	0.372796048	0.167811456
ENSGALG00010015320	0.458485716	0.154552907
ENSGALG00010015321	0.166398148	0.161280892
ENSGALG00010015322	0.07225596	0.582335108
ENSGALG00010015323	0.350705	0.102583314
ENSGALG00010015325	0.234826202	0.098542803
ENSGALG00010015327	0.932473766	0.39359942
ENSGALG00010015328	0.294527392	0.082168456
ENSGALG00010015329	0.77067115	0.36535249
ENSGALG00010015330	0.215246452	0.39899041
ENSGALG00010015333	0.615148806	0.02000199
ENSGALG00010015334	0.521301754	0.381497611
ENSGALG00010015335	0.378821885	0.065964134
ENSGALG00010015336	0.080223077	0.059688596
ENSGALG00010015337	0.041573802	0.219004385
ENSGALG00010015338	0.88811925	0.263254757
ENSGALG00010015339	0.313104699	0.151578969
ENSGALG00010015343	0.979465251	0.344930578
ENSGALG00010015345	0.44208044	0.146690892
ENSGALG00010015346	0.443372537	0.223310699
ENSGALG00010015347	0.32707355	0.281479387
ENSGALG00010015348	0.931191731	0.269271036
ENSGALG00010015349	0.366810591	0.293049106
ENSGALG00010015350	0.265558089	0.028821141
ENSGALG00010015351	0.205920741	0.227294065
ENSGALG00010015353	0.73073309	0.315067929
ENSGALG00010015356	0.772965765	0.009462731
ENSGALG00010015357	0.385953062	0.133261917
ENSGALG00010015362	0.951482575	0.246736094
ENSGALG00010015364	0.754787621	0.01581206
ENSGALG00010015365	0.095545711	0.195851575
ENSGALG00010015366	0.35194972	0.140777882
ENSGALG00010015368	0.673834264	0.497774557
ENSGALG00010015370	0.980292638	0.293320239
ENSGALG00010015371	0.225950931	0.304109222
ENSGALG00010015372	0.008620651	0.150707699
ENSGALG00010015374	0.246447285	0.071226122
ENSGALG00010015375	0.764522381	0.135937547
ENSGALG00010015376	0.219311648	0.194349778
ENSGALG00010015378	0.37671603	0.150307264
ENSGALG00010015380	0.270451009	0.090541667
ENSGALG00010015382	0.476003345	0.18239726
ENSGALG00010015384	0.970701667	0.24501383
ENSGALG00010015386	0.914670939	0.162956437
ENSGALG00010015387	0.266137114	0.107784983
ENSGALG00010015388	0.501751166	0.237288483
ENSGALG00010015389	0.580082544	0.154539226
ENSGALG00010015390	0.491224668	0.107972585
ENSGALG00010015391	0.629403017	0.230123838
ENSGALG00010015392	0.846777631	0.30113299

ENSGALG00010015393	0.628783	0.588441226
ENSGALG00010015394	0.930432676	0.156773654
ENSGALG00010015396	0.179975995	0.055700153
ENSGALG00010015397	0.706640474	0.23544162
ENSGALG00010015400	0.269272437	0.073182666
ENSGALG00010015401	0.90340499	0.180581966
ENSGALG00010015402	0.317364765	0.164256295
ENSGALG00010015403	0.200570274	0.091667991
ENSGALG00010015404	0.449876277	0.171691912
ENSGALG00010015406	0.3194288	0.141230071
ENSGALG00010015407	0.925903791	0.220787726
ENSGALG00010015409	0.709498359	0.38073381
ENSGALG00010015410	0.496777251	0.026902031
ENSGALG00010015411	0.223275225	0.21086075
ENSGALG00010015412	0.116753802	0.216599268
ENSGALG00010015413	0.951043286	0.270338305
ENSGALG00010015414	0.396365709	0.157137627
ENSGALG00010015415	0.017411055	0.13007104
ENSGALG00010015416	0.973174328	0.336151439
ENSGALG00010015417	0.441258972	0.224404481
ENSGALG00010015418	0.3212722	0.082708406
ENSGALG00010015419	0.265558089	0.028821141
ENSGALG00010015422	0.30559399	0.04288704
ENSGALG00010015423	0.37526789	0.118093036
ENSGALG00010015424	0.341200679	0.235750727
ENSGALG00010015426	0.40308773	0.146014129
ENSGALG00010015430	0.970922869	0.310110876
ENSGALG00010015431	0.944804339	0.377935235
ENSGALG00010015436	0.943781875	0.297448274
ENSGALG00010015438	0.29624688	0.221994937
ENSGALG00010015439	0.050164932	0.210392742
ENSGALG00010015440	0.941731027	0.290999182
ENSGALG00010015441	0.187182325	0.228815171
ENSGALG00010015443	0.75708374	0.219464398
ENSGALG00010015449	0.727069718	0.312569169
ENSGALG00010015456	0.82853944	0.461782908
ENSGALG00010015457	0.183775481	0.135894353
ENSGALG00010015458	0.012746987	0.420385053
ENSGALG00010015461	0.804501582	0.325970809
ENSGALG00010015466	0.952189127	0.334688745
ENSGALG00010015467	0.031329216	0.144699932
ENSGALG00010015468	0.963353926	0.357952135
ENSGALG00010015469	0.769297981	0.306093153
ENSGALG00010015471	0.992580378	0.340752049
ENSGALG00010015472	0.949130076	0.230681946
ENSGALG00010015474	0.428249613	0.256139924
ENSGALG00010015475	0.303894092	0.04073523
ENSGALG00010015476	0.748750829	0.377438035
ENSGALG00010015477	0.522653581	0.03076589
ENSGALG00010015478	0.04258163	0.065370901
ENSGALG00010015479	0.325566907	0.157344775
ENSGALG00010015480	0.652236755	0.200214783
ENSGALG00010015481	0.840227046	0.451646835
ENSGALG00010015483	0.952297798	0.294129458
ENSGALG00010015484	0.932941463	0.310729614
ENSGALG00010015485	0.777844557	0.470888325

ENSGALG00010015486	0.976349823	0.337757337
ENSGALG00010015487	0.847071896	0.292001997
ENSGALG00010015489	0.571008411	0.152873466
ENSGALG00010015490	0.750828315	0.332724171
ENSGALG00010015491	0.798625639	0.131578225
ENSGALG00010015493	0.978503316	0.397713041
ENSGALG00010015494	0.805498415	0.473484526
ENSGALG00010015495	0.812765127	0.2741179
ENSGALG00010015498	0.37923329	0.504846861
ENSGALG00010015499	0.448478106	0.179792629
ENSGALG00010015501	0.314182873	0.070723306
ENSGALG00010015502	0.189240572	0.04996112
ENSGALG00010015505	0.93486157	0.23351405
ENSGALG00010015506	0.51084206	0.720295104
ENSGALG00010015507	0.733480734	0.180581946
ENSGALG00010015508	0.876179475	0.269983199
ENSGALG00010015511	0.503679165	0.483180131
ENSGALG00010015513	0.371659973	0.137371432
ENSGALG00010015514	0.157098351	0.195363428
ENSGALG00010015516	0.070712624	0.085411293
ENSGALG00010015518	0.927122533	0.326552527
ENSGALG00010015519	0.544366024	0.042888614
ENSGALG00010015520	0.989255863	0.302694109
ENSGALG00010015521	0.619567318	0.350524369
ENSGALG00010015522	0.936827318	0.339547367
ENSGALG00010015523	0.858342432	0.257762071
ENSGALG00010015524	0.750350987	0.109843978
ENSGALG00010015525	0.93720358	0.296923975
ENSGALG00010015526	0.460864324	0.20908277
ENSGALG00010015527	0.146353876	0.171027412
ENSGALG00010015528	0.505011811	0.188959509
ENSGALG00010015531	0.39190877	0.131701825
ENSGALG00010015532	0.843616841	0.269971349
ENSGALG00010015533	0.983297658	0.336574789
ENSGALG00010015534	0.103840257	0.151401781
ENSGALG00010015535	0.155637439	0.051090579
ENSGALG00010015537	0.390218599	0.089311719
ENSGALG00010015539	0.649561588	0.304904556
ENSGALG00010015540	0.908097822	0.205867144
ENSGALG00010015541	0.01125475	0.242609522
ENSGALG00010015542	0.940569546	0.420449333
ENSGALG00010015543	0.43829715	0.089013026
ENSGALG00010015544	0.956624938	0.391927945
ENSGALG00010015545	0.452868476	0.101098737
ENSGALG00010015546	0.468227249	0.169945428
ENSGALG00010015547	0.123715291	0.101269724
ENSGALG00010015551	0.927392503	0.3483985
ENSGALG00010015553	0.845725485	0.286602538
ENSGALG00010015554	0.948613477	0.324880186
ENSGALG00010015555	0.877653251	0.423307216
ENSGALG00010015556	0.266137114	0.107784983
ENSGALG00010015557	0.526829689	0.178638714
ENSGALG00010015558	0.603906421	0.34393111
ENSGALG00010015559	0.384359772	0.116398661
ENSGALG00010015560	0.821594192	0.248321101
ENSGALG00010015561	0.650095249	0.153789455

ENSGALG00010015562	0.755410085	0.316906446
ENSGALG00010015563	0.696542809	0.309579282
ENSGALG00010015564	0.840613186	0.379385162
ENSGALG00010015566	0.270451009	0.090541667
ENSGALG00010015567	0.480513849	0.081742122
ENSGALG00010015568	0.382775976	0.1757733
ENSGALG00010015569	0.219569429	0.22587284
ENSGALG00010015570	0.991825267	0.338629571
ENSGALG00010015571	0.722819041	0.486304695
ENSGALG00010015573	0.940137005	0.303813673
ENSGALG00010015574	0.191574773	0.000963986
ENSGALG00010015576	0.472637348	0.266062131
ENSGALG00010015577	0.376148692	0.091164598
ENSGALG00010015580	0.931979244	0.275615532
ENSGALG00010015582	0.940506858	0.300831959
ENSGALG00010015583	0.995328057	0.305121919
ENSGALG00010015584	0.150085279	0.378465762
ENSGALG00010015585	0.795923895	0.264880429
ENSGALG00010015586	0.219386918	0.079388121
ENSGALG00010015587	0.386254376	0.161451439
ENSGALG00010015588	0.073732153	0.097669803
ENSGALG00010015589	0.955620307	0.292992502
ENSGALG00010015590	0.215256434	0.218672447
ENSGALG00010015592	0.628575001	0.314169702
ENSGALG00010015593	0.067621059	0.167686639
ENSGALG00010015594	0.911060615	0.33720526
ENSGALG00010015596	0.677291081	0.295865878
ENSGALG00010015599	0.871038744	0.496603493
ENSGALG00010015600	0.377096693	0.134016806
ENSGALG00010015603	0.967838402	0.332235708
ENSGALG00010015605	0.628052373	0.058087595
ENSGALG00010015606	0.40691561	0.100611437
ENSGALG00010015607	0.350670686	0.28946165
ENSGALG00010015608	0.39035496	0.270804034
ENSGALG00010015609	0.240644424	0.208767635
ENSGALG00010015610	0.988674944	0.31067105
ENSGALG00010015613	0.218239996	0.292028106
ENSGALG00010015614	0.02031629	0.107383513
ENSGALG00010015615	0.224449644	0.273689373
ENSGALG00010015616	0.99142721	0.340012643
ENSGALG00010015617	0.738205826	0.02985613
ENSGALG00010015618	0.539375996	0.184403832
ENSGALG00010015620	0.972190157	0.274995898
ENSGALG00010015621	0.927246879	0.195129751
ENSGALG00010015622	0.279729665	0.482251581
ENSGALG00010015623	0.489825871	0.517747953
ENSGALG00010015624	0.971184531	0.242729016
ENSGALG00010015625	0.827227507	0.277870705
ENSGALG00010015626	0.015005683	0.32469751
ENSGALG00010015627	0.327036234	0.031202811
ENSGALG00010015628	0.952369557	0.207677057
ENSGALG00010015629	0.397400334	0.1702444
ENSGALG00010015630	0.567107104	0.051764356
ENSGALG00010015631	0.572367007	0.162614151
ENSGALG00010015632	0.920369152	0.326012237
ENSGALG00010015633	0.791791066	0.424592878

ENSGALG00010015635	0.770443695	0.199370644
ENSGALG00010015636	0.987213775	0.313371419
ENSGALG00010015637	0.911447213	0.295113171
ENSGALG00010015640	0.724289608	0.138918086
ENSGALG00010015641	0.617798173	0.026016793
ENSGALG00010015643	0.641123156	0.47917747
ENSGALG00010015644	0.18401263	0.070193553
ENSGALG00010015645	0.948978142	0.321666576
ENSGALG00010015647	0.574265051	0.317297889
ENSGALG00010015648	0.264239718	0.073427965
ENSGALG00010015649	0.092293281	0.247321007
ENSGALG00010015650	0.388404098	0.148641621
ENSGALG00010015653	0.870652211	0.390487493
ENSGALG00010015654	0.229443295	0.170412511
ENSGALG00010015655	0.479010373	0.036739982
ENSGALG00010015656	0.742068881	0.107116936
ENSGALG00010015660	0.947682089	0.339481469
ENSGALG00010015663	0.482145283	0.177847326
ENSGALG00010015664	0.607040673	0.242668684
ENSGALG00010015666	0.469177354	0.169929321
ENSGALG00010015667	0.364871524	0.123582396
ENSGALG00010015668	0.965618523	0.437687099
ENSGALG00010015669	0.884610181	0.356095515
ENSGALG00010015670	0.411893538	0.021669246
ENSGALG00010015671	0.269990618	0.090697252
ENSGALG00010015672	0.154035871	0.017573108
ENSGALG00010015673	0.930573256	0.435095797
ENSGALG00010015674	0.810328307	0.338307569
ENSGALG00010015676	0.547132379	0.211320092
ENSGALG00010015677	0.467380829	0.3175207
ENSGALG00010015679	0.323858548	0.343474408
ENSGALG00010015680	0.71119465	0.215198889
ENSGALG00010015682	0.22804884	0.128559605
ENSGALG00010015684	0.415155335	0.097831039
ENSGALG00010015685	0.067820563	0.132238412
ENSGALG00010015686	0.95797497	0.254974496
ENSGALG00010015687	0.934743716	0.310077369
ENSGALG00010015691	0.988189868	0.350242784
ENSGALG00010015693	0.937691982	0.282592414
ENSGALG00010015694	0.37479064	0.077949893
ENSGALG00010015695	0.975001599	0.268687537
ENSGALG00010015696	0.266137114	0.107784983
ENSGALG00010015697	0.88866038	0.293966385
ENSGALG00010015698	0.818060676	0.136718322
ENSGALG00010015700	0.306269874	0.488490028
ENSGALG00010015701	0.765588748	0.277434282
ENSGALG00010015702	0.450194842	0.071698579
ENSGALG00010015703	0.940681022	0.315154953
ENSGALG00010015705	0.015356881	0.221156745
ENSGALG00010015706	0.545057	0.058394904
ENSGALG00010015709	0.577329906	0.338048169
ENSGALG00010015710	0.09323381	0.14790944
ENSGALG00010015711	0.991234186	0.396966641
ENSGALG00010015714	0.975437953	0.332064529
ENSGALG00010015716	0.962343927	0.297048089
ENSGALG00010015718	0.431553612	0.192661548

ENSGALG00010015719	0.87295564	0.37542866
ENSGALG00010015721	0.483044133	0.200897137
ENSGALG00010015722	0.577932677	0.204867016
ENSGALG00010015723	0.395556858	0.102965232
ENSGALG00010015725	0.264239718	0.073427965
ENSGALG00010015728	0.41983171	0.288568924
ENSGALG00010015729	0.712169999	0.289587992
ENSGALG00010015730	0.89590453	0.285830281
ENSGALG00010015731	0.89566972	0.435874208
ENSGALG00010015732	0.90711206	0.325222551
ENSGALG00010015733	0.971142215	0.370539915
ENSGALG00010015734	0.233337379	0.002929328
ENSGALG00010015735	0.870976367	0.29001576
ENSGALG00010015736	0.12691976	0.23780547
ENSGALG00010015737	0.0510309	0.078661096
ENSGALG00010015738	0.81198594	0.077874235
ENSGALG00010015742	0.969967756	0.29883654
ENSGALG00010015743	0.583676413	0.228984121
ENSGALG00010015744	0.408109737	0.157233138
ENSGALG00010015745	0.482335527	0.147132467
ENSGALG00010015746	0.520605378	0.435508582
ENSGALG00010015747	0.754256363	0.281081381
ENSGALG00010015749	0.746245098	0.478011184
ENSGALG00010015750	0.416169558	0.2793359
ENSGALG00010015751	0.416007421	0.362106739
ENSGALG00010015752	0.926407086	0.284296921
ENSGALG00010015753	0.542411778	0.456876762
ENSGALG00010015756	0.816642054	0.305534847
ENSGALG00010015757	0.970065325	0.36111586
ENSGALG00010015758	0.469212831	0.389117892
ENSGALG00010015759	0.826896568	0.086081902
ENSGALG00010015762	0.37552597	0.101692059
ENSGALG00010015764	0.285992397	0.373119687
ENSGALG00010015765	0.22554445	0.02819802
ENSGALG00010015766	0.922316143	0.333695934
ENSGALG00010015769	0.845037666	0.268195939
ENSGALG00010015771	0.079762376	0.384839794
ENSGALG00010015772	0.317994761	0.302132706
ENSGALG00010015773	0.435249353	0.209258117
ENSGALG00010015774	0.867021418	0.338581822
ENSGALG00010015776	0.155965215	0.152430822
ENSGALG00010015778	0.920180145	0.419546759
ENSGALG00010015780	0.392894344	0.133607003
ENSGALG00010015781	0.483761819	0.14768566
ENSGALG00010015782	0.743568954	0.063902705
ENSGALG00010015785	0.672183344	0.420440896
ENSGALG00010015787	0.799033558	0.242705882
ENSGALG00010015788	0.373640438	0.161243791
ENSGALG00010015789	0.270451009	0.090541667
ENSGALG00010015790	0.56591591	0.278242648
ENSGALG00010015791	0.588483485	0.08307131
ENSGALG00010015792	0.975807272	0.244794705
ENSGALG00010015793	0.388550926	0.184246236
ENSGALG00010015794	0.308844183	0.110939188
ENSGALG00010015795	0.760967977	0.259166587
ENSGALG00010015796	0.742401652	0.197685779

ENSGALG00010015797	0.859684337	0.305283592
ENSGALG00010015798	0.917051463	0.25662841
ENSGALG00010015799	0.096860439	0.341799876
ENSGALG00010015800	0.987706584	0.319169375
ENSGALG00010015801	0.219293666	0.049108603
ENSGALG00010015802	0.821390218	0.323876199
ENSGALG00010015803	0.669308604	0.028794303
ENSGALG00010015804	0.256887088	0.302367881
ENSGALG00010015806	0.266137114	0.107784983
ENSGALG00010015807	0.628366251	0.038245468
ENSGALG00010015808	0.463783118	0.149693479
ENSGALG00010015809	0.822102007	0.363834224
ENSGALG00010015810	0.541128613	0.149408069
ENSGALG00010015811	0.877875365	0.307203264
ENSGALG00010015813	0.929539101	0.281562612
ENSGALG00010015814	0.963766347	0.35948718
ENSGALG00010015815	0.230766395	0.172305962
ENSGALG00010015816	0.327496192	0.044761675
ENSGALG00010015817	0.952927459	0.273872557
ENSGALG00010015818	0.530956342	0.24764282
ENSGALG00010015819	0.603015529	0.041846011
ENSGALG00010015820	0.751368038	0.326999087
ENSGALG00010015821	0.488860496	0.212264849
ENSGALG00010015822	0.489121629	0.131571089
ENSGALG00010015823	0.958778843	0.324226427
ENSGALG00010015824	0.323105438	0.173197139
ENSGALG00010015825	0.904046355	0.49703093
ENSGALG00010015827	0.433358478	0.454502492
ENSGALG00010015829	0.878225772	0.066134041
ENSGALG00010015830	0.271701261	0.072417625
ENSGALG00010015831	0.976928603	0.283632967
ENSGALG00010015832	0.938259791	0.316151306
ENSGALG00010015833	0.044969106	0.225756118
ENSGALG00010015834	0.65632332	0.116467077
ENSGALG00010015835	0.043659357	0.108092216
ENSGALG00010015836	0.064166065	0.566329296
ENSGALG00010015837	0.954740742	0.234948801
ENSGALG00010015839	0.95808357	0.295517081
ENSGALG00010015840	0.572845382	0.258869917
ENSGALG00010015841	0.716823214	0.465978752
ENSGALG00010015842	0.265558089	0.028821141
ENSGALG00010015843	0.052124379	0.605798082
ENSGALG00010015844	0.8386183	0.126766841
ENSGALG00010015845	0.942462263	0.24357382
ENSGALG00010015846	0.289661925	0.000223194
ENSGALG00010015847	0.09147966	0.071568051
ENSGALG00010015848	0.853818065	0.223234065
ENSGALG00010015850	0.33717368	0.193442029
ENSGALG00010015852	0.943290432	0.326606766
ENSGALG00010015855	0.879853632	0.30630345
ENSGALG00010015856	0.966446506	0.246754226
ENSGALG00010015857	0.200570274	0.091667991
ENSGALG00010015858	0.947711879	0.323030405
ENSGALG00010015860	0.065670831	0.18287313
ENSGALG00010015861	0.963711628	0.339239591
ENSGALG00010015862	0.929836596	0.464617144

ENSGALG00010015863	0.011074683	0.420588111
ENSGALG00010015865	0.882312306	0.342325798
ENSGALG00010015866	0.104729442	0.050076474
ENSGALG00010015867	0.920548719	0.404871988
ENSGALG00010015868	0.233036103	0.331083723
ENSGALG00010015869	0.913713688	0.360152878
ENSGALG00010015870	0.957352353	0.276153033
ENSGALG00010015871	0.449621013	0.468709091
ENSGALG00010015872	0.243187225	0.24776988
ENSGALG00010015873	0.710240385	0.016597102
ENSGALG00010015874	0.665925693	0.023310875
ENSGALG00010015875	0.974890461	0.300242823
ENSGALG00010015876	0.505628957	0.183652593
ENSGALG00010015877	0.56337362	0.282257147
ENSGALG00010015878	0.583416119	0.122759513
ENSGALG00010015879	0.502922234	0.03208718
ENSGALG00010015881	0.926598603	0.170187632
ENSGALG00010015883	0.541904204	0.539011532
ENSGALG00010015886	0.935107787	0.233277877
ENSGALG00010015887	0.813057541	0.205342295
ENSGALG00010015888	0.392206929	0.143464443
ENSGALG00010015889	0.97041813	0.26379729
ENSGALG00010015892	0.154867609	0.13140084
ENSGALG00010015893	0.96577922	0.44901822
ENSGALG00010015894	0.968598355	0.295668942
ENSGALG00010015895	0.895683702	0.341284789
ENSGALG00010015896	0.494663885	0.070182632
ENSGALG00010015897	0.271701261	0.072417625
ENSGALG00010015898	0.815733162	0.363119988
ENSGALG00010015899	0.946326873	0.41012017
ENSGALG00010015900	0.816800526	0.326525182
ENSGALG00010015901	0.044515167	0.113439077
ENSGALG00010015902	0.906614367	0.249859614
ENSGALG00010015904	0.937232648	0.340973595
ENSGALG00010015905	0.965071951	0.277268924
ENSGALG00010015906	0.028559198	0.363224209
ENSGALG00010015908	0.536154111	0.480254771
ENSGALG00010015909	0.98911239	0.323250151
ENSGALG00010015910	0.425008326	0.334624978
ENSGALG00010015911	0.024032732	0.04687212
ENSGALG00010015912	0.121870983	0.37074683
ENSGALG00010015913	0.793803355	0.16248863
ENSGALG00010015914	0.918446796	0.375045717
ENSGALG00010015916	0.923952553	0.168362687
ENSGALG00010015917	0.708635169	0.202198295
ENSGALG00010015918	0.447143733	0.496786036
ENSGALG00010015919	0.45202616	0.044117872
ENSGALG00010015921	0.982218765	0.356507235
ENSGALG00010015923	0.971008108	0.363165715
ENSGALG00010015927	0.82605348	0.412321465
ENSGALG00010015928	0.459599422	0.198601935
ENSGALG00010015929	0.985435613	0.305182509
ENSGALG00010015931	0.984371967	0.238060149
ENSGALG00010015932	0.45244244	0.001177905
ENSGALG00010015934	0.575160268	0.167347099
ENSGALG00010015935	0.986091515	0.322264834

ENSGALG00010015936	0.863846097	0.314008088
ENSGALG00010015937	0.879049644	0.264171797
ENSGALG00010015939	0.927969779	0.385366822
ENSGALG00010015940	0.388571781	0.151666204
ENSGALG00010015942	0.853735869	0.279009834
ENSGALG00010015943	0.450104737	0.172335588
ENSGALG00010015946	0.5905019	0.365551696
ENSGALG00010015951	0.951406552	0.350853777
ENSGALG00010015952	0.270451009	0.090541667
ENSGALG00010015953	0.152271242	0.081723275
ENSGALG00010015954	0.732691444	0.207050588
ENSGALG00010015955	0.842221461	0.340037603
ENSGALG00010015957	0.968747155	0.287084866
ENSGALG00010015958	0.807681228	0.270785519
ENSGALG00010015959	0.962144542	0.302747224
ENSGALG00010015960	0.893872951	0.337227998
ENSGALG00010015961	0.711492962	0.06668915
ENSGALG00010015963	0.777981912	0.437865038
ENSGALG00010015964	0.566136218	0.235903171
ENSGALG00010015965	0.354243613	0.162759819
ENSGALG00010015967	0.174926759	0.266538787
ENSGALG00010015968	0.219049348	0.375294351
ENSGALG00010015970	0.904544111	0.452997219
ENSGALG00010015971	0.583979397	0.23535048
ENSGALG00010015972	0.204262592	0.418689276
ENSGALG00010015973	0.743112664	0.252436153
ENSGALG00010015974	0.895121723	0.319362928
ENSGALG00010015975	0.937844571	0.329574679
ENSGALG00010015976	0.481606469	0.120553617
ENSGALG00010015977	0.017946301	0.123217195
ENSGALG00010015978	0.384332656	0.113021677
ENSGALG00010015979	0.971887455	0.396993832
ENSGALG00010015980	0.665948712	0.417189323
ENSGALG00010015981	0.384165008	0.165101073
ENSGALG00010015983	0.807088569	0.415807775
ENSGALG00010015984	0.982338871	0.343467398
ENSGALG00010015985	0.645072317	0.044291338
ENSGALG00010015986	0.991242058	0.325898968
ENSGALG00010015987	0.722586489	0.403935193
ENSGALG00010015988	0.140537457	0.874033653
ENSGALG00010015989	0.705526841	0.166721381
ENSGALG00010015990	0.183539761	0.191827542
ENSGALG00010015992	0.868271651	0.112958456
ENSGALG00010015993	0.40308773	0.146014129
ENSGALG00010015994	0.695999801	0.135641895
ENSGALG00010016001	0.258959909	0.095870072
ENSGALG00010016002	0.636191667	0.195200254
ENSGALG00010016004	0.896709346	0.464632526
ENSGALG00010016005	0.525295177	0.328372109
ENSGALG00010016006	0.63521956	0.226888105
ENSGALG00010016008	0.108595938	0.155410534
ENSGALG00010016009	0.72673248	0.216249646
ENSGALG00010016010	0.054298359	0.498888325
ENSGALG00010016011	0.936398638	0.334852521
ENSGALG00010016012	0.29169373	0.221115931
ENSGALG00010016013	0.050070995	0.079130983

ENSGALG00010016014	0.821426253	0.256801182
ENSGALG00010016015	0.971946677	0.312617167
ENSGALG00010016016	0.938651127	0.271322963
ENSGALG00010016017	0.95338468	0.296436649
ENSGALG00010016018	0.329744371	0.371370966
ENSGALG00010016020	0.200570274	0.091667991
ENSGALG00010016021	0.319349856	0.37353642
ENSGALG00010016022	0.126969977	0.169747044
ENSGALG00010016023	0.444710566	0.414277856
ENSGALG00010016024	0.358793093	0.212812756
ENSGALG00010016025	0.915807016	0.344278123
ENSGALG00010016026	0.388181886	0.168275567
ENSGALG00010016027	0.987281981	0.305552003
ENSGALG00010016028	0.907626586	0.447893916
ENSGALG00010016030	0.952166999	0.342219835
ENSGALG00010016031	0.284649087	0.277893399
ENSGALG00010016038	0.025180588	0.075082988
ENSGALG00010016039	0.296272339	0.491563762
ENSGALG00010016040	0.894422304	0.314041616
ENSGALG00010016041	0.721738097	0.216975797
ENSGALG00010016042	0.409525165	0.299962141
ENSGALG00010016043	0.759018182	0.234401631
ENSGALG00010016044	0.175495308	0.078870995
ENSGALG00010016045	0.46575669	0.153341734
ENSGALG00010016046	0.476985655	0.249084232
ENSGALG00010016047	0.965749605	0.301889904
ENSGALG00010016048	0.833707815	0.493375693
ENSGALG00010016049	0.975748695	0.362186092
ENSGALG00010016051	0.535784444	0.730539701
ENSGALG00010016052	0.235630588	0.132393411
ENSGALG00010016053	0.602144261	0.205081217
ENSGALG00010016057	0.286562309	0.078822832
ENSGALG00010016058	0.383947044	0.128562519
ENSGALG00010016059	0.989529192	0.314522935
ENSGALG00010016062	0.902979949	0.183430948
ENSGALG00010016065	0.135297277	0.028198572
ENSGALG00010016066	0.936048735	0.33064406
ENSGALG00010016067	0.994065125	0.27499534
ENSGALG00010016068	0.504336944	0.078426904
ENSGALG00010016069	0.92516945	0.21683967
ENSGALG00010016071	0.549393522	0.332801912
ENSGALG00010016073	0.420716904	0.48115035
ENSGALG00010016075	0.371576879	0.166670591
ENSGALG00010016076	0.066054439	0.23146438
ENSGALG00010016077	0.249286039	0.212819636
ENSGALG00010016079	0.54886763	0.517632316
ENSGALG00010016082	0.756643304	0.390030026
ENSGALG00010016083	0.286425688	0.308748051
ENSGALG00010016086	0.126063904	0.268218517
ENSGALG00010016087	0.539158557	0.014841275
ENSGALG00010016088	0.200570274	0.091667991
ENSGALG00010016089	0.382977183	0.103780214
ENSGALG00010016090	0.562562737	0.253096747
ENSGALG00010016093	0.739836792	0.140121554
ENSGALG00010016094	0.92591897	0.329097154
ENSGALG00010016097	0.935128173	0.241193997

ENSGALG00010016098	0.962944884	0.315769782
ENSGALG00010016099	0.390466756	0.106163828
ENSGALG00010016100	0.349179242	0.107617825
ENSGALG00010016103	0.97919737	0.315171441
ENSGALG00010016107	0.524338045	0.170935322
ENSGALG00010016109	0.323212609	0.29722347
ENSGALG00010016111	0.833672126	0.258618705
ENSGALG00010016112	0.09938646	0.144176797
ENSGALG00010016113	0.774936748	0.156271524
ENSGALG00010016114	0.758986905	0.106619939
ENSGALG00010016115	0.31655667	0.016549586
ENSGALG00010016117	0.660233912	0.038202284
ENSGALG00010016118	0.947409282	0.212390035
ENSGALG00010016119	0.993826534	0.304842373
ENSGALG00010016121	0.987455182	0.30081227
ENSGALG00010016122	0.616512797	0.211900992
ENSGALG00010016123	0.832311765	0.281287072
ENSGALG00010016124	0.248167198	0.219554426
ENSGALG00010016125	0.078535062	0.140437839
ENSGALG00010016126	0.827411852	0.473072173
ENSGALG00010016127	0.824989638	0.399259558
ENSGALG00010016128	0.689114744	0.272345346
ENSGALG00010016129	0.573465061	0.097256739
ENSGALG00010016131	0.953426728	0.340629187
ENSGALG00010016132	0.270451009	0.090541667
ENSGALG00010016133	0.865675305	0.364023775
ENSGALG00010016136	0.186242382	0.419579744
ENSGALG00010016137	0.938599534	0.475767697
ENSGALG00010016138	0.80408886	0.281467498
ENSGALG00010016139	0.058311168	0.076408812
ENSGALG00010016140	0.749297364	0.265235164
ENSGALG00010016141	0.679683403	0.130018088
ENSGALG00010016142	0.185453987	0.140651822
ENSGALG00010016144	0.285272007	0.109169955
ENSGALG00010016147	0.99342931	0.300825621
ENSGALG00010016150	0.793860588	0.504528562
ENSGALG00010016151	0.385917349	0.261153519
ENSGALG00010016154	0.485801976	0.167509571
ENSGALG00010016155	0.516740371	0.188296051
ENSGALG00010016158	0.392587933	0.102850336
ENSGALG00010016159	0.985708036	0.345880759
ENSGALG00010016160	0.387818129	0.081671798
ENSGALG00010016163	0.813959904	0.239315922
ENSGALG00010016164	0.796065612	0.401815671
ENSGALG00010016165	0.67022071	0.385731031
ENSGALG00010016166	0.877257798	0.347434083
ENSGALG00010016167	0.269241976	0.073173439
ENSGALG00010016168	0.908705675	0.249021229
ENSGALG00010016169	0.060067667	0.22427787
ENSGALG00010016173	0.19921065	0.101741548
ENSGALG00010016176	0.220986766	0.039126356
ENSGALG00010016177	0.995774624	0.345564723
ENSGALG00010016181	0.935050611	0.381619644
ENSGALG00010016182	0.851947731	0.282948398
ENSGALG00010016188	0.69391123	0.375947279
ENSGALG00010016190	0.913013088	0.324517425

ENSGALG00010016193	0.929377859	0.455743393
ENSGALG00010016197	0.765078395	0.233015864
ENSGALG00010016199	0.085081312	0.245008948
ENSGALG00010016200	0.309444066	0.156651418
ENSGALG00010016204	0.927613457	0.220236117
ENSGALG00010016205	0.732103343	0.608646457
ENSGALG00010016206	0.988484873	0.387836869
ENSGALG00010016207	0.676465569	0.249633426
ENSGALG00010016208	0.395556858	0.102965232
ENSGALG00010016209	0.742260876	0.226775094
ENSGALG00010016210	0.219386918	0.079388121
ENSGALG00010016211	0.828271986	0.1864031
ENSGALG00010016212	0.294869419	0.092251697
ENSGALG00010016214	0.011730111	0.196288363
ENSGALG00010016215	0.170955113	0.137699973
ENSGALG00010016218	0.764327193	0.06306163
ENSGALG00010016221	0.801743528	0.247720686
ENSGALG00010016224	0.863356925	0.244150624
ENSGALG00010016225	0.773538286	0.173910132
ENSGALG00010016227	0.733425053	0.213079039
ENSGALG00010016229	0.996247266	0.347295786
ENSGALG00010016231	0.898065918	0.305050482
ENSGALG00010016232	0.273203553	0.22470318
ENSGALG00010016235	0.466631312	0.050994032
ENSGALG00010016236	0.799469086	0.19318674
ENSGALG00010016240	0.938673659	0.143796617
ENSGALG00010016241	0.956294086	0.353147549
ENSGALG00010016242	0.878846264	0.163029352
ENSGALG00010016243	0.168323018	0.058419001
ENSGALG00010016245	0.945642605	0.237947464
ENSGALG00010016247	0.713988731	0.34842621
ENSGALG00010016248	0.483659028	0.368376674
ENSGALG00010016249	0.97301695	0.292163784
ENSGALG00010016251	0.8455816	0.125913634
ENSGALG00010016253	0.526867884	0.100558144
ENSGALG00010016255	0.913529064	0.345890534
ENSGALG00010016257	0.817555658	0.384868282
ENSGALG00010016258	0.959844839	0.267111325
ENSGALG00010016260	0.525466999	0.392861981
ENSGALG00010016262	0.538175519	0.125502819
ENSGALG00010016263	0.996595544	0.307776116
ENSGALG00010016266	0.931490622	0.422957705
ENSGALG00010016270	0.521605846	0.501890082
ENSGALG00010016273	0.548737434	0.159124595
ENSGALG00010016274	0.874905482	0.025120849
ENSGALG00010016275	0.014136607	0.064713326
ENSGALG00010016276	0.93407827	0.330681508
ENSGALG00010016277	0.450029279	0.406839901
ENSGALG00010016279	0.77682189	0.18333656
ENSGALG00010016280	0.140487315	0.226137306
ENSGALG00010016281	0.981622152	0.376819288
ENSGALG00010016284	0.04285744	0.659429294
ENSGALG00010016286	0.469148654	0.455725825
ENSGALG00010016287	0.922655465	0.402584868
ENSGALG00010016288	0.488583769	0.130802663
ENSGALG00010016289	0.946117958	0.309092661

ENSGALG00010016291	0.360497503	0.054970648
ENSGALG00010016292	0.99839086	0.338324894
ENSGALG00010016295	0.919113601	0.293478409
ENSGALG00010016297	0.560462923	0.162133252
ENSGALG00010016298	0.204552216	0.204121451
ENSGALG00010016299	0.863676354	0.346727197
ENSGALG00010016301	0.688066446	0.549758404
ENSGALG00010016302	0.516804126	0.16545839
ENSGALG00010016303	0.894934996	0.309324279
ENSGALG00010016304	0.990270361	0.311473686
ENSGALG00010016305	0.971171265	0.430944186
ENSGALG00010016306	0.964153135	0.286008379
ENSGALG00010016307	0.965150522	0.293416641
ENSGALG00010016308	0.593629743	0.244773828
ENSGALG00010016309	0.365401829	0.098829438
ENSGALG00010016310	0.954916147	0.40800133
ENSGALG00010016312	0.564461379	0.041067284
ENSGALG00010016314	0.758057184	0.399201016
ENSGALG00010016315	0.763356188	0.314953207
ENSGALG00010016316	0.571240054	0.18833381
ENSGALG00010016317	0.872202344	0.122982996
ENSGALG00010016319	0.88720095	0.275994982
ENSGALG00010016320	0.146098381	0.192474529
ENSGALG00010016322	0.949066362	0.298811973
ENSGALG00010016324	0.045074849	0.138626677
ENSGALG00010016325	0.761836426	0.226591284
ENSGALG00010016326	0.435281146	0.220330623
ENSGALG00010016327	0.208550923	0.676301294
ENSGALG00010016328	0.983707791	0.337278192
ENSGALG00010016330	0.193293814	0.024632447
ENSGALG00010016331	0.731703313	0.268139745
ENSGALG00010016332	0.094833829	0.005003323
ENSGALG00010016333	0.659030536	0.228077943
ENSGALG00010016334	0.812851675	0.287846728
ENSGALG00010016335	0.907098817	0.277330185
ENSGALG00010016336	0.681223765	0.336412272
ENSGALG00010016337	0.879121572	0.324144617
ENSGALG00010016338	0.945081986	0.122465041
ENSGALG00010016339	0.956375093	0.274485377
ENSGALG00010016340	0.933213122	0.41423777
ENSGALG00010016341	0.884948878	0.262786137
ENSGALG00010016342	0.87747416	0.329387921
ENSGALG00010016344	0.966378172	0.283963228
ENSGALG00010016345	0.926085236	0.323286864
ENSGALG00010016346	0.846751255	0.289975882
ENSGALG00010016347	0.944982563	0.360403702
ENSGALG00010016349	0.264239718	0.073427965
ENSGALG00010016350	0.226856701	0.276372299
ENSGALG00010016351	0.606414747	0.410267991
ENSGALG00010016352	0.877897249	0.326273224
ENSGALG00010016353	0.216690666	0.254935735
ENSGALG00010016355	0.527195777	0.039333227
ENSGALG00010016357	0.467275937	0.311472226
ENSGALG00010016359	0.20887102	0.308792689
ENSGALG00010016361	0.720241619	0.195852243
ENSGALG00010016362	0.873639959	0.340936668

ENSGALG00010016363	0.540443997	0.281586923
ENSGALG00010016365	0.099028626	0.079591415
ENSGALG00010016366	0.068483667	0.200974959
ENSGALG00010016367	0.285272007	0.109169955
ENSGALG00010016368	0.758290251	0.048662516
ENSGALG00010016369	0.49667724	0.129274834
ENSGALG00010016370	0.969800882	0.344899493
ENSGALG00010016372	0.906244171	0.270662569
ENSGALG00010016373	0.992537878	0.341533625
ENSGALG00010016374	0.988012719	0.308581907
ENSGALG00010016375	0.931744521	0.402650935
ENSGALG00010016376	0.803512967	0.278209456
ENSGALG00010016377	0.935532184	0.346567772
ENSGALG00010016378	0.921733994	0.230796713
ENSGALG00010016379	0.468375439	0.245945711
ENSGALG00010016380	0.665671155	0.036674639
ENSGALG00010016382	0.983552374	0.328938189
ENSGALG00010016383	0.483179301	0.241270085
ENSGALG00010016384	0.534273356	0.070255614
ENSGALG00010016385	0.956888266	0.392319116
ENSGALG00010016386	0.767428465	0.319048214
ENSGALG00010016387	0.944094979	0.37318035
ENSGALG00010016390	0.682325725	0.064250629
ENSGALG00010016392	0.030219813	0.063120102
ENSGALG00010016393	0.751629557	0.374970015
ENSGALG00010016394	0.926494179	0.309677717
ENSGALG00010016395	0.832771453	0.259524669
ENSGALG00010016397	0.845177809	0.204294219
ENSGALG00010016398	0.733820073	0.415259534
ENSGALG00010016399	0.94033757	0.430004942
ENSGALG00010016400	0.996331474	0.316450302
ENSGALG00010016402	0.956576879	0.292344008
ENSGALG00010016403	0.918542115	0.323408046
ENSGALG00010016404	0.940547038	0.400056506
ENSGALG00010016406	0.917545795	0.321131368
ENSGALG00010016407	0.756209149	0.354575436
ENSGALG00010016409	0.933188591	0.314622269
ENSGALG00010016410	0.92616268	0.304899598
ENSGALG00010016411	0.904558374	0.22975824
ENSGALG00010016412	0.975658205	0.328895926
ENSGALG00010016413	0.442172085	0.226056926
ENSGALG00010016414	0.979377314	0.292955789
ENSGALG00010016415	0.400164632	0.160024928
ENSGALG00010016416	0.904654296	0.161745178
ENSGALG00010016417	0.532864725	0.196644624
ENSGALG00010016418	0.422524278	0.153442406
ENSGALG00010016421	0.301753662	0.0251386
ENSGALG00010016422	0.992500165	0.350196999
ENSGALG00010016423	0.192751706	0.225726704
ENSGALG00010016424	0.331267319	0.379636003
ENSGALG00010016425	0.984853459	0.296475239
ENSGALG00010016426	0.952825288	0.326033047
ENSGALG00010016428	0.297013702	0.006120238
ENSGALG00010016429	0.434892598	0.262376375
ENSGALG00010016431	0.376156992	0.044713964
ENSGALG00010016432	0.495911024	0.154704313

ENSGALG00010016433	0.838499816	0.197710326
ENSGALG00010016434	0.380838651	0.159033541
ENSGALG00010016436	0.719813134	0.061866246
ENSGALG00010016437	0.90734651	0.331053893
ENSGALG00010016438	0.500645134	0.208879733
ENSGALG00010016440	0.381352663	0.078374659
ENSGALG00010016441	0.12830585	0.232254388
ENSGALG00010016444	0.48775796	0.189318879
ENSGALG00010016445	0.862800815	0.366439285
ENSGALG00010016447	0.95703057	0.301141875
ENSGALG00010016449	0.718884236	0.326248122
ENSGALG00010016450	0.318640984	0.296289522
ENSGALG00010016451	0.945268922	0.225526116
ENSGALG00010016452	0.591692612	0.077419841
ENSGALG00010016453	0.911778563	0.228871181
ENSGALG00010016454	0.923577977	0.335534742
ENSGALG00010016455	0.777226027	0.092274481
ENSGALG00010016456	0.917110714	0.362571912
ENSGALG00010016457	0.765451421	0.15842002
ENSGALG00010016458	0.857304442	0.202504935
ENSGALG00010016459	0.6612653	0.165259428
ENSGALG00010016460	0.979176088	0.325910833
ENSGALG00010016461	0.753743318	0.456758783
ENSGALG00010016462	0.530825215	0.061122277
ENSGALG00010016463	0.453378702	0.252065869
ENSGALG00010016464	0.085893651	0.227642825
ENSGALG00010016465	0.444161307	0.362728448
ENSGALG00010016466	0.892115836	0.323188968
ENSGALG00010016468	0.965827575	0.243111349
ENSGALG00010016469	0.4385448	0.153973416
ENSGALG00010016470	0.029454428	0.045015667
ENSGALG00010016471	0.988008495	0.37299672
ENSGALG00010016472	0.386042959	0.251457033
ENSGALG00010016473	0.345905068	0.137054118
ENSGALG00010016474	0.424932398	0.216580534
ENSGALG00010016475	0.776865605	0.126455788
ENSGALG00010016476	0.08796732	0.167832124
ENSGALG00010016477	0.515522889	0.18220432
ENSGALG00010016479	0.570491898	0.245923558
ENSGALG00010016480	0.454712994	0.241250554
ENSGALG00010016482	0.985744687	0.318260979
ENSGALG00010016483	0.771403022	0.313789003
ENSGALG00010016485	0.486398155	0.197317856
ENSGALG00010016486	0.42204462	0.074337987
ENSGALG00010016487	0.54083973	0.020071667
ENSGALG00010016489	0.308949431	0.165913182
ENSGALG00010016490	0.198445645	0.09434737
ENSGALG00010016491	0.811581336	0.249513709
ENSGALG00010016492	0.497995268	0.219620827
ENSGALG00010016493	0.156127797	0.48196368
ENSGALG00010016494	0.701096922	0.208645755
ENSGALG00010016496	0.892971968	0.413297338
ENSGALG00010016497	0.41088235	0.529716891
ENSGALG00010016498	0.615944922	0.107553777
ENSGALG00010016499	0.204625936	0.347920376
ENSGALG00010016500	0.968251199	0.300478386

ENSGALG00010016501	0.18434576	0.199610304
ENSGALG00010016502	0.975582419	0.313129644
ENSGALG00010016503	0.300059767	0.357958306
ENSGALG00010016504	0.773253986	0.268842096
ENSGALG00010016505	0.034807712	0.02685467
ENSGALG00010016506	0.587681599	0.293270196
ENSGALG00010016507	0.742429589	0.227597598
ENSGALG00010016508	0.75642296	0.097657671
ENSGALG00010016509	0.659608416	0.021347957
ENSGALG00010016510	0.194772588	0.214413605
ENSGALG00010016511	0.754160984	0.023464616
ENSGALG00010016512	0.438852492	0.143468944
ENSGALG00010016513	0.050115024	0.155140157
ENSGALG00010016514	0.568888056	0.50615468
ENSGALG00010016517	0.316133857	0.530068019
ENSGALG00010016518	0.520808633	0.326810167
ENSGALG00010016519	0.894982107	0.353133217
ENSGALG00010016520	0.104015521	0.29833598
ENSGALG00010016521	0.804909383	0.287464548
ENSGALG00010016522	0.687836685	0.184195047
ENSGALG00010016523	0.36451069	0.333737215
ENSGALG00010016526	0.963925992	0.277969428
ENSGALG00010016527	0.673480714	0.220125385
ENSGALG00010016529	0.404862142	0.155183269
ENSGALG00010016531	0.94691447	0.307357683
ENSGALG00010016532	0.933161401	0.330191379
ENSGALG00010016533	0.761176864	0.088460662
ENSGALG00010016534	0.669126764	0.06137904
ENSGALG00010016535	0.664119606	0.387161952
ENSGALG00010016538	0.666969823	0.351340513
ENSGALG00010016539	0.216433634	0.680598293
ENSGALG00010016541	0.193702696	0.019758057
ENSGALG00010016542	0.445136375	0.008031032
ENSGALG00010016543	0.91397227	0.245501278
ENSGALG00010016544	0.991678416	0.298114494
ENSGALG00010016545	0.376085884	0.180749605
ENSGALG00010016546	0.705097455	0.288132886
ENSGALG00010016547	0.985098013	0.338582461
ENSGALG00010016548	0.56214221	0.258370802
ENSGALG00010016549	0.710456665	0.060116041
ENSGALG00010016550	0.250632286	0.235575858
ENSGALG00010016551	0.881264796	0.27532896
ENSGALG00010016552	0.694576094	0.033272137
ENSGALG00010016553	0.885120581	0.377270302
ENSGALG00010016556	0.53141527	0.221578146
ENSGALG00010016557	0.300090076	0.087382432
ENSGALG00010016558	0.953344734	0.336245878
ENSGALG00010016559	0.058635451	0.160532315
ENSGALG00010016560	0.309016749	0.315540562
ENSGALG00010016561	0.877073002	0.293106548
ENSGALG00010016562	0.715953429	0.204952655
ENSGALG00010016563	0.461467044	0.356250549
ENSGALG00010016564	0.812139722	0.10755887
ENSGALG00010016565	0.310725139	0.099441788
ENSGALG00010016566	0.736745201	0.141127406
ENSGALG00010016567	0.560456957	0.453324587

ENSGALG00010016568	0.882482381	0.337282563
ENSGALG00010016569	0.804747196	0.165804373
ENSGALG00010016570	0.83386181	0.249792199
ENSGALG00010016571	0.936726345	0.310842321
ENSGALG00010016572	0.301926858	0.122797728
ENSGALG00010016573	0.919320135	0.184463928
ENSGALG00010016574	0.885581262	0.273577655
ENSGALG00010016575	0.67460584	0.031313618
ENSGALG00010016576	0.953530221	0.370217866
ENSGALG00010016577	0.930855197	0.378762141
ENSGALG00010016580	0.750871772	0.313470343
ENSGALG00010016581	0.916539266	0.301818383
ENSGALG00010016582	0.61940917	0.459696796
ENSGALG00010016583	0.811843632	0.27103607
ENSGALG00010016584	0.049369938	0.02962301
ENSGALG00010016585	0.327395037	0.23559956
ENSGALG00010016587	0.435557604	0.225613862
ENSGALG00010016588	0.750038545	0.393414246
ENSGALG00010016589	0.756364287	0.306563381
ENSGALG00010016591	0.888157647	0.26923305
ENSGALG00010016592	0.958426023	0.245558253
ENSGALG00010016594	0.28293882	0.145057094
ENSGALG00010016595	0.843276313	0.197197274
ENSGALG00010016596	0.700178844	0.406463411
ENSGALG00010016597	0.918305647	0.293168533
ENSGALG00010016598	0.556902314	0.021716284
ENSGALG00010016599	0.968928938	0.424319134
ENSGALG00010016600	0.30046948	0.15279235
ENSGALG00010016601	0.608695829	0.073457235
ENSGALG00010016602	0.904927368	0.248839307
ENSGALG00010016603	0.939570344	0.294134824
ENSGALG00010016604	0.882501913	0.22590417
ENSGALG00010016605	0.688577888	0.276892575
ENSGALG00010016606	0.350108996	0.183548795
ENSGALG00010016607	0.432193539	0.022113042
ENSGALG00010016608	0.467211485	0.039034571
ENSGALG00010016610	0.318072251	0.131989285
ENSGALG00010016611	0.01407268	0.098936353
ENSGALG00010016612	0.64970217	0.003559266
ENSGALG00010016613	0.491922195	0.024012954
ENSGALG00010016614	0.903049504	0.315258163
ENSGALG00010016615	0.468012111	0.073424383
ENSGALG00010016616	0.851567204	0.360451014
ENSGALG00010016617	0.028008735	0.138260234
ENSGALG00010016618	0.821000809	0.223074681
ENSGALG00010016619	0.966698766	0.325031066
ENSGALG00010016620	0.03159394	0.023468385
ENSGALG00010016621	0.378132611	0.75278282
ENSGALG00010016622	0.391106478	0.144472949
ENSGALG00010016623	0.945787492	0.240967037
ENSGALG00010016624	0.344448068	0.062309616
ENSGALG00010016625	0.011918622	0.249719435
ENSGALG00010016626	0.556759714	0.463423328
ENSGALG00010016627	0.485455767	0.729266223
ENSGALG00010016628	0.301772951	0.966506966
ENSGALG00010016629	0.701057901	0.253429413

ENSGALG00010016630	0.726294725	0.14019815
ENSGALG00010016631	0.967789148	0.326024047
ENSGALG00010016634	0.491151149	0.232069802
ENSGALG00010016636	0.629005897	0.057699845
ENSGALG00010016637	0.402384438	0.115161426
ENSGALG00010016638	0.790221493	0.283737521
ENSGALG00010016639	0.749750882	0.249939547
ENSGALG00010016640	0.705810092	0.468188252
ENSGALG00010016641	0.242697762	0.046307277
ENSGALG00010016643	0.990327268	0.314616729
ENSGALG00010016644	0.945507095	0.274466124
ENSGALG00010016645	0.200570274	0.091667991
ENSGALG00010016646	0.385953062	0.133261917
ENSGALG00010016647	0.466033185	0.003228921
ENSGALG00010016648	0.825346991	0.312829897
ENSGALG00010016651	0.945522787	0.260912958
ENSGALG00010016652	0.993435734	0.316704328
ENSGALG00010016653	0.309178419	0.206984408
ENSGALG00010016654	0.51666812	0.190115639
ENSGALG00010016655	0.943124751	0.325792946
ENSGALG00010016657	0.950106302	0.313762608
ENSGALG00010016658	0.487905955	0.038988181
ENSGALG00010016659	0.134070044	0.223921099
ENSGALG00010016661	0.709855411	0.333598734
ENSGALG00010016663	0.867910052	0.049783568
ENSGALG00010016664	0.585721903	0.36100014
ENSGALG00010016665	0.973568724	0.283866846
ENSGALG00010016666	0.635933338	0.029686833
ENSGALG00010016667	0.099441885	0.153915144
ENSGALG00010016670	0.900679879	0.393640299
ENSGALG00010016671	0.299518609	0.069647191
ENSGALG00010016672	0.973048558	0.37554206
ENSGALG00010016673	0.958967256	0.18939979
ENSGALG00010016674	0.697148467	0.049520887
ENSGALG00010016675	0.917938164	0.269637059
ENSGALG00010016676	0.949414213	0.335981051
ENSGALG00010016677	0.815839008	0.140603521
ENSGALG00010016678	0.896833507	0.268866405
ENSGALG00010016679	0.49906172	0.179357039
ENSGALG00010016680	0.264239718	0.073427965
ENSGALG00010016681	0.839005403	0.282282276
ENSGALG00010016682	0.982477654	0.373579728
ENSGALG00010016683	0.575703183	0.069100181
ENSGALG00010016684	0.396108191	0.15301941
ENSGALG00010016686	0.205170479	0.096085038
ENSGALG00010016687	0.256828349	0.119064955
ENSGALG00010016688	0.872200858	0.348506558
ENSGALG00010016690	0.26826433	0.16644847
ENSGALG00010016691	0.384262585	0.091757058
ENSGALG00010016692	0.842292962	0.412463321
ENSGALG00010016694	0.043659357	0.108092216
ENSGALG00010016695	0.678893091	0.353375183
ENSGALG00010016696	0.981683481	0.291579971
ENSGALG00010016699	0.997055028	0.342515793
ENSGALG00010016700	0.047704397	0.244258012
ENSGALG00010016701	0.573126974	0.001411343

ENSGALG00010016702	0.514000577	0.061815838
ENSGALG00010016703	0.366395198	0.115102176
ENSGALG00010016704	0.392868622	0.196336235
ENSGALG00010016706	0.982205006	0.350468932
ENSGALG00010016707	0.983473132	0.295110017
ENSGALG00010016708	0.573474405	0.293507608
ENSGALG00010016709	0.414402319	0.043916107
ENSGALG00010016710	0.296648799	0.034280929
ENSGALG00010016711	0.409911588	0.349233755
ENSGALG00010016712	0.925265163	0.306592916
ENSGALG00010016713	0.926930366	0.140404895
ENSGALG00010016714	0.317727434	0.146443343
ENSGALG00010016715	0.671772924	0.245611548
ENSGALG00010016717	0.669391596	0.294152317
ENSGALG00010016718	0.391919077	0.131677909
ENSGALG00010016719	0.310438704	0.069044687
ENSGALG00010016720	0.718833003	0.497949515
ENSGALG00010016722	0.710763355	0.326537428
ENSGALG00010016724	0.949321755	0.23239435
ENSGALG00010016729	0.679971718	0.247187241
ENSGALG00010016730	0.902010039	0.306129355
ENSGALG00010016731	0.878383862	0.117979299
ENSGALG00010016732	0.129757092	0.312745481
ENSGALG00010016733	0.393157339	0.241151433
ENSGALG00010016734	0.142087934	0.18257098
ENSGALG00010016735	0.744336755	0.37851223
ENSGALG00010016736	0.508679709	0.032621771
ENSGALG00010016738	0.958238126	0.304941313
ENSGALG00010016739	0.518638572	0.273046581
ENSGALG00010016741	0.800306187	0.250435394
ENSGALG00010016742	0.383642761	0.158269652
ENSGALG00010016743	0.14997172	0.177400817
ENSGALG00010016744	0.507817099	0.166841031
ENSGALG00010016745	0.984360867	0.275678078
ENSGALG00010016746	0.080864191	0.503836742
ENSGALG00010016747	0.299979544	0.223068876
ENSGALG00010016748	0.153327401	0.262227393
ENSGALG00010016750	0.741152762	0.344279312
ENSGALG00010016751	0.269500008	0.205458342
ENSGALG00010016752	0.895256182	0.307452145
ENSGALG00010016753	0.866904182	0.224485041
ENSGALG00010016754	0.872249898	0.105374166
ENSGALG00010016756	0.994383023	0.346969213
ENSGALG00010016757	0.863261458	0.356445633
ENSGALG00010016758	0.631885073	0.361615615
ENSGALG00010016760	0.97215907	0.249598504
ENSGALG00010016761	0.943723549	0.307223018
ENSGALG00010016762	0.880918027	0.453625587
ENSGALG00010016763	0.971049076	0.363930915
ENSGALG00010016765	0.060830804	0.115976931
ENSGALG00010016766	0.977719727	0.251226335
ENSGALG00010016767	0.990498087	0.300515256
ENSGALG00010016768	0.724835659	0.449339628
ENSGALG00010016769	0.204125996	0.170747153
ENSGALG00010016770	0.372520634	0.173408192
ENSGALG00010016771	0.575267436	0.072504719

ENSGALG00010016772	0.429724711	0.003125211
ENSGALG00010016773	0.919158932	0.31542703
ENSGALG00010016774	0.845306592	0.150551371
ENSGALG00010016776	0.25368922	0.213271183
ENSGALG00010016777	0.711792285	0.317755922
ENSGALG00010016778	0.90073249	0.19841052
ENSGALG00010016779	0.931392834	0.264495815
ENSGALG00010016780	0.069598168	0.039760573
ENSGALG00010016781	0.997219035	0.345963924
ENSGALG00010016782	0.547597802	0.097172401
ENSGALG00010016783	0.098489538	0.318453401
ENSGALG00010016784	0.837342833	0.231850237
ENSGALG00010016785	0.539165004	0.342717647
ENSGALG00010016786	0.794118343	0.123712563
ENSGALG00010016787	0.9622098	0.28235594
ENSGALG00010016788	0.47124279	0.000788952
ENSGALG00010016789	0.945268366	0.215620406
ENSGALG00010016792	0.935678755	0.299848485
ENSGALG00010016793	0.984666096	0.310133321
ENSGALG00010016795	0.963508682	0.334824344
ENSGALG00010016796	0.879139646	0.314506946
ENSGALG00010016797	0.976982633	0.384998471
ENSGALG00010016798	0.840200328	0.318078468
ENSGALG00010016799	0.965255776	0.237143281
ENSGALG00010016800	0.150337883	0.101391765
ENSGALG00010016801	0.969940114	0.323593474
ENSGALG00010016802	0.871687701	0.170289206
ENSGALG00010016803	0.628639671	0.089767857
ENSGALG00010016804	0.977177885	0.320919621
ENSGALG00010016805	0.325621802	0.13143486
ENSGALG00010016806	0.508940138	0.050703399
ENSGALG00010016807	0.993233173	0.317981347
ENSGALG00010016808	0.243003602	0.209494585
ENSGALG00010016809	0.740411785	0.56739181
ENSGALG00010016810	0.804187226	0.404845945
ENSGALG00010016812	0.750956071	0.39463898
ENSGALG00010016813	0.922319226	0.297237029
ENSGALG00010016814	0.040465578	0.015829063
ENSGALG00010016815	0.127220573	0.433349718
ENSGALG00010016816	0.628939454	0.275635417
ENSGALG00010016817	0.866532272	0.171472643
ENSGALG00010016818	0.691809599	0.116664272
ENSGALG00010016820	0.989174562	0.281924388
ENSGALG00010016821	0.10375347	0.066576005
ENSGALG00010016823	0.307357231	0.20020637
ENSGALG00010016825	0.987623827	0.332398239
ENSGALG00010016828	0.986755525	0.382069997
ENSGALG00010016831	0.865367626	0.325740472
ENSGALG00010016832	0.552375923	0.081141804
ENSGALG00010016834	0.868958795	0.175528044
ENSGALG00010016835	0.813808459	0.290208924
ENSGALG00010016836	0.80435934	0.437253665
ENSGALG00010016838	0.922410817	0.327356125
ENSGALG00010016840	0.939811126	0.297735516
ENSGALG00010016841	0.011589493	0.110944875
ENSGALG00010016842	0.260523852	0.32016603

ENSGALG00010016843	0.739812253	0.392050831
ENSGALG00010016845	0.963496666	0.300631823
ENSGALG00010016847	0.938178874	0.20429919
ENSGALG00010016848	0.11586867	0.103637016
ENSGALG00010016849	0.926772028	0.341631176
ENSGALG00010016850	0.519843462	0.314613684
ENSGALG00010016851	0.366271267	0.111713436
ENSGALG00010016852	0.963986542	0.315072204
ENSGALG00010016853	0.508772524	0.248804662
ENSGALG00010016854	0.15695072	0.002337982
ENSGALG00010016855	0.775301723	0.277813169
ENSGALG00010016856	0.957965463	0.28002331
ENSGALG00010016858	0.583544465	0.3984062
ENSGALG00010016859	0.301366939	0.546014643
ENSGALG00010016860	0.408149558	0.262548722
ENSGALG00010016861	0.541748048	0.082162559
ENSGALG00010016862	0.821433198	0.560636355
ENSGALG00010016863	0.968278859	0.294174979
ENSGALG00010016864	0.236836204	0.236412506
ENSGALG00010016865	0.935902199	0.371588786
ENSGALG00010016866	0.986446958	0.350360787
ENSGALG00010016867	0.705004197	0.198861639
ENSGALG00010016868	0.799308067	0.168536485
ENSGALG00010016869	0.089760662	0.103921741
ENSGALG00010016870	0.37023851	0.413234014
ENSGALG00010016872	0.879239974	0.306827925
ENSGALG00010016873	0.933014815	0.026776609
ENSGALG00010016874	0.905139139	0.284601311
ENSGALG00010016875	0.850832617	0.160498435
ENSGALG00010016876	0.852962447	0.104413585
ENSGALG00010016877	0.938225167	0.212759573
ENSGALG00010016878	0.988410145	0.344066602
ENSGALG00010016879	0.894944195	0.186040265
ENSGALG00010016880	0.87521319	0.074867914
ENSGALG00010016881	0.974611107	0.295332913
ENSGALG00010016882	0.952154552	0.332281318
ENSGALG00010016883	0.931454214	0.106005368
ENSGALG00010016884	0.96913974	0.346892086
ENSGALG00010016885	0.472663836	0.118491751
ENSGALG00010016887	0.954304469	0.291248297
ENSGALG00010016888	0.605612193	0.213034196
ENSGALG00010016889	0.980186175	0.336255172
ENSGALG00010016890	0.062763256	0.13762019
ENSGALG00010016891	0.428321792	0.072217874
ENSGALG00010016892	0.097404455	0.078746816
ENSGALG00010016893	0.80905541	0.385982379
ENSGALG00010016894	0.558607002	0.140187754
ENSGALG00010016895	0.26909056	0.238139731
ENSGALG00010016896	0.70191657	0.131458763
ENSGALG00010016897	0.42301224	0.007930546
ENSGALG00010016899	0.917544676	0.331321433
ENSGALG00010016900	0.85183972	0.333368725
ENSGALG00010016901	0.413488808	0.258811614
ENSGALG00010016902	0.397793307	0.136785604
ENSGALG00010016903	0.935741086	0.258991616
ENSGALG00010016904	0.878858347	0.183048273

ENSGALG00010016905	0.959121213	0.262133416
ENSGALG00010016907	0.433538767	0.193137182
ENSGALG00010016908	0.969713731	0.267303135
ENSGALG00010016909	0.575987681	0.265489419
ENSGALG00010016910	0.843720946	0.251189013
ENSGALG00010016911	0.861041085	0.251620061
ENSGALG00010016912	0.837430266	0.215618109
ENSGALG00010016913	0.396699027	0.226585893
ENSGALG00010016914	0.209038957	0.052196575
ENSGALG00010016915	0.921221098	0.223349214
ENSGALG00010016916	0.995814823	0.317131713
ENSGALG00010016917	0.877614222	0.155846822
ENSGALG00010016918	0.825516097	0.243014578
ENSGALG00010016919	0.841267191	0.284123927
ENSGALG00010016920	0.981593459	0.250943822
ENSGALG00010016921	0.345491692	0.482412701
ENSGALG00010016922	0.293985649	0.453685798
ENSGALG00010016923	0.673928746	0.269819078
ENSGALG00010016924	0.261073952	0.119572608
ENSGALG00010016925	0.659178297	0.196298564
ENSGALG00010016926	0.040232552	0.433250674
ENSGALG00010016927	0.875886292	0.247358975
ENSGALG00010016928	0.759869117	0.311999838
ENSGALG00010016929	0.304978835	0.12191445
ENSGALG00010016930	0.901307395	0.288430031
ENSGALG00010016931	0.618596893	0.175370641
ENSGALG00010016932	0.809176524	0.357633817
ENSGALG00010016933	0.717821057	0.064290785
ENSGALG00010016934	0.333982889	0.287104687
ENSGALG00010016935	0.951254361	0.303486359
ENSGALG00010016936	0.956628296	0.326197408
ENSGALG00010016937	0.994149039	0.296882601
ENSGALG00010016938	0.303508198	0.409726521
ENSGALG00010016939	0.981267817	0.267769635
ENSGALG00010016940	0.98066465	0.363456354
ENSGALG00010016941	0.8193455	0.138167874
ENSGALG00010016942	0.478605851	0.139358175
ENSGALG00010016943	0.799691273	0.141719483
ENSGALG00010016944	0.050564	0.120729262
ENSGALG00010016945	0.972668466	0.42869222
ENSGALG00010016946	0.992864443	0.286526798
ENSGALG00010016947	0.983569886	0.250934987
ENSGALG00010016948	0.7690025	0.183408986
ENSGALG00010016949	0.730103411	0.287986403
ENSGALG00010016950	0.737986284	0.314879896
ENSGALG00010016951	0.975661399	0.36047349
ENSGALG00010016952	0.53223597	0.286650162
ENSGALG00010016953	0.111935284	0.074526226
ENSGALG00010016955	0.691443501	0.40963159
ENSGALG00010016956	0.778923712	0.272994589
ENSGALG00010016957	0.822529149	0.228493929
ENSGALG00010016958	0.624798952	0.150568029
ENSGALG00010016959	0.655725183	0.274846711
ENSGALG00010016960	0.372397461	0.026267902
ENSGALG00010016961	0.335891967	0.16849738
ENSGALG00010016962	0.038871183	0.142553515

ENSGALG00010016963	0.83220226	0.350073296
ENSGALG00010016964	0.943472601	0.290675908
ENSGALG00010016965	0.880070373	0.303367895
ENSGALG00010016966	0.564782563	0.0182566
ENSGALG00010016967	0.906638395	0.367617469
ENSGALG00010016968	0.974805828	0.32160973
ENSGALG00010016969	0.9832842	0.322495601
ENSGALG00010016970	0.854191145	0.275688022
ENSGALG00010016971	0.369981499	0.264773568
ENSGALG00010016972	0.007814858	0.127811066
ENSGALG00010016973	0.652978782	0.214261526
ENSGALG00010016974	0.380793985	0.176306365
ENSGALG00010016975	0.049882549	0.26275067
ENSGALG00010016976	0.819607822	0.140042749
ENSGALG00010016977	0.038903348	0.022762323
ENSGALG00010016978	0.929181227	0.223094329
ENSGALG00010016979	0.383947044	0.128562519
ENSGALG00010016980	0.539128446	0.097974642
ENSGALG00010016981	0.90069054	0.257953683
ENSGALG00010016982	0.621994765	0.100279203
ENSGALG00010016983	0.227838373	0.034538273
ENSGALG00010016984	0.762347793	0.388360272
ENSGALG00010016985	0.174722339	0.063496408
ENSGALG00010016986	0.900781877	0.226077957
ENSGALG00010016987	0.916033639	0.224496849
ENSGALG00010016988	0.886154524	0.184528031
ENSGALG00010016989	0.769425681	0.158803806
ENSGALG00010016991	0.989411723	0.314479491
ENSGALG00010016992	0.825957283	0.18154777
ENSGALG00010016993	0.935799236	0.255404475
ENSGALG00010016994	0.906767113	0.257519394
ENSGALG00010016995	0.938208151	0.346502951
ENSGALG00010016996	0.684801254	0.358099128
ENSGALG00010016998	0.874211603	0.125822869
ENSGALG00010016999	0.992755324	0.355441538
ENSGALG00010017000	0.04875482	0.180301697
ENSGALG00010017001	0.1711804	0.032623259
ENSGALG00010017002	0.765747031	0.131076514
ENSGALG00010017003	0.829600291	0.419051577
ENSGALG00010017004	0.968851849	0.195787315
ENSGALG00010017006	0.785999679	0.157856966
ENSGALG00010017008	0.961707637	0.33627684
ENSGALG00010017010	0.314312913	0.300052519
ENSGALG00010017011	0.638074004	0.078030583
ENSGALG00010017012	0.98911454	0.34977737
ENSGALG00010017013	0.44682585	0.150075723
ENSGALG00010017014	0.057863266	0.091469403
ENSGALG00010017015	0.247518097	0.193646827
ENSGALG00010017016	0.391395104	0.158003851
ENSGALG00010017017	0.315512486	0.171285599
ENSGALG00010017018	0.918619161	0.192632019
ENSGALG00010017019	0.519431689	0.204839096
ENSGALG00010017020	0.024459739	0.181034195
ENSGALG00010017021	0.400215036	0.132215663
ENSGALG00010017022	0.640378443	0.001786785
ENSGALG00010017024	0.75267692	0.211839103

ENSGALG00010017027	0.760085585	0.03851519
ENSGALG00010017029	0.77289795	0.297203314
ENSGALG00010017030	0.843574735	0.542718072
ENSGALG00010017032	0.341811318	0.527341176
ENSGALG00010017033	0.987047516	0.354450652
ENSGALG00010017034	0.437738318	0.0978347
ENSGALG00010017035	0.989159422	0.298333031
ENSGALG00010017038	0.214668377	0.322849606
ENSGALG00010017039	0.715360868	0.136424808
ENSGALG00010017040	0.02337546	0.15959425
ENSGALG00010017041	0.6763219	0.323997603
ENSGALG00010017042	0.884956926	0.311429356
ENSGALG00010017043	0.030686825	0.45212583
ENSGALG00010017044	0.424270929	0.173825238
ENSGALG00010017045	0.922804057	0.156073028
ENSGALG00010017046	0.31068245	0.189010659
ENSGALG00010017047	0.481627181	0.371195624
ENSGALG00010017049	0.80038872	0.427430561
ENSGALG00010017050	0.416138999	0.068251873
ENSGALG00010017052	0.09769597	0.256883266
ENSGALG00010017053	0.865336018	0.31888527
ENSGALG00010017054	0.963266199	0.302569013
ENSGALG00010017055	0.58452392	0.142293925
ENSGALG00010017056	0.872404848	0.355014021
ENSGALG00010017057	0.807139682	0.313271006
ENSGALG00010017058	0.353306567	0.159752261
ENSGALG00010017060	0.896256421	0.34765607
ENSGALG00010017061	0.850053312	0.395320683
ENSGALG00010017062	0.142503903	0.100429809
ENSGALG00010017063	0.939954766	0.196281564
ENSGALG00010017065	0.753820638	0.293191775
ENSGALG00010017068	0.490560405	0.360798996
ENSGALG00010017070	0.88190452	0.298591427
ENSGALG00010017071	0.628974201	0.121025125
ENSGALG00010017073	0.862574511	0.267955868
ENSGALG00010017074	0.825933196	0.306437124
ENSGALG00010017075	0.782860247	0.408480981
ENSGALG00010017077	0.957571212	0.356771632
ENSGALG00010017078	0.035387674	0.035601977
ENSGALG00010017079	0.530752511	0.347460603
ENSGALG00010017080	0.566077628	0.085889457
ENSGALG00010017083	0.76081873	0.23090358
ENSGALG00010017085	0.960311071	0.324404374
ENSGALG00010017088	0.29339099	0.433285595
ENSGALG00010017089	0.743788695	0.387703333
ENSGALG00010017090	0.009876626	0.286589717
ENSGALG00010017091	0.598669277	0.113971987
ENSGALG00010017092	0.509804734	0.130051195
ENSGALG00010017093	0.387408492	0.038314914
ENSGALG00010017094	0.989787508	0.303168976
ENSGALG00010017095	0.291895501	0.15301281
ENSGALG00010017096	0.409636587	0.55843813
ENSGALG00010017097	0.568901522	0.167200803
ENSGALG00010017098	0.943033355	0.196380806
ENSGALG00010017100	0.103884623	0.06798884
ENSGALG00010017101	0.444823664	0.397613018

ENSGALG00010017102	0.034007678	0.115333164
ENSGALG00010017103	0.451553087	0.120227008
ENSGALG00010017104	0.877096434	0.169556566
ENSGALG00010017105	0.589354346	0.125715389
ENSGALG00010017106	0.935088281	0.132292438
ENSGALG00010017109	0.969575399	0.329036556
ENSGALG00010017110	0.508080511	0.202321484
ENSGALG00010017111	0.990457433	0.273121359
ENSGALG00010017112	0.945348492	0.233026638
ENSGALG00010017114	0.094214503	0.128734632
ENSGALG00010017115	0.962180077	0.331301648
ENSGALG00010017116	0.193019462	0.153232988
ENSGALG00010017118	0.263548636	0.341804522
ENSGALG00010017119	0.96411524	0.326611604
ENSGALG00010017120	0.911170949	0.320854435
ENSGALG00010017121	0.144103871	0.025345762
ENSGALG00010017122	0.299652785	0.028471245
ENSGALG00010017124	0.271701261	0.072417625
ENSGALG00010017125	0.213450411	0.147848948
ENSGALG00010017126	0.987715877	0.233592136
ENSGALG00010017127	0.943489541	0.341820503
ENSGALG00010017128	0.470617807	0.148833615
ENSGALG00010017129	0.671896127	0.424436859
ENSGALG00010017131	0.967281949	0.371788515
ENSGALG00010017132	0.106358205	0.106765945
ENSGALG00010017133	0.76317893	0.497772402
ENSGALG00010017134	0.270451009	0.090541667
ENSGALG00010017135	0.315392201	0.038781939
ENSGALG00010017136	0.467966181	0.316061604
ENSGALG00010017137	0.269272437	0.073182666
ENSGALG00010017138	0.467018431	0.073431361
ENSGALG00010017139	0.810796383	0.270589464
ENSGALG00010017140	0.673767869	0.296218992
ENSGALG00010017141	0.953032091	0.452358481
ENSGALG00010017142	0.97098173	0.280271555
ENSGALG00010017143	0.977245418	0.29301184
ENSGALG00010017144	0.321440542	0.080101054
ENSGALG00010017145	0.580402188	0.222866202
ENSGALG00010017147	0.544232311	0.530895175
ENSGALG00010017148	0.481271616	0.133297069
ENSGALG00010017149	0.384897716	0.112786777
ENSGALG00010017150	0.61482352	0.050520512
ENSGALG00010017151	0.932038788	0.443682016
ENSGALG00010017152	0.991130202	0.289269534
ENSGALG00010017153	0.437103749	0.012674061
ENSGALG00010017154	0.512194375	0.240993356
ENSGALG00010017156	0.817053423	0.548260608
ENSGALG00010017157	0.397860288	0.045886469
ENSGALG00010017158	0.530324799	0.19678275
ENSGALG00010017159	0.407680041	0.161965077
ENSGALG00010017160	0.073376936	0.164024345
ENSGALG00010017161	0.986028162	0.334584006
ENSGALG00010017162	0.506323298	0.027914306
ENSGALG00010017163	0.378406323	0.485627561
ENSGALG00010017164	0.270451009	0.090541667
ENSGALG00010017165	0.982770543	0.329091923

ENSGALG00010017166	0.296272339	0.491563762
ENSGALG00010017167	0.916535754	0.245382473
ENSGALG00010017169	0.444741259	0.214470928
ENSGALG00010017172	0.197063832	0.359582983
ENSGALG00010017174	0.803126494	0.261058663
ENSGALG00010017176	0.115511175	0.303927215
ENSGALG00010017180	0.112526779	0.034368165
ENSGALG00010017182	0.320729011	0.144028995
ENSGALG00010017183	0.319432253	0.226386267
ENSGALG00010017184	0.917154207	0.128417488
ENSGALG00010017185	0.981706144	0.242217884
ENSGALG00010017186	0.767318044	0.36455075
ENSGALG00010017187	0.775733273	0.274588852
ENSGALG00010017188	0.722903729	0.133379687
ENSGALG00010017190	0.942085453	0.469959499
ENSGALG00010017192	0.556171307	0.201218857
ENSGALG00010017193	0.959070523	0.336832616
ENSGALG00010017195	0.682426665	0.075555835
ENSGALG00010017197	0.026501066	0.052690148
ENSGALG00010017198	0.607433668	0.05925068
ENSGALG00010017199	0.516484792	0.358831355
ENSGALG00010017200	0.232006413	0.261085152
ENSGALG00010017201	0.846961243	0.159211053
ENSGALG00010017202	0.309031157	0.31725819
ENSGALG00010017204	0.743496993	0.321518133
ENSGALG00010017205	0.737692653	0.282996941
ENSGALG00010017206	0.359924451	0.169240891
ENSGALG00010017207	0.983322909	0.331107836
ENSGALG00010017208	0.915940232	0.261599654
ENSGALG00010017209	0.634144537	0.033246729
ENSGALG00010017211	0.982829362	0.293553473
ENSGALG00010017212	0.556973957	0.42524659
ENSGALG00010017214	0.535583747	0.068695955
ENSGALG00010017215	0.895650475	0.237653089
ENSGALG00010017216	0.051606007	0.145348651
ENSGALG00010017218	0.261326278	0.156713752
ENSGALG00010017219	0.679258023	0.199507063
ENSGALG00010017221	0.753390742	0.219758739
ENSGALG00010017222	0.685350045	0.062524292
ENSGALG00010017223	0.344391634	0.119875951
ENSGALG00010017224	0.557976166	0.031252929
ENSGALG00010017225	0.19754544	0.177927375
ENSGALG00010017226	0.620712866	0.448020442
ENSGALG00010017227	0.063683658	0.087267703
ENSGALG00010017228	0.486363446	0.091558152
ENSGALG00010017229	0.698194684	0.37643124
ENSGALG00010017230	0.834408618	0.31087318
ENSGALG00010017231	0.771222549	0.380689665
ENSGALG00010017234	0.511974447	0.015653456
ENSGALG00010017235	0.345957381	0.377989906
ENSGALG00010017237	0.60987391	0.023802721
ENSGALG00010017240	0.91763033	0.242041905
ENSGALG00010017246	0.371767508	0.345509902
ENSGALG00010017248	0.918483714	0.163505619
ENSGALG00010017250	0.683330431	0.238568257
ENSGALG00010017251	0.301952912	0.225538146

ENSGALG00010017252	0.032247106	0.068242938
ENSGALG00010017253	0.176223345	0.19443888
ENSGALG00010017254	0.366688394	0.154271198
ENSGALG00010017255	0.765714645	0.232919177
ENSGALG00010017256	0.966264253	0.330536478
ENSGALG00010017258	0.248794789	0.225810363
ENSGALG00010017260	0.601786635	0.200707696
ENSGALG00010017262	0.404167701	0.144831306
ENSGALG00010017263	0.809376506	0.256910798
ENSGALG00010017264	0.659119463	0.345494699
ENSGALG00010017265	0.659171246	0.180780359
ENSGALG00010017266	0.974595784	0.413099561
ENSGALG00010017270	0.812789995	0.365867978
ENSGALG00010017272	0.594682544	0.487032014
ENSGALG00010017274	0.69437292	0.331690821
ENSGALG00010017276	0.378386997	0.131934372
ENSGALG00010017278	0.904639044	0.434135633
ENSGALG00010017280	0.808337507	0.214695305
ENSGALG00010017281	0.882825502	0.170032239
ENSGALG00010017283	0.994246313	0.312288348
ENSGALG00010017284	0.920837602	0.200139121
ENSGALG00010017285	0.946061944	0.320973721
ENSGALG00010017286	0.732712798	0.206011873
ENSGALG00010017289	0.464411218	0.028446036
ENSGALG00010017290	0.536339396	0.239070185
ENSGALG00010017292	0.665617349	0.199598922
ENSGALG00010017293	0.946970157	0.321864591
ENSGALG00010017294	0.406377991	0.538354817
ENSGALG00010017295	0.379098883	0.108694718
ENSGALG00010017296	0.920971328	0.306976366
ENSGALG00010017297	0.97522469	0.260199781
ENSGALG00010017299	0.360546224	0.12646624
ENSGALG00010017300	0.982723164	0.418893443
ENSGALG00010017301	0.387718767	0.276994253
ENSGALG00010017304	0.972186977	0.332602035
ENSGALG00010017305	0.512921639	0.130657483
ENSGALG00010017306	0.531961048	0.261320476
ENSGALG00010017307	0.429829999	0.32596791
ENSGALG00010017308	0.560966986	0.10688555
ENSGALG00010017309	0.405797923	0.13200104
ENSGALG00010017310	0.703570757	0.111619771
ENSGALG00010017311	0.064841871	0.131229091
ENSGALG00010017312	0.985896304	0.238987485
ENSGALG00010017313	0.394312958	0.021415714
ENSGALG00010017314	0.42580478	0.041827627
ENSGALG00010017315	0.199488819	0.094321839
ENSGALG00010017318	0.994811544	0.304092362
ENSGALG00010017319	0.665868481	0.052453343
ENSGALG00010017320	0.707425468	0.416157882
ENSGALG00010017322	0.877931258	0.295168997
ENSGALG00010017323	0.563856724	0.383239953
ENSGALG00010017325	0.667054751	0.070901223
ENSGALG00010017326	0.781053172	0.29811852
ENSGALG00010017327	0.972086644	0.27877666
ENSGALG00010017330	0.823410408	0.124341464
ENSGALG00010017332	0.969389104	0.343733432

ENSGALG00010017334	0.332399589	0.113427957
ENSGALG00010017335	0.00014904	0.169563208
ENSGALG00010017337	0.719188789	0.454637804
ENSGALG00010017338	0.873508957	0.129879469
ENSGALG00010017339	0.884131246	0.294913322
ENSGALG00010017340	0.888574956	0.296296337
ENSGALG00010017341	0.361887629	0.128498832
ENSGALG00010017342	0.473033189	0.126932614
ENSGALG00010017343	0.136662935	0.211990407
ENSGALG00010017344	0.989821448	0.278122315
ENSGALG00010017345	0.187514554	0.108479243
ENSGALG00010017346	0.957553317	0.286725704
ENSGALG00010017347	0.447755468	0.081634798
ENSGALG00010017349	0.021527755	0.295737633
ENSGALG00010017350	0.990661515	0.345211731
ENSGALG00010017351	0.978744348	0.318900061
ENSGALG00010017352	0.592757704	0.725010621
ENSGALG00010017354	0.834344857	0.248541475
ENSGALG00010017355	0.352478527	0.272458233
ENSGALG00010017356	0.432188719	0.078920009
ENSGALG00010017358	0.493431099	0.187927311
ENSGALG00010017359	0.708997671	0.335532128
ENSGALG00010017361	0.920003906	0.243506542
ENSGALG00010017362	0.904721281	0.263152387
ENSGALG00010017363	0.202692128	0.133815772
ENSGALG00010017364	0.966616505	0.340438978
ENSGALG00010017365	0.962505566	0.227189102
ENSGALG00010017367	0.97752721	0.333947477
ENSGALG00010017368	0.323514001	0.068799813
ENSGALG00010017369	0.023500597	0.155340452
ENSGALG00010017372	0.907596102	0.276107056
ENSGALG00010017373	0.433271604	0.089781439
ENSGALG00010017374	0.263768666	0.29094256
ENSGALG00010017375	0.967239052	0.376690128
ENSGALG00010017376	0.656914507	0.020361484
ENSGALG00010017377	0.55122186	0.141301778
ENSGALG00010017378	0.732332648	0.502177995
ENSGALG00010017380	0.095322368	0.088460471
ENSGALG00010017381	0.620313372	0.285608351
ENSGALG00010017382	0.727815022	0.331252355
ENSGALG00010017383	0.4479925	0.382197937
ENSGALG00010017384	0.966419215	0.256783441
ENSGALG00010017385	0.088179649	0.287182419
ENSGALG00010017386	0.645801469	0.300576002
ENSGALG00010017387	0.817260138	0.251254787
ENSGALG00010017388	0.404493179	0.072574432
ENSGALG00010017389	0.086741006	0.127231402
ENSGALG00010017390	0.870465699	0.195336288
ENSGALG00010017391	0.812451483	0.291692453
ENSGALG00010017393	0.984657341	0.259786319
ENSGALG00010017394	0.207045062	0.119048488
ENSGALG00010017395	0.927813135	0.260015142
ENSGALG00010017396	0.928377753	0.335817001
ENSGALG00010017398	0.990682009	0.34273316
ENSGALG00010017399	0.802944571	0.406745471
ENSGALG00010017400	0.876469575	0.313881962

ENSGALG00010017402	0.960456275	0.451191997
ENSGALG00010017403	0.553833062	0.40146269
ENSGALG00010017405	0.958820022	0.209284133
ENSGALG00010017406	0.589687944	0.273079133
ENSGALG00010017407	0.359986475	0.089989034
ENSGALG00010017408	0.264239718	0.073427965
ENSGALG00010017409	0.635519237	0.447570536
ENSGALG00010017411	0.870821551	0.240572436
ENSGALG00010017413	0.203991683	0.058221925
ENSGALG00010017414	0.934286354	0.360636402
ENSGALG00010017415	0.975332708	0.316050963
ENSGALG00010017418	0.482621303	0.03737141
ENSGALG00010017419	0.517001087	0.087503678
ENSGALG00010017421	0.833975918	0.224846675
ENSGALG00010017422	0.622881316	0.233174266
ENSGALG00010017423	0.873852591	0.216238696
ENSGALG00010017424	0.935717287	0.244311919
ENSGALG00010017425	0.985672771	0.327252829
ENSGALG00010017428	0.936081048	0.292815846
ENSGALG00010017429	0.802333447	0.346704523
ENSGALG00010017430	0.397406754	0.190153304
ENSGALG00010017431	0.926731971	0.315331771
ENSGALG00010017433	0.720044555	0.124897654
ENSGALG00010017434	0.992227552	0.3190973
ENSGALG00010017435	0.864806998	0.263982178
ENSGALG00010017437	0.90334649	0.367005439
ENSGALG00010017439	0.883683998	0.252620599
ENSGALG00010017440	0.582239405	0.167272153
ENSGALG00010017441	0.90045185	0.211638864
ENSGALG00010017442	0.916279054	0.352651325
ENSGALG00010017445	0.994344875	0.262738331
ENSGALG00010017447	0.950666955	0.328488586
ENSGALG00010017448	0.18954039	0.293505383
ENSGALG00010017449	0.761253705	0.547209605
ENSGALG00010017450	0.453151065	0.15324544
ENSGALG00010017451	0.914770317	0.457652459
ENSGALG00010017452	0.1989348	0.118792223
ENSGALG00010017453	0.990439715	0.316693964
ENSGALG00010017454	0.961472274	0.301058899
ENSGALG00010017455	0.961191895	0.260210864
ENSGALG00010017456	0.315876731	0.251108411
ENSGALG00010017457	0.52247484	0.180840599
ENSGALG00010017458	0.961827015	0.33269702
ENSGALG00010017459	0.879656216	0.384296409
ENSGALG00010017460	0.99681897	0.338127351
ENSGALG00010017461	0.356536265	0.161495931
ENSGALG00010017462	0.803515121	0.355990934
ENSGALG00010017463	0.786789719	0.192197909
ENSGALG00010017464	0.935223318	0.280264298
ENSGALG00010017465	0.743836997	0.174975562
ENSGALG00010017466	0.996820149	0.337415738
ENSGALG00010017467	0.949255686	0.324067422
ENSGALG00010017468	0.448061099	0.084908372
ENSGALG00010017469	0.808287847	0.311571307
ENSGALG00010017470	0.936298827	0.251429588
ENSGALG00010017471	0.21648972	0.139950938

ENSGALG00010017472	0.066716362	0.004473822
ENSGALG00010017473	0.794435232	0.275700039
ENSGALG00010017474	0.895221416	0.310612214
ENSGALG00010017475	0.940607925	0.419452211
ENSGALG00010017476	0.951651781	0.332692331
ENSGALG00010017477	0.962337627	0.328537781
ENSGALG00010017478	0.283529993	0.098309861
ENSGALG00010017479	0.013836069	0.008004162
ENSGALG00010017480	0.59147871	0.045187536
ENSGALG00010017481	0.997267805	0.335491674
ENSGALG00010017482	0.952887474	0.441039847
ENSGALG00010017483	0.729498148	0.216893087
ENSGALG00010017484	0.731098951	0.274961416
ENSGALG00010017485	0.88299168	0.196402164
ENSGALG00010017486	0.347091119	0.431574999
ENSGALG00010017487	0.771319805	0.309133699
ENSGALG00010017488	0.460815871	0.107439019
ENSGALG00010017489	0.972956951	0.293219327
ENSGALG00010017490	0.049201542	0.077915163
ENSGALG00010017491	0.665535365	0.422530731
ENSGALG00010017493	0.66510151	0.097593354
ENSGALG00010017494	0.916442016	0.47351924
ENSGALG00010017495	0.441148347	0.022208272
ENSGALG00010017496	0.049731361	0.245677174
ENSGALG00010017499	0.01258272	0.094273745
ENSGALG00010017500	0.740104494	0.240064487
ENSGALG00010017501	0.997434861	0.336004222
ENSGALG00010017502	0.868084153	0.353058203
ENSGALG00010017503	0.982522707	0.302502195
ENSGALG00010017505	0.146667765	0.056160849
ENSGALG00010017506	0.009565724	0.089300248
ENSGALG00010017509	0.961047441	0.245711693
ENSGALG00010017510	0.875352386	0.134896855
ENSGALG00010017512	0.997024343	0.335984588
ENSGALG00010017513	0.760335081	0.121343636
ENSGALG00010017514	0.561478937	0.100543333
ENSGALG00010017515	0.99548951	0.335505765
ENSGALG00010017516	0.401863969	0.119614576
ENSGALG00010017517	0.730221675	0.47315466
ENSGALG00010017518	0.902853058	0.290204524
ENSGALG00010017519	0.44448616	0.263136237
ENSGALG00010017520	0.929418762	0.400485445
ENSGALG00010017521	0.680664093	0.566297279
ENSGALG00010017522	0.910485594	0.200533736
ENSGALG00010017524	0.420503369	0.255048054
ENSGALG00010017525	0.862057274	0.199743042
ENSGALG00010017526	0.91491399	0.111262572
ENSGALG00010017527	0.954004115	0.323764817
ENSGALG00010017528	0.846545524	0.062803598
ENSGALG00010017529	0.917997252	0.313740566
ENSGALG00010017530	0.943652214	0.222497632
ENSGALG00010017531	0.98410078	0.296028026
ENSGALG00010017532	0.900847571	0.327108413
ENSGALG00010017533	0.886146647	0.334924261
ENSGALG00010017534	0.987463717	0.307716323
ENSGALG00010017535	0.942443396	0.287185742

ENSGALG00010017536	0.7645122	0.376746963
ENSGALG00010017537	0.988391075	0.390278549
ENSGALG00010017538	0.770224489	0.335826592
ENSGALG00010017539	0.975375697	0.389520399
ENSGALG00010017540	0.881945221	0.270305056
ENSGALG00010017541	0.540859642	0.067512567
ENSGALG00010017542	0.313256264	0.147338485
ENSGALG00010017543	0.920010441	0.363235224
ENSGALG00010017544	0.804745589	0.120919707
ENSGALG00010017545	0.718940668	0.402330466
ENSGALG00010017546	0.941002634	0.217623859
ENSGALG00010017547	0.882361879	0.368980041
ENSGALG00010017548	0.169752046	0.126219749
ENSGALG00010017549	0.332434862	0.054204961
ENSGALG00010017550	0.954486898	0.326117061
ENSGALG00010017551	0.864334385	0.303797641
ENSGALG00010017552	0.808361024	0.102887278
ENSGALG00010017553	0.06146493	0.374999601
ENSGALG00010017554	0.369655128	0.212112174
ENSGALG00010017555	0.470668626	0.116119829
ENSGALG00010017556	0.925463288	0.30683162
ENSGALG00010017557	0.486156032	0.502902749
ENSGALG00010017558	0.924375083	0.307603072
ENSGALG00010017559	0.983353172	0.348074999
ENSGALG00010017560	0.277566769	0.107857127
ENSGALG00010017561	0.196452115	0.112355381
ENSGALG00010017562	0.996189462	0.310186957
ENSGALG00010017563	0.947961111	0.372348767
ENSGALG00010017564	0.312151319	0.076731353
ENSGALG00010017565	0.51055728	0.036807648
ENSGALG00010017566	0.316450358	0.016888961
ENSGALG00010017567	0.305552509	0.401971759
ENSGALG00010017568	0.769248451	0.031741061
ENSGALG00010017569	0.977405413	0.343800106
ENSGALG00010017570	0.730833363	0.470467396
ENSGALG00010017571	0.815734775	0.37589541
ENSGALG00010017574	0.843437324	0.278109773
ENSGALG00010017575	0.959866851	0.379675375
ENSGALG00010017576	0.797612198	0.224711986
ENSGALG00010017577	0.610379889	0.117639447
ENSGALG00010017578	0.256604077	0.185877882
ENSGALG00010017579	0.082956089	0.268787459
ENSGALG00010017580	0.673851262	0.151039346
ENSGALG00010017581	0.124864647	0.503726515
ENSGALG00010017582	0.830063771	0.422067235
ENSGALG00010017583	0.987964714	0.310690937
ENSGALG00010017584	0.251188285	0.370593425
ENSGALG00010017585	0.209645075	0.079387343
ENSGALG00010017586	0.818952833	0.079125175
ENSGALG00010017587	0.500326277	0.118719631
ENSGALG00010017588	0.177702564	0.102056874
ENSGALG00010017589	0.923678718	0.302689953
ENSGALG00010017590	0.9320803	0.374537971
ENSGALG00010017591	0.738015915	0.309993219
ENSGALG00010017592	0.955496133	0.289616351
ENSGALG00010017593	0.490888943	0.132027086

ENSGALG00010017594	0.977807369	0.30014963
ENSGALG00010017595	0.584942172	0.21907936
ENSGALG00010017596	0.410153591	0.162644608
ENSGALG00010017597	0.476155535	0.119972006
ENSGALG00010017598	0.97877649	0.275410789
ENSGALG00010017599	0.443609446	0.319176828
ENSGALG00010017600	0.657847451	0.000366782
ENSGALG00010017601	0.07343421	0.303688964
ENSGALG00010017603	0.993555847	0.298156806
ENSGALG00010017604	0.119461082	0.256845134
ENSGALG00010017605	0.964743161	0.265238776
ENSGALG00010017606	0.586672304	0.355632903
ENSGALG00010017608	0.686447291	0.103090082
ENSGALG00010017609	0.135002105	0.165821506
ENSGALG00010017610	0.951268182	0.364002319
ENSGALG00010017612	0.372010435	0.244417323
ENSGALG00010017613	0.551053246	0.220923917
ENSGALG00010017615	0.926032072	0.364364068
ENSGALG00010017616	0.894910847	0.162787842
ENSGALG00010017618	0.069283666	0.12825803
ENSGALG00010017619	0.364819035	0.215582163
ENSGALG00010017620	0.16154644	0.317934481
ENSGALG00010017621	0.9111046	0.208571619
ENSGALG00010017622	0.864371979	0.255284398
ENSGALG00010017625	0.98010268	0.356634968
ENSGALG00010017626	0.926151468	0.265614393
ENSGALG00010017627	0.505595959	0.101263738
ENSGALG00010017628	0.16899017	0.508239365
ENSGALG00010017629	0.363256288	0.165264164
ENSGALG00010017631	0.654772561	0.108746545
ENSGALG00010017632	0.991730375	0.34401906
ENSGALG00010017633	0.129871437	0.301455538
ENSGALG00010017634	0.121231418	0.176370819
ENSGALG00010017635	0.396046331	0.44788194
ENSGALG00010017636	0.892237293	0.35675527
ENSGALG00010017638	0.93801436	0.227160718
ENSGALG00010017639	0.351818796	0.001616153
ENSGALG00010017640	0.989827525	0.312343638
ENSGALG00010017641	0.993937239	0.323906452
ENSGALG00010017642	0.731545932	0.256043163
ENSGALG00010017643	0.99355848	0.346027778
ENSGALG00010017644	0.573125576	0.036599395
ENSGALG00010017645	0.987892017	0.254471689
ENSGALG00010017646	0.947995982	0.315240826
ENSGALG00010017647	0.183229953	0.146691036
ENSGALG00010017648	0.715737409	0.210427132
ENSGALG00010017649	0.973981932	0.315241818
ENSGALG00010017650	0.594676391	0.150304719
ENSGALG00010017651	0.214018368	0.023629893
ENSGALG00010017652	0.967099594	0.275097119
ENSGALG00010017653	0.993321841	0.296013394
ENSGALG00010017654	0.939893315	0.371615373
ENSGALG00010017655	0.621885786	0.215000514
ENSGALG00010017656	0.996382906	0.310883926
ENSGALG00010017657	0.236666115	0.042143743
ENSGALG00010017658	0.970374548	0.20776701

ENSGALG00010017659	0.768158649	0.441019428
ENSGALG00010017660	0.983783987	0.357231177
ENSGALG00010017661	0.975055306	0.33841853
ENSGALG00010017662	0.82085348	0.223313622
ENSGALG00010017663	0.733666795	0.207251035
ENSGALG00010017664	0.738108516	0.014613334
ENSGALG00010017665	0.555440424	0.069655178
ENSGALG00010017666	0.285700292	0.244698352
ENSGALG00010017667	0.473838036	0.229493872
ENSGALG00010017668	0.892096036	0.124938431
ENSGALG00010017669	0.105574677	0.008363559
ENSGALG00010017670	0.868467338	0.529324481
ENSGALG00010017671	0.936171351	0.396830061
ENSGALG00010017672	0.876628112	0.292852835
ENSGALG00010017673	0.874604752	0.228782299
ENSGALG00010017675	0.667050995	0.011401403
ENSGALG00010017676	0.70641826	0.25361323
ENSGALG00010017677	0.30741302	0.088980984
ENSGALG00010017678	0.672142782	0.044225528
ENSGALG00010017679	0.872900737	0.185867582
ENSGALG00010017681	0.963497125	0.327602155
ENSGALG00010017682	0.978174908	0.344190085
ENSGALG00010017683	0.882284099	0.383615075
ENSGALG00010017684	0.311854309	0.205790051
ENSGALG00010017685	0.980969535	0.332166566
ENSGALG00010017687	0.079100666	0.203549952
ENSGALG00010017688	0.770018161	0.496099514
ENSGALG00010017689	0.949170662	0.320214842
ENSGALG00010017690	0.780247114	0.343559501
ENSGALG00010017691	0.770959069	0.103600326
ENSGALG00010017692	0.992836161	0.355580693
ENSGALG00010017693	0.404662227	0.076424184
ENSGALG00010017694	0.77030865	0.361962652
ENSGALG00010017695	0.975143144	0.336530737
ENSGALG00010017696	0.1483747	0.34261117
ENSGALG00010017697	0.672346161	0.305296586
ENSGALG00010017698	0.075925671	0.020377447
ENSGALG00010017699	0.801684879	0.235016801
ENSGALG00010017700	0.754371995	0.272546827
ENSGALG00010017701	0.726378168	0.086750139
ENSGALG00010017703	0.623629889	0.069547698
ENSGALG00010017704	0.973498166	0.337767021
ENSGALG00010017705	0.970232472	0.286266491
ENSGALG00010017706	0.940531159	0.33283339
ENSGALG00010017707	0.653928752	0.188755228
ENSGALG00010017708	0.976732901	0.372194776
ENSGALG00010017710	0.928845806	0.282627162
ENSGALG00010017711	0.859510024	0.201921907
ENSGALG00010017712	0.486672478	0.311686208
ENSGALG00010017713	0.988284422	0.33408437
ENSGALG00010017714	0.799371941	0.070979863
ENSGALG00010017715	0.984031383	0.32835311
ENSGALG00010017716	0.189025428	0.100613405
ENSGALG00010017717	0.94014372	0.324762733
ENSGALG00010017718	0.831116507	0.201022045
ENSGALG00010017720	0.208539525	0.33080183

ENSGALG00010017721	0.589075306	0.337812509
ENSGALG00010017723	0.248030557	0.190725343
ENSGALG00010017724	0.995526131	0.327634681
ENSGALG00010017725	0.963901833	0.251058182
ENSGALG00010017726	0.19052018	0.544002549
ENSGALG00010017727	0.737162158	0.3921208
ENSGALG00010017729	0.985174787	0.347774593
ENSGALG00010017730	0.946665662	0.283248778
ENSGALG00010017732	0.42504936	0.00202996
ENSGALG00010017733	0.629216187	0.073387933
ENSGALG00010017734	0.667314387	0.353422577
ENSGALG00010017735	0.789635514	0.060197048
ENSGALG00010017737	0.734644493	0.123747816
ENSGALG00010017738	0.961419408	0.338000147
ENSGALG00010017739	0.845230291	0.285982447
ENSGALG00010017740	0.491514942	0.166389109
ENSGALG00010017741	0.910754557	0.27642688
ENSGALG00010017742	0.880673863	0.469224717
ENSGALG00010017744	0.734024949	0.380071325
ENSGALG00010017745	0.980177192	0.317565303
ENSGALG00010017746	0.959451802	0.316302844
ENSGALG00010017747	0.970661996	0.325802577
ENSGALG00010017748	0.134496827	0.181222575
ENSGALG00010017749	0.141003976	0.019420038
ENSGALG00010017750	0.264239718	0.073427965
ENSGALG00010017751	0.90830789	0.268153719
ENSGALG00010017752	0.944610834	0.352173878
ENSGALG00010017753	0.100091479	0.026080987
ENSGALG00010017754	0.94115821	0.257554318
ENSGALG00010017755	0.231370633	0.493429574
ENSGALG00010017756	0.607172897	0.324430856
ENSGALG00010017757	0.994131567	0.332777038
ENSGALG00010017758	0.026966462	0.158284821
ENSGALG00010017759	0.86727575	0.213216753
ENSGALG00010017760	0.978706612	0.331913357
ENSGALG00010017761	0.409040733	0.029323072
ENSGALG00010017762	0.743428999	0.275087934
ENSGALG00010017763	0.509304147	0.440981467
ENSGALG00010017764	0.879410865	0.525590733
ENSGALG00010017765	0.715834777	0.206469747
ENSGALG00010017766	0.983865088	0.279857101
ENSGALG00010017767	0.694711639	0.106082651
ENSGALG00010017768	0.933301348	0.439117408
ENSGALG00010017769	0.653501435	0.335721438
ENSGALG00010017770	0.373060534	0.068156341
ENSGALG00010017771	0.938809313	0.317643163
ENSGALG00010017772	0.981963821	0.368105402
ENSGALG00010017773	0.604597607	0.038385673
ENSGALG00010017774	0.877287953	0.212081489
ENSGALG00010017775	0.132679179	0.228599164
ENSGALG00010017776	0.81552272	0.376019465
ENSGALG00010017777	0.428366052	0.503171251
ENSGALG00010017778	0.983551705	0.306871736
ENSGALG00010017779	0.943364749	0.286469065
ENSGALG00010017780	0.591540172	0.263878366
ENSGALG00010017781	0.286891908	0.113554265

ENSGALG00010017782	0.975372994	0.331600997
ENSGALG00010017783	0.781312459	0.203920658
ENSGALG00010017784	0.775673867	0.08386867
ENSGALG00010017785	0.525456912	0.378156691
ENSGALG00010017786	0.961522855	0.3715569
ENSGALG00010017787	0.795133985	0.062716539
ENSGALG00010017788	0.211022858	0.278044165
ENSGALG00010017789	0.967987222	0.32032632
ENSGALG00010017790	0.74810742	0.3360599
ENSGALG00010017791	0.971158416	0.369599161
ENSGALG00010017792	0.930582103	0.34169405
ENSGALG00010017793	0.780399978	0.309977525
ENSGALG00010017794	0.394927857	0.119113854
ENSGALG00010017795	0.976644091	0.319370549
ENSGALG00010017796	0.035668542	0.049867246
ENSGALG00010017797	0.943235521	0.371541481
ENSGALG00010017798	0.90214524	0.28021892
ENSGALG00010017799	0.858859777	0.168717967
ENSGALG00010017800	0.674225094	0.255502056
ENSGALG00010017801	0.656248875	0.184801821
ENSGALG00010017802	0.547547761	0.182574154
ENSGALG00010017803	0.051359204	0.091655989
ENSGALG00010017804	0.833364259	0.199679938
ENSGALG00010017805	0.973407529	0.253664268
ENSGALG00010017806	0.614218282	0.285501484
ENSGALG00010017807	0.990523899	0.32062728
ENSGALG00010017808	0.965277612	0.330167065
ENSGALG00010017809	0.705651906	0.253037802
ENSGALG00010017810	0.949188944	0.300928912
ENSGALG00010017811	0.960210235	0.341618737
ENSGALG00010017812	0.592280479	0.161656445
ENSGALG00010017813	0.785461042	0.384477121
ENSGALG00010017814	0.949353222	0.405179177
ENSGALG00010017815	0.682175885	0.350774704
ENSGALG00010017816	0.136717886	0.060412273
ENSGALG00010017817	0.990889918	0.328149777
ENSGALG00010017818	0.672913425	0.299101007
ENSGALG00010017819	0.564443915	0.089936092
ENSGALG00010017820	0.965876515	0.340510367
ENSGALG00010017821	0.234217401	0.153493537
ENSGALG00010017822	0.02254112	0.295702002
ENSGALG00010017823	0.886630636	0.350808351
ENSGALG00010017824	0.770731764	0.560557227
ENSGALG00010017825	0.670107282	0.404990075
ENSGALG00010017826	0.998035976	0.353543851
ENSGALG00010017827	0.388459236	0.25023441
ENSGALG00010017828	0.053619445	0.019509842
ENSGALG00010017830	0.248600584	0.018740054
ENSGALG00010017831	0.959620773	0.354610081
ENSGALG00010017832	0.322502828	0.568703667
ENSGALG00010017833	0.177462875	0.066943453
ENSGALG00010017834	0.500023784	0.496886448
ENSGALG00010017835	0.260616931	0.093722119
ENSGALG00010017836	0.193841428	0.299238428
ENSGALG00010017837	0.28880012	0.418789177
ENSGALG00010017838	0.960526294	0.450910384

ENSGALG00010017839	0.541377515	0.003194044
ENSGALG00010017840	0.04515196	0.174262167
ENSGALG00010017841	0.863413924	0.343971537
ENSGALG00010017842	0.45915399	0.156003325
ENSGALG00010017843	0.932332695	0.403419582
ENSGALG00010017844	0.010676477	0.136800314
ENSGALG00010017846	0.018411501	0.102218592
ENSGALG00010017847	0.508027854	0.357448149
ENSGALG00010017848	0.895721992	0.245414134
ENSGALG00010017849	0.663072481	0.111988127
ENSGALG00010017850	0.734975559	0.403772651
ENSGALG00010017851	0.250541966	0.050696871
ENSGALG00010017852	0.007193065	0.068657475
ENSGALG00010017853	0.55985554	0.358394578
ENSGALG00010017854	0.565474246	0.223228225
ENSGALG00010017855	0.231410825	0.361724914
ENSGALG00010017856	0.787754325	0.143636812
ENSGALG00010017857	0.803453562	0.251332955
ENSGALG00010017858	0.964876794	0.387955554
ENSGALG00010017859	0.941632292	0.230361993
ENSGALG00010017860	0.192755438	0.295097319
ENSGALG00010017861	0.589691259	0.053022113
ENSGALG00010017862	0.663188297	0.043013948
ENSGALG00010017863	0.572708465	0.188772026
ENSGALG00010017864	0.752409906	0.268174788
ENSGALG00010017865	0.842961599	0.228477773
ENSGALG00010017866	0.460563159	0.147833835
ENSGALG00010017867	0.751399383	0.126544029
ENSGALG00010017868	0.978354966	0.283449831
ENSGALG00010017869	0.553339965	0.193914998
ENSGALG00010017870	0.851825683	0.059441769
ENSGALG00010017871	0.714171842	0.138525092
ENSGALG00010017872	0.784013297	0.30918584
ENSGALG00010017873	0.962832207	0.394264009
ENSGALG00010017874	0.889150124	0.204728432
ENSGALG00010017875	0.011061717	0.17509238
ENSGALG00010017876	0.889964588	0.328599285
ENSGALG00010017877	0.570588423	0.222050898
ENSGALG00010017878	0.908105351	0.2738189
ENSGALG00010017879	0.207215102	0.157405026
ENSGALG00010017880	0.909598981	0.31246621
ENSGALG00010017881	0.907337194	0.312455187
ENSGALG00010017882	0.905925696	0.372467326
ENSGALG00010017883	0.572186061	0.171354425
ENSGALG00010017884	0.690321685	0.269795861
ENSGALG00010017885	0.678529666	0.077889295
ENSGALG00010017886	0.891939137	0.260952521
ENSGALG00010017887	0.927520956	0.368327407
ENSGALG00010017888	0.658564382	0.425846059
ENSGALG00010017889	0.189816433	0.25584371
ENSGALG00010017890	0.67816757	0.273763697
ENSGALG00010017891	0.993678842	0.316676665
ENSGALG00010017894	0.802221872	0.260546254
ENSGALG00010017895	0.783400801	0.16204469
ENSGALG00010017898	0.952912981	0.256898683
ENSGALG00010017901	0.131629194	0.118217065

ENSGALG00010017902	0.405924564	0.006458455
ENSGALG00010017903	0.430265427	0.126239061
ENSGALG00010017904	0.369389236	0.349340578
ENSGALG00010017905	0.928020344	0.307971764
ENSGALG00010017906	0.557172774	0.010679925
ENSGALG00010017908	0.927681218	0.245247546
ENSGALG00010017909	0.734873293	0.254438086
ENSGALG00010017911	0.127903215	0.166108816
ENSGALG00010017913	0.838137049	0.23303016
ENSGALG00010017915	0.759464361	0.400873747
ENSGALG00010017917	0.787392311	0.40979765
ENSGALG00010017918	0.836491628	0.116927936
ENSGALG00010017920	0.940767713	0.316171432
ENSGALG00010017921	0.172797959	0.256003108
ENSGALG00010017922	0.979617243	0.279895483
ENSGALG00010017924	0.270451009	0.090541667
ENSGALG00010017927	0.054964012	0.205318313
ENSGALG00010017930	0.797208695	0.298582956
ENSGALG00010017931	0.052838346	0.100637787
ENSGALG00010017932	0.3619239	0.131870846
ENSGALG00010017933	0.979618755	0.327121818
ENSGALG00010017936	0.488428693	0.127907235
ENSGALG00010017937	0.908787673	0.401609348
ENSGALG00010017938	0.555100443	0.072140719
ENSGALG00010017939	0.152614871	0.489874215
ENSGALG00010017940	0.975092723	0.237275857
ENSGALG00010017941	0.01758475	0.114985429
ENSGALG00010017942	0.04000971	0.185397503
ENSGALG00010017943	0.838117914	0.18295197
ENSGALG00010017944	0.194426889	0.183420005
ENSGALG00010017945	0.067800255	0.408748399
ENSGALG00010017946	0.716379133	0.453014838
ENSGALG00010017948	0.62235469	0.292489823
ENSGALG00010017949	0.983266007	0.30396545
ENSGALG00010017950	0.709546166	0.335802311
ENSGALG00010017951	0.271052982	0.134649085
ENSGALG00010017952	0.670270691	0.310508581
ENSGALG00010017954	0.848888375	0.306182243
ENSGALG00010017955	0.530490574	0.017921516
ENSGALG00010017956	0.54776638	0.027258934
ENSGALG00010017957	0.973797325	0.332361911
ENSGALG00010017958	0.207828297	0.108328404
ENSGALG00010017959	0.928169313	0.224797962
ENSGALG00010017962	0.89533856	0.149559003
ENSGALG00010017963	0.644046366	0.219624887
ENSGALG00010017964	0.552711739	0.006521871
ENSGALG00010017967	0.94972925	0.396086587
ENSGALG00010017968	0.308758304	0.100827364
ENSGALG00010017970	0.003044037	0.055935543
ENSGALG00010017972	0.844489632	0.119761656
ENSGALG00010017973	0.491137744	0.013775933
ENSGALG00010017974	0.887484114	0.437121222
ENSGALG00010017975	0.277566769	0.107857127
ENSGALG00010017976	0.349606711	0.249319179
ENSGALG00010017977	0.165954831	0.243506135
ENSGALG00010017978	0.054846918	0.196234361

ENSGALG00010017979	0.758592122	0.026540677
ENSGALG00010017980	0.680588875	0.281707244
ENSGALG00010017981	0.803854741	0.23175255
ENSGALG00010017982	0.122838001	0.173682415
ENSGALG00010017983	0.564868986	0.393248003
ENSGALG00010017985	0.794459582	0.416281347
ENSGALG00010017986	0.31143421	0.181196866
ENSGALG00010017987	0.871256383	0.47957794
ENSGALG00010017990	0.433273665	0.432412529
ENSGALG00010017991	0.942702706	0.3656959
ENSGALG00010017992	0.539961739	0.103166807
ENSGALG00010017993	0.889234497	0.428845755
ENSGALG00010017994	0.500566545	0.203434961
ENSGALG00010017995	0.676881798	0.23471744
ENSGALG00010017996	0.867808323	0.239977179
ENSGALG00010017997	0.041837841	0.076085566
ENSGALG00010017998	0.367818795	0.105765563
ENSGALG00010017999	0.965561326	0.261428074
ENSGALG00010018000	0.96518015	0.306417171
ENSGALG00010018001	0.047567746	0.06079998
ENSGALG00010018002	0.906924705	0.248457894
ENSGALG00010018003	0.702196713	0.004294275
ENSGALG00010018004	0.055893501	0.041576028
ENSGALG00010018005	0.049999924	0.07065685
ENSGALG00010018006	0.896773104	0.280418177
ENSGALG00010018007	0.715815088	0.233986813
ENSGALG00010018008	0.995053269	0.339438169
ENSGALG00010018009	0.950941439	0.335112368
ENSGALG00010018010	0.971852208	0.388900615
ENSGALG00010018011	0.981843532	0.27425119
ENSGALG00010018012	0.983557332	0.303605035
ENSGALG00010018013	0.853827164	0.18424478
ENSGALG00010018014	0.680133411	0.323884306
ENSGALG00010018015	0.189826181	0.004568278
ENSGALG00010018016	0.910814891	0.388227881
ENSGALG00010018017	0.576346197	0.304512843
ENSGALG00010018018	0.903236495	0.141533873
ENSGALG00010018019	0.861761451	0.170560248
ENSGALG00010018020	0.301625718	0.111908818
ENSGALG00010018021	0.91785779	0.376411869
ENSGALG00010018022	0.968003434	0.317024338
ENSGALG00010018023	0.936036252	0.312922821
ENSGALG00010018024	0.126855773	0.139884367
ENSGALG00010018025	0.978280159	0.228431217
ENSGALG00010018026	0.479701227	0.391334908
ENSGALG00010018027	0.826967329	0.202897954
ENSGALG00010018028	0.461383739	0.199272115
ENSGALG00010018029	0.000888923	0.059466089
ENSGALG00010018030	0.868101025	0.319039733
ENSGALG00010018031	0.557006097	0.154178119
ENSGALG00010018032	0.891914502	0.339515046
ENSGALG00010018033	0.951016777	0.278848127
ENSGALG00010018034	0.597106113	0.319046609
ENSGALG00010018035	0.914623439	0.263954061
ENSGALG00010018036	0.866113602	0.340118639
ENSGALG00010018037	0.825011149	0.234491925

ENSGALG00010018038	0.367717776	0.130093535
ENSGALG00010018039	0.76605016	0.51463475
ENSGALG00010018042	0.797799184	0.189115692
ENSGALG00010018043	0.909530183	0.391285324
ENSGALG00010018044	0.714139541	0.350998402
ENSGALG00010018045	0.912369376	0.343094789
ENSGALG00010018046	0.886784978	0.208995525
ENSGALG00010018048	0.426505629	0.235093301
ENSGALG00010018049	0.958655457	0.347578062
ENSGALG00010018051	0.971204207	0.299609335
ENSGALG00010018052	0.665037995	0.402793431
ENSGALG00010018053	0.941455091	0.337795607
ENSGALG00010018054	0.56213197	0.447135922
ENSGALG00010018055	0.425484694	0.099613236
ENSGALG00010018057	0.842049611	0.172096598
ENSGALG00010018058	0.791917641	0.17411745
ENSGALG00010018059	0.316699311	0.214961316
ENSGALG00010018060	0.500875013	0.18230077
ENSGALG00010018061	0.826778382	0.325563975
ENSGALG00010018062	0.967562358	0.363988831
ENSGALG00010018063	0.921613585	0.28169475
ENSGALG00010018064	0.8898593	0.323011824
ENSGALG00010018065	0.36254603	0.12261176
ENSGALG00010018066	0.601925518	0.054566628
ENSGALG00010018067	0.863683069	0.120874259
ENSGALG00010018068	0.152896559	0.248771144
ENSGALG00010018069	0.754308668	0.071150062
ENSGALG00010018070	0.422683229	0.240384059
ENSGALG00010018071	0.535267994	0.367698726
ENSGALG00010018072	0.209270226	0.319472177
ENSGALG00010018073	0.764005397	0.324506798
ENSGALG00010018075	0.944762985	0.306764033
ENSGALG00010018076	0.996640814	0.336776725
ENSGALG00010018078	0.898686936	0.454886174
ENSGALG00010018080	0.910213072	0.252958832
ENSGALG00010018081	0.773814269	0.143874908
ENSGALG00010018082	0.822130492	0.16718836
ENSGALG00010018083	0.273159857	0.750342917
ENSGALG00010018085	0.966032936	0.26884337
ENSGALG00010018086	0.393756035	0.130583636
ENSGALG00010018087	0.847881914	0.435314291
ENSGALG00010018089	0.59869031	0.179321018
ENSGALG00010018090	0.285272007	0.109169955
ENSGALG00010018092	0.804918359	0.40534144
ENSGALG00010018095	0.782688222	0.142087971
ENSGALG00010018098	0.027666716	0.187870113
ENSGALG00010018099	0.901038158	0.332053503
ENSGALG00010018101	0.284227058	0.103920777
ENSGALG00010018102	0.939677628	0.28663351
ENSGALG00010018103	0.120313157	0.054763501
ENSGALG00010018105	0.265977428	0.217865653
ENSGALG00010018108	0.971259959	0.359426983
ENSGALG00010018109	0.637417611	0.297829976
ENSGALG00010018110	0.54882741	0.203422153
ENSGALG00010018112	0.622792236	0.314178094
ENSGALG00010018114	0.933528517	0.44791585

ENSGALG00010018115	0.965678642	0.2649282
ENSGALG00010018118	0.13021111	0.300852233
ENSGALG00010018119	0.557422601	0.461361938
ENSGALG00010018120	0.318427197	0.195947252
ENSGALG00010018121	0.741657068	0.193495781
ENSGALG00010018122	0.254040886	0.265753446
ENSGALG00010018123	0.985752236	0.323779362
ENSGALG00010018124	0.333180514	0.13683277
ENSGALG00010018126	0.159731047	0.165430564
ENSGALG00010018127	0.080384603	0.080231585
ENSGALG00010018128	0.872096024	0.472434795
ENSGALG00010018129	0.378986826	0.110289096
ENSGALG00010018131	0.899376285	0.320784544
ENSGALG00010018132	0.400215036	0.132215663
ENSGALG00010018133	0.469113109	0.11455071
ENSGALG00010018136	0.368635925	0.132759256
ENSGALG00010018137	0.977461642	0.335597902
ENSGALG00010018138	0.032840259	0.449987318
ENSGALG00010018139	0.98141951	0.337260117
ENSGALG00010018140	0.448811626	0.193613413
ENSGALG00010018141	0.955446526	0.340408681
ENSGALG00010018142	0.958417015	0.2760092
ENSGALG00010018143	0.8583868	0.240095091
ENSGALG00010018145	0.236959829	0.160727502
ENSGALG00010018147	0.165167041	0.158354236
ENSGALG00010018148	0.156888767	0.061708278
ENSGALG00010018149	0.983060717	0.330897118
ENSGALG00010018150	0.843298153	0.054272914
ENSGALG00010018152	0.462916886	0.275660467
ENSGALG00010018153	0.433535616	0.10403681
ENSGALG00010018154	0.983208029	0.308642556
ENSGALG00010018155	0.465740677	0.770245842
ENSGALG00010018156	0.660016214	0.044794608
ENSGALG00010018157	0.78391554	0.315879361
ENSGALG00010018158	0.175739858	0.298352128
ENSGALG00010018160	0.845474192	0.439083788
ENSGALG00010018162	0.970945712	0.406606663
ENSGALG00010018163	0.728071235	0.165345872
ENSGALG00010018165	0.923482348	0.256736976
ENSGALG00010018166	0.991494632	0.343572151
ENSGALG00010018168	0.351542667	0.070035234
ENSGALG00010018169	0.578677792	0.178011943
ENSGALG00010018170	0.962906973	0.200663615
ENSGALG00010018171	0.085497629	0.146819802
ENSGALG00010018172	0.573647885	0.320234974
ENSGALG00010018173	0.609584644	0.299505944
ENSGALG00010018174	0.308050833	0.289148505
ENSGALG00010018175	0.2722955	0.398974216
ENSGALG00010018176	0.585131647	0.678573333
ENSGALG00010018177	0.891497909	0.085170109
ENSGALG00010018179	0.747413471	0.26303761
ENSGALG00010018181	0.267456708	0.077408342
ENSGALG00010018183	0.853266223	0.124055687
ENSGALG00010018185	0.952215056	0.20844782
ENSGALG00010018186	0.353657346	0.311005176
ENSGALG00010018187	0.285272007	0.109169955

ENSGALG00010018188	0.9116409	0.332550607
ENSGALG00010018189	0.435052461	0.525352419
ENSGALG00010018190	0.706192791	0.399562171
ENSGALG00010018192	0.793095863	0.015778074
ENSGALG00010018193	0.948915742	0.347495662
ENSGALG00010018194	0.576378169	0.285692827
ENSGALG00010018197	0.149727174	0.352546499
ENSGALG00010018198	0.898773448	0.255645635
ENSGALG00010018199	0.463446747	0.317166694
ENSGALG00010018200	0.607422279	0.494353532
ENSGALG00010018202	0.420966174	0.396635401
ENSGALG00010018203	0.783041095	0.300094701
ENSGALG00010018205	0.825007148	0.368713724
ENSGALG00010018206	0.590245529	0.04409343
ENSGALG00010018208	0.401138341	0.518829888
ENSGALG00010018209	0.034906606	0.287651478
ENSGALG00010018212	0.618937223	0.039431781
ENSGALG00010018213	0.152476645	0.19105403
ENSGALG00010018215	0.96582613	0.295467289
ENSGALG00010018216	0.586724787	0.61968082
ENSGALG00010018217	0.154332453	0.156853136
ENSGALG00010018219	0.391902249	0.004503542
ENSGALG00010018221	0.024966345	0.138378133
ENSGALG00010018224	0.45704954	0.358567505
ENSGALG00010018225	0.059539862	0.180376229
ENSGALG00010018226	0.173201081	0.002098709
ENSGALG00010018227	0.686592638	0.478898406
ENSGALG00010018229	0.929505117	0.336220861
ENSGALG00010018231	0.262108355	0.189503318
ENSGALG00010018232	0.388181886	0.168275567
ENSGALG00010018233	0.962336395	0.266735184
ENSGALG00010018236	0.869888561	0.127993862
ENSGALG00010018237	0.033499114	0.176182674
ENSGALG00010018240	0.813832549	0.07486169
ENSGALG00010018241	0.044910983	0.476980333
ENSGALG00010018244	0.809473505	0.199682991
ENSGALG00010018247	0.619296958	0.134826483
ENSGALG00010018248	0.310184688	0.17631624
ENSGALG00010018250	0.282976025	0.145658897
ENSGALG00010018251	0.864417713	0.306814343
ENSGALG00010018252	0.59008763	0.190354068
ENSGALG00010018254	0.609424461	0.258284295
ENSGALG00010018255	0.824554521	0.227901838
ENSGALG00010018258	0.1178595	0.237221595
ENSGALG00010018259	0.547764701	0.165515542
ENSGALG00010018260	0.84924653	0.287138949
ENSGALG00010018261	0.762315461	0.332134517
ENSGALG00010018262	0.528128989	0.195214608
ENSGALG00010018263	0.640091324	0.524642395
ENSGALG00010018264	0.823218128	0.32011759
ENSGALG00010018265	0.562762353	0.187451176
ENSGALG00010018267	0.908014351	0.212716389
ENSGALG00010018268	0.672098666	0.205481243
ENSGALG00010018270	0.602383798	0.09281779
ENSGALG00010018274	0.839008211	0.12756403
ENSGALG00010018275	0.921797909	0.247732813

ENSGALG00010018276	0.817281129	0.179613976
ENSGALG00010018277	0.576003696	0.238182964
ENSGALG00010018278	0.501834662	0.622256436
ENSGALG00010018281	0.480409836	0.168729102
ENSGALG00010018282	0.436826324	0.193864427
ENSGALG00010018283	0.432298881	0.223631983
ENSGALG00010018284	0.983436508	0.320380473
ENSGALG00010018285	0.205300762	0.057100233
ENSGALG00010018287	0.881541768	0.367611292
ENSGALG00010018288	0.830489702	0.304164334
ENSGALG00010018289	0.81628082	0.25705247
ENSGALG00010018290	0.84754241	0.355573821
ENSGALG00010018291	0.817911521	0.16956202
ENSGALG00010018292	0.111439996	0.148089802
ENSGALG00010018294	0.97758636	0.285124658
ENSGALG00010018295	0.203991683	0.058221925
ENSGALG00010018296	0.226449844	0.298525499
ENSGALG00010018298	0.931762003	0.323065633
ENSGALG00010018299	0.92376294	0.276092399
ENSGALG00010018300	0.270451009	0.090541667
ENSGALG00010018301	0.496101277	0.045103494
ENSGALG00010018302	0.037592416	0.268579382
ENSGALG00010018304	0.177011403	0.149495548
ENSGALG00010018305	0.076101516	0.165144032
ENSGALG00010018306	0.997207517	0.337643545
ENSGALG00010018307	0.163720607	0.08610252
ENSGALG00010018308	0.036967671	0.099576166
ENSGALG00010018309	0.879462496	0.391648335
ENSGALG00010018310	0.94898744	0.393812569
ENSGALG00010018311	0.220351476	0.25023501
ENSGALG00010018312	0.892606361	0.431115585
ENSGALG00010018313	0.694082327	0.359131411
ENSGALG00010018314	0.548199788	0.243903107
ENSGALG00010018315	0.663567949	0.288718694
ENSGALG00010018317	0.86288867	0.174847429
ENSGALG00010018319	0.950715772	0.29153987
ENSGALG00010018321	0.147443429	0.191673149
ENSGALG00010018322	0.997178838	0.333586913
ENSGALG00010018323	0.827643811	0.333776522
ENSGALG00010018324	0.657003213	0.223749926
ENSGALG00010018325	0.985946109	0.237286234
ENSGALG00010018326	0.707341106	0.286910435
ENSGALG00010018327	0.565636165	0.133725131
ENSGALG00010018328	0.790654137	0.299822009
ENSGALG00010018329	0.896816293	0.144582944
ENSGALG00010018330	0.240323044	0.14483563
ENSGALG00010018331	0.936190426	0.335673563
ENSGALG00010018332	0.905658791	0.317448214
ENSGALG00010018333	0.943350006	0.288275918
ENSGALG00010018334	0.550037248	0.252762033
ENSGALG00010018335	0.285272007	0.109169955
ENSGALG00010018336	0.949673181	0.387442161
ENSGALG00010018337	0.431166402	0.296849804
ENSGALG00010018338	0.673951038	0.225992024
ENSGALG00010018339	0.936772822	0.224014801
ENSGALG00010018340	0.998162697	0.338390868

ENSGALG00010018342	0.957162826	0.373118749
ENSGALG00010018344	0.868577795	0.288954074
ENSGALG00010018345	0.86383788	0.302653775
ENSGALG00010018346	0.751442841	0.11171154
ENSGALG00010018347	0.509102951	0.346877213
ENSGALG00010018348	0.248829363	0.260345702
ENSGALG00010018349	0.856186704	0.444122562
ENSGALG00010018350	0.64114195	0.292099903
ENSGALG00010018351	0.564518001	0.169223913
ENSGALG00010018352	0.94508962	0.244068558
ENSGALG00010018353	0.917764524	0.410942924
ENSGALG00010018354	0.860642904	0.286197711
ENSGALG00010018355	0.947526041	0.32671705
ENSGALG00010018356	0.399244056	0.168787816
ENSGALG00010018357	0.974260892	0.374058907
ENSGALG00010018358	0.795869934	0.228743742
ENSGALG00010018359	0.048522101	0.113076891
ENSGALG00010018360	0.657242799	0.371004524
ENSGALG00010018361	0.841534077	0.3979628
ENSGALG00010018363	0.940267433	0.430018629
ENSGALG00010018364	0.422425593	0.109687795
ENSGALG00010018365	0.276160635	0.065765646
ENSGALG00010018366	0.345783546	0.016904492
ENSGALG00010018367	0.873565041	0.523649649
ENSGALG00010018368	0.115358744	0.101312651
ENSGALG00010018369	0.932202656	0.31757879
ENSGALG00010018370	0.058329623	0.014006682
ENSGALG00010018371	0.653768353	0.529077528
ENSGALG00010018372	0.977837793	0.247362494
ENSGALG00010018373	0.413681442	0.210398046
ENSGALG00010018374	0.959669929	0.243540521
ENSGALG00010018375	0.285272007	0.109169955
ENSGALG00010018376	0.772264328	0.188351026
ENSGALG00010018377	0.560961564	0.19671491
ENSGALG00010018378	0.989165638	0.332718789
ENSGALG00010018379	0.985530937	0.292498059
ENSGALG00010018380	0.853629428	0.294432934
ENSGALG00010018381	0.971457349	0.309897673
ENSGALG00010018383	0.811136184	0.26867791
ENSGALG00010018384	0.893241011	0.162112924
ENSGALG00010018385	0.964192501	0.233674951
ENSGALG00010018387	0.988020897	0.369288776
ENSGALG00010018388	0.959214809	0.30480063
ENSGALG00010018390	0.719034557	0.268083045
ENSGALG00010018391	0.890756035	0.422851667
ENSGALG00010018392	0.363332073	0.116301156
ENSGALG00010018393	0.551666263	0.07165635
ENSGALG00010018394	0.295736713	0.165463329
ENSGALG00010018395	0.73741703	0.230692692
ENSGALG00010018396	0.695793464	0.08967859
ENSGALG00010018397	0.236036274	0.006577611
ENSGALG00010018398	0.689826658	0.328704745
ENSGALG00010018399	0.955783873	0.208770799
ENSGALG00010018400	0.908588171	0.457443138
ENSGALG00010018401	0.733221193	0.118139071
ENSGALG00010018402	0.329661263	0.151807418

ENSGALG00010018403	0.636265573	0.282527515
ENSGALG00010018404	0.974178703	0.304980592
ENSGALG00010018405	0.850335864	0.468930368
ENSGALG00010018406	0.918494472	0.273294666
ENSGALG00010018407	0.375927362	0.072713138
ENSGALG00010018409	0.828457425	0.239115988
ENSGALG00010018410	0.595593199	0.49166769
ENSGALG00010018413	0.10444442	0.029044879
ENSGALG00010018414	0.954636055	0.258980576
ENSGALG00010018415	0.899136375	0.288761391
ENSGALG00010018416	0.577218298	0.142984689
ENSGALG00010018417	0.538787396	0.073820137
ENSGALG00010018418	0.467424892	0.192766651
ENSGALG00010018419	0.977652103	0.236591313
ENSGALG00010018420	0.982973539	0.313453298
ENSGALG00010018421	0.882468973	0.301080321
ENSGALG00010018422	0.921933533	0.321252753
ENSGALG00010018423	0.993620816	0.338536433
ENSGALG00010018424	0.365255074	0.237443633
ENSGALG00010018425	0.697581123	0.092244013
ENSGALG00010018426	0.709168115	0.199258531
ENSGALG00010018427	0.977610155	0.323943539
ENSGALG00010018428	0.354738917	0.143589189
ENSGALG00010018429	0.581967834	0.207525449
ENSGALG00010018430	0.954228362	0.235078355
ENSGALG00010018431	0.719916402	0.139460461
ENSGALG00010018432	0.50026114	0.146664567
ENSGALG00010018433	0.757067395	0.232564728
ENSGALG00010018434	0.951481588	0.231563144
ENSGALG00010018435	0.554093061	0.000155523
ENSGALG00010018436	0.249638649	0.135084198
ENSGALG00010018437	0.581904939	0.321691422
ENSGALG00010018438	0.966612103	0.27361025
ENSGALG00010018439	0.871964999	0.28429807
ENSGALG00010018440	0.957283268	0.331168264
ENSGALG00010018441	0.755835659	0.035147607
ENSGALG00010018442	0.518152467	0.294480795
ENSGALG00010018443	0.973465908	0.309957333
ENSGALG00010018444	0.681526195	0.190464461
ENSGALG00010018445	0.11424227	0.089371422
ENSGALG00010018446	0.571221408	0.466449675
ENSGALG00010018447	0.666979454	0.256711722
ENSGALG00010018448	0.989281708	0.321325364
ENSGALG00010018449	0.438958713	0.265364588
ENSGALG00010018450	0.841666987	0.275939515
ENSGALG00010018451	0.492376372	0.034852948
ENSGALG00010018452	0.865111946	0.294717534
ENSGALG00010018453	0.93785623	0.194151738
ENSGALG00010018454	0.573616664	0.213996849
ENSGALG00010018455	0.992961418	0.340117412
ENSGALG00010018457	0.459512896	0.172576762
ENSGALG00010018458	0.93963489	0.255217007
ENSGALG00010018459	0.959157336	0.276962618
ENSGALG00010018460	0.915595911	0.363632877
ENSGALG00010018461	0.592989352	0.170406447
ENSGALG00010018462	0.928229012	0.254460158

ENSGALG00010018463	0.941082455	0.314093013
ENSGALG00010018465	0.983659369	0.387876719
ENSGALG00010018466	0.985495922	0.300315083
ENSGALG00010018467	0.844095442	0.333246545
ENSGALG00010018468	0.927007695	0.319918958
ENSGALG00010018469	0.379837363	0.045397379
ENSGALG00010018470	0.690281225	0.033747074
ENSGALG00010018471	0.954957395	0.160605846
ENSGALG00010018472	0.957636501	0.195936584
ENSGALG00010018473	0.924500137	0.313478872
ENSGALG00010018474	0.791332075	0.309455302
ENSGALG00010018475	0.101450501	0.13043918
ENSGALG00010018476	0.88150855	0.308380845
ENSGALG00010018478	0.270451009	0.090541667
ENSGALG00010018480	0.509996401	0.289997197
ENSGALG00010018481	0.233285045	0.256694515
ENSGALG00010018482	0.873722099	0.324473501
ENSGALG00010018484	0.365259184	0.356937382
ENSGALG00010018485	0.922185506	0.341368997
ENSGALG00010018486	0.574907892	0.07415261
ENSGALG00010018487	0.242771369	0.092389954
ENSGALG00010018488	0.62159949	0.048618726
ENSGALG00010018489	0.487323392	0.293889626
ENSGALG00010018490	0.846309855	0.214321261
ENSGALG00010018491	0.889412208	0.410833945
ENSGALG00010018492	0.956529469	0.342782836
ENSGALG00010018493	0.83168522	0.282082934
ENSGALG00010018494	0.986962594	0.300561157
ENSGALG00010018495	0.320729011	0.144028995
ENSGALG00010018496	0.36832015	0.432096526
ENSGALG00010018497	0.155710768	0.124093449
ENSGALG00010018498	0.541988294	0.330580754
ENSGALG00010018499	0.017946301	0.123217195
ENSGALG00010018500	0.385516603	0.085383902
ENSGALG00010018501	0.193693735	0.05612844
ENSGALG00010018502	0.605796386	0.204685789
ENSGALG00010018503	0.774760229	0.418117052
ENSGALG00010018504	0.97265006	0.334219022
ENSGALG00010018505	0.119792704	0.123972007
ENSGALG00010018506	0.188768338	0.079027933
ENSGALG00010018508	0.432990893	0.170114811
ENSGALG00010018509	0.759668534	0.473464594
ENSGALG00010018510	0.702958742	0.231147391
ENSGALG00010018511	0.314192443	0.038581821
ENSGALG00010018512	0.956330986	0.319375767
ENSGALG00010018513	0.929634639	0.300489233
ENSGALG00010018514	0.644970597	0.109236815
ENSGALG00010018515	0.96838217	0.3269434
ENSGALG00010018516	0.952523049	0.386233369
ENSGALG00010018517	0.773382465	0.163922628
ENSGALG00010018518	0.652899121	0.1841315
ENSGALG00010018519	0.975514895	0.333424992
ENSGALG00010018520	0.296890738	0.383143551
ENSGALG00010018521	0.938105637	0.21977879
ENSGALG00010018522	0.289230757	0.27977729
ENSGALG00010018523	0.800755656	0.271241071

ENSGALG00010018524	0.91848643	0.368410113
ENSGALG00010018525	0.985437402	0.337227122
ENSGALG00010018526	0.605577292	0.041810821
ENSGALG00010018527	0.558829644	0.144497775
ENSGALG00010018528	0.201765669	0.224282393
ENSGALG00010018529	0.740917238	0.1816104
ENSGALG00010018530	0.81794206	0.183129466
ENSGALG00010018531	0.817813593	0.249742609
ENSGALG00010018532	0.926885967	0.304697789
ENSGALG00010018533	0.891532988	0.290511481
ENSGALG00010018534	0.890728568	0.208730592
ENSGALG00010018535	0.472593331	0.011712359
ENSGALG00010018536	0.874983417	0.257469752
ENSGALG00010018537	0.393347877	0.27953304
ENSGALG00010018539	0.836361247	0.379812779
ENSGALG00010018540	0.943274409	0.347300932
ENSGALG00010018542	0.888526394	0.28786534
ENSGALG00010018543	0.664173085	0.023798692
ENSGALG00010018544	0.990363165	0.332087094
ENSGALG00010018546	0.921717817	0.32746249
ENSGALG00010018548	0.977342837	0.348826945
ENSGALG00010018549	0.633872582	0.2992129
ENSGALG00010018551	0.810177441	0.376747227
ENSGALG00010018552	0.950279086	0.235009626
ENSGALG00010018553	0.951999596	0.339289174
ENSGALG00010018554	0.428808266	0.072056468
ENSGALG00010018555	0.776656349	0.546405835
ENSGALG00010018556	0.969339992	0.340913027
ENSGALG00010018557	0.898781581	0.238541809
ENSGALG00010018558	0.778367905	0.275376062
ENSGALG00010018559	0.903087775	0.182917331
ENSGALG00010018560	0.959413933	0.333513314
ENSGALG00010018561	0.960770801	0.344526728
ENSGALG00010018562	0.49751762	0.13639528
ENSGALG00010018563	0.424460353	0.38996328
ENSGALG00010018564	0.605052997	0.28177213
ENSGALG00010018565	0.948624906	0.299064127
ENSGALG00010018566	0.929087463	0.327538335
ENSGALG00010018568	0.556688282	0.128242977
ENSGALG00010018572	0.697073407	0.120137418
ENSGALG00010018573	0.878596962	0.202765396
ENSGALG00010018574	0.439051397	0.058346462
ENSGALG00010018575	0.157659324	0.193545808
ENSGALG00010018576	0.391138237	0.045179838
ENSGALG00010018577	0.196148984	0.112781612
ENSGALG00010018579	0.892335014	0.259910311
ENSGALG00010018581	0.797047753	0.254654497
ENSGALG00010018582	0.968287525	0.362837555
ENSGALG00010018583	0.860820497	0.47439157
ENSGALG00010018585	0.187514554	0.108479243
ENSGALG00010018587	0.968273533	0.377562706
ENSGALG00010018588	0.784933461	0.026620439
ENSGALG00010018590	0.198189675	0.600964987
ENSGALG00010018592	0.406358069	0.095586701
ENSGALG00010018593	0.962046377	0.233856409
ENSGALG00010018594	0.867516075	0.372833136

ENSGALG00010018595	0.356646144	0.112989608
ENSGALG00010018596	0.497682353	0.262695088
ENSGALG00010018597	0.893448216	0.310027168
ENSGALG00010018598	0.00194312	0.39464953
ENSGALG00010018599	0.854293767	0.295106743
ENSGALG00010018601	0.800238419	0.146996468
ENSGALG00010018602	0.841563354	0.139179334
ENSGALG00010018604	0.981687607	0.35117459
ENSGALG00010018606	0.726470279	0.352009054
ENSGALG00010018607	0.655893894	0.406490563
ENSGALG00010018608	0.939183265	0.309822725
ENSGALG00010018609	0.95400363	0.385534096
ENSGALG00010018611	0.975637653	0.330133884
ENSGALG00010018612	0.745507064	0.253676706
ENSGALG00010018613	0.353985317	0.089620528
ENSGALG00010018614	0.167788947	0.189987988
ENSGALG00010018615	0.526260727	0.311089116
ENSGALG00010018616	0.461627311	0.218392127
ENSGALG00010018617	0.154449768	0.134778997
ENSGALG00010018618	0.154952521	0.237339793
ENSGALG00010018619	0.222303042	0.406341651
ENSGALG00010018620	0.057696991	0.254212354
ENSGALG00010018621	0.364997592	0.385832511
ENSGALG00010018623	0.576735778	0.020606917
ENSGALG00010018624	0.418831742	0.347202975
ENSGALG00010018625	0.278307637	0.137000131
ENSGALG00010018627	0.987141708	0.274150574
ENSGALG00010018628	0.98697434	0.390100707
ENSGALG00010018630	0.800957158	0.28019599
ENSGALG00010018631	0.939112586	0.297608537
ENSGALG00010018633	0.941815168	0.302003639
ENSGALG00010018634	0.662392926	0.599397474
ENSGALG00010018635	0.380006087	0.091193993
ENSGALG00010018636	0.419267344	0.223422683
ENSGALG00010018638	0.305992917	0.014365182
ENSGALG00010018641	0.789113636	0.107250744
ENSGALG00010018644	0.702950742	0.178472201
ENSGALG00010018646	0.247327187	0.114340784
ENSGALG00010018649	0.603708806	0.320942568
ENSGALG00010018650	0.496354282	0.119669621
ENSGALG00010018654	0.838277634	0.238778363
ENSGALG00010018655	0.86344515	0.326335317
ENSGALG00010018658	0.298918274	0.082467963
ENSGALG00010018659	0.777141479	0.255150004
ENSGALG00010018662	0.473755225	0.073232749
ENSGALG00010018665	0.943549308	0.181452616
ENSGALG00010018668	0.886021432	0.183085252
ENSGALG00010018669	0.397516991	0.125566479
ENSGALG00010018670	0.976218113	0.314535132
ENSGALG00010018672	0.947708106	0.369364525
ENSGALG00010018673	0.916702143	0.394319237
ENSGALG00010018679	0.208550923	0.676301294
ENSGALG00010018683	0.208550923	0.676301294
ENSGALG00010018684	0.176393029	0.071464513
ENSGALG00010018685	0.685691742	0.235550476
ENSGALG00010018686	0.699102129	0.402545651

ENSGALG00010018687	0.84778351	0.379659475
ENSGALG00010018688	0.469355273	0.169426084
ENSGALG00010018689	0.446527462	0.416082474
ENSGALG00010018690	0.663174831	0.200692854
ENSGALG00010018692	0.441819133	0.039039095
ENSGALG00010018693	0.96290535	0.296489539
ENSGALG00010018694	0.727719639	0.003157501
ENSGALG00010018696	0.714922253	0.261356024
ENSGALG00010018697	0.222592387	0.008744502
ENSGALG00010018698	0.761576325	0.343511832
ENSGALG00010018699	0.881775489	0.08931558
ENSGALG00010018700	0.168674977	0.164599621
ENSGALG00010018701	0.936989634	0.297790176
ENSGALG00010018702	0.381415582	0.158761082
ENSGALG00010018704	0.807112181	0.281279979
ENSGALG00010018705	0.674203857	0.286106111
ENSGALG00010018707	0.948204603	0.38508539
ENSGALG00010018708	0.266137114	0.107784983
ENSGALG00010018709	0.955373827	0.450483143
ENSGALG00010018710	0.151941586	0.192919805
ENSGALG00010018711	0.38480368	0.180523942
ENSGALG00010018713	0.915762995	0.474527484
ENSGALG00010018715	0.751464647	0.3773513
ENSGALG00010018716	0.879677436	0.130618666
ENSGALG00010018717	0.822713393	0.351816894
ENSGALG00010018718	0.937252053	0.442919536
ENSGALG00010018719	0.978873322	0.325356699
ENSGALG00010018721	0.880626999	0.23461364
ENSGALG00010018724	0.944006893	0.254450847
ENSGALG00010018726	0.960952931	0.343236127
ENSGALG00010018727	0.982402705	0.361141793
ENSGALG00010018729	0.062647106	0.113238793
ENSGALG00010018730	0.791416476	0.162767393
ENSGALG00010018732	0.996872234	0.340631941
ENSGALG00010018733	0.109611382	0.062066971
ENSGALG00010018736	0.946414448	0.285660204
ENSGALG00010018741	0.140930048	0.25285935
ENSGALG00010018746	0.984543733	0.362420302
ENSGALG00010018747	0.884921724	0.36081032
ENSGALG00010018749	0.874403378	0.323776205
ENSGALG00010018750	0.767443589	0.144918622
ENSGALG00010018752	0.943728326	0.252856107
ENSGALG00010018753	0.973926435	0.280459437
ENSGALG00010018754	0.210737532	0.2782819
ENSGALG00010018756	0.772119388	0.359404629
ENSGALG00010018758	0.989389653	0.389287499
ENSGALG00010018759	0.02436834	0.087960992
ENSGALG00010018761	0.364062464	0.234751622
ENSGALG00010018763	0.307721442	0.16757223
ENSGALG00010018764	0.531504014	0.091306841
ENSGALG00010018767	0.965454231	0.311399593
ENSGALG00010018768	0.789288964	0.005506829
ENSGALG00010018770	0.464931148	0.111851499
ENSGALG00010018771	0.651791878	0.209806587
ENSGALG00010018773	0.712394963	0.405575087
ENSGALG00010018774	0.813404301	0.391761977

ENSGALG00010018779	0.219386918	0.079388121
ENSGALG00010018780	0.81008733	0.17544446
ENSGALG00010018782	0.610909726	0.249168689
ENSGALG00010018783	0.996700432	0.328735346
ENSGALG00010018786	0.955714767	0.256330821
ENSGALG00010018787	0.285272007	0.109169955
ENSGALG00010018791	0.271701261	0.072417625
ENSGALG00010018793	0.282406982	0.207798308
ENSGALG00010018794	0.798611229	0.279321711
ENSGALG00010018796	0.296907026	0.29766939
ENSGALG00010018799	0.477696248	0.22234344
ENSGALG00010018802	0.76286099	0.105819436
ENSGALG00010018805	0.675546319	0.213871642
ENSGALG00010018806	0.981704464	0.362032325
ENSGALG00010018807	0.272338042	0.636062138
ENSGALG00010018808	0.470442704	0.153775362
ENSGALG00010018809	0.052043394	0.148409613
ENSGALG00010018810	0.760117846	0.132548844
ENSGALG00010018811	0.98761931	0.328217619
ENSGALG00010018815	0.601111149	0.441611185
ENSGALG00010018818	0.548712388	0.414185835
ENSGALG00010018819	0.936358241	0.172216048
ENSGALG00010018821	0.521637529	0.255429938
ENSGALG00010018822	0.925324788	0.198321803
ENSGALG00010018823	0.391554564	0.254219257
ENSGALG00010018826	0.17788957	0.176536204
ENSGALG00010018827	0.052836869	0.101827534
ENSGALG00010018829	0.980285866	0.212631097
ENSGALG00010018831	0.174937087	0.339205564
ENSGALG00010018832	0.876651933	0.257282073
ENSGALG00010018833	0.122233383	0.066518296
ENSGALG00010018834	0.967895866	0.28631698
ENSGALG00010018835	0.194347177	0.260186353
ENSGALG00010018837	0.595202122	0.142912236
ENSGALG00010018838	0.129994491	0.083997967
ENSGALG00010018839	0.991523793	0.317016231
ENSGALG00010018840	0.47427365	0.157841352
ENSGALG00010018841	0.331513246	0.087475111
ENSGALG00010018842	0.189240572	0.04996112
ENSGALG00010018843	0.805806052	0.262027781
ENSGALG00010018846	0.97301304	0.296090519
ENSGALG00010018847	0.745608352	0.350294965
ENSGALG00010018848	0.990791591	0.290231376
ENSGALG00010018849	0.385618883	0.148250795
ENSGALG00010018851	0.187514554	0.108479243
ENSGALG00010018852	0.838223409	0.304289
ENSGALG00010018854	0.912196715	0.304564828
ENSGALG00010018855	0.096017235	0.130411689
ENSGALG00010018857	0.778658092	0.328995355
ENSGALG00010018858	0.949426765	0.403132551
ENSGALG00010018859	0.385953062	0.133261917
ENSGALG00010018860	0.870677566	0.438876855
ENSGALG00010018864	0.535546203	0.111251061
ENSGALG00010018865	0.617158982	0.136691966
ENSGALG00010018867	0.883729926	0.311244665
ENSGALG00010018870	0.009429422	0.134618735

ENSGALG00010018872	0.323560159	0.151171516
ENSGALG00010018873	0.378628271	0.092431246
ENSGALG00010018874	0.547547085	0.093171378
ENSGALG00010018876	0.968343007	0.277940909
ENSGALG00010018877	0.910901004	0.263327956
ENSGALG00010018879	0.240819747	0.227392974
ENSGALG00010018880	0.575777377	0.044672158
ENSGALG00010018881	0.150772323	0.206989863
ENSGALG00010018882	0.569881342	0.309321171
ENSGALG00010018883	0.977671525	0.306747367
ENSGALG00010018884	0.634592111	0.478344833
ENSGALG00010018885	0.907991563	0.384289734
ENSGALG00010018886	0.013892708	0.185886393
ENSGALG00010018887	0.375078681	0.05260243
ENSGALG00010018888	0.398716294	0.159015355
ENSGALG00010018889	0.614722612	0.250993963
ENSGALG00010018891	0.30111148	0.294565426
ENSGALG00010018892	0.987766531	0.328731458
ENSGALG00010018893	0.659278087	0.333695388
ENSGALG00010018894	0.968565895	0.414131888
ENSGALG00010018895	0.750398528	0.27374889
ENSGALG00010018896	0.535770959	0.068979637
ENSGALG00010018897	0.923596816	0.379227333
ENSGALG00010018899	0.118598935	0.089623314
ENSGALG00010018900	0.919135754	0.475461983
ENSGALG00010018901	0.627789214	0.171513431
ENSGALG00010018902	0.950866655	0.343217111
ENSGALG00010018903	0.991683601	0.306134521
ENSGALG00010018904	0.590425043	0.288321107
ENSGALG00010018905	0.873077306	0.326834841
ENSGALG00010018908	0.187514554	0.108479243
ENSGALG00010018909	0.240009845	0.061560005
ENSGALG00010018910	0.427672062	0.266490373
ENSGALG00010018911	0.915990076	0.291537727
ENSGALG00010018912	0.554029887	0.170548871
ENSGALG00010018913	0.905161159	0.183546191
ENSGALG00010018914	0.373070537	0.117661301
ENSGALG00010018915	0.930682543	0.229328927
ENSGALG00010018916	0.157439455	0.232970686
ENSGALG00010018917	0.674455934	0.529229924
ENSGALG00010018918	0.744361756	0.228350004
ENSGALG00010018919	0.925856531	0.281383642
ENSGALG00010018920	0.984903895	0.318408216
ENSGALG00010018921	0.194295924	0.153515203
ENSGALG00010018922	0.934270202	0.275249335
ENSGALG00010018923	0.961414803	0.335692235
ENSGALG00010018924	0.575824478	0.177244929
ENSGALG00010018925	0.066557466	0.010019976
ENSGALG00010018927	0.310433083	0.239339214
ENSGALG00010018928	0.165579132	0.333956354
ENSGALG00010018929	0.606278308	0.640429872
ENSGALG00010018930	0.36325893	0.316306182
ENSGALG00010018931	0.840758667	0.26697124
ENSGALG00010018932	0.923208958	0.293153652
ENSGALG00010018933	0.780720094	0.312484852
ENSGALG00010018934	0.707927097	0.242764883

ENSGALG00010018936	0.489226367	0.072243656
ENSGALG00010018937	0.033313717	0.12050399
ENSGALG00010018939	0.795836049	0.243067264
ENSGALG00010018940	0.840099865	0.42653
ENSGALG00010018941	0.994213018	0.307078522
ENSGALG00010018943	0.96936573	0.334354198
ENSGALG00010018944	0.386642026	0.442142809
ENSGALG00010018945	0.958015021	0.411698961
ENSGALG00010018946	0.271701261	0.072417625
ENSGALG00010018947	0.90939555	0.585869381
ENSGALG00010018948	0.976075598	0.322943309
ENSGALG00010018949	0.602687917	0.085122408
ENSGALG00010018950	0.983774028	0.324431927
ENSGALG00010018951	0.625124748	0.25239842
ENSGALG00010018953	0.401639624	0.101384476
ENSGALG00010018954	0.397218637	0.284730518
ENSGALG00010018955	0.976610984	0.229297638
ENSGALG00010018956	0.806968855	0.291434979
ENSGALG00010018957	0.794883116	0.336122723
ENSGALG00010018958	0.904101236	0.265005379
ENSGALG00010018959	0.971564962	0.305794677
ENSGALG00010018960	0.981993579	0.298978855
ENSGALG00010018961	0.879547636	0.272849493
ENSGALG00010018962	0.383947044	0.128562519
ENSGALG00010018963	0.738601169	0.300973049
ENSGALG00010018964	0.491445346	0.305578384
ENSGALG00010018966	0.781669233	0.079777164
ENSGALG00010018967	0.232855177	0.454557133
ENSGALG00010018968	0.951048431	0.363459742
ENSGALG00010018969	0.423189025	0.110904155
ENSGALG00010018970	0.815390905	0.258170078
ENSGALG00010018971	0.967082725	0.298333915
ENSGALG00010018972	0.645090941	0.180742242
ENSGALG00010018973	0.359638984	0.152426409
ENSGALG00010018974	0.706938987	0.022530142
ENSGALG00010018975	0.358375733	0.338828017
ENSGALG00010018977	0.959895487	0.415865326
ENSGALG00010018980	0.189240572	0.04996112
ENSGALG00010018981	0.099028626	0.079591415
ENSGALG00010018982	0.442836758	0.194898196
ENSGALG00010018983	0.570530671	0.21917747
ENSGALG00010018985	0.737024331	0.284065326
ENSGALG00010018986	0.978690966	0.261823128
ENSGALG00010018987	0.900918521	0.258215449
ENSGALG00010018990	0.291991191	0.116811562
ENSGALG00010018991	0.113555875	0.360422532
ENSGALG00010018992	0.975199533	0.334013745
ENSGALG00010018993	0.080303579	0.497702184
ENSGALG00010018997	0.17023583	0.001774827
ENSGALG00010018998	0.974945724	0.353785002
ENSGALG00010018999	0.66791336	0.146992295
ENSGALG00010019000	0.892846652	0.346891182
ENSGALG00010019001	0.96090872	0.307128398
ENSGALG00010019004	0.931237378	0.381578863
ENSGALG00010019005	0.9074545	0.385436281
ENSGALG00010019006	0.979842995	0.348490644

ENSGALG00010019008	0.958257763	0.315718497
ENSGALG00010019009	0.94752288	0.248897664
ENSGALG00010019011	0.842755081	0.037914339
ENSGALG00010019012	0.964080107	0.264899543
ENSGALG00010019013	0.865839186	0.163531797
ENSGALG00010019014	0.295081293	0.447565639
ENSGALG00010019015	0.326066916	0.174218685
ENSGALG00010019016	0.966275812	0.388815178
ENSGALG00010019017	0.957252732	0.257880854
ENSGALG00010019018	0.926040697	0.249626141
ENSGALG00010019019	0.881711356	0.202027939
ENSGALG00010019020	0.875370791	0.194920877
ENSGALG00010019021	0.521791128	0.348402103
ENSGALG00010019023	0.262410674	0.206406221
ENSGALG00010019024	0.926597137	0.345544175
ENSGALG00010019025	0.92200619	0.306055302
ENSGALG00010019026	0.915367999	0.25157971
ENSGALG00010019027	0.947203633	0.296004694
ENSGALG00010019028	0.904047918	0.255923183
ENSGALG00010019029	0.647232203	0.00213186
ENSGALG00010019030	0.533449398	0.047459005
ENSGALG00010019032	0.966805935	0.407574338
ENSGALG00010019033	0.38480368	0.180523942
ENSGALG00010019034	0.966786854	0.345637241
ENSGALG00010019035	0.7047876	0.310130893
ENSGALG00010019036	0.779525619	0.292027315
ENSGALG00010019038	0.32144915	0.012786713
ENSGALG00010019040	0.916471928	0.325049571
ENSGALG00010019041	0.301507978	0.081049267
ENSGALG00010019042	0.544635693	0.393481026
ENSGALG00010019043	0.103884326	0.060421836
ENSGALG00010019044	0.277566769	0.107857127
ENSGALG00010019045	0.997976491	0.325427191
ENSGALG00010019046	0.001915583	0.038512321
ENSGALG00010019047	0.105081013	0.114717373
ENSGALG00010019049	0.874844802	0.288814279
ENSGALG00010019050	0.003329705	0.110284121
ENSGALG00010019051	0.956911359	0.266973305
ENSGALG00010019052	0.567647804	0.419756545
ENSGALG00010019053	0.645542203	0.140728894
ENSGALG00010019058	0.111723115	0.155918956
ENSGALG00010019059	0.939154025	0.379093755
ENSGALG00010019060	0.580283279	0.16117949
ENSGALG00010019061	0.374419648	0.109023883
ENSGALG00010019062	0.468093952	0.429360409
ENSGALG00010019063	0.355639478	0.141537604
ENSGALG00010019064	0.224165794	0.101285947
ENSGALG00010019065	0.997339361	0.321679337
ENSGALG00010019067	0.289652683	0.380292044
ENSGALG00010019068	0.95007601	0.282467658
ENSGALG00010019069	0.333634937	0.155730388
ENSGALG00010019072	0.805531489	0.522985589
ENSGALG00010019073	0.086476784	0.029149839
ENSGALG00010019076	0.61607162	0.452256617
ENSGALG00010019077	0.580995155	0.035643329
ENSGALG00010019078	0.579332134	0.197981249

ENSGALG00010019079	0.475715639	0.171211702
ENSGALG00010019080	0.276941659	0.150920925
ENSGALG00010019081	0.956693442	0.23236151
ENSGALG00010019082	0.928779629	0.305089067
ENSGALG00010019083	0.694385691	0.074118005
ENSGALG00010019084	0.477096086	0.175579694
ENSGALG00010019086	0.203991683	0.058221925
ENSGALG00010019087	0.927829949	0.373333627
ENSGALG00010019088	0.596856531	0.13887257
ENSGALG00010019093	0.935559974	0.317814833
ENSGALG00010019094	0.82746688	0.36680545
ENSGALG00010019096	0.33040952	0.152955128
ENSGALG00010019097	0.295416887	0.201236025
ENSGALG00010019099	0.339843734	0.219060419
ENSGALG00010019100	0.393293574	0.180300648
ENSGALG00010019101	0.836609247	0.508939629
ENSGALG00010019102	0.466560581	0.137287145
ENSGALG00010019103	0.630782635	0.443080753
ENSGALG00010019104	0.822686563	0.258783481
ENSGALG00010019106	0.103173471	0.274363727
ENSGALG00010019107	0.324020854	0.340314887
ENSGALG00010019108	0.82313181	0.332494807
ENSGALG00010019109	0.017946301	0.123217195
ENSGALG00010019110	0.956066819	0.30756257
ENSGALG00010019111	0.285272007	0.109169955
ENSGALG00010019112	0.427497446	0.200374047
ENSGALG00010019113	0.736013186	0.033865451
ENSGALG00010019114	0.58023346	0.402537575
ENSGALG00010019115	0.37186876	0.224920886
ENSGALG00010019116	0.799215624	0.128147167
ENSGALG00010019117	0.894242581	0.348343693
ENSGALG00010019120	0.93809383	0.347116141
ENSGALG00010019121	0.225881852	0.168191494
ENSGALG00010019123	0.931008283	0.191790267
ENSGALG00010019124	0.917641602	0.341526686
ENSGALG00010019125	0.611644498	0.3433324
ENSGALG00010019126	0.921537936	0.347231242
ENSGALG00010019127	0.8083089	0.282707086
ENSGALG00010019128	0.947931201	0.317573113
ENSGALG00010019130	0.92848771	0.185535724
ENSGALG00010019131	0.705631249	0.294174373
ENSGALG00010019132	0.952469454	0.31646251
ENSGALG00010019133	0.216433634	0.680598293
ENSGALG00010019134	0.16910645	0.545330104
ENSGALG00010019135	0.296265053	0.120759158
ENSGALG00010019136	0.168179534	0.388908483
ENSGALG00010019138	0.356980791	0.3524601
ENSGALG00010019139	0.439556067	0.175744343
ENSGALG00010019140	0.286755814	0.501844446
ENSGALG00010019141	0.341306915	0.054975724
ENSGALG00010019143	0.325929383	0.143359216
ENSGALG00010019144	0.687080181	0.7102957
ENSGALG00010019145	0.885033492	0.236895055
ENSGALG00010019146	0.991668134	0.336639034
ENSGALG00010019147	0.270260157	0.18667361
ENSGALG00010019148	0.446413027	0.174454307

ENSGALG00010019149	0.980393123	0.330860909
ENSGALG00010019152	0.734955618	0.008034594
ENSGALG00010019155	0.763104523	0.284755224
ENSGALG00010019156	0.200570274	0.091667991
ENSGALG00010019157	0.31155034	0.03137051
ENSGALG00010019158	0.871937246	0.412499128
ENSGALG00010019160	0.219386918	0.079388121
ENSGALG00010019161	0.214000092	0.162332731
ENSGALG00010019163	0.036215381	0.132705637
ENSGALG00010019164	0.48486274	0.087297481
ENSGALG00010019166	0.873167455	0.199206327
ENSGALG00010019168	0.292794004	0.478363193
ENSGALG00010019169	0.118149305	0.076199018
ENSGALG00010019171	0.722581579	0.353345307
ENSGALG00010019172	0.924379694	0.16122084
ENSGALG00010019173	0.772048074	0.274065654
ENSGALG00010019174	0.968687262	0.333042312
ENSGALG00010019175	0.542793897	0.310904867
ENSGALG00010019176	0.126447213	0.085856668
ENSGALG00010019178	0.1025044	0.187932477
ENSGALG00010019180	0.090998159	0.09480036
ENSGALG00010019182	0.22560639	0.280052113
ENSGALG00010019183	0.771536514	0.332185659
ENSGALG00010019184	0.921100052	0.301912013
ENSGALG00010019185	0.976113162	0.277686762
ENSGALG00010019186	0.968741769	0.397571835
ENSGALG00010019187	0.285272007	0.109169955
ENSGALG00010019188	0.658546451	0.246847922
ENSGALG00010019189	0.370422358	0.310252012
ENSGALG00010019190	0.010610177	0.193088893
ENSGALG00010019193	0.757705459	0.236551853
ENSGALG00010019194	0.864670686	0.430272211
ENSGALG00010019195	0.048269049	0.154346147
ENSGALG00010019200	0.311541422	0.175102084
ENSGALG00010019204	0.899573913	0.236514309
ENSGALG00010019205	0.074772865	0.02402643
ENSGALG00010019206	0.905087096	0.365522692
ENSGALG00010019208	0.571951029	0.314218538
ENSGALG00010019209	0.814555117	0.354478482
ENSGALG00010019212	0.776729833	0.376686723
ENSGALG00010019213	0.626615374	0.120418318
ENSGALG00010019215	0.412072705	0.174752596
ENSGALG00010019218	0.169808299	0.037463341
ENSGALG00010019220	0.067239119	0.578238882
ENSGALG00010019221	0.85445504	0.216446279
ENSGALG00010019223	0.821033135	0.049690242
ENSGALG00010019225	0.940797562	0.393642266
ENSGALG00010019226	0.264589269	0.150326967
ENSGALG00010019227	0.960291367	0.296287513
ENSGALG00010019229	0.389704111	0.119474745
ENSGALG00010019230	0.972856985	0.315378881
ENSGALG00010019232	0.504963247	0.116614512
ENSGALG00010019233	0.918743838	0.213466206
ENSGALG00010019234	0.043544722	0.088274573
ENSGALG00010019235	0.616664892	0.237403631
ENSGALG00010019236	0.818636588	0.371999744

ENSGALG00010019237	0.408248733	0.341871092
ENSGALG00010019238	0.044165284	0.108128939
ENSGALG00010019239	0.79988127	0.238236512
ENSGALG00010019240	0.451472003	0.268942556
ENSGALG00010019243	0.865654102	0.213955554
ENSGALG00010019245	0.662938251	0.27795676
ENSGALG00010019246	0.890785492	0.336419163
ENSGALG00010019247	0.400215036	0.132215663
ENSGALG00010019248	0.844805343	0.246378922
ENSGALG00010019249	0.359626391	0.02035457
ENSGALG00010019250	0.378403277	0.149830149
ENSGALG00010019252	0.902358482	0.306906414
ENSGALG00010019253	0.124989663	0.129465107
ENSGALG00010019254	0.846340405	0.204321318
ENSGALG00010019256	0.7074612	0.380341784
ENSGALG00010019257	0.883734288	0.141049471
ENSGALG00010019258	0.90786892	0.370208848
ENSGALG00010019259	0.824291272	0.2684609
ENSGALG00010019260	0.391520552	0.065399671
ENSGALG00010019262	0.445028333	0.041401531
ENSGALG00010019263	0.958720943	0.271463667
ENSGALG00010019264	0.386720587	0.038989607
ENSGALG00010019265	0.427394042	0.171353786
ENSGALG00010019266	0.434452305	0.050078324
ENSGALG00010019267	0.965630378	0.268877978
ENSGALG00010019268	0.817352818	0.226152439
ENSGALG00010019269	0.900216718	0.308639018
ENSGALG00010019270	0.959275664	0.364365121
ENSGALG00010019271	0.815363098	0.419301299
ENSGALG00010019273	0.755876762	0.37290288
ENSGALG00010019274	0.324020854	0.340314887
ENSGALG00010019275	0.715660326	0.294216032
ENSGALG00010019276	0.068933014	0.036514652
ENSGALG00010019277	0.885154398	0.350701277
ENSGALG00010019278	0.460468218	0.102759802
ENSGALG00010019279	0.182855371	0.171838264
ENSGALG00010019280	0.791205139	0.273501809
ENSGALG00010019282	0.867605119	0.269082354
ENSGALG00010019283	0.986366764	0.323737835
ENSGALG00010019284	0.886257423	0.249045919
ENSGALG00010019285	0.920776805	0.321089854
ENSGALG00010019286	0.025513556	0.004025169
ENSGALG00010019287	0.725446276	0.226576035
ENSGALG00010019288	0.937656417	0.30967369
ENSGALG00010019289	0.824214937	0.344420708
ENSGALG00010019290	0.258836631	0.282706581
ENSGALG00010019291	0.701818988	0.254275579
ENSGALG00010019292	0.977441199	0.365179554
ENSGALG00010019294	0.401606939	0.133521938
ENSGALG00010019295	0.883459671	0.435598426
ENSGALG00010019296	0.506003672	0.137821335
ENSGALG00010019297	0.935183876	0.368896888
ENSGALG00010019298	0.804665833	0.318092269
ENSGALG00010019299	0.459629367	0.692775726
ENSGALG00010019300	0.088415256	0.045818649
ENSGALG00010019301	0.491881344	0.145226145

ENSGALG00010019302	0.796249866	0.377839757
ENSGALG00010019303	0.549653586	0.066014554
ENSGALG00010019304	0.698453726	0.23805344
ENSGALG00010019305	0.983001965	0.322642379
ENSGALG00010019306	0.003616255	0.020981987
ENSGALG00010019307	0.822629003	0.191522436
ENSGALG00010019308	0.263763053	0.168119672
ENSGALG00010019310	0.890052809	0.361530558
ENSGALG00010019311	0.662362941	0.233865915
ENSGALG00010019312	0.608599366	0.222728219
ENSGALG00010019313	0.587756186	0.357924034
ENSGALG00010019315	0.399864362	0.02586642
ENSGALG00010019316	0.845583266	0.256145363
ENSGALG00010019317	0.642880085	0.173144998
ENSGALG00010019318	0.944706407	0.324994022
ENSGALG00010019319	0.983459226	0.336004437
ENSGALG00010019322	0.757376469	0.426969565
ENSGALG00010019323	0.626194708	0.21073538
ENSGALG00010019325	0.863605738	0.224896785
ENSGALG00010019326	0.063606688	0.124346693
ENSGALG00010019327	0.042405664	0.223332755
ENSGALG00010019328	0.050216828	0.232292189
ENSGALG00010019329	0.31793183	0.229053843
ENSGALG00010019330	0.147390775	0.391670696
ENSGALG00010019331	0.97603812	0.352984137
ENSGALG00010019333	0.912727819	0.240100858
ENSGALG00010019334	0.84268767	0.222742535
ENSGALG00010019335	0.911434054	0.347893129
ENSGALG00010019336	0.897499877	0.287688206
ENSGALG00010019337	0.825698036	0.374766722
ENSGALG00010019339	0.582285199	0.028510764
ENSGALG00010019340	0.391460109	0.674779943
ENSGALG00010019341	0.297090418	0.136289521
ENSGALG00010019342	0.783668658	0.42142033
ENSGALG00010019343	0.270451009	0.090541667
ENSGALG00010019344	0.476789055	0.108463039
ENSGALG00010019345	0.892846707	0.303605443
ENSGALG00010019346	0.264239718	0.073427965
ENSGALG00010019347	0.637415015	0.326486369
ENSGALG00010019348	0.542405948	0.147375687
ENSGALG00010019349	0.660836626	0.097124777
ENSGALG00010019350	0.882528593	0.243362895
ENSGALG00010019352	0.457010693	0.302962748
ENSGALG00010019353	0.446789332	0.270690673
ENSGALG00010019355	0.835970205	0.110759811
ENSGALG00010019356	0.960222239	0.258069982
ENSGALG00010019357	0.764207149	0.09670652
ENSGALG00010019359	0.892282153	0.172762084
ENSGALG00010019360	0.924050446	0.214674583
ENSGALG00010019361	0.436167963	0.205943322
ENSGALG00010019362	0.352108103	0.037003535
ENSGALG00010019363	0.777326144	0.314735513
ENSGALG00010019365	0.472951924	0.10312309
ENSGALG00010019366	0.504189449	0.301178594
ENSGALG00010019368	0.402344685	0.144981318
ENSGALG00010019370	0.433328622	0.366830239

ENSGALG00010019371	0.887386211	0.209851784
ENSGALG00010019372	0.081151286	0.17285279
ENSGALG00010019373	0.294956036	0.384269906
ENSGALG00010019375	0.247842294	0.110509314
ENSGALG00010019376	0.949684318	0.374573436
ENSGALG00010019378	0.387012569	0.137721351
ENSGALG00010019379	0.67325979	0.327127743
ENSGALG00010019381	0.854283758	0.346542087
ENSGALG00010019384	0.320112398	0.076847516
ENSGALG00010019385	0.35255689	0.488340344
ENSGALG00010019386	0.193220178	0.353700786
ENSGALG00010019387	0.774743206	0.165741627
ENSGALG00010019388	0.749698576	0.594904244
ENSGALG00010019390	0.450459874	0.284506407
ENSGALG00010019391	0.973143816	0.3214346
ENSGALG00010019392	0.159562845	0.284588947
ENSGALG00010019397	0.636980851	0.188245851
ENSGALG00010019398	0.80241582	0.379643268
ENSGALG00010019399	0.643816463	0.374369933
ENSGALG00010019401	0.912600428	0.37727731
ENSGALG00010019403	0.291370056	0.315671263
ENSGALG00010019405	0.517999275	0.149539616
ENSGALG00010019408	0.066975135	0.131726206
ENSGALG00010019409	0.331509393	0.156730178
ENSGALG00010019411	0.599820337	0.366246803
ENSGALG00010019412	0.915282604	0.273567615
ENSGALG00010019414	0.384831073	0.205747021
ENSGALG00010019415	0.229431786	0.758985273
ENSGALG00010019416	0.958784303	0.212032197
ENSGALG00010019417	0.219006177	0.189840674
ENSGALG00010019418	0.968206622	0.358076486
ENSGALG00010019419	0.153340062	0.151847247
ENSGALG00010019420	0.019832685	0.161555892
ENSGALG00010019422	0.822927429	0.161571525
ENSGALG00010019423	0.59703398	0.381592162
ENSGALG00010019424	0.265558089	0.028821141
ENSGALG00010019426	0.846783867	0.264649861
ENSGALG00010019428	0.150872385	0.266006492
ENSGALG00010019429	0.07074662	0.222051839
ENSGALG00010019430	0.39047417	0.286719999
ENSGALG00010019431	0.992306158	0.323499149
ENSGALG00010019432	0.265624263	0.242681341
ENSGALG00010019433	0.395556858	0.102965232
ENSGALG00010019434	0.376995479	0.131258754
ENSGALG00010019438	0.874469236	0.2764168
ENSGALG00010019441	0.864582626	0.368307861
ENSGALG00010019443	0.799530531	0.000894555
ENSGALG00010019446	0.3238092	0.228042266
ENSGALG00010019447	0.008043983	0.110635211
ENSGALG00010019448	0.379394869	0.170237467
ENSGALG00010019449	0.008984826	0.14972573
ENSGALG00010019451	0.998629169	0.341302834
ENSGALG00010019454	0.040262459	0.118277088
ENSGALG00010019456	0.346756653	0.20843815
ENSGALG00010019458	0.526545625	0.100600065
ENSGALG00010019459	0.399453749	0.144668892

ENSGALG00010019461	0.902927678	0.255224267
ENSGALG00010019463	0.838475031	0.297886394
ENSGALG00010019464	0.139919825	0.215228643
ENSGALG00010019470	0.950517971	0.281990413
ENSGALG00010019471	0.7601619	0.177896514
ENSGALG00010019472	0.985130221	0.311648267
ENSGALG00010019473	0.697164731	0.087845869
ENSGALG00010019474	0.120588646	0.036127785
ENSGALG00010019477	0.573510946	0.180539433
ENSGALG00010019480	0.595755189	0.011331714
ENSGALG00010019481	0.947359328	0.270875898
ENSGALG00010019482	0.436840389	0.147435237
ENSGALG00010019483	0.297116197	0.41599158
ENSGALG00010019484	0.868634678	0.277812414
ENSGALG00010019485	0.556485924	0.226321444
ENSGALG00010019487	0.951643457	0.265962418
ENSGALG00010019488	0.504020497	0.141821405
ENSGALG00010019489	0.309655755	0.987979031
ENSGALG00010019490	0.198078233	0.140231658
ENSGALG00010019491	0.592469137	0.161344344
ENSGALG00010019492	0.477215269	0.036619118
ENSGALG00010019493	0.870617313	0.457355406
ENSGALG00010019494	0.213034228	0.217907685
ENSGALG00010019495	0.919612908	0.375589762
ENSGALG00010019496	0.150783357	0.138472589
ENSGALG00010019498	0.964276808	0.246474319
ENSGALG00010019500	0.488774598	0.153390563
ENSGALG00010019501	0.386764803	0.105517621
ENSGALG00010019502	0.529513804	0.184251181
ENSGALG00010019504	0.77432206	0.138991993
ENSGALG00010019505	0.069749942	0.335536912
ENSGALG00010019506	0.264239718	0.073427965
ENSGALG00010019509	0.986956137	0.331604483
ENSGALG00010019510	0.256828349	0.119064955
ENSGALG00010019512	0.216433634	0.680598293
ENSGALG00010019513	0.951579747	0.309193281
ENSGALG00010019515	0.283339801	0.139137713
ENSGALG00010019516	0.011512255	0.172988189
ENSGALG00010019520	0.697582504	0.09360967
ENSGALG00010019521	0.847223003	0.386473972
ENSGALG00010019522	0.751579775	0.162790379
ENSGALG00010019524	0.789053322	0.251760866
ENSGALG00010019526	0.18918263	0.131669257
ENSGALG00010019527	0.122535307	0.201969958
ENSGALG00010019529	0.96188296	0.318497384
ENSGALG00010019532	0.359572359	0.215759064
ENSGALG00010019533	0.012433676	0.134633327
ENSGALG00010019534	0.894788581	0.052035262
ENSGALG00010019536	0.728382158	0.225640642
ENSGALG00010019539	0.591137526	0.538817871
ENSGALG00010019540	0.501401869	0.079033641
ENSGALG00010019541	0.875419723	0.402359334
ENSGALG00010019542	0.187679389	0.434625542
ENSGALG00010019543	0.503625771	0.167124734
ENSGALG00010019546	0.977536216	0.329085948
ENSGALG00010019547	0.685122725	0.3271086

ENSGALG00010019549	0.641400114	0.218366777
ENSGALG00010019550	0.909871949	0.553681489
ENSGALG00010019554	0.791191039	0.321415103
ENSGALG00010019557	0.871769954	0.276883388
ENSGALG00010019561	0.657529483	0.420613029
ENSGALG00010019563	0.656363414	0.528448183
ENSGALG00010019564	0.387967253	0.165125476
ENSGALG00010019566	0.781009668	0.212677142
ENSGALG00010019569	0.007468656	0.049904393
ENSGALG00010019570	0.131354461	0.148795542
ENSGALG00010019572	0.642951836	0.391722066
ENSGALG00010019574	0.402235172	0.110063183
ENSGALG00010019576	0.861403467	0.471066783
ENSGALG00010019577	0.854966497	0.46095803
ENSGALG00010019578	0.277003752	0.123429704
ENSGALG00010019580	0.798989435	0.355220355
ENSGALG00010019581	0.204000582	0.195456204
ENSGALG00010019583	0.087838418	0.172074926
ENSGALG00010019584	0.042410527	0.304346966
ENSGALG00010019585	0.76668054	0.235748522
ENSGALG00010019586	0.982348165	0.321476432
ENSGALG00010019587	0.87122226	0.289203758
ENSGALG00010019588	0.994175815	0.297069257
ENSGALG00010019591	0.972796868	0.385616864
ENSGALG00010019592	0.874956951	0.333937612
ENSGALG00010019593	0.265558089	0.028821141
ENSGALG00010019594	0.1220949	0.321728692
ENSGALG00010019595	0.194323177	0.15612226
ENSGALG00010019596	0.273556489	0.254934839
ENSGALG00010019598	0.169093436	0.185905303
ENSGALG00010019600	0.515990997	0.25740723
ENSGALG00010019601	0.556123064	0.103073039
ENSGALG00010019602	0.984550729	0.330305215
ENSGALG00010019603	0.786931478	0.288972551
ENSGALG00010019604	0.713363648	0.368966409
ENSGALG00010019605	0.347317946	0.329723052
ENSGALG00010019606	0.286231124	0.564253946
ENSGALG00010019607	0.218358475	0.234724241
ENSGALG00010019609	0.178596619	0.267662718
ENSGALG00010019610	0.037000306	0.026150352
ENSGALG00010019611	0.397604372	0.185366621
ENSGALG00010019612	0.096276908	0.128250401
ENSGALG00010019613	0.749292232	0.060111168
ENSGALG00010019614	0.91388052	0.207824511
ENSGALG00010019615	0.449851794	0.288683491
ENSGALG00010019617	0.508583297	0.323843252
ENSGALG00010019618	0.555722965	0.413006886
ENSGALG00010019619	0.502673267	0.17289254
ENSGALG00010019620	0.719473207	0.321758217
ENSGALG00010019621	0.996279284	0.334988911
ENSGALG00010019622	0.731349986	0.32699825
ENSGALG00010019623	0.559033941	0.341316387
ENSGALG00010019624	0.660257177	0.33128696
ENSGALG00010019625	0.346497454	0.186692691
ENSGALG00010019626	0.794887085	0.309482466
ENSGALG00010019630	0.526182145	0.487723413

ENSGALG00010019633	0.120837503	0.12463419
ENSGALG00010019634	0.705631159	0.140691706
ENSGALG00010019635	0.539877438	0.180246127
ENSGALG00010019636	0.260737039	0.071294785
ENSGALG00010019638	0.12557176	0.257281993
ENSGALG00010019639	0.447708034	0.006632806
ENSGALG00010019640	0.669681752	0.243801879
ENSGALG00010019641	0.98105011	0.353298628
ENSGALG00010019642	0.975209175	0.36033124
ENSGALG00010019643	0.673550183	0.217712688
ENSGALG00010019644	0.188136	0.203869654
ENSGALG00010019645	0.512700534	0.249786058
ENSGALG00010019646	0.991533908	0.357166991
ENSGALG00010019647	0.451226839	0.07510809
ENSGALG00010019648	0.464015477	0.103819775
ENSGALG00010019649	0.845700649	0.435351041
ENSGALG00010019650	0.82344564	0.328207759
ENSGALG00010019652	0.952893608	0.251474879
ENSGALG00010019653	0.12782002	0.079061164
ENSGALG00010019654	0.066636898	0.034508313
ENSGALG00010019655	0.994458757	0.32562902
ENSGALG00010019656	0.034711552	0.174561793
ENSGALG00010019657	0.215539175	0.267155037
ENSGALG00010019659	0.962756718	0.296546883
ENSGALG00010019660	0.124208349	0.231610899
ENSGALG00010019661	0.178023947	0.015309573
ENSGALG00010019663	0.118889497	0.169789476
ENSGALG00010019665	0.243318161	0.127581427
ENSGALG00010019666	0.729383263	0.217233313
ENSGALG00010019667	0.447561227	0.024542757
ENSGALG00010019668	0.449833658	0.266381134
ENSGALG00010019669	0.096001156	0.423019274
ENSGALG00010019670	0.767503164	0.27766659
ENSGALG00010019671	0.530466824	0.043131505
ENSGALG00010019673	0.866690025	0.450190535
ENSGALG00010019674	0.69439929	0.27173476
ENSGALG00010019675	0.058334703	0.32478189
ENSGALG00010019676	0.361355167	0.130059372
ENSGALG00010019679	0.110708131	0.163989027
ENSGALG00010019680	0.902428937	0.268203524
ENSGALG00010019682	0.660930774	0.375275974
ENSGALG00010019683	0.960483363	0.505145002
ENSGALG00010019684	0.772755404	0.401822483
ENSGALG00010019690	0.861814663	0.335693848
ENSGALG00010019691	0.270451009	0.090541667
ENSGALG00010019692	0.262631387	0.271769228
ENSGALG00010019693	0.886558462	0.168828938
ENSGALG00010019694	0.992617593	0.34573777
ENSGALG00010019696	0.979256079	0.273177585
ENSGALG00010019697	0.984804863	0.289469805
ENSGALG00010019698	0.990213226	0.359571497
ENSGALG00010019699	0.22050683	0.289579243
ENSGALG00010019700	0.473811722	0.106344856
ENSGALG00010019702	0.235205039	0.228774835
ENSGALG00010019703	0.689803208	0.199812012
ENSGALG00010019704	0.686765439	0.104420521

ENSGALG00010019705	0.438496929	0.085491879
ENSGALG00010019706	0.505035128	0.226798462
ENSGALG00010019708	0.312521416	0.270764182
ENSGALG00010019709	0.029372996	0.136538814
ENSGALG00010019710	0.619259653	0.32710741
ENSGALG00010019711	0.264239718	0.073427965
ENSGALG00010019712	0.489148369	0.15157912
ENSGALG00010019713	0.724464196	0.115003174
ENSGALG00010019714	0.488364789	0.178119529
ENSGALG00010019716	0.799153579	0.26315868
ENSGALG00010019717	0.585049779	0.30584014
ENSGALG00010019718	0.118666798	0.010778759
ENSGALG00010019722	0.250038398	0.197820031
ENSGALG00010019723	0.972209331	0.254082397
ENSGALG00010019724	0.845952158	0.267780353
ENSGALG00010019725	0.986290639	0.402216277
ENSGALG00010019726	0.98885097	0.328534067
ENSGALG00010019727	0.728227632	0.228816981
ENSGALG00010019729	0.201702771	0.045897305
ENSGALG00010019730	0.288220476	0.161651656
ENSGALG00010019731	0.958173478	0.270849178
ENSGALG00010019732	0.215763709	0.003063604
ENSGALG00010019733	0.249977783	0.036254529
ENSGALG00010019734	0.355783338	0.182683574
ENSGALG00010019735	0.189893275	0.238180505
ENSGALG00010019736	0.265558089	0.028821141
ENSGALG00010019738	0.480546695	0.070494589
ENSGALG00010019741	0.694368256	0.010370289
ENSGALG00010019743	0.994532709	0.325705153
ENSGALG00010019744	0.688298933	0.155483314
ENSGALG00010019747	0.941764272	0.164407644
ENSGALG00010019751	0.0231524	0.47459642
ENSGALG00010019752	0.93835768	0.235409371
ENSGALG00010019754	0.357744648	0.403997765
ENSGALG00010019755	0.888752294	0.327751232
ENSGALG00010019758	0.332069977	0.462193367
ENSGALG00010019761	0.861965929	0.111534364
ENSGALG00010019763	0.668555284	0.085420865
ENSGALG00010019764	0.995515063	0.332219641
ENSGALG00010019767	0.983736427	0.344624793
ENSGALG00010019770	0.097308482	0.337550935
ENSGALG00010019771	0.979396327	0.292050386
ENSGALG00010019772	0.963107819	0.349765373
ENSGALG00010019773	0.287420179	0.104388345
ENSGALG00010019774	0.246789093	0.173070302
ENSGALG00010019775	0.994344924	0.330818385
ENSGALG00010019777	0.154731256	0.464287855
ENSGALG00010019778	0.175549973	0.046711546
ENSGALG00010019779	0.288888964	0.235006592
ENSGALG00010019780	0.075484733	0.135992864
ENSGALG00010019781	0.182591789	0.203069719
ENSGALG00010019785	0.993635693	0.393462051
ENSGALG00010019786	0.994205653	0.325442035
ENSGALG00010019789	0.50515211	0.031313277
ENSGALG00010019792	0.294661357	0.109904448
ENSGALG00010019793	0.804346819	0.240143709

ENSGALG00010019794	0.62923367	0.065848748
ENSGALG00010019798	0.300390954	0.171341096
ENSGALG00010019799	0.863885427	0.101965272
ENSGALG00010019800	0.978694132	0.212571511
ENSGALG00010019801	0.943471308	0.352548327
ENSGALG00010019802	0.233207078	0.190025168
ENSGALG00010019803	0.987560743	0.318982376
ENSGALG00010019804	0.702698702	0.344679863
ENSGALG00010019807	0.907357314	0.268484173
ENSGALG00010019808	0.285179533	0.121954912
ENSGALG00010019810	0.439768451	0.097952257
ENSGALG00010019811	0.778294233	0.231230227
ENSGALG00010019812	0.546136267	0.298039469
ENSGALG00010019813	0.982932582	0.30771775
ENSGALG00010019816	0.954023928	0.20728608
ENSGALG00010019818	0.518946868	0.279639876
ENSGALG00010019820	0.933998319	0.393613668
ENSGALG00010019823	0.749168112	0.264595191
ENSGALG00010019825	0.944661148	0.235766754
ENSGALG00010019828	0.87462367	0.15477305
ENSGALG00010019829	0.897688447	0.321610876
ENSGALG00010019830	0.980680018	0.283769036
ENSGALG00010019832	0.965738721	0.272829635
ENSGALG00010019833	0.970208363	0.279425654
ENSGALG00010019834	0.930603404	0.2139387
ENSGALG00010019836	0.629422924	0.156780531
ENSGALG00010019838	0.718173646	0.685083237
ENSGALG00010019839	0.828492259	0.27411951
ENSGALG00010019842	0.422889611	0.302620283
ENSGALG00010019843	0.214413232	0.061734559
ENSGALG00010019844	0.94272879	0.218100622
ENSGALG00010019845	0.658585938	0.280618612
ENSGALG00010019846	0.302093679	0.200883588
ENSGALG00010019847	0.053422351	0.093673139
ENSGALG00010019848	0.254773239	0.043440209
ENSGALG00010019849	0.933498773	0.297230343
ENSGALG00010019851	0.665416918	0.297620943
ENSGALG00010019852	0.286212763	0.139913221
ENSGALG00010019853	0.408661366	0.194193874
ENSGALG00010019854	0.119618822	0.084577823
ENSGALG00010019855	0.555043349	0.131280367
ENSGALG00010019856	0.142674579	0.492022664
ENSGALG00010019857	0.245700908	0.010899108
ENSGALG00010019859	0.659693034	0.095934823
ENSGALG00010019861	0.566754197	0.518680502
ENSGALG00010019864	0.388899421	0.120062276
ENSGALG00010019865	0.971331893	0.343841153
ENSGALG00010019867	0.967937304	0.262297941
ENSGALG00010019868	0.274586994	0.115798603
ENSGALG00010019869	0.82762102	0.347861432
ENSGALG00010019870	0.200570274	0.091667991
ENSGALG00010019872	0.939013859	0.227781702
ENSGALG00010019873	0.968342661	0.300934921
ENSGALG00010019875	0.927433889	0.401183897
ENSGALG00010019876	0.690504962	0.144641915
ENSGALG00010019877	0.335339564	0.294710305

ENSGALG00010019878	0.981053641	0.408633039
ENSGALG00010019880	0.025739541	0.105032112
ENSGALG00010019886	0.514649421	0.364338745
ENSGALG00010019887	0.795311838	0.271251513
ENSGALG00010019888	0.573795379	0.135681927
ENSGALG00010019889	0.380458755	0.122221739
ENSGALG00010019890	0.765777911	0.260020673
ENSGALG00010019891	0.822712559	0.23047447
ENSGALG00010019892	0.079237238	0.103487154
ENSGALG00010019893	0.36117416	0.054562941
ENSGALG00010019895	0.083417541	0.625326881
ENSGALG00010019896	0.974983205	0.337527453
ENSGALG00010019897	0.57615418	0.219426346
ENSGALG00010019898	0.816848385	0.273189707
ENSGALG00010019899	0.961496306	0.324280801
ENSGALG00010019900	0.187176172	0.2981231
ENSGALG00010019901	0.318813342	0.16516172
ENSGALG00010019902	0.187514554	0.108479243
ENSGALG00010019905	0.336366535	0.073523437
ENSGALG00010019906	0.891585199	0.217000451
ENSGALG00010019908	0.604430696	0.028134881
ENSGALG00010019909	0.213883565	0.238548392
ENSGALG00010019910	0.492681882	0.312314194
ENSGALG00010019912	0.513207262	0.138554333
ENSGALG00010019914	0.409100652	0.202453273
ENSGALG00010019915	0.266137114	0.107784983
ENSGALG00010019916	0.716531546	0.301892511
ENSGALG00010019917	0.678321431	0.007390911
ENSGALG00010019919	0.90176753	0.282329874
ENSGALG00010019920	0.249295355	0.150040874
ENSGALG00010019921	0.980024865	0.317849545
ENSGALG00010019922	0.596365203	0.190795924
ENSGALG00010019923	0.988952236	0.339724564
ENSGALG00010019924	0.300017658	0.583254388
ENSGALG00010019925	0.966329742	0.312537262
ENSGALG00010019926	0.87004873	0.105606226
ENSGALG00010019929	0.078695333	0.302709344
ENSGALG00010019930	0.160827757	0.488970864
ENSGALG00010019931	0.975675243	0.354629155
ENSGALG00010019932	0.908470869	0.398229665
ENSGALG00010019934	0.898036135	0.365884497
ENSGALG00010019935	0.101536066	0.037078272
ENSGALG00010019936	0.392748352	0.445400077
ENSGALG00010019937	0.338714282	0.13932343
ENSGALG00010019939	0.244796653	0.206273058
ENSGALG00010019940	0.467414401	0.136298694
ENSGALG00010019942	0.038535107	0.148656461
ENSGALG00010019943	0.583723844	0.426360387
ENSGALG00010019944	0.777918554	0.517843787
ENSGALG00010019945	0.097877284	0.350176787
ENSGALG00010019947	0.008896856	0.297805425
ENSGALG00010019948	0.042295127	0.087773508
ENSGALG00010019949	0.749038177	0.358112712
ENSGALG00010019951	0.11323327	0.255540944
ENSGALG00010019953	0.292495439	0.090784524
ENSGALG00010019954	0.730076384	0.205535857

ENSGALG00010019955	0.073980033	0.247145833
ENSGALG00010019956	0.88291556	0.62481339
ENSGALG00010019959	0.179860993	0.179713399
ENSGALG00010019961	0.863554948	0.34454017
ENSGALG00010019962	0.980906027	0.277586588
ENSGALG00010019964	0.738575175	0.258500396
ENSGALG00010019966	0.191214941	0.24424656
ENSGALG00010019967	0.847992536	0.291779858
ENSGALG00010019968	0.445697602	0.219895474
ENSGALG00010019969	0.027823108	0.310938776
ENSGALG00010019970	0.71554899	0.295272369
ENSGALG00010019972	0.987640654	0.261134664
ENSGALG00010019975	0.92657394	0.355803036
ENSGALG00010019977	0.218869449	0.316126237
ENSGALG00010019982	0.943708716	0.30864672
ENSGALG00010019983	0.371874213	0.063394792
ENSGALG00010019985	0.917889152	0.287162243
ENSGALG00010019988	0.271701261	0.072417625
ENSGALG00010019989	0.854304846	0.195113085
ENSGALG00010019990	0.941864853	0.297088682
ENSGALG00010019991	0.960258004	0.269621869
ENSGALG00010019992	0.481789544	0.752209756
ENSGALG00010019993	0.593997736	0.08654065
ENSGALG00010019994	0.836549704	0.495972991
ENSGALG00010019995	0.200459704	0.179464795
ENSGALG00010019996	0.217214742	0.068736115
ENSGALG00010019997	0.930344015	0.227954416
ENSGALG00010019998	0.346857435	0.15482493
ENSGALG00010019999	0.078982618	0.158326079
ENSGALG00010020002	0.387007219	0.32718801
ENSGALG00010020003	0.946168379	0.370926775
ENSGALG00010020005	0.704249373	0.425472857
ENSGALG00010020006	0.733263125	0.332673951
ENSGALG00010020007	0.103443347	0.338877422
ENSGALG00010020008	0.930052829	0.152616025
ENSGALG00010020009	0.938870862	0.228042781
ENSGALG00010020010	0.9472898	0.293839136
ENSGALG00010020011	0.737411417	0.250092108
ENSGALG00010020012	0.880669974	0.403399298
ENSGALG00010020013	0.856611092	0.090586719
ENSGALG00010020014	0.277794258	0.110745598
ENSGALG00010020015	0.824429256	0.258323138
ENSGALG00010020017	0.988717082	0.312915531
ENSGALG00010020018	0.417195075	0.438321435
ENSGALG00010020019	0.97568914	0.310131876
ENSGALG00010020023	0.76042559	0.12933663
ENSGALG00010020025	0.722159626	0.382979251
ENSGALG00010020026	0.873753111	0.393287802
ENSGALG00010020027	0.280056159	0.177627276
ENSGALG00010020028	0.907634415	0.196271621
ENSGALG00010020029	0.59122585	0.159725877
ENSGALG00010020030	0.925032016	0.274292183
ENSGALG00010020032	0.81138571	0.330848036
ENSGALG00010020033	0.930322987	0.451044674
ENSGALG00010020035	0.282096614	0.332024048
ENSGALG00010020036	0.212273854	0.161504247

ENSGALG00010020037	0.410403324	0.131484176
ENSGALG00010020038	0.056521171	0.029729535
ENSGALG00010020039	0.409331554	0.235659671
ENSGALG00010020041	0.84243441	0.389810993
ENSGALG00010020042	0.03592601	0.44110895
ENSGALG00010020044	0.696764186	0.460707791
ENSGALG00010020046	0.665390152	0.026661202
ENSGALG00010020048	0.478812278	0.359727071
ENSGALG00010020050	0.606668492	0.458127313
ENSGALG00010020051	0.86040396	0.541797106
ENSGALG00010020053	0.743585277	0.468882332
ENSGALG00010020055	0.784796562	0.279862298
ENSGALG00010020056	0.276668027	0.140206885
ENSGALG00010020057	0.684268013	0.231232892
ENSGALG00010020058	0.189240572	0.04996112
ENSGALG00010020059	0.859827806	0.321258183
ENSGALG00010020061	0.515577418	0.157905822
ENSGALG00010020062	0.843362421	0.119601852
ENSGALG00010020063	0.58211732	0.156626527
ENSGALG00010020064	0.307306451	0.282964964
ENSGALG00010020065	0.930229292	0.225561408
ENSGALG00010020066	0.436962567	0.059157051
ENSGALG00010020067	0.487029626	0.057204252
ENSGALG00010020069	0.172903936	0.107891578
ENSGALG00010020070	0.995510706	0.314984806
ENSGALG00010020071	0.98733594	0.31096061
ENSGALG00010020072	0.692445445	0.369979111
ENSGALG00010020073	0.541766033	0.138128296
ENSGALG00010020074	0.839909611	0.124258675
ENSGALG00010020075	0.031516128	0.030149339
ENSGALG00010020078	0.215547553	0.055090951
ENSGALG00010020080	0.604231815	0.096686645
ENSGALG00010020081	0.773894541	0.120447064
ENSGALG00010020082	0.008043983	0.110635211
ENSGALG00010020083	0.466968707	0.077433213
ENSGALG00010020084	0.767964559	0.088319862
ENSGALG00010020087	0.490888107	0.426957497
ENSGALG00010020088	0.991930919	0.341317824
ENSGALG00010020089	0.925283398	0.276388106
ENSGALG00010020090	0.850696325	0.102100208
ENSGALG00010020091	0.599310847	0.258794322
ENSGALG00010020092	0.944664945	0.318904326
ENSGALG00010020094	0.888876998	0.425499314
ENSGALG00010020095	0.478059971	0.457712796
ENSGALG00010020096	0.037331804	0.156433824
ENSGALG00010020097	0.934440206	0.267405458
ENSGALG00010020098	0.915260184	0.182779673
ENSGALG00010020100	0.517857661	0.230350709
ENSGALG00010020101	0.779872011	0.237984082
ENSGALG00010020102	0.933275149	0.377189483
ENSGALG00010020103	0.971986878	0.261087219
ENSGALG00010020104	0.448967064	0.321328006
ENSGALG00010020106	0.946548268	0.316424224
ENSGALG00010020108	0.625889071	0.08972274
ENSGALG00010020109	0.976553569	0.364608031
ENSGALG00010020111	0.907963185	0.397873884

ENSGALG00010020112	0.729084255	0.248997353
ENSGALG00010020113	0.968241435	0.419266182
ENSGALG00010020115	0.713083852	0.3197751
ENSGALG00010020116	0.9067651	0.154815883
ENSGALG00010020117	0.955508017	0.318375401
ENSGALG00010020118	0.919959502	0.297161355
ENSGALG00010020120	0.949002965	0.289222985
ENSGALG00010020121	0.071632426	0.114212428
ENSGALG00010020123	0.738833228	0.029096111
ENSGALG00010020125	0.794031035	0.279120523
ENSGALG00010020126	0.993767231	0.372178414
ENSGALG00010020127	0.884739269	0.395020995
ENSGALG00010020128	0.139920369	0.086566347
ENSGALG00010020129	0.95387847	0.280952039
ENSGALG00010020130	0.956241471	0.345353376
ENSGALG00010020131	0.388545176	0.174882184
ENSGALG00010020132	0.875807163	0.227719229
ENSGALG00010020133	0.146792371	0.169891493
ENSGALG00010020136	0.80641198	0.234511191
ENSGALG00010020137	0.547019104	0.072345498
ENSGALG00010020138	0.544172866	0.336003988
ENSGALG00010020140	0.8267718	0.282761894
ENSGALG00010020141	0.559007683	0.48396727
ENSGALG00010020142	0.99549528	0.311042995
ENSGALG00010020143	0.938191913	0.260956421
ENSGALG00010020146	0.271701261	0.072417625
ENSGALG00010020147	0.901105971	0.291447687
ENSGALG00010020149	0.916233433	0.187574695
ENSGALG00010020150	0.009328574	0.083802298
ENSGALG00010020152	0.602106283	0.000521017
ENSGALG00010020153	0.987630688	0.29522325
ENSGALG00010020154	0.262922434	0.138601464
ENSGALG00010020155	0.974479059	0.391636241
ENSGALG00010020160	0.157938721	0.144488287
ENSGALG00010020163	0.928651962	0.357797024
ENSGALG00010020164	0.988188776	0.33004743
ENSGALG00010020166	0.684288517	0.320399821
ENSGALG00010020168	0.947280091	0.362827028
ENSGALG00010020169	0.304133466	0.450346192
ENSGALG00010020170	0.715820585	0.288727454
ENSGALG00010020171	0.948432439	0.329673774
ENSGALG00010020174	0.629066859	0.22839624
ENSGALG00010020175	0.499825406	0.277899341
ENSGALG00010020176	0.845332307	0.120832948
ENSGALG00010020177	0.305704808	0.008698637
ENSGALG00010020179	0.957640255	0.407317686
ENSGALG00010020180	0.961776901	0.362091783
ENSGALG00010020181	0.992236103	0.407339702
ENSGALG00010020182	0.954007002	0.322399176
ENSGALG00010020183	0.089582073	0.120246751
ENSGALG00010020184	0.776399382	0.289639337
ENSGALG00010020186	0.942929866	0.448584857
ENSGALG00010020187	0.94822787	0.305953815
ENSGALG00010020189	0.277566769	0.107857127
ENSGALG00010020192	0.388181886	0.168275567
ENSGALG00010020193	0.049818185	0.170201035

ENSGALG00010020194	0.868449347	0.100609643
ENSGALG00010020196	0.561530937	0.030127274
ENSGALG00010020198	0.318929676	0.136196818
ENSGALG00010020199	0.579949033	0.241923084
ENSGALG00010020202	0.750408666	0.179327305
ENSGALG00010020204	0.938345656	0.294278413
ENSGALG00010020205	0.297090418	0.136289521
ENSGALG00010020206	0.960908734	0.282865422
ENSGALG00010020207	0.318121149	0.063067284
ENSGALG00010020208	0.988601461	0.395591338
ENSGALG00010020209	0.480082909	0.111927031
ENSGALG00010020210	0.465829381	0.191579387
ENSGALG00010020211	0.528613725	0.244230284
ENSGALG00010020212	0.457738473	0.089658185
ENSGALG00010020213	0.287571043	0.097865007
ENSGALG00010020215	0.9542151	0.304079168
ENSGALG00010020216	0.672989678	0.019537847
ENSGALG00010020217	0.901482736	0.329393544
ENSGALG00010020218	0.659380756	0.322205127
ENSGALG00010020220	0.752805333	0.278066716
ENSGALG00010020223	0.387070339	0.105589029
ENSGALG00010020224	0.869706154	0.37927876
ENSGALG00010020226	0.918084877	0.306161785
ENSGALG00010020228	0.985527861	0.319743506
ENSGALG00010020229	0.545112016	0.168558227
ENSGALG00010020230	0.858476851	0.361414867
ENSGALG00010020231	0.994301755	0.334224246
ENSGALG00010020234	0.066975135	0.131726206
ENSGALG00010020235	0.004142612	0.369612744
ENSGALG00010020236	0.103465014	0.394646778
ENSGALG00010020237	0.90646765	0.259634637
ENSGALG00010020240	0.004548546	0.260743011
ENSGALG00010020244	0.954184444	0.20931271
ENSGALG00010020246	0.487295094	0.151955111
ENSGALG00010020247	0.489688642	0.142768469
ENSGALG00010020248	0.93608277	0.280383141
ENSGALG00010020250	0.877862036	0.090254142
ENSGALG00010020251	0.205392353	0.048267044
ENSGALG00010020252	0.176948081	0.188039463
ENSGALG00010020254	0.697141454	0.112228752
ENSGALG00010020257	0.968699266	0.29644739
ENSGALG00010020258	0.410185387	0.15690962
ENSGALG00010020259	0.758369745	0.031741635
ENSGALG00010020260	0.303793964	0.471654236
ENSGALG00010020261	0.90503726	0.335493714
ENSGALG00010020262	0.18454997	0.160977104
ENSGALG00010020265	0.444366257	0.233031149
ENSGALG00010020266	0.591683149	0.438908213
ENSGALG00010020267	0.799651782	0.437243871
ENSGALG00010020268	0.916961219	0.252392455
ENSGALG00010020269	0.261653625	0.225901408
ENSGALG00010020270	0.224020725	0.161177186
ENSGALG00010020272	0.660027856	0.152336487
ENSGALG00010020273	0.200570274	0.091667991
ENSGALG00010020274	0.382985366	0.1291087
ENSGALG00010020275	0.29212014	0.404766674

ENSGALG00010020276	0.136512308	0.205061022
ENSGALG00010020277	0.525778537	0.164762089
ENSGALG00010020278	0.189240572	0.04996112
ENSGALG00010020279	0.401639624	0.101384476
ENSGALG00010020282	0.979260861	0.319686205
ENSGALG00010020283	0.946651791	0.298804409
ENSGALG00010020284	0.349093479	0.169858773
ENSGALG00010020285	0.499120598	0.069785254
ENSGALG00010020286	0.396211517	0.126961164
ENSGALG00010020287	0.765461697	0.441941099
ENSGALG00010020290	0.319320077	0.024051854
ENSGALG00010020291	0.958347801	0.329512035
ENSGALG00010020292	0.434860708	0.100756569
ENSGALG00010020293	0.825543496	0.176392723
ENSGALG00010020294	0.116057914	0.149502427
ENSGALG00010020295	0.179043696	0.046726239
ENSGALG00010020297	0.360444497	0.098868233
ENSGALG00010020298	0.939665823	0.279643978
ENSGALG00010020300	0.221485348	0.180571296
ENSGALG00010020302	0.996213389	0.336265371
ENSGALG00010020303	0.706511058	0.41967498
ENSGALG00010020304	0.894974301	0.264374075
ENSGALG00010020305	0.608825599	0.531264444
ENSGALG00010020306	0.822688596	0.287483828
ENSGALG00010020307	0.073583463	0.3709226
ENSGALG00010020309	0.316919054	0.259055221
ENSGALG00010020311	0.9744157	0.269011357
ENSGALG00010020313	0.10226259	0.270664646
ENSGALG00010020314	0.7036622	0.033899401
ENSGALG00010020315	0.958914663	0.354854638
ENSGALG00010020316	0.384186114	0.249334314
ENSGALG00010020319	0.937470555	0.391516107
ENSGALG00010020320	0.573834299	0.092471704
ENSGALG00010020321	0.867625476	0.343458316
ENSGALG00010020322	0.044640162	0.167754086
ENSGALG00010020323	0.934315017	0.331287994
ENSGALG00010020324	0.817341214	0.230711573
ENSGALG00010020325	0.2393273	0.222490344
ENSGALG00010020326	0.24614705	0.19254487
ENSGALG00010020327	0.996503057	0.351952451
ENSGALG00010020328	0.290616396	0.103443064
ENSGALG00010020329	0.292482362	0.359423709
ENSGALG00010020331	0.832467751	0.030715327
ENSGALG00010020334	0.875752366	0.28930357
ENSGALG00010020336	0.88603643	0.325488185
ENSGALG00010020337	0.387151135	0.264371195
ENSGALG00010020338	0.968989051	0.248445009
ENSGALG00010020339	0.158147761	0.13255959
ENSGALG00010020340	0.680347823	0.289989621
ENSGALG00010020341	0.899559999	0.141917287
ENSGALG00010020342	0.957718898	0.359455036
ENSGALG00010020343	0.952645749	0.284015513
ENSGALG00010020344	0.63684563	0.028973585
ENSGALG00010020345	0.919519258	0.373991505
ENSGALG00010020346	0.982658457	0.30592838
ENSGALG00010020347	0.984551353	0.406962003

ENSGALG00010020350	0.989171446	0.336323288
ENSGALG00010020351	0.677910474	0.498094026
ENSGALG00010020352	0.974664507	0.292707412
ENSGALG00010020353	0.782635951	0.071656968
ENSGALG00010020354	0.818723244	0.018282466
ENSGALG00010020356	0.946457071	0.318527052
ENSGALG00010020357	0.993534897	0.305633561
ENSGALG00010020359	0.503707307	0.365635161
ENSGALG00010020360	0.97138188	0.32579612
ENSGALG00010020361	0.400253218	0.132142018
ENSGALG00010020362	0.948750567	0.252604044
ENSGALG00010020364	0.973666524	0.313810356
ENSGALG00010020365	0.711248135	0.136276183
ENSGALG00010020368	0.994554293	0.344207214
ENSGALG00010020369	0.788957252	0.184275096
ENSGALG00010020370	0.711240541	0.098563187
ENSGALG00010020371	0.31547063	0.309810246
ENSGALG00010020372	0.012184093	0.540210871
ENSGALG00010020373	0.98488434	0.286161005
ENSGALG00010020374	0.795636289	0.152574441
ENSGALG00010020375	0.450315715	0.108469459
ENSGALG00010020377	0.791099214	0.14412233
ENSGALG00010020379	0.946860467	0.264508966
ENSGALG00010020380	0.262922434	0.138601464
ENSGALG00010020381	0.674539653	0.190915282
ENSGALG00010020382	0.882534286	0.376081999
ENSGALG00010020383	0.857957252	0.231391894
ENSGALG00010020384	0.689912718	0.07053755
ENSGALG00010020385	0.951031534	0.368970981
ENSGALG00010020386	0.8100781	0.176608129
ENSGALG00010020388	0.935559933	0.391108068
ENSGALG00010020389	0.938217365	0.235902397
ENSGALG00010020390	0.473312807	0.314760548
ENSGALG00010020391	0.253372503	0.032327539
ENSGALG00010020392	0.951736191	0.491713758
ENSGALG00010020393	0.256019099	0.216834183
ENSGALG00010020396	0.707291406	0.438007498
ENSGALG00010020397	0.200570274	0.091667991
ENSGALG00010020399	0.920779815	0.32458969
ENSGALG00010020400	0.449842243	0.880367635
ENSGALG00010020402	0.939354813	0.342820024
ENSGALG00010020405	0.579190936	0.058075035
ENSGALG00010020407	0.075248743	0.102913545
ENSGALG00010020408	0.495916114	0.179082398
ENSGALG00010020410	0.203309254	0.296023659
ENSGALG00010020411	0.055172705	0.149124272
ENSGALG00010020412	0.587058424	0.051759982
ENSGALG00010020413	0.509235349	0.036591239
ENSGALG00010020414	0.255766848	0.224447046
ENSGALG00010020415	0.730206753	0.169915086
ENSGALG00010020417	0.887924591	0.403302773
ENSGALG00010020418	0.349432482	0.011755753
ENSGALG00010020419	0.383103953	0.01627307
ENSGALG00010020420	0.891676166	0.372534867
ENSGALG00010020421	0.494148056	0.064369836
ENSGALG00010020422	0.24254036	0.086944743

ENSGALG00010020427	0.41296042	0.806690559
ENSGALG00010020428	0.400184835	0.084575054
ENSGALG00010020429	0.71876846	0.39042322
ENSGALG00010020430	0.420431013	0.707899194
ENSGALG00010020432	0.386375828	0.172117916
ENSGALG00010020433	0.681693441	0.346836297
ENSGALG00010020434	0.615740126	0.2849496
ENSGALG00010020436	0.761128607	0.063695792
ENSGALG00010020437	0.707864496	0.28829985
ENSGALG00010020438	0.919103171	0.264253744
ENSGALG00010020439	0.277566769	0.107857127
ENSGALG00010020440	0.978050674	0.350687461
ENSGALG00010020441	0.743938086	0.420616735
ENSGALG00010020442	0.682971434	0.327743641
ENSGALG00010020443	0.422106722	0.136384099
ENSGALG00010020445	0.873063824	0.354453937
ENSGALG00010020446	0.121099407	0.267700667
ENSGALG00010020447	0.45948101	0.229067773
ENSGALG00010020448	0.121055082	0.149905879
ENSGALG00010020449	0.928704229	0.3648326
ENSGALG00010020450	0.871059902	0.263540938
ENSGALG00010020451	0.090602943	0.026032714
ENSGALG00010020452	0.721740863	0.289362654
ENSGALG00010020453	0.81908753	0.487437521
ENSGALG00010020454	0.862858465	0.483983261
ENSGALG00010020455	0.956626403	0.323724769
ENSGALG00010020457	0.488782136	0.091882936
ENSGALG00010020459	0.652430022	0.199761189
ENSGALG00010020460	0.462075186	0.277406906
ENSGALG00010020461	0.78587489	0.25121653
ENSGALG00010020462	0.316983401	0.11751522
ENSGALG00010020463	0.187514554	0.108479243
ENSGALG00010020465	0.917161163	0.328750242
ENSGALG00010020467	0.068177567	0.142182378
ENSGALG00010020468	0.901709005	0.301534187
ENSGALG00010020469	0.886813364	0.319630359
ENSGALG00010020470	0.337009149	0.19622042
ENSGALG00010020472	0.478700858	0.154815381
ENSGALG00010020473	0.963221253	0.311346287
ENSGALG00010020474	0.324368703	0.149815344
ENSGALG00010020475	0.114287061	0.131886953
ENSGALG00010020476	0.724611528	0.23610225
ENSGALG00010020478	0.940320863	0.338775799
ENSGALG00010020479	0.369780764	0.1127516
ENSGALG00010020480	0.201101128	0.166332235
ENSGALG00010020481	0.897132241	0.342851435
ENSGALG00010020482	0.208550923	0.676301294
ENSGALG00010020484	0.905086219	0.166792425
ENSGALG00010020485	0.726186731	0.471397572
ENSGALG00010020486	0.050645232	0.152289163
ENSGALG00010020488	0.208550923	0.676301294
ENSGALG00010020489	0.455898289	0.021567847
ENSGALG00010020491	0.609951877	0.287986821
ENSGALG00010020493	0.942582385	0.351532723
ENSGALG00010020497	0.972798341	0.308215589
ENSGALG00010020498	0.912180971	0.319596426

ENSGALG00010020500	0.064519051	0.143619078
ENSGALG00010020501	0.476493638	0.163642365
ENSGALG00010020507	0.963914694	0.339621005
ENSGALG00010020509	0.764781875	0.093175426
ENSGALG00010020510	0.417145881	0.03944592
ENSGALG00010020513	0.509290079	0.261262817
ENSGALG00010020514	0.26536627	0.210350919
ENSGALG00010020516	0.839478048	0.317581957
ENSGALG00010020517	0.808244003	0.303456432
ENSGALG00010020519	0.341637029	0.127743113
ENSGALG00010020520	0.853064942	0.459256374
ENSGALG00010020521	0.888122685	0.377377191
ENSGALG00010020522	0.922058643	0.31168468
ENSGALG00010020524	0.325349187	0.290512841
ENSGALG00010020525	0.991377857	0.33024993
ENSGALG00010020526	0.489827757	0.225686727
ENSGALG00010020530	0.952047621	0.385926679
ENSGALG00010020533	0.866168448	0.298942091
ENSGALG00010020534	0.514392207	0.178555385
ENSGALG00010020535	0.92687953	0.328825069
ENSGALG00010020537	0.710435459	0.344800277
ENSGALG00010020538	0.757140355	0.235576216
ENSGALG00010020539	0.383913097	0.180221586
ENSGALG00010020540	0.751088457	0.147475828
ENSGALG00010020542	0.501827259	0.171775072
ENSGALG00010020544	0.976743382	0.362900393
ENSGALG00010020545	0.953231448	0.301430741
ENSGALG00010020546	0.980910205	0.322605762
ENSGALG00010020547	0.285272007	0.109169955
ENSGALG00010020548	0.874476467	0.358901663
ENSGALG00010020550	0.754658998	0.189305589
ENSGALG00010020554	0.671275217	0.162691826
ENSGALG00010020556	0.778256928	0.118953508
ENSGALG00010020557	0.963236059	0.328272996
ENSGALG00010020558	0.985405919	0.267712358
ENSGALG00010020559	0.767056618	0.270121799
ENSGALG00010020560	0.314520251	0.081504091
ENSGALG00010020561	0.778581377	0.347910667
ENSGALG00010020562	0.900728202	0.177059341
ENSGALG00010020563	0.410235727	0.491862739
ENSGALG00010020564	0.817797576	0.37153093
ENSGALG00010020565	0.682530937	0.170765948
ENSGALG00010020568	0.944117345	0.357497814
ENSGALG00010020569	0.969122506	0.323595003
ENSGALG00010020570	0.060268175	0.168077398
ENSGALG00010020571	0.981251775	0.260015671
ENSGALG00010020572	0.795798891	0.335985589
ENSGALG00010020573	0.592330361	0.217084871
ENSGALG00010020574	0.572547474	0.507567
ENSGALG00010020575	0.748818935	0.413120295
ENSGALG00010020576	0.988599929	0.312339366
ENSGALG00010020577	0.004887549	0.04546436
ENSGALG00010020578	0.742411644	0.290116055
ENSGALG00010020579	0.421600795	0.168736327
ENSGALG00010020580	0.38511333	0.256177352
ENSGALG00010020581	0.745999508	0.23900349

ENSGALG00010020582	0.983775112	0.315178108
ENSGALG00010020583	0.678445138	0.24356165
ENSGALG00010020584	0.92602171	0.376811096
ENSGALG00010020585	0.290122505	0.464993379
ENSGALG00010020586	0.269503727	0.383637442
ENSGALG00010020587	0.603056842	0.268516678
ENSGALG00010020588	0.976247421	0.382201372
ENSGALG00010020590	0.86821197	0.127117406
ENSGALG00010020591	0.70482047	0.327324576
ENSGALG00010020592	0.914473359	0.307967632
ENSGALG00010020593	0.690119443	0.336310208
ENSGALG00010020594	0.905089303	0.273401166
ENSGALG00010020595	0.57493456	0.060471239
ENSGALG00010020596	0.308097187	0.310428988
ENSGALG00010020597	0.408114531	0.113315477
ENSGALG00010020598	0.481124533	0.17287833
ENSGALG00010020599	0.960248444	0.263144592
ENSGALG00010020600	0.968858557	0.362270085
ENSGALG00010020601	0.375699607	0.182609349
ENSGALG00010020602	0.978818149	0.348975324
ENSGALG00010020603	0.039735217	0.00817848
ENSGALG00010020604	0.958809139	0.381503866
ENSGALG00010020605	0.874257857	0.324358444
ENSGALG00010020606	0.270451009	0.090541667
ENSGALG00010020607	0.777509399	0.126196597
ENSGALG00010020608	0.991669579	0.281747639
ENSGALG00010020609	0.020659512	0.201787801
ENSGALG00010020610	0.375927362	0.072713138
ENSGALG00010020611	0.980985627	0.335134889
ENSGALG00010020612	0.851811717	0.28780543
ENSGALG00010020613	0.213204188	0.253729922
ENSGALG00010020614	0.922600038	0.345002588
ENSGALG00010020615	0.892948425	0.298715329
ENSGALG00010020616	0.792927161	0.174608427
ENSGALG00010020617	0.553887071	0.144149795
ENSGALG00010020618	0.956553601	0.18648785
ENSGALG00010020619	0.95413562	0.236892657
ENSGALG00010020620	0.099841857	0.210681669
ENSGALG00010020621	0.815047886	0.286409037
ENSGALG00010020622	0.968154354	0.309313587
ENSGALG00010020623	0.546846383	0.319762739
ENSGALG00010020624	0.96786482	0.322454164
ENSGALG00010020625	0.992231475	0.297038744
ENSGALG00010020626	0.55117692	0.529721626
ENSGALG00010020628	0.116087723	0.034760228
ENSGALG00010020629	0.184177001	0.381409633
ENSGALG00010020630	0.914467364	0.373977361
ENSGALG00010020632	0.917017626	0.126661379
ENSGALG00010020633	0.155915113	0.433989127
ENSGALG00010020634	0.123711406	0.412306205
ENSGALG00010020635	0.894961069	0.312459291
ENSGALG00010020636	0.951136639	0.386214095
ENSGALG00010020637	0.322358098	0.049764011
ENSGALG00010020638	0.986591013	0.344884596
ENSGALG00010020640	0.746659661	0.243607547
ENSGALG00010020641	0.390688024	0.154376612

ENSGALG00010020642	0.718248245	0.306968442
ENSGALG00010020643	0.044955329	0.304961216
ENSGALG00010020644	0.200570274	0.091667991
ENSGALG00010020645	0.3370502	0.133259151
ENSGALG00010020646	0.776706201	0.263331159
ENSGALG00010020647	0.638671809	0.648426078
ENSGALG00010020648	0.858100571	0.352133078
ENSGALG00010020649	0.961045661	0.231735211
ENSGALG00010020650	0.886587911	0.265219615
ENSGALG00010020651	0.76246313	0.014323299
ENSGALG00010020652	0.636058693	0.466203476
ENSGALG00010020653	0.940296383	0.297408915
ENSGALG00010020654	0.338453547	0.383259594
ENSGALG00010020655	0.481663103	0.0475525
ENSGALG00010020657	0.883257637	0.172358994
ENSGALG00010020658	0.887708926	0.346018357
ENSGALG00010020659	0.886096834	0.442405396
ENSGALG00010020660	0.595486125	0.195240614
ENSGALG00010020662	0.923919423	0.292125295
ENSGALG00010020663	0.252637566	0.305424595
ENSGALG00010020664	0.555622921	0.171124523
ENSGALG00010020665	0.582687075	0.199697713
ENSGALG00010020666	0.691288674	0.189763308
ENSGALG00010020667	0.596531208	0.092646998
ENSGALG00010020668	0.466317853	0.337985432
ENSGALG00010020669	0.736224633	0.320743243
ENSGALG00010020670	0.357803227	0.160665132
ENSGALG00010020671	0.281348506	0.111358441
ENSGALG00010020672	0.694063467	0.070495835
ENSGALG00010020673	0.469603526	0.211873123
ENSGALG00010020675	0.968110656	0.373559859
ENSGALG00010020676	0.991237741	0.349262765
ENSGALG00010020677	0.3112811	0.088460727
ENSGALG00010020678	0.552279388	0.224606277
ENSGALG00010020679	0.864511251	0.247631834
ENSGALG00010020680	0.488638928	0.284647358
ENSGALG00010020681	0.784707411	0.350995972
ENSGALG00010020682	0.180808184	0.323375394
ENSGALG00010020684	0.215007606	0.046290421
ENSGALG00010020685	0.884414921	0.279184649
ENSGALG00010020686	0.787869223	0.240500272
ENSGALG00010020689	0.341484671	0.405588297
ENSGALG00010020690	0.193228965	0.297822659
ENSGALG00010020691	0.962226561	0.329266192
ENSGALG00010020692	0.846477431	0.440220051
ENSGALG00010020693	0.051306891	0.024134752
ENSGALG00010020694	0.586621357	0.055892992
ENSGALG00010020695	0.268465557	0.010121625
ENSGALG00010020696	0.890725633	0.271982527
ENSGALG00010020697	0.278765262	0.561539442
ENSGALG00010020698	0.616891431	0.16351448
ENSGALG00010020699	0.930513288	0.277049639
ENSGALG00010020700	0.967185337	0.29712803
ENSGALG00010020701	0.943056699	0.36198063
ENSGALG00010020702	0.185500048	0.211168809
ENSGALG00010020704	0.982100966	0.32946532

ENSGALG00010020705	0.974791316	0.333409929
ENSGALG00010020706	0.909822225	0.486994214
ENSGALG00010020707	0.967072377	0.285795557
ENSGALG00010020708	0.247410978	0.125394905
ENSGALG00010020709	0.893906014	0.312636614
ENSGALG00010020711	0.948157883	0.343590262
ENSGALG00010020712	0.941889272	0.366685719
ENSGALG00010020713	0.941536463	0.337071032
ENSGALG00010020714	0.996503184	0.35503628
ENSGALG00010020715	0.894264602	0.448888572
ENSGALG00010020716	0.935103864	0.270535501
ENSGALG00010020717	0.459285249	0.136630132
ENSGALG00010020718	0.792043998	0.340400786
ENSGALG00010020719	0.778842421	0.322684913
ENSGALG00010020720	0.974496187	0.378066573
ENSGALG00010020721	0.410899696	0.452255136
ENSGALG00010020723	0.920108523	0.308010623
ENSGALG00010020724	0.43009708	0.077028092
ENSGALG00010020725	0.618466543	0.176924127
ENSGALG00010020726	0.541780325	0.192305211
ENSGALG00010020727	0.984211736	0.295950318
ENSGALG00010020728	0.863449701	0.340198219
ENSGALG00010020729	0.327392726	0.342589916
ENSGALG00010020730	0.995729976	0.359316109
ENSGALG00010020731	0.559301295	0.274132423
ENSGALG00010020732	0.985526912	0.323502023
ENSGALG00010020733	0.990932981	0.39161896
ENSGALG00010020734	0.995857443	0.336232846
ENSGALG00010020735	0.871052727	0.343644104
ENSGALG00010020736	0.487292843	0.092080489
ENSGALG00010020737	0.598876456	0.131356243
ENSGALG00010020738	0.306269874	0.488490028
ENSGALG00010020739	0.879045607	0.298849044
ENSGALG00010020741	0.941974405	0.389983449
ENSGALG00010020742	0.196511741	0.117092107
ENSGALG00010020743	0.857413243	0.369702998
ENSGALG00010020744	0.600821133	0.228499379
ENSGALG00010020745	0.887355398	0.218267221
ENSGALG00010020746	0.582131949	0.168287701
ENSGALG00010020747	0.915334277	0.293170466
ENSGALG00010020748	0.830312444	0.275370539
ENSGALG00010020749	0.154167952	0.472083635
ENSGALG00010020750	0.399427869	0.208545082
ENSGALG00010020752	0.624685222	0.033647005
ENSGALG00010020753	0.829815168	0.207128509
ENSGALG00010020754	0.946000873	0.426279773
ENSGALG00010020755	0.754024641	0.162560877
ENSGALG00010020756	0.271626977	0.127748201
ENSGALG00010020758	0.294909804	0.0629881
ENSGALG00010020759	0.21496372	0.036745329
ENSGALG00010020760	0.957149002	0.345848081
ENSGALG00010020762	0.547710349	0.16109788
ENSGALG00010020763	0.468132971	0.278535379
ENSGALG00010020764	0.982921788	0.282694651
ENSGALG00010020765	0.956028114	0.349358418
ENSGALG00010020766	0.797305075	0.341124992

ENSGALG00010020767	0.968886756	0.298083183
ENSGALG00010020769	0.407281254	0.337026701
ENSGALG00010020770	0.769041059	0.305033639
ENSGALG00010020771	0.988681991	0.329909162
ENSGALG00010020772	0.044800328	0.15752141
ENSGALG00010020773	0.978671904	0.344461834
ENSGALG00010020774	0.002808211	0.318497366
ENSGALG00010020775	0.814281533	0.259232971
ENSGALG00010020776	0.741794208	0.247639585
ENSGALG00010020777	0.445286975	0.158165284
ENSGALG00010020778	0.881277495	0.294482977
ENSGALG00010020779	0.434601125	0.383146991
ENSGALG00010020780	0.237246509	0.183928226
ENSGALG00010020781	0.95704372	0.393869253
ENSGALG00010020782	0.973510224	0.342797266
ENSGALG00010020783	0.398964639	0.068333442
ENSGALG00010020784	0.483803411	0.002859434
ENSGALG00010020786	0.917066274	0.268081021
ENSGALG00010020787	0.686944969	0.476037482
ENSGALG00010020789	0.019377489	0.195136276
ENSGALG00010020790	0.885542203	0.358951499
ENSGALG00010020791	0.397516991	0.125566479
ENSGALG00010020792	0.571395579	0.036659766
ENSGALG00010020793	0.127328431	0.511200971
ENSGALG00010020794	0.975721881	0.387913674
ENSGALG00010020795	0.331669341	0.196143555
ENSGALG00010020796	0.504401928	0.30445242
ENSGALG00010020797	0.86175939	0.161965103
ENSGALG00010020798	0.611262508	0.005356014
ENSGALG00010020799	0.296984403	0.336871547
ENSGALG00010020800	0.372757682	0.112441993
ENSGALG00010020802	0.186136844	0.268763825
ENSGALG00010020803	0.986048145	0.358063292
ENSGALG00010020804	0.945034336	0.298668343
ENSGALG00010020806	0.793400146	0.186192634
ENSGALG00010020808	0.238485933	0.201823685
ENSGALG00010020809	0.993868773	0.330467937
ENSGALG00010020810	0.93692646	0.296275723
ENSGALG00010020811	0.993924841	0.267399461
ENSGALG00010020813	0.119508885	0.430755897
ENSGALG00010020816	0.883163545	0.29800357
ENSGALG00010020818	0.905601522	0.086200595
ENSGALG00010020819	0.370951809	0.117490575
ENSGALG00010020820	0.714101558	0.053407554
ENSGALG00010020822	0.745017789	0.198556494
ENSGALG00010020824	0.597126524	0.095740434
ENSGALG00010020825	0.915185197	0.293723345
ENSGALG00010020826	0.893103763	0.306692709
ENSGALG00010020827	0.952905769	0.22571159
ENSGALG00010020828	0.358340038	0.218603587
ENSGALG00010020829	0.92792625	0.26190552
ENSGALG00010020830	0.965698896	0.289314749
ENSGALG00010020831	0.932685071	0.300801763
ENSGALG00010020832	0.407452051	0.243070492
ENSGALG00010020833	0.7905035	0.321560206
ENSGALG00010020835	0.898555596	0.381041046

ENSGALG00010020838	0.850442079	0.307333819
ENSGALG00010020839	0.8504118	0.26362143
ENSGALG00010020840	0.945602006	0.320729502
ENSGALG00010020842	0.262922434	0.138601464
ENSGALG00010020843	0.937044668	0.49878054
ENSGALG00010020844	0.156767252	0.165521014
ENSGALG00010020845	0.146524323	0.151387256
ENSGALG00010020846	0.491540463	0.126862234
ENSGALG00010020847	0.636854656	0.197528793
ENSGALG00010020848	0.225836681	0.217082097
ENSGALG00010020850	0.161330889	0.128849645
ENSGALG00010020852	0.270451009	0.090541667
ENSGALG00010020854	0.988669325	0.32502654
ENSGALG00010020855	0.844741431	0.428334638
ENSGALG00010020860	0.351096783	0.165551313
ENSGALG00010020861	0.963250979	0.332821937
ENSGALG00010020862	0.266521434	0.312856314
ENSGALG00010020863	0.227888411	0.173427787
ENSGALG00010020864	0.358813744	0.197864007
ENSGALG00010020865	0.565625586	0.33598432
ENSGALG00010020866	0.753331692	0.241184258
ENSGALG00010020867	0.224008667	0.143568574
ENSGALG00010020868	0.060562387	0.06174965
ENSGALG00010020869	0.396668506	0.181671381
ENSGALG00010020870	0.962495104	0.390638316
ENSGALG00010020871	0.693818916	0.210155422
ENSGALG00010020872	0.368720526	0.277125447
ENSGALG00010020873	0.980047239	0.280417526
ENSGALG00010020875	0.988870374	0.319309522
ENSGALG00010020877	0.814139662	0.181176778
ENSGALG00010020878	0.646360667	0.123885799
ENSGALG00010020879	0.869858387	0.277400699
ENSGALG00010020880	0.113642075	0.088679342
ENSGALG00010020882	0.033024477	0.241212674
ENSGALG00010020883	0.342798517	0.150343339
ENSGALG00010020885	0.323046176	0.318166017
ENSGALG00010020886	0.359175204	0.300173877
ENSGALG00010020887	0.382370486	0.096472474
ENSGALG00010020889	0.82731135	0.200105974
ENSGALG00010020890	0.743518262	0.253551097
ENSGALG00010020893	0.366356945	0.080496265
ENSGALG00010020894	0.916290833	0.339769199
ENSGALG00010020895	0.701540713	0.528393939
ENSGALG00010020896	0.967485767	0.232461823
ENSGALG00010020897	0.41296042	0.806690559
ENSGALG00010020898	0.761189854	0.125509903
ENSGALG00010020899	0.266137114	0.107784983
ENSGALG00010020900	0.673523001	0.146493161
ENSGALG00010020901	0.090108996	0.186196746
ENSGALG00010020902	0.235505015	0.175050375
ENSGALG00010020903	0.965543958	0.269561909
ENSGALG00010020904	0.289607518	0.919337459
ENSGALG00010020905	0.889074716	0.25085926
ENSGALG00010020906	0.752091177	0.252366577
ENSGALG00010020908	0.903411798	0.263279578
ENSGALG00010020909	0.400215036	0.132215663

ENSGALG00010020912	0.42166784	0.088901208
ENSGALG00010020913	0.774835182	0.010596332
ENSGALG00010020915	0.072999106	0.13664561
ENSGALG00010020916	0.961456248	0.257140988
ENSGALG00010020917	0.283513089	0.60631955
ENSGALG00010020918	0.598561102	0.016828313
ENSGALG00010020920	0.884661618	0.137527911
ENSGALG00010020921	0.031164765	0.03301008
ENSGALG00010020922	0.988180665	0.359186876
ENSGALG00010020923	0.003611467	0.115041207
ENSGALG00010020924	0.85675648	0.438769316
ENSGALG00010020925	0.974684492	0.358500821
ENSGALG00010020926	0.031576253	0.123183528
ENSGALG00010020927	0.110539129	0.188098904
ENSGALG00010020928	0.558778746	0.133983178
ENSGALG00010020929	0.098051802	0.175743111
ENSGALG00010020932	0.971065742	0.280632118
ENSGALG00010020933	0.989693103	0.304890737
ENSGALG00010020934	0.985324088	0.34981691
ENSGALG00010020935	0.949267271	0.267976972
ENSGALG00010020936	0.870648891	0.192278104
ENSGALG00010020937	0.967212817	0.189181241
ENSGALG00010020938	0.330508564	0.019587901
ENSGALG00010020940	0.739006674	0.483107047
ENSGALG00010020941	0.954632934	0.379112871
ENSGALG00010020943	0.966694214	0.378098982
ENSGALG00010020944	0.976960125	0.271123346
ENSGALG00010020945	0.980658749	0.327218125
ENSGALG00010020946	0.947773051	0.254808446
ENSGALG00010020947	0.802096747	0.159296138
ENSGALG00010020948	0.311289807	0.13851383
ENSGALG00010020949	0.519909436	0.297684865
ENSGALG00010020950	0.384309334	0.121080256
ENSGALG00010020951	0.689811566	0.359726938
ENSGALG00010020952	0.968858164	0.366422786
ENSGALG00010020953	0.240091936	0.158881448
ENSGALG00010020954	0.792065607	0.260342909
ENSGALG00010020955	0.742383644	0.326145834
ENSGALG00010020956	0.15137346	0.072214025
ENSGALG00010020957	0.719198105	0.300187993
ENSGALG00010020958	0.935409825	0.323010417
ENSGALG00010020959	0.717076954	0.466373821
ENSGALG00010020960	0.919724948	0.119790263
ENSGALG00010020961	0.210120672	0.383689609
ENSGALG00010020962	0.845599687	0.464827035
ENSGALG00010020963	0.760371254	0.139104511
ENSGALG00010020965	0.746348502	0.152959554
ENSGALG00010020966	0.9574516	0.294893142
ENSGALG00010020967	0.744780435	0.49751561
ENSGALG00010020968	0.339444113	0.045741665
ENSGALG00010020969	0.951415482	0.361574336
ENSGALG00010020970	0.952658003	0.329020975
ENSGALG00010020971	0.985728336	0.270035797
ENSGALG00010020972	0.089565001	0.154445367
ENSGALG00010020973	0.443536048	0.009782871
ENSGALG00010020974	0.271270171	0.107578172

ENSGALG00010020975	0.881476277	0.321961424
ENSGALG00010020976	0.143162467	0.192206689
ENSGALG00010020977	0.065713322	0.261555779
ENSGALG00010020978	0.905771409	0.291010675
ENSGALG00010020979	0.537515473	0.05670132
ENSGALG00010020980	0.342463317	0.142361432
ENSGALG00010020981	0.246404875	0.138114697
ENSGALG00010020982	0.612150895	0.280045213
ENSGALG00010020983	0.043904895	0.157504155
ENSGALG00010020984	0.981201042	0.281620924
ENSGALG00010020985	0.082866238	0.086007567
ENSGALG00010020986	0.776543276	0.093734882
ENSGALG00010020987	0.912795484	0.35449441
ENSGALG00010020988	0.880125963	0.103992011
ENSGALG00010020989	0.913449815	0.276388003
ENSGALG00010020990	0.583496902	0.427948511
ENSGALG00010020991	0.621378995	0.156899063
ENSGALG00010020992	0.976042187	0.35008758
ENSGALG00010020993	0.889241051	0.302312688
ENSGALG00010020994	0.954509115	0.249098979
ENSGALG00010020995	0.880315373	0.246531003
ENSGALG00010020996	0.262734614	0.270303331
ENSGALG00010020997	0.836252066	0.264299586
ENSGALG00010020998	0.846853536	0.336328229
ENSGALG00010020999	0.901447205	0.310994776
ENSGALG00010021000	0.923364422	0.304056067
ENSGALG00010021001	0.684949656	0.293027331
ENSGALG00010021002	0.870396448	0.324648288
ENSGALG00010021003	0.738230749	0.183049818
ENSGALG00010021004	0.973918422	0.368067701
ENSGALG00010021010	0.695115318	0.023156158
ENSGALG00010021011	0.934268106	0.284947495
ENSGALG00010021012	0.671052755	0.180134279
ENSGALG00010021013	0.444457376	0.108784589
ENSGALG00010021014	0.967159102	0.237446278
ENSGALG00010021015	0.044890037	0.24423872
ENSGALG00010021016	0.562211186	0.049345714
ENSGALG00010021017	0.865839912	0.459478281
ENSGALG00010021018	0.372732431	0.375576766
ENSGALG00010021019	0.394438458	0.320085186
ENSGALG00010021020	0.929300294	0.195874688
ENSGALG00010021021	0.603391953	0.447440598
ENSGALG00010021022	0.691714303	0.046575166
ENSGALG00010021023	0.865194791	0.189914265
ENSGALG00010021024	0.213376706	0.153329428
ENSGALG00010021025	0.986055326	0.27399203
ENSGALG00010021026	0.857087804	0.310267784
ENSGALG00010021027	0.944819842	0.375290751
ENSGALG00010021028	0.460937806	0.038984849
ENSGALG00010021029	0.891962305	0.311578479
ENSGALG00010021030	0.866389668	0.279355678
ENSGALG00010021031	0.707763816	0.042798516
ENSGALG00010021032	0.895341292	0.357277409
ENSGALG00010021033	0.91454956	0.403860376
ENSGALG00010021034	0.93040332	0.456733353
ENSGALG00010021036	0.798485683	0.169437539

ENSGALG00010021038	0.951025974	0.294576979
ENSGALG00010021039	0.368811714	0.142697385
ENSGALG00010021041	0.864182722	0.353206296
ENSGALG00010021042	0.978110709	0.377535071
ENSGALG00010021043	0.972158279	0.251593261
ENSGALG00010021044	0.946217749	0.212780722
ENSGALG00010021045	0.952301578	0.262233082
ENSGALG00010021046	0.572069921	0.205464264
ENSGALG00010021047	0.958758904	0.314346913
ENSGALG00010021048	0.587308794	0.028998449
ENSGALG00010021050	0.770096089	0.41070108
ENSGALG00010021051	0.470001907	0.171834962
ENSGALG00010021052	0.824919234	0.341171504
ENSGALG00010021054	0.157484768	0.120027785
ENSGALG00010021055	0.139629072	0.295467283
ENSGALG00010021056	0.262922434	0.138601464
ENSGALG00010021057	0.032202385	0.105848779
ENSGALG00010021058	0.085780402	0.073999969
ENSGALG00010021060	0.111315923	0.319375103
ENSGALG00010021062	0.887801125	0.23494162
ENSGALG00010021063	0.294277215	0.008754743
ENSGALG00010021064	0.985075675	0.312171736
ENSGALG00010021065	0.928749052	0.305348297
ENSGALG00010021066	0.27096321	0.478281169
ENSGALG00010021067	0.938057831	0.374628257
ENSGALG00010021068	0.367319118	0.191398843
ENSGALG00010021069	0.902705132	0.42388217
ENSGALG00010021070	0.643337466	0.039973004
ENSGALG00010021071	0.987364922	0.324911797
ENSGALG00010021072	0.911867812	0.181929108
ENSGALG00010021073	0.864420874	0.365777594
ENSGALG00010021074	0.921538502	0.464807482
ENSGALG00010021075	0.979464548	0.334283524
ENSGALG00010021076	0.890078601	0.298986022
ENSGALG00010021077	0.938351632	0.20936594
ENSGALG00010021078	0.817662606	0.348474609
ENSGALG00010021079	0.770096248	0.084913514
ENSGALG00010021080	0.974452027	0.307470407
ENSGALG00010021081	0.952878307	0.500723052
ENSGALG00010021082	0.986705752	0.329925233
ENSGALG00010021083	0.89485466	0.218991046
ENSGALG00010021085	0.972308323	0.304689379
ENSGALG00010021086	0.810465386	0.284430951
ENSGALG00010021087	0.88220969	0.278241204
ENSGALG00010021088	0.694069233	0.253675222
ENSGALG00010021089	0.964279764	0.286313837
ENSGALG00010021090	0.497172482	0.047761259
ENSGALG00010021091	0.16854104	0.128241089
ENSGALG00010021092	0.935567309	0.258447291
ENSGALG00010021093	0.992152859	0.312526583
ENSGALG00010021094	0.216617034	0.076842875
ENSGALG00010021095	0.952050909	0.341041261
ENSGALG00010021096	0.909509002	0.260605608
ENSGALG00010021097	0.136947146	0.268309739
ENSGALG00010021098	0.839698454	0.223729724
ENSGALG00010021099	0.036589361	0.309600981

ENSGALG00010021100	0.147544011	0.079060464
ENSGALG00010021102	0.8523105	0.249539672
ENSGALG00010021103	0.761891907	0.43237284
ENSGALG00010021104	0.470466534	0.185769266
ENSGALG00010021105	0.625821429	0.431032002
ENSGALG00010021106	0.861192621	0.299793467
ENSGALG00010021107	0.482292316	0.278342016
ENSGALG00010021108	0.734389445	0.397857524
ENSGALG00010021109	0.025977801	0.148665342
ENSGALG00010021110	0.997566311	0.332254123
ENSGALG00010021111	0.987845492	0.303309872
ENSGALG00010021112	0.494544772	0.212999153
ENSGALG00010021113	0.666350691	0.295125779
ENSGALG00010021114	0.985729065	0.325621479
ENSGALG00010021115	0.901578408	0.256368564
ENSGALG00010021116	0.950059089	0.23958428
ENSGALG00010021117	0.989732564	0.357265739
ENSGALG00010021118	0.800854053	0.413957545
ENSGALG00010021120	0.547698073	0.149707754
ENSGALG00010021121	0.963872126	0.282574065
ENSGALG00010021122	0.965167822	0.326947545
ENSGALG00010021123	0.993610517	0.320733152
ENSGALG00010021124	0.433464225	0.186137581
ENSGALG00010021125	0.22046875	0.595801529
ENSGALG00010021126	0.94204959	0.220729626
ENSGALG00010021127	0.920761862	0.381607981
ENSGALG00010021128	0.208550923	0.676301294
ENSGALG00010021129	0.609422275	0.317948122
ENSGALG00010021130	0.990022768	0.30851861
ENSGALG00010021131	0.288837326	0.096909964
ENSGALG00010021132	0.301772951	0.966506966
ENSGALG00010021133	0.821138388	0.244324065
ENSGALG00010021134	0.968800269	0.427331756
ENSGALG00010021135	0.869848801	0.229076178
ENSGALG00010021136	0.356674995	0.077564006
ENSGALG00010021137	0.95499035	0.240200574
ENSGALG00010021138	0.315473351	0.24335246
ENSGALG00010021139	0.846857098	0.058634647
ENSGALG00010021141	0.959828485	0.475512576
ENSGALG00010021142	0.463363719	0.389756237
ENSGALG00010021143	0.438678746	0.103266126
ENSGALG00010021144	0.83023731	0.218032551
ENSGALG00010021145	0.801663607	0.167520196
ENSGALG00010021146	0.559379703	0.1605864
ENSGALG00010021147	0.805688679	0.23462254
ENSGALG00010021148	0.729959565	0.155801824
ENSGALG00010021152	0.161821862	0.098757786
ENSGALG00010021153	0.972385652	0.28425539
ENSGALG00010021154	0.977693634	0.292174643
ENSGALG00010021155	0.874628471	0.485277681
ENSGALG00010021156	0.972235746	0.30009525
ENSGALG00010021157	0.910302878	0.309150584
ENSGALG00010021158	0.527825769	0.389746731
ENSGALG00010021159	0.992426083	0.359268691
ENSGALG00010021160	0.342500778	0.030022035
ENSGALG00010021161	0.86983055	0.453711963

ENSGALG00010021162	0.905527013	0.213930254
ENSGALG00010021163	0.735906337	0.14924234
ENSGALG00010021164	0.705164452	0.161622806
ENSGALG00010021165	0.991099849	0.372264436
ENSGALG00010021166	0.894429328	0.164682432
ENSGALG00010021167	0.970607293	0.317434408
ENSGALG00010021168	0.816116778	0.320379746
ENSGALG00010021169	0.991846576	0.337795295
ENSGALG00010021170	0.723942706	0.392407596
ENSGALG00010021171	0.372864851	0.219372345
ENSGALG00010021172	0.274506148	0.237011335
ENSGALG00010021173	0.761087273	0.425204459
ENSGALG00010021174	0.544393435	0.148577283
ENSGALG00010021175	0.915164927	0.278886278
ENSGALG00010021176	0.77819588	0.23926245
ENSGALG00010021177	0.987543418	0.320177512
ENSGALG00010021178	0.989800342	0.358909475
ENSGALG00010021180	0.961243281	0.340443005
ENSGALG00010021181	0.885890176	0.172535822
ENSGALG00010021182	0.41706746	0.156331863
ENSGALG00010021183	0.848805203	0.130910889
ENSGALG00010021184	0.592520341	0.591458683
ENSGALG00010021185	0.238945004	0.401007102
ENSGALG00010021186	0.759519407	0.042104238
ENSGALG00010021187	0.928246985	0.275265703
ENSGALG00010021188	0.005716958	0.208013707
ENSGALG00010021189	0.876172444	0.327651669
ENSGALG00010021190	0.936135166	0.456130833
ENSGALG00010021191	0.968168651	0.277957781
ENSGALG00010021192	0.498549357	0.044109836
ENSGALG00010021193	0.747863473	0.361761791
ENSGALG00010021194	0.396530764	0.023277046
ENSGALG00010021195	0.964788738	0.236375686
ENSGALG00010021196	0.978264753	0.325622917
ENSGALG00010021197	0.950080447	0.230095738
ENSGALG00010021199	0.325440147	0.001715882
ENSGALG00010021200	0.814077063	0.360904935
ENSGALG00010021201	0.713745339	0.505548629
ENSGALG00010021202	0.988570578	0.369390927
ENSGALG00010021203	0.949224373	0.416897339
ENSGALG00010021204	0.530638225	0.100173848
ENSGALG00010021205	0.452248863	0.162896312
ENSGALG00010021206	0.045665413	0.009527025
ENSGALG00010021207	0.919821803	0.090781201
ENSGALG00010021208	0.211022858	0.278044165
ENSGALG00010021210	0.947053408	0.350119368
ENSGALG00010021211	0.28416405	0.283723572
ENSGALG00010021212	0.994249349	0.30123471
ENSGALG00010021213	0.876730973	0.174890759
ENSGALG00010021214	0.790854933	0.226076673
ENSGALG00010021215	0.975126551	0.321378236
ENSGALG00010021217	0.815997182	0.436258775
ENSGALG00010021218	0.741015198	0.234942005
ENSGALG00010021220	0.985941439	0.339083372
ENSGALG00010021221	0.313684988	0.055668615
ENSGALG00010021222	0.471170925	0.320973334

ENSGALG00010021223	0.403059424	0.120443769
ENSGALG00010021224	0.986348488	0.334185926
ENSGALG00010021225	0.910576438	0.330115112
ENSGALG00010021227	0.960630868	0.297888823
ENSGALG00010021228	0.997105832	0.335912631
ENSGALG00010021229	0.989736712	0.339329706
ENSGALG00010021230	0.616685287	0.123200582
ENSGALG00010021231	0.772727049	0.089989664
ENSGALG00010021232	0.927844881	0.226217132
ENSGALG00010021233	0.282715514	0.232347252
ENSGALG00010021234	0.338962852	0.220700174
ENSGALG00010021235	0.867157748	0.278889398
ENSGALG00010021236	0.21723332	0.17470015
ENSGALG00010021237	0.420719908	0.342286786
ENSGALG00010021238	0.947691437	0.308254731
ENSGALG00010021239	0.535593617	0.198249774
ENSGALG00010021240	0.922288198	0.276069393
ENSGALG00010021241	0.245815091	0.22680516
ENSGALG00010021242	0.232917055	0.094533741
ENSGALG00010021243	0.497654955	0.390933862
ENSGALG00010021244	0.972567803	0.273037241
ENSGALG00010021245	0.975124716	0.277455204
ENSGALG00010021246	0.962698549	0.308606619
ENSGALG00010021247	0.668278438	0.319993832
ENSGALG00010021248	0.952734226	0.239138111
ENSGALG00010021249	0.396514982	0.355208977
ENSGALG00010021250	0.854954138	0.436397097
ENSGALG00010021251	0.620293089	0.003804214
ENSGALG00010021252	0.083657292	0.305515726
ENSGALG00010021253	0.353203363	0.124657082
ENSGALG00010021254	0.768124826	0.299840357
ENSGALG00010021255	0.886951447	0.332957262
ENSGALG00010021256	0.906838706	0.307486624
ENSGALG00010021257	0.523085699	0.176300784
ENSGALG00010021258	0.467260937	0.092809959
ENSGALG00010021259	0.435568589	0.301395022
ENSGALG00010021260	0.765559042	0.288710238
ENSGALG00010021261	0.97514771	0.424851968
ENSGALG00010021262	0.199091409	0.008690348
ENSGALG00010021263	0.838031539	0.220781682
ENSGALG00010021264	0.252240304	0.173980408
ENSGALG00010021265	0.083490404	0.082355717
ENSGALG00010021266	0.417590842	0.270759894
ENSGALG00010021267	0.337732685	0.202448997
ENSGALG00010021268	0.270451009	0.090541667
ENSGALG00010021269	0.759283749	0.198433749
ENSGALG00010021271	0.977903605	0.348328704
ENSGALG00010021272	0.894241347	0.375019679
ENSGALG00010021275	0.952798699	0.488254485
ENSGALG00010021276	0.85022333	0.295974603
ENSGALG00010021277	0.278555151	0.379192236
ENSGALG00010021278	0.354842934	0.074344186
ENSGALG00010021280	0.396365709	0.157137627
ENSGALG00010021281	0.617004757	0.316574516
ENSGALG00010021282	0.064282799	0.176650367
ENSGALG00010021283	0.488293262	0.007527113

ENSGALG00010021284	0.769419096	0.160893678
ENSGALG00010021285	0.110907219	0.039739511
ENSGALG00010021286	0.130058858	0.155301609
ENSGALG00010021287	0.812791731	0.30011579
ENSGALG00010021288	0.480337673	0.31086332
ENSGALG00010021290	0.587650857	0.315246583
ENSGALG00010021292	0.105945485	0.229581322
ENSGALG00010021293	0.984941765	0.316715041
ENSGALG00010021294	0.78696639	0.568791048
ENSGALG00010021295	0.54330064	0.131568803
ENSGALG00010021296	0.957860206	0.35790694
ENSGALG00010021298	0.121115808	0.401444111
ENSGALG00010021299	0.053819373	0.056702352
ENSGALG00010021301	0.983087221	0.355303763
ENSGALG00010021302	0.016423554	0.009302513
ENSGALG00010021303	0.742725675	0.359398826
ENSGALG00010021307	0.711173504	0.006534254
ENSGALG00010021308	0.107122696	0.18196647
ENSGALG00010021309	0.93357536	0.323275108
ENSGALG00010021311	0.821167551	0.42907811
ENSGALG00010021312	0.983305491	0.33336323
ENSGALG00010021313	0.612509828	0.306224167
ENSGALG00010021314	0.078047127	0.032127636
ENSGALG00010021315	0.107723114	0.22629214
ENSGALG00010021316	0.988604248	0.342015738
ENSGALG00010021317	0.013744607	0.263681249
ENSGALG00010021318	0.884666388	0.212873503
ENSGALG00010021320	0.978375928	0.251381462
ENSGALG00010021321	0.336118445	0.277401528
ENSGALG00010021325	0.908630189	0.40758817
ENSGALG00010021327	0.960208738	0.411794199
ENSGALG00010021329	0.773509858	0.18168102
ENSGALG00010021330	0.798693348	0.365306784
ENSGALG00010021332	0.994084318	0.328952924
ENSGALG00010021333	0.408511391	0.051283331
ENSGALG00010021334	0.372365604	0.116154215
ENSGALG00010021335	0.661394151	0.378354015
ENSGALG00010021337	0.760847613	0.214134821
ENSGALG00010021338	0.977145697	0.35023091
ENSGALG00010021339	0.789306894	0.105377821
ENSGALG00010021340	0.178690539	0.104880205
ENSGALG00010021341	0.888556749	0.250946482
ENSGALG00010021342	0.49421039	0.014551216
ENSGALG00010021344	0.356494703	0.029773258
ENSGALG00010021345	0.886741446	0.102236835
ENSGALG00010021346	0.806544962	0.32323063
ENSGALG00010021348	0.956709313	0.296371105
ENSGALG00010021350	0.74267398	0.34324766
ENSGALG00010021352	0.618005071	0.430176942
ENSGALG00010021353	0.987646816	0.277493796
ENSGALG00010021354	0.848199006	0.285831948
ENSGALG00010021357	0.581934035	0.148412937
ENSGALG00010021358	0.926307	0.382177934
ENSGALG00010021360	0.882049588	0.50270681
ENSGALG00010021361	0.814106223	0.215565892
ENSGALG00010021362	0.830984815	0.197827548

ENSGALG00010021363	0.959032671	0.364988281
ENSGALG00010021366	0.900888961	0.156382604
ENSGALG00010021367	0.818709501	0.129344398
ENSGALG00010021369	0.235630588	0.132393411
ENSGALG00010021370	0.839631042	0.07048721
ENSGALG00010021371	0.08314654	0.268325236
ENSGALG00010021372	0.903906743	0.070562637
ENSGALG00010021373	0.614487963	0.270396945
ENSGALG00010021374	0.822528308	0.287752074
ENSGALG00010021375	0.826785315	0.262351253
ENSGALG00010021376	0.169144926	0.246837998
ENSGALG00010021377	0.557583216	0.15756993
ENSGALG00010021378	0.699845186	0.24492978
ENSGALG00010021379	0.717595251	0.518974717
ENSGALG00010021380	0.453139391	0.301014156
ENSGALG00010021381	0.995848195	0.309035849
ENSGALG00010021382	0.939329019	0.21682364
ENSGALG00010021383	0.906628179	0.340241562
ENSGALG00010021384	0.495000548	0.190954161
ENSGALG00010021386	0.026265784	0.082890826
ENSGALG00010021387	0.576759017	0.001354273
ENSGALG00010021388	0.478046386	0.544765308
ENSGALG00010021390	0.8925425	0.481397609
ENSGALG00010021391	0.885297158	0.13849728
ENSGALG00010021392	0.769811148	0.033352841
ENSGALG00010021393	0.179906894	0.194351137
ENSGALG00010021394	0.95841121	0.321537707
ENSGALG00010021395	0.9833474	0.351399176
ENSGALG00010021396	0.526665948	0.05497845
ENSGALG00010021399	0.222464	0.195401375
ENSGALG00010021400	0.987409604	0.315924789
ENSGALG00010021401	0.97516903	0.28583422
ENSGALG00010021402	0.809753067	0.152147221
ENSGALG00010021404	0.92558452	0.284441885
ENSGALG00010021405	0.981822141	0.294851973
ENSGALG00010021406	0.309926173	0.088116819
ENSGALG00010021407	0.710915206	0.001038817
ENSGALG00010021408	0.823237984	0.523193207
ENSGALG00010021409	0.914011366	0.214922883
ENSGALG00010021411	0.98825773	0.334395262
ENSGALG00010021412	0.947646482	0.404760362
ENSGALG00010021413	0.789964609	0.117807461
ENSGALG00010021414	0.94294098	0.28163113
ENSGALG00010021415	0.753166971	0.155723932
ENSGALG00010021416	0.982150271	0.396940169
ENSGALG00010021417	0.529272171	0.128227065
ENSGALG00010021419	0.239201584	0.261312635
ENSGALG00010021420	0.563817705	0.324551313
ENSGALG00010021421	0.882695567	0.301498393
ENSGALG00010021423	0.214232768	0.198854563
ENSGALG00010021424	0.893437181	0.271558933
ENSGALG00010021425	0.629217692	0.215248989
ENSGALG00010021426	0.924162783	0.376869465
ENSGALG00010021427	0.824976516	0.369980989
ENSGALG00010021429	0.933962985	0.334147595
ENSGALG00010021431	0.831924498	0.258129699

ENSGALG00010021432	0.298009839	0.475159739
ENSGALG00010021433	0.943830978	0.29629444
ENSGALG00010021434	0.423002515	0.104793551
ENSGALG00010021435	0.967353001	0.22438581
ENSGALG00010021436	0.360841789	0.245455387
ENSGALG00010021438	0.945108981	0.283424856
ENSGALG00010021439	0.974999709	0.283752358
ENSGALG00010021440	0.209224158	0.141084697
ENSGALG00010021441	0.627523992	0.22858539
ENSGALG00010021443	0.869413922	0.296662881
ENSGALG00010021444	0.982354231	0.305633025
ENSGALG00010021447	0.348032803	0.146426442
ENSGALG00010021448	0.819527261	0.333443094
ENSGALG00010021450	0.707534729	0.212488698
ENSGALG00010021452	0.992537281	0.291532995
ENSGALG00010021453	0.848546034	0.486005203
ENSGALG00010021454	0.784605464	0.264186249
ENSGALG00010021455	0.61320549	0.063273901
ENSGALG00010021457	0.855921942	0.230180647
ENSGALG00010021458	0.778249026	0.3981127
ENSGALG00010021459	0.963374211	0.330254707
ENSGALG00010021460	0.540360415	0.242292095
ENSGALG00010021461	0.529760564	0.094041594
ENSGALG00010021462	0.353331941	0.071532956
ENSGALG00010021463	0.506652925	0.067505744
ENSGALG00010021464	0.945237895	0.27891948
ENSGALG00010021465	0.979421832	0.301385059
ENSGALG00010021466	0.810080048	0.364181565
ENSGALG00010021467	0.566701848	0.022229614
ENSGALG00010021468	0.925589669	0.296106358
ENSGALG00010021469	0.965366319	0.331796089
ENSGALG00010021470	0.880481047	0.183210263
ENSGALG00010021472	0.57605649	0.407257104
ENSGALG00010021473	0.948668761	0.28739796
ENSGALG00010021474	0.96635828	0.472458556
ENSGALG00010021475	0.702445938	0.379198528
ENSGALG00010021476	0.119116039	0.271351263
ENSGALG00010021477	0.726301529	0.340659009
ENSGALG00010021478	0.896480702	0.211053323
ENSGALG00010021479	0.834627285	0.515466876
ENSGALG00010021480	0.910182823	0.291273843
ENSGALG00010021481	0.953055802	0.358395288
ENSGALG00010021482	0.144693667	0.428850417
ENSGALG00010021483	0.89616453	0.309391721
ENSGALG00010021484	0.566379864	0.050258776
ENSGALG00010021485	0.19968793	0.024506792
ENSGALG00010021487	0.340069836	0.197736436
ENSGALG00010021488	0.769967476	0.110486625
ENSGALG00010021489	0.488992148	0.139362686
ENSGALG00010021490	0.6317296	0.218691585
ENSGALG00010021492	0.933878529	0.420633794
ENSGALG00010021493	0.970127447	0.267911585
ENSGALG00010021494	0.911626893	0.208773514
ENSGALG00010021495	0.602847268	0.160291358
ENSGALG00010021496	0.893839163	0.180696445
ENSGALG00010021497	0.009346827	0.215206228

ENSGALG00010021498	0.919529217	0.298223101
ENSGALG00010021499	0.659380641	0.166734484
ENSGALG00010021500	0.933927787	0.180568488
ENSGALG00010021501	0.363075546	0.281950975
ENSGALG00010021502	0.904712126	0.234141072
ENSGALG00010021503	0.738426689	0.213495084
ENSGALG00010021504	0.950480525	0.421941368
ENSGALG00010021505	0.935796015	0.382486478
ENSGALG00010021506	0.241299605	0.09007608
ENSGALG00010021507	0.072280778	0.12225007
ENSGALG00010021508	0.381842179	0.231163936
ENSGALG00010021509	0.964557174	0.30427256
ENSGALG00010021510	0.972376219	0.264344494
ENSGALG00010021511	0.024272986	0.203024922
ENSGALG00010021512	0.676647074	0.264425584
ENSGALG00010021513	0.819530058	0.078132839
ENSGALG00010021514	0.961786029	0.325700261
ENSGALG00010021515	0.939982598	0.299979706
ENSGALG00010021516	0.50790501	0.261863211
ENSGALG00010021517	0.154837879	0.024680553
ENSGALG00010021518	0.459926806	0.132413853
ENSGALG00010021519	0.990732347	0.363872495
ENSGALG00010021520	0.448278173	0.154751904
ENSGALG00010021521	0.475893774	0.012334798
ENSGALG00010021522	0.812577502	0.293441603
ENSGALG00010021523	0.811083847	0.304204807
ENSGALG00010021524	0.103192516	0.33770639
ENSGALG00010021525	0.823383161	0.200870609
ENSGALG00010021526	0.984602709	0.350060867
ENSGALG00010021527	0.507951217	0.266445882
ENSGALG00010021528	0.95322725	0.280657187
ENSGALG00010021529	0.738551157	0.284655291
ENSGALG00010021530	0.24255666	0.069588525
ENSGALG00010021531	0.415304269	0.091288242
ENSGALG00010021532	0.290771179	0.089898397
ENSGALG00010021533	0.066212143	0.43799137
ENSGALG00010021534	0.94759499	0.289719891
ENSGALG00010021535	0.902663288	0.354296184
ENSGALG00010021536	0.384387589	0.602708267
ENSGALG00010021537	0.186934798	0.402872853
ENSGALG00010021538	0.886586265	0.245316504
ENSGALG00010021539	0.93241022	0.245875981
ENSGALG00010021540	0.984942238	0.324146019
ENSGALG00010021541	0.957418807	0.33939942
ENSGALG00010021543	0.608205762	0.312662424
ENSGALG00010021545	0.989574671	0.3384259
ENSGALG00010021546	0.56962571	0.46398112
ENSGALG00010021547	0.396668506	0.181671381
ENSGALG00010021549	0.97048101	0.39053354
ENSGALG00010021550	0.897820269	0.349731068
ENSGALG00010021551	0.605576273	0.167183587
ENSGALG00010021552	0.971614851	0.33631496
ENSGALG00010021553	0.98630468	0.296935275
ENSGALG00010021554	0.611749252	0.212190167
ENSGALG00010021555	0.268346037	0.249143613
ENSGALG00010021556	0.951487423	0.252679896

ENSGALG00010021557	0.499571562	0.142812618
ENSGALG00010021558	0.986634134	0.3318284
ENSGALG00010021559	0.871762378	0.322800002
ENSGALG00010021560	0.598169158	0.2162124
ENSGALG00010021561	0.931081963	0.413604501
ENSGALG00010021562	0.954142924	0.291893197
ENSGALG00010021564	0.987878255	0.302732125
ENSGALG00010021565	0.96892782	0.309114099
ENSGALG00010021566	0.672096447	0.123206994
ENSGALG00010021568	0.966929566	0.33083126
ENSGALG00010021569	0.702022904	0.032681104
ENSGALG00010021570	0.864410743	0.337175449
ENSGALG00010021571	0.912664203	0.322194852
ENSGALG00010021572	0.961647469	0.380993923
ENSGALG00010021573	0.926184431	0.296874831
ENSGALG00010021574	0.954996459	0.340819862
ENSGALG00010021575	0.105747864	0.131309275
ENSGALG00010021576	0.89303679	0.169742694
ENSGALG00010021577	0.993172581	0.319591306
ENSGALG00010021578	0.92737744	0.3058951
ENSGALG00010021579	0.97442743	0.289257679
ENSGALG00010021580	0.752241556	0.119426116
ENSGALG00010021581	0.472657959	0.124919375
ENSGALG00010021582	0.079630903	0.163135907
ENSGALG00010021583	0.97958253	0.322990757
ENSGALG00010021584	0.870113099	0.28587354
ENSGALG00010021585	0.938738693	0.256225113
ENSGALG00010021586	0.386317418	0.100081823
ENSGALG00010021587	0.902461099	0.251068338
ENSGALG00010021588	0.889641204	0.380443434
ENSGALG00010021590	0.13947765	0.15261326
ENSGALG00010021591	0.48798866	0.177542328
ENSGALG00010021593	0.648557765	0.081767849
ENSGALG00010021594	0.835187067	0.263359157
ENSGALG00010021595	0.656481078	0.127197643
ENSGALG00010021597	0.853924177	0.217787297
ENSGALG00010021599	0.747157315	0.259203387
ENSGALG00010021600	0.941154931	0.307353926
ENSGALG00010021601	0.840047556	0.090120511
ENSGALG00010021602	0.950193458	0.257444274
ENSGALG00010021603	0.021324362	0.253113905
ENSGALG00010021604	0.982067895	0.309978172
ENSGALG00010021605	0.16505232	0.14714086
ENSGALG00010021606	0.920771327	0.114430318
ENSGALG00010021607	0.329738642	0.028090181
ENSGALG00010021608	0.480593894	0.175264503
ENSGALG00010021609	0.952629495	0.320153951
ENSGALG00010021610	0.964977603	0.322211116
ENSGALG00010021611	0.892793397	0.322751261
ENSGALG00010021612	0.890011075	0.431462307
ENSGALG00010021613	0.477586967	0.009722635
ENSGALG00010021614	0.180831451	0.025295205
ENSGALG00010021615	0.880304777	0.096098152
ENSGALG00010021616	0.941784602	0.416113146
ENSGALG00010021617	0.558742622	0.135232823
ENSGALG00010021618	0.435266042	0.04291818

ENSGALG00010021619	0.995361811	0.342048595
ENSGALG00010021620	0.526612595	0.192636446
ENSGALG00010021621	0.97317492	0.291324684
ENSGALG00010021622	0.807236674	0.463490271
ENSGALG00010021623	0.97955549	0.31941656
ENSGALG00010021624	0.664059174	0.120036509
ENSGALG00010021626	0.982924689	0.294345152
ENSGALG00010021627	0.727421674	0.322941162
ENSGALG00010021628	0.876820495	0.369190059
ENSGALG00010021629	0.978465466	0.33821921
ENSGALG00010021630	0.222856819	0.396279848
ENSGALG00010021631	0.815672249	0.123579478
ENSGALG00010021632	0.9031505	0.290054454
ENSGALG00010021633	0.914948453	0.198787372
ENSGALG00010021634	0.972816174	0.308248215
ENSGALG00010021635	0.982224091	0.343811747
ENSGALG00010021636	0.326554699	0.124094753
ENSGALG00010021637	0.374130813	0.076000432
ENSGALG00010021638	0.97850001	0.244485004
ENSGALG00010021639	0.194978575	0.206766921
ENSGALG00010021640	0.112514054	0.081532262
ENSGALG00010021641	0.129174336	0.296426611
ENSGALG00010021642	0.617857062	0.1934846
ENSGALG00010021643	0.882530739	0.319056157
ENSGALG00010021644	0.722662092	0.450407046
ENSGALG00010021645	0.948117239	0.324313189
ENSGALG00010021646	0.947357301	0.289412132
ENSGALG00010021647	0.959260136	0.4191708
ENSGALG00010021648	0.961461339	0.26899199
ENSGALG00010021649	0.981377407	0.293674526
ENSGALG00010021650	0.785415908	0.007455934
ENSGALG00010021651	0.899329737	0.190215028
ENSGALG00010021652	0.707051105	0.411997397
ENSGALG00010021655	0.733625132	0.294455221
ENSGALG00010021656	0.924746455	0.225773759
ENSGALG00010021657	0.914643951	0.244930684
ENSGALG00010021658	0.813204286	0.201190256
ENSGALG00010021659	0.953030803	0.387011013
ENSGALG00010021660	0.952901211	0.268274945
ENSGALG00010021661	0.919058524	0.283079359
ENSGALG00010021662	0.81055915	0.265398878
ENSGALG00010021663	0.983048417	0.325258389
ENSGALG00010021664	0.698358445	0.291620135
ENSGALG00010021665	0.102593712	0.211517358
ENSGALG00010021666	0.93920369	0.367029145
ENSGALG00010021667	0.994309003	0.339017672
ENSGALG00010021668	0.939572862	0.171422767
ENSGALG00010021669	0.324441194	0.168858887
ENSGALG00010021670	0.997368465	0.339337846
ENSGALG00010021671	0.748910818	0.223921596
ENSGALG00010021672	0.697752389	0.352177091
ENSGALG00010021674	0.953742196	0.445759798
ENSGALG00010021675	0.153340564	0.061027572
ENSGALG00010021676	0.795190836	0.297861344
ENSGALG00010021677	0.942812879	0.276207545
ENSGALG00010021678	0.646023493	0.229609435

ENSGALG00010021679	0.983942034	0.294270898
ENSGALG00010021680	0.398065772	0.227683145
ENSGALG00010021681	0.94056105	0.270862541
ENSGALG00010021682	0.967107545	0.29875333
ENSGALG00010021683	0.348507506	0.083315492
ENSGALG00010021684	0.991851422	0.329367466
ENSGALG00010021685	0.990886035	0.327954704
ENSGALG00010021686	0.709131474	0.143455285
ENSGALG00010021687	0.469259003	0.050254553
ENSGALG00010021688	0.908838419	0.309552483
ENSGALG00010021689	0.960932426	0.293080571
ENSGALG00010021690	0.12495962	0.137192538
ENSGALG00010021691	0.250749635	0.171170121
ENSGALG00010021692	0.935786941	0.336991549
ENSGALG00010021693	0.782879574	0.060306595
ENSGALG00010021694	0.845201663	0.325984662
ENSGALG00010021695	0.724582799	0.411199828
ENSGALG00010021696	0.394555696	0.165889936
ENSGALG00010021697	0.6158374	0.057085973
ENSGALG00010021698	0.71056046	0.190986286
ENSGALG00010021699	0.563144304	0.294068842
ENSGALG00010021700	0.661534044	0.367096212
ENSGALG00010021701	0.825157924	0.162349927
ENSGALG00010021702	0.649030336	0.193147706
ENSGALG00010021703	0.944155795	0.252666438
ENSGALG00010021704	0.994059122	0.332574089
ENSGALG00010021705	0.687030624	0.353299128
ENSGALG00010021706	0.962703241	0.273357009
ENSGALG00010021707	0.235630588	0.132393411
ENSGALG00010021708	0.213066302	0.184369322
ENSGALG00010021709	0.022908794	0.24480423
ENSGALG00010021710	0.750525022	0.334164612
ENSGALG00010021711	0.935285379	0.335670985
ENSGALG00010021713	0.973149484	0.375069933
ENSGALG00010021714	0.125113197	0.122244335
ENSGALG00010021715	0.667473042	0.251634081
ENSGALG00010021716	0.961924884	0.324214324
ENSGALG00010021717	0.85571602	0.314032969
ENSGALG00010021718	0.232025493	0.462527537
ENSGALG00010021719	0.944896157	0.339566453
ENSGALG00010021720	0.988538682	0.31221772
ENSGALG00010021721	0.751365722	0.392280962
ENSGALG00010021722	0.729165669	0.210224585
ENSGALG00010021723	0.983622702	0.311789406
ENSGALG00010021724	0.629455916	0.091114927
ENSGALG00010021725	0.957935794	0.312712286
ENSGALG00010021726	0.02307017	0.176006377
ENSGALG00010021727	0.841038431	0.210881229
ENSGALG00010021728	0.9153994	0.261356697
ENSGALG00010021729	0.069722375	0.235962854
ENSGALG00010021730	0.92212795	0.269075888
ENSGALG00010021731	0.928265503	0.345528281
ENSGALG00010021732	0.168800679	0.059729037
ENSGALG00010021733	0.989249708	0.290724707
ENSGALG00010021734	0.6323964	0.246772453
ENSGALG00010021735	0.99000216	0.338438708

ENSGALG00010021736	0.993378545	0.287073606
ENSGALG00010021737	0.711195879	0.114505029
ENSGALG00010021738	0.984538805	0.348554255
ENSGALG00010021739	0.825880639	0.159546736
ENSGALG00010021740	0.490720275	0.095682594
ENSGALG00010021741	0.812736482	0.282667323
ENSGALG00010021742	0.449776562	0.046501443
ENSGALG00010021744	0.938407306	0.306895829
ENSGALG00010021745	0.907947572	0.141670306
ENSGALG00010021746	0.941552001	0.248149404
ENSGALG00010021747	0.920669485	0.39364239
ENSGALG00010021748	0.398208492	0.151400832
ENSGALG00010021749	0.966136008	0.238386042
ENSGALG00010021750	0.880436803	0.141307382
ENSGALG00010021751	0.931500634	0.315737841
ENSGALG00010021752	0.076627257	0.214412405
ENSGALG00010021753	0.82817062	0.487447742
ENSGALG00010021754	0.994277601	0.365274335
ENSGALG00010021755	0.88496426	0.269011699
ENSGALG00010021756	0.801797158	0.384365839
ENSGALG00010021757	0.534842868	0.304617881
ENSGALG00010021758	0.394888276	0.298935234
ENSGALG00010021759	0.408212362	0.16109877
ENSGALG00010021760	0.213236643	0.09979905
ENSGALG00010021761	0.881389284	0.284621256
ENSGALG00010021762	0.954147707	0.31912579
ENSGALG00010021763	0.811108771	0.410273004
ENSGALG00010021764	0.976920215	0.251571217
ENSGALG00010021765	0.370520025	0.502856064
ENSGALG00010021766	0.84584482	0.180354648
ENSGALG00010021767	0.773641232	0.218398578
ENSGALG00010021768	0.957923392	0.330996947
ENSGALG00010021769	0.600887273	0.443914207
ENSGALG00010021770	0.453425501	0.01239588
ENSGALG00010021771	0.965871119	0.277820389
ENSGALG00010021772	0.44285577	0.561283145
ENSGALG00010021774	0.095720953	0.085467438
ENSGALG00010021775	0.640712135	0.278439643
ENSGALG00010021776	0.90704045	0.244149619
ENSGALG00010021777	0.244699373	0.20034948
ENSGALG00010021778	0.934994708	0.257126745
ENSGALG00010021779	0.685237898	0.351396742
ENSGALG00010021781	0.856722632	0.139662299
ENSGALG00010021782	0.892898515	0.385097461
ENSGALG00010021783	0.45058772	0.602995055
ENSGALG00010021784	0.031846118	0.162328396
ENSGALG00010021785	0.667948809	0.004041992
ENSGALG00010021786	0.801752172	0.359536978
ENSGALG00010021788	0.856084374	0.273613413
ENSGALG00010021789	0.889352148	0.309089118
ENSGALG00010021790	0.150179848	0.022381454
ENSGALG00010021791	0.987875752	0.297316792
ENSGALG00010021792	0.97105846	0.358684017
ENSGALG00010021793	0.376356239	0.040919961
ENSGALG00010021794	0.769669948	0.170925122
ENSGALG00010021795	0.849242279	0.300035432

ENSGALG00010021796	0.208550923	0.676301294
ENSGALG00010021797	0.661003279	0.429537324
ENSGALG00010021798	0.35517674	0.08512991
ENSGALG00010021799	0.938601003	0.329248996
ENSGALG00010021800	0.96787262	0.403100937
ENSGALG00010021801	0.84872831	0.372593007
ENSGALG00010021803	0.824964879	0.20099551
ENSGALG00010021805	0.130761128	0.433881428
ENSGALG00010021807	0.951839822	0.273607856
ENSGALG00010021808	0.586890164	0.154015663
ENSGALG00010021809	0.098711169	0.292522257
ENSGALG00010021810	0.982391715	0.336503623
ENSGALG00010021812	0.879386015	0.227850334
ENSGALG00010021813	0.967343929	0.325509367
ENSGALG00010021816	0.319027225	0.065581945
ENSGALG00010021817	0.944017349	0.318316094
ENSGALG00010021818	0.895261024	0.178031987
ENSGALG00010021819	0.906079281	0.413762036
ENSGALG00010021820	0.465469044	0.191936734
ENSGALG00010021821	0.974987581	0.328443408
ENSGALG00010021823	0.133066531	0.094016123
ENSGALG00010021825	0.990438954	0.337514295
ENSGALG00010021826	0.993751793	0.346442621
ENSGALG00010021827	0.877799907	0.392126081
ENSGALG00010021829	0.502949535	0.224604419
ENSGALG00010021831	0.499630621	0.01576159
ENSGALG00010021832	0.405321009	0.397160722
ENSGALG00010021834	0.265558089	0.028821141
ENSGALG00010021835	0.041884014	0.070068395
ENSGALG00010021837	0.089090811	0.17241992
ENSGALG00010021838	0.318434782	0.008370214
ENSGALG00010021839	0.353535039	0.130781335
ENSGALG00010021840	0.176832663	0.193967005
ENSGALG00010021841	0.614127319	0.371794761
ENSGALG00010021842	0.437775118	0.38052896
ENSGALG00010021843	0.945835228	0.317020013
ENSGALG00010021844	0.352052777	0.170637509
ENSGALG00010021846	0.424243998	0.135578444
ENSGALG00010021847	0.75120015	0.246481328
ENSGALG00010021848	0.762298862	0.193827528
ENSGALG00010021849	0.032901312	0.101028217
ENSGALG00010021850	0.259861278	0.049935416
ENSGALG00010021851	0.795689766	0.248825597
ENSGALG00010021853	0.961697457	0.30622826
ENSGALG00010021854	0.858281959	0.226668746
ENSGALG00010021856	0.203991683	0.058221925
ENSGALG00010021860	0.26802757	0.428800267
ENSGALG00010021861	0.945080816	0.229228055
ENSGALG00010021862	0.807829126	0.246490991
ENSGALG00010021863	0.750032913	0.225299216
ENSGALG00010021864	0.810278676	0.158744833
ENSGALG00010021865	0.312822111	0.367410415
ENSGALG00010021866	0.063446542	0.053959664
ENSGALG00010021867	0.045985391	0.177518246
ENSGALG00010021869	0.731429485	0.414698643
ENSGALG00010021870	0.548728997	0.264720413

ENSGALG00010021871	0.996189042	0.343415978
ENSGALG00010021874	0.85844692	0.208890137
ENSGALG00010021875	0.370493859	0.13155368
ENSGALG00010021876	0.054255029	0.496773776
ENSGALG00010021877	0.940366579	0.283475083
ENSGALG00010021879	0.308927052	0.069480796
ENSGALG00010021880	0.492260278	0.418948462
ENSGALG00010021881	0.711868313	0.222622212
ENSGALG00010021883	0.460011653	0.315823706
ENSGALG00010021885	0.550570079	0.3928606
ENSGALG00010021886	0.600100322	0.280917506
ENSGALG00010021887	0.962479636	0.279196345
ENSGALG00010021888	0.631449021	0.107638888
ENSGALG00010021889	0.013863674	0.10879459
ENSGALG00010021890	0.143647229	0.247379516
ENSGALG00010021892	0.815840805	0.245347914
ENSGALG00010021893	0.695320439	0.018567225
ENSGALG00010021897	0.843218659	0.15661911
ENSGALG00010021898	0.390688024	0.154376612
ENSGALG00010021899	0.05197934	0.14495833
ENSGALG00010021900	0.352449399	0.037405458
ENSGALG00010021902	0.846020863	0.391911396
ENSGALG00010021903	0.492572949	0.221509938
ENSGALG00010021905	0.198313271	0.151397209
ENSGALG00010021906	0.426196799	0.104791432
ENSGALG00010021908	0.823888848	0.285338239
ENSGALG00010021910	0.350431973	0.357190512
ENSGALG00010021912	0.035682459	0.136951644
ENSGALG00010021913	0.884635678	0.237073551
ENSGALG00010021916	0.770834239	0.253411407
ENSGALG00010021918	0.45921958	0.192543902
ENSGALG00010021919	0.817910982	0.192555359
ENSGALG00010021921	0.030780691	0.19932702
ENSGALG00010021922	0.922158673	0.265312726
ENSGALG00010021923	0.409811515	0.212720457
ENSGALG00010021924	0.858810409	0.359407199
ENSGALG00010021925	0.884512198	0.326257433
ENSGALG00010021927	0.582053862	0.090039234
ENSGALG00010021928	0.669247895	0.13294219
ENSGALG00010021929	0.588084591	0.101926705
ENSGALG00010021930	0.713395728	0.028180055
ENSGALG00010021931	0.92649192	0.351949739
ENSGALG00010021932	0.875765425	0.311094292
ENSGALG00010021933	0.832215033	0.254642617
ENSGALG00010021934	0.58516767	0.113340091
ENSGALG00010021935	0.425849182	0.368188691
ENSGALG00010021938	0.146680991	0.102923884
ENSGALG00010021939	0.941098926	0.359556923
ENSGALG00010021941	0.422656453	0.211461575
ENSGALG00010021942	0.607683692	0.126449819
ENSGALG00010021943	0.669835223	0.063412618
ENSGALG00010021944	0.231094662	0.151443066
ENSGALG00010021945	0.885506092	0.348190402
ENSGALG00010021946	0.307123807	0.205922273
ENSGALG00010021947	0.977121539	0.309763305
ENSGALG00010021948	0.910489644	0.295264563

ENSGALG00010021949	0.515559139	0.093972472
ENSGALG00010021950	0.643193011	0.038801785
ENSGALG00010021951	0.673249275	0.341184418
ENSGALG00010021952	0.854937916	0.039052359
ENSGALG00010021953	0.024042239	0.036913314
ENSGALG00010021957	0.376463374	0.101862289
ENSGALG00010021958	0.829096832	0.141581802
ENSGALG00010021960	0.960416553	0.279832197
ENSGALG00010021961	0.946205903	0.362951838
ENSGALG00010021962	0.126246052	0.097988911
ENSGALG00010021963	0.705029154	0.274467565
ENSGALG00010021965	0.500950635	0.143403802
ENSGALG00010021966	0.516872794	0.204609814
ENSGALG00010021967	0.252063895	0.027194544
ENSGALG00010021968	0.461777812	0.03487007
ENSGALG00010021969	0.554015385	0.112943724
ENSGALG00010021970	0.94052695	0.184668993
ENSGALG00010021971	0.849019646	0.16248781
ENSGALG00010021972	0.965738104	0.411926239
ENSGALG00010021973	0.607246705	0.514974372
ENSGALG00010021974	0.387086037	0.474139128
ENSGALG00010021975	0.633660994	0.186123752
ENSGALG00010021976	0.28880012	0.418789177
ENSGALG00010021978	0.412024031	0.178760902
ENSGALG00010021979	0.277648716	0.624295683
ENSGALG00010021980	0.697622377	0.012649063
ENSGALG00010021983	0.743200717	0.112555201
ENSGALG00010021984	0.290122505	0.464993379
ENSGALG00010021986	0.551057778	0.686989794
ENSGALG00010021987	0.943069351	0.301688302
ENSGALG00010021988	0.886150894	0.348430655
ENSGALG00010021989	0.100808788	0.035591072
ENSGALG00010021990	0.948875332	0.397641007
ENSGALG00010021991	0.475029225	0.165477163
ENSGALG00010021992	0.896222376	0.299813239
ENSGALG00010021994	0.505279404	0.182452181
ENSGALG00010021995	0.927752105	0.304437622
ENSGALG00010021996	0.355293024	0.212466313
ENSGALG00010021998	0.255478022	0.244308282
ENSGALG00010021999	0.846124425	0.30394201
ENSGALG00010022000	0.618255185	0.457775114
ENSGALG00010022001	0.976406933	0.336704934
ENSGALG00010022002	0.335813257	0.090432952
ENSGALG00010022003	0.002319943	0.47043011
ENSGALG00010022005	0.255062598	0.092778572
ENSGALG00010022006	0.949627686	0.291324651
ENSGALG00010022007	0.385574031	0.058624199
ENSGALG00010022008	0.567937867	0.154970481
ENSGALG00010022011	0.251164052	0.19860548
ENSGALG00010022012	0.479249347	0.150800206
ENSGALG00010022013	0.572332987	0.050647526
ENSGALG00010022014	0.385953062	0.133261917
ENSGALG00010022015	0.53528178	0.386809451
ENSGALG00010022017	0.350975867	0.03147439
ENSGALG00010022019	0.147671754	0.104310724
ENSGALG00010022020	0.203991683	0.058221925

ENSGALG00010022021	0.841205999	0.204547933
ENSGALG00010022022	0.073307264	0.231459874
ENSGALG00010022023	0.27332741	0.290269067
ENSGALG00010022024	0.922668501	0.275876309
ENSGALG00010022025	0.600563398	0.190472907
ENSGALG00010022026	0.495164822	0.62246793
ENSGALG00010022027	0.497818118	0.010178143
ENSGALG00010022028	0.000781198	0.136542139
ENSGALG00010022029	0.27602972	0.126880626
ENSGALG00010022030	0.653596109	0.327451007
ENSGALG00010022032	0.885257881	0.284209482
ENSGALG00010022033	0.952455593	0.214931165
ENSGALG00010022034	0.978243474	0.315976026
ENSGALG00010022035	0.290587188	0.257419453
ENSGALG00010022036	0.388899421	0.120062276
ENSGALG00010022037	0.964350206	0.264254291
ENSGALG00010022038	0.99718835	0.329779406
ENSGALG00010022039	0.009934226	0.071417744
ENSGALG00010022040	0.367689286	0.134680195
ENSGALG00010022041	0.963815688	0.288213837
ENSGALG00010022042	0.901797274	0.421482963
ENSGALG00010022043	0.356429521	0.314833398
ENSGALG00010022044	0.068152449	0.075241904
ENSGALG00010022046	0.700240438	0.018678678
ENSGALG00010022047	0.066331228	0.219417363
ENSGALG00010022048	0.661026369	0.371277264
ENSGALG00010022049	0.751142592	0.282548061
ENSGALG00010022050	0.612861148	0.283379033
ENSGALG00010022051	0.959370298	0.368124679
ENSGALG00010022052	0.807701703	0.110674965
ENSGALG00010022053	0.959500226	0.205173968
ENSGALG00010022054	0.932950009	0.427929706
ENSGALG00010022055	0.809796941	0.094314108
ENSGALG00010022056	0.584241619	0.153182535
ENSGALG00010022057	0.493036158	0.357625766
ENSGALG00010022058	0.791463746	0.067146789
ENSGALG00010022059	0.83250014	0.353324118
ENSGALG00010022061	0.8067515	0.407220685
ENSGALG00010022062	0.975509959	0.222267109
ENSGALG00010022063	0.786322587	0.170507773
ENSGALG00010022065	0.483002407	0.071835726
ENSGALG00010022066	0.721329599	0.248360955
ENSGALG00010022067	0.44768085	0.324207462
ENSGALG00010022069	0.85559188	0.380347277
ENSGALG00010022070	0.050344777	0.078939408
ENSGALG00010022071	0.833904389	0.118283473
ENSGALG00010022072	0.882517297	0.194528728
ENSGALG00010022073	0.970574072	0.26967401
ENSGALG00010022076	0.99083777	0.274762493
ENSGALG00010022078	0.90222255	0.234716428
ENSGALG00010022079	0.607691028	0.272905295
ENSGALG00010022081	0.922911838	0.305631467
ENSGALG00010022082	0.490394542	0.401813326
ENSGALG00010022084	0.789975557	0.050929306
ENSGALG00010022085	0.604719501	0.262001713
ENSGALG00010022086	0.627483312	0.247936486

ENSGALG00010022087	0.938141886	0.262267643
ENSGALG00010022088	0.583043632	0.187915471
ENSGALG00010022090	0.910928124	0.412882943
ENSGALG00010022091	0.522487999	0.016101952
ENSGALG00010022092	0.213849312	0.141928425
ENSGALG00010022093	0.931986176	0.278742004
ENSGALG00010022095	0.469098579	0.005285232
ENSGALG00010022096	0.387850048	0.007987487
ENSGALG00010022097	0.971030083	0.376507469
ENSGALG00010022098	0.650037351	0.08935652
ENSGALG00010022100	0.851038618	0.290734892
ENSGALG00010022101	0.467810792	0.067190185
ENSGALG00010022102	0.416084008	0.033397578
ENSGALG00010022104	0.253815956	0.482626714
ENSGALG00010022105	0.089327662	0.048919637
ENSGALG00010022106	0.553530935	0.25474138
ENSGALG00010022108	0.980043369	0.210486589
ENSGALG00010022110	0.158978089	0.103816627
ENSGALG00010022111	0.742644896	0.379220601
ENSGALG00010022112	0.300683727	0.461762941
ENSGALG00010022113	0.282145715	0.223348484
ENSGALG00010022114	0.57647334	0.080690695
ENSGALG00010022115	0.323773569	0.131260756
ENSGALG00010022116	0.979001005	0.306596754
ENSGALG00010022117	0.915573627	0.422478829
ENSGALG00010022118	0.119471243	0.208742735
ENSGALG00010022119	0.814868148	0.340697366
ENSGALG00010022120	0.193167071	0.283934491
ENSGALG00010022121	0.383549736	0.180028334
ENSGALG00010022122	0.910462102	0.395799779
ENSGALG00010022124	0.12980523	0.009072357
ENSGALG00010022125	0.976724702	0.288114634
ENSGALG00010022128	0.956924396	0.336137348
ENSGALG00010022129	0.969426712	0.389166755
ENSGALG00010022130	0.364639449	0.209777265
ENSGALG00010022134	0.388183575	0.081314947
ENSGALG00010022135	0.912258499	0.348912552
ENSGALG00010022136	0.498082113	0.194392333
ENSGALG00010022137	0.921264672	0.242399863
ENSGALG00010022138	0.866789602	0.275090368
ENSGALG00010022141	0.939077175	0.308352302
ENSGALG00010022142	0.812939146	0.152477614
ENSGALG00010022143	0.704363463	0.392192549
ENSGALG00010022144	0.992450987	0.336703761
ENSGALG00010022145	0.990777323	0.328949948
ENSGALG00010022146	0.268686642	0.236683914
ENSGALG00010022148	0.750982319	0.210067972
ENSGALG00010022149	0.961756573	0.31835012
ENSGALG00010022150	0.244264081	0.308326511
ENSGALG00010022151	0.976999606	0.326424257
ENSGALG00010022152	0.970854051	0.386461739
ENSGALG00010022154	0.82978689	0.449537554
ENSGALG00010022157	0.974223162	0.280181042
ENSGALG00010022158	0.590378303	0.12785401
ENSGALG00010022159	0.889875256	0.337190504
ENSGALG00010022160	0.374225086	0.079757343

ENSGALG00010022161	0.891681801	0.245617493
ENSGALG00010022162	0.651247568	0.106190977
ENSGALG00010022164	0.982851581	0.389303752
ENSGALG00010022165	0.975497701	0.36498611
ENSGALG00010022166	0.910285541	0.254898273
ENSGALG00010022167	0.544254101	0.265682684
ENSGALG00010022168	0.445181481	0.314904609
ENSGALG00010022169	0.698488616	0.045566584
ENSGALG00010022170	0.906636295	0.391298239
ENSGALG00010022171	0.947462007	0.225669115
ENSGALG00010022172	0.872084724	0.302872438
ENSGALG00010022173	0.943509219	0.305546125
ENSGALG00010022174	0.912449401	0.3630775
ENSGALG00010022175	0.645847651	0.662281891
ENSGALG00010022176	0.370139014	0.175677182
ENSGALG00010022178	0.686617267	0.397622637
ENSGALG00010022179	0.022131436	0.198334219
ENSGALG00010022180	0.256514663	0.19972267
ENSGALG00010022181	0.916020408	0.399646947
ENSGALG00010022182	0.873926789	0.402728127
ENSGALG00010022184	0.89132025	0.402214485
ENSGALG00010022186	0.936016232	0.208757459
ENSGALG00010022187	0.189890692	0.196551403
ENSGALG00010022188	0.266137114	0.107784983
ENSGALG00010022189	0.040734307	0.154016134
ENSGALG00010022190	0.932231774	0.277932649
ENSGALG00010022192	0.270451009	0.090541667
ENSGALG00010022193	0.218099082	0.350254781
ENSGALG00010022194	0.923344905	0.34951138
ENSGALG00010022195	0.240472544	0.101172713
ENSGALG00010022196	0.357586709	0.230180831
ENSGALG00010022199	0.969977587	0.364279221
ENSGALG00010022200	0.816782271	0.162086177
ENSGALG00010022202	0.865615506	0.400024214
ENSGALG00010022203	0.616869791	0.066218335
ENSGALG00010022204	0.574143528	0.030424516
ENSGALG00010022205	0.975577605	0.317218936
ENSGALG00010022206	0.719646486	0.525206411
ENSGALG00010022207	0.955333452	0.415418001
ENSGALG00010022208	0.269707025	0.220966775
ENSGALG00010022209	0.101705585	0.178318792
ENSGALG00010022210	0.750866006	0.209140347
ENSGALG00010022211	0.991665226	0.363429962
ENSGALG00010022213	0.969475469	0.327834894
ENSGALG00010022214	0.934750059	0.171439537
ENSGALG00010022215	0.210332825	0.16260767
ENSGALG00010022216	0.933860799	0.134617048
ENSGALG00010022217	0.853457129	0.132133387
ENSGALG00010022218	0.985223851	0.338866267
ENSGALG00010022219	0.622707764	0.181163539
ENSGALG00010022220	0.727382172	0.446879856
ENSGALG00010022221	0.75139707	0.418974503
ENSGALG00010022222	0.927075057	0.325877254
ENSGALG00010022223	0.682661686	0.072157326
ENSGALG00010022224	0.222597248	0.14781795
ENSGALG00010022225	0.601829022	0.025433818

ENSGALG00010022226	0.769521454	0.307867885
ENSGALG00010022227	0.625613846	0.334109761
ENSGALG00010022228	0.686516312	0.430297589
ENSGALG00010022229	0.985193668	0.315766804
ENSGALG00010022230	0.984181289	0.344484149
ENSGALG00010022231	0.924408321	0.368044832
ENSGALG00010022232	0.456071243	0.425928717
ENSGALG00010022233	0.975645258	0.361439219
ENSGALG00010022234	0.859490614	0.444155133
ENSGALG00010022235	0.913521919	0.39318058
ENSGALG00010022236	0.995785403	0.327076585
ENSGALG00010022237	0.707975938	0.483318757
ENSGALG00010022238	0.974916981	0.33309875
ENSGALG00010022239	0.656969114	0.115316856
ENSGALG00010022240	0.964092926	0.367508328
ENSGALG00010022241	0.975038082	0.314224807
ENSGALG00010022242	0.979496099	0.318107374
ENSGALG00010022243	0.966586631	0.315864929
ENSGALG00010022244	0.889932018	0.146108002
ENSGALG00010022245	0.777334018	0.357797189
ENSGALG00010022246	0.144987931	0.283241891
ENSGALG00010022247	0.641543723	0.150422361
ENSGALG00010022248	0.400007934	0.244327522
ENSGALG00010022249	0.658672581	0.363363708
ENSGALG00010022250	0.951423022	0.31892453
ENSGALG00010022251	0.005910663	0.01624284
ENSGALG00010022252	0.978903216	0.317268271
ENSGALG00010022253	0.98821483	0.30958834
ENSGALG00010022255	0.957271831	0.265699146
ENSGALG00010022256	0.974415458	0.283964986
ENSGALG00010022257	0.396668506	0.181671381
ENSGALG00010022258	0.725856871	0.003790952
ENSGALG00010022259	0.919252004	0.31579109
ENSGALG00010022260	0.241258347	0.184127043
ENSGALG00010022261	0.669916022	0.207587907
ENSGALG00010022264	0.739234101	0.325448794
ENSGALG00010022266	0.776835318	0.50506669
ENSGALG00010022267	0.862667774	0.17760325
ENSGALG00010022269	0.041614593	0.239061706
ENSGALG00010022270	0.947771168	0.31073342
ENSGALG00010022271	0.713073607	0.654507716
ENSGALG00010022273	0.819482721	0.312633297
ENSGALG00010022274	0.189364163	0.181211771
ENSGALG00010022275	0.97417542	0.294326463
ENSGALG00010022276	0.88116101	0.197292539
ENSGALG00010022277	0.145445488	0.107454263
ENSGALG00010022278	0.93616591	0.357633628
ENSGALG00010022279	0.744246465	0.380948149
ENSGALG00010022280	0.969672065	0.265997283
ENSGALG00010022281	0.737348247	0.239326458
ENSGALG00010022282	0.890209248	0.376789707
ENSGALG00010022284	0.879744983	0.323525604
ENSGALG00010022287	0.939642238	0.333895175
ENSGALG00010022290	0.990005169	0.318012793
ENSGALG00010022293	0.748050298	0.113186454
ENSGALG00010022294	0.521438226	0.269844322

ENSGALG00010022296	0.923667192	0.310577614
ENSGALG00010022297	0.520391138	0.289523629
ENSGALG00010022299	0.633258398	0.305677408
ENSGALG00010022301	0.339323922	0.227455498
ENSGALG00010022302	0.980186261	0.335393202
ENSGALG00010022303	0.994460472	0.296011847
ENSGALG00010022304	0.953539299	0.391777045
ENSGALG00010022305	0.40135901	0.158038591
ENSGALG00010022306	0.988857596	0.313837384
ENSGALG00010022307	0.990687384	0.299210477
ENSGALG00010022308	0.84365877	0.262839276
ENSGALG00010022309	0.635961725	0.269284966
ENSGALG00010022310	0.779987134	0.013138033
ENSGALG00010022311	0.855461004	0.267353434
ENSGALG00010022312	0.696021308	0.379880454
ENSGALG00010022313	0.391378541	0.014984304
ENSGALG00010022314	0.90990876	0.286228272
ENSGALG00010022315	0.947276802	0.298484905
ENSGALG00010022316	0.907972356	0.216102883
ENSGALG00010022317	0.643108019	0.051846046
ENSGALG00010022318	0.455456485	0.121318594
ENSGALG00010022319	0.39198709	0.383967097
ENSGALG00010022320	0.792541607	0.361651275
ENSGALG00010022322	0.682988081	0.300277043
ENSGALG00010022323	0.992141266	0.32810647
ENSGALG00010022324	0.201433424	0.235000413
ENSGALG00010022325	0.8231569	0.560185112
ENSGALG00010022326	0.752776048	0.219777058
ENSGALG00010022327	0.798956077	0.105398444
ENSGALG00010022328	0.559126594	0.019511555
ENSGALG00010022329	0.886350224	0.355821577
ENSGALG00010022330	0.98038129	0.317351
ENSGALG00010022331	0.136216199	0.22288475
ENSGALG00010022332	0.593140392	0.033683697
ENSGALG00010022333	0.843405955	0.245082421
ENSGALG00010022334	0.555478268	0.360193454
ENSGALG00010022335	0.264239718	0.073427965
ENSGALG00010022338	0.39190877	0.131701825
ENSGALG00010022340	0.900116117	0.392833001
ENSGALG00010022341	0.113650382	0.043890876
ENSGALG00010022343	0.976586525	0.250552486
ENSGALG00010022345	0.99027851	0.3021248
ENSGALG00010022347	0.938645008	0.333330184
ENSGALG00010022348	0.993192524	0.335503881
ENSGALG00010022352	0.374974364	0.078620432
ENSGALG00010022353	0.949547088	0.310917945
ENSGALG00010022354	0.482590919	0.137131017
ENSGALG00010022355	0.350983868	0.128157198
ENSGALG00010022356	0.608552769	0.306019088
ENSGALG00010022357	0.978063827	0.327874841
ENSGALG00010022358	0.307242338	0.098889492
ENSGALG00010022359	0.842422519	0.209967835
ENSGALG00010022360	0.290122505	0.464993379
ENSGALG00010022361	0.622443595	0.469258101
ENSGALG00010022363	0.525823237	0.117324086
ENSGALG00010022365	0.223883554	0.140808323

ENSGALG00010022366	0.861870419	0.206520899
ENSGALG00010022368	0.38718504	0.101838932
ENSGALG00010022370	0.366356945	0.080496265
ENSGALG00010022373	0.345733183	0.771407729
ENSGALG00010022375	0.426397163	0.22810538
ENSGALG00010022377	0.8551441	0.279599837
ENSGALG00010022378	0.262922434	0.138601464
ENSGALG00010022379	0.540215559	0.073412036
ENSGALG00010022381	0.265558089	0.028821141
ENSGALG00010022383	0.337350312	0.486558046
ENSGALG00010022385	0.305262806	0.293907863
ENSGALG00010022386	0.981503687	0.308435213
ENSGALG00010022388	0.977847833	0.310338925
ENSGALG00010022391	0.008936839	0.090201086
ENSGALG00010022392	0.935254381	0.356759742
ENSGALG00010022393	0.192752914	0.375098429
ENSGALG00010022395	0.937766495	0.331582308
ENSGALG00010022396	0.034383068	0.018800754
ENSGALG00010022397	0.895932097	0.178714455
ENSGALG00010022400	0.66580992	0.367788904
ENSGALG00010022401	0.98351587	0.28754461
ENSGALG00010022403	0.277566769	0.107857127
ENSGALG00010022404	0.379394869	0.170237467
ENSGALG00010022406	0.975630584	0.284537412
ENSGALG00010022407	0.967336612	0.28822511
ENSGALG00010022408	0.38321826	0.113113547
ENSGALG00010022409	0.992792049	0.342916413
ENSGALG00010022411	0.820053593	0.152724833
ENSGALG00010022412	0.300848163	0.101461559
ENSGALG00010022414	0.650496204	0.129956994
ENSGALG00010022415	0.978332848	0.393260053
ENSGALG00010022416	0.956199455	0.303667957
ENSGALG00010022417	0.142182469	0.149983465
ENSGALG00010022418	0.328351802	0.128015076
ENSGALG00010022419	0.406706929	0.075613654
ENSGALG00010022420	0.07133679	0.180876351
ENSGALG00010022421	0.885647585	0.079245339
ENSGALG00010022422	0.980668122	0.350374982
ENSGALG00010022423	0.422279618	0.252990097
ENSGALG00010022425	0.073389205	0.182303476
ENSGALG00010022426	0.452220863	0.225003423
ENSGALG00010022427	0.827283769	0.282153368
ENSGALG00010022428	0.395556858	0.102965232
ENSGALG00010022429	0.978777865	0.320702697
ENSGALG00010022430	0.168643922	0.317071488
ENSGALG00010022431	0.977315388	0.284166418
ENSGALG00010022433	0.980457876	0.332682399
ENSGALG00010022435	0.938304325	0.394290287
ENSGALG00010022436	0.142280769	0.281419068
ENSGALG00010022437	0.347664293	0.093632472
ENSGALG00010022438	0.936279662	0.233944789
ENSGALG00010022439	0.833878649	0.348157006
ENSGALG00010022440	0.927896242	0.239680879
ENSGALG00010022441	0.828274891	0.148049572
ENSGALG00010022443	0.08188551	0.154726792
ENSGALG00010022444	0.485424642	0.161671104

ENSGALG00010022445	0.059539862	0.180376229
ENSGALG00010022446	0.385118354	0.132533137
ENSGALG00010022448	0.53022471	0.001125904
ENSGALG00010022449	0.307896896	0.108693604
ENSGALG00010022450	0.848358054	0.158647341
ENSGALG00010022451	0.942871909	0.213283369
ENSGALG00010022452	0.541129039	0.160298772
ENSGALG00010022453	0.8980627	0.311071574
ENSGALG00010022454	0.925093288	0.261855754
ENSGALG00010022455	0.847567089	0.101395633
ENSGALG00010022456	0.689741309	0.40865989
ENSGALG00010022458	0.019712204	0.418846563
ENSGALG00010022459	0.502122009	0.474299492
ENSGALG00010022460	0.956572573	0.266896607
ENSGALG00010022462	0.31126492	0.041654644
ENSGALG00010022463	0.827860901	0.35796764
ENSGALG00010022464	0.010994392	0.115336973
ENSGALG00010022465	0.583676413	0.228984121
ENSGALG00010022466	0.655526408	0.088702673
ENSGALG00010022467	0.922412961	0.291659367
ENSGALG00010022468	0.928633898	0.377676033
ENSGALG00010022469	0.478056432	0.184736379
ENSGALG00010022470	0.967012056	0.32730341
ENSGALG00010022471	0.404535974	0.076467456
ENSGALG00010022472	0.56002463	0.07447504
ENSGALG00010022473	0.654803775	0.151785787
ENSGALG00010022474	0.958905457	0.329981431
ENSGALG00010022476	0.570776324	0.198716506
ENSGALG00010022477	0.210604799	0.300022113
ENSGALG00010022478	0.975342478	0.295407245
ENSGALG00010022479	0.984503379	0.376082186
ENSGALG00010022480	0.655082018	0.213033851
ENSGALG00010022481	0.135038205	0.205620754
ENSGALG00010022482	0.273936874	0.321919722
ENSGALG00010022483	0.745687445	0.058754426
ENSGALG00010022484	0.184616839	0.16415848
ENSGALG00010022485	0.920510412	0.271233252
ENSGALG00010022486	0.22235895	0.005531054
ENSGALG00010022488	0.164478933	0.270582683
ENSGALG00010022489	0.507545279	0.021767074
ENSGALG00010022490	0.113459865	0.170691905
ENSGALG00010022493	0.936043751	0.249831672
ENSGALG00010022494	0.702206141	0.290033586
ENSGALG00010022495	0.923713824	0.376707
ENSGALG00010022496	0.458020087	0.084885561
ENSGALG00010022497	0.917051739	0.184859597
ENSGALG00010022498	0.9880771	0.324012176
ENSGALG00010022499	0.687373871	0.348023241
ENSGALG00010022500	0.988577947	0.317079281
ENSGALG00010022501	0.901489209	0.32396067
ENSGALG00010022502	0.953231149	0.291390236
ENSGALG00010022503	0.853777074	0.031268625
ENSGALG00010022504	0.981546128	0.317284612
ENSGALG00010022505	0.848509651	0.126777931
ENSGALG00010022506	0.670138558	0.036255664
ENSGALG00010022507	0.929368703	0.188160989

ENSGALG00010022508	0.909180865	0.336003339
ENSGALG00010022509	0.938503794	0.321720263
ENSGALG00010022511	0.960289225	0.260730849
ENSGALG00010022512	0.779671013	0.613615532
ENSGALG00010022513	0.863099666	0.240933826
ENSGALG00010022514	0.425296556	0.09607093
ENSGALG00010022515	0.951105521	0.289006592
ENSGALG00010022516	0.952476784	0.270465052
ENSGALG00010022517	0.927256386	0.263611577
ENSGALG00010022518	0.88039188	0.394460462
ENSGALG00010022520	0.889162421	0.255789026
ENSGALG00010022521	0.955501308	0.294137462
ENSGALG00010022522	0.719779281	0.197783925
ENSGALG00010022524	0.7091124	0.386632071
ENSGALG00010022525	0.451788251	0.00193286
ENSGALG00010022526	0.988499471	0.322426319
ENSGALG00010022528	0.931917651	0.386301006
ENSGALG00010022529	0.918160739	0.277823211
ENSGALG00010022531	0.351039436	0.15590662
ENSGALG00010022532	0.386349697	0.164817078
ENSGALG00010022533	0.70768849	0.174592241
ENSGALG00010022534	0.40002651	0.04441826
ENSGALG00010022536	0.927652652	0.298270657
ENSGALG00010022538	0.610263678	0.078957811
ENSGALG00010022539	0.58554549	0.069036089
ENSGALG00010022540	0.966323632	0.364280761
ENSGALG00010022541	0.992416323	0.280156213
ENSGALG00010022544	0.189993433	0.116230849
ENSGALG00010022545	0.426654555	0.234652331
ENSGALG00010022546	0.786414822	0.246430523
ENSGALG00010022547	0.968966044	0.272224849
ENSGALG00010022548	0.948444701	0.322335938
ENSGALG00010022550	0.202166268	0.193360909
ENSGALG00010022551	0.841389821	0.028380112
ENSGALG00010022552	0.988945066	0.28461035
ENSGALG00010022553	0.910739974	0.199899321
ENSGALG00010022554	0.847306506	0.167054324
ENSGALG00010022556	0.286755814	0.501844446
ENSGALG00010022557	0.376640253	0.024581485
ENSGALG00010022558	0.852310422	0.435552022
ENSGALG00010022559	0.901604185	0.248474797
ENSGALG00010022560	0.162569977	0.295452318
ENSGALG00010022562	0.86863777	0.316526095
ENSGALG00010022563	0.271701261	0.072417625
ENSGALG00010022564	0.478592401	0.003490424
ENSGALG00010022565	0.834799771	0.216040025
ENSGALG00010022566	0.197588125	0.17325697
ENSGALG00010022567	0.523922383	0.359040943
ENSGALG00010022568	0.995688189	0.318311891
ENSGALG00010022569	0.442307023	0.454670983
ENSGALG00010022570	0.377008934	0.178767925
ENSGALG00010022571	0.806846233	0.416721723
ENSGALG00010022572	0.942818777	0.260292824
ENSGALG00010022573	0.312866467	0.233272305
ENSGALG00010022574	0.006609734	0.234178371
ENSGALG00010022575	0.976905959	0.353249505

ENSGALG00010022576	0.500450145	0.200577411
ENSGALG00010022577	0.050025814	0.364858299
ENSGALG00010022579	0.957731943	0.230336819
ENSGALG00010022581	0.985686551	0.314842774
ENSGALG00010022582	0.920728767	0.270328846
ENSGALG00010022583	0.834601401	0.239971691
ENSGALG00010022584	0.001135721	0.491064829
ENSGALG00010022585	0.685619368	0.030256125
ENSGALG00010022586	0.645549306	0.138289887
ENSGALG00010022587	0.2896197	0.603980439
ENSGALG00010022588	0.67412518	0.155681287
ENSGALG00010022590	0.983824584	0.33547154
ENSGALG00010022591	0.391097471	0.144336359
ENSGALG00010022592	0.835063097	0.332710727
ENSGALG00010022593	0.658848673	0.326645987
ENSGALG00010022594	0.011570714	0.087085695
ENSGALG00010022597	0.99079094	0.337147332
ENSGALG00010022598	0.406255908	0.242722818
ENSGALG00010022599	0.668744317	0.242016615
ENSGALG00010022600	0.736884248	0.084856184
ENSGALG00010022602	0.10463914	0.225992092
ENSGALG00010022603	0.37731953	0.44456732
ENSGALG00010022604	0.38620775	0.086736513
ENSGALG00010022606	0.612786315	0.531157742
ENSGALG00010022607	0.140628138	0.409068537
ENSGALG00010022608	0.616962232	0.395203675
ENSGALG00010022609	0.956286694	0.319447851
ENSGALG00010022611	0.492688894	0.725556925
ENSGALG00010022613	0.554520902	0.253830733
ENSGALG00010022614	0.97999837	0.277800953
ENSGALG00010022615	0.79641214	0.00152185
ENSGALG00010022616	0.554250638	0.270919913
ENSGALG00010022618	0.228720766	0.143882467
ENSGALG00010022619	0.731761422	0.314064931
ENSGALG00010022621	0.627875824	0.334555094
ENSGALG00010022622	0.943623966	0.293497477
ENSGALG00010022623	0.508863299	0.142414474
ENSGALG00010022624	0.572250264	0.327529188
ENSGALG00010022625	0.808129734	0.191896482
ENSGALG00010022626	0.951491834	0.326409533
ENSGALG00010022627	0.935716839	0.301051888
ENSGALG00010022628	0.064069694	0.430763997
ENSGALG00010022629	0.705614295	0.363499417
ENSGALG00010022630	0.099344815	0.178605837
ENSGALG00010022633	0.784367921	0.200063566
ENSGALG00010022634	0.262922434	0.138601464
ENSGALG00010022635	0.863775532	0.209987403
ENSGALG00010022636	0.816184737	0.23538873
ENSGALG00010022637	0.152010125	0.213116426
ENSGALG00010022638	0.858489817	0.178189251
ENSGALG00010022640	0.984075722	0.287510856
ENSGALG00010022641	0.024374485	0.442743073
ENSGALG00010022642	0.085042597	0.005420494
ENSGALG00010022643	0.550387836	0.295940071
ENSGALG00010022644	0.916020042	0.32947108
ENSGALG00010022645	0.980019863	0.292781205

ENSGALG00010022646	0.770420494	0.283408262
ENSGALG00010022647	0.960032622	0.336658419
ENSGALG00010022648	0.162241107	0.114782335
ENSGALG00010022649	0.07382069	0.083668104
ENSGALG00010022650	0.932889778	0.320445998
ENSGALG00010022651	0.228336748	0.204985534
ENSGALG00010022652	0.878161852	0.230334602
ENSGALG00010022653	0.644192458	0.293296367
ENSGALG00010022654	0.605893084	0.266429251
ENSGALG00010022655	0.101561507	0.144081659
ENSGALG00010022656	0.558161394	0.408938051
ENSGALG00010022657	0.600007988	0.033868286
ENSGALG00010022658	0.51433486	0.101818046
ENSGALG00010022659	0.649129838	0.325302828
ENSGALG00010022660	0.120522951	0.143700346
ENSGALG00010022661	0.065890588	0.321687627
ENSGALG00010022662	0.963347771	0.292450669
ENSGALG00010022664	0.673707329	0.076390389
ENSGALG00010022665	0.376801121	0.142341445
ENSGALG00010022666	0.418200955	0.203620139
ENSGALG00010022667	0.437658353	0.382782334
ENSGALG00010022669	0.175232246	0.381900686
ENSGALG00010022670	0.286555462	0.078819245
ENSGALG00010022671	0.825686895	0.323187805
ENSGALG00010022672	0.307860476	0.247884083
ENSGALG00010022673	0.037101857	0.023625526
ENSGALG00010022674	0.79080706	0.288735829
ENSGALG00010022676	0.985882586	0.304904522
ENSGALG00010022677	0.971787249	0.360488343
ENSGALG00010022678	0.955881917	0.388358249
ENSGALG00010022680	0.632859768	0.159253507
ENSGALG00010022681	0.977175	0.282893536
ENSGALG00010022682	0.989132295	0.323748422
ENSGALG00010022683	0.339836176	0.257377495
ENSGALG00010022684	0.705930976	0.023783618
ENSGALG00010022685	0.64805876	0.237489803
ENSGALG00010022689	0.545577486	0.625173244
ENSGALG00010022690	0.719880199	0.105123037
ENSGALG00010022691	0.615222744	0.099648965
ENSGALG00010022695	0.949040859	0.248641144
ENSGALG00010022696	0.285272007	0.109169955
ENSGALG00010022697	0.770568187	0.274515199
ENSGALG00010022698	0.927281796	0.298883277
ENSGALG00010022699	0.820346378	0.103809371
ENSGALG00010022700	0.32829454	0.451866978
ENSGALG00010022702	0.521377009	0.102311913
ENSGALG00010022703	0.258481337	0.146910782
ENSGALG00010022704	0.361219176	0.129630793
ENSGALG00010022705	0.993912305	0.329764736
ENSGALG00010022707	0.9940005	0.316948711
ENSGALG00010022708	0.916107412	0.243282232
ENSGALG00010022709	0.941115813	0.262372277
ENSGALG00010022710	0.161858822	0.266359545
ENSGALG00010022711	0.489665598	0.448707682
ENSGALG00010022712	0.885443303	0.322134098
ENSGALG00010022715	0.875679375	0.353323541

ENSGALG00010022717	0.924963095	0.095438156
ENSGALG00010022718	0.892383117	0.315437859
ENSGALG00010022719	0.154391753	0.075137614
ENSGALG00010022722	0.189240572	0.04996112
ENSGALG00010022723	0.468260787	0.141962844
ENSGALG00010022724	0.264135059	0.122428802
ENSGALG00010022725	0.08908866	0.183956964
ENSGALG00010022726	0.799144707	0.262800645
ENSGALG00010022727	0.969087508	0.336424906
ENSGALG00010022728	0.54655015	0.214551661
ENSGALG00010022731	0.944224316	0.273684569
ENSGALG00010022732	0.542827985	0.351003608
ENSGALG00010022733	0.926552568	0.404825858
ENSGALG00010022734	0.956221411	0.348088537
ENSGALG00010022735	0.666171994	0.176556353
ENSGALG00010022736	0.286799024	0.210879307
ENSGALG00010022737	0.819304669	0.382205126
ENSGALG00010022738	0.598002198	0.426835949
ENSGALG00010022739	0.465513101	0.118017327
ENSGALG00010022740	0.815375053	0.115467016
ENSGALG00010022741	0.969977782	0.259822799
ENSGALG00010022742	0.139877562	0.35493793
ENSGALG00010022743	0.868794912	0.48200002
ENSGALG00010022745	0.976263215	0.359720692
ENSGALG00010022746	0.710551534	0.156573694
ENSGALG00010022747	0.900508102	0.327246939
ENSGALG00010022748	0.549059156	0.633864384
ENSGALG00010022749	0.659181032	0.39575394
ENSGALG00010022751	0.98712403	0.359426282
ENSGALG00010022754	0.552821432	0.350880231
ENSGALG00010022755	0.936187615	0.356813624
ENSGALG00010022756	0.102845326	0.156156484
ENSGALG00010022759	0.89631143	0.208706726
ENSGALG00010022760	0.106320184	0.296519917
ENSGALG00010022761	0.764570267	0.15188003
ENSGALG00010022763	0.252191774	0.795988502
ENSGALG00010022764	0.951421753	0.394879997
ENSGALG00010022765	0.044037286	0.232480103
ENSGALG00010022766	0.502531638	0.246552155
ENSGALG00010022767	0.248066728	0.116800122
ENSGALG00010022768	0.562295553	0.165128148
ENSGALG00010022769	0.990521837	0.363099832
ENSGALG00010022770	0.557692005	0.281513255
ENSGALG00010022771	0.910248994	0.325126813
ENSGALG00010022772	0.477671531	0.320145305
ENSGALG00010022774	0.997640949	0.344707343
ENSGALG00010022776	0.947427651	0.30974598
ENSGALG00010022778	0.865425903	0.110318597
ENSGALG00010022780	0.21633306	0.098502548
ENSGALG00010022781	0.845146134	0.385931265
ENSGALG00010022782	0.895120865	0.456461942
ENSGALG00010022783	0.911092143	0.144260996
ENSGALG00010022784	0.489856264	0.121819625
ENSGALG00010022785	0.910495624	0.238723728
ENSGALG00010022786	0.808133056	0.235946414
ENSGALG00010022787	0.927851046	0.43841685

ENSGALG00010022788	0.840668245	0.40675518
ENSGALG00010022789	0.962991607	0.27512788
ENSGALG00010022790	0.887981856	0.537412741
ENSGALG00010022791	0.924703174	0.384970727
ENSGALG00010022792	0.839959871	0.36487897
ENSGALG00010022793	0.498524812	0.23012935
ENSGALG00010022795	0.720556051	0.244475131
ENSGALG00010022796	0.385217368	0.300564124
ENSGALG00010022797	0.960399898	0.253363963
ENSGALG00010022798	0.664000664	0.16292465
ENSGALG00010022799	0.441674678	0.455774693
ENSGALG00010022800	0.848603515	0.101614898
ENSGALG00010022801	0.928442468	0.36383382
ENSGALG00010022802	0.546330823	0.237820109
ENSGALG00010022803	0.293723519	0.344691562
ENSGALG00010022804	0.822676349	0.027198901
ENSGALG00010022805	0.524257857	0.05415808
ENSGALG00010022806	0.984000803	0.327529753
ENSGALG00010022807	0.751169046	0.453153807
ENSGALG00010022808	0.87643212	0.262643037
ENSGALG00010022809	0.930442145	0.319082299
ENSGALG00010022810	0.716041583	0.272113302
ENSGALG00010022811	0.769864345	0.085126852
ENSGALG00010022812	0.851422106	0.315100004
ENSGALG00010022813	0.954415351	0.325096141
ENSGALG00010022814	0.238850225	0.196690181
ENSGALG00010022815	0.961523125	0.288224384
ENSGALG00010022816	0.828673843	0.121500824
ENSGALG00010022818	0.78066525	0.441886264
ENSGALG00010022819	0.990503483	0.332585955
ENSGALG00010022820	0.96615109	0.331147459
ENSGALG00010022821	0.995248824	0.336502161
ENSGALG00010022822	0.897523457	0.224781145
ENSGALG00010022823	0.965164985	0.210936558
ENSGALG00010022824	0.329947021	0.165781736
ENSGALG00010022825	0.960770428	0.262854353
ENSGALG00010022826	0.065172727	0.136778041
ENSGALG00010022827	0.884532006	0.274608053
ENSGALG00010022828	0.857460989	0.220510083
ENSGALG00010022829	0.80696137	0.284452361
ENSGALG00010022830	0.855123196	0.137419664
ENSGALG00010022831	0.447114012	0.116515789
ENSGALG00010022832	0.559338046	0.125826849
ENSGALG00010022833	0.113553107	0.333743478
ENSGALG00010022834	0.114924896	0.173020819
ENSGALG00010022836	0.530101512	0.168787218
ENSGALG00010022837	0.911330112	0.426700042
ENSGALG00010022838	0.955616647	0.29499065
ENSGALG00010022839	0.891825767	0.28961038
ENSGALG00010022840	0.165085328	0.579650452
ENSGALG00010022841	0.100267692	0.330310359
ENSGALG00010022842	0.83125309	0.187033182
ENSGALG00010022843	0.110041974	0.123910509
ENSGALG00010022844	0.027851816	0.14441112
ENSGALG00010022845	0.743369985	0.289091656
ENSGALG00010022846	0.000893823	0.500599188

ENSGALG00010022847	0.663039987	0.223659035
ENSGALG00010022848	0.216433634	0.680598293
ENSGALG00010022849	0.315941415	0.593915018
ENSGALG00010022850	0.592895454	0.053303305
ENSGALG00010022851	0.42868344	0.64194763
ENSGALG00010022852	0.764314347	0.293375178
ENSGALG00010022853	0.774460409	0.059717664
ENSGALG00010022856	0.464117993	0.328429109
ENSGALG00010022857	0.251531739	0.178153052
ENSGALG00010022858	0.744049284	0.312095508
ENSGALG00010022860	0.972281984	0.240731634
ENSGALG00010022861	0.730999346	0.226024257
ENSGALG00010022862	0.925255021	0.350869714
ENSGALG00010022863	0.190288411	0.37006115
ENSGALG00010022864	0.748459996	0.430924261
ENSGALG00010022865	0.277566769	0.107857127
ENSGALG00010022866	0.935529822	0.209548244
ENSGALG00010022867	0.974513851	0.336288544
ENSGALG00010022868	0.730590263	0.305336176
ENSGALG00010022869	0.751879992	0.188296041
ENSGALG00010022870	0.160411737	0.150137187
ENSGALG00010022871	0.350206248	0.230703012
ENSGALG00010022872	0.058419119	0.122260959
ENSGALG00010022873	0.810948797	0.303597336
ENSGALG00010022874	0.881269443	0.318546022
ENSGALG00010022875	0.947553952	0.424016481
ENSGALG00010022877	0.154564568	0.119876687
ENSGALG00010022878	0.257495438	0.124672868
ENSGALG00010022879	0.926731747	0.161910162
ENSGALG00010022880	0.135694619	0.134705453
ENSGALG00010022881	0.143789094	0.019347315
ENSGALG00010022882	0.334090543	0.210043301
ENSGALG00010022883	0.617318004	0.210325894
ENSGALG00010022885	0.932822926	0.24641326
ENSGALG00010022886	0.899961482	0.275625496
ENSGALG00010022888	0.935099918	0.233379152
ENSGALG00010022890	0.984181301	0.322003482
ENSGALG00010022891	0.888227727	0.067410087
ENSGALG00010022892	0.265558089	0.028821141
ENSGALG00010022894	0.847284895	0.280944133
ENSGALG00010022895	0.681910899	0.424666953
ENSGALG00010022897	0.973111006	0.28087243
ENSGALG00010022899	0.685410856	0.385360621
ENSGALG00010022900	0.17234983	0.329145981
ENSGALG00010022903	0.382985366	0.1291087
ENSGALG00010022904	0.405745866	0.078539969
ENSGALG00010022905	0.242059507	0.208144847
ENSGALG00010022906	0.021553974	0.325011588
ENSGALG00010022907	0.516521745	0.209332593
ENSGALG00010022909	0.078939733	0.184522641
ENSGALG00010022911	0.852385123	0.038248204
ENSGALG00010022913	0.488195452	0.180499421
ENSGALG00010022914	0.962100015	0.380000246
ENSGALG00010022915	0.426196336	0.036601532
ENSGALG00010022917	0.987950128	0.296360984
ENSGALG00010022919	0.991093651	0.299915668

ENSGALG00010022920	0.014123847	0.097825401
ENSGALG00010022921	0.831083949	0.10267063
ENSGALG00010022922	0.405959509	0.278890396
ENSGALG00010022923	0.930099222	0.271197437
ENSGALG00010022924	0.02551123	0.177368566
ENSGALG00010022925	0.873692103	0.173407118
ENSGALG00010022926	0.264090193	0.140913682
ENSGALG00010022927	0.243173907	0.148349226
ENSGALG00010022928	0.403172961	0.028071491
ENSGALG00010022929	0.97882537	0.317442408
ENSGALG00010022930	0.868588812	0.414728686
ENSGALG00010022931	0.948250378	0.31983632
ENSGALG00010022932	0.847878553	0.267137625
ENSGALG00010022933	0.884402805	0.285939783
ENSGALG00010022934	0.382090837	0.062273071
ENSGALG00010022935	0.000893823	0.500599188
ENSGALG00010022937	0.981202009	0.302939197
ENSGALG00010022942	0.574276377	0.105585458
ENSGALG00010022943	0.882376361	0.311077058
ENSGALG00010022944	0.558258517	0.357680795
ENSGALG00010022946	0.069304212	0.158640267
ENSGALG00010022947	0.982017121	0.329204422
ENSGALG00010022948	0.954126398	0.192076646
ENSGALG00010022949	0.031090316	0.095543765
ENSGALG00010022950	0.992030283	0.339091229
ENSGALG00010022952	0.672739593	0.269276637
ENSGALG00010022954	0.828293852	0.365059301
ENSGALG00010022955	0.765961329	0.317310492
ENSGALG00010022956	0.924579462	0.256338846
ENSGALG00010022957	0.943485911	0.313111627
ENSGALG00010022958	0.708032723	0.354052075
ENSGALG00010022959	0.7361941	0.309912784
ENSGALG00010022960	0.267156317	0.118341176
ENSGALG00010022962	0.104359641	0.043511391
ENSGALG00010022963	0.502604739	0.366088542
ENSGALG00010022965	0.511694189	0.096925921
ENSGALG00010022966	0.760144912	0.352672876
ENSGALG00010022967	0.250346325	0.370439644
ENSGALG00010022968	0.934016877	0.420441106
ENSGALG00010022969	0.989412225	0.308928575
ENSGALG00010022970	0.853159157	0.250525971
ENSGALG00010022971	0.063182317	0.050201258
ENSGALG00010022972	0.99703904	0.335616701
ENSGALG00010022973	0.035949723	0.15676341
ENSGALG00010022974	0.987546712	0.3052732
ENSGALG00010022975	0.048720881	0.155114111
ENSGALG00010022976	0.947612716	0.304085931
ENSGALG00010022977	0.209217166	0.130147632
ENSGALG00010022978	0.88697272	0.163534452
ENSGALG00010022979	0.867925563	0.201341501
ENSGALG00010022980	0.379115405	0.206626367
ENSGALG00010022981	0.236262014	0.187731371
ENSGALG00010022982	0.633768134	0.056091135
ENSGALG00010022983	0.970297989	0.307004468
ENSGALG00010022985	0.803751455	0.29307783
ENSGALG00010022986	0.963674466	0.339912103

ENSGALG00010022987	0.996134126	0.35229465
ENSGALG00010022988	0.38715636	0.027422718
ENSGALG00010022989	0.586396573	0.313192369
ENSGALG00010022990	0.969272341	0.241942227
ENSGALG00010022991	0.679817527	0.283143945
ENSGALG00010022992	0.284804707	0.501661021
ENSGALG00010022994	0.966009855	0.285004772
ENSGALG00010022996	0.399453749	0.144668892
ENSGALG00010022997	0.771145419	0.112969534
ENSGALG00010022998	0.234092969	0.282062645
ENSGALG00010022999	0.633562388	0.260183361
ENSGALG00010023001	0.505520628	0.130024147
ENSGALG00010023002	0.983233595	0.298554463
ENSGALG00010023003	0.993786397	0.288774995
ENSGALG00010023004	0.830758813	0.129363179
ENSGALG00010023005	0.886537082	0.286554215
ENSGALG00010023006	0.501894671	0.375631667
ENSGALG00010023007	0.967845184	0.265468457
ENSGALG00010023008	0.973050334	0.332877116
ENSGALG00010023009	0.377293369	0.176429511
ENSGALG00010023010	0.897173838	0.368577219
ENSGALG00010023012	0.43516155	0.399347951
ENSGALG00010023013	0.97075444	0.367906333
ENSGALG00010023015	0.929961436	0.160674247
ENSGALG00010023016	0.567608652	0.180888036
ENSGALG00010023017	0.460764147	0.054732163
ENSGALG00010023019	0.317212487	0.04984452
ENSGALG00010023020	0.669646223	0.224942513
ENSGALG00010023022	0.6448968	0.220997327
ENSGALG00010023023	0.324712176	0.287838644
ENSGALG00010023024	0.705222179	0.074460881
ENSGALG00010023026	0.853450812	0.13353964
ENSGALG00010023028	0.587484876	0.33160754
ENSGALG00010023029	0.313161496	0.127746808
ENSGALG00010023030	0.510149741	0.380141913
ENSGALG00010023031	0.262922434	0.138601464
ENSGALG00010023032	0.050690267	0.14779477
ENSGALG00010023034	0.410231722	0.16984461
ENSGALG00010023037	0.384486165	0.786883991
ENSGALG00010023038	0.870419483	0.215462499
ENSGALG00010023039	0.765815699	0.272385841
ENSGALG00010023040	0.359142284	0.158091928
ENSGALG00010023041	0.875547378	0.367714153
ENSGALG00010023042	0.990465658	0.340939992
ENSGALG00010023043	0.546015045	0.161432397
ENSGALG00010023044	0.382985366	0.1291087
ENSGALG00010023045	0.097545881	0.09040332
ENSGALG00010023046	0.754224612	0.323903647
ENSGALG00010023047	0.445698713	0.048953051
ENSGALG00010023048	0.478461742	0.12713415
ENSGALG00010023049	0.405797923	0.13200104
ENSGALG00010023050	0.100021722	0.090739801
ENSGALG00010023051	0.016069541	0.511799972
ENSGALG00010023052	0.121652119	0.058852463
ENSGALG00010023053	0.91881428	0.318916476
ENSGALG00010023054	0.987840739	0.31215146

ENSGALG00010023055	0.689910534	0.193084995
ENSGALG00010023056	0.490183712	0.206853373
ENSGALG00010023057	0.927168375	0.26912026
ENSGALG00010023058	0.988042283	0.323504304
ENSGALG00010023059	0.915594048	0.327479951
ENSGALG00010023060	0.856014186	0.475589367
ENSGALG00010023061	0.457999501	0.151970763
ENSGALG00010023062	0.953454159	0.270501146
ENSGALG00010023063	0.864760862	0.328963826
ENSGALG00010023064	0.936750943	0.307343978
ENSGALG00010023065	0.571875688	0.277003544
ENSGALG00010023066	0.143712685	0.023649583
ENSGALG00010023067	0.869499105	0.180292377
ENSGALG00010023068	0.982631978	0.33436178
ENSGALG00010023069	0.844617201	0.559195369
ENSGALG00010023070	0.728562824	0.309577796
ENSGALG00010023071	0.97095833	0.283293287
ENSGALG00010023072	0.325016977	0.371468092
ENSGALG00010023073	0.938730167	0.406041841
ENSGALG00010023074	0.910014732	0.313242586
ENSGALG00010023075	0.905019372	0.224045547
ENSGALG00010023076	0.984310336	0.31996951
ENSGALG00010023077	0.648667983	0.149012522
ENSGALG00010023078	0.420176502	0.494228039
ENSGALG00010023079	0.912856018	0.25380346
ENSGALG00010023080	0.973826535	0.332760503
ENSGALG00010023081	0.182515023	0.10074783
ENSGALG00010023082	0.376189922	0.190265999
ENSGALG00010023083	0.735507098	0.273731394
ENSGALG00010023084	0.859231817	0.461898195
ENSGALG00010023085	0.647827407	0.262257697
ENSGALG00010023086	0.887662227	0.340877255
ENSGALG00010023087	0.976422655	0.264281325
ENSGALG00010023088	0.481124533	0.17287833
ENSGALG00010023089	0.640642962	0.31407038
ENSGALG00010023090	0.750014327	0.332852595
ENSGALG00010023091	0.345434828	0.004027976
ENSGALG00010023092	0.400215036	0.132215663
ENSGALG00010023093	0.968104858	0.306850843
ENSGALG00010023094	0.101237755	0.297312526
ENSGALG00010023096	0.743571613	0.25946076
ENSGALG00010023097	0.503970897	0.18304166
ENSGALG00010023098	0.067501704	0.182705483
ENSGALG00010023099	0.997306623	0.334927788
ENSGALG00010023100	0.070188579	0.042362924
ENSGALG00010023101	0.724296372	0.258894544
ENSGALG00010023102	0.972596569	0.279247755
ENSGALG00010023103	0.942878933	0.187695449
ENSGALG00010023104	0.790036305	0.094641306
ENSGALG00010023105	0.281014372	0.081680553
ENSGALG00010023107	0.975032331	0.340926706
ENSGALG00010023108	0.404363207	0.188164135
ENSGALG00010023110	0.887274242	0.210149726
ENSGALG00010023111	0.499911959	0.176896944
ENSGALG00010023112	0.904819865	0.293584671
ENSGALG00010023113	0.22764221	0.205349295

ENSGALG00010023114	0.734144595	0.34143054
ENSGALG00010023115	0.891870013	0.345999682
ENSGALG00010023116	0.987485876	0.272617888
ENSGALG00010023117	0.1351616	0.103731814
ENSGALG00010023118	0.933735121	0.294945134
ENSGALG00010023119	0.90352572	0.369280573
ENSGALG00010023120	0.737857447	0.049197863
ENSGALG00010023121	0.337471791	0.167003685
ENSGALG00010023122	0.938504964	0.497781085
ENSGALG00010023123	0.247758405	0.052279419
ENSGALG00010023124	0.845730195	0.320514826
ENSGALG00010023125	0.941967731	0.34752609
ENSGALG00010023126	0.991145379	0.361082603
ENSGALG00010023127	0.981882179	0.355662071
ENSGALG00010023128	0.954189568	0.219834361
ENSGALG00010023129	0.461237041	0.033132576
ENSGALG00010023130	0.432481777	0.121195283
ENSGALG00010023131	0.856023231	0.306472843
ENSGALG00010023133	0.821395863	0.233859945
ENSGALG00010023134	0.809221598	0.325325958
ENSGALG00010023135	0.637602955	0.078026723
ENSGALG00010023136	0.802851709	0.01402681
ENSGALG00010023137	0.709929539	0.300460954
ENSGALG00010023138	0.188353248	0.077257433
ENSGALG00010023140	0.229243984	0.5702469
ENSGALG00010023141	0.382985366	0.1291087
ENSGALG00010023142	0.951747326	0.197273623
ENSGALG00010023144	0.547638737	0.182346164
ENSGALG00010023146	0.487320017	0.195958317
ENSGALG00010023147	0.798463103	0.453316051
ENSGALG00010023149	0.861249531	0.379799006
ENSGALG00010023150	0.019460217	0.206595923
ENSGALG00010023151	0.984059808	0.261839823
ENSGALG00010023154	0.618748885	0.005036442
ENSGALG00010023155	0.164440723	0.366930101
ENSGALG00010023156	0.155440085	0.150790236
ENSGALG00010023157	0.136095582	0.231793088
ENSGALG00010023158	0.81789983	0.206514346
ENSGALG00010023159	0.526964233	0.030367953
ENSGALG00010023160	0.810587064	0.302729346
ENSGALG00010023161	0.970628196	0.235760197
ENSGALG00010023162	0.956471549	0.406270413
ENSGALG00010023163	0.922433937	0.400235816
ENSGALG00010023164	0.875354177	0.396950457
ENSGALG00010023165	0.385170559	0.033926908
ENSGALG00010023166	0.838076062	0.317041864
ENSGALG00010023167	0.983170926	0.335146733
ENSGALG00010023168	0.403148405	0.16396482
ENSGALG00010023169	0.752564641	0.323515551
ENSGALG00010023170	0.903845767	0.298005607
ENSGALG00010023171	0.48525293	0.171682166
ENSGALG00010023172	0.868076048	0.307331094
ENSGALG00010023173	0.386254376	0.161451439
ENSGALG00010023174	0.611692077	0.641461025
ENSGALG00010023175	0.867796282	0.233911309
ENSGALG00010023176	0.262922434	0.138601464

ENSGALG00010023177	0.987590751	0.318256909
ENSGALG00010023178	0.821085842	0.139276228
ENSGALG00010023179	0.253828841	0.024839892
ENSGALG00010023180	0.985164291	0.332848518
ENSGALG00010023181	0.939785969	0.21050929
ENSGALG00010023182	0.032259654	0.215395319
ENSGALG00010023183	0.309052815	0.071829493
ENSGALG00010023184	0.809971929	0.176062324
ENSGALG00010023185	0.98267692	0.307640967
ENSGALG00010023186	0.487041991	0.56453703
ENSGALG00010023187	0.981841718	0.308280349
ENSGALG00010023188	0.985239247	0.313356405
ENSGALG00010023189	0.906490101	0.431082113
ENSGALG00010023190	0.794795489	0.152341454
ENSGALG00010023192	0.917916327	0.326635439
ENSGALG00010023193	0.471658102	0.162683497
ENSGALG00010023194	0.933957659	0.403174609
ENSGALG00010023195	0.881788452	0.200261972
ENSGALG00010023196	0.92172164	0.338105214
ENSGALG00010023197	0.296272339	0.491563762
ENSGALG00010023198	0.03368789	0.250671139
ENSGALG00010023199	0.763272431	0.264760019
ENSGALG00010023200	0.909276694	0.231550806
ENSGALG00010023201	0.822583947	0.445606513
ENSGALG00010023202	0.673536865	0.21137413
ENSGALG00010023203	0.741929936	0.313784533
ENSGALG00010023204	0.847603135	0.259536577
ENSGALG00010023205	0.936966987	0.43663659
ENSGALG00010023206	0.973464527	0.290724622
ENSGALG00010023207	0.118088859	0.104285473
ENSGALG00010023208	0.733905471	0.016255872
ENSGALG00010023209	0.922665036	0.296487336
ENSGALG00010023210	0.062094683	0.462362884
ENSGALG00010023211	0.782709407	0.321603283
ENSGALG00010023212	0.809468578	0.212410309
ENSGALG00010023213	0.42628256	0.091407402
ENSGALG00010023214	0.699105174	0.150613073
ENSGALG00010023215	0.788728557	0.140847318
ENSGALG00010023216	0.649228365	0.356810619
ENSGALG00010023217	0.815623434	0.300234746
ENSGALG00010023218	0.488837505	0.083304764
ENSGALG00010023219	0.543451949	0.189310854
ENSGALG00010023220	0.959980385	0.352121144
ENSGALG00010023221	0.011728427	0.130036293
ENSGALG00010023222	0.294226904	0.099361029
ENSGALG00010023223	0.837330329	0.083032571
ENSGALG00010023225	0.933496361	0.267250527
ENSGALG00010023226	0.30962043	0.189081312
ENSGALG00010023227	0.758545313	0.266281797
ENSGALG00010023228	0.987471546	0.347384235
ENSGALG00010023229	0.791196219	0.403638833
ENSGALG00010023230	0.984006485	0.290870829
ENSGALG00010023231	0.109965949	0.024893637
ENSGALG00010023233	0.338700547	0.042090119
ENSGALG00010023234	0.332337017	0.163881835
ENSGALG00010023235	0.491517784	0.008894752

ENSGALG00010023237	0.229147801	0.161026159
ENSGALG00010023238	0.933511161	0.280251497
ENSGALG00010023239	0.859594947	0.305921088
ENSGALG00010023240	0.59055418	0.399522111
ENSGALG00010023243	0.994905249	0.320985601
ENSGALG00010023244	0.874804104	0.211854548
ENSGALG00010023245	0.82799123	0.338838473
ENSGALG00010023246	0.483944261	0.636547299
ENSGALG00010023247	0.322549733	0.226920139
ENSGALG00010023248	0.568863479	0.294182265
ENSGALG00010023249	0.716575912	0.059566683
ENSGALG00010023250	0.448343057	0.088243838
ENSGALG00010023251	0.934711206	0.268525987
ENSGALG00010023252	0.909929695	0.209293983
ENSGALG00010023253	0.519811601	0.19993089
ENSGALG00010023254	0.308445237	0.068934387
ENSGALG00010023255	0.95258963	0.376144864
ENSGALG00010023257	0.226482362	0.246608449
ENSGALG00010023258	0.02160797	0.114437157
ENSGALG00010023259	0.955540337	0.30694989
ENSGALG00010023260	0.058139656	0.005230107
ENSGALG00010023261	0.8929486	0.238507142
ENSGALG00010023262	0.875994928	0.275375397
ENSGALG00010023263	0.891196596	0.468198339
ENSGALG00010023264	0.436204244	0.010604866
ENSGALG00010023265	0.848666304	0.154117116
ENSGALG00010023266	0.599714871	0.293889639
ENSGALG00010023267	0.307328828	0.270960138
ENSGALG00010023268	0.989616943	0.325949437
ENSGALG00010023269	0.572361302	0.11410364
ENSGALG00010023270	0.739920173	0.460725995
ENSGALG00010023271	0.763942469	0.357634107
ENSGALG00010023272	0.816253193	0.311188694
ENSGALG00010023273	0.944031088	0.293436827
ENSGALG00010023274	0.794534486	0.261127968
ENSGALG00010023276	0.831254586	0.230977515
ENSGALG00010023277	0.627419073	0.383779683
ENSGALG00010023278	0.351196515	0.083424408
ENSGALG00010023279	0.975097706	0.320298153
ENSGALG00010023280	0.147559824	0.053397793
ENSGALG00010023281	0.608421081	0.490316239
ENSGALG00010023282	0.912569474	0.327142053
ENSGALG00010023283	0.508622295	0.266078524
ENSGALG00010023284	0.839781419	0.406309304
ENSGALG00010023285	0.953976319	0.316211098
ENSGALG00010023286	0.552977899	0.062822788
ENSGALG00010023287	0.819222182	0.341572257
ENSGALG00010023288	0.902921127	0.2524466
ENSGALG00010023289	0.300972266	0.114787999
ENSGALG00010023290	0.048815315	0.369445105
ENSGALG00010023291	0.848463669	0.184702106
ENSGALG00010023292	0.456865547	0.27132674
ENSGALG00010023293	0.123854023	0.012507325
ENSGALG00010023294	0.285272007	0.109169955
ENSGALG00010023295	0.441798016	0.818179307
ENSGALG00010023296	0.651984719	0.125581153

ENSGALG00010023297	0.501684793	0.152725632
ENSGALG00010023298	0.00291881	0.189464508
ENSGALG00010023299	0.483145695	0.089065759
ENSGALG00010023302	0.757125527	0.055425501
ENSGALG00010023304	0.601921454	0.050374347
ENSGALG00010023305	0.909231715	0.230531608
ENSGALG00010023307	0.470122212	0.141233929
ENSGALG00010023309	0.308967521	0.10461628
ENSGALG00010023311	0.870611655	0.141893694
ENSGALG00010023312	0.986040795	0.295726849
ENSGALG00010023314	0.837330459	0.299852571
ENSGALG00010023315	0.733010074	0.109306149
ENSGALG00010023316	0.256828349	0.119064955
ENSGALG00010023317	0.825224335	0.352312509
ENSGALG00010023319	0.89939368	0.392122281
ENSGALG00010023320	0.961038511	0.33979238
ENSGALG00010023322	0.094352618	0.242242076
ENSGALG00010023324	0.892911824	0.296198255
ENSGALG00010023325	0.376808449	0.786806161
ENSGALG00010023326	0.310945388	0.732980472
ENSGALG00010023327	0.190585904	0.011950567
ENSGALG00010023328	0.821232634	0.234675989
ENSGALG00010023329	0.876462347	0.316931848
ENSGALG00010023330	0.986803757	0.332718071
ENSGALG00010023331	0.856009647	0.050708652
ENSGALG00010023332	0.988648206	0.341557207
ENSGALG00010023333	0.413850864	0.68252835
ENSGALG00010023334	0.875225721	0.288598389
ENSGALG00010023335	0.012811482	0.078178341
ENSGALG00010023336	0.247660946	0.300978107
ENSGALG00010023337	0.932281696	0.198096842
ENSGALG00010023338	0.124705648	0.149092768
ENSGALG00010023339	0.450637699	0.70414841
ENSGALG00010023340	0.860269702	0.09439476
ENSGALG00010023341	0.177454464	0.008894243
ENSGALG00010023342	0.067802737	0.115040004
ENSGALG00010023343	0.993398513	0.285154482
ENSGALG00010023344	0.105327499	0.095352743
ENSGALG00010023345	0.184202368	0.190569538
ENSGALG00010023346	0.584277259	0.19960296
ENSGALG00010023347	0.984520238	0.298486715
ENSGALG00010023349	0.507366828	0.176993171
ENSGALG00010023350	0.994924892	0.357401436
ENSGALG00010023351	0.79040185	0.128181724
ENSGALG00010023352	0.059891944	0.301840631
ENSGALG00010023353	0.649777823	0.21868166
ENSGALG00010023354	0.973834725	0.287997084
ENSGALG00010023355	0.71541942	0.571922382
ENSGALG00010023356	0.756305648	0.474033545
ENSGALG00010023359	0.129917853	0.363354375
ENSGALG00010023362	0.986857638	0.347203668
ENSGALG00010023363	0.206973096	0.396378723
ENSGALG00010023364	0.049695279	0.1582619
ENSGALG00010023365	0.845322321	0.268748751
ENSGALG00010023366	0.535861882	0.165813128
ENSGALG00010023368	0.476378483	0.249613167

ENSGALG00010023369	0.974868042	0.344720888
ENSGALG00010023371	0.203991683	0.058221925
ENSGALG00010023372	0.50200739	0.086492125
ENSGALG00010023373	0.304953602	0.087447627
ENSGALG00010023374	0.735621418	0.35289718
ENSGALG00010023375	0.954811062	0.367437089
ENSGALG00010023376	0.223634466	0.38097584
ENSGALG00010023377	0.96848228	0.359911688
ENSGALG00010023378	0.977985172	0.315310005
ENSGALG00010023379	0.248483015	0.473183049
ENSGALG00010023380	0.905680636	0.38380976
ENSGALG00010023381	0.964899	0.320232832
ENSGALG00010023382	0.167358607	0.152036058
ENSGALG00010023383	0.982805596	0.370694022
ENSGALG00010023384	0.60101134	0.275111357
ENSGALG00010023385	0.095322175	0.101865217
ENSGALG00010023387	0.98666365	0.326858079
ENSGALG00010023388	0.929058431	0.316182109
ENSGALG00010023389	0.423105544	0.342077982
ENSGALG00010023390	0.987605174	0.306448455
ENSGALG00010023391	0.846963964	0.182492671
ENSGALG00010023392	0.997917864	0.3310629
ENSGALG00010023393	0.900660169	0.419366969
ENSGALG00010023394	0.70614802	0.245833822
ENSGALG00010023395	0.926739686	0.315925291
ENSGALG00010023396	0.975492047	0.349719607
ENSGALG00010023397	0.757180813	0.44577217
ENSGALG00010023398	0.325642985	0.094857903
ENSGALG00010023399	0.742534087	0.027328259
ENSGALG00010023400	0.458680952	0.198997647
ENSGALG00010023401	0.871551657	0.352570062
ENSGALG00010023402	0.640788081	0.420152725
ENSGALG00010023404	0.594599986	0.228949474
ENSGALG00010023405	0.371953477	0.00858918
ENSGALG00010023406	0.001647015	0.154950019
ENSGALG00010023407	0.453540879	0.247014721
ENSGALG00010023408	0.692827028	0.217863393
ENSGALG00010023411	0.99059785	0.367231381
ENSGALG00010023412	0.957039132	0.328944395
ENSGALG00010023414	0.509219387	0.000331018
ENSGALG00010023415	0.932614978	0.393338596
ENSGALG00010023416	0.318813342	0.16516172
ENSGALG00010023418	0.974862628	0.327506489
ENSGALG00010023419	0.927391933	0.386690364
ENSGALG00010023420	0.973414127	0.354678056
ENSGALG00010023421	0.209686214	0.215611122
ENSGALG00010023422	0.810874695	0.328305461
ENSGALG00010023423	0.970649705	0.31142936
ENSGALG00010023425	0.097780439	0.187912744
ENSGALG00010023428	0.632822554	0.409454531
ENSGALG00010023429	0.265877925	0.086118689
ENSGALG00010023431	0.228755643	0.026989953
ENSGALG00010023432	0.934461598	0.235882338
ENSGALG00010023434	0.07749962	0.0183784
ENSGALG00010023437	0.568571688	0.197862468
ENSGALG00010023438	0.710221076	0.163777404

ENSGALG00010023439	0.621515981	0.260693873
ENSGALG00010023441	0.835989789	0.322006881
ENSGALG00010023442	0.808343993	0.17719435
ENSGALG00010023443	0.952903983	0.310164851
ENSGALG00010023446	0.938125062	0.326131992
ENSGALG00010023447	0.876092822	0.613655108
ENSGALG00010023448	0.765357825	0.297039493
ENSGALG00010023449	0.486399034	0.623919683
ENSGALG00010023453	0.943728836	0.223203414
ENSGALG00010023454	0.878673554	0.29278963
ENSGALG00010023455	0.865104486	0.361193518
ENSGALG00010023456	0.981011868	0.367353057
ENSGALG00010023458	0.91456267	0.357124355
ENSGALG00010023459	0.396033798	0.454055866
ENSGALG00010023460	0.620498859	0.424563545
ENSGALG00010023462	0.644539275	0.188882475
ENSGALG00010023463	0.02436834	0.087960992
ENSGALG00010023464	0.372022124	0.177125962
ENSGALG00010023465	0.575823416	0.288840255
ENSGALG00010023466	0.0668632	0.112335511
ENSGALG00010023468	0.242943051	0.154260633
ENSGALG00010023469	0.572495283	0.291977943
ENSGALG00010023470	0.028230893	0.387372767
ENSGALG00010023471	0.175020934	0.053743361
ENSGALG00010023472	0.986644452	0.281788047
ENSGALG00010023473	0.715617245	0.097711052
ENSGALG00010023475	0.200703594	0.32543139
ENSGALG00010023476	0.878862439	0.227432753
ENSGALG00010023477	0.071547032	0.081090131
ENSGALG00010023478	0.610117519	0.253443186
ENSGALG00010023479	0.6847275	0.010730322
ENSGALG00010023480	0.20888882	0.297887481
ENSGALG00010023482	0.261935957	0.356883497
ENSGALG00010023484	0.59338899	0.008488204
ENSGALG00010023485	0.043795587	0.036855734
ENSGALG00010023487	0.919179868	0.241361337
ENSGALG00010023488	0.024374485	0.442743073
ENSGALG00010023489	0.9731692	0.337152307
ENSGALG00010023490	0.3945995	0.218335422
ENSGALG00010023493	0.469322941	0.22948855
ENSGALG00010023494	0.490989737	0.119927485
ENSGALG00010023496	0.172527235	0.185827519
ENSGALG00010023497	0.580986752	0.168589055
ENSGALG00010023498	0.58690654	0.362556344
ENSGALG00010023499	0.942843726	0.351744821
ENSGALG00010023500	0.194449479	0.324733411
ENSGALG00010023502	0.777443271	0.2930474
ENSGALG00010023503	0.029037298	0.209055746
ENSGALG00010023504	0.309774718	0.023936599
ENSGALG00010023505	0.366057894	0.402915854
ENSGALG00010023506	0.086843815	0.069053955
ENSGALG00010023507	0.919001146	0.311213323
ENSGALG00010023508	0.314030453	0.025377178
ENSGALG00010023509	0.809459617	0.340956382
ENSGALG00010023510	0.491978806	0.276805245
ENSGALG00010023512	0.113557046	0.149841513

ENSGALG00010023513	0.969423782	0.346570347
ENSGALG00010023514	0.960538235	0.319817056
ENSGALG00010023515	0.476506975	0.085873798
ENSGALG00010023516	0.115840831	0.197904535
ENSGALG00010023517	0.201733461	0.252939512
ENSGALG00010023518	0.991466283	0.302739993
ENSGALG00010023519	0.590180888	0.057800757
ENSGALG00010023520	0.675622977	0.150020555
ENSGALG00010023521	0.621254102	0.233086882
ENSGALG00010023522	0.960535905	0.304838558
ENSGALG00010023523	0.421878182	0.24960237
ENSGALG00010023524	0.276986606	0.094261334
ENSGALG00010023525	0.18987241	0.309037298
ENSGALG00010023526	0.359014583	0.112997448
ENSGALG00010023527	0.945280679	0.397662329
ENSGALG00010023529	0.485646704	0.165312571
ENSGALG00010023530	0.148707918	0.00266598
ENSGALG00010023531	0.24623071	0.162379792
ENSGALG00010023532	0.149097942	0.053318751
ENSGALG00010023533	0.945010819	0.273941011
ENSGALG00010023534	0.010574754	0.139589123
ENSGALG00010023535	0.000893823	0.500599188
ENSGALG00010023536	0.791535895	0.244454688
ENSGALG00010023537	0.840407872	0.290516418
ENSGALG00010023539	0.955031412	0.222988726
ENSGALG00010023540	0.443892347	0.242324155
ENSGALG00010023543	0.911343694	0.182436821
ENSGALG00010023544	0.30012854	0.069535251
ENSGALG00010023545	0.612433973	0.237018082
ENSGALG00010023546	0.58172978	0.140153325
ENSGALG00010023547	0.739597703	0.162953653
ENSGALG00010023549	0.54305534	0.046263071
ENSGALG00010023550	0.226384882	0.175738806
ENSGALG00010023551	0.841323377	0.223496619
ENSGALG00010023552	0.384280955	0.141063301
ENSGALG00010023553	0.421071569	0.25896118
ENSGALG00010023554	0.664166216	0.012474472
ENSGALG00010023555	0.078740801	0.064527914
ENSGALG00010023556	0.584205955	0.549042928
ENSGALG00010023558	0.373310088	0.577638593
ENSGALG00010023559	0.475455904	0.093583958
ENSGALG00010023560	0.873363682	0.147462065
ENSGALG00010023561	0.518863658	0.190313836
ENSGALG00010023562	0.135086737	0.382847154
ENSGALG00010023564	0.684758639	0.216524064
ENSGALG00010023565	0.535175555	0.270437609
ENSGALG00010023569	0.954180281	0.218690441
ENSGALG00010023571	0.862946101	0.121573151
ENSGALG00010023572	0.329974751	0.054497305
ENSGALG00010023573	0.234683281	0.15335379
ENSGALG00010023574	0.536637983	0.032250921
ENSGALG00010023577	0.412003148	0.194027701
ENSGALG00010023578	0.914217652	0.217264191
ENSGALG00010023580	0.873803891	0.373138229
ENSGALG00010023583	0.751114277	0.274212426
ENSGALG00010023584	0.583651981	0.209341691

ENSGALG00010023585	0.450627971	0.267869353
ENSGALG00010023587	0.302237345	0.026759815
ENSGALG00010023588	0.934310159	0.222992737
ENSGALG00010023590	0.409716357	0.034904619
ENSGALG00010023592	0.605833859	0.008404212
ENSGALG00010023593	0.149840297	0.2335156
ENSGALG00010023594	0.576881929	0.155694121
ENSGALG00010023596	0.169555175	0.152663134
ENSGALG00010023597	0.753421774	0.18017723
ENSGALG00010023598	0.937515837	0.243831403
ENSGALG00010023599	0.256828349	0.119064955
ENSGALG00010023600	0.037122937	0.074267814
ENSGALG00010023601	0.894964929	0.250385304
ENSGALG00010023602	0.207422078	0.205899859
ENSGALG00010023603	0.990290301	0.345629515
ENSGALG00010023604	0.590640075	0.168898637
ENSGALG00010023605	0.997386063	0.31528846
ENSGALG00010023606	0.73916801	0.336752091
ENSGALG00010023607	0.367038581	0.198100366
ENSGALG00010023609	0.089560573	0.064827157
ENSGALG00010023611	0.376335967	0.225830727
ENSGALG00010023612	0.946014636	0.235500649
ENSGALG00010023613	0.907732074	0.41118074
ENSGALG00010023614	0.348032803	0.146426442
ENSGALG00010023615	0.271701261	0.072417625
ENSGALG00010023617	0.549003596	0.215398015
ENSGALG00010023618	0.184928855	0.202237853
ENSGALG00010023619	0.191411902	0.032149612
ENSGALG00010023620	0.918840987	0.302623085
ENSGALG00010023621	0.798684394	0.081410645
ENSGALG00010023622	0.361055318	0.356743384
ENSGALG00010023623	0.698377062	0.368053924
ENSGALG00010023624	0.885518271	0.399533239
ENSGALG00010023625	0.511186951	0.312227349
ENSGALG00010023626	0.959941295	0.389307738
ENSGALG00010023627	0.927180423	0.247840506
ENSGALG00010023628	0.288786203	0.111633975
ENSGALG00010023631	0.914728878	0.354267127
ENSGALG00010023633	0.045917004	0.109214977
ENSGALG00010023634	0.334983846	0.25736038
ENSGALG00010023635	0.490332839	0.117047211
ENSGALG00010023636	0.372644781	0.294065648
ENSGALG00010023637	0.92763948	0.317022664
ENSGALG00010023638	0.98838834	0.345951063
ENSGALG00010023639	0.986461806	0.294630025
ENSGALG00010023640	0.013611624	0.197553281
ENSGALG00010023641	0.422567363	0.042733148
ENSGALG00010023642	0.070044076	0.122805121
ENSGALG00010023643	0.868419107	0.309923891
ENSGALG00010023644	0.517881182	0.235040734
ENSGALG00010023645	0.603632826	0.152678479
ENSGALG00010023646	0.042747212	0.39423924
ENSGALG00010023647	0.55507486	0.122186723
ENSGALG00010023648	0.993400118	0.324833823
ENSGALG00010023649	0.244608491	0.145774328
ENSGALG00010023650	0.401639624	0.101384476

ENSGALG00010023651	0.483083958	0.120980463
ENSGALG00010023652	0.97501819	0.319422058
ENSGALG00010023654	0.291591738	0.38361006
ENSGALG00010023655	0.993093414	0.331036162
ENSGALG00010023656	0.450630049	0.55940982
ENSGALG00010023657	0.863111904	0.125885954
ENSGALG00010023658	0.68170358	0.373282021
ENSGALG00010023659	0.77294538	0.280926073
ENSGALG00010023660	0.032804077	0.393326
ENSGALG00010023663	0.898573549	0.221611186
ENSGALG00010023664	0.427771098	0.103025857
ENSGALG00010023665	0.847556785	0.219561161
ENSGALG00010023666	0.939273532	0.259710111
ENSGALG00010023667	0.660101963	0.18770249
ENSGALG00010023668	0.198273719	0.289512717
ENSGALG00010023670	0.710599603	0.202511978
ENSGALG00010023671	0.831928338	0.147152015
ENSGALG00010023673	0.992312555	0.341612817
ENSGALG00010023675	0.38813597	0.156768404
ENSGALG00010023676	0.916495629	0.28390264
ENSGALG00010023677	0.992625157	0.309520772
ENSGALG00010023679	0.85745941	0.362844732
ENSGALG00010023680	0.91705622	0.33101852
ENSGALG00010023681	0.800119699	0.286926624
ENSGALG00010023682	0.827261739	0.44244777
ENSGALG00010023683	0.766631351	0.289052456
ENSGALG00010023684	0.396211517	0.126961164
ENSGALG00010023685	0.122283444	0.198093229
ENSGALG00010023686	0.818903619	0.428047486
ENSGALG00010023687	0.075597773	0.114185715
ENSGALG00010023689	0.264931047	0.003549441
ENSGALG00010023690	0.564235723	0.202866004
ENSGALG00010023691	0.404821901	0.251237489
ENSGALG00010023692	0.125447572	0.005709717
ENSGALG00010023693	0.69241226	0.383127088
ENSGALG00010023694	0.758456323	0.255792577
ENSGALG00010023695	0.951940891	0.291435345
ENSGALG00010023696	0.853087182	0.39809182
ENSGALG00010023698	0.336901568	0.194339256
ENSGALG00010023699	0.957583496	0.331776029
ENSGALG00010023701	0.777400118	0.211604183
ENSGALG00010023702	0.378146111	0.183191241
ENSGALG00010023703	0.934755027	0.428555295
ENSGALG00010023704	0.789004443	0.117762019
ENSGALG00010023705	0.815197892	0.392689248
ENSGALG00010023706	0.036454286	0.043760178
ENSGALG00010023707	0.856510256	0.110859853
ENSGALG00010023708	0.665153208	0.066881722
ENSGALG00010023709	0.589530675	0.088166891
ENSGALG00010023710	0.896750011	0.19800571
ENSGALG00010023711	0.77899328	0.119747038
ENSGALG00010023712	0.462911791	0.282728495
ENSGALG00010023713	0.902059608	0.289768787
ENSGALG00010023714	0.519033498	0.227455988
ENSGALG00010023715	0.963657082	0.234232436
ENSGALG00010023716	0.528710171	0.344752015

ENSGALG00010023717	0.753297307	0.296880195
ENSGALG00010023719	0.317410796	0.133134556
ENSGALG00010023720	0.776768424	0.042135757
ENSGALG00010023721	0.283529993	0.098309861
ENSGALG00010023722	0.975687522	0.290852889
ENSGALG00010023724	0.063863594	0.306334881
ENSGALG00010023725	0.475332411	0.009060829
ENSGALG00010023726	0.896216365	0.148130782
ENSGALG00010023727	0.572524857	0.313115277
ENSGALG00010023728	0.454261635	0.381204938
ENSGALG00010023730	0.945304776	0.3294732
ENSGALG00010023731	0.287116597	0.083672352
ENSGALG00010023732	0.633601232	0.287387743
ENSGALG00010023733	0.885886526	0.462354939
ENSGALG00010023734	0.012184093	0.540210871
ENSGALG00010023736	0.788987846	0.121211317
ENSGALG00010023738	0.792539665	0.082640567
ENSGALG00010023739	0.837597559	0.315426837
ENSGALG00010023740	0.839075181	0.292065838
ENSGALG00010023741	0.469231849	0.226779169
ENSGALG00010023742	0.017411055	0.13007104
ENSGALG00010023743	0.939841907	0.291379468
ENSGALG00010023744	0.882056739	0.233339269
ENSGALG00010023745	0.887180978	0.305827029
ENSGALG00010023746	0.798234353	0.214021128
ENSGALG00010023747	0.658451612	0.185316664
ENSGALG00010023748	0.918625427	0.274552824
ENSGALG00010023749	0.076843932	0.181738623
ENSGALG00010023750	0.263338982	0.010334164
ENSGALG00010023751	0.810957661	0.368040313
ENSGALG00010023752	0.284755755	0.069408504
ENSGALG00010023754	0.208550923	0.676301294
ENSGALG00010023756	0.47280763	0.151326378
ENSGALG00010023757	0.428556337	0.149110462
ENSGALG00010023758	0.962658833	0.299728401
ENSGALG00010023759	0.968952678	0.368650293
ENSGALG00010023760	0.044449593	0.078223158
ENSGALG00010023762	0.995818444	0.317031331
ENSGALG00010023764	0.846784687	0.293924258
ENSGALG00010023765	0.964236876	0.273648625
ENSGALG00010023766	0.294139098	0.154124277
ENSGALG00010023767	0.971751606	0.323600228
ENSGALG00010023770	0.291516086	0.043362024
ENSGALG00010023773	0.763807712	0.177426579
ENSGALG00010023774	0.580310529	0.1979157
ENSGALG00010023775	0.903377693	0.269974786
ENSGALG00010023776	0.978695107	0.300902774
ENSGALG00010023777	0.935944575	0.265245801
ENSGALG00010023778	0.61904602	0.347261136
ENSGALG00010023781	0.219720361	0.040602198
ENSGALG00010023782	0.083918784	0.055037538
ENSGALG00010023783	0.939551829	0.359561402
ENSGALG00010023784	0.062343341	0.224527132
ENSGALG00010023786	0.033186437	0.031802851
ENSGALG00010023787	0.274632186	0.093812359
ENSGALG00010023788	0.504635697	0.336317259

ENSGALG00010023789	0.591924376	0.235659208
ENSGALG00010023790	0.18361269	0.179546988
ENSGALG00010023791	0.869891044	0.301728017
ENSGALG00010023792	0.920064556	0.321401662
ENSGALG00010023793	0.843668832	0.428007724
ENSGALG00010023794	0.949111051	0.193125103
ENSGALG00010023795	0.853037871	0.318165115
ENSGALG00010023798	0.822997261	0.202853793
ENSGALG00010023799	0.952122879	0.350301319
ENSGALG00010023800	0.948858322	0.219093769
ENSGALG00010023802	0.864937096	0.215543857
ENSGALG00010023803	0.682479832	0.210753172
ENSGALG00010023804	0.172911281	0.120219707
ENSGALG00010023805	0.81678081	0.199994501
ENSGALG00010023806	0.983615315	0.310218397
ENSGALG00010023807	0.919954359	0.167849329
ENSGALG00010023808	0.914586621	0.205983403
ENSGALG00010023809	0.92419192	0.381773181
ENSGALG00010023811	0.965475276	0.367164069
ENSGALG00010023813	0.277566769	0.107857127
ENSGALG00010023815	0.585986486	0.355036868
ENSGALG00010023817	0.91115932	0.193228073
ENSGALG00010023818	0.861368532	0.350233115
ENSGALG00010023819	0.931697855	0.429233075
ENSGALG00010023820	0.553702501	0.064845178
ENSGALG00010023821	0.872274686	0.145301997
ENSGALG00010023822	0.947372421	0.33961358
ENSGALG00010023823	0.822687215	0.199663241
ENSGALG00010023826	0.082620896	0.08126272
ENSGALG00010023827	0.329661263	0.151807418
ENSGALG00010023828	0.644080509	0.03444992
ENSGALG00010023829	0.430056041	0.123307063
ENSGALG00010023830	0.705934774	0.349333721
ENSGALG00010023831	0.50224189	0.304310982
ENSGALG00010023832	0.374061733	0.202918729
ENSGALG00010023833	0.874181903	0.348084925
ENSGALG00010023834	0.58842481	0.228910828
ENSGALG00010023835	0.980002199	0.382834835
ENSGALG00010023836	0.734764094	0.213719857
ENSGALG00010023837	0.967950359	0.241021052
ENSGALG00010023838	0.992707996	0.342172296
ENSGALG00010023840	0.812403923	0.135071898
ENSGALG00010023841	0.597769709	0.017869425
ENSGALG00010023842	0.92188737	0.303274491
ENSGALG00010023843	0.709717284	0.231313921
ENSGALG00010023845	0.216854643	0.100923479
ENSGALG00010023847	0.981211809	0.298691556
ENSGALG00010023849	0.30622539	0.12456887
ENSGALG00010023850	0.948776778	0.366907383
ENSGALG00010023851	0.500071497	0.296266538
ENSGALG00010023852	0.904549101	0.232984064
ENSGALG00010023854	0.537118235	0.117288
ENSGALG00010023857	0.737873073	0.467187998
ENSGALG00010023858	0.390466756	0.106163828
ENSGALG00010023859	0.768966884	0.290295246
ENSGALG00010023860	0.851439429	0.296113046

ENSGALG00010023861	0.772635152	0.304476088
ENSGALG00010023862	0.872468681	0.219168285
ENSGALG00010023863	0.723415313	0.400283995
ENSGALG00010023864	0.956617898	0.314646387
ENSGALG00010023866	0.918000287	0.380685492
ENSGALG00010023867	0.877279606	0.474338443
ENSGALG00010023869	0.520756827	0.173569384
ENSGALG00010023871	0.912334363	0.329115959
ENSGALG00010023872	0.812037447	0.261580288
ENSGALG00010023874	0.983797539	0.281614284
ENSGALG00010023875	0.397831073	0.425497365
ENSGALG00010023876	0.613535088	0.018140172
ENSGALG00010023878	0.97017313	0.327510325
ENSGALG00010023879	0.633473825	0.290917368
ENSGALG00010023880	0.649824708	0.183381784
ENSGALG00010023881	0.745725555	0.314471417
ENSGALG00010023883	0.862360138	0.303653855
ENSGALG00010023884	0.736569358	0.092056202
ENSGALG00010023885	0.915201912	0.333569912
ENSGALG00010023886	0.971787264	0.377211602
ENSGALG00010023887	0.936654421	0.31136357
ENSGALG00010023888	0.957187889	0.249468741
ENSGALG00010023889	0.832040122	0.153263176
ENSGALG00010023890	0.620658295	0.293519121
ENSGALG00010023891	0.621315059	0.000332034
ENSGALG00010023894	0.907533367	0.254969984
ENSGALG00010023895	0.360611506	0.184110222
ENSGALG00010023896	0.676619151	0.247856788
ENSGALG00010023902	0.912113522	0.467154971
ENSGALG00010023903	0.108099198	0.032051756
ENSGALG00010023906	0.298206494	0.36290518
ENSGALG00010023909	0.769591395	0.214923149
ENSGALG00010023910	0.981012843	0.262688988
ENSGALG00010023911	0.753007977	0.278728769
ENSGALG00010023912	0.797056042	0.233243617
ENSGALG00010023913	0.428073908	0.388937358
ENSGALG00010023914	0.906082902	0.247267713
ENSGALG00010023917	0.190266623	0.453669804
ENSGALG00010023918	0.950662783	0.270497509
ENSGALG00010023920	0.144053007	0.068080557
ENSGALG00010023921	0.840701425	0.095351219
ENSGALG00010023923	0.893538113	0.350729237
ENSGALG00010023925	0.94960542	0.274952255
ENSGALG00010023926	0.901881539	0.245252013
ENSGALG00010023927	0.956845633	0.370918807
ENSGALG00010023930	0.13811827	0.307094306
ENSGALG00010023931	0.779494941	0.380023919
ENSGALG00010023936	0.478167583	0.139734341
ENSGALG00010023937	0.964785521	0.289992345
ENSGALG00010023938	0.352442969	0.236447956
ENSGALG00010023939	0.002908085	0.034405677
ENSGALG00010023940	0.957029318	0.364134369
ENSGALG00010023941	0.07069158	0.39804429
ENSGALG00010023942	0.969768504	0.385317856
ENSGALG00010023944	0.200570274	0.091667991
ENSGALG00010023945	0.908645511	0.372554007

ENSGALG00010023946	0.917744836	0.309679569
ENSGALG00010023947	0.156157181	0.387596908
ENSGALG00010023948	0.961519069	0.235325464
ENSGALG00010023949	0.397152239	0.070389111
ENSGALG00010023953	0.936883793	0.438999264
ENSGALG00010023954	0.726408571	0.328469985
ENSGALG00010023955	0.104578818	0.09343012
ENSGALG00010023956	0.936794704	0.354744973
ENSGALG00010023957	0.997799459	0.345445892
ENSGALG00010023959	0.672955182	0.022965583
ENSGALG00010023960	0.6021949	0.04301688
ENSGALG00010023962	0.451619587	0.118509443
ENSGALG00010023963	0.562717383	0.104239849
ENSGALG00010023964	0.884428049	0.39193876
ENSGALG00010023965	0.914359563	0.31691033
ENSGALG00010023966	0.845141463	0.320927715
ENSGALG00010023967	0.416405993	0.051714552
ENSGALG00010023968	0.915039858	0.307836581
ENSGALG00010023969	0.266137114	0.107784983
ENSGALG00010023971	0.359423266	0.191109669
ENSGALG00010023972	0.877168693	0.398026361
ENSGALG00010023973	0.117321144	0.307147193
ENSGALG00010023974	0.985313903	0.292059767
ENSGALG00010023978	0.966531684	0.328942406
ENSGALG00010023979	0.295616522	0.090723462
ENSGALG00010023980	0.370747868	0.079551735
ENSGALG00010023981	0.846826886	0.327357398
ENSGALG00010023982	0.223104079	0.194413031
ENSGALG00010023983	0.400215036	0.132215663
ENSGALG00010023984	0.922596106	0.290872751
ENSGALG00010023986	0.177461543	0.306723528
ENSGALG00010023987	0.688287329	0.174255804
ENSGALG00010023989	0.09256207	0.159732277
ENSGALG00010023991	0.875703322	0.17414117
ENSGALG00010023992	0.661461841	0.38904805
ENSGALG00010023993	0.778082061	0.21614252
ENSGALG00010023996	0.856601303	0.097790814
ENSGALG00010023997	0.984278707	0.341158909
ENSGALG00010023998	0.582031621	0.001568817
ENSGALG00010023999	0.646281189	0.446419641
ENSGALG00010024001	0.945682253	0.348937381
ENSGALG00010024002	0.576721149	0.011488473
ENSGALG00010024003	0.917298932	0.398179367
ENSGALG00010024004	0.860706885	0.309515251
ENSGALG00010024005	0.953339611	0.306717714
ENSGALG00010024008	0.924888608	0.392864657
ENSGALG00010024009	0.764734738	0.028914713
ENSGALG00010024010	0.974060085	0.33903007
ENSGALG00010024011	0.969414343	0.246688096
ENSGALG00010024013	0.973212872	0.271763719
ENSGALG00010024014	0.994699132	0.337012579
ENSGALG00010024015	0.216433634	0.680598293
ENSGALG00010024016	0.895728789	0.376886014
ENSGALG00010024017	0.749394846	0.243246269
ENSGALG00010024018	0.996462935	0.344678076
ENSGALG00010024019	0.708805481	0.275187014

ENSGALG00010024020	0.867092569	0.312231358
ENSGALG00010024021	0.48197906	0.081963142
ENSGALG00010024023	0.976993451	0.322858396
ENSGALG00010024024	0.99439029	0.314359675
ENSGALG00010024025	0.979766242	0.323778175
ENSGALG00010024026	0.754865858	0.430293671
ENSGALG00010024028	0.531335435	0.288731318
ENSGALG00010024029	0.606084414	0.253395198
ENSGALG00010024030	0.965034395	0.299536693
ENSGALG00010024031	0.751722528	0.444747418
ENSGALG00010024032	0.371558109	0.423898957
ENSGALG00010024034	0.035350163	0.019912966
ENSGALG00010024035	0.574502777	0.397067528
ENSGALG00010024037	0.126324028	0.034819573
ENSGALG00010024038	0.868406946	0.075132863
ENSGALG00010024039	0.553041257	0.00907853
ENSGALG00010024040	0.653704324	0.176204078
ENSGALG00010024041	0.137855553	0.293890864
ENSGALG00010024042	0.912036849	0.25108167
ENSGALG00010024043	0.447373657	0.184053152
ENSGALG00010024044	0.05112109	0.231227304
ENSGALG00010024045	0.891028943	0.28452427
ENSGALG00010024047	0.97396033	0.233577492
ENSGALG00010024049	0.476988233	0.235688974
ENSGALG00010024051	0.997182087	0.336667203
ENSGALG00010024052	0.916091114	0.253412086
ENSGALG00010024053	0.499840346	0.201293354
ENSGALG00010024054	0.048522101	0.113076891
ENSGALG00010024056	0.620540855	0.071419948
ENSGALG00010024057	0.947503492	0.251335802
ENSGALG00010024058	0.725090021	0.232479175
ENSGALG00010024060	0.8642565	0.278192591
ENSGALG00010024061	0.262922434	0.138601464
ENSGALG00010024062	0.875843964	0.262390485
ENSGALG00010024063	0.927593497	0.185873629
ENSGALG00010024064	0.356290077	0.026301218
ENSGALG00010024065	0.824737324	0.313423516
ENSGALG00010024066	0.360294103	0.034700385
ENSGALG00010024067	0.874876159	0.395836993
ENSGALG00010024068	0.932852982	0.28754108
ENSGALG00010024069	0.670746059	0.169405533
ENSGALG00010024070	0.357147265	0.343232312
ENSGALG00010024071	0.924798793	0.438504542
ENSGALG00010024072	0.285272007	0.109169955
ENSGALG00010024073	0.300802144	0.235170054
ENSGALG00010024074	0.938380487	0.298246925
ENSGALG00010024075	0.986982301	0.339958075
ENSGALG00010024076	0.310179942	0.17632649
ENSGALG00010024077	0.702548876	0.26274626
ENSGALG00010024078	0.933449004	0.261567391
ENSGALG00010024079	0.877230931	0.38523536
ENSGALG00010024080	0.971100069	0.280153701
ENSGALG00010024081	0.456484278	0.121976299
ENSGALG00010024082	0.161377811	0.146430816
ENSGALG00010024083	0.977995676	0.377561498
ENSGALG00010024085	0.51609489	0.164505088

ENSGALG00010024086	0.088023759	0.267686088
ENSGALG00010024087	0.216433634	0.680598293
ENSGALG00010024089	0.473351661	0.011868739
ENSGALG00010024090	0.693839902	0.143514781
ENSGALG00010024091	0.954508194	0.366396619
ENSGALG00010024092	0.417149349	0.144531054
ENSGALG00010024093	0.814904523	0.097117889
ENSGALG00010024094	0.265558089	0.028821141
ENSGALG00010024095	0.930423481	0.211082171
ENSGALG00010024096	0.916854266	0.251935257
ENSGALG00010024099	0.411042209	0.405776208
ENSGALG00010024101	0.61113471	0.064504647
ENSGALG00010024102	0.705182637	0.323425353
ENSGALG00010024104	0.343448205	0.289739478
ENSGALG00010024106	0.875632043	0.2581533
ENSGALG00010024107	0.560037501	0.211796087
ENSGALG00010024110	0.971059194	0.382620561
ENSGALG00010024111	0.300468035	0.258773229
ENSGALG00010024112	0.983678345	0.383114234
ENSGALG00010024113	0.963595834	0.419528911
ENSGALG00010024114	0.285272007	0.109169955
ENSGALG00010024115	0.796027408	0.280446759
ENSGALG00010024116	0.972398009	0.420374748
ENSGALG00010024117	0.219386918	0.079388121
ENSGALG00010024118	0.921852296	0.316390748
ENSGALG00010024119	0.947081029	0.258714905
ENSGALG00010024120	0.394443593	0.09072273
ENSGALG00010024121	0.921772761	0.303818346
ENSGALG00010024122	0.973872163	0.367948352
ENSGALG00010024123	0.558114528	0.232579037
ENSGALG00010024124	0.683082269	0.694778198
ENSGALG00010024126	0.907639118	0.42300725
ENSGALG00010024128	0.89357648	0.247827819
ENSGALG00010024129	0.898807911	0.308760727
ENSGALG00010024130	0.282390713	0.086738369
ENSGALG00010024132	0.984166658	0.413467998
ENSGALG00010024133	0.964033393	0.357324331
ENSGALG00010024134	0.203991683	0.058221925
ENSGALG00010024135	0.985617392	0.322109411
ENSGALG00010024136	0.282565391	0.08222932
ENSGALG00010024137	0.987713863	0.32415635
ENSGALG00010024138	0.98601536	0.277880618
ENSGALG00010024139	0.991219017	0.315342687
ENSGALG00010024140	0.319961911	0.076840466
ENSGALG00010024141	0.253037397	0.325238276
ENSGALG00010024142	0.765097831	0.262071711
ENSGALG00010024143	0.993252319	0.280451834
ENSGALG00010024144	0.858606386	0.387068677
ENSGALG00010024145	0.04485282	0.206333841
ENSGALG00010024146	0.246181364	0.082928468
ENSGALG00010024147	0.918580542	0.363917737
ENSGALG00010024148	0.978184293	0.259585376
ENSGALG00010024149	0.944049993	0.42523136
ENSGALG00010024150	0.792580437	0.139045495
ENSGALG00010024151	0.946566084	0.240130965
ENSGALG00010024152	0.944086697	0.346729109

ENSGALG00010024153	0.305838704	0.315015182
ENSGALG00010024154	0.893507478	0.636199611
ENSGALG00010024155	0.913926807	0.260330917
ENSGALG00010024156	0.516703067	0.106045665
ENSGALG00010024157	0.849981593	0.268000526
ENSGALG00010024158	0.179699172	0.053815743
ENSGALG00010024159	0.919231567	0.340070537
ENSGALG00010024160	0.043659357	0.108092216
ENSGALG00010024162	0.755168063	0.394873083
ENSGALG00010024163	0.97420654	0.333772591
ENSGALG00010024164	0.735002391	0.20432368
ENSGALG00010024165	0.562606237	0.013379815
ENSGALG00010024166	0.531439801	0.228363633
ENSGALG00010024167	0.694135851	0.206839731
ENSGALG00010024168	0.522281502	0.237832467
ENSGALG00010024169	0.357515753	0.171891932
ENSGALG00010024170	0.440958823	0.29300341
ENSGALG00010024172	0.91535785	0.34161249
ENSGALG00010024173	0.575366559	0.219832896
ENSGALG00010024174	0.914218043	0.347527861
ENSGALG00010024175	0.768121779	0.390184258
ENSGALG00010024176	0.806400166	0.202436907
ENSGALG00010024177	0.939082924	0.283353589
ENSGALG00010024178	0.610037921	0.147477821
ENSGALG00010024179	0.680800478	0.206931468
ENSGALG00010024180	0.872756396	0.331174065
ENSGALG00010024181	0.998643363	0.322357776
ENSGALG00010024182	0.555263572	0.163067513
ENSGALG00010024183	0.964988706	0.265675309
ENSGALG00010024184	0.702973568	0.186224372
ENSGALG00010024185	0.898397193	0.163485781
ENSGALG00010024186	0.980238965	0.361163722
ENSGALG00010024187	0.928782001	0.318781044
ENSGALG00010024188	0.905199852	0.274468624
ENSGALG00010024189	0.725355825	0.099954034
ENSGALG00010024190	0.699162127	0.269329118
ENSGALG00010024191	0.980063621	0.28524585
ENSGALG00010024192	0.910821826	0.55955886
ENSGALG00010024193	0.904054629	0.299057778
ENSGALG00010024194	0.968807902	0.381762349
ENSGALG00010024195	0.941137365	0.299753379
ENSGALG00010024196	0.705024018	0.031750344
ENSGALG00010024197	0.620133574	0.416263711
ENSGALG00010024198	0.888609868	0.437472006
ENSGALG00010024199	0.552568221	0.273575816
ENSGALG00010024200	0.984474051	0.268184996
ENSGALG00010024201	0.900383539	0.185243863
ENSGALG00010024202	0.337879886	0.062255476
ENSGALG00010024203	0.685666463	0.067801887
ENSGALG00010024204	0.863463293	0.360902134
ENSGALG00010024205	0.906134097	0.31677363
ENSGALG00010024206	0.637940332	0.559304839
ENSGALG00010024207	0.473274684	0.024337086
ENSGALG00010024208	0.994441733	0.335513709
ENSGALG00010024209	0.023594547	0.219067378
ENSGALG00010024210	0.017871382	0.169452493

ENSGALG00010024211	0.390861112	0.306774675
ENSGALG00010024212	0.948483226	0.306452538
ENSGALG00010024213	0.599632921	0.162644539
ENSGALG00010024214	0.41997115	0.735256301
ENSGALG00010024215	0.40135901	0.158038591
ENSGALG00010024218	0.215971014	0.287078286
ENSGALG00010024220	0.823899404	0.307430261
ENSGALG00010024221	0.933447558	0.220766653
ENSGALG00010024222	0.278098273	0.380595449
ENSGALG00010024223	0.780833603	0.559265129
ENSGALG00010024224	0.583726226	0.120748184
ENSGALG00010024225	0.532640203	0.080229553
ENSGALG00010024226	0.982809135	0.348071085
ENSGALG00010024227	0.34525571	0.119899837
ENSGALG00010024228	0.349000274	0.018432695
ENSGALG00010024229	0.954253389	0.430098334
ENSGALG00010024230	0.956619003	0.334890282
ENSGALG00010024231	0.453780517	0.178712415
ENSGALG00010024232	0.695884453	0.276219019
ENSGALG00010024233	0.06780546	0.070637038
ENSGALG00010024234	0.920698834	0.234391233
ENSGALG00010024236	0.534258486	0.171291874
ENSGALG00010024237	0.19570691	0.192118931
ENSGALG00010024238	0.975190199	0.289841379
ENSGALG00010024240	0.72431654	0.41342
ENSGALG00010024241	0.553844186	0.199848708
ENSGALG00010024242	0.74595891	0.166817323
ENSGALG00010024243	0.382314229	0.323101603
ENSGALG00010024245	0.987622028	0.330365263
ENSGALG00010024248	0.136156562	0.206808112
ENSGALG00010024250	0.859559327	0.536151548
ENSGALG00010024251	0.976194362	0.352778379
ENSGALG00010024252	0.124893167	0.123664865
ENSGALG00010024254	0.889420171	0.162723268
ENSGALG00010024255	0.891298202	0.200053022
ENSGALG00010024256	0.160630103	0.195466318
ENSGALG00010024258	0.624152598	0.311031094
ENSGALG00010024259	0.965670858	0.322475932
ENSGALG00010024263	0.989978395	0.306643831
ENSGALG00010024264	0.96196715	0.402443649
ENSGALG00010024265	0.115416321	0.123848617
ENSGALG00010024266	0.505842873	0.539622835
ENSGALG00010024267	0.759703053	0.336050222
ENSGALG00010024270	0.963645604	0.317841953
ENSGALG00010024272	0.383947044	0.128562519
ENSGALG00010024273	0.579541593	0.209548455
ENSGALG00010024274	0.987359673	0.363140361
ENSGALG00010024275	0.719613177	0.080396286
ENSGALG00010024276	0.747850837	0.312559162
ENSGALG00010024277	0.785516777	0.097209311
ENSGALG00010024278	0.008588083	0.163472264
ENSGALG00010024280	0.923340219	0.327901885
ENSGALG00010024282	0.938472478	0.241735951
ENSGALG00010024283	0.670242187	0.041769806
ENSGALG00010024285	0.694095506	0.362040559
ENSGALG00010024286	0.307745884	0.173030822

ENSGALG00010024287	0.483744414	0.179181822
ENSGALG00010024288	0.935028593	0.231357093
ENSGALG00010024289	0.058009884	0.259299954
ENSGALG00010024290	0.455588518	0.386125355
ENSGALG00010024291	0.741210447	0.001371915
ENSGALG00010024292	0.241638714	0.135130836
ENSGALG00010024293	0.644347911	0.095730054
ENSGALG00010024295	0.078844566	0.039732258
ENSGALG00010024296	0.286398484	0.038855808
ENSGALG00010024300	0.26724906	0.207302423
ENSGALG00010024302	0.270451009	0.090541667
ENSGALG00010024303	0.86271864	0.281733221
ENSGALG00010024304	0.740305824	0.344204136
ENSGALG00010024305	0.896313593	0.249203275
ENSGALG00010024309	0.978234208	0.246189888
ENSGALG00010024311	0.942945674	0.164075482
ENSGALG00010024313	0.96296839	0.214736651
ENSGALG00010024314	0.862404771	0.315155569
ENSGALG00010024316	0.666072151	0.022482376
ENSGALG00010024317	0.560588614	0.159716999
ENSGALG00010024321	0.80234747	0.160455877
ENSGALG00010024322	0.98640537	0.35188617
ENSGALG00010024323	0.149535722	0.068962659
ENSGALG00010024325	0.862584997	0.342249514
ENSGALG00010024326	0.141170608	0.0585404
ENSGALG00010024327	0.897724576	0.175687641
ENSGALG00010024328	0.396615883	0.054829952
ENSGALG00010024330	0.976274835	0.241859513
ENSGALG00010024332	0.928792003	0.259112604
ENSGALG00010024333	0.281831589	0.169178248
ENSGALG00010024334	0.985158647	0.316352496
ENSGALG00010024335	0.763723873	0.327278743
ENSGALG00010024336	0.953492229	0.293443388
ENSGALG00010024337	0.054101188	0.196447772
ENSGALG00010024338	0.414392356	0.086207952
ENSGALG00010024339	0.377109681	0.165960302
ENSGALG00010024340	0.956128017	0.273855424
ENSGALG00010024342	0.98279423	0.310857809
ENSGALG00010024343	0.993156861	0.337531531
ENSGALG00010024344	0.300742251	0.20883146
ENSGALG00010024345	0.321663115	0.58615923
ENSGALG00010024346	0.42516356	0.082151133
ENSGALG00010024347	0.888208889	0.369220805
ENSGALG00010024348	0.525684152	0.203083479
ENSGALG00010024349	0.569457283	0.185093444
ENSGALG00010024350	0.995186503	0.32538432
ENSGALG00010024351	0.859036335	0.221887125
ENSGALG00010024352	0.325513958	0.002237464
ENSGALG00010024353	0.374990794	0.095543633
ENSGALG00010024354	0.924200145	0.253847418
ENSGALG00010024355	0.121435942	0.130497039
ENSGALG00010024356	0.871993098	0.294239668
ENSGALG00010024357	0.34081212	0.34730063
ENSGALG00010024358	0.950896939	0.3675656
ENSGALG00010024359	0.403975313	0.210042477
ENSGALG00010024360	0.93809323	0.405267509

ENSGALG00010024361	0.101848016	0.328795008
ENSGALG00010024362	0.759144895	0.274941017
ENSGALG00010024363	0.92421773	0.444420018
ENSGALG00010024364	0.256111629	0.409080009
ENSGALG00010024365	0.951515415	0.29100591
ENSGALG00010024366	0.297741951	0.095008226
ENSGALG00010024367	0.039056979	0.063555219
ENSGALG00010024368	0.728616235	0.249019073
ENSGALG00010024369	0.990246785	0.314954392
ENSGALG00010024370	0.96947825	0.265452472
ENSGALG00010024371	0.926830509	0.286390453
ENSGALG00010024373	0.993968977	0.351056436
ENSGALG00010024374	0.982971037	0.358673173
ENSGALG00010024376	0.279307332	0.330709573
ENSGALG00010024377	0.832344409	0.180303167
ENSGALG00010024378	0.700438333	0.237638469
ENSGALG00010024380	0.371293896	0.683259107
ENSGALG00010024382	0.306756732	0.327062686
ENSGALG00010024383	0.97680145	0.352296304
ENSGALG00010024384	0.36437197	0.115555231
ENSGALG00010024386	0.946120875	0.372447118
ENSGALG00010024387	0.66229661	0.308173107
ENSGALG00010024388	0.88460955	0.400714472
ENSGALG00010024389	0.958433884	0.336442111
ENSGALG00010024390	0.848926858	0.118144942
ENSGALG00010024391	0.898923763	0.263775317
ENSGALG00010024392	0.981726533	0.335951824
ENSGALG00010024393	0.745954491	0.012573642
ENSGALG00010024394	0.524974397	0.236555333
ENSGALG00010024396	0.782143223	0.330569409
ENSGALG00010024397	0.290122505	0.464993379
ENSGALG00010024398	0.733530075	0.207300024
ENSGALG00010024399	0.945507512	0.238718466
ENSGALG00010024400	0.730051702	0.023873644
ENSGALG00010024401	0.94771443	0.369086826
ENSGALG00010024402	0.986089243	0.365336158
ENSGALG00010024403	0.543912245	0.445639322
ENSGALG00010024404	0.219982136	0.067777564
ENSGALG00010024405	0.394494248	0.128304162
ENSGALG00010024406	0.493944368	0.007579746
ENSGALG00010024407	0.822106224	0.179298165
ENSGALG00010024409	0.654172822	0.239627258
ENSGALG00010024410	0.939249224	0.297047629
ENSGALG00010024411	0.081605615	0.316663828
ENSGALG00010024412	0.894237202	0.144383439
ENSGALG00010024413	0.969331077	0.398687953
ENSGALG00010024414	0.896780116	0.331752071
ENSGALG00010024415	0.893098686	0.206534817
ENSGALG00010024416	0.668121329	0.141282544
ENSGALG00010024418	0.037531409	0.198315327
ENSGALG00010024419	0.262272194	0.246897288
ENSGALG00010024420	0.378770369	0.182010471
ENSGALG00010024421	0.191554037	0.346673119
ENSGALG00010024423	0.442136407	0.16808539
ENSGALG00010024424	0.602949015	0.163139349
ENSGALG00010024425	0.946186299	0.272644626

ENSGALG00010024426	0.486630026	0.077363626
ENSGALG00010024427	0.954865792	0.265593765
ENSGALG00010024428	0.905334068	0.26161319
ENSGALG00010024430	0.094278299	0.214518607
ENSGALG00010024431	0.665219189	0.158210097
ENSGALG00010024433	0.85255321	0.322427061
ENSGALG00010024434	0.421367771	0.056980992
ENSGALG00010024437	0.921041112	0.355376791
ENSGALG00010024438	0.855377832	0.358705465
ENSGALG00010024439	0.976766196	0.393848383
ENSGALG00010024440	0.996964743	0.310337661
ENSGALG00010024441	0.946549793	0.272645677
ENSGALG00010024442	0.062393725	0.163601889
ENSGALG00010024446	0.298345482	0.168936545
ENSGALG00010024449	0.937687803	0.343427512
ENSGALG00010024451	0.193560686	0.051572353
ENSGALG00010024452	0.923207365	0.275771477
ENSGALG00010024453	0.58708625	0.190948712
ENSGALG00010024454	0.372565772	0.258158389
ENSGALG00010024455	0.235386633	0.576334352
ENSGALG00010024456	0.420587617	0.221407689
ENSGALG00010024457	0.814440789	0.270544962
ENSGALG00010024459	0.97920429	0.274504665
ENSGALG00010024460	0.847103313	0.363656718
ENSGALG00010024462	0.195616753	0.017793808
ENSGALG00010024463	0.717453766	0.151103561
ENSGALG00010024464	0.746308201	0.216902622
ENSGALG00010024465	0.780523086	0.263854289
ENSGALG00010024466	0.413687687	0.012270907
ENSGALG00010024467	0.903113941	0.20086854
ENSGALG00010024469	0.114859951	0.072620953
ENSGALG00010024470	0.421519706	0.326436763
ENSGALG00010024471	0.463952287	0.041461167
ENSGALG00010024472	0.989114173	0.32784241
ENSGALG00010024473	0.904432941	0.189384343
ENSGALG00010024474	0.942395058	0.312938165
ENSGALG00010024475	0.629716211	0.333757603
ENSGALG00010024476	0.355103667	0.406369538
ENSGALG00010024477	0.636925286	0.263980061
ENSGALG00010024478	0.362208264	0.229117202
ENSGALG00010024479	0.795502108	0.24890471
ENSGALG00010024480	0.560720159	0.065498826
ENSGALG00010024481	0.477031301	0.031061569
ENSGALG00010024482	0.97724602	0.27536714
ENSGALG00010024486	0.405598105	0.197024946
ENSGALG00010024488	0.811573327	0.313762984
ENSGALG00010024490	0.900456918	0.329225006
ENSGALG00010024492	0.443706647	0.636113511
ENSGALG00010024493	0.946772446	0.34883034
ENSGALG00010024494	0.206872378	0.294006858
ENSGALG00010024495	0.419809954	0.209692258
ENSGALG00010024496	0.991886738	0.325889153
ENSGALG00010024497	0.960522415	0.400577485
ENSGALG00010024498	0.745417199	0.430371777
ENSGALG00010024499	0.411709875	0.233774138
ENSGALG00010024500	0.85100392	0.430111268

ENSGALG00010024501	0.894788342	0.406439456
ENSGALG00010024502	0.989225409	0.314278076
ENSGALG00010024503	0.358264974	0.222718588
ENSGALG00010024504	0.932881248	0.289063515
ENSGALG00010024505	0.984716162	0.287069872
ENSGALG00010024506	0.977189137	0.301765131
ENSGALG00010024507	0.614816731	0.06924813
ENSGALG00010024508	0.93565148	0.4035489
ENSGALG00010024509	0.25310292	0.004509242
ENSGALG00010024510	0.866769034	0.221599439
ENSGALG00010024511	0.570967201	0.345811581
ENSGALG00010024512	0.582128374	0.100112354
ENSGALG00010024513	0.820465703	0.309808729
ENSGALG00010024514	0.669411774	0.307448069
ENSGALG00010024515	0.420105365	0.00246261
ENSGALG00010024516	0.976541153	0.346282839
ENSGALG00010024517	0.926502886	0.251136033
ENSGALG00010024518	0.623577956	0.23736519
ENSGALG00010024519	0.91347205	0.251633679
ENSGALG00010024520	0.992247546	0.343413731
ENSGALG00010024521	0.351650508	0.103083755
ENSGALG00010024522	0.601181112	0.169222213
ENSGALG00010024523	0.837233901	0.334677824
ENSGALG00010024524	0.732395306	0.17047981
ENSGALG00010024525	0.865390814	0.203163316
ENSGALG00010024526	0.653990471	0.299089903
ENSGALG00010024527	0.105509146	0.088660351
ENSGALG00010024528	0.443683066	0.347974878
ENSGALG00010024529	0.971615881	0.333689181
ENSGALG00010024530	0.709181471	0.143689578
ENSGALG00010024531	0.606378832	0.318854128
ENSGALG00010024532	0.987451337	0.297105872
ENSGALG00010024533	0.962062188	0.30187078
ENSGALG00010024534	0.056329846	0.124788368
ENSGALG00010024535	0.908011413	0.289802287
ENSGALG00010024536	0.84956814	0.010479029
ENSGALG00010024537	0.795389563	0.313418231
ENSGALG00010024538	0.271701261	0.072417625
ENSGALG00010024539	0.978103804	0.295834202
ENSGALG00010024540	0.927050141	0.365236609
ENSGALG00010024541	0.819110136	0.259231697
ENSGALG00010024542	0.956129924	0.388778807
ENSGALG00010024544	0.721349453	0.32597117
ENSGALG00010024545	0.882423251	0.430054572
ENSGALG00010024546	0.995571292	0.357075407
ENSGALG00010024547	0.993333584	0.330116766
ENSGALG00010024548	0.363656258	0.105554037
ENSGALG00010024549	0.620366695	0.283453462
ENSGALG00010024550	0.684924159	0.080069634
ENSGALG00010024551	0.989572314	0.37038694
ENSGALG00010024552	0.798085397	0.243544033
ENSGALG00010024553	0.764340388	0.22764294
ENSGALG00010024554	0.069183191	0.109148621
ENSGALG00010024555	0.792518093	0.408160938
ENSGALG00010024556	0.94526187	0.357716709
ENSGALG00010024557	0.703835502	0.075634491

ENSGALG00010024558	0.828084835	0.275772124
ENSGALG00010024559	0.520924328	0.007104511
ENSGALG00010024560	0.201073322	0.178485389
ENSGALG00010024561	0.874568813	0.220147561
ENSGALG00010024562	0.762268852	0.236716063
ENSGALG00010024563	0.305820093	0.038271891
ENSGALG00010024564	0.97540583	0.339153307
ENSGALG00010024565	0.265891102	0.176970097
ENSGALG00010024566	0.944253777	0.349031723
ENSGALG00010024567	0.970480981	0.377087436
ENSGALG00010024568	0.433930714	0.243138129
ENSGALG00010024569	0.258340534	0.102016698
ENSGALG00010024570	0.998919402	0.333438041
ENSGALG00010024571	0.954501426	0.347966514
ENSGALG00010024573	0.857180308	0.208117038
ENSGALG00010024574	0.237340083	0.254250233
ENSGALG00010024575	0.94691443	0.168857687
ENSGALG00010024576	0.963097427	0.29460877
ENSGALG00010024577	0.064315755	0.042890996
ENSGALG00010024578	0.467566706	0.290424436
ENSGALG00010024579	0.916102362	0.312623029
ENSGALG00010024580	0.975473445	0.327938537
ENSGALG00010024581	0.961816548	0.29727456
ENSGALG00010024582	0.917012776	0.289073621
ENSGALG00010024583	0.819163263	0.348627411
ENSGALG00010024584	0.192273884	0.326216822
ENSGALG00010024585	0.942011931	0.266924282
ENSGALG00010024586	0.974396052	0.301084805
ENSGALG00010024587	0.5924837	0.439479214
ENSGALG00010024588	0.688350495	0.403498763
ENSGALG00010024589	0.900756522	0.159815087
ENSGALG00010024590	0.954520345	0.35100248
ENSGALG00010024591	0.568120494	0.147854416
ENSGALG00010024592	0.964209314	0.386062614
ENSGALG00010024593	0.899856322	0.413215331
ENSGALG00010024594	0.983843162	0.330776316
ENSGALG00010024595	0.989736769	0.345415666
ENSGALG00010024596	0.007365385	0.049175167
ENSGALG00010024597	0.928996311	0.22027782
ENSGALG00010024598	0.089692171	0.143904485
ENSGALG00010024599	0.416876165	0.226255574
ENSGALG00010024600	0.877643849	0.265967241
ENSGALG00010024601	0.960488133	0.494747036
ENSGALG00010024602	0.429345117	0.098190018
ENSGALG00010024603	0.191834552	0.177665559
ENSGALG00010024604	0.971567019	0.330286684
ENSGALG00010024605	0.66195373	0.485018175
ENSGALG00010024606	0.532144827	0.174565258
ENSGALG00010024607	0.665930992	0.51567616
ENSGALG00010024608	0.921061847	0.252341354
ENSGALG00010024609	0.994674521	0.340453068
ENSGALG00010024610	0.952910729	0.258316971
ENSGALG00010024611	0.676477491	0.062151194
ENSGALG00010024612	0.32996091	0.145094988
ENSGALG00010024613	0.935681983	0.279657978
ENSGALG00010024614	0.956793339	0.32153939

ENSGALG00010024615	0.497507349	0.397748813
ENSGALG00010024616	0.857521745	0.287463623
ENSGALG00010024617	0.358098542	0.0976513
ENSGALG00010024618	0.947805167	0.360829149
ENSGALG00010024619	0.499142582	0.432132385
ENSGALG00010024620	0.871351189	0.232832656
ENSGALG00010024621	0.93894484	0.300272524
ENSGALG00010024622	0.863174239	0.310945371
ENSGALG00010024623	0.982863169	0.255781617
ENSGALG00010024624	0.804492513	0.139495945
ENSGALG00010024625	0.93958399	0.360175124
ENSGALG00010024626	0.190754494	0.355847884
ENSGALG00010024627	0.991776263	0.33943188
ENSGALG00010024628	0.736741874	0.195219022
ENSGALG00010024629	0.602919333	0.295079302
ENSGALG00010024630	0.839213727	0.137504255
ENSGALG00010024631	0.948232131	0.346171508
ENSGALG00010024632	0.558439856	0.475666593
ENSGALG00010024633	0.993575003	0.318968455
ENSGALG00010024634	0.794257661	0.257616995
ENSGALG00010024635	0.789209856	0.352276847
ENSGALG00010024636	0.252096162	0.201695579
ENSGALG00010024637	0.950715785	0.329206622
ENSGALG00010024638	0.259427789	0.330853963
ENSGALG00010024639	0.320095025	0.178953894
ENSGALG00010024640	0.403564887	0.075513854
ENSGALG00010024641	0.959156938	0.402061857
ENSGALG00010024642	0.064747855	0.283695507
ENSGALG00010024643	0.907834095	0.346134967
ENSGALG00010024644	0.842142111	0.251358244
ENSGALG00010024645	0.905131243	0.242795197
ENSGALG00010024646	0.811570128	0.344976885
ENSGALG00010024647	0.301969328	0.094409867
ENSGALG00010024648	0.688005732	0.331872662
ENSGALG00010024649	0.193469558	0.076744911
ENSGALG00010024650	0.995876569	0.343354452
ENSGALG00010024651	0.93058187	0.314932678
ENSGALG00010024652	0.669656369	0.295810794
ENSGALG00010024653	0.923822202	0.200824506
ENSGALG00010024654	0.945748973	0.288437262
ENSGALG00010024655	0.873449666	0.463988549
ENSGALG00010024656	0.858805244	0.266327593
ENSGALG00010024657	0.817821204	0.093534496
ENSGALG00010024658	0.922522885	0.351293118
ENSGALG00010024659	0.972960813	0.257527856
ENSGALG00010024660	0.324830857	0.075410556
ENSGALG00010024662	0.977804945	0.270999346
ENSGALG00010024663	0.87715115	0.294531875
ENSGALG00010024664	0.253629479	0.007484988
ENSGALG00010024665	0.961836848	0.256318622
ENSGALG00010024666	0.122944626	0.004296002
ENSGALG00010024667	0.924510534	0.281564285
ENSGALG00010024668	0.858896091	0.378085121
ENSGALG00010024669	0.452233976	0.113922075
ENSGALG00010024670	0.405203022	0.382460151
ENSGALG00010024671	0.933363404	0.325761949

ENSGALG00010024672	0.918739619	0.282774592
ENSGALG00010024673	0.971999004	0.313564488
ENSGALG00010024674	0.60152572	0.393403705
ENSGALG00010024675	0.94556374	0.320511142
ENSGALG00010024676	0.978929436	0.322304902
ENSGALG00010024677	0.936347049	0.193460062
ENSGALG00010024678	0.505240785	0.210037772
ENSGALG00010024679	0.95950786	0.27834512
ENSGALG00010024680	0.40243889	0.412441434
ENSGALG00010024681	0.918886362	0.26552176
ENSGALG00010024682	0.588431339	0.020960522
ENSGALG00010024683	0.988910733	0.33338333
ENSGALG00010024684	0.710948732	0.078913808
ENSGALG00010024685	0.375245549	0.38007076
ENSGALG00010024686	0.979936427	0.233613041
ENSGALG00010024687	0.868854824	0.396792595
ENSGALG00010024688	0.853770931	0.220154128
ENSGALG00010024689	0.994961193	0.358333155
ENSGALG00010024690	0.865333099	0.44728367
ENSGALG00010024691	0.818939077	0.342782001
ENSGALG00010024692	0.8756233	0.349844053
ENSGALG00010024693	0.913679535	0.471011141
ENSGALG00010024694	0.373674325	0.539832752
ENSGALG00010024695	0.267595285	0.248200624
ENSGALG00010024696	0.992147061	0.367167732
ENSGALG00010024697	0.853673804	0.158517556
ENSGALG00010024698	0.695616895	0.513998324
ENSGALG00010024699	0.965856706	0.296622501
ENSGALG00010024700	0.971760762	0.272753701
ENSGALG00010024701	0.588976545	0.170396878
ENSGALG00010024702	0.537084042	0.243805645
ENSGALG00010024703	0.114513717	0.23893036
ENSGALG00010024705	0.946529569	0.222749881
ENSGALG00010024706	0.075951677	0.352844518
ENSGALG00010024707	0.356640388	0.092267518
ENSGALG00010024708	0.245809998	0.281845415
ENSGALG00010024709	0.985285649	0.311370123
ENSGALG00010024710	0.980739633	0.34642565
ENSGALG00010024711	0.788115565	0.23832183
ENSGALG00010024712	0.886064071	0.483632916
ENSGALG00010024713	0.965787816	0.212740799
ENSGALG00010024714	0.968763351	0.333712563
ENSGALG00010024715	0.337012172	0.084610335
ENSGALG00010024716	0.852127736	0.107560521
ENSGALG00010024717	0.987632262	0.325405337
ENSGALG00010024718	0.974090001	0.407870468
ENSGALG00010024719	0.591793903	0.320427475
ENSGALG00010024720	0.382885845	0.114235036
ENSGALG00010024721	0.149520162	0.3561471
ENSGALG00010024722	0.822271942	0.300354992
ENSGALG00010024723	0.960549872	0.371677262
ENSGALG00010024724	0.79101278	0.565495187
ENSGALG00010024726	0.512523124	0.216052958
ENSGALG00010024728	0.778539144	0.430435878
ENSGALG00010024729	0.731000329	0.374219058
ENSGALG00010024730	0.079697525	0.247475839

ENSGALG00010024731	0.833607743	0.233954597
ENSGALG00010024732	0.914484282	0.362328684
ENSGALG00010024733	0.951300763	0.315025833
ENSGALG00010024734	0.677431268	0.214805839
ENSGALG00010024735	0.90448037	0.244648514
ENSGALG00010024736	0.397407812	0.1461189
ENSGALG00010024737	0.572619595	0.368302797
ENSGALG00010024738	0.980023513	0.360370743
ENSGALG00010024739	0.066270896	0.0153241
ENSGALG00010024741	0.888479379	0.293219564
ENSGALG00010024742	0.305859218	0.287846736
ENSGALG00010024743	0.140036041	0.46691462
ENSGALG00010024744	0.755821401	0.303315899
ENSGALG00010024745	0.743585378	0.255460116
ENSGALG00010024747	0.791089726	0.48498188
ENSGALG00010024748	0.931822221	0.404458081
ENSGALG00010024749	0.974759828	0.244822196
ENSGALG00010024750	0.983944441	0.333548212
ENSGALG00010024751	0.156734101	0.030679904
ENSGALG00010024752	0.280061691	0.1627956
ENSGALG00010024753	0.89207282	0.383053964
ENSGALG00010024754	0.982573186	0.319434912
ENSGALG00010024755	0.847804458	0.235840228
ENSGALG00010024756	0.794067842	0.092664177
ENSGALG00010024757	0.972582771	0.318814594
ENSGALG00010024758	0.743165017	0.485752857
ENSGALG00010024759	0.994732262	0.297286764
ENSGALG00010024760	0.252743649	0.138609265
ENSGALG00010024761	0.770575514	0.404414092
ENSGALG00010024762	0.633477786	0.214861527
ENSGALG00010024763	0.609384942	0.623083179
ENSGALG00010024764	0.952790551	0.18568364
ENSGALG00010024765	0.977634444	0.30096179
ENSGALG00010024766	0.388582483	0.339106569
ENSGALG00010024767	0.93800205	0.387383449
ENSGALG00010024768	0.812789139	0.436819204
ENSGALG00010024769	0.98919052	0.328059362
ENSGALG00010024770	0.988286618	0.320761892
ENSGALG00010024771	0.922581862	0.24932104
ENSGALG00010024772	0.508846919	0.38673315
ENSGALG00010024773	0.972806604	0.326609324
ENSGALG00010024775	0.319535121	0.299641561
ENSGALG00010024776	0.537575252	0.495017876
ENSGALG00010024777	0.682012421	0.208543461
ENSGALG00010024778	0.965899643	0.350670658
ENSGALG00010024779	0.041009301	0.044772762
ENSGALG00010024781	0.883499015	0.176086165
ENSGALG00010024783	0.182804957	0.281769643
ENSGALG00010024784	0.249440845	0.172568822
ENSGALG00010024785	0.839772061	0.369647034
ENSGALG00010024786	0.988742133	0.340508498
ENSGALG00010024787	0.829531142	0.355695101
ENSGALG00010024788	0.942684556	0.312312632
ENSGALG00010024789	0.934506564	0.266301166
ENSGALG00010024791	0.972800951	0.263326447
ENSGALG00010024792	0.861637784	0.432620394

ENSGALG00010024793	0.822197428	0.408123314
ENSGALG00010024794	0.720073806	0.275653957
ENSGALG00010024795	0.957314281	0.196228815
ENSGALG00010024796	0.186582289	0.445156674
ENSGALG00010024797	0.980817577	0.316852726
ENSGALG00010024798	0.564732697	0.00178397
ENSGALG00010024799	0.161420921	0.134754424
ENSGALG00010024800	0.567229998	0.070357518
ENSGALG00010024801	0.365076351	0.244094112
ENSGALG00010024802	0.236934387	0.240578385
ENSGALG00010024803	0.82642178	0.408570029
ENSGALG00010024804	0.927019301	0.385075678
ENSGALG00010024805	0.909778703	0.251532241
ENSGALG00010024806	0.538492845	0.119257342
ENSGALG00010024807	0.935446183	0.350853368
ENSGALG00010024808	0.798470656	0.314803141
ENSGALG00010024809	0.64282685	0.078889244
ENSGALG00010024810	0.977005661	0.362638273
ENSGALG00010024811	0.943688558	0.207991316
ENSGALG00010024812	0.185427325	0.19785289
ENSGALG00010024813	0.475125876	0.004425468
ENSGALG00010024814	0.272451366	0.364131713
ENSGALG00010024816	0.414775335	0.149645142
ENSGALG00010024817	0.968022918	0.382848071
ENSGALG00010024818	0.713520704	0.26760434
ENSGALG00010024820	0.696377772	0.27145458
ENSGALG00010024821	0.953356635	0.361282307
ENSGALG00010024822	0.939143804	0.300262636
ENSGALG00010024823	0.992698609	0.314684921
ENSGALG00010024824	0.229997225	0.050584848
ENSGALG00010024825	0.961179963	0.295138342
ENSGALG00010024826	0.71187845	0.262733942
ENSGALG00010024827	0.231665585	0.14295467
ENSGALG00010024828	0.739913083	0.322589287
ENSGALG00010024829	0.975193578	0.330554744
ENSGALG00010024830	0.619593331	0.178983235
ENSGALG00010024831	0.310232863	0.094339566
ENSGALG00010024833	0.990494436	0.345302743
ENSGALG00010024834	0.907417583	0.235733588
ENSGALG00010024835	0.771756901	0.16030583
ENSGALG00010024836	0.961170492	0.277032015
ENSGALG00010024837	0.962932737	0.232984537
ENSGALG00010024839	0.924405583	0.24746687
ENSGALG00010024840	0.446884218	0.130034947
ENSGALG00010024841	0.620222601	0.202023861
ENSGALG00010024842	0.915583648	0.258067654
ENSGALG00010024843	0.945933115	0.298426284
ENSGALG00010024844	0.673395279	0.702571801
ENSGALG00010024845	0.956839761	0.324100564
ENSGALG00010024846	0.952399951	0.131585431
ENSGALG00010024847	0.923538253	0.270936733
ENSGALG00010024848	0.926520626	0.328967713
ENSGALG00010024849	0.297608073	0.300759368
ENSGALG00010024850	0.969623896	0.30272169
ENSGALG00010024851	0.272141256	0.21228556
ENSGALG00010024852	0.747463451	0.2196531

ENSGALG00010024853	0.831640384	0.243044304
ENSGALG00010024854	0.944639246	0.304406816
ENSGALG00010024855	0.953883958	0.248276655
ENSGALG00010024856	0.968357344	0.370491506
ENSGALG00010024857	0.829996854	0.337821529
ENSGALG00010024858	0.788094636	0.218046758
ENSGALG00010024859	0.937662456	0.291733394
ENSGALG00010024860	0.240438453	0.047706487
ENSGALG00010024861	0.820703279	0.273809835
ENSGALG00010024862	0.97926546	0.238989574
ENSGALG00010024863	0.247650251	0.143702356
ENSGALG00010024864	0.655501463	0.358957709
ENSGALG00010024865	0.487194845	0.226895153
ENSGALG00010024866	0.919870403	0.329331489
ENSGALG00010024867	0.501763497	0.062230736
ENSGALG00010024868	0.948410996	0.297228534
ENSGALG00010024869	0.960432753	0.348354755
ENSGALG00010024870	0.968352748	0.277729042
ENSGALG00010024871	0.719931425	0.098343627
ENSGALG00010024872	0.480064631	0.223011743
ENSGALG00010024873	0.947364425	0.299710479
ENSGALG00010024874	0.984878347	0.306054417
ENSGALG00010024875	0.670306739	0.21391956
ENSGALG00010024876	0.96646384	0.379907292
ENSGALG00010024877	0.828634354	0.066060048
ENSGALG00010024878	0.977359631	0.279656718
ENSGALG00010024879	0.430033034	0.07689666
ENSGALG00010024880	0.116438289	0.032494398
ENSGALG00010024881	0.497697539	0.125367511
ENSGALG00010024882	0.733636158	0.115083961
ENSGALG00010024883	0.829610613	0.215670537
ENSGALG00010024884	0.952741772	0.306601657
ENSGALG00010024885	0.12813337	0.142822932
ENSGALG00010024886	0.925932856	0.312321543
ENSGALG00010024887	0.969866016	0.247911804
ENSGALG00010024888	0.884747929	0.230116724
ENSGALG00010024889	0.921326726	0.244451252
ENSGALG00010024890	0.937277227	0.354286179
ENSGALG00010024891	0.948931383	0.210993427
ENSGALG00010024892	0.581145035	0.301720316
ENSGALG00010024893	0.553561688	0.188699465
ENSGALG00010024894	0.96044577	0.348539935
ENSGALG00010024895	0.340363993	0.260480035
ENSGALG00010024896	0.98659107	0.321933022
ENSGALG00010024897	0.971655449	0.363961461
ENSGALG00010024898	0.944942546	0.34076401
ENSGALG00010024899	0.916436179	0.338206309
ENSGALG00010024900	0.80972239	0.403953044
ENSGALG00010024901	0.805335911	0.163057856
ENSGALG00010024902	0.885162121	0.278813442
ENSGALG00010024903	0.945916226	0.339914505
ENSGALG00010024904	0.944918904	0.291626145
ENSGALG00010024905	0.76164317	0.462641058
ENSGALG00010024907	0.965831071	0.268838175
ENSGALG00010024908	0.979730628	0.292088652
ENSGALG00010024909	0.988935131	0.321132266

ENSGALG00010024910	0.971762965	0.392089788
ENSGALG00010024911	0.956221037	0.328915234
ENSGALG00010024913	0.158051691	0.208262414
ENSGALG00010024914	0.241821292	0.324550082
ENSGALG00010024915	0.869728045	0.12166943
ENSGALG00010024917	0.107953177	0.026242807
ENSGALG00010024918	0.911814058	0.23070267
ENSGALG00010024919	0.747851888	0.159434361
ENSGALG00010024920	0.981364775	0.286529719
ENSGALG00010024921	0.761297445	0.442330444
ENSGALG00010024922	0.81250693	0.282300494
ENSGALG00010024924	0.322642809	0.120881016
ENSGALG00010024925	0.91235628	0.235775612
ENSGALG00010024926	0.018778296	0.060692393
ENSGALG00010024927	0.480317847	0.017805989
ENSGALG00010024928	0.95657031	0.273968721
ENSGALG00010024929	0.975939025	0.301107415
ENSGALG00010024930	0.873512706	0.214624059
ENSGALG00010024931	0.042418018	0.112480393
ENSGALG00010024933	0.989264147	0.319634053
ENSGALG00010024934	0.988453997	0.347088676
ENSGALG00010024935	0.987974622	0.305156664
ENSGALG00010024936	0.766488883	0.146884804
ENSGALG00010024937	0.203077011	0.083059123
ENSGALG00010024938	0.704159287	0.395939705
ENSGALG00010024939	0.950219708	0.418434161
ENSGALG00010024940	0.893938395	0.347346704
ENSGALG00010024941	0.908935449	0.195594173
ENSGALG00010024942	0.446864709	0.223963771
ENSGALG00010024943	0.947189166	0.315988817
ENSGALG00010024944	0.164765055	0.16438162
ENSGALG00010024945	0.261261723	0.134821708
ENSGALG00010024946	0.359521837	0.193677693
ENSGALG00010024947	0.896512193	0.311733395
ENSGALG00010024948	0.980629202	0.304710522
ENSGALG00010024949	0.657814105	0.046957714
ENSGALG00010024950	0.969969024	0.365484631
ENSGALG00010024951	0.189240572	0.04996112
ENSGALG00010024952	0.00196969	0.072516884
ENSGALG00010024953	0.614958015	0.002177562
ENSGALG00010024954	0.081528906	0.092545242
ENSGALG00010024955	0.026966462	0.158284821
ENSGALG00010024956	0.230041278	0.202353941
ENSGALG00010024958	0.28880012	0.418789177
ENSGALG00010024959	0.995015266	0.338041235
ENSGALG00010024960	0.887442078	0.649127297
ENSGALG00010024961	0.984829417	0.315108334
ENSGALG00010024962	0.994923944	0.339038493
ENSGALG00010024963	0.702496018	0.193369339
ENSGALG00010024964	0.164507058	0.357627441
ENSGALG00010024965	0.645546274	0.301340509
ENSGALG00010024966	0.59657963	0.144958332
ENSGALG00010024967	0.307395467	0.506952586
ENSGALG00010024968	0.904123013	0.328560984
ENSGALG00010024969	0.161250365	0.239164779
ENSGALG00010024970	0.922646512	0.216277339

ENSGALG00010024971	0.364596763	0.264660927
ENSGALG00010024973	0.591131714	0.336231502
ENSGALG00010024974	0.933942759	0.264401557
ENSGALG00010024975	0.948055091	0.333549656
ENSGALG00010024976	0.161898872	0.170558899
ENSGALG00010024977	0.944580095	0.233679633
ENSGALG00010024978	0.639323424	0.286491768
ENSGALG00010024979	0.203991683	0.058221925
ENSGALG00010024980	0.431645979	0.199150541
ENSGALG00010024982	0.410227587	0.202927549
ENSGALG00010024983	0.963816852	0.36580031
ENSGALG00010024984	0.37375514	0.063525011
ENSGALG00010024985	0.450053866	0.189032749
ENSGALG00010024986	0.941085989	0.416508571
ENSGALG00010024987	0.021004637	0.090375244
ENSGALG00010024988	0.198172997	0.262926778
ENSGALG00010024989	0.213951738	0.050693271
ENSGALG00010024990	0.62934714	0.328551814
ENSGALG00010024991	0.40834889	0.0940038
ENSGALG00010024992	0.298140893	0.421788492
ENSGALG00010024993	0.693152292	0.192497857
ENSGALG00010024994	0.965084281	0.328806081
ENSGALG00010024996	0.597491619	0.285401279
ENSGALG00010024997	0.24841393	0.254540292
ENSGALG00010024999	0.508758851	0.37888574
ENSGALG00010025000	0.33135134	0.153585636
ENSGALG00010025003	0.981628141	0.326772285
ENSGALG00010025004	0.157068597	0.037983046
ENSGALG00010025005	0.983872808	0.27750662
ENSGALG00010025007	0.940619419	0.302355111
ENSGALG00010025008	0.340235079	0.376875117
ENSGALG00010025010	0.951881189	0.285224722
ENSGALG00010025011	0.639147631	0.103407227
ENSGALG00010025012	0.488132209	0.641674675
ENSGALG00010025013	0.388360885	0.148933175
ENSGALG00010025015	0.414596716	0.066174972
ENSGALG00010025016	0.203991683	0.058221925
ENSGALG00010025018	0.364695497	0.432034705
ENSGALG00010025019	0.008533611	0.190617541
ENSGALG00010025020	0.089260919	0.269988056
ENSGALG00010025023	0.029758472	0.139135504
ENSGALG00010025024	0.802140703	0.136080927
ENSGALG00010025025	0.925025105	0.235413326
ENSGALG00010025026	0.95496316	0.362289263
ENSGALG00010025027	0.247162476	0.191578568
ENSGALG00010025028	0.94487128	0.410704628
ENSGALG00010025029	0.527460055	0.145518388
ENSGALG00010025030	0.620444896	0.325136552
ENSGALG00010025031	0.120618136	0.020476545
ENSGALG00010025032	0.8992302	0.421246412
ENSGALG00010025033	0.034060073	0.094660884
ENSGALG00010025034	0.119676382	0.177148554
ENSGALG00010025035	0.615121791	0.045816259
ENSGALG00010025036	0.176235095	0.78804764
ENSGALG00010025038	0.118115358	0.146883891
ENSGALG00010025039	0.979799472	0.274834712

ENSGALG00010025040	0.277566769	0.107857127
ENSGALG00010025041	0.115998335	0.068879627
ENSGALG00010025042	0.326352227	0.296872118
ENSGALG00010025043	0.830988298	0.122219019
ENSGALG00010025044	0.912876958	0.306891478
ENSGALG00010025045	0.93826793	0.301966349
ENSGALG00010025046	0.433139787	0.147713633
ENSGALG00010025047	0.346219757	0.266064828
ENSGALG00010025048	0.938999529	0.283475907
ENSGALG00010025050	0.01215542	0.094021119
ENSGALG00010025052	0.661313483	0.309061303
ENSGALG00010025054	0.351426963	0.019018966
ENSGALG00010025055	0.248496247	0.324614358
ENSGALG00010025056	0.798591824	0.294145386
ENSGALG00010025057	0.979371872	0.34156393
ENSGALG00010025058	0.62644523	0.055146778
ENSGALG00010025059	0.578875156	0.14867564
ENSGALG00010025060	0.384010335	0.305953376
ENSGALG00010025061	0.70849635	0.082078832
ENSGALG00010025063	0.808911008	0.285985632
ENSGALG00010025064	0.911871495	0.151148203
ENSGALG00010025065	0.410409955	0.23872141
ENSGALG00010025067	0.542424544	0.11274235
ENSGALG00010025069	0.895045434	0.363095152
ENSGALG00010025070	0.182570305	0.205924811
ENSGALG00010025071	0.812258376	0.394471426
ENSGALG00010025072	0.053133577	0.172599439
ENSGALG00010025073	0.726911459	0.241471036
ENSGALG00010025075	0.131284031	0.26489006
ENSGALG00010025076	0.211936166	0.274867062
ENSGALG00010025077	0.541459165	0.191502374
ENSGALG00010025078	0.785287896	0.46197732
ENSGALG00010025080	0.054305167	0.153573709
ENSGALG00010025085	0.375287285	0.114121296
ENSGALG00010025088	0.950494352	0.271744153
ENSGALG00010025089	0.430823118	0.12415925
ENSGALG00010025090	0.903266014	0.322486282
ENSGALG00010025091	0.5413914	0.215142031
ENSGALG00010025092	0.956935833	0.393448326
ENSGALG00010025093	0.00107637	0.201850013
ENSGALG00010025097	0.765789609	0.465072622
ENSGALG00010025098	0.747650063	0.305883581
ENSGALG00010025100	0.285179533	0.121954912
ENSGALG00010025102	0.932467293	0.391824454
ENSGALG00010025103	0.969523006	0.325377343
ENSGALG00010025105	0.295478522	0.152427427
ENSGALG00010025106	0.285272007	0.109169955
ENSGALG00010025108	0.938067483	0.326012235
ENSGALG00010025109	0.54967768	0.131646634
ENSGALG00010025110	0.208550923	0.676301294
ENSGALG00010025111	0.641662661	0.017630084
ENSGALG00010025113	0.816683242	0.229932364
ENSGALG00010025114	0.103296136	0.261817969
ENSGALG00010025115	0.141045986	0.011990063
ENSGALG00010025116	0.463628627	0.19876714
ENSGALG00010025117	0.824464571	0.363758749

ENSGALG00010025118	0.934166572	0.320295984
ENSGALG00010025119	0.308917405	0.210985547
ENSGALG00010025120	0.331509393	0.156730178
ENSGALG00010025121	0.531125511	0.005969025
ENSGALG00010025122	0.700242725	0.016395606
ENSGALG00010025125	0.939128589	0.228113341
ENSGALG00010025126	0.659342023	0.341351428
ENSGALG00010025127	0.558844302	0.350774258
ENSGALG00010025128	0.895231675	0.434100082
ENSGALG00010025129	0.784615346	0.461155679
ENSGALG00010025130	0.974523983	0.292311316
ENSGALG00010025131	0.408562239	0.236752053
ENSGALG00010025132	0.579389511	0.240277667
ENSGALG00010025133	0.626019989	0.195207285
ENSGALG00010025134	0.272241446	0.007471202
ENSGALG00010025136	0.254123497	0.181359519
ENSGALG00010025137	0.932110606	0.266105055
ENSGALG00010025138	0.643105708	0.008526398
ENSGALG00010025139	0.832618008	0.288275315
ENSGALG00010025140	0.597389611	0.224516766
ENSGALG00010025142	0.285272007	0.109169955
ENSGALG00010025144	0.233067903	0.095485992
ENSGALG00010025145	0.960478612	0.333930477
ENSGALG00010025146	0.549080842	0.002696549
ENSGALG00010025147	0.955097153	0.248241975
ENSGALG00010025148	0.22849409	0.039128681
ENSGALG00010025149	0.825674228	0.182073809
ENSGALG00010025151	0.369239878	0.007910514
ENSGALG00010025152	0.471110355	0.206108819
ENSGALG00010025154	0.909990022	0.324203814
ENSGALG00010025155	0.074070766	0.02191982
ENSGALG00010025156	0.142338824	0.01527242
ENSGALG00010025157	0.104816559	0.250895583
ENSGALG00010025158	0.400051822	0.226634549
ENSGALG00010025159	0.759379287	0.423081251
ENSGALG00010025160	0.896173353	0.130742478
ENSGALG00010025161	0.037103126	0.135840299
ENSGALG00010025162	0.124832317	0.132770016
ENSGALG00010025163	0.606458025	0.132483332
ENSGALG00010025164	0.820732978	0.252215337
ENSGALG00010025166	0.918680192	0.31724359
ENSGALG00010025167	0.688895072	0.068926328
ENSGALG00010025168	0.944505233	0.31879072
ENSGALG00010025169	0.352155798	0.161261905
ENSGALG00010025170	0.85161083	0.233044273
ENSGALG00010025171	0.447623677	0.1674086
ENSGALG00010025172	0.959909368	0.295464817
ENSGALG00010025173	0.474105198	0.267803845
ENSGALG00010025175	0.401669771	0.597749459
ENSGALG00010025176	0.856529302	0.277006138
ENSGALG00010025177	0.669511942	0.221787745
ENSGALG00010025178	0.596143543	0.056801663
ENSGALG00010025180	0.78384213	0.279262144
ENSGALG00010025181	0.264239718	0.073427965
ENSGALG00010025182	0.97214018	0.346721858
ENSGALG00010025186	0.722966722	0.158872402

ENSGALG00010025188	0.354188534	0.215413583
ENSGALG00010025189	0.615786921	0.117129476
ENSGALG00010025190	0.471309782	0.112372609
ENSGALG00010025191	0.166328932	0.250056524
ENSGALG00010025192	0.991233129	0.301767186
ENSGALG00010025193	0.671616678	0.459454274
ENSGALG00010025194	0.8662221	0.326823702
ENSGALG00010025195	0.93789494	0.411431514
ENSGALG00010025196	0.364675373	0.164356268
ENSGALG00010025198	0.551892743	0.134270135
ENSGALG00010025200	0.95041453	0.266028623
ENSGALG00010025201	0.270451009	0.090541667
ENSGALG00010025202	0.501750882	0.3280941
ENSGALG00010025204	0.637801164	0.465215962
ENSGALG00010025205	0.647023284	0.258725278
ENSGALG00010025206	0.319058215	0.168300175
ENSGALG00010025207	0.237498436	0.030269156
ENSGALG00010025208	0.65783448	0.190847689
ENSGALG00010025210	0.989476258	0.355972296
ENSGALG00010025211	0.647840576	0.195802818
ENSGALG00010025212	0.993811848	0.309227452
ENSGALG00010025213	0.027851816	0.14441112
ENSGALG00010025214	0.183557842	0.371817764
ENSGALG00010025215	0.993649079	0.323473086
ENSGALG00010025216	0.556862908	0.093918925
ENSGALG00010025217	0.380163374	0.082113198
ENSGALG00010025219	0.889999176	0.245707738
ENSGALG00010025220	0.044657074	0.54792616
ENSGALG00010025221	0.795839835	0.300271208
ENSGALG00010025222	0.959949614	0.2180522
ENSGALG00010025223	0.551605364	0.041865855
ENSGALG00010025224	0.789574558	0.310622715
ENSGALG00010025225	0.518638566	0.174634352
ENSGALG00010025226	0.218298356	0.216007457
ENSGALG00010025227	0.467901446	0.112482642
ENSGALG00010025228	0.665067107	0.128825674
ENSGALG00010025229	0.982450788	0.349181593
ENSGALG00010025230	0.74189509	0.258274872
ENSGALG00010025232	0.41723425	0.2668753
ENSGALG00010025233	0.902242025	0.181394855
ENSGALG00010025234	0.270451009	0.090541667
ENSGALG00010025236	0.97974652	0.294786813
ENSGALG00010025238	0.47205787	0.694646782
ENSGALG00010025239	0.74962802	0.219755167
ENSGALG00010025240	0.914811573	0.325707678
ENSGALG00010025241	0.299725404	0.140818236
ENSGALG00010025242	0.625556967	0.343361878
ENSGALG00010025243	0.882480937	0.246714122
ENSGALG00010025244	0.672149796	0.131484466
ENSGALG00010025245	0.098584232	0.127285755
ENSGALG00010025246	0.940744194	0.292236445
ENSGALG00010025247	0.989405612	0.335493533
ENSGALG00010025248	0.512295115	0.141251594
ENSGALG00010025249	0.160368076	0.256392964
ENSGALG00010025250	0.795273823	0.320125807
ENSGALG00010025251	0.21622191	0.257890352

ENSGALG00010025252	0.812585581	0.289956575
ENSGALG00010025253	0.874494789	0.185348702
ENSGALG00010025254	0.879640834	0.236524394
ENSGALG00010025255	0.970065039	0.300964339
ENSGALG00010025256	0.951438529	0.413603063
ENSGALG00010025257	0.753943234	0.31963332
ENSGALG00010025259	0.992394328	0.33356428
ENSGALG00010025260	0.065701053	0.184318944
ENSGALG00010025261	0.97841525	0.313281573
ENSGALG00010025262	0.792647849	0.272754367
ENSGALG00010025263	0.473451822	0.152307208
ENSGALG00010025266	0.990885372	0.373883686
ENSGALG00010025268	0.964121887	0.316462046
ENSGALG00010025270	0.956290562	0.309866791
ENSGALG00010025271	0.963745588	0.236777809
ENSGALG00010025272	0.546141018	0.083648566
ENSGALG00010025273	0.008113557	0.327426402
ENSGALG00010025274	0.942303971	0.295965261
ENSGALG00010025275	0.704219923	0.351388808
ENSGALG00010025276	0.635984177	0.043152307
ENSGALG00010025280	0.873569545	0.28172635
ENSGALG00010025281	0.29282415	0.184357635
ENSGALG00010025283	0.69768656	0.307521007
ENSGALG00010025284	0.045431216	0.019593835
ENSGALG00010025286	0.859584068	0.36952422
ENSGALG00010025287	0.385953062	0.133261917
ENSGALG00010025289	0.38481335	0.130001139
ENSGALG00010025291	0.44177723	0.367243954
ENSGALG00010025292	0.360268197	0.15773279
ENSGALG00010025297	0.920334944	0.278100938
ENSGALG00010025298	0.595166151	0.344886275
ENSGALG00010025300	0.670803337	0.290629249
ENSGALG00010025301	0.556914319	0.210195898
ENSGALG00010025303	0.745167477	0.124965585
ENSGALG00010025304	0.276354659	0.052375499
ENSGALG00010025305	0.903619199	0.488417181
ENSGALG00010025307	0.274586994	0.115798603
ENSGALG00010025309	0.934435794	0.35073784
ENSGALG00010025310	0.074228737	0.048703972
ENSGALG00010025311	0.120203364	0.135084415
ENSGALG00010025312	0.574689654	0.177440138
ENSGALG00010025313	0.970916728	0.287194656
ENSGALG00010025314	0.673287914	0.227064033
ENSGALG00010025315	0.981615677	0.370853916
ENSGALG00010025316	0.611904758	0.135748472
ENSGALG00010025317	0.37996605	0.103859099
ENSGALG00010025318	0.984193275	0.272541197
ENSGALG00010025319	0.703076018	0.217290375
ENSGALG00010025320	0.776014145	0.46019344
ENSGALG00010025322	0.757655916	0.241119181
ENSGALG00010025323	0.650415252	0.204626726
ENSGALG00010025324	0.975854273	0.324537646
ENSGALG00010025325	0.944399835	0.388215369
ENSGALG00010025327	0.792618975	0.263681095
ENSGALG00010025328	0.953983277	0.315460468
ENSGALG00010025329	0.645339514	0.25572833

ENSGALG00010025330	0.86668232	0.393750576
ENSGALG00010025331	0.87986544	0.224121239
ENSGALG00010025332	0.952705566	0.368248131
ENSGALG00010025333	0.437589781	0.265262432
ENSGALG00010025334	0.948958203	0.245787567
ENSGALG00010025335	0.954035957	0.348400155
ENSGALG00010025336	0.978605995	0.273342137
ENSGALG00010025337	0.780901265	0.072765555
ENSGALG00010025338	0.968786882	0.30217612
ENSGALG00010025339	0.981047547	0.344380355
ENSGALG00010025340	0.923177163	0.37284125
ENSGALG00010025341	0.557110473	0.143427403
ENSGALG00010025342	0.517851161	0.478907429
ENSGALG00010025343	0.846021729	0.493259074
ENSGALG00010025344	0.061990633	0.252050695
ENSGALG00010025345	0.6634547	0.098312917
ENSGALG00010025346	0.941358868	0.272589523
ENSGALG00010025348	0.872118364	0.329838158
ENSGALG00010025349	0.576452708	0.495827681
ENSGALG00010025350	0.051108153	0.173546243
ENSGALG00010025351	0.403734227	0.119578312
ENSGALG00010025353	0.976381968	0.30452238
ENSGALG00010025354	0.547453926	0.183060818
ENSGALG00010025355	0.955509993	0.321520269
ENSGALG00010025357	0.975248257	0.379708422
ENSGALG00010025358	0.667016845	0.192260114
ENSGALG00010025359	0.691700599	0.522972987
ENSGALG00010025360	0.975293694	0.343293374
ENSGALG00010025362	0.760785941	0.367243656
ENSGALG00010025363	0.266137114	0.107784983
ENSGALG00010025364	0.857013316	0.032855833
ENSGALG00010025365	0.978178677	0.288966315
ENSGALG00010025367	0.486762748	0.190685035
ENSGALG00010025368	0.939993908	0.226998859
ENSGALG00010025369	0.985561645	0.388968158
ENSGALG00010025370	0.986129337	0.318856667
ENSGALG00010025371	0.860262924	0.280818089
ENSGALG00010025373	0.715413808	0.136059729
ENSGALG00010025374	0.309297036	0.020926432
ENSGALG00010025376	0.590635679	0.15541149
ENSGALG00010025377	0.936266582	0.290809093
ENSGALG00010025378	0.925977157	0.128453585
ENSGALG00010025379	0.983254747	0.327522646
ENSGALG00010025380	0.197866827	0.027986115
ENSGALG00010025381	0.955610262	0.374517503
ENSGALG00010025382	0.53772341	0.2142241
ENSGALG00010025383	0.212956652	0.216908197
ENSGALG00010025384	0.943240016	0.246126177
ENSGALG00010025385	0.313094935	0.224123301
ENSGALG00010025386	0.95332442	0.379464924
ENSGALG00010025387	0.689405423	0.326608205
ENSGALG00010025389	0.90857459	0.185361579
ENSGALG00010025390	0.964808944	0.399454972
ENSGALG00010025392	0.001330564	0.275812528
ENSGALG00010025394	0.899339307	0.405143446
ENSGALG00010025395	0.675869829	0.283579069

ENSGALG00010025396	0.394327047	0.133628127
ENSGALG00010025398	0.559567353	0.265250553
ENSGALG00010025399	0.187514554	0.108479243
ENSGALG00010025400	0.953263162	0.316920027
ENSGALG00010025403	0.925521534	0.360534218
ENSGALG00010025404	0.488537825	0.227100959
ENSGALG00010025405	0.936675435	0.213462662
ENSGALG00010025406	0.914497786	0.308721383
ENSGALG00010025407	0.822427983	0.26263614
ENSGALG00010025410	0.14323154	0.195038989
ENSGALG00010025411	0.127541883	0.101503614
ENSGALG00010025413	0.904749576	0.274029245
ENSGALG00010025414	0.92168626	0.452508826
ENSGALG00010025415	0.949982297	0.328450408
ENSGALG00010025416	0.724434269	0.363229366
ENSGALG00010025417	0.870932456	0.339738147
ENSGALG00010025418	0.986482301	0.332237443
ENSGALG00010025419	0.915234631	0.256609005
ENSGALG00010025420	0.774955436	0.188626076
ENSGALG00010025421	0.97564244	0.329170689
ENSGALG00010025422	0.616932816	0.209491807
ENSGALG00010025423	0.862640765	0.19091262
ENSGALG00010025424	0.245700908	0.010899108
ENSGALG00010025425	0.408545516	0.270441321
ENSGALG00010025426	0.895578048	0.201960399
ENSGALG00010025427	0.933696204	0.377496103
ENSGALG00010025428	0.953430775	0.236958831
ENSGALG00010025429	0.328743492	0.129197287
ENSGALG00010025431	0.111816492	0.088629934
ENSGALG00010025432	0.601988976	0.00950416
ENSGALG00010025433	0.941720455	0.322825692
ENSGALG00010025434	0.677019532	0.253534204
ENSGALG00010025435	0.899184442	0.421181456
ENSGALG00010025436	0.265558089	0.028821141
ENSGALG00010025437	0.311398252	0.127905775
ENSGALG00010025438	0.901582935	0.378347282
ENSGALG00010025440	0.962705774	0.364216645
ENSGALG00010025442	0.993997467	0.319162593
ENSGALG00010025444	0.869416989	0.296891146
ENSGALG00010025445	0.860261955	0.455631464
ENSGALG00010025446	0.024864555	0.352909512
ENSGALG00010025447	0.523092456	0.640312765
ENSGALG00010025448	0.61646547	0.2533504
ENSGALG00010025449	0.652389385	0.202034898
ENSGALG00010025451	0.105273261	0.02311766
ENSGALG00010025452	0.665100665	0.388473743
ENSGALG00010025454	0.394271748	0.144488011
ENSGALG00010025455	0.954442394	0.376422205
ENSGALG00010025456	0.844930195	0.355639006
ENSGALG00010025458	0.524649134	0.064551918
ENSGALG00010025459	0.632842372	0.141045055
ENSGALG00010025460	0.661245705	0.29296673
ENSGALG00010025463	0.648118454	0.104239374
ENSGALG00010025464	0.620381441	0.130315556
ENSGALG00010025465	0.953428033	0.203403866
ENSGALG00010025466	0.020656918	0.064774402

ENSGALG00010025470	0.250982019	0.138810371
ENSGALG00010025471	0.319320077	0.024051854
ENSGALG00010025472	0.714144873	0.331339269
ENSGALG00010025473	0.254854622	0.325784632
ENSGALG00010025476	0.44250459	0.194406598
ENSGALG00010025477	0.843332981	0.296047489
ENSGALG00010025478	0.203991683	0.058221925
ENSGALG00010025479	0.256828349	0.119064955
ENSGALG00010025480	0.364118301	0.144929949
ENSGALG00010025481	0.933111417	0.306017159
ENSGALG00010025483	0.977600694	0.280956896
ENSGALG00010025485	0.932512267	0.329240771
ENSGALG00010025486	0.889428576	0.268166304
ENSGALG00010025488	0.228167445	0.174508845
ENSGALG00010025490	0.480317276	0.038215845
ENSGALG00010025491	0.978652175	0.341512959
ENSGALG00010025492	0.134010064	0.201768367
ENSGALG00010025493	0.655946165	0.343142924
ENSGALG00010025494	0.285272007	0.109169955
ENSGALG00010025496	0.977706643	0.34482428
ENSGALG00010025499	0.996412146	0.327278646
ENSGALG00010025504	0.995496304	0.335472491
ENSGALG00010025505	0.502421136	0.002727107
ENSGALG00010025506	0.960858715	0.296680085
ENSGALG00010025508	0.371363241	0.262803777
ENSGALG00010025509	0.379969153	0.146014072
ENSGALG00010025511	0.271701261	0.072417625
ENSGALG00010025512	0.336529924	0.338246544
ENSGALG00010025513	0.427383873	0.389429401
ENSGALG00010025515	0.425533569	0.152495024
ENSGALG00010025516	0.856849604	0.341451921
ENSGALG00010025517	0.779121322	0.325614795
ENSGALG00010025520	0.618166591	0.030526867
ENSGALG00010025522	0.02356128	0.063279562
ENSGALG00010025523	0.479418787	0.111787351
ENSGALG00010025524	0.265558089	0.028821141
ENSGALG00010025527	0.756331681	0.263315629
ENSGALG00010025530	0.315020464	0.160576986
ENSGALG00010025531	0.935701388	0.425824021
ENSGALG00010025532	0.112701073	0.170190663
ENSGALG00010025533	0.964363091	0.429008421
ENSGALG00010025535	0.555221782	0.223462608
ENSGALG00010025536	0.489745363	0.210530503
ENSGALG00010025537	0.501612846	0.160124173
ENSGALG00010025538	0.34118751	0.032724182
ENSGALG00010025541	0.634657014	0.064510317
ENSGALG00010025542	0.474658292	0.023443461
ENSGALG00010025543	0.601907254	0.313795732
ENSGALG00010025544	0.981804653	0.269706078
ENSGALG00010025545	0.976634722	0.193956702
ENSGALG00010025546	0.975189082	0.34372375
ENSGALG00010025547	0.83679314	0.293038668
ENSGALG00010025548	0.54832323	0.224399958
ENSGALG00010025549	0.911207582	0.233840833
ENSGALG00010025553	0.503366675	0.147030659
ENSGALG00010025554	0.506759125	0.137777757

ENSGALG00010025555	0.942397469	0.380789102
ENSGALG00010025556	0.417333311	0.169634281
ENSGALG00010025557	0.950532109	0.397124603
ENSGALG00010025558	0.778987991	0.216552077
ENSGALG00010025560	0.293185829	0.256216488
ENSGALG00010025561	0.387659376	0.117915307
ENSGALG00010025562	0.770713167	0.176147492
ENSGALG00010025563	0.354294363	0.32433785
ENSGALG00010025564	0.287306084	0.323228033
ENSGALG00010025565	0.386506079	0.168170232
ENSGALG00010025567	0.482282027	0.149449428
ENSGALG00010025568	0.499238288	0.39137666
ENSGALG00010025569	0.308093876	0.191573855
ENSGALG00010025570	0.189240572	0.04996112
ENSGALG00010025571	0.539180348	0.463613076
ENSGALG00010025572	0.038114492	0.060138221
ENSGALG00010025573	0.87765187	0.411114659
ENSGALG00010025574	0.724811224	0.104098157
ENSGALG00010025575	0.467679419	0.440970569
ENSGALG00010025576	0.243917537	0.031017863
ENSGALG00010025577	0.971690859	0.390299605
ENSGALG00010025578	0.827481711	0.335969721
ENSGALG00010025579	0.769902836	0.035092762
ENSGALG00010025580	0.935710989	0.411394636
ENSGALG00010025581	0.39155441	0.07460765
ENSGALG00010025582	0.784462464	0.162015247
ENSGALG00010025583	0.198196562	0.151040066
ENSGALG00010025585	0.159052054	0.177497987
ENSGALG00010025586	0.05566616	0.236883668
ENSGALG00010025587	0.829522192	0.375301092
ENSGALG00010025588	0.596446383	0.247898115
ENSGALG00010025589	0.16370231	0.222721419
ENSGALG00010025590	0.869212398	0.323197453
ENSGALG00010025591	0.610714521	0.345085022
ENSGALG00010025592	0.81079556	0.262765646
ENSGALG00010025594	0.250503259	0.171342079
ENSGALG00010025595	0.957484185	0.362267032
ENSGALG00010025597	0.208908584	0.09677408
ENSGALG00010025598	0.427284053	0.512993905
ENSGALG00010025599	0.978573302	0.323420126
ENSGALG00010025600	0.760421295	0.304759964
ENSGALG00010025601	0.333496467	0.22479672
ENSGALG00010025604	0.677832206	0.198411175
ENSGALG00010025605	0.264239718	0.073427965
ENSGALG00010025606	0.817990243	0.133745564
ENSGALG00010025607	0.712237583	0.100187794
ENSGALG00010025608	0.388000333	0.289740802
ENSGALG00010025609	0.277566769	0.107857127
ENSGALG00010025610	0.257317102	0.20095426
ENSGALG00010025611	0.871956531	0.294149446
ENSGALG00010025612	0.793028663	0.47651601
ENSGALG00010025613	0.909559466	0.329958504
ENSGALG00010025614	0.51803537	0.00193194
ENSGALG00010025617	0.309488172	0.159626796
ENSGALG00010025618	0.989562192	0.364287411
ENSGALG00010025621	0.855758328	0.360710533

ENSGALG00010025623	0.91737847	0.605700787
ENSGALG00010025624	0.111491635	0.302461543
ENSGALG00010025628	0.29212014	0.404766674
ENSGALG00010025629	0.954666411	0.264607967
ENSGALG00010025632	0.098176012	0.230786181
ENSGALG00010025633	0.975932792	0.332599367
ENSGALG00010025634	0.723138806	0.090943541
ENSGALG00010025635	0.947797965	0.250208631
ENSGALG00010025636	0.469547605	0.111209253
ENSGALG00010025637	0.991261705	0.270677475
ENSGALG00010025638	0.595846964	0.243043205
ENSGALG00010025639	0.42873774	0.191369203
ENSGALG00010025640	0.759455879	0.227233532
ENSGALG00010025641	0.445381203	0.017983906
ENSGALG00010025642	0.811627412	0.31500232
ENSGALG00010025643	0.044986643	0.113504759
ENSGALG00010025644	0.247131793	0.284383911
ENSGALG00010025645	0.961212125	0.329665575
ENSGALG00010025646	0.361724536	0.300421964
ENSGALG00010025647	0.660096532	0.192525209
ENSGALG00010025648	0.954511811	0.218659196
ENSGALG00010025649	0.326003616	0.301008457
ENSGALG00010025650	0.961638347	0.291183972
ENSGALG00010025652	0.867103479	0.453492582
ENSGALG00010025653	0.916207647	0.234206381
ENSGALG00010025654	0.975043955	0.269956054
ENSGALG00010025655	0.027351409	0.220055346
ENSGALG00010025657	0.877709958	0.118928864
ENSGALG00010025658	0.905157502	0.317085728
ENSGALG00010025659	0.970788093	0.303035377
ENSGALG00010025660	0.69632177	0.124189822
ENSGALG00010025661	0.936397477	0.324005718
ENSGALG00010025663	0.463235288	0.115149902
ENSGALG00010025664	0.888825786	0.076763241
ENSGALG00010025665	0.580960191	0.221984986
ENSGALG00010025666	0.983267396	0.383364664
ENSGALG00010025668	0.888194465	0.271185001
ENSGALG00010025671	0.984678595	0.347142111
ENSGALG00010025672	0.446756698	0.055080262
ENSGALG00010025673	0.954511574	0.24347901
ENSGALG00010025674	0.920753256	0.236968002
ENSGALG00010025675	0.031329216	0.144699932
ENSGALG00010025678	0.261521937	0.275508417
ENSGALG00010025681	0.852282678	0.468446352
ENSGALG00010025684	0.882264674	0.187888346
ENSGALG00010025685	0.951031726	0.295014549
ENSGALG00010025687	0.482518465	0.063868665
ENSGALG00010025688	0.412716415	0.03873236
ENSGALG00010025689	0.481103591	0.067111283
ENSGALG00010025691	0.612098934	0.03374001
ENSGALG00010025692	0.826178942	0.230382445
ENSGALG00010025693	0.758879358	0.168235041
ENSGALG00010025696	0.924677134	0.282991474
ENSGALG00010025698	0.277566769	0.107857127
ENSGALG00010025699	0.769468925	0.361032123
ENSGALG00010025702	0.604372131	0.20468805

ENSGALG00010025703	0.808753474	0.02208857
ENSGALG00010025704	0.329744371	0.371370966
ENSGALG00010025705	0.877664146	0.286008002
ENSGALG00010025708	0.886607486	0.04814033
ENSGALG00010025712	0.797210278	0.127171789
ENSGALG00010025713	0.649479105	0.348539741
ENSGALG00010025714	0.319311594	0.353226242
ENSGALG00010025716	0.24825154	0.586610038
ENSGALG00010025717	0.922027122	0.410116659
ENSGALG00010025720	0.970841823	0.257012643
ENSGALG00010025723	0.743931467	0.196103509
ENSGALG00010025727	0.864372139	0.228437055
ENSGALG00010025731	0.948038447	0.304798918
ENSGALG00010025733	0.016763015	0.076327924
ENSGALG00010025734	0.419514768	0.2613498
ENSGALG00010025735	0.441297911	0.226728942
ENSGALG00010025736	0.778076799	0.443698137
ENSGALG00010025737	0.971431329	0.228674199
ENSGALG00010025738	0.53402376	0.081083233
ENSGALG00010025739	0.375927362	0.072713138
ENSGALG00010025740	0.103562557	0.343273643
ENSGALG00010025741	0.39190877	0.131701825
ENSGALG00010025742	0.980202079	0.352848275
ENSGALG00010025743	0.949274095	0.250163998
ENSGALG00010025744	0.275740267	0.144739672
ENSGALG00010025745	0.983245581	0.319871146
ENSGALG00010025747	0.489688642	0.142768469
ENSGALG00010025748	0.923756663	0.444853608
ENSGALG00010025750	0.948741924	0.305683774
ENSGALG00010025753	0.987540831	0.31345112
ENSGALG00010025755	0.798467054	0.321109259
ENSGALG00010025756	0.353313305	0.24075221
ENSGALG00010025758	0.101653335	0.142607874
ENSGALG00010025763	0.386661145	0.092431087
ENSGALG00010025766	0.473932792	0.434932788
ENSGALG00010025767	0.44214781	0.148485418
ENSGALG00010025769	0.293985632	0.14519096
ENSGALG00010025770	0.329713792	0.203369349
ENSGALG00010025771	0.270451009	0.090541667
ENSGALG00010025772	0.379394869	0.170237467
ENSGALG00010025774	0.228836647	0.070265399
ENSGALG00010025775	0.052836869	0.101827534
ENSGALG00010025776	0.114803004	0.15756782
ENSGALG00010025781	0.125104501	0.078185614
ENSGALG00010025785	0.936020627	0.386104493
ENSGALG00010025786	0.539710938	0.307598517
ENSGALG00010025787	0.748030983	0.486251314
ENSGALG00010025788	0.490726529	0.122373879
ENSGALG00010025789	0.785178061	0.224148124
ENSGALG00010025790	0.957774242	0.33653173
ENSGALG00010025791	0.712405576	0.310939724
ENSGALG00010025793	0.597269398	0.160997067
ENSGALG00010025796	0.341269885	0.029948769
ENSGALG00010025798	0.30407317	0.400998254
ENSGALG00010025799	0.899637482	0.28990369
ENSGALG00010025800	0.515721984	0.567269421

ENSGALG00010025801	0.78500674	0.117548414
ENSGALG00010025802	0.354205285	0.12892787
ENSGALG00010025805	0.830058728	0.264806513
ENSGALG00010025806	0.947700121	0.143019741
ENSGALG00010025809	0.734910658	0.41954087
ENSGALG00010025810	0.070197589	0.372408104
ENSGALG00010025811	0.348032803	0.146426442
ENSGALG00010025815	0.912480374	0.371598186
ENSGALG00010025817	0.355639478	0.141537604
ENSGALG00010025820	0.270451009	0.090541667
ENSGALG00010025822	0.851856135	0.089007078
ENSGALG00010025824	0.595232013	0.042886564
ENSGALG00010025825	0.199041595	0.200784023
ENSGALG00010025826	0.290515358	0.070869158
ENSGALG00010025828	0.647850157	0.302984245
ENSGALG00010025829	0.454143018	0.291641342
ENSGALG00010025830	0.966787429	0.352517077
ENSGALG00010025831	0.884880918	0.244338758
ENSGALG00010025832	0.834401424	0.528013399
ENSGALG00010025833	0.944006856	0.253210231
ENSGALG00010025835	0.919332334	0.454547054
ENSGALG00010025836	0.94539839	0.3796478
ENSGALG00010025837	0.455491161	0.002055857
ENSGALG00010025838	0.348165477	0.035563623
ENSGALG00010025839	0.939275016	0.248181833
ENSGALG00010025843	0.863676758	0.342582847
ENSGALG00010025844	0.670532637	0.042565208
ENSGALG00010025845	0.539193948	0.2152994
ENSGALG00010025846	0.038165923	0.10358575
ENSGALG00010025847	0.882975952	0.18400148
ENSGALG00010025848	0.174596352	0.460105186
ENSGALG00010025849	0.99391261	0.321728716
ENSGALG00010025850	0.175960236	0.195005168
ENSGALG00010025851	0.000893823	0.500599188
ENSGALG00010025852	0.668537827	0.481461662
ENSGALG00010025854	0.212199218	0.180143923
ENSGALG00010025856	0.403349636	0.298877835
ENSGALG00010025857	0.857812885	0.260769396
ENSGALG00010025860	0.676335277	0.31057721
ENSGALG00010025861	0.782360091	0.02258801
ENSGALG00010025862	0.538039428	0.125690459
ENSGALG00010025863	0.204589043	0.218713816
ENSGALG00010025864	0.81633546	0.415676695
ENSGALG00010025865	0.265555066	0.192319121
ENSGALG00010025866	0.099242338	0.132709837
ENSGALG00010025867	0.058751725	0.152800327
ENSGALG00010025869	0.873518073	0.315852203
ENSGALG00010025870	0.301280706	0.449879566
ENSGALG00010025872	0.957516422	0.415703228
ENSGALG00010025874	0.641905225	0.466989187
ENSGALG00010025880	0.946599985	0.454522468
ENSGALG00010025881	0.675088565	0.178540007
ENSGALG00010025882	0.906459459	0.360552123
ENSGALG00010025883	0.538073254	0.132130203
ENSGALG00010025886	0.047989055	0.114544364
ENSGALG00010025887	0.34875575	0.37938357

ENSGALG00010025888	0.102937049	0.231959204
ENSGALG00010025889	0.958064568	0.362700408
ENSGALG00010025890	0.900670861	0.391869239
ENSGALG00010025891	0.68770806	0.439847158
ENSGALG00010025892	0.834906674	0.447211998
ENSGALG00010025894	0.02931309	0.104495807
ENSGALG00010025896	0.187514554	0.108479243
ENSGALG00010025897	0.959967841	0.287660438
ENSGALG00010025899	0.870141156	0.322945307
ENSGALG00010025900	0.404186295	0.077281686
ENSGALG00010025903	0.129889745	0.169631389
ENSGALG00010025904	0.832537526	0.309615779
ENSGALG00010025905	0.981552618	0.347064609
ENSGALG00010025906	0.847888971	0.487179621
ENSGALG00010025907	0.914465119	0.286103745
ENSGALG00010025908	0.360407633	0.310955723
ENSGALG00010025909	0.260203497	0.242950444
ENSGALG00010025910	0.92965732	0.20685052
ENSGALG00010025911	0.068572699	0.253569288
ENSGALG00010025912	0.168321586	0.146624045
ENSGALG00010025913	0.972330396	0.25351719
ENSGALG00010025914	0.559627486	0.147134965
ENSGALG00010025917	0.971811831	0.365275061
ENSGALG00010025918	0.451631885	0.333966577
ENSGALG00010025919	0.480030643	0.186463434
ENSGALG00010025920	0.780212031	0.463565837
ENSGALG00010025922	0.384165008	0.165101073
ENSGALG00010025923	0.343522853	0.200260068
ENSGALG00010025924	0.780305009	0.179313781
ENSGALG00010025925	0.366349475	0.082494236
ENSGALG00010025926	0.478023857	0.258352166
ENSGALG00010025928	0.554520669	0.021827879
ENSGALG00010025929	0.907410697	0.248970532
ENSGALG00010025930	0.742133042	0.217009887
ENSGALG00010025931	0.826962648	0.286057345
ENSGALG00010025932	0.973359165	0.242863688
ENSGALG00010025933	0.979100401	0.291106016
ENSGALG00010025934	0.07108268	0.297262155
ENSGALG00010025935	0.563215423	0.097885273
ENSGALG00010025936	0.907935387	0.221989655
ENSGALG00010025937	0.861789741	0.317552219
ENSGALG00010025938	0.960140638	0.310399016
ENSGALG00010025939	0.247406418	0.154863212
ENSGALG00010025940	0.367011271	0.244011328
ENSGALG00010025941	0.590220505	0.228803027
ENSGALG00010025943	0.889945584	0.214194937
ENSGALG00010025945	0.574273966	0.219839548
ENSGALG00010025946	0.309152578	0.416634701
ENSGALG00010025949	0.714700956	0.312365395
ENSGALG00010025950	0.671640244	0.191813156
ENSGALG00010025951	0.882190548	0.257361014
ENSGALG00010025952	0.947109327	0.32518133
ENSGALG00010025954	0.584914176	0.014182997
ENSGALG00010025955	0.199586067	0.015517605
ENSGALG00010025956	0.900892708	0.255879081
ENSGALG00010025957	0.989118895	0.307524083

ENSGALG00010025958	0.696714708	0.02108306
ENSGALG00010025959	0.063693293	0.122451768
ENSGALG00010025960	0.940286284	0.317911325
ENSGALG00010025961	0.210381064	0.122626751
ENSGALG00010025962	0.741366582	0.02889454
ENSGALG00010025963	0.982004917	0.312771073
ENSGALG00010025965	0.055573343	0.49874354
ENSGALG00010025966	0.003830079	0.379623043
ENSGALG00010025968	0.990028283	0.297240404
ENSGALG00010025969	0.283012089	0.206673807
ENSGALG00010025970	0.940709949	0.311647297
ENSGALG00010025971	0.968213203	0.334778335
ENSGALG00010025972	0.179797963	0.163193665
ENSGALG00010025973	0.879583975	0.395517891
ENSGALG00010025974	0.943357507	0.306243279
ENSGALG00010025975	0.20823267	0.068514942
ENSGALG00010025976	0.046708758	0.290796188
ENSGALG00010025977	0.870684843	0.191365601
ENSGALG00010025978	0.052836869	0.101827534
ENSGALG00010025979	0.949616221	0.26370202
ENSGALG00010025980	0.965613546	0.349673743
ENSGALG00010025981	0.057588202	0.120194332
ENSGALG00010025982	0.080096649	0.231139376
ENSGALG00010025983	0.885325272	0.029465539
ENSGALG00010025984	0.860785337	0.30539625
ENSGALG00010025985	0.311726247	0.083677434
ENSGALG00010025986	0.932613564	0.328412586
ENSGALG00010025988	0.825726884	0.335977741
ENSGALG00010025990	0.442184346	0.175638411
ENSGALG00010025991	0.367295315	0.457889722
ENSGALG00010025992	0.705832487	0.375101214
ENSGALG00010025993	0.308600579	0.142811058
ENSGALG00010025994	0.879104721	0.26470511
ENSGALG00010025995	0.948042524	0.384135636
ENSGALG00010025996	0.996377386	0.306274094
ENSGALG00010025997	0.966453521	0.225412671
ENSGALG00010025998	0.098948842	0.344731271
ENSGALG00010025999	0.566877157	0.318571238
ENSGALG00010026000	0.430396274	0.163132962
ENSGALG00010026001	0.118027285	0.023079149
ENSGALG00010026002	0.835995065	0.284265156
ENSGALG00010026003	0.805140081	0.007945764
ENSGALG00010026004	0.381415582	0.158761082
ENSGALG00010026005	0.916159629	0.296357135
ENSGALG00010026006	0.464757457	0.084879844
ENSGALG00010026007	0.460363179	0.196807947
ENSGALG00010026008	0.595963434	0.096814938
ENSGALG00010026009	0.908191008	0.038480022
ENSGALG00010026010	0.931499445	0.202088546
ENSGALG00010026011	0.745032148	0.412465477
ENSGALG00010026013	0.965971079	0.391285518
ENSGALG00010026015	0.11966357	0.272919694
ENSGALG00010026016	0.897972284	0.294745885
ENSGALG00010026018	0.245430813	0.175511689
ENSGALG00010026019	0.467720654	0.324622962
ENSGALG00010026021	0.212146324	0.27647893

ENSGALG00010026023	0.958635198	0.377037058
ENSGALG00010026026	0.405071667	0.170520944
ENSGALG00010026027	0.901014717	0.20736042
ENSGALG00010026030	0.593907031	0.113729652
ENSGALG00010026031	0.616488709	0.089788433
ENSGALG00010026033	0.891583497	0.296246811
ENSGALG00010026034	0.27125251	0.180804602
ENSGALG00010026035	0.327798841	0.049503364
ENSGALG00010026036	0.739377072	0.164367494
ENSGALG00010026037	0.266137114	0.107784983
ENSGALG00010026038	0.395556858	0.102965232
ENSGALG00010026040	0.943471936	0.304690506
ENSGALG00010026041	0.528687065	0.431812039
ENSGALG00010026042	0.02436834	0.087960992
ENSGALG00010026043	0.39276112	0.274252474
ENSGALG00010026044	0.690492776	0.506179969
ENSGALG00010026045	0.944421526	0.349923239
ENSGALG00010026046	0.780404524	0.22429707
ENSGALG00010026051	0.897754269	0.352874572
ENSGALG00010026053	0.510458542	0.009963926
ENSGALG00010026056	0.403610966	0.309898237
ENSGALG00010026058	0.097513339	0.023967495
ENSGALG00010026059	0.987197197	0.366180821
ENSGALG00010026060	0.467875361	0.023091982
ENSGALG00010026061	0.864151599	0.366805829
ENSGALG00010026062	0.948398883	0.220824103
ENSGALG00010026063	0.887963455	0.203975301
ENSGALG00010026064	0.940690137	0.254897417
ENSGALG00010026066	0.978289576	0.252016137
ENSGALG00010026068	0.857619197	0.278976458
ENSGALG00010026069	0.990377185	0.355987957
ENSGALG00010026070	0.31383023	0.338454411
ENSGALG00010026073	0.765995143	0.111564953
ENSGALG00010026076	0.904922726	0.255588174
ENSGALG00010026078	0.98614277	0.302853919
ENSGALG00010026079	0.677733381	0.270816745
ENSGALG00010026081	0.872613739	0.388525745
ENSGALG00010026083	0.821859445	0.378318399
ENSGALG00010026085	0.114043601	0.287895224
ENSGALG00010026086	0.359730054	0.159389574
ENSGALG00010026087	0.939584143	0.216976082
ENSGALG00010026088	0.968899685	0.23398072
ENSGALG00010026089	0.977024524	0.318279355
ENSGALG00010026090	0.98736338	0.354701973
ENSGALG00010026091	7.00E-01	5.24E-05
ENSGALG00010026092	0.547167668	0.150577659
ENSGALG00010026093	0.83487461	0.307206688
ENSGALG00010026094	0.838030321	0.263868101
ENSGALG00010026095	0.848400042	0.312607674
ENSGALG00010026097	0.459205388	0.305516661
ENSGALG00010026098	0.902980501	0.283162219
ENSGALG00010026099	0.099101311	0.375967491
ENSGALG00010026100	0.068004522	0.318885568
ENSGALG00010026101	0.059650661	0.385152578
ENSGALG00010026102	0.689032312	0.262972367
ENSGALG00010026103	0.570937068	0.276092743

ENSGALG00010026104	0.946031212	0.282044541
ENSGALG00010026105	0.733169866	0.157631584
ENSGALG00010026106	0.569198987	0.221814102
ENSGALG00010026107	0.402060142	0.145911736
ENSGALG00010026108	0.20979263	0.06535578
ENSGALG00010026109	0.142268032	0.383513863
ENSGALG00010026111	0.152839347	0.032980537
ENSGALG00010026112	0.988763922	0.26469499
ENSGALG00010026113	0.858986656	0.18559086
ENSGALG00010026114	0.942879339	0.395332529
ENSGALG00010026115	0.922174647	0.296496653
ENSGALG00010026116	0.238115312	0.800944552
ENSGALG00010026117	0.701174695	0.657340456
ENSGALG00010026120	0.661306668	0.404767329
ENSGALG00010026121	0.843572655	0.175183002
ENSGALG00010026122	0.431831647	0.199512818
ENSGALG00010026124	0.047117039	0.095493806
ENSGALG00010026125	0.187221887	0.16859055
ENSGALG00010026126	0.258087249	0.123701198
ENSGALG00010026127	0.728800034	0.395832485
ENSGALG00010026128	0.985216973	0.312887193
ENSGALG00010026130	0.385392063	0.133931937
ENSGALG00010026131	0.295817771	0.175286565
ENSGALG00010026132	0.979477284	0.34883989
ENSGALG00010026133	0.897378512	0.391602176
ENSGALG00010026135	0.270451009	0.090541667
ENSGALG00010026137	0.932226692	0.31944971
ENSGALG00010026143	0.880178983	0.167528969
ENSGALG00010026145	0.204394796	0.384273876
ENSGALG00010026148	0.910334375	0.295088521
ENSGALG00010026149	0.81608809	0.069698708
ENSGALG00010026151	0.8805117	0.382301971
ENSGALG00010026154	0.869638821	0.403210659
ENSGALG00010026155	0.277566769	0.107857127
ENSGALG00010026156	0.906072768	0.269659227
ENSGALG00010026159	0.943361119	0.376945104
ENSGALG00010026162	0.65494678	0.280658559
ENSGALG00010026163	0.964586306	0.288974471
ENSGALG00010026165	0.518638737	0.197697833
ENSGALG00010026166	0.741715828	0.133357132
ENSGALG00010026167	0.825026827	0.338049956
ENSGALG00010026170	0.10010901	0.111729267
ENSGALG00010026171	0.264239718	0.073427965
ENSGALG00010026172	0.069215939	0.312787575
ENSGALG00010026173	0.487397397	0.059643958
ENSGALG00010026174	0.006684895	0.161510246
ENSGALG00010026176	0.388876865	0.186701289
ENSGALG00010026177	0.762260386	0.208290321
ENSGALG00010026183	0.857206263	0.278175807
ENSGALG00010026184	0.991640899	0.334345149
ENSGALG00010026186	0.391097471	0.144336359
ENSGALG00010026187	0.002953388	0.313313918
ENSGALG00010026188	0.504166556	0.007180957
ENSGALG00010026189	0.494986208	0.164505697
ENSGALG00010026190	0.373668088	0.160332228
ENSGALG00010026191	0.171048961	0.278010478

ENSGALG00010026192	0.988452028	0.381074846
ENSGALG00010026193	0.252804965	0.381929009
ENSGALG00010026195	0.901275807	0.319796629
ENSGALG00010026196	0.451685302	0.411064578
ENSGALG00010026199	0.886449478	0.237585089
ENSGALG00010026202	0.933626744	0.410713281
ENSGALG00010026203	0.657022453	0.521619407
ENSGALG00010026205	0.992473198	0.289788206
ENSGALG00010026208	0.392329638	0.175828901
ENSGALG00010026209	0.9810841	0.322570182
ENSGALG00010026210	0.245700908	0.010899108
ENSGALG00010026211	0.25542332	0.219823953
ENSGALG00010026213	0.949710079	0.332034082
ENSGALG00010026214	0.952582229	0.307917922
ENSGALG00010026215	0.286977485	0.247439799
ENSGALG00010026216	0.82841021	0.303285128
ENSGALG00010026217	0.526981051	0.20452168
ENSGALG00010026218	0.872200791	0.255825279
ENSGALG00010026219	0.942755076	0.347499484
ENSGALG00010026220	0.95948404	0.399843418
ENSGALG00010026221	0.971868884	0.332860396
ENSGALG00010026223	0.626123579	0.182683871
ENSGALG00010026224	0.953631566	0.324750931
ENSGALG00010026225	0.410675159	0.384532076
ENSGALG00010026226	0.942860282	0.309207449
ENSGALG00010026229	0.3374524	0.16253848
ENSGALG00010026232	0.982806177	0.313271071
ENSGALG00010026233	0.065517623	0.014192041
ENSGALG00010026234	0.956691671	0.298906183
ENSGALG00010026235	0.973166857	0.23655297
ENSGALG00010026236	0.325957356	0.610236796
ENSGALG00010026237	0.978640368	0.307108801
ENSGALG00010026238	0.095473757	0.237416683
ENSGALG00010026239	0.504573231	0.197114758
ENSGALG00010026240	0.041532654	0.541509146
ENSGALG00010026241	0.946679813	0.231498074
ENSGALG00010026242	0.405687968	0.171428318
ENSGALG00010026244	0.515539735	0.365915727
ENSGALG00010026246	0.847941077	0.139598941
ENSGALG00010026249	0.305300423	0.100923143
ENSGALG00010026250	0.824442609	0.269732527
ENSGALG00010026252	0.461240722	0.292501289
ENSGALG00010026253	0.178751709	0.427200959
ENSGALG00010026255	0.203250927	0.146016153
ENSGALG00010026256	0.017362381	0.009256585
ENSGALG00010026258	0.670391699	0.228627598
ENSGALG00010026259	0.943769854	0.287217799
ENSGALG00010026261	0.939334253	0.361171748
ENSGALG00010026262	0.769931077	0.421337598
ENSGALG00010026264	0.05484482	0.109033392
ENSGALG00010026266	0.419232156	0.016158533
ENSGALG00010026267	0.277566769	0.107857127
ENSGALG00010026268	0.813528607	0.244943411
ENSGALG00010026269	0.394927857	0.119113854
ENSGALG00010026272	0.961962585	0.309233742
ENSGALG00010026277	0.945547001	0.283727704

ENSGALG00010026282	0.247203048	0.342104972
ENSGALG00010026283	0.934501459	0.248505022
ENSGALG00010026284	0.153212822	0.121645841
ENSGALG00010026287	0.520477459	0.105672889
ENSGALG00010026289	0.957099034	0.285540196
ENSGALG00010026290	0.258778752	0.242330282
ENSGALG00010026291	0.977082035	0.32010623
ENSGALG00010026292	0.791941507	0.430891537
ENSGALG00010026293	0.958525662	0.313096245
ENSGALG00010026295	0.923798348	0.393829432
ENSGALG00010026296	0.98270843	0.235863883
ENSGALG00010026297	0.914874755	0.321767339
ENSGALG00010026298	0.97364899	0.301953463
ENSGALG00010026300	0.922750027	0.37229541
ENSGALG00010026301	0.445934283	0.210130073
ENSGALG00010026303	0.256658325	0.290346532
ENSGALG00010026305	0.627505245	0.331202186
ENSGALG00010026306	0.89904087	0.337730338
ENSGALG00010026307	0.045582495	0.125953552
ENSGALG00010026308	0.909871448	0.336858492
ENSGALG00010026310	0.67811027	0.26634437
ENSGALG00010026311	0.021428271	0.17143496
ENSGALG00010026312	0.825971836	0.440364672
ENSGALG00010026313	0.308369125	0.226033304
ENSGALG00010026314	0.49100695	0.155099052
ENSGALG00010026315	0.507355254	0.303764549
ENSGALG00010026316	0.81906179	0.310102992
ENSGALG00010026317	0.062152491	0.095068005
ENSGALG00010026318	0.280865346	0.279865599
ENSGALG00010026320	0.693289803	0.068707058
ENSGALG00010026321	0.952898083	0.341607606
ENSGALG00010026325	0.076113767	0.189429522
ENSGALG00010026328	0.948872271	0.216258104
ENSGALG00010026329	0.767278521	0.306817902
ENSGALG00010026331	0.129250193	0.225463741
ENSGALG00010026332	0.78408296	0.307055952
ENSGALG00010026333	0.364472745	0.19216895
ENSGALG00010026334	0.918464758	0.329703745
ENSGALG00010026335	0.691163381	0.143712061
ENSGALG00010026336	0.911041478	0.350384655
ENSGALG00010026337	0.47842013	0.059230431
ENSGALG00010026338	0.325516549	0.016600499
ENSGALG00010026339	0.806220419	0.296510033
ENSGALG00010026343	0.586659847	0.23651777
ENSGALG00010026344	0.915938387	0.366742801
ENSGALG00010026345	0.298140893	0.421788492
ENSGALG00010026346	0.995284848	0.323751682
ENSGALG00010026350	0.107901641	0.015396987
ENSGALG00010026352	0.972052807	0.35111119
ENSGALG00010026353	0.178260204	0.199723546
ENSGALG00010026354	0.903063055	0.355388785
ENSGALG00010026355	0.989356761	0.356610423
ENSGALG00010026356	0.463306883	0.178580614
ENSGALG00010026360	0.753313329	0.056276536
ENSGALG00010026361	0.016050289	0.159824694
ENSGALG00010026362	0.051774122	0.211366192

ENSGALG00010026363	0.216433634	0.680598293
ENSGALG00010026365	0.255910915	0.217723976
ENSGALG00010026366	0.889988379	0.383382225
ENSGALG00010026367	0.814702142	0.07557615
ENSGALG00010026368	0.106985859	0.466670428
ENSGALG00010026369	0.884808621	0.123896494
ENSGALG00010026371	0.663185263	0.381158278
ENSGALG00010026372	0.381415582	0.158761082
ENSGALG00010026375	0.775430391	0.404436746
ENSGALG00010026376	0.286231124	0.564253946
ENSGALG00010026377	0.5768335	0.127520542
ENSGALG00010026381	0.027409972	0.251906952
ENSGALG00010026382	0.60094183	0.229635484
ENSGALG00010026385	0.589688307	0.137395849
ENSGALG00010026387	0.434972361	0.277236379
ENSGALG00010026388	0.971717877	0.285968324
ENSGALG00010026391	0.337350312	0.486558046
ENSGALG00010026393	0.427632194	0.130335722
ENSGALG00010026395	0.265558089	0.028821141
ENSGALG00010026402	0.988939261	0.310131774
ENSGALG00010026403	0.982848555	0.293954926
ENSGALG00010026407	0.44375007	0.033134044
ENSGALG00010026409	0.949227515	0.208324683
ENSGALG00010026410	0.890594557	0.3497538
ENSGALG00010026415	0.140566953	0.087599282
ENSGALG00010026416	0.044307996	0.132991646
ENSGALG00010026420	0.148527247	0.406509534
ENSGALG00010026421	0.314201399	0.242348686
ENSGALG00010026422	0.814286106	0.345908826
ENSGALG00010026423	0.592247103	0.149260289
ENSGALG00010026424	0.027177818	0.116762193
ENSGALG00010026425	0.82742789	0.311704954
ENSGALG00010026426	0.018284215	0.551803092
ENSGALG00010026427	0.699138204	0.154102787
ENSGALG00010026431	0.225405672	0.042049997
ENSGALG00010026433	0.048838644	0.138324083
ENSGALG00010026434	0.951163863	0.27616691
ENSGALG00010026436	0.932951949	0.278490115
ENSGALG00010026438	0.967815292	0.354947697
ENSGALG00010026439	0.350009831	0.150816787
ENSGALG00010026442	0.264239718	0.073427965
ENSGALG00010026443	0.978327261	0.368763936
ENSGALG00010026448	0.793642624	0.019749406
ENSGALG00010026450	0.348243575	0.14397611
ENSGALG00010026453	0.306269874	0.488490028
ENSGALG00010026456	0.457253341	0.217983055
ENSGALG00010026457	0.547422609	0.076817887
ENSGALG00010026458	0.931909647	0.360760386
ENSGALG00010026459	0.744492135	0.264237256
ENSGALG00010026461	0.347438992	0.106165656
ENSGALG00010026463	0.200570274	0.091667991
ENSGALG00010026464	0.862878031	0.055534287
ENSGALG00010026465	0.123742382	0.213929497
ENSGALG00010026466	0.868486978	0.228808269
ENSGALG00010026467	0.592063798	0.293318199
ENSGALG00010026469	0.133185764	0.334922938

ENSGALG00010026470	0.904035533	0.391324171
ENSGALG00010026472	0.545885614	0.104446966
ENSGALG00010026474	0.245315472	0.062109122
ENSGALG00010026478	0.391097471	0.144336359
ENSGALG00010026480	0.946468928	0.269526519
ENSGALG00010026481	0.671416234	0.21462929
ENSGALG00010026483	0.256828349	0.119064955
ENSGALG00010026484	0.514643593	0.292730282
ENSGALG00010026485	0.986688258	0.343306577
ENSGALG00010026487	0.101438177	0.236256937
ENSGALG00010026488	0.33901797	0.388221913
ENSGALG00010026490	0.216433634	0.680598293
ENSGALG00010026492	0.865726529	0.174324169
ENSGALG00010026493	0.099781655	0.015180604
ENSGALG00010026494	0.409436305	0.178404713
ENSGALG00010026495	0.635614611	0.279958864
ENSGALG00010026498	0.291948523	0.142721471
ENSGALG00010026500	0.45367877	0.273662537
ENSGALG00010026502	0.06492309	0.207553562
ENSGALG00010026503	0.217204044	0.138280225
ENSGALG00010026506	0.242125662	0.515155453
ENSGALG00010026507	0.585786249	0.113413787
ENSGALG00010026508	0.681256891	0.428904824
ENSGALG00010026510	0.09887047	0.238061524
ENSGALG00010026514	0.500631287	0.434477371
ENSGALG00010026516	0.639273076	0.00773297
ENSGALG00010026521	0.200570274	0.091667991
ENSGALG00010026522	0.947718142	0.405001504
ENSGALG00010026523	0.36727005	0.486010284
ENSGALG00010026524	0.035357573	0.073332577
ENSGALG00010026526	0.470388556	0.132676015
ENSGALG00010026527	0.349177561	0.31565517
ENSGALG00010026529	0.01224994	0.401818935
ENSGALG00010026532	0.92935542	0.306686805
ENSGALG00010026534	0.850576667	0.119525817
ENSGALG00010026536	0.928488066	0.283115712
ENSGALG00010026537	0.629597693	0.078509287
ENSGALG00010026538	0.169102026	0.231254333
ENSGALG00010026539	0.888982786	0.327604864
ENSGALG00010026541	0.286226772	0.009585674
ENSGALG00010026542	0.469498937	0.01311824
ENSGALG00010026543	0.050497546	0.174451807
ENSGALG00010026544	0.675738065	0.235414759
ENSGALG00010026545	0.959945141	0.362203136
ENSGALG00010026546	0.394582412	0.053996367
ENSGALG00010026549	0.467703276	0.197874296
ENSGALG00010026550	0.952545283	0.293279521
ENSGALG00010026553	0.928272873	0.192952984
ENSGALG00010026555	0.942155365	0.284896139
ENSGALG00010026558	0.202386985	0.179141798
ENSGALG00010026559	0.030171494	0.375046429
ENSGALG00010026560	0.750515917	0.064586994
ENSGALG00010026562	0.94574881	0.14578227
ENSGALG00010026563	0.12466491	0.04557714
ENSGALG00010026564	0.676001734	0.257949011
ENSGALG00010026565	0.12192239	0.168061017

ENSGALG00010026566	0.958230498	0.245619621
ENSGALG00010026567	0.140197935	0.154160862
ENSGALG00010026568	0.918986326	0.242466673
ENSGALG00010026569	0.192718436	0.363784003
ENSGALG00010026572	0.204549963	0.089854095
ENSGALG00010026573	0.061723759	0.209476687
ENSGALG00010026575	0.959885694	0.319338762
ENSGALG00010026576	0.177270153	0.317017532
ENSGALG00010026577	0.208550923	0.676301294
ENSGALG00010026578	0.881691158	0.283741789
ENSGALG00010026580	0.430931775	0.218368361
ENSGALG00010026581	0.288076026	0.149878403
ENSGALG00010026583	0.687099515	0.179391419
ENSGALG00010026584	0.942862743	0.266281961
ENSGALG00010026585	0.527890335	0.170232821
ENSGALG00010026586	0.047368886	0.202239219
ENSGALG00010026587	0.264210299	0.781219858
ENSGALG00010026588	0.960067607	0.253554998
ENSGALG00010026590	0.796908771	0.380748616
ENSGALG00010026591	0.021534578	0.056414416
ENSGALG00010026592	0.561783509	0.089051534
ENSGALG00010026593	0.411711016	0.426891687
ENSGALG00010026595	0.971091577	0.306992993
ENSGALG00010026596	0.769283925	0.315879234
ENSGALG00010026597	0.352127931	0.305877975
ENSGALG00010026600	0.122983455	0.091049012
ENSGALG00010026601	0.992154522	0.320414224
ENSGALG00010026602	0.103733265	0.216099768
ENSGALG00010026603	0.548753735	0.241192955
ENSGALG00010026604	0.043326458	0.15749233
ENSGALG00010026605	0.795149731	0.077995436
ENSGALG00010026607	0.541545868	0.033903869
ENSGALG00010026608	0.571419124	0.054231907
ENSGALG00010026609	0.694887908	0.097470167
ENSGALG00010026610	0.72757015	0.248487591
ENSGALG00010026611	0.872019729	0.426574557
ENSGALG00010026612	0.91215214	0.447141624
ENSGALG00010026613	0.492451889	0.15666988
ENSGALG00010026614	0.235930578	0.458275173
ENSGALG00010026615	0.67806841	0.047912239
ENSGALG00010026616	0.264239718	0.073427965
ENSGALG00010026617	0.248593158	0.148204836
ENSGALG00010026618	0.535569679	0.199854117
ENSGALG00010026619	0.266137114	0.107784983
ENSGALG00010026620	0.189240572	0.04996112
ENSGALG00010026621	0.882078413	0.335279908
ENSGALG00010026622	0.738158832	0.503276596
ENSGALG00010026623	0.765108698	0.301005883
ENSGALG00010026625	0.927524157	0.18768831
ENSGALG00010026626	0.192273128	0.478995801
ENSGALG00010026628	0.971778443	0.376173348
ENSGALG00010026629	0.928652008	0.152859515
ENSGALG00010026630	0.962783942	0.307582
ENSGALG00010026631	0.649899874	0.231027621
ENSGALG00010026632	0.914323996	0.290931236
ENSGALG00010026633	0.93460415	0.249209433

ENSGALG00010026634	0.943579892	0.402184604
ENSGALG00010026635	0.997272574	0.336828458
ENSGALG00010026636	0.155959328	0.058280009
ENSGALG00010026637	0.285586431	0.170268186
ENSGALG00010026638	0.986909271	0.333870376
ENSGALG00010026641	0.296587781	0.382880915
ENSGALG00010026642	0.597271108	0.21030295
ENSGALG00010026643	0.974468179	0.329239349
ENSGALG00010026644	0.368476583	0.099553507
ENSGALG00010026645	0.419647781	0.161476394
ENSGALG00010026646	0.472380551	0.286466018
ENSGALG00010026647	0.800804838	0.285961707
ENSGALG00010026648	0.670201974	0.216954754
ENSGALG00010026649	0.259881341	0.078034867
ENSGALG00010026652	0.974827767	0.307125353
ENSGALG00010026653	0.617476318	0.041897655
ENSGALG00010026656	0.392408103	0.129411401
ENSGALG00010026659	0.425382975	0.030313533
ENSGALG00010026665	0.442030445	0.20194474
ENSGALG00010026666	0.712163627	0.408576821
ENSGALG00010026667	0.203991683	0.058221925
ENSGALG00010026670	0.270451009	0.090541667
ENSGALG00010026671	0.90040616	0.389074513
ENSGALG00010026672	0.177664802	0.017311945
ENSGALG00010026673	0.579216392	0.265524783
ENSGALG00010026677	0.014586239	0.094444991
ENSGALG00010026678	0.33777847	0.133444889
ENSGALG00010026681	0.946013701	0.4098681
ENSGALG00010026682	0.266137114	0.107784983
ENSGALG00010026684	0.585311105	0.191509464
ENSGALG00010026686	0.920673059	0.136323769
ENSGALG00010026688	0.501146403	0.19894571
ENSGALG00010026689	0.496532988	0.342585256
ENSGALG00010026690	0.076517201	0.139714939
ENSGALG00010026692	0.963850792	0.322250082
ENSGALG00010026693	0.228227044	0.3004198
ENSGALG00010026694	0.402394827	0.134676964
ENSGALG00010026696	0.482682836	0.107496503
ENSGALG00010026697	0.735504565	0.256788726
ENSGALG00010026698	0.19882392	0.315375103
ENSGALG00010026700	0.996983037	0.312213562
ENSGALG00010026704	0.917888081	0.330447534
ENSGALG00010026705	0.494403796	0.08868767
ENSGALG00010026706	0.993637082	0.337330885
ENSGALG00010026707	0.933112356	0.398843461
ENSGALG00010026709	0.502503961	0.205675001
ENSGALG00010026710	0.908066578	0.241335541
ENSGALG00010026711	0.651584314	0.206026503
ENSGALG00010026712	0.857384421	0.282635981
ENSGALG00010026713	0.372757682	0.112441993
ENSGALG00010026715	0.962748174	0.392950422
ENSGALG00010026717	0.965857059	0.324957439
ENSGALG00010026718	0.463412451	0.24596013
ENSGALG00010026719	0.332420727	0.288957198
ENSGALG00010026720	0.702620711	0.10414353
ENSGALG00010026721	0.330422795	0.283359363

ENSGALG00010026722	0.391896176	0.137012793
ENSGALG00010026723	0.71015424	0.235019833
ENSGALG00010026724	0.991158137	0.317095862
ENSGALG00010026725	0.319198355	0.199400058
ENSGALG00010026726	0.304868484	0.040509294
ENSGALG00010026727	0.697890852	0.245090581
ENSGALG00010026728	0.547603714	0.373398819
ENSGALG00010026729	0.98712145	0.277711494
ENSGALG00010026730	0.393547834	0.048755937
ENSGALG00010026731	0.378549276	0.412504084
ENSGALG00010026732	0.517452626	0.191158547
ENSGALG00010026733	0.960308075	0.332845031
ENSGALG00010026734	0.857419181	0.315534157
ENSGALG00010026735	0.620487687	0.295395679
ENSGALG00010026736	0.674207929	0.1899693
ENSGALG00010026740	0.929630272	0.243598472
ENSGALG00010026741	0.730224045	0.22587958
ENSGALG00010026743	0.956953012	0.220178194
ENSGALG00010026744	0.702311219	0.250019441
ENSGALG00010026745	0.962319496	0.313160079
ENSGALG00010026746	0.294688833	0.109855282
ENSGALG00010026749	0.114880295	0.165755657
ENSGALG00010026750	0.025967878	0.216501572
ENSGALG00010026752	0.487001843	0.27960257
ENSGALG00010026753	0.058070471	0.384313191
ENSGALG00010026754	0.579240978	0.261945405
ENSGALG00010026755	0.782950913	0.150102308
ENSGALG00010026757	0.637177065	0.315588765
ENSGALG00010026758	0.755865703	0.42644049
ENSGALG00010026759	0.073522075	0.142530222
ENSGALG00010026760	0.898046119	0.46496531
ENSGALG00010026767	0.581109127	0.185720949
ENSGALG00010026768	0.95167073	0.199620437
ENSGALG00010026769	0.394327047	0.133628127
ENSGALG00010026770	0.657820003	0.247292993
ENSGALG00010026771	0.422421937	0.183824808
ENSGALG00010026772	0.917550034	0.108262712
ENSGALG00010026773	0.969613597	0.298673212
ENSGALG00010026774	0.872362267	0.243876731
ENSGALG00010026775	0.305263952	0.153708414
ENSGALG00010026776	0.298469588	0.087870078
ENSGALG00010026777	0.285272007	0.109169955
ENSGALG00010026778	0.749036536	0.236719382
ENSGALG00010026779	0.383880038	0.331887406
ENSGALG00010026780	0.765818954	0.660851411
ENSGALG00010026781	0.556926897	0.100490541
ENSGALG00010026782	0.27184447	0.115107639
ENSGALG00010026783	0.242655753	0.006005106
ENSGALG00010026785	0.930191795	0.253345778
ENSGALG00010026786	0.893555004	0.453728514
ENSGALG00010026787	0.186557583	0.175131276
ENSGALG00010026788	0.415243474	0.210776538
ENSGALG00010026789	0.419622933	0.447331032
ENSGALG00010026790	0.176238473	0.295157422
ENSGALG00010026792	0.38480368	0.180523942
ENSGALG00010026793	0.910737028	0.140987221

ENSGALG00010026796	0.570418782	0.393876404
ENSGALG00010026798	0.888393869	0.067772081
ENSGALG00010026800	0.931355365	0.26811417
ENSGALG00010026801	0.580948032	0.141395323
ENSGALG00010026803	0.747324082	0.166555117
ENSGALG00010026804	0.167743938	0.538865452
ENSGALG00010026805	0.098858836	0.16668442
ENSGALG00010026806	0.704014357	0.023337755
ENSGALG00010026807	0.695065596	0.330268959
ENSGALG00010026808	0.671388698	0.20316357
ENSGALG00010026809	0.474942477	0.017340426
ENSGALG00010026810	0.799412418	0.303091461
ENSGALG00010026811	0.947752068	0.420063539
ENSGALG00010026812	0.508128282	0.020505038
ENSGALG00010026813	0.254086111	0.149558259
ENSGALG00010026814	0.808517684	0.161099434
ENSGALG00010026816	0.400778541	0.133797302
ENSGALG00010026817	0.383929351	0.106068647
ENSGALG00010026818	0.817580367	0.175877736
ENSGALG00010026819	0.40785333	0.157127562
ENSGALG00010026820	0.635640424	0.027981339
ENSGALG00010026821	0.695019486	0.230817849
ENSGALG00010026822	0.95514621	0.388443568
ENSGALG00010026823	0.88087553	0.280976963
ENSGALG00010026824	0.853486561	0.406687214
ENSGALG00010026825	0.040499019	0.21226841
ENSGALG00010026826	0.942329301	0.217460869
ENSGALG00010026827	0.982377477	0.324198528
ENSGALG00010026828	0.209971262	0.188326374
ENSGALG00010026829	0.825220675	0.187611998
ENSGALG00010026830	0.919713188	0.3125615
ENSGALG00010026831	0.94070878	0.227686856
ENSGALG00010026832	0.558304144	0.566446487
ENSGALG00010026835	0.425802778	0.287336904
ENSGALG00010026836	0.777880451	0.168399556
ENSGALG00010026838	0.483989418	0.138186428
ENSGALG00010026839	0.769105235	0.277779366
ENSGALG00010026840	0.249357771	0.231293069
ENSGALG00010026842	0.636911888	0.188463404
ENSGALG00010026844	0.181452055	0.122872189
ENSGALG00010026846	0.923200472	0.412534692
ENSGALG00010026848	0.314676458	0.072548852
ENSGALG00010026849	0.730319263	0.420569869
ENSGALG00010026850	0.149638706	0.188230914
ENSGALG00010026852	0.867879752	0.141296308
ENSGALG00010026853	0.500698309	0.025371098
ENSGALG00010026854	0.575462008	0.33806619
ENSGALG00010026856	0.215362595	0.01063801
ENSGALG00010026857	0.220163267	0.064584858
ENSGALG00010026860	0.89619285	0.222873094
ENSGALG00010026861	0.280448278	0.10678977
ENSGALG00010026862	0.495916114	0.179082398
ENSGALG00010026863	0.442333775	0.168954952
ENSGALG00010026866	0.859295506	0.299211562
ENSGALG00010026869	0.327597133	0.344787667
ENSGALG00010026870	0.37430743	0.536491602

ENSGALG00010026871	0.50038714	0.018586524
ENSGALG00010026873	0.07822304	0.346011397
ENSGALG00010026874	0.993837533	0.335135728
ENSGALG00010026875	0.492153829	0.195633769
ENSGALG00010026876	0.266137114	0.107784983
ENSGALG00010026877	0.372044779	0.369275802
ENSGALG00010026878	0.881827469	0.143841827
ENSGALG00010026881	0.070756479	0.287651046
ENSGALG00010026883	0.914987587	0.359253498
ENSGALG00010026885	0.564250359	0.077207509
ENSGALG00010026886	0.87474508	0.262534224
ENSGALG00010026887	0.963699031	0.343447769
ENSGALG00010026889	0.856386402	0.355737201
ENSGALG00010026891	0.420272235	0.211221886
ENSGALG00010026892	0.538634907	0.009014636
ENSGALG00010026893	0.745964727	0.126261416
ENSGALG00010026894	0.770494692	0.145570217
ENSGALG00010026895	0.618687411	0.207380546
ENSGALG00010026896	0.492471405	0.107463898
ENSGALG00010026897	0.782378717	0.259880796
ENSGALG00010026898	0.110197733	0.121304828
ENSGALG00010026899	0.960038963	0.303387306
ENSGALG00010026900	0.231754367	0.068663376
ENSGALG00010026901	0.106667112	0.177206863
ENSGALG00010026902	0.547676326	0.117813054
ENSGALG00010026904	0.045894358	0.166606844
ENSGALG00010026907	0.986765179	0.328333867
ENSGALG00010026909	0.960061265	0.314181595
ENSGALG00010026911	0.141927155	0.195577158
ENSGALG00010026914	0.878095171	0.146061596
ENSGALG00010026916	0.638399274	0.135267505
ENSGALG00010026917	0.978754706	0.323002347
ENSGALG00010026918	0.011730111	0.196288363
ENSGALG00010026919	0.934031228	0.458954308
ENSGALG00010026920	0.016788076	0.035497135
ENSGALG00010026923	0.077588172	0.084507011
ENSGALG00010026924	0.440259168	0.126461093
ENSGALG00010026928	0.306552732	0.317176326
ENSGALG00010026932	0.233126711	0.359820733
ENSGALG00010026933	0.47448324	0.236886836
ENSGALG00010026934	0.546168784	0.174358438
ENSGALG00010026936	0.220465126	0.150205488
ENSGALG00010026937	0.63842469	0.104047661
ENSGALG00010026942	0.92500752	0.234607488
ENSGALG00010026943	0.634039865	0.440409252
ENSGALG00010026945	0.318283099	0.114521143
ENSGALG00010026947	0.164579882	0.384164528
ENSGALG00010026948	0.89934064	0.315978324
ENSGALG00010026949	0.807143253	0.388697337
ENSGALG00010026950	0.356272512	0.234220966
ENSGALG00010026951	0.873681543	0.464510064
ENSGALG00010026952	0.090074327	0.596986925
ENSGALG00010026954	0.903971767	0.318300186
ENSGALG00010026956	0.030637844	0.185580366
ENSGALG00010026957	0.483761819	0.14768566
ENSGALG00010026958	0.968111373	0.286301932

ENSGALG00010026959	0.887802231	0.313286808
ENSGALG00010026960	0.986507112	0.300218298
ENSGALG00010026961	0.976506555	0.218681704
ENSGALG00010026962	0.375886509	0.193891102
ENSGALG00010026964	0.847829067	0.542200512
ENSGALG00010026971	0.445792948	0.233733911
ENSGALG00010026972	0.991120663	0.33025749
ENSGALG00010026973	0.964182245	0.353980316
ENSGALG00010026977	0.955333895	0.301405207
ENSGALG00010026980	0.381354712	0.44489958
ENSGALG00010026981	0.205002845	0.010871643
ENSGALG00010026982	0.926187818	0.151782614
ENSGALG00010026983	0.11186161	0.186699168
ENSGALG00010026984	0.632162773	0.027483895
ENSGALG00010026985	0.913913734	0.115390923
ENSGALG00010026986	0.463830908	0.004747734
ENSGALG00010026987	0.490239448	0.07557755
ENSGALG00010026988	0.885329951	0.207810355
ENSGALG00010026990	0.755018508	0.36545843
ENSGALG00010026991	0.575961042	0.181484671
ENSGALG00010026992	0.271701261	0.072417625
ENSGALG00010026993	0.407858851	0.138056267
ENSGALG00010026994	0.860571461	0.269406302
ENSGALG00010026995	0.560368155	0.139608595
ENSGALG00010026996	0.20304497	0.112778149
ENSGALG00010026997	0.69017027	0.014393054
ENSGALG00010026999	0.839074856	0.146714346
ENSGALG00010027000	0.739711929	0.493977492
ENSGALG00010027001	0.896916672	0.246508449
ENSGALG00010027002	0.929583591	0.286541968
ENSGALG00010027003	0.07367667	0.123663983
ENSGALG00010027004	0.520109653	0.240348827
ENSGALG00010027005	0.765317926	0.263042138
ENSGALG00010027006	0.38632594	0.125435199
ENSGALG00010027007	0.947631085	0.36805057
ENSGALG00010027010	0.796983387	0.190918361
ENSGALG00010027012	0.978860659	0.305971281
ENSGALG00010027013	0.172459806	0.171048156
ENSGALG00010027014	0.384831073	0.205747021
ENSGALG00010027015	0.942524885	0.343034064
ENSGALG00010027017	0.190942577	0.144175928
ENSGALG00010027018	0.353350086	0.126267336
ENSGALG00010027019	0.821874386	0.414022023
ENSGALG00010027020	0.537660022	0.323504659
ENSGALG00010027021	0.189240572	0.04996112
ENSGALG00010027024	0.505229181	0.224186331
ENSGALG00010027025	0.83234329	0.326040676
ENSGALG00010027026	0.949775236	0.207930918
ENSGALG00010027027	0.954781701	0.185475339
ENSGALG00010027029	0.214951439	0.133553844
ENSGALG00010027031	0.378863545	0.143149895
ENSGALG00010027034	0.790058231	0.190050848
ENSGALG00010027035	0.863543899	0.27062822
ENSGALG00010027036	0.941498775	0.325031564
ENSGALG00010027037	0.766880301	0.322522482
ENSGALG00010027038	0.695991179	0.258960896

ENSGALG00010027039	0.842015245	0.408462616
ENSGALG00010027040	0.642563569	0.229034862
ENSGALG00010027041	0.796474909	0.20255322
ENSGALG00010027042	0.520094538	0.065343547
ENSGALG00010027043	0.915821215	0.355106753
ENSGALG00010027044	0.219476199	0.307031231
ENSGALG00010027045	0.585110498	0.179664511
ENSGALG00010027046	0.608113108	0.241513949
ENSGALG00010027048	0.311289807	0.13851383
ENSGALG00010027050	0.91830994	0.240479699
ENSGALG00010027052	0.969449669	0.272125067
ENSGALG00010027053	0.566669933	0.033554097
ENSGALG00010027058	0.18738148	0.216898636
ENSGALG00010027059	0.995718672	0.31096316
ENSGALG00010027060	0.521632079	0.266201086
ENSGALG00010027061	0.446045556	0.389506667
ENSGALG00010027062	0.591760154	0.089453841
ENSGALG00010027063	0.933465213	0.271146692
ENSGALG00010027064	0.620738036	0.153109646
ENSGALG00010027069	0.111962688	0.34922536
ENSGALG00010027073	0.28363851	0.015327754
ENSGALG00010027074	0.192824112	0.116792681
ENSGALG00010027075	0.860305714	0.26873341
ENSGALG00010027076	0.898466888	0.270158966
ENSGALG00010027077	0.748988492	0.205916231
ENSGALG00010027078	0.357038425	0.269925462
ENSGALG00010027079	0.941625007	0.217587011
ENSGALG00010027080	0.928574335	0.21834484
ENSGALG00010027081	0.672314852	0.239075206
ENSGALG00010027082	0.659288186	0.027652646
ENSGALG00010027083	0.955916041	0.433789025
ENSGALG00010027084	0.693851727	0.218742332
ENSGALG00010027085	0.955147853	0.297599011
ENSGALG00010027086	0.994136943	0.322925918
ENSGALG00010027087	0.110965132	0.270342779
ENSGALG00010027088	0.959632936	0.419848788
ENSGALG00010027090	0.482405887	0.153626122
ENSGALG00010027091	0.379263365	0.120316915
ENSGALG00010027092	0.601079505	0.032336325
ENSGALG00010027093	0.675416556	0.18884627
ENSGALG00010027095	0.899046509	0.421982604
ENSGALG00010027096	0.242332652	0.070038475
ENSGALG00010027097	0.158159734	0.162855794
ENSGALG00010027098	0.881311814	0.200533536
ENSGALG00010027099	0.767485824	0.038732579
ENSGALG00010027100	0.16925738	0.111584026
ENSGALG00010027101	0.70406978	0.099825223
ENSGALG00010027102	0.650144645	0.206408898
ENSGALG00010027103	0.20620333	0.032178396
ENSGALG00010027105	0.511632059	0.406425521
ENSGALG00010027106	0.939019675	0.410403423
ENSGALG00010027107	0.971633762	0.364614124
ENSGALG00010027108	0.977935207	0.351158521
ENSGALG00010027109	0.266655332	0.094010386
ENSGALG00010027110	0.951167912	0.496403194
ENSGALG00010027111	0.093778861	0.124676379

ENSGALG00010027112	0.938974036	0.482067955
ENSGALG00010027113	0.049346651	0.184181931
ENSGALG00010027114	0.853333364	0.116925283
ENSGALG00010027115	0.971976995	0.312533167
ENSGALG00010027116	0.92096076	0.324472921
ENSGALG00010027117	0.966639394	0.254966888
ENSGALG00010027118	0.975949689	0.275950771
ENSGALG00010027119	0.696524537	0.189723911
ENSGALG00010027120	0.238868594	0.111653783
ENSGALG00010027121	0.918853271	0.387401517
ENSGALG00010027122	0.232399766	0.00713467
ENSGALG00010027123	0.379463362	0.069734421
ENSGALG00010027124	0.969485865	0.373262163
ENSGALG00010027125	0.683259515	0.033804976
ENSGALG00010027126	0.770136698	0.176961413
ENSGALG00010027127	0.767337834	0.047482094
ENSGALG00010027128	0.909287741	0.335560986
ENSGALG00010027129	0.099427165	0.027145828
ENSGALG00010027130	0.866163438	0.534705213
ENSGALG00010027131	0.915284414	0.286590614
ENSGALG00010027132	0.597749771	0.416389884
ENSGALG00010027133	0.785091637	0.305895126
ENSGALG00010027134	0.980436533	0.269505088
ENSGALG00010027135	0.218919434	0.159605423
ENSGALG00010027136	0.96983192	0.285378716
ENSGALG00010027137	0.217786815	0.071107651
ENSGALG00010027138	0.679718007	0.218586852
ENSGALG00010027139	0.597752476	0.013705383
ENSGALG00010027140	0.222326676	0.164988737
ENSGALG00010027141	0.986543646	0.38138105
ENSGALG00010027142	0.875910799	0.279658391
ENSGALG00010027143	0.937037685	0.164725866
ENSGALG00010027144	0.725574972	0.245275022
ENSGALG00010027145	0.55782729	0.118090113
ENSGALG00010027146	0.958745004	0.309692096
ENSGALG00010027147	0.5135963	0.042814963
ENSGALG00010027148	0.467765316	0.016217425
ENSGALG00010027149	0.957232546	0.243284793
ENSGALG00010027150	0.241523922	0.027277549
ENSGALG00010027151	0.657200986	0.19579719
ENSGALG00010027152	0.989343228	0.37128444
ENSGALG00010027153	0.995429138	0.322530076
ENSGALG00010027154	0.737910578	0.006268848
ENSGALG00010027155	0.994604311	0.375367804
ENSGALG00010027156	0.468027796	0.118920477
ENSGALG00010027157	0.819278433	0.198517833
ENSGALG00010027158	0.426591332	0.111779921
ENSGALG00010027159	0.984725935	0.398104417
ENSGALG00010027160	0.990477324	0.349826682
ENSGALG00010027161	0.969897331	0.292439384
ENSGALG00010027162	0.386696843	0.08099491
ENSGALG00010027163	0.063740091	0.192177987
ENSGALG00010027164	0.783089239	0.057703981
ENSGALG00010027165	0.752696809	0.032118068
ENSGALG00010027166	0.210174723	0.206154628
ENSGALG00010027167	0.565893736	0.07375293

ENSGALG00010027168	0.125884112	0.077725038
ENSGALG00010027169	0.93219644	0.341474883
ENSGALG00010027170	0.080180209	0.05477426
ENSGALG00010027171	0.990034188	0.382681099
ENSGALG00010027172	0.933774164	0.274332049
ENSGALG00010027173	0.018805314	0.085603636
ENSGALG00010027174	0.972561411	0.281405323
ENSGALG00010027175	0.966909772	0.357410665
ENSGALG00010027176	0.971656168	0.260999415
ENSGALG00010027177	0.160877949	0.012318119
ENSGALG00010027178	0.927029104	0.454931387
ENSGALG00010027179	0.600625565	0.246696092
ENSGALG00010027183	0.955372791	0.359160803
ENSGALG00010027184	0.853455258	0.278849538
ENSGALG00010027185	0.952487987	0.231760958
ENSGALG00010027186	0.804606508	0.445874988
ENSGALG00010027187	0.517084689	0.26196957
ENSGALG00010027188	0.89736298	0.267738974
ENSGALG00010027189	0.828823667	0.139772921
ENSGALG00010027190	0.859470485	0.260792423
ENSGALG00010027191	0.995168095	0.313774924
ENSGALG00010027192	0.976663266	0.235070741
ENSGALG00010027193	0.553235977	0.16485959
ENSGALG00010027194	0.506957574	0.295483608
ENSGALG00010027195	0.917789115	0.413500794
ENSGALG00010027196	0.504046175	0.065820102
ENSGALG00010027197	0.453854594	0.229154358
ENSGALG00010027198	0.95938506	0.383157041
ENSGALG00010027199	0.876771718	0.151854604
ENSGALG00010027200	0.18859791	0.029612216
ENSGALG00010027201	0.967080918	0.320830573
ENSGALG00010027202	0.044515524	0.053162021
ENSGALG00010027203	0.83176864	0.101679152
ENSGALG00010027204	0.390968473	0.13262855
ENSGALG00010027205	0.958473412	0.407076459
ENSGALG00010027206	0.992772966	0.302278743
ENSGALG00010027207	0.57790966	0.225170959
ENSGALG00010027208	0.640245143	0.420868713
ENSGALG00010027209	0.954691367	0.208486735
ENSGALG00010027210	0.643765686	0.034479458
ENSGALG00010027211	0.931510032	0.236255027
ENSGALG00010027212	0.962591871	0.373927615
ENSGALG00010027213	0.401033272	0.15803561
ENSGALG00010027214	0.735629261	0.110036748
ENSGALG00010027215	0.260735235	0.376001902
ENSGALG00010027216	0.874392819	0.081148154
ENSGALG00010027217	0.981173843	0.337790708
ENSGALG00010027218	0.435857741	0.028303951
ENSGALG00010027219	0.943294705	0.374172465
ENSGALG00010027220	0.338652557	0.021050665
ENSGALG00010027221	0.983446463	0.290100865
ENSGALG00010027222	0.262121726	0.12896837
ENSGALG00010027223	0.669555365	0.247175673
ENSGALG00010027224	0.980620635	0.279302353
ENSGALG00010027225	0.995321057	0.375328569
ENSGALG00010027227	0.984856391	0.304381857

ENSGALG00010027228	0.098683553	0.447716887
ENSGALG00010027229	0.836031806	0.489533289
ENSGALG00010027230	0.935981024	0.342476826
ENSGALG00010027231	0.770258178	0.456778498
ENSGALG00010027232	0.186024012	0.121599966
ENSGALG00010027233	0.953569893	0.270255648
ENSGALG00010027235	0.099329655	0.064271874
ENSGALG00010027236	0.813092259	0.189958971
ENSGALG00010027237	0.253191372	0.217417586
ENSGALG00010027238	0.846040966	0.307334975
ENSGALG00010027239	0.865369002	0.050014457
ENSGALG00010027240	0.478028287	0.004726755
ENSGALG00010027241	0.274150045	0.380501304
ENSGALG00010027242	0.236765457	0.022484258
ENSGALG00010027243	0.836113298	0.312385037
ENSGALG00010027244	0.956937154	0.330047016
ENSGALG00010027245	0.923948089	0.263509326
ENSGALG00010027246	0.550070341	0.49046125
ENSGALG00010027247	0.889369103	0.331772597
ENSGALG00010027248	0.243609936	0.050093473
ENSGALG00010027249	0.354775057	0.172857848
ENSGALG00010027250	0.09740914	0.037187539
ENSGALG00010027251	0.793979148	0.398699782
ENSGALG00010027252	0.912380833	0.339247993
ENSGALG00010027253	0.716987259	0.197474224
ENSGALG00010027254	0.862279795	0.309597762
ENSGALG00010027255	0.876590038	0.278955585
ENSGALG00010027256	0.810953869	0.186359949
ENSGALG00010027257	0.270989838	0.093392929
ENSGALG00010027258	0.612662727	0.137504315
ENSGALG00010027259	0.518606955	0.352260564
ENSGALG00010027260	0.840459927	0.294454015
ENSGALG00010027261	0.933361166	0.378166335
ENSGALG00010027262	0.821699309	0.228181793
ENSGALG00010027263	0.573375005	0.026948417
ENSGALG00010027264	0.708898351	0.050003353
ENSGALG00010027265	0.993346826	0.321720231
ENSGALG00010027266	0.475612077	0.164386681
ENSGALG00010027267	0.981927832	0.385501159
ENSGALG00010027268	0.792668309	0.366598015
ENSGALG00010027269	0.395848703	0.093633004
ENSGALG00010027270	0.424780338	0.007323134
ENSGALG00010027271	0.835825401	0.388984787
ENSGALG00010027272	0.476775764	0.430090138
ENSGALG00010027273	0.687875303	0.400484852
ENSGALG00010027274	0.396697347	0.124692782
ENSGALG00010027275	0.808496541	0.065158469
ENSGALG00010027276	0.983225368	0.332715743
ENSGALG00010027277	0.964872234	0.224606692
ENSGALG00010027278	0.971294185	0.307303771
ENSGALG00010027279	0.884552123	0.137304969
ENSGALG00010027280	0.464609721	0.148847343
ENSGALG00010027281	0.973096709	0.323122416
ENSGALG00010027282	0.994765419	0.334669597
ENSGALG00010027283	0.957219448	0.31796606
ENSGALG00010027284	0.87430726	0.410425033

ENSGALG00010027285	0.170354246	0.145765641
ENSGALG00010027286	0.811207566	0.283562774
ENSGALG00010027287	0.036506424	0.333028776
ENSGALG00010027288	0.98523295	0.353029214
ENSGALG00010027289	0.939347122	0.329845251
ENSGALG00010027290	0.417114815	0.278041149
ENSGALG00010027291	0.981515952	0.349584838
ENSGALG00010027292	0.723871821	0.343506551
ENSGALG00010027293	0.104977685	0.168858492
ENSGALG00010027295	0.067445671	0.00099302
ENSGALG00010027298	0.21983606	0.195833374
ENSGALG00010027299	0.251460375	0.3281414
ENSGALG00010027301	0.86215128	0.237091508
ENSGALG00010027302	0.750820096	0.098691126
ENSGALG00010027303	0.872373582	0.188549594
ENSGALG00010027304	0.863164695	0.386126207
ENSGALG00010027305	0.782327265	0.258565578
ENSGALG00010027306	0.74124342	0.212010275
ENSGALG00010027307	0.920701656	0.288808835
ENSGALG00010027308	0.696083861	0.215373404
ENSGALG00010027309	0.379253257	0.133484065
ENSGALG00010027310	0.015770557	0.351965662
ENSGALG00010027311	0.102373688	0.088541624
ENSGALG00010027312	0.65626791	0.226291583
ENSGALG00010027313	0.592939514	0.248444414
ENSGALG00010027314	0.207566041	0.115866567
ENSGALG00010027315	0.943230702	0.307220921
ENSGALG00010027316	0.511151157	0.096564785
ENSGALG00010027317	0.888257284	0.278894041
ENSGALG00010027319	0.898661518	0.28501487
ENSGALG00010027320	0.916896945	0.327824676
ENSGALG00010027321	0.911638626	0.272529235
ENSGALG00010027322	0.062591358	0.308974745
ENSGALG00010027323	0.823677752	0.526544763
ENSGALG00010027324	0.773648363	0.184509933
ENSGALG00010027325	0.867875278	0.309461295
ENSGALG00010027326	0.916084191	0.303926997
ENSGALG00010027327	0.019822206	0.030899204
ENSGALG00010027328	0.883212414	0.33497984
ENSGALG00010027329	0.14395321	0.077863881
ENSGALG00010027330	0.912682879	0.340202735
ENSGALG00010027331	0.888870379	0.393537135
ENSGALG00010027332	0.992088797	0.319082537
ENSGALG00010027333	0.590010665	0.165968702
ENSGALG00010027334	0.410049505	0.048390329
ENSGALG00010027335	0.779499072	0.120784294
ENSGALG00010027336	0.4596063	0.112804291
ENSGALG00010027337	0.898842368	0.193336208
ENSGALG00010027338	0.688952385	0.228997225
ENSGALG00010027339	0.964848811	0.255441953
ENSGALG00010027340	0.829565677	0.099542952
ENSGALG00010027341	0.943040508	0.334050679
ENSGALG00010027342	0.90619498	0.274187297
ENSGALG00010027343	0.39902227	0.15681398
ENSGALG00010027345	0.995315898	0.334314166
ENSGALG00010027346	0.975590496	0.323795027

ENSGALG00010027348	0.864060794	0.372153484
ENSGALG00010027349	0.343869248	0.108583644
ENSGALG00010027350	0.7613431	0.252829308
ENSGALG00010027351	0.997173478	0.347834996
ENSGALG00010027353	0.850563306	0.285919686
ENSGALG00010027354	0.220818482	0.205768094
ENSGALG00010027355	0.250650061	0.181903159
ENSGALG00010027356	0.873283012	0.43357397
ENSGALG00010027358	0.25507883	0.018812448
ENSGALG00010027359	0.973024932	0.338748987
ENSGALG00010027360	0.982100316	0.363955718
ENSGALG00010027361	0.951242448	0.268108389
ENSGALG00010027362	0.906732874	0.343796118
ENSGALG00010027363	0.983056568	0.324529791
ENSGALG00010027364	0.888997368	0.308317406
ENSGALG00010027365	0.941380547	0.342563957
ENSGALG00010027366	0.849417654	0.406028124
ENSGALG00010027367	0.968857091	0.30562357
ENSGALG00010027368	0.912960378	0.186117923
ENSGALG00010027369	0.482375381	0.205016808
ENSGALG00010027370	0.015605199	0.287868235
ENSGALG00010027371	0.828348563	0.258662681
ENSGALG00010027372	0.91397857	0.331438123
ENSGALG00010027374	0.870316608	0.323321363
ENSGALG00010027375	0.902566682	0.228504174
ENSGALG00010027376	0.903523693	0.192692895
ENSGALG00010027377	0.343963812	0.193657218
ENSGALG00010027378	0.949465306	0.37237196
ENSGALG00010027379	0.634621335	0.258867571
ENSGALG00010027380	0.650547804	0.263040418
ENSGALG00010027381	0.977758819	0.310978673
ENSGALG00010027382	0.606661124	0.319458182
ENSGALG00010027383	0.955807697	0.298315346
ENSGALG00010027384	0.979101431	0.29956387
ENSGALG00010027385	0.214586984	0.706975737
ENSGALG00010027386	0.868335488	0.378094427
ENSGALG00010027387	0.804346467	0.493400444
ENSGALG00010027388	0.905654474	0.354417482
ENSGALG00010027389	0.81033988	0.270978602
ENSGALG00010027390	0.136624304	0.162266219
ENSGALG00010027391	0.887360729	0.213567453
ENSGALG00010027392	0.874672756	0.184560305
ENSGALG00010027393	0.456444857	0.005070985
ENSGALG00010027394	0.975195161	0.271782478
ENSGALG00010027395	0.965386756	0.397067107
ENSGALG00010027396	0.932462324	0.302171426
ENSGALG00010027397	0.209367105	0.088230139
ENSGALG00010027398	0.802082618	0.251403212
ENSGALG00010027399	0.918176524	0.274527629
ENSGALG00010027400	0.90998707	0.315840671
ENSGALG00010027401	0.571502716	0.087120228
ENSGALG00010027402	0.691180816	0.420734726
ENSGALG00010027403	0.943490822	0.226555318
ENSGALG00010027404	0.348392707	0.223347139
ENSGALG00010027405	0.589633768	0.026422543
ENSGALG00010027406	0.969845909	0.323687436

ENSGALG00010027407	0.147441892	0.081240921
ENSGALG00010027408	0.840288828	0.120542047
ENSGALG00010027409	0.997117853	0.336142618
ENSGALG00010027410	0.587397052	0.114686982
ENSGALG00010027411	0.203374105	0.161442747
ENSGALG00010027412	0.175114286	0.030075348
ENSGALG00010027413	0.106557069	0.147443835
ENSGALG00010027414	0.41673091	0.179938717
ENSGALG00010027415	0.48236159	0.200526498
ENSGALG00010027416	0.264239718	0.073427965
ENSGALG00010027417	0.004290657	0.08735817
ENSGALG00010027418	0.981783842	0.321320893
ENSGALG00010027419	0.992626244	0.295756919
ENSGALG00010027421	0.59754162	0.001825941
ENSGALG00010027423	0.880822445	0.319467498
ENSGALG00010027425	0.953901924	0.275380373
ENSGALG00010027427	0.995162511	0.310020224
ENSGALG00010027429	0.385953062	0.133261917
ENSGALG00010027430	0.946055039	0.364061162
ENSGALG00010027437	0.285272007	0.109169955
ENSGALG00010027438	0.604303313	0.157418565
ENSGALG00010027439	0.141921482	0.120746505
ENSGALG00010027440	0.950705815	0.246189224
ENSGALG00010027442	0.665801698	0.200317472
ENSGALG00010027443	0.906687656	0.354471636
ENSGALG00010027444	0.946908872	0.277802304
ENSGALG00010027445	0.817884647	0.206727442
ENSGALG00010027446	0.367937607	0.139259061
ENSGALG00010027447	0.947808231	0.314480071
ENSGALG00010027448	0.949486118	0.276479672
ENSGALG00010027450	0.97661897	0.275992397
ENSGALG00010027451	0.942114484	0.421759436
ENSGALG00010027452	0.498363078	0.178723607
ENSGALG00010027455	0.952002095	0.30288667
ENSGALG00010027456	0.804537764	0.386097113
ENSGALG00010027457	0.953273984	0.321733127
ENSGALG00010027458	0.836112204	0.235340956
ENSGALG00010027460	0.83954526	0.310217337
ENSGALG00010027465	0.285272007	0.109169955
ENSGALG00010027467	0.897849157	0.399203483
ENSGALG00010027468	0.859579477	0.103115551
ENSGALG00010027471	0.957064972	0.301321612
ENSGALG00010027473	0.3043248	0.138242451
ENSGALG00010027475	0.849846442	0.247295142
ENSGALG00010027479	0.577970235	0.18535283
ENSGALG00010027482	0.985691654	0.266665815
ENSGALG00010027484	0.84596384	0.147155221
ENSGALG00010027485	0.427571157	0.212401488
ENSGALG00010027487	0.940526174	0.325086191
ENSGALG00010027488	0.03966364	0.431234357
ENSGALG00010027490	0.973545836	0.34721996
ENSGALG00010027492	0.820562627	0.241314115
ENSGALG00010027494	0.501872963	0.17834951
ENSGALG00010027496	0.978458056	0.294404943
ENSGALG00010027497	0.961642729	0.283738256
ENSGALG00010027499	0.956127828	0.360336052

ENSGALG00010027501	0.086039983	0.38613704
ENSGALG00010027502	0.174789153	0.245336692
ENSGALG00010027508	0.735472024	0.064804056
ENSGALG00010027509	0.32650125	0.087795048
ENSGALG00010027510	0.104429505	0.115460514
ENSGALG00010027511	0.287939373	0.14004179
ENSGALG00010027513	0.957882194	0.347017216
ENSGALG00010027514	0.427961052	0.017589223
ENSGALG00010027516	0.53347991	0.038325463
ENSGALG00010027519	0.37189097	0.203344338
ENSGALG00010027520	0.212962903	0.146646555
ENSGALG00010027521	0.429649667	0.197737581
ENSGALG00010027522	0.508969132	0.023987625
ENSGALG00010027523	0.000700814	0.093369101
ENSGALG00010027524	0.930935039	0.330126786
ENSGALG00010027525	0.979094715	0.307558943
ENSGALG00010027526	0.117359235	0.246998166
ENSGALG00010027527	0.032047763	0.300258532
ENSGALG00010027528	0.996545913	0.343909087
ENSGALG00010027529	0.881370685	0.424940845
ENSGALG00010027530	0.73035168	0.17770394
ENSGALG00010027531	0.926657557	0.329123572
ENSGALG00010027532	0.76889775	0.39549808
ENSGALG00010027533	0.28278745	0.194921985
ENSGALG00010027534	0.01062693	0.23176015
ENSGALG00010027535	0.922288532	0.327172978
ENSGALG00010027536	0.954328762	0.357080004
ENSGALG00010027537	0.795687678	0.394229378
ENSGALG00010027538	0.157585179	0.325083935
ENSGALG00010027539	0.978753555	0.305773775
ENSGALG00010027540	0.965130019	0.214910096
ENSGALG00010027541	0.327157917	0.295427091
ENSGALG00010027543	0.580507235	0.302293869
ENSGALG00010027544	0.381352663	0.078374659
ENSGALG00010027545	0.848397778	0.042766979
ENSGALG00010027547	0.962802053	0.341663045
ENSGALG00010027548	0.72258646	0.182989504
ENSGALG00010027550	0.982128897	0.346425606
ENSGALG00010027551	0.266137114	0.107784983
ENSGALG00010027552	0.57901627	0.161917044
ENSGALG00010027553	0.4385742	0.179389012
ENSGALG00010027554	0.744482627	0.357791779
ENSGALG00010027555	0.205614849	0.197314934
ENSGALG00010027557	0.986664849	0.276385177
ENSGALG00010027558	0.574744657	0.198171048
ENSGALG00010027559	0.586069436	0.015848861
ENSGALG00010027560	0.306259766	0.000467764
ENSGALG00010027561	0.548073247	0.258119604
ENSGALG00010027562	0.181593194	0.455162489
ENSGALG00010027563	0.882121959	0.115883549
ENSGALG00010027564	0.203991683	0.058221925
ENSGALG00010027565	0.24866164	0.087370686
ENSGALG00010027566	0.119246839	0.199824633
ENSGALG00010027567	0.880941291	0.285871107
ENSGALG00010027568	0.930762775	0.232272757
ENSGALG00010027569	0.937486166	0.340208802

ENSGALG00010027570	0.846571992	0.323424289
ENSGALG00010027571	0.499632416	0.32599196
ENSGALG00010027572	0.265558089	0.028821141
ENSGALG00010027573	0.439899265	0.153465875
ENSGALG00010027574	0.507566857	0.224266854
ENSGALG00010027575	0.902141357	0.345372919
ENSGALG00010027576	0.792798608	0.26796391
ENSGALG00010027577	0.929488333	0.406413568
ENSGALG00010027578	0.749755696	0.419184992
ENSGALG00010027579	0.942818241	0.236776597
ENSGALG00010027582	0.969592994	0.357801855
ENSGALG00010027583	0.099997557	0.080800681
ENSGALG00010027584	0.358566787	0.022041791
ENSGALG00010027585	0.934429272	0.441306551
ENSGALG00010027586	0.70794526	0.096293218
ENSGALG00010027587	0.455302352	0.144545543
ENSGALG00010027589	0.931818767	0.297792726
ENSGALG00010027590	0.755947158	0.185129658
ENSGALG00010027591	0.043070681	0.341217638
ENSGALG00010027592	0.924119693	0.314145152
ENSGALG00010027593	0.865892813	0.282826829
ENSGALG00010027594	0.415098449	0.112543059
ENSGALG00010027595	0.106346801	0.099646415
ENSGALG00010027596	0.622314352	0.19748367
ENSGALG00010027597	0.869141766	0.278977912
ENSGALG00010027598	0.833157916	0.095375349
ENSGALG00010027599	0.56828583	0.155197336
ENSGALG00010027600	0.940237023	0.467909621
ENSGALG00010027601	0.394065288	0.189158137
ENSGALG00010027603	0.267108756	0.00253245
ENSGALG00010027604	0.371009605	0.131442379
ENSGALG00010027605	0.809257815	0.444778589
ENSGALG00010027606	0.100997164	0.100050188
ENSGALG00010027607	0.701855984	0.07791454
ENSGALG00010027608	0.669373366	0.042440855
ENSGALG00010027609	0.872869143	0.341564054
ENSGALG00010027610	0.084026167	0.142254473
ENSGALG00010027611	0.817127185	0.363399865
ENSGALG00010027612	0.664008527	0.406713482
ENSGALG00010027613	0.974004613	0.350385126
ENSGALG00010027614	0.59677279	0.224999051
ENSGALG00010027615	0.947223802	0.421988643
ENSGALG00010027616	0.915695878	0.347046678
ENSGALG00010027617	0.896809568	0.243289424
ENSGALG00010027618	0.934286265	0.455573513
ENSGALG00010027619	0.857258523	0.220595834
ENSGALG00010027620	0.490375351	0.145312611
ENSGALG00010027621	0.648538837	0.098540465
ENSGALG00010027623	0.991693196	0.327940594
ENSGALG00010027624	0.706700907	0.299967779
ENSGALG00010027625	0.356803561	0.133583842
ENSGALG00010027626	0.918391599	0.476930125
ENSGALG00010027627	0.982320861	0.297036448
ENSGALG00010027628	0.92554469	0.331105476
ENSGALG00010027630	0.029548167	0.351637376
ENSGALG00010027632	0.143761322	0.197372915

ENSGALG00010027633	0.235630588	0.132393411
ENSGALG00010027634	0.900892085	0.400738983
ENSGALG00010027635	0.949008939	0.29225683
ENSGALG00010027636	0.843803778	0.112044564
ENSGALG00010027638	0.933561406	0.357830582
ENSGALG00010027639	0.393172979	0.190906177
ENSGALG00010027641	0.913089109	0.328031027
ENSGALG00010027642	0.194229538	0.282535142
ENSGALG00010027644	0.874664886	0.166346748
ENSGALG00010027645	0.845572395	0.15855498
ENSGALG00010027648	0.492996679	0.296109303
ENSGALG00010027649	0.978832386	0.327653071
ENSGALG00010027650	0.991833929	0.348325027
ENSGALG00010027653	0.949698021	0.32755404
ENSGALG00010027654	0.923593057	0.264359974
ENSGALG00010027656	0.265558089	0.028821141
ENSGALG00010027657	0.040432554	0.053381713
ENSGALG00010027658	0.143167506	0.121789685
ENSGALG00010027660	0.951908224	0.25622129
ENSGALG00010027662	0.101113316	0.192317771
ENSGALG00010027663	0.670479033	0.344550517
ENSGALG00010027664	0.866574173	0.225371968
ENSGALG00010027665	0.898552751	0.195787722
ENSGALG00010027666	0.509209055	0.174704086
ENSGALG00010027667	0.871479525	0.368553922
ENSGALG00010027668	0.912254063	0.233748797
ENSGALG00010027669	0.397377422	0.153175915
ENSGALG00010027670	0.470730783	0.24369868
ENSGALG00010027671	0.600870891	0.037970672
ENSGALG00010027672	0.308445475	0.061167449
ENSGALG00010027673	0.570625664	0.062485931
ENSGALG00010027674	0.303247085	0.41322054
ENSGALG00010027675	0.805867616	0.156208411
ENSGALG00010027676	0.59822768	0.186897106
ENSGALG00010027677	0.04158158	0.286470121
ENSGALG00010027678	0.040848327	0.018271937
ENSGALG00010027682	0.573281563	0.217712352
ENSGALG00010027684	0.566202921	0.145654308
ENSGALG00010027685	0.953511868	0.366886022
ENSGALG00010027692	0.211882199	0.329278391
ENSGALG00010027693	0.974261067	0.373256087
ENSGALG00010027695	0.825965898	0.449090128
ENSGALG00010027697	0.943282854	0.297944966
ENSGALG00010027698	0.888440219	0.466161467
ENSGALG00010027699	0.448997106	0.332276674
ENSGALG00010027700	0.716204879	0.312748014
ENSGALG00010027701	0.360419722	0.108643065
ENSGALG00010027702	0.834663992	0.096370023
ENSGALG00010027703	0.938336459	0.306462876
ENSGALG00010027704	0.936919388	0.313258086
ENSGALG00010027705	0.881625682	0.347760803
ENSGALG00010027706	0.977078147	0.38404087
ENSGALG00010027707	0.81604685	0.19600113
ENSGALG00010027708	0.968194825	0.340685466
ENSGALG00010027709	0.967473365	0.367306742
ENSGALG00010027710	0.982399958	0.326319903

ENSGALG00010027711	0.990430712	0.31651893
ENSGALG00010027712	0.768774303	0.323704118
ENSGALG00010027713	0.95691432	0.296983232
ENSGALG00010027714	0.997016399	0.345954726
ENSGALG00010027715	0.958357955	0.249423449
ENSGALG00010027716	0.909201355	0.250037144
ENSGALG00010027717	0.961513132	0.276929668
ENSGALG00010027718	0.964800343	0.400411275
ENSGALG00010027719	0.308096699	0.206514912
ENSGALG00010027720	0.612185896	0.058162034
ENSGALG00010027721	0.836153587	0.100779761
ENSGALG00010027722	0.954650195	0.250133679
ENSGALG00010027723	0.573096012	0.621055914
ENSGALG00010027724	0.287251733	0.188756347
ENSGALG00010027725	0.870356835	0.249465394
ENSGALG00010027726	0.337546929	0.226422631
ENSGALG00010027727	0.863421977	0.312923692
ENSGALG00010027729	0.479533975	0.239974749
ENSGALG00010027730	0.367279456	0.018476538
ENSGALG00010027731	0.774928351	0.499782603
ENSGALG00010027732	0.892086871	0.322388543
ENSGALG00010027733	0.857077213	0.291603288
ENSGALG00010027734	0.943879036	0.226026221
ENSGALG00010027735	0.327648449	0.122209282
ENSGALG00010027737	0.543058831	0.186462903
ENSGALG00010027738	0.563142106	0.188517936
ENSGALG00010027739	0.919858173	0.24738343
ENSGALG00010027740	0.021901189	0.214789868
ENSGALG00010027741	0.892983021	0.214731766
ENSGALG00010027742	0.041111327	0.416080968
ENSGALG00010027743	0.983069759	0.295799566
ENSGALG00010027745	0.983172807	0.313440409
ENSGALG00010027746	0.937048932	0.258602381
ENSGALG00010027747	0.416630567	0.118574617
ENSGALG00010027749	0.199173202	0.242170123
ENSGALG00010027750	0.039562992	0.352215587
ENSGALG00010027751	0.960132384	0.232840991
ENSGALG00010027752	0.831370312	0.28434921
ENSGALG00010027753	0.552408025	0.334425252
ENSGALG00010027755	0.28880012	0.418789177
ENSGALG00010027757	0.399426914	0.178230835
ENSGALG00010027760	0.285272007	0.109169955
ENSGALG00010027761	0.405430886	0.133933538
ENSGALG00010027762	0.410509628	0.075378376
ENSGALG00010027763	0.188457015	0.255709569
ENSGALG00010027764	0.084003755	0.097042014
ENSGALG00010027766	0.294193127	0.028634191
ENSGALG00010027767	0.370747868	0.079551735
ENSGALG00010027768	0.869041395	0.091010289
ENSGALG00010027771	0.208685486	0.28183655
ENSGALG00010027773	0.347170959	0.008041418
ENSGALG00010027774	0.550540597	0.19826893
ENSGALG00010027775	0.939337612	0.282733118
ENSGALG00010027776	0.963142042	0.291489491
ENSGALG00010027777	0.058556974	0.175380619
ENSGALG00010027778	0.680910238	0.012039566

ENSGALG00010027781	0.943591338	0.214037865
ENSGALG00010027782	0.380172439	0.107848187
ENSGALG00010027784	0.612941046	0.475862792
ENSGALG00010027785	0.211318708	0.247254828
ENSGALG00010027786	0.096552008	0.170527818
ENSGALG00010027787	0.640486897	0.236476377
ENSGALG00010027788	0.285272007	0.109169955
ENSGALG00010027789	0.967010303	0.305937135
ENSGALG00010027790	0.919789344	0.379820664
ENSGALG00010027791	0.140881287	0.187713542
ENSGALG00010027792	0.092853389	0.297573914
ENSGALG00010027797	0.34812827	0.225215696
ENSGALG00010027798	0.586758396	0.298237524
ENSGALG00010027799	0.695845721	0.232330082
ENSGALG00010027801	0.777891615	0.515580832
ENSGALG00010027803	0.958414234	0.305365601
ENSGALG00010027804	0.012433676	0.134633327
ENSGALG00010027806	0.541923285	0.131629089
ENSGALG00010027807	0.210073371	0.148173087
ENSGALG00010027808	0.38480368	0.180523942
ENSGALG00010027809	0.589106733	0.286402863
ENSGALG00010027810	0.130338192	0.245298534
ENSGALG00010027811	0.978218585	0.292182737
ENSGALG00010027812	0.299847948	0.140446925
ENSGALG00010027813	0.237809095	0.300688278
ENSGALG00010027814	0.91820048	0.373071754
ENSGALG00010027815	0.292709036	0.327963953
ENSGALG00010027816	0.771181319	0.217385567
ENSGALG00010027818	0.200570274	0.091667991
ENSGALG00010027819	0.276757289	0.268822049
ENSGALG00010027820	0.383562316	0.07752016
ENSGALG00010027821	0.979676956	0.369014162
ENSGALG00010027822	0.960353243	0.25107578
ENSGALG00010027824	0.884681756	0.329133544
ENSGALG00010027825	0.768835864	0.424094847
ENSGALG00010027826	0.924742383	0.270563297
ENSGALG00010027827	0.71805029	0.203558695
ENSGALG00010027829	0.967752845	0.384160912
ENSGALG00010027830	0.890213231	0.313610921
ENSGALG00010027831	0.961637058	0.38376708
ENSGALG00010027833	0.011292696	0.05037781
ENSGALG00010027834	0.950255114	0.326024641
ENSGALG00010027835	0.354612028	0.410667109
ENSGALG00010027836	0.749740794	0.103909624
ENSGALG00010027837	0.222393692	0.167305868
ENSGALG00010027838	0.9218141	0.20275979
ENSGALG00010027839	0.635119414	0.187980408
ENSGALG00010027841	0.888396384	0.374062279
ENSGALG00010027842	0.507989482	0.360747869
ENSGALG00010027844	0.156244913	0.107042072
ENSGALG00010027845	0.601887444	0.407056354
ENSGALG00010027847	0.824036526	0.525810791
ENSGALG00010027851	0.208550923	0.676301294
ENSGALG00010027854	0.34156832	0.078045473
ENSGALG00010027855	0.249075056	0.249313821
ENSGALG00010027856	0.486419223	0.143168371

ENSGALG00010027857	0.34608874	0.077180519
ENSGALG00010027860	0.766725669	0.497002004
ENSGALG00010027861	0.05551787	0.163912731
ENSGALG00010027862	0.873302101	0.224925461
ENSGALG00010027863	0.977133349	0.299538255
ENSGALG00010027864	0.826418521	0.263131805
ENSGALG00010027865	0.791946254	0.160253292
ENSGALG00010027866	0.477938861	0.471203489
ENSGALG00010027867	0.860922693	0.407692714
ENSGALG00010027868	0.216433634	0.680598293
ENSGALG00010027869	0.708664859	0.231550135
ENSGALG00010027870	0.988299734	0.339861339
ENSGALG00010027872	0.8011377	0.233885809
ENSGALG00010027873	0.17838717	0.164539164
ENSGALG00010027874	0.61643466	0.123922025
ENSGALG00010027875	0.900332636	0.411154019
ENSGALG00010027876	0.390785544	0.021697962
ENSGALG00010027877	0.651715614	0.242954885
ENSGALG00010027878	0.12305961	0.187427303
ENSGALG00010027879	0.969378774	0.310198663
ENSGALG00010027880	0.969262576	0.301184856
ENSGALG00010027881	0.768951637	0.291536768
ENSGALG00010027882	0.962855204	0.339095759
ENSGALG00010027883	0.783474408	0.355975677
ENSGALG00010027884	0.806169129	0.187023244
ENSGALG00010027885	0.954006102	0.32778919
ENSGALG00010027886	0.804889489	0.413428069
ENSGALG00010027887	0.490561027	0.208223985
ENSGALG00010027888	0.86566571	0.373693331
ENSGALG00010027889	0.809686329	0.3567007
ENSGALG00010027890	0.793480855	0.394502713
ENSGALG00010027891	0.639630538	0.016481666
ENSGALG00010027892	0.018276013	0.250391033
ENSGALG00010027893	0.828675731	0.193306945
ENSGALG00010027894	0.78544965	0.222931805
ENSGALG00010027895	0.904262128	0.407029509
ENSGALG00010027896	0.97215516	0.201172636
ENSGALG00010027897	0.78624664	0.275493523
ENSGALG00010027898	0.854437628	0.549761491
ENSGALG00010027899	0.832592941	0.329226147
ENSGALG00010027900	0.985862893	0.274140174
ENSGALG00010027901	0.828057865	0.124369428
ENSGALG00010027902	0.837245212	0.226966386
ENSGALG00010027903	0.903776698	0.341075048
ENSGALG00010027904	0.722813866	0.378436968
ENSGALG00010027905	0.250329824	0.018573168
ENSGALG00010027906	0.952406078	0.357282364
ENSGALG00010027907	0.992026518	0.317799095
ENSGALG00010027908	0.968449283	0.339400791
ENSGALG00010027909	0.691976352	0.094312341
ENSGALG00010027910	0.006226029	0.262741413
ENSGALG00010027911	0.915121699	0.258310589
ENSGALG00010027912	0.4936239	0.398267853
ENSGALG00010027913	0.533493931	0.099499711
ENSGALG00010027914	0.974116727	0.29360225
ENSGALG00010027915	0.204556209	0.071256561

ENSGALG00010027916	0.86143543	0.234874196
ENSGALG00010027917	0.917773924	0.261116626
ENSGALG00010027918	0.977567631	0.34856789
ENSGALG00010027919	0.876753513	0.281049537
ENSGALG00010027920	0.751680243	0.177820533
ENSGALG00010027921	0.7675496	0.241180735
ENSGALG00010027922	0.121346884	0.231968526
ENSGALG00010027923	0.94266326	0.197522003
ENSGALG00010027924	0.920184545	0.392673156
ENSGALG00010027925	0.746553643	0.159637869
ENSGALG00010027926	0.418582331	0.182800994
ENSGALG00010027927	0.991842681	0.338443282
ENSGALG00010027928	0.427723161	0.152431441
ENSGALG00010027929	0.422184508	0.168783767
ENSGALG00010027930	0.985590979	0.286373296
ENSGALG00010027931	0.991659978	0.345268049
ENSGALG00010027932	0.892738624	0.20293662
ENSGALG00010027933	0.637942814	0.504347223
ENSGALG00010027934	0.908531127	0.345598751
ENSGALG00010027935	0.530090258	0.217974375
ENSGALG00010027936	0.809663338	0.319812283
ENSGALG00010027937	0.494424598	0.11827745
ENSGALG00010027938	0.851013265	0.40054003
ENSGALG00010027939	0.826565432	0.185642909
ENSGALG00010027940	0.861766563	0.069633464
ENSGALG00010027941	0.074160509	0.204569886
ENSGALG00010027942	0.500070257	0.211469438
ENSGALG00010027943	0.988871207	0.340736349
ENSGALG00010027944	0.722522172	0.175527126
ENSGALG00010027946	0.179126645	0.381609164
ENSGALG00010027947	0.861326966	0.397382879
ENSGALG00010027948	0.870540291	0.271511389
ENSGALG00010027949	0.54335597	0.155217282
ENSGALG00010027950	0.958624489	0.29034508
ENSGALG00010027951	0.799186891	0.306930076
ENSGALG00010027952	0.907356032	0.146018758
ENSGALG00010027953	0.986792862	0.246607177
ENSGALG00010027954	0.938081162	0.411184831
ENSGALG00010027955	0.848476	0.360028122
ENSGALG00010027956	0.986117527	0.282315065
ENSGALG00010027957	0.044602121	0.233381933
ENSGALG00010027958	0.853578103	0.306715845
ENSGALG00010027959	0.059652645	0.261265823
ENSGALG00010027960	0.877321349	0.34615161
ENSGALG00010027961	0.605510821	0.177294406
ENSGALG00010027962	0.926636825	0.350522491
ENSGALG00010027964	0.927132315	0.317447616
ENSGALG00010027965	0.948538641	0.422359318
ENSGALG00010027966	0.60014083	0.096182301
ENSGALG00010027968	0.927350372	0.484463056
ENSGALG00010027969	0.911677962	0.292776168
ENSGALG00010027970	0.838068253	0.141894378
ENSGALG00010027971	0.681050715	0.515519332
ENSGALG00010027972	0.151264133	0.0303821
ENSGALG00010027973	0.659041381	0.452683621
ENSGALG00010027974	0.697805349	0.363782812

ENSGALG00010027975	0.974761718	0.258408486
ENSGALG00010027976	0.630777234	0.382104906
ENSGALG00010027977	0.981326414	0.39872327
ENSGALG00010027979	0.776593307	0.2923142
ENSGALG00010027980	0.960100574	0.319635804
ENSGALG00010027981	0.856711768	0.091168862
ENSGALG00010027982	0.759239156	0.376444782
ENSGALG00010027983	0.873269456	0.326985134
ENSGALG00010027984	0.909543409	0.352862712
ENSGALG00010027985	0.968854513	0.335392014
ENSGALG00010027986	0.958330493	0.309861378
ENSGALG00010027987	0.416182109	0.168190418
ENSGALG00010027988	0.345776417	0.207947535
ENSGALG00010027989	0.53259045	0.25240673
ENSGALG00010027990	0.867875843	0.224178074
ENSGALG00010027991	0.408735413	0.237258012
ENSGALG00010027993	0.782214684	0.357875878
ENSGALG00010027994	0.006297287	0.466792324
ENSGALG00010027995	0.387857269	0.098722115
ENSGALG00010027996	0.891356629	0.260885249
ENSGALG00010027997	0.361444391	0.09663825
ENSGALG00010027998	0.991190369	0.350223263
ENSGALG00010027999	0.186336073	0.186221145
ENSGALG00010028000	0.982738541	0.314925882
ENSGALG00010028001	0.949396272	0.342820369
ENSGALG00010028002	0.448248202	0.13158303
ENSGALG00010028004	0.850342042	0.373072828
ENSGALG00010028005	0.975296179	0.309237314
ENSGALG00010028006	0.543931112	0.254682888
ENSGALG00010028007	0.911404866	0.233287762
ENSGALG00010028008	0.975060978	0.344839458
ENSGALG00010028009	0.95213814	0.316961303
ENSGALG00010028010	0.702302852	0.35670526
ENSGALG00010028011	0.376956865	0.079022251
ENSGALG00010028012	0.729742595	0.374264265
ENSGALG00010028013	0.982677003	0.309318955
ENSGALG00010028014	0.16840497	0.108071966
ENSGALG00010028015	0.97384841	0.272320284
ENSGALG00010028016	0.861587925	0.291385633
ENSGALG00010028017	0.649055997	0.403801177
ENSGALG00010028019	0.961342552	0.307323205
ENSGALG00010028020	0.588580492	0.322746844
ENSGALG00010028021	0.980194721	0.297832345
ENSGALG00010028022	0.659161713	0.083747521
ENSGALG00010028023	0.304058409	0.967808645
ENSGALG00010028024	0.879401055	0.281753495
ENSGALG00010028025	0.991222954	0.311363977
ENSGALG00010028026	0.108320634	0.034270087
ENSGALG00010028027	0.172709069	0.130692177
ENSGALG00010028028	0.913993945	0.406786299
ENSGALG00010028029	0.477386554	0.240567571
ENSGALG00010028030	0.158507398	0.282957871
ENSGALG00010028031	0.322587846	0.104186967
ENSGALG00010028032	0.827934194	0.206770762
ENSGALG00010028033	0.419651701	0.182461132
ENSGALG00010028034	0.791239597	0.314743495

ENSGALG00010028035	0.179171407	0.246488957
ENSGALG00010028036	0.945383872	0.343668477
ENSGALG00010028037	0.849165445	0.224605964
ENSGALG00010028038	0.01417792	0.172123804
ENSGALG00010028039	0.972855518	0.292332095
ENSGALG00010028040	0.934176967	0.15753134
ENSGALG00010028041	0.492697196	0.305275753
ENSGALG00010028042	0.534267653	0.02428475
ENSGALG00010028043	0.901711451	0.247296632
ENSGALG00010028044	0.017230816	0.102968279
ENSGALG00010028045	0.56497305	0.036845467
ENSGALG00010028046	0.920546623	0.364697512
ENSGALG00010028047	0.016243446	0.057520701
ENSGALG00010028048	0.91062255	0.351667646
ENSGALG00010028049	0.963452289	0.31893964
ENSGALG00010028050	0.852718785	0.380247291
ENSGALG00010028051	0.699393136	0.237781055
ENSGALG00010028052	0.740759973	0.546324371
ENSGALG00010028053	0.995880482	0.337974263
ENSGALG00010028054	0.889647012	0.275821473
ENSGALG00010028055	0.186894171	0.217495048
ENSGALG00010028056	0.934389771	0.275410129
ENSGALG00010028057	0.408721057	0.019695582
ENSGALG00010028058	0.899844628	0.179924334
ENSGALG00010028059	0.607347395	0.175057512
ENSGALG00010028060	0.89066886	0.38421711
ENSGALG00010028061	0.67590401	0.362507023
ENSGALG00010028062	0.34279423	0.134133055
ENSGALG00010028064	0.244701234	0.16673831
ENSGALG00010028065	0.835646055	0.215994113
ENSGALG00010028066	0.962013354	0.230341263
ENSGALG00010028067	0.919289568	0.188386328
ENSGALG00010028068	0.872009998	0.249569054
ENSGALG00010028069	0.29932927	0.180646537
ENSGALG00010028070	0.837722559	0.323055981
ENSGALG00010028071	0.906163213	0.097897742
ENSGALG00010028072	0.705713797	0.215542292
ENSGALG00010028073	0.989894394	0.364524542
ENSGALG00010028074	0.903453221	0.181026621
ENSGALG00010028075	0.964995762	0.335123876
ENSGALG00010028076	0.982663318	0.329147793
ENSGALG00010028077	0.365894691	0.232214944
ENSGALG00010028078	0.82395818	0.253886612
ENSGALG00010028079	0.99044456	0.283934478
ENSGALG00010028080	0.934703617	0.381541825
ENSGALG00010028081	0.798227271	0.24550553
ENSGALG00010028082	0.996367332	0.335278693
ENSGALG00010028083	0.121990842	0.189637871
ENSGALG00010028084	0.737669545	0.031347261
ENSGALG00010028085	0.701501203	0.192234501
ENSGALG00010028086	0.879184692	0.385463875
ENSGALG00010028087	0.992568801	0.270006268
ENSGALG00010028088	0.959792598	0.37113636
ENSGALG00010028089	0.415286156	0.321052467
ENSGALG00010028090	0.936208123	0.281589895
ENSGALG00010028091	0.942130638	0.243697751

ENSGALG00010028092	0.394015974	0.103970839
ENSGALG00010028093	0.900811519	0.415969639
ENSGALG00010028094	0.46399919	0.320225977
ENSGALG00010028095	0.38250133	0.114283922
ENSGALG00010028096	0.955399844	0.264260787
ENSGALG00010028097	0.960654121	0.402439912
ENSGALG00010028098	0.70971105	0.263952961
ENSGALG00010028099	0.861776014	0.211838659
ENSGALG00010028100	0.233503217	0.025146126
ENSGALG00010028101	0.961168043	0.31138096
ENSGALG00010028102	0.899308536	0.476234277
ENSGALG00010028103	0.937935362	0.263839415
ENSGALG00010028104	0.953229089	0.304869721
ENSGALG00010028105	0.985160285	0.343607225
ENSGALG00010028106	0.847597183	0.261386269
ENSGALG00010028107	0.331732426	0.057424403
ENSGALG00010028108	0.320321366	0.004604427
ENSGALG00010028109	0.905189148	0.322199289
ENSGALG00010028110	0.514678397	0.3796821
ENSGALG00010028111	0.39483326	0.099921928
ENSGALG00010028112	0.653880446	0.434988707
ENSGALG00010028113	0.741090436	0.04934692
ENSGALG00010028114	0.534948545	0.018797602
ENSGALG00010028115	0.967995203	0.32767184
ENSGALG00010028116	0.981237802	0.317302095
ENSGALG00010028117	0.964414109	0.33755039
ENSGALG00010028118	0.87749905	0.25072887
ENSGALG00010028119	0.924905933	0.324274396
ENSGALG00010028120	0.6223184	0.155574414
ENSGALG00010028121	0.977015333	0.331566223
ENSGALG00010028122	0.964946611	0.34226543
ENSGALG00010028123	0.916336341	0.380046641
ENSGALG00010028124	0.436005305	0.204504047
ENSGALG00010028125	0.859355286	0.280881542
ENSGALG00010028126	0.961618222	0.312284046
ENSGALG00010028127	0.771666131	0.34363156
ENSGALG00010028128	0.879891523	0.230284022
ENSGALG00010028129	0.911961228	0.245384229
ENSGALG00010028130	0.952082307	0.302499911
ENSGALG00010028131	0.913554294	0.380832508
ENSGALG00010028132	0.760663751	0.167021327
ENSGALG00010028133	0.816068469	0.301658128
ENSGALG00010028134	0.777095998	0.361395974
ENSGALG00010028135	0.627840989	0.083643133
ENSGALG00010028136	0.66854655	0.119252656
ENSGALG00010028137	0.591233376	0.404696013
ENSGALG00010028138	0.396080963	0.234725335
ENSGALG00010028139	0.956371083	0.278470732
ENSGALG00010028140	0.823131879	0.340554593
ENSGALG00010028141	0.732559994	0.150917192
ENSGALG00010028142	0.50454932	0.201716762
ENSGALG00010028144	0.934484675	0.260121282
ENSGALG00010028145	0.670522007	0.064040035
ENSGALG00010028146	0.733147306	0.327625448
ENSGALG00010028147	0.873972512	0.143659682
ENSGALG00010028148	0.991577109	0.312281158

ENSGALG00010028149	0.874518111	0.258040896
ENSGALG00010028150	0.6096093	0.573528567
ENSGALG00010028151	0.662722831	0.016557706
ENSGALG00010028152	0.486324452	0.695992403
ENSGALG00010028153	0.399389175	0.163283405
ENSGALG00010028154	0.049138588	0.34885672
ENSGALG00010028155	0.985286126	0.296643229
ENSGALG00010028156	0.953209041	0.321407031
ENSGALG00010028157	0.701406354	0.060324443
ENSGALG00010028158	0.662787262	0.574928682
ENSGALG00010028159	0.183641364	0.143268904
ENSGALG00010028160	0.756174438	0.260029936
ENSGALG00010028161	0.909735981	0.339164964
ENSGALG00010028162	0.890418983	0.554284061
ENSGALG00010028163	0.874497671	0.313029551
ENSGALG00010028164	0.38268392	0.37685511
ENSGALG00010028165	0.896343287	0.362447653
ENSGALG00010028166	0.061531154	0.288695837
ENSGALG00010028167	0.958235061	0.277603786
ENSGALG00010028168	0.963333987	0.334502135
ENSGALG00010028169	0.971359527	0.279320471
ENSGALG00010028170	0.916247797	0.355908404
ENSGALG00010028171	0.993864346	0.344029864
ENSGALG00010028172	0.846996183	0.270568898
ENSGALG00010028173	0.975128757	0.293427429
ENSGALG00010028174	0.827117353	0.300653246
ENSGALG00010028175	0.927639748	0.270098095
ENSGALG00010028176	0.860143302	0.203919293
ENSGALG00010028177	0.95898139	0.391482325
ENSGALG00010028178	0.947943183	0.431695166
ENSGALG00010028179	0.587386199	0.089795015
ENSGALG00010028180	0.317508343	0.006938615
ENSGALG00010028181	0.718825646	0.210516313
ENSGALG00010028182	0.913278456	0.308536994
ENSGALG00010028183	0.12558323	0.175468314
ENSGALG00010028184	0.727461212	0.231468021
ENSGALG00010028185	0.844752843	0.164508769
ENSGALG00010028186	0.953927981	0.322021971
ENSGALG00010028187	0.707442134	0.046223755
ENSGALG00010028188	0.965413089	0.287192158
ENSGALG00010028189	0.754212683	0.097615034
ENSGALG00010028190	0.911026698	0.232858834
ENSGALG00010028191	0.948110264	0.399512811
ENSGALG00010028192	0.715964996	0.303110779
ENSGALG00010028193	0.936565729	0.364190349
ENSGALG00010028194	0.857271273	0.338671359
ENSGALG00010028195	0.862089227	0.292876488
ENSGALG00010028196	0.964897913	0.274086817
ENSGALG00010028197	0.898700779	0.321724552
ENSGALG00010028198	0.94873886	0.349427188
ENSGALG00010028199	0.184255302	0.188105183
ENSGALG00010028200	0.421006053	0.290336603
ENSGALG00010028201	0.983105348	0.336840062
ENSGALG00010028202	0.94903935	0.240297848
ENSGALG00010028204	0.74106493	0.230268633
ENSGALG00010028205	0.99108802	0.298213375

ENSGALG00010028206	0.844063842	0.20921822
ENSGALG00010028207	0.908215205	0.315488625
ENSGALG00010028208	0.635705932	0.164955432
ENSGALG00010028211	0.972023346	0.271890225
ENSGALG00010028214	0.407102152	0.199420323
ENSGALG00010028215	0.271701261	0.072417625
ENSGALG00010028217	0.433096566	0.307096488
ENSGALG00010028221	0.953836811	0.233827903
ENSGALG00010028224	0.863836175	0.56169598
ENSGALG00010028225	0.317155256	0.13063615
ENSGALG00010028228	0.234771306	0.207497118
ENSGALG00010028231	0.333832984	0.163897725
ENSGALG00010028232	0.954402118	0.254440221
ENSGALG00010028233	0.304106886	0.03208708
ENSGALG00010028235	0.657675788	0.321878198
ENSGALG00010028238	0.25824367	0.193626791
ENSGALG00010028241	0.294093629	0.19319161
ENSGALG00010028242	0.180457871	0.122865108
ENSGALG00010028244	0.901053025	0.121899606
ENSGALG00010028246	0.858948828	0.037850134
ENSGALG00010028247	0.078080342	0.163556713
ENSGALG00010028248	0.62562242	0.042089194
ENSGALG00010028249	0.855198511	0.23923833
ENSGALG00010028250	0.345023716	0.157482062
ENSGALG00010028251	0.490982144	0.141261977
ENSGALG00010028252	0.323828903	0.027237309
ENSGALG00010028254	0.191186883	0.091883276
ENSGALG00010028255	0.266514019	0.223679135
ENSGALG00010028258	0.628904518	0.434953629
ENSGALG00010028259	0.798391926	0.458387828
ENSGALG00010028260	0.285533721	0.190043249
ENSGALG00010028261	0.015935051	0.173680644
ENSGALG00010028262	0.935255749	0.25599142
ENSGALG00010028263	0.015616096	0.166431014
ENSGALG00010028264	0.386719876	0.191163161
ENSGALG00010028265	0.19020302	0.31813848
ENSGALG00010028267	0.364055186	0.42690201
ENSGALG00010028268	0.169528072	0.055502654
ENSGALG00010028269	0.219386918	0.079388121
ENSGALG00010028270	0.970050129	0.248295467
ENSGALG00010028271	0.38813597	0.156768404
ENSGALG00010028272	0.314874757	0.361635101
ENSGALG00010028274	0.285179533	0.121954912
ENSGALG00010028277	0.872700884	0.306345208
ENSGALG00010028278	0.001469805	0.175131669
ENSGALG00010028281	0.062935807	0.101865633
ENSGALG00010028282	0.669949337	0.488511518
ENSGALG00010028283	0.960406589	0.316452541
ENSGALG00010028284	0.173867179	0.018916982
ENSGALG00010028287	0.159280159	0.118171765
ENSGALG00010028288	0.271699272	0.336976622
ENSGALG00010028296	0.353817108	0.280339226
ENSGALG00010028297	0.390218599	0.089311719
ENSGALG00010028298	0.991007398	0.310715915
ENSGALG00010028301	0.55532104	0.340628811
ENSGALG00010028306	0.083977401	0.264925397

ENSGALG00010028307	0.063657685	0.134567151
ENSGALG00010028308	0.396343306	0.152518913
ENSGALG00010028309	0.457678933	0.122453557
ENSGALG00010028311	0.510170206	0.501690956
ENSGALG00010028312	0.695687691	0.361337073
ENSGALG00010028313	0.839207488	0.281340433
ENSGALG00010028314	0.982799717	0.323516825
ENSGALG00010028316	0.684551278	0.23807148
ENSGALG00010028317	0.807533559	0.161415215
ENSGALG00010028318	0.223820863	0.025578236
ENSGALG00010028319	0.442711702	0.1454099
ENSGALG00010028320	0.300123619	0.204779738
ENSGALG00010028321	0.688968321	0.022773809
ENSGALG00010028322	0.298125726	0.587497397
ENSGALG00010028324	0.303942447	0.152546132
ENSGALG00010028326	0.123974279	0.324303115
ENSGALG00010028328	0.599804723	0.110158189
ENSGALG00010028329	0.401327218	0.103777949
ENSGALG00010028331	0.005675254	0.152307444
ENSGALG00010028332	0.838003105	0.26958222
ENSGALG00010028333	0.867986486	0.245498137
ENSGALG00010028334	0.917999647	0.298143312
ENSGALG00010028335	0.891595533	0.252103673
ENSGALG00010028337	0.973043181	0.294171229
ENSGALG00010028338	0.399428581	0.10718574
ENSGALG00010028339	0.669609291	0.165911192
ENSGALG00010028340	0.834364326	0.096616145
ENSGALG00010028341	0.753752784	0.100546484
ENSGALG00010028342	0.501075399	0.177682095
ENSGALG00010028343	0.208190352	0.146696756
ENSGALG00010028344	0.140282969	0.037579917
ENSGALG00010028345	0.075275665	0.128781405
ENSGALG00010028346	0.186528505	0.146846001
ENSGALG00010028347	0.714538923	0.270929631
ENSGALG00010028348	0.890825136	0.335424938
ENSGALG00010028349	0.477393685	0.189411753
ENSGALG00010028352	0.254758994	0.252732843
ENSGALG00010028354	0.575816562	0.182921777
ENSGALG00010028355	0.943282966	0.271735703
ENSGALG00010028357	0.826863542	0.240979711
ENSGALG00010028358	0.87200405	0.230061462
ENSGALG00010028359	0.032232389	0.38723197
ENSGALG00010028362	0.081124257	0.37131748
ENSGALG00010028363	0.941621606	0.266700206
ENSGALG00010028364	0.975512785	0.35212538
ENSGALG00010028365	0.520823946	0.307483387
ENSGALG00010028366	0.901264346	0.386166154
ENSGALG00010028367	0.285179533	0.121954912
ENSGALG00010028368	0.867549058	0.251714682
ENSGALG00010028369	0.551396218	0.080036539
ENSGALG00010028370	0.933759202	0.39192207
ENSGALG00010028372	0.861673252	0.337891599
ENSGALG00010028374	0.085303677	0.081706369
ENSGALG00010028375	0.119510349	0.291295431
ENSGALG00010028376	0.406942834	0.032764134
ENSGALG00010028377	0.810527411	0.45108025

ENSGALG00010028378	0.310043156	0.36908045
ENSGALG00010028380	0.921594547	0.275578097
ENSGALG00010028382	0.934153511	0.332102631
ENSGALG00010028383	0.1661022	0.376192566
ENSGALG00010028388	0.491886059	0.325801125
ENSGALG00010028389	0.187400028	0.199108296
ENSGALG00010028390	0.194390396	0.166300887
ENSGALG00010028391	0.455139312	0.129475505
ENSGALG00010028392	0.924227289	0.127028705
ENSGALG00010028393	0.385917095	0.083976977
ENSGALG00010028394	0.201458959	0.265843204
ENSGALG00010028395	0.633386799	0.322591717
ENSGALG00010028398	0.298399403	0.094963927
ENSGALG00010028399	0.902994612	0.466180497
ENSGALG00010028400	0.696814712	0.221484982
ENSGALG00010028403	0.5278691	0.342775586
ENSGALG00010028405	0.931719737	0.329341748
ENSGALG00010028407	0.197090373	0.262558979
ENSGALG00010028409	0.492765214	0.168083719
ENSGALG00010028411	0.990678018	0.338370521
ENSGALG00010028412	0.926503332	0.354485628
ENSGALG00010028413	0.903991562	0.310429052
ENSGALG00010028414	0.930377656	0.395753032
ENSGALG00010028415	0.984934259	0.314233072
ENSGALG00010028416	0.146717733	0.186292138
ENSGALG00010028417	0.611708584	0.224122606
ENSGALG00010028418	0.989932691	0.334317674
ENSGALG00010028419	0.865636596	0.41674532
ENSGALG00010028420	0.554978532	0.268297266
ENSGALG00010028421	0.428456045	0.220898209
ENSGALG00010028422	0.961343228	0.293399489
ENSGALG00010028425	0.970426829	0.294241823
ENSGALG00010028426	0.544220967	0.006676139
ENSGALG00010028427	0.085488368	0.238055966
ENSGALG00010028428	0.232744471	0.145437826
ENSGALG00010028429	0.623920385	0.011790796
ENSGALG00010028430	0.938380923	0.261762187
ENSGALG00010028431	0.729076068	0.452691386
ENSGALG00010028432	0.958138658	0.334185509
ENSGALG00010028433	0.393097515	0.246928066
ENSGALG00010028434	0.884642526	0.445703404
ENSGALG00010028435	0.799806924	0.333572259
ENSGALG00010028436	0.983999052	0.356842718
ENSGALG00010028437	0.913427677	0.538671245
ENSGALG00010028438	0.876439338	0.384991547
ENSGALG00010028439	0.114171529	0.297722856
ENSGALG00010028440	0.916382584	0.218359995
ENSGALG00010028441	0.957789311	0.326902504
ENSGALG00010028442	0.795748016	0.005433034
ENSGALG00010028443	0.260901931	0.360175578
ENSGALG00010028444	0.94357007	0.303089401
ENSGALG00010028445	0.088780292	0.506210968
ENSGALG00010028447	0.784101788	0.439597527
ENSGALG00010028448	0.919702861	0.397400188
ENSGALG00010028449	0.722170628	0.03264654
ENSGALG00010028450	0.800545214	0.145390338

ENSGALG00010028451	0.871069519	0.400888696
ENSGALG00010028452	0.809798122	0.074837915
ENSGALG00010028453	0.532576802	0.077740821
ENSGALG00010028454	0.987899209	0.320633055
ENSGALG00010028455	0.28149508	0.113236359
ENSGALG00010028456	0.971260633	0.318940917
ENSGALG00010028457	0.913605834	0.425256593
ENSGALG00010028458	0.074770747	0.07271716
ENSGALG00010028459	0.355618501	0.151276023
ENSGALG00010028460	0.921791886	0.449116748
ENSGALG00010028461	0.438438085	0.190786501
ENSGALG00010028462	0.163508537	0.011402347
ENSGALG00010028463	0.99149919	0.317537641
ENSGALG00010028464	0.602607235	0.198112452
ENSGALG00010028465	0.919315974	0.34762962
ENSGALG00010028466	0.987946026	0.358999328
ENSGALG00010028467	0.961288014	0.339014545
ENSGALG00010028468	0.47628697	0.164924065
ENSGALG00010028470	0.540987655	0.124705891
ENSGALG00010028471	0.832892498	0.329828838
ENSGALG00010028472	0.926775947	0.287813146
ENSGALG00010028473	0.968658694	0.269034246
ENSGALG00010028474	0.899034289	0.388887883
ENSGALG00010028475	0.983200123	0.271337253
ENSGALG00010028476	0.994094042	0.326997787
ENSGALG00010028477	0.350455964	0.27830169
ENSGALG00010028478	0.153308058	0.231161324
ENSGALG00010028479	0.995472424	0.350238687
ENSGALG00010028480	0.980798179	0.306811385
ENSGALG00010028481	0.674149976	0.280027664
ENSGALG00010028482	0.481949378	0.570373855
ENSGALG00010028483	0.859245925	0.294436629
ENSGALG00010028484	0.935805621	0.427273373
ENSGALG00010028485	0.988747149	0.294243674
ENSGALG00010028486	0.93227671	0.170957351
ENSGALG00010028487	0.987455068	0.340191931
ENSGALG00010028488	0.958818623	0.369475383
ENSGALG00010028489	0.770138303	0.320417131
ENSGALG00010028490	0.929519189	0.241756114
ENSGALG00010028491	0.849932353	0.225704379
ENSGALG00010028492	0.962252367	0.277063784
ENSGALG00010028493	0.959090507	0.273107897
ENSGALG00010028494	0.909482752	0.290220565
ENSGALG00010028495	0.918257664	0.313066414
ENSGALG00010028496	0.976724583	0.265231702
ENSGALG00010028497	0.319962382	0.029634672
ENSGALG00010028498	0.850233424	0.441250977
ENSGALG00010028499	0.849203007	0.269233425
ENSGALG00010028500	0.849953411	0.220039934
ENSGALG00010028501	0.932993824	0.223417983
ENSGALG00010028502	0.952949396	0.306406139
ENSGALG00010028503	0.6598265	0.339096807
ENSGALG00010028506	0.050979664	0.142981828
ENSGALG00010028507	0.970818188	0.312637533
ENSGALG00010028508	0.480232974	0.412250666
ENSGALG00010028510	0.833418194	0.027146688

ENSGALG00010028511	0.120869112	0.158641392
ENSGALG00010028512	0.475457535	0.07559826
ENSGALG00010028513	0.441438777	0.553197771
ENSGALG00010028514	0.944841712	0.326312691
ENSGALG00010028516	0.934732154	0.413907441
ENSGALG00010028517	0.294826952	0.114439813
ENSGALG00010028518	0.909266099	0.393131589
ENSGALG00010028520	0.482286234	0.152831865
ENSGALG00010028523	0.456396609	0.387658885
ENSGALG00010028525	0.993904832	0.337256312
ENSGALG00010028528	0.892998391	0.211406428
ENSGALG00010028529	0.271701261	0.072417625
ENSGALG00010028535	0.994119331	0.326295196
ENSGALG00010028536	0.945137497	0.365394335
ENSGALG00010028538	0.99421042	0.325745689
ENSGALG00010028541	0.495098378	0.303900124
ENSGALG00010028543	0.151242978	0.024739024
ENSGALG00010028544	0.880021036	0.210153605
ENSGALG00010028547	0.701281596	0.163567749
ENSGALG00010028548	0.947293682	0.272139471
ENSGALG00010028549	0.001708573	0.132604552
ENSGALG00010028550	0.977495902	0.359727732
ENSGALG00010028551	0.452558713	0.277950787
ENSGALG00010028553	0.796874164	0.051170403
ENSGALG00010028556	0.670542552	0.207303904
ENSGALG00010028559	0.915025878	0.377777237
ENSGALG00010028561	0.716031809	0.083694131
ENSGALG00010028562	0.871363557	0.001264741
ENSGALG00010028565	0.841171397	0.270891019
ENSGALG00010028566	0.431360016	0.316354216
ENSGALG00010028568	0.602680346	0.181887058
ENSGALG00010028569	0.416626919	0.015148479
ENSGALG00010028570	0.916613445	0.319669993
ENSGALG00010028571	0.230810078	0.278328982
ENSGALG00010028572	0.4409894	0.320808527
ENSGALG00010028573	0.884279773	0.366655758
ENSGALG00010028574	0.895285536	0.443762346
ENSGALG00010028575	0.681192846	0.107051716
ENSGALG00010028576	0.557080381	0.159997392
ENSGALG00010028577	0.95491254	0.254255106
ENSGALG00010028578	0.530543095	0.108416183
ENSGALG00010028579	0.754821316	0.415303213
ENSGALG00010028580	0.802205149	0.347157133
ENSGALG00010028581	0.844253665	0.281017423
ENSGALG00010028582	0.625253149	0.035973824
ENSGALG00010028583	0.771867965	0.108849973
ENSGALG00010028584	0.970429455	0.226076268
ENSGALG00010028585	0.910836376	0.270298678
ENSGALG00010028586	0.964592487	0.325847083
ENSGALG00010028587	0.20710621	0.028654473
ENSGALG00010028588	0.259798891	0.849430008
ENSGALG00010028589	0.927923509	0.170404664
ENSGALG00010028590	0.980105101	0.328901864
ENSGALG00010028591	0.218579171	0.007353185
ENSGALG00010028592	0.972999245	0.26212559
ENSGALG00010028593	0.950045067	0.300779977

ENSGALG00010028594	0.978049011	0.310550248
ENSGALG00010028595	0.994713331	0.321905236
ENSGALG00010028596	0.986862637	0.328650974
ENSGALG00010028597	0.962666559	0.294708586
ENSGALG00010028598	0.999184491	0.32932947
ENSGALG00010028599	0.989288635	0.294256249
ENSGALG00010028600	0.983058712	0.342984566
ENSGALG00010028601	0.992502295	0.325577296
ENSGALG00010028602	0.920635549	0.356703159
ENSGALG00010028603	0.936727342	0.229215839
ENSGALG00010028604	0.030971975	0.01833132
ENSGALG00010028605	0.828551812	0.311051411
ENSGALG00010028606	0.968525456	0.279902418
ENSGALG00010028607	0.942205963	0.331724776
ENSGALG00010028608	0.976751232	0.359116541
ENSGALG00010028609	0.936565978	0.353537388
ENSGALG00010028610	0.939457166	0.437428888
ENSGALG00010028611	0.854962819	0.350201483
ENSGALG00010028612	0.685890177	0.227393664
ENSGALG00010028613	0.821148199	0.268768265
ENSGALG00010028614	0.557322348	0.184745089
ENSGALG00010028615	0.088904751	0.024105003
ENSGALG00010028616	0.309655755	0.987979031
ENSGALG00010028617	0.144040418	0.155016492
ENSGALG00010028618	0.979896134	0.311263289
ENSGALG00010028619	0.365862997	0.257073287
ENSGALG00010028620	0.270851313	0.216716485
ENSGALG00010028622	0.189240572	0.04996112
ENSGALG00010028626	0.974456756	0.316235652
ENSGALG00010028627	0.386800217	0.214616758
ENSGALG00010028628	0.251056315	0.192486239
ENSGALG00010028633	0.837962202	0.414826671
ENSGALG00010028634	0.708798997	0.547343251
ENSGALG00010028635	0.51259949	0.327173137
ENSGALG00010028636	0.415585971	0.352768884
ENSGALG00010028641	0.371942231	0.168336798
ENSGALG00010028642	0.857129801	0.349623958
ENSGALG00010028643	0.513849806	0.2018346
ENSGALG00010028645	0.332566097	0.145842773
ENSGALG00010028646	0.205334133	0.18475969
ENSGALG00010028648	0.107126787	0.270085544
ENSGALG00010028649	0.925028481	0.406673146
ENSGALG00010028650	0.899139007	0.311291546
ENSGALG00010028651	0.095918798	0.385687932
ENSGALG00010028652	0.646047855	0.330587407
ENSGALG00010028653	0.217540133	0.027611342
ENSGALG00010028654	0.362179464	0.653663197
ENSGALG00010028655	0.677487142	0.126834392
ENSGALG00010028657	0.148901949	0.334499326
ENSGALG00010028658	0.933213103	0.264613419
ENSGALG00010028660	0.101500259	0.192665648
ENSGALG00010028661	0.88017148	0.471725069
ENSGALG00010028662	0.52272835	0.084598193
ENSGALG00010028663	0.570557434	0.231043008
ENSGALG00010028664	0.363397673	0.209391989
ENSGALG00010028665	0.730052098	0.252912954

ENSGALG00010028666	0.825879311	0.285773782
ENSGALG00010028667	0.103877244	0.131405303
ENSGALG00010028668	0.090150639	0.269233068
ENSGALG00010028669	0.072582137	0.360728672
ENSGALG00010028670	0.096584746	0.230715513
ENSGALG00010028671	0.717356105	0.193282083
ENSGALG00010028673	0.054470997	0.402410723
ENSGALG00010028674	0.858851214	0.383272553
ENSGALG00010028675	0.957289872	0.300563413
ENSGALG00010028676	0.960113891	0.309680516
ENSGALG00010028677	0.771571997	0.08846253
ENSGALG00010028678	0.078418949	0.131024373
ENSGALG00010028679	0.40308773	0.146014129
ENSGALG00010028680	0.092838636	0.16556646
ENSGALG00010028681	0.610788432	0.255458751
ENSGALG00010028682	0.011589493	0.110944875
ENSGALG00010028683	0.975649969	0.397527032
ENSGALG00010028684	0.855786102	0.291677561
ENSGALG00010028685	0.89924336	0.39805766
ENSGALG00010028686	0.640095604	0.382573794
ENSGALG00010028689	0.742551957	0.117506154
ENSGALG00010028690	0.440387508	0.523944502
ENSGALG00010028691	0.658593102	0.371953082
ENSGALG00010028692	0.919623358	0.397038564
ENSGALG00010028693	0.365259184	0.356937382
ENSGALG00010028694	0.700970403	0.604842321
ENSGALG00010028695	0.256350973	0.133778452
ENSGALG00010028696	0.629709439	0.268256273
ENSGALG00010028699	0.882964898	0.314460817
ENSGALG00010028700	0.804796421	0.391767411
ENSGALG00010028701	0.74555256	0.19221884
ENSGALG00010028702	0.741942672	0.370967699
ENSGALG00010028705	0.415654447	0.189116078
ENSGALG00010028706	0.173458425	0.199362002
ENSGALG00010028708	0.975215344	0.342762051
ENSGALG00010028709	0.728950546	0.249573786
ENSGALG00010028711	0.731451787	0.027399138
ENSGALG00010028712	0.131447791	0.201484131
ENSGALG00010028714	0.764773799	0.414827242
ENSGALG00010028715	0.265558089	0.028821141
ENSGALG00010028722	0.264677033	0.216624317
ENSGALG00010028723	0.059104051	0.085538612
ENSGALG00010028724	0.432922988	0.142055724
ENSGALG00010028725	0.993638364	0.359287019
ENSGALG00010028726	0.947638329	0.308103566
ENSGALG00010028727	0.833092108	0.271438322
ENSGALG00010028728	0.203991683	0.058221925
ENSGALG00010028730	0.379887859	0.102864813
ENSGALG00010028732	0.435024422	0.326149724
ENSGALG00010028733	0.370756474	0.275399566
ENSGALG00010028734	0.858362682	0.196238811
ENSGALG00010028735	0.682738314	0.080199917
ENSGALG00010028738	0.413239085	0.364941309
ENSGALG00010028739	0.804161938	0.171208125
ENSGALG00010028740	0.107931762	0.050084819
ENSGALG00010028742	0.060208389	0.611333422

ENSGALG00010028743	0.534227901	0.042476155
ENSGALG00010028744	0.989116048	0.333260469
ENSGALG00010028745	0.689430254	0.271640472
ENSGALG00010028746	0.976427553	0.320907665
ENSGALG00010028747	0.270560426	0.152183132
ENSGALG00010028748	0.856178752	0.243457892
ENSGALG00010028750	0.932212178	0.22640269
ENSGALG00010028751	0.819047142	0.571878405
ENSGALG00010028752	0.969748302	0.256057155
ENSGALG00010028753	0.138510682	0.263671194
ENSGALG00010028756	0.264239718	0.073427965
ENSGALG00010028760	0.969296733	0.282710725
ENSGALG00010028761	0.982595857	0.2675316
ENSGALG00010028762	0.695451228	0.214269561
ENSGALG00010028763	0.903366724	0.415089513
ENSGALG00010028764	0.943890396	0.18153461
ENSGALG00010028768	0.903376912	0.393023166
ENSGALG00010028771	0.913612925	0.37863935
ENSGALG00010028772	0.261891035	0.050295821
ENSGALG00010028777	0.554782995	0.2064046
ENSGALG00010028782	0.978202946	0.334896842
ENSGALG00010028783	0.185357993	0.139330399
ENSGALG00010028785	0.241608376	0.122620388
ENSGALG00010028786	0.850378787	0.484455432
ENSGALG00010028793	0.277566769	0.107857127
ENSGALG00010028794	0.482505748	0.164169546
ENSGALG00010028797	0.132583218	0.19457471
ENSGALG00010028798	0.694348348	0.294400796
ENSGALG00010028799	0.102263818	0.231665894
ENSGALG00010028800	0.119990717	0.08394926
ENSGALG00010028801	0.560759663	0.038769391
ENSGALG00010028802	0.682397829	0.265589914
ENSGALG00010028803	0.135899574	0.128824344
ENSGALG00010028805	0.046193222	0.197262472
ENSGALG00010028806	0.399695266	0.011610469
ENSGALG00010028807	0.671935532	0.223886379
ENSGALG00010028810	0.425485765	0.024326886
ENSGALG00010028811	0.022266455	0.458482365
ENSGALG00010028812	0.142866237	0.388358232
ENSGALG00010028813	0.111059707	0.204208036
ENSGALG00010028814	0.189240572	0.04996112
ENSGALG00010028815	0.587867944	0.253116799
ENSGALG00010028817	0.929043676	0.306493057
ENSGALG00010028818	0.336910174	0.272324369
ENSGALG00010028820	0.978810428	0.299832699
ENSGALG00010028821	0.810777367	0.326929511
ENSGALG00010028822	0.472034295	0.432818764
ENSGALG00010028823	0.569565773	0.312327991
ENSGALG00010028824	0.562212276	0.362160791
ENSGALG00010028825	0.404167701	0.144831306
ENSGALG00010028826	0.231848192	0.144441284
ENSGALG00010028827	0.794315943	0.289572692
ENSGALG00010028828	0.863338979	0.421123201
ENSGALG00010028829	0.482885603	0.016080589
ENSGALG00010028831	0.872954685	0.282951701
ENSGALG00010028832	0.886539737	0.204409146

ENSGALG00010028833	0.990199241	0.336153378
ENSGALG00010028834	0.884682315	0.396977583
ENSGALG00010028836	0.510958236	0.337941296
ENSGALG00010028837	0.771025187	0.228612314
ENSGALG00010028838	0.001640067	0.238025727
ENSGALG00010028839	0.142284408	0.232950918
ENSGALG00010028842	0.292351161	0.111245881
ENSGALG00010028843	0.341172574	0.238879914
ENSGALG00010028844	0.40023056	0.132187227
ENSGALG00010028845	0.235489199	0.200926615
ENSGALG00010028847	0.211991405	0.286722041
ENSGALG00010028849	0.996870045	0.333112743
ENSGALG00010028851	0.000960407	0.085810649
ENSGALG00010028853	0.827512838	0.284846491
ENSGALG00010028854	0.880997759	0.29688509
ENSGALG00010028855	0.918469705	0.243565791
ENSGALG00010028856	0.154776551	0.461347173
ENSGALG00010028857	0.70883611	0.114341113
ENSGALG00010028858	0.290366012	0.276219181
ENSGALG00010028859	0.888007386	0.317082793
ENSGALG00010028860	0.835076841	0.292431768
ENSGALG00010028861	0.949994021	0.349567226
ENSGALG00010028865	0.926983422	0.221812478
ENSGALG00010028866	0.763994682	0.080775848
ENSGALG00010028867	0.10898139	0.117849347
ENSGALG00010028869	0.985078236	0.342298004
ENSGALG00010028870	0.943995544	0.255132197
ENSGALG00010028871	0.973585095	0.403634289
ENSGALG00010028872	0.969980738	0.364146317
ENSGALG00010028873	0.977339702	0.279028282
ENSGALG00010028874	0.619308934	0.13492423
ENSGALG00010028875	0.96011965	0.323880152
ENSGALG00010028876	0.416437857	0.144697522
ENSGALG00010028878	0.751641455	0.293822193
ENSGALG00010028881	0.803912427	0.333201198
ENSGALG00010028884	0.979109272	0.262814581
ENSGALG00010028885	0.936115594	0.279365798
ENSGALG00010028887	0.014780113	0.093338841
ENSGALG00010028888	0.824986267	0.300913203
ENSGALG00010028889	0.938323188	0.282870267
ENSGALG00010028890	0.059539862	0.180376229
ENSGALG00010028891	0.817933597	0.460378755
ENSGALG00010028892	0.912617253	0.450875468
ENSGALG00010028893	0.916159434	0.207657233
ENSGALG00010028894	0.640076666	0.094129266
ENSGALG00010028896	0.949261875	0.293904084
ENSGALG00010028897	0.96307149	0.405574192
ENSGALG00010028898	0.822643833	0.26043989
ENSGALG00010028899	0.853688716	0.431232264
ENSGALG00010028900	0.823427467	0.191674757
ENSGALG00010028901	0.863596796	0.245468023
ENSGALG00010028902	0.274364069	0.161105648
ENSGALG00010028903	0.223536814	0.302581821
ENSGALG00010028904	0.733111782	0.193511353
ENSGALG00010028905	0.876358207	0.403454199
ENSGALG00010028906	0.711092839	0.376002045

ENSGALG00010028909	0.535919624	0.29097414
ENSGALG00010028912	0.917819819	0.280346647
ENSGALG00010028913	0.769924307	0.150591577
ENSGALG00010028914	0.962352839	0.251484589
ENSGALG00010028915	0.539655856	0.172468264
ENSGALG00010028916	0.196621825	0.258317461
ENSGALG00010028917	0.951333704	0.426966211
ENSGALG00010028918	0.701284223	0.029037434
ENSGALG00010028919	0.554736829	0.270151541
ENSGALG00010028921	0.983193054	0.31982648
ENSGALG00010028922	0.37867901	0.208370193
ENSGALG00010028923	0.681199044	0.352191514
ENSGALG00010028924	0.885765058	0.277919966
ENSGALG00010028925	0.985036985	0.368212209
ENSGALG00010028926	0.229514601	0.252459805
ENSGALG00010028930	0.271701261	0.072417625
ENSGALG00010028931	0.749128298	0.276849937
ENSGALG00010028932	0.122172626	0.391373971
ENSGALG00010028935	0.276156205	0.230511396
ENSGALG00010028936	0.262939219	0.025300248
ENSGALG00010028937	0.378356569	0.111059644
ENSGALG00010028939	0.41124688	0.494068853
ENSGALG00010028940	0.216433634	0.680598293
ENSGALG00010028943	0.327171109	0.165614547
ENSGALG00010028945	0.369201932	0.878545561
ENSGALG00010028948	0.309655755	0.987979031
ENSGALG00010028949	0.627875785	0.180920851
ENSGALG00010028951	0.490247427	0.088932749
ENSGALG00010028957	0.309677636	0.988082733
ENSGALG00010028960	0.310184688	0.17631624
ENSGALG00010028962	0.34081212	0.34730063
ENSGALG00010028963	0.40407931	0.24317184
ENSGALG00010028966	0.012368823	0.162842107
ENSGALG00010028967	0.393634217	0.115318426
ENSGALG00010028972	0.237514972	0.409481613
ENSGALG00010028973	0.458750443	0.177069425
ENSGALG00010028980	0.020656918	0.064774402
ENSGALG00010028981	0.476711311	0.203859313
ENSGALG00010028983	0.398511795	0.272244852
ENSGALG00010028984	0.081791238	0.074811512
ENSGALG00010028989	0.234345033	0.123532749
ENSGALG00010028990	0.429385192	0.113102805
ENSGALG00010028991	0.115305899	0.328817113
ENSGALG00010028992	0.197061897	0.237610802
ENSGALG00010028993	0.076469307	0.242971074
ENSGALG00010028994	0.396668506	0.181671381
ENSGALG00010028995	0.372796048	0.167811456
ENSGALG00010028997	0.715019265	0.056469794
ENSGALG00010028998	0.271701261	0.072417625
ENSGALG00010029000	0.739995572	0.312935623
ENSGALG00010029001	0.388293838	0.285628693
ENSGALG00010029005	0.200570274	0.091667991
ENSGALG00010029007	0.668780928	0.193518964
ENSGALG00010029009	0.271701261	0.072417625
ENSGALG00010029011	0.064206171	0.20106995
ENSGALG00010029021	0.263966013	0.445665299

ENSGALG00010029022	0.419611914	0.482493353
ENSGALG00010029023	0.948207778	0.34686547
ENSGALG00010029024	0.819798795	0.199099035
ENSGALG00010029025	0.473415435	0.206300472
ENSGALG00010029026	0.453081598	0.284083708
ENSGALG00010029027	0.383211517	0.152712488
ENSGALG00010029028	0.631436966	0.437858499
ENSGALG00010029029	0.138101678	0.223751001
ENSGALG00010029030	0.464292879	0.521619199
ENSGALG00010029031	0.810050852	0.266439075
ENSGALG00010029035	0.276222946	0.413406195
ENSGALG00010029038	0.180005877	0.055220627
ENSGALG00010029039	0.450975048	0.209694345
ENSGALG00010029042	0.201497347	0.289630029
ENSGALG00010029047	0.131594986	0.150403193
ENSGALG00010029048	0.796564942	0.37658117
ENSGALG00010029049	0.570541241	0.31797224
ENSGALG00010029050	0.357479516	0.140501891
ENSGALG00010029053	0.298014721	0.246852884
ENSGALG00010029056	0.553919903	0.379340892
ENSGALG00010029057	0.480373529	0.01967922
ENSGALG00010029058	0.558657554	0.003269642
ENSGALG00010029059	0.266137114	0.107784983
ENSGALG00010029065	0.164390601	0.253906274
ENSGALG00010029066	0.00989989	0.414012244
ENSGALG00010029069	0.423276176	0.613019395
ENSGALG00010029070	0.313696025	0.350296666
ENSGALG00010029071	0.370930594	0.701731144
ENSGALG00010029072	0.44463987	0.22173105
ENSGALG00010029073	0.447613441	0.524085947
ENSGALG00010029074	0.377445378	0.160177126
ENSGALG00010029076	0.90160722	0.191127693
ENSGALG00010029078	0.300683727	0.461762941
ENSGALG00010029083	0.05197934	0.14495833
ENSGALG00010029084	0.418385807	0.202957738
ENSGALG00010029085	0.332752772	0.064402972
ENSGALG00010029086	0.38480368	0.180523942
ENSGALG00010029087	0.462726142	0.107104348
ENSGALG00010029091	0.223829944	0.334880464
ENSGALG00010029092	0.081738198	0.084882396
ENSGALG00010029094	0.143769036	0.164222446
ENSGALG00010029100	0.548312897	0.099516943
ENSGALG00010029101	0.007242584	0.179665658
ENSGALG00010029102	0.802808539	0.413510627
ENSGALG00010029103	0.37300765	0.246974419
ENSGALG00010029104	0.122850389	0.45041869
ENSGALG00010029105	0.039141949	0.214534127
ENSGALG00010029106	0.456573993	0.062879435
ENSGALG00010029107	0.721945569	0.244187138
ENSGALG00010029109	0.983818776	0.334131535
ENSGALG00010029110	0.285272007	0.109169955
ENSGALG00010029111	0.316866425	0.158245935
ENSGALG00010029114	0.264239718	0.073427965
ENSGALG00010029115	0.461643443	0.006569855
ENSGALG00010029116	0.474944767	0.095250436
ENSGALG00010029118	0.385011888	0.139928827

ENSGALG00010029119	0.730280825	0.239610101
ENSGALG00010029120	0.453458843	0.187889608
ENSGALG00010029121	0.324102667	0.023744924
ENSGALG00010029122	0.262922434	0.138601464
ENSGALG00010029124	0.10019489	0.316009192
ENSGALG00010029125	0.476017749	0.17850825
ENSGALG00010029126	0.417776658	0.081103746
ENSGALG00010029127	0.496746903	0.024733722
ENSGALG00010029128	0.66621252	0.303190969
ENSGALG00010029132	0.007420952	0.120965845
ENSGALG00010029134	0.11668492	0.19461373
ENSGALG00010029136	0.132120601	0.175896385
ENSGALG00010029137	0.494740301	0.258198608
ENSGALG00010029140	0.809994521	0.298551193
ENSGALG00010029141	0.988709152	0.314316153
ENSGALG00010029142	0.638233944	0.319686785
ENSGALG00010029143	0.744944869	0.22281256
ENSGALG00010029144	0.991881937	0.296653808
ENSGALG00010029145	0.01516189	0.13320567
ENSGALG00010029146	0.970700383	0.339694799
ENSGALG00010029147	0.894939905	0.2994993
ENSGALG00010029148	0.843832015	0.360827586
ENSGALG00010029149	0.262360613	0.235488877
ENSGALG00010029150	0.249348091	0.113349243
ENSGALG00010029151	0.944838329	0.180072443
ENSGALG00010029152	0.285284598	0.406142071
ENSGALG00010029153	0.940676418	0.361739034
ENSGALG00010029154	0.549753698	0.402657339
ENSGALG00010029155	0.836226773	0.518716846
ENSGALG00010029158	0.953070051	0.275196582
ENSGALG00010029159	0.135949415	0.000248613
ENSGALG00010029160	0.859193621	0.299048562
ENSGALG00010029161	0.993898242	0.336270377
ENSGALG00010029162	0.849443431	0.489887035
ENSGALG00010029163	0.977048155	0.267495135
ENSGALG00010029164	0.965401846	0.344740428
ENSGALG00010029165	0.89871675	0.209179151
ENSGALG00010029166	0.881908447	0.26286489
ENSGALG00010029167	0.029819876	0.115613244
ENSGALG00010029168	0.612694238	0.16714768
ENSGALG00010029169	0.719200548	0.379472271
ENSGALG00010029170	0.989950672	0.27924424
ENSGALG00010029171	0.841061032	0.236612873
ENSGALG00010029172	0.724030438	0.124514873
ENSGALG00010029173	0.943382319	0.33034851
ENSGALG00010029175	0.631871146	0.0793096
ENSGALG00010029176	0.96364414	0.253651758
ENSGALG00010029177	0.796647838	0.252731434
ENSGALG00010029178	0.972814649	0.297188106
ENSGALG00010029179	0.008638603	0.404093717
ENSGALG00010029180	0.908786125	0.295715379
ENSGALG00010029181	0.938378248	0.248938321
ENSGALG00010029182	0.19888029	0.549654377
ENSGALG00010029183	0.986050205	0.375621746
ENSGALG00010029184	0.959745243	0.34361537
ENSGALG00010029185	0.959663245	0.338744594

ENSGALG00010029186	0.900099644	0.319981707
ENSGALG00010029187	0.914620069	0.509740154
ENSGALG00010029188	0.979457493	0.378594492
ENSGALG00010029189	0.827101461	0.277385834
ENSGALG00010029190	0.989889648	0.310633792
ENSGALG00010029191	0.975100978	0.308444965
ENSGALG00010029192	0.96550911	0.350724296
ENSGALG00010029193	0.451465204	0.128206002
ENSGALG00010029194	0.540093666	0.019739162
ENSGALG00010029195	0.133535385	0.23320484
ENSGALG00010029196	0.884762045	0.328594923
ENSGALG00010029197	0.303021111	0.156563333
ENSGALG00010029198	0.621906136	0.352621278
ENSGALG00010029199	0.219217366	0.5574567
ENSGALG00010029200	0.350591321	0.039099589
ENSGALG00010029201	0.553793219	0.130376506
ENSGALG00010029202	0.9673714	0.313729259
ENSGALG00010029203	0.035319214	0.390637982
ENSGALG00010029204	0.421606057	0.124690281
ENSGALG00010029205	0.985755867	0.319528026
ENSGALG00010029206	0.612794338	0.252115504
ENSGALG00010029207	0.056394145	0.343211141
ENSGALG00010029208	0.308025201	0.07234198
ENSGALG00010029209	0.943524943	0.279528668
ENSGALG00010029210	0.455122004	0.035069526
ENSGALG00010029211	0.541015114	0.393082032
ENSGALG00010029212	0.953748356	0.283087657
ENSGALG00010029213	0.997220432	0.318165417
ENSGALG00010029214	0.995195275	0.318947863
ENSGALG00010029215	0.99892395	0.327637273
ENSGALG00010029216	0.920026788	0.335523641
ENSGALG00010029217	0.15260558	0.217190615
ENSGALG00010029218	0.878029615	0.301700166
ENSGALG00010029219	0.182290514	0.043764392
ENSGALG00010029220	0.285425135	0.016324676
ENSGALG00010029221	0.931974747	0.353415255
ENSGALG00010029222	0.768556466	0.242947478
ENSGALG00010029223	0.808082202	0.370163745
ENSGALG00010029224	0.539512166	0.118202645
ENSGALG00010029225	0.665376765	0.352078638
ENSGALG00010029226	0.923714022	0.275090758
ENSGALG00010029227	0.426631115	0.302196951
ENSGALG00010029228	0.716922185	0.094256081
ENSGALG00010029229	0.868557997	0.150066517
ENSGALG00010029230	0.94706583	0.380879262
ENSGALG00010029231	0.426926433	0.176728923
ENSGALG00010029232	0.800926146	0.283489893
ENSGALG00010029233	0.598624142	0.440042723
ENSGALG00010029234	0.422037886	0.238728387
ENSGALG00010029235	0.86093373	0.246675818
ENSGALG00010029236	0.583515313	0.179560718
ENSGALG00010029237	0.388194624	0.175916204
ENSGALG00010029238	0.889492655	0.358212569
ENSGALG00010029239	0.095907681	0.418447317
ENSGALG00010029240	0.874955377	0.422797818
ENSGALG00010029241	0.049817691	0.215035813

ENSGALG00010029242	0.921002761	0.367405337
ENSGALG00010029243	0.028947784	0.017742903
ENSGALG00010029244	0.732659654	0.130649255
ENSGALG00010029245	0.585867347	0.041110428
ENSGALG00010029246	0.715553499	0.139825036
ENSGALG00010029247	0.980849193	0.348899418
ENSGALG00010029248	0.598371231	0.185880253
ENSGALG00010029249	0.410478445	0.130338355
ENSGALG00010029250	0.88692493	0.342841877
ENSGALG00010029251	0.548103956	0.215254737
ENSGALG00010029252	0.674493304	0.138032081
ENSGALG00010029253	0.352261695	0.077439344
ENSGALG00010029254	0.956325023	0.347011851
ENSGALG00010029255	0.694915518	0.108884916
ENSGALG00010029256	0.719217435	0.007892197
ENSGALG00010029257	0.362097152	0.096916306
ENSGALG00010029258	0.529636464	0.119310852
ENSGALG00010029259	0.865095777	0.178433723
ENSGALG00010029260	0.641980954	0.195013804
ENSGALG00010029261	0.725458062	0.197257811
ENSGALG00010029262	0.976280094	0.285383967
ENSGALG00010029263	0.905465474	0.285482906
ENSGALG00010029264	0.659626039	0.314569136
ENSGALG00010029265	0.981789345	0.315427321
ENSGALG00010029266	0.876029988	0.417789344
ENSGALG00010029267	0.725759261	0.257266845
ENSGALG00010029268	0.95290615	0.249826495
ENSGALG00010029269	0.916735154	0.396968122
ENSGALG00010029270	0.850634039	0.441582982
ENSGALG00010029271	0.026193426	0.299497212
ENSGALG00010029272	0.831204692	0.087085049
ENSGALG00010029273	0.888961126	0.146733609
ENSGALG00010029274	0.942152266	0.203171419
ENSGALG00010029275	0.978698482	0.326660493
ENSGALG00010029276	0.706661014	0.293606817
ENSGALG00010029277	0.601038405	0.254077623
ENSGALG00010029278	0.855366681	0.261586886
ENSGALG00010029279	0.881558795	0.290952831
ENSGALG00010029280	0.93058034	0.315387798
ENSGALG00010029281	0.336296958	0.249498879
ENSGALG00010029282	0.674260577	0.381113975
ENSGALG00010029283	0.210852824	0.339939127
ENSGALG00010029284	0.945699421	0.31945447
ENSGALG00010029285	0.105018858	0.014356426
ENSGALG00010029286	0.784445298	0.320017624
ENSGALG00010029287	0.961145465	0.342461528
ENSGALG00010029288	0.884813464	0.240298675
ENSGALG00010029289	0.869528824	0.195190514
ENSGALG00010029290	0.937087405	0.247176879
ENSGALG00010029291	0.926765013	0.307325664
ENSGALG00010029292	0.692650615	0.125787178
ENSGALG00010029293	0.840469612	0.381624086
ENSGALG00010029294	0.187404371	0.118968722
ENSGALG00010029295	0.815028248	0.367058808
ENSGALG00010029296	0.682163534	0.142365416
ENSGALG00010029297	0.960503625	0.314215342

ENSGALG00010029298	0.126314084	0.271012273
ENSGALG00010029299	0.913767996	0.346295358
ENSGALG00010029300	0.808731648	0.243325066
ENSGALG00010029301	0.936022963	0.383423524
ENSGALG00010029302	0.923952367	0.395747597
ENSGALG00010029303	0.764430354	0.227468943
ENSGALG00010029304	0.469883057	0.487864319
ENSGALG00010029305	0.991029133	0.351310648
ENSGALG00010029306	0.063096039	0.072309363
ENSGALG00010029307	0.918774345	0.339853491
ENSGALG00010029308	0.841625014	0.366938304
ENSGALG00010029309	0.97594425	0.273621112
ENSGALG00010029310	0.216160673	0.192054217
ENSGALG00010029311	0.855751639	0.15977216
ENSGALG00010029312	0.386416735	0.10974354
ENSGALG00010029313	0.66235366	0.21644103
ENSGALG00010029314	0.659436748	0.271316035
ENSGALG00010029315	0.888828306	0.268285672
ENSGALG00010029316	0.899334897	0.371062548
ENSGALG00010029317	0.667519842	0.13130821
ENSGALG00010029318	0.946339409	0.39014473
ENSGALG00010029319	0.883689442	0.015816329
ENSGALG00010029321	0.899860301	0.149733698
ENSGALG00010029322	0.905526461	0.331190243
ENSGALG00010029323	0.934085789	0.166269647
ENSGALG00010029324	0.955104805	0.345834732
ENSGALG00010029325	0.210477708	0.351392496
ENSGALG00010029326	0.555613282	0.442045446
ENSGALG00010029327	0.824429744	0.260392127
ENSGALG00010029328	0.959737879	0.336090717
ENSGALG00010029329	0.281520391	0.237858739
ENSGALG00010029330	0.978118323	0.375062797
ENSGALG00010029331	0.785832885	0.161207976
ENSGALG00010029332	0.932028271	0.200783883
ENSGALG00010029333	0.572505202	0.032949131
ENSGALG00010029336	0.997160552	0.348422617
ENSGALG00010029337	0.949739061	0.289739116
ENSGALG00010029338	0.949804487	0.297498265
ENSGALG00010029339	0.776097663	0.187293763
ENSGALG00010029340	0.856797526	0.262658902
ENSGALG00010029341	0.319547257	0.223317377
ENSGALG00010029342	0.082107063	0.174000743
ENSGALG00010029343	0.937931984	0.282244306
ENSGALG00010029344	0.895716438	0.16208545
ENSGALG00010029345	0.261412418	0.083069029
ENSGALG00010029346	0.885121303	0.261529458
ENSGALG00010029347	0.38244303	0.126083347
ENSGALG00010029348	0.749322918	0.241043573
ENSGALG00010029349	0.952136342	0.231862096
ENSGALG00010029350	0.868928309	0.342134098
ENSGALG00010029351	0.982854244	0.28131335
ENSGALG00010029352	0.173030078	0.06482495
ENSGALG00010029353	0.976645018	0.278254129
ENSGALG00010029354	0.347015121	0.336051287
ENSGALG00010029355	0.978391376	0.310144655
ENSGALG00010029356	0.717582264	0.114217884

ENSGALG00010029357	0.933305179	0.193857225
ENSGALG00010029359	0.236877526	0.13201527
ENSGALG00010029360	0.936959532	0.294284709
ENSGALG00010029361	0.44471812	0.265435523
ENSGALG00010029362	0.770990329	0.212682408
ENSGALG00010029363	0.769106971	0.424481466
ENSGALG00010029364	0.973172983	0.415377412
ENSGALG00010029365	0.938098104	0.317062191
ENSGALG00010029366	0.861585085	0.376244793
ENSGALG00010029367	0.801487907	0.054867863
ENSGALG00010029368	0.229589011	0.585814115
ENSGALG00010029369	0.673036286	0.420330574
ENSGALG00010029370	0.739491179	0.37509867
ENSGALG00010029371	0.466343761	0.337248842
ENSGALG00010029372	0.823316881	0.163563693
ENSGALG00010029373	0.957898711	0.286683228
ENSGALG00010029374	0.576675461	0.285415698
ENSGALG00010029375	0.969255832	0.323993941
ENSGALG00010029376	0.89069278	0.401539082
ENSGALG00010029377	0.927593371	0.281079928
ENSGALG00010029378	0.573697331	0.438979747
ENSGALG00010029379	0.48947446	0.003163972
ENSGALG00010029380	0.778696451	0.375716312
ENSGALG00010029381	0.836382543	0.346169195
ENSGALG00010029382	0.988353374	0.308007015
ENSGALG00010029383	0.81161633	0.37003118
ENSGALG00010029384	0.881052685	0.055257723
ENSGALG00010029385	0.771088078	0.544199108
ENSGALG00010029386	0.743832438	0.415160891
ENSGALG00010029387	0.930205064	0.224740284
ENSGALG00010029388	0.741662948	0.19328328
ENSGALG00010029389	0.014815925	0.283718791
ENSGALG00010029390	0.354934685	0.343618001
ENSGALG00010029391	0.929981838	0.278031351
ENSGALG00010029392	0.248447067	0.048863123
ENSGALG00010029393	0.291637091	0.096160096
ENSGALG00010029394	0.971987471	0.394967954
ENSGALG00010029395	0.117716651	0.35180016
ENSGALG00010029396	0.970439553	0.324382292
ENSGALG00010029397	0.666661333	0.422564617
ENSGALG00010029398	0.354887295	0.335494438
ENSGALG00010029399	0.978707916	0.28090998
ENSGALG00010029400	0.858156381	0.330177021
ENSGALG00010029401	0.776503048	0.268092038
ENSGALG00010029402	0.723104072	0.227610718
ENSGALG00010029403	0.973531834	0.306383523
ENSGALG00010029404	0.383349556	0.308465858
ENSGALG00010029405	0.726497674	0.13712705
ENSGALG00010029406	0.304653396	0.075761233
ENSGALG00010029407	0.777726276	0.126458712
ENSGALG00010029408	0.985865216	0.353444277
ENSGALG00010029409	0.967046596	0.289808907
ENSGALG00010029410	0.990611573	0.306814748
ENSGALG00010029411	0.973513434	0.290105172
ENSGALG00010029412	0.805858103	0.180887459
ENSGALG00010029413	0.497151878	0.184431313

ENSGALG00010029414	0.802404966	0.30563552
ENSGALG00010029416	0.326286323	0.130037105
ENSGALG00010029417	0.82759108	0.251268499
ENSGALG00010029418	0.880650469	0.296654605
ENSGALG00010029419	0.769588531	0.23928495
ENSGALG00010029420	0.53839665	0.343660313
ENSGALG00010029421	0.975839697	0.278936643
ENSGALG00010029422	0.93104502	0.257355422
ENSGALG00010029423	0.450151842	0.049406798
ENSGALG00010029424	0.841915434	0.368939249
ENSGALG00010029425	0.884297043	0.353840055
ENSGALG00010029427	0.528307017	0.181203273
ENSGALG00010029428	0.973840632	0.384637472
ENSGALG00010029429	0.990989359	0.298405767
ENSGALG00010029430	0.899862067	0.379837684
ENSGALG00010029431	0.364130097	0.168510939
ENSGALG00010029432	0.494369564	0.164218257
ENSGALG00010029434	0.509499309	0.420972333
ENSGALG00010029436	0.170227232	0.012252927
ENSGALG00010029437	0.548096646	0.16498867
ENSGALG00010029439	0.358112066	0.164961849
ENSGALG00010029440	0.973787883	0.247145933
ENSGALG00010029442	0.038638171	0.109562364
ENSGALG00010029443	0.090670275	0.261041598
ENSGALG00010029445	0.665897109	0.369695753
ENSGALG00010029446	0.968987974	0.245538753
ENSGALG00010029447	0.274885481	0.169614777
ENSGALG00010029449	0.970475289	0.258943281
ENSGALG00010029451	0.090102811	0.295791431
ENSGALG00010029452	0.2909684	0.148873921
ENSGALG00010029453	0.372844675	0.121260847
ENSGALG00010029456	0.735253355	0.424713995
ENSGALG00010029459	0.505711214	0.23408374
ENSGALG00010029463	0.05197934	0.14495833
ENSGALG00010029465	0.264086386	0.150531706
ENSGALG00010029466	0.957385211	0.186526233
ENSGALG00010029467	0.348489793	0.158514494
ENSGALG00010029469	0.395418784	0.135316935
ENSGALG00010029470	0.595369388	0.305111527
ENSGALG00010029473	0.929445523	0.206411483
ENSGALG00010029474	0.061332302	0.385743211
ENSGALG00010029475	0.296726552	0.12798907
ENSGALG00010029476	0.360302963	0.034133254
ENSGALG00010029477	0.697281771	0.087636018
ENSGALG00010029478	0.369586233	0.175264494
ENSGALG00010029479	0.026013658	0.201342713
ENSGALG00010029480	0.076687646	0.231573049
ENSGALG00010029481	0.384893753	0.071722217
ENSGALG00010029482	0.821829984	0.097302883
ENSGALG00010029483	0.229565439	0.252046404
ENSGALG00010029484	0.703560225	0.15439048
ENSGALG00010029486	0.568204855	0.246630293
ENSGALG00010029487	0.431385704	0.175633551
ENSGALG00010029488	0.737688716	0.262881879
ENSGALG00010029489	0.42642119	0.122522338
ENSGALG00010029490	0.203991683	0.058221925

ENSGALG00010029491	0.572057954	0.289273991
ENSGALG00010029492	0.630149953	0.446748666
ENSGALG00010029494	0.084879627	0.073141606
ENSGALG00010029495	0.936421338	0.301388366
ENSGALG00010029496	0.585131293	0.469470978
ENSGALG00010029497	0.032345365	0.334658117
ENSGALG00010029498	0.953258309	0.361046138
ENSGALG00010029499	0.569296062	0.228185068
ENSGALG00010029500	0.277566769	0.107857127
ENSGALG00010029501	0.062039976	0.134108662
ENSGALG00010029502	0.515473214	0.395405172
ENSGALG00010029503	0.266135033	0.10195952
ENSGALG00010029504	0.566443797	0.123466428
ENSGALG00010029505	0.968166068	0.234522686
ENSGALG00010029506	0.627849049	0.046221486
ENSGALG00010029507	0.382982725	0.066000061
ENSGALG00010029508	0.257752143	0.306620164
ENSGALG00010029509	0.937233615	0.362589504
ENSGALG00010029510	0.121214993	0.131654747
ENSGALG00010029511	0.962550538	0.311609701
ENSGALG00010029512	0.932854772	0.290197856
ENSGALG00010029513	0.295314363	0.052564455
ENSGALG00010029514	0.965858513	0.339405559
ENSGALG00010029515	0.966650769	0.344256203
ENSGALG00010029516	0.971943431	0.361430464
ENSGALG00010029517	0.966286951	0.223775747
ENSGALG00010029518	0.521646979	0.210628976
ENSGALG00010029519	0.434516781	0.076508357
ENSGALG00010029520	0.807693478	0.206145068
ENSGALG00010029521	0.45402556	0.028215975
ENSGALG00010029522	0.722953661	0.379128089
ENSGALG00010029523	0.515218569	0.750526749
ENSGALG00010029524	0.957146225	0.259047816
ENSGALG00010029525	0.980714679	0.263190064
ENSGALG00010029526	0.982020314	0.357646589
ENSGALG00010029527	0.977045088	0.313971162
ENSGALG00010029528	0.532717117	0.4749944
ENSGALG00010029529	0.599920746	0.329729189
ENSGALG00010029530	0.984338765	0.386445356
ENSGALG00010029531	0.952146393	0.269886431
ENSGALG00010029532	0.723711551	0.12222211
ENSGALG00010029533	0.946073606	0.283586021
ENSGALG00010029534	0.931608848	0.27235028
ENSGALG00010029536	0.95530849	0.314820188
ENSGALG00010029537	0.984617241	0.326837298
ENSGALG00010029538	0.775472075	0.27727794
ENSGALG00010029539	0.222778	0.14046234
ENSGALG00010029540	0.102362161	0.104164023
ENSGALG00010029541	0.978134655	0.317011959
ENSGALG00010029542	0.628933055	0.080416398
ENSGALG00010029543	0.845951787	0.339753034
ENSGALG00010029544	0.882687397	0.335960074
ENSGALG00010029545	0.738779747	0.002153666
ENSGALG00010029546	0.957172041	0.33848504
ENSGALG00010029547	0.984840743	0.318301002
ENSGALG00010029548	0.984166834	0.333595347

ENSGALG00010029549	0.916746786	0.317105635
ENSGALG00010029550	0.958053094	0.328520052
ENSGALG00010029552	0.791104586	0.226860457
ENSGALG00010029553	0.985701039	0.330137903
ENSGALG00010029554	0.981807108	0.315939797
ENSGALG00010029555	0.205650116	0.322334566
ENSGALG00010029556	0.901865358	0.315686418
ENSGALG00010029557	0.99413505	0.336099963
ENSGALG00010029558	0.880460169	0.241041517
ENSGALG00010029559	0.91595601	0.419228024
ENSGALG00010029560	0.7575435	0.196735602
ENSGALG00010029561	0.346021511	0.369462447
ENSGALG00010029562	0.722450394	0.412946245
ENSGALG00010029563	0.616969383	0.444590879
ENSGALG00010029564	0.719022763	0.320294024
ENSGALG00010029565	0.397725943	0.173871128
ENSGALG00010029566	0.984394125	0.362220664
ENSGALG00010029567	0.982092049	0.329582978
ENSGALG00010029568	0.178108277	0.124234799
ENSGALG00010029569	0.849921222	0.262024598
ENSGALG00010029570	0.932398585	0.376308922
ENSGALG00010029571	0.906491966	0.248867545
ENSGALG00010029572	0.504909531	0.312361421
ENSGALG00010029573	0.921162088	0.277067154
ENSGALG00010029574	0.758266409	0.134484667
ENSGALG00010029575	0.353460169	0.153659574
ENSGALG00010029576	0.892858988	0.111266538
ENSGALG00010029577	0.105614442	0.105420647
ENSGALG00010029578	0.423670969	0.253752419
ENSGALG00010029579	0.5867965	0.436556664
ENSGALG00010029580	0.880643402	0.08582166
ENSGALG00010029581	0.853755025	0.271203933
ENSGALG00010029582	0.988813204	0.287259219
ENSGALG00010029583	0.914100586	0.300953985
ENSGALG00010029584	0.979234444	0.367539545
ENSGALG00010029585	0.686688883	0.41250708
ENSGALG00010029586	0.854110646	0.325112357
ENSGALG00010029587	0.064059294	0.306337965
ENSGALG00010029588	0.965386313	0.318082597
ENSGALG00010029589	0.144560807	0.137212893
ENSGALG00010029590	0.731479905	0.059959051
ENSGALG00010029591	0.939968926	0.40062819
ENSGALG00010029592	0.164341713	0.058562557
ENSGALG00010029593	0.928768888	0.334291596
ENSGALG00010029594	0.979064313	0.253504255
ENSGALG00010029595	0.889630914	0.343488753
ENSGALG00010029596	0.95102668	0.317264072
ENSGALG00010029597	0.965961857	0.305318349
ENSGALG00010029598	0.139172544	0.119786931
ENSGALG00010029599	0.994126471	0.360679418
ENSGALG00010029600	0.912427531	0.246418344
ENSGALG00010029601	0.895859325	0.273574544
ENSGALG00010029602	0.981368804	0.406397479
ENSGALG00010029603	0.139316547	0.121525453
ENSGALG00010029604	0.896881416	0.265457597
ENSGALG00010029605	0.946489343	0.343811363

ENSGALG00010029606	0.576247137	0.238750469
ENSGALG00010029607	0.538275946	0.117753848
ENSGALG00010029608	0.9793059	0.370977895
ENSGALG00010029609	0.949946557	0.439563005
ENSGALG00010029610	0.985589659	0.305617132
ENSGALG00010029611	0.971978797	0.32733802
ENSGALG00010029612	0.988867994	0.32105186
ENSGALG00010029613	0.353767897	0.186851719
ENSGALG00010029614	0.930115671	0.305615211
ENSGALG00010029615	0.56614739	0.332793814
ENSGALG00010029616	0.965803044	0.347048975
ENSGALG00010029617	0.982603832	0.326566227
ENSGALG00010029618	0.924184058	0.14621009
ENSGALG00010029619	0.875961462	0.397391212
ENSGALG00010029620	0.692899444	0.259612608
ENSGALG00010029621	0.080604005	0.142749464
ENSGALG00010029622	0.964714182	0.358601689
ENSGALG00010029623	0.732922526	0.286611107
ENSGALG00010029624	0.553524565	0.059085326
ENSGALG00010029625	0.383290923	0.228090811
ENSGALG00010029626	0.092131694	0.031403681
ENSGALG00010029627	0.289908941	0.060469215
ENSGALG00010029628	0.995316391	0.330749356
ENSGALG00010029629	0.835413178	0.434729396
ENSGALG00010029630	0.022664457	0.033414052
ENSGALG00010029631	0.86390738	0.486958628
ENSGALG00010029632	0.953000563	0.340092547
ENSGALG00010029633	0.743607491	0.295516909
ENSGALG00010029634	0.986584122	0.337278036
ENSGALG00010029635	0.926711889	0.28650608
ENSGALG00010029636	0.883690597	0.270699199
ENSGALG00010029638	0.964410834	0.286849895
ENSGALG00010029639	0.067004358	0.159893419
ENSGALG00010029641	0.429964376	0.167835486
ENSGALG00010029642	0.98823935	0.339999874
ENSGALG00010029643	0.277566769	0.107857127
ENSGALG00010029644	0.211967288	0.186486131
ENSGALG00010029645	0.535575212	0.109766377
ENSGALG00010029646	0.923232147	0.337330869
ENSGALG00010029647	0.786378825	0.195655639
ENSGALG00010029648	0.954146995	0.299373224
ENSGALG00010029649	0.980519367	0.33124782
ENSGALG00010029650	0.976603057	0.335848751
ENSGALG00010029651	0.923636373	0.363047471
ENSGALG00010029652	0.723259734	0.088943588
ENSGALG00010029653	0.98630906	0.335562515
ENSGALG00010029654	0.914447301	0.321867915
ENSGALG00010029655	0.998949766	0.332091847
ENSGALG00010029656	0.974860017	0.300704733
ENSGALG00010029657	0.984085278	0.349975437
ENSGALG00010029658	0.481477	0.303834297
ENSGALG00010029659	0.891429633	0.20349327
ENSGALG00010029660	0.937925855	0.193468917
ENSGALG00010029661	0.617711969	0.032243239
ENSGALG00010029662	0.549245171	0.193332842
ENSGALG00010029663	0.979687017	0.256405297

ENSGALG00010029664	0.95946444	0.240207791
ENSGALG00010029665	0.890932049	0.101476927
ENSGALG00010029666	0.979497017	0.35802556
ENSGALG00010029667	0.792418338	0.218868137
ENSGALG00010029668	0.91464091	0.168717
ENSGALG00010029669	0.753718308	0.362825839
ENSGALG00010029670	0.972692887	0.318693142
ENSGALG00010029671	0.681816794	0.404037534
ENSGALG00010029672	0.5971229	0.284720316
ENSGALG00010029673	0.9040481	0.289165293
ENSGALG00010029674	0.961735161	0.331588739
ENSGALG00010029675	0.909761144	0.164277892
ENSGALG00010029676	0.99167741	0.339098601
ENSGALG00010029677	0.856641707	0.133037457
ENSGALG00010029679	0.948249763	0.339288626
ENSGALG00010029680	0.884839882	0.312822911
ENSGALG00010029681	0.565241436	0.275769557
ENSGALG00010029682	0.620424513	0.431351966
ENSGALG00010029683	0.932095036	0.278544979
ENSGALG00010029684	0.691449381	0.20609873
ENSGALG00010029685	0.78606913	0.305829756
ENSGALG00010029686	0.695450315	0.205895444
ENSGALG00010029687	0.989376421	0.350463601
ENSGALG00010029688	0.985562303	0.300328155
ENSGALG00010029689	0.983224145	0.294185703
ENSGALG00010029690	0.9790953	0.324099489
ENSGALG00010029691	0.07097039	0.086666223
ENSGALG00010029692	0.553862524	0.460490662
ENSGALG00010029693	0.608527171	0.443603669
ENSGALG00010029694	0.295349069	0.091739094
ENSGALG00010029695	0.533017513	0.165157425
ENSGALG00010029696	0.7112329	0.028488445
ENSGALG00010029697	0.985131659	0.306614098
ENSGALG00010029698	0.957689124	0.344568747
ENSGALG00010029699	0.978338313	0.336481843
ENSGALG00010029700	0.962179114	0.379520956
ENSGALG00010029701	0.671908065	0.249084839
ENSGALG00010029702	0.983092795	0.338516457
ENSGALG00010029703	0.953286015	0.367457819
ENSGALG00010029704	0.504760521	0.23238702
ENSGALG00010029705	0.898562355	0.305571035
ENSGALG00010029706	0.950394857	0.22016055
ENSGALG00010029707	0.196599738	0.053299441
ENSGALG00010029708	0.613949329	0.10678424
ENSGALG00010029709	0.951990415	0.305695818
ENSGALG00010029710	0.955766059	0.222673216
ENSGALG00010029711	0.723258967	0.106970104
ENSGALG00010029712	0.2991341	0.047692132
ENSGALG00010029713	0.808008374	0.110013799
ENSGALG00010029715	0.755936788	0.265900391
ENSGALG00010029716	0.862375725	0.364114768
ENSGALG00010029717	0.564447972	0.174835608
ENSGALG00010029718	0.643756177	0.013090941
ENSGALG00010029719	0.660198961	0.210405758
ENSGALG00010029720	0.876717055	0.181644501
ENSGALG00010029721	0.791386456	0.192735235

ENSGALG00010029722	0.957110914	0.188538106
ENSGALG00010029723	0.066558328	0.0795024
ENSGALG00010029724	0.659032863	0.23793205
ENSGALG00010029725	0.850379544	0.188025413
ENSGALG00010029726	0.102946481	0.422305277
ENSGALG00010029727	0.884915543	0.231452051
ENSGALG00010029728	0.823329964	0.231283686
ENSGALG00010029730	0.939438847	0.177839851
ENSGALG00010029731	0.630587136	0.057365575
ENSGALG00010029732	0.570524881	0.282084471
ENSGALG00010029733	0.960839061	0.303574135
ENSGALG00010029734	0.912564651	0.248943793
ENSGALG00010029735	0.645160486	0.416899512
ENSGALG00010029736	0.635336226	0.198390919
ENSGALG00010029737	0.80546896	0.508744708
ENSGALG00010029738	0.791834541	0.55019744
ENSGALG00010029739	0.851466477	0.225160503
ENSGALG00010029740	0.481962646	0.321881709
ENSGALG00010029741	0.978772002	0.28491992
ENSGALG00010029742	0.902982461	0.184725063
ENSGALG00010029743	0.990447602	0.319475667
ENSGALG00010029744	0.127581942	0.112069311
ENSGALG00010029745	0.935113877	0.445646255
ENSGALG00010029746	0.178786633	0.157251584
ENSGALG00010029747	0.095074944	0.376673614
ENSGALG00010029748	0.991955861	0.345137803
ENSGALG00010029749	0.573029552	0.264574624
ENSGALG00010029750	0.908944496	0.347408226
ENSGALG00010029751	0.749925956	0.249238092
ENSGALG00010029752	0.162948555	0.108866622
ENSGALG00010029753	0.934153459	0.399166916
ENSGALG00010029754	0.81368558	0.380113799
ENSGALG00010029755	0.98879695	0.338230868
ENSGALG00010029756	0.304875474	0.144074166
ENSGALG00010029757	0.923256595	0.298429498
ENSGALG00010029758	0.084656592	0.141693424
ENSGALG00010029759	0.975329139	0.329063429
ENSGALG00010029761	0.799116051	0.329210711
ENSGALG00010029762	0.76117642	0.140620643
ENSGALG00010029763	0.51534521	0.048273123
ENSGALG00010029764	0.81486154	0.28467014
ENSGALG00010029765	0.339321283	0.243147379
ENSGALG00010029766	0.900371865	0.276194936
ENSGALG00010029767	0.922013614	0.338145274
ENSGALG00010029768	0.971851037	0.353715855
ENSGALG00010029769	0.068967245	0.000587985
ENSGALG00010029770	0.370843422	0.342049638
ENSGALG00010029771	0.664526599	0.192990407
ENSGALG00010029772	0.760693661	0.02481403
ENSGALG00010029773	0.913812966	0.391341523
ENSGALG00010029774	0.982804086	0.312708401
ENSGALG00010029775	0.892562942	0.167865458
ENSGALG00010029776	0.97032975	0.34957846
ENSGALG00010029777	0.899345163	0.153261652
ENSGALG00010029778	0.462614994	0.302062805
ENSGALG00010029780	0.406491347	0.346953861

ENSGALG00010029781	0.949796825	0.368258118
ENSGALG00010029782	0.660806056	0.17013627
ENSGALG00010029783	0.140888165	0.047638098
ENSGALG00010029784	0.975978899	0.311095856
ENSGALG00010029785	0.997479405	0.327130528
ENSGALG00010029786	0.998131827	0.331919977
ENSGALG00010029787	0.936374746	0.22467007
ENSGALG00010029788	0.785202706	0.233551649
ENSGALG00010029790	0.065158438	0.217455258
ENSGALG00010029791	0.920092929	0.196942673
ENSGALG00010029792	0.08849734	0.252962123
ENSGALG00010029793	0.238022712	0.095493128
ENSGALG00010029794	0.382985366	0.1291087
ENSGALG00010029795	0.383041837	0.148092965
ENSGALG00010029796	0.857610095	0.502617149
ENSGALG00010029797	0.818936978	0.271026599
ENSGALG00010029798	0.330589625	0.32753095
ENSGALG00010029801	0.714285371	0.009316713
ENSGALG00010029802	0.265558089	0.028821141
ENSGALG00010029804	0.418403859	0.137262211
ENSGALG00010029805	0.437101788	0.120409956
ENSGALG00010029806	0.701625928	0.313395722
ENSGALG00010029807	0.28050743	0.185921568
ENSGALG00010029808	0.426866692	0.538467599
ENSGALG00010029809	0.000957769	0.339665072
ENSGALG00010029810	0.804002542	0.076246751
ENSGALG00010029811	0.780542968	0.122255382
ENSGALG00010029812	0.025122514	0.626982856
ENSGALG00010029813	0.58139716	0.534111761
ENSGALG00010029814	0.799314364	0.249033611
ENSGALG00010029815	0.836051824	0.220289839
ENSGALG00010029816	0.664155278	0.266036686
ENSGALG00010029817	0.891454072	0.327708327
ENSGALG00010029818	0.936478481	0.326534776
ENSGALG00010029819	0.2430798	0.207668055
ENSGALG00010029820	0.387374433	0.109360451
ENSGALG00010029823	0.311997177	0.424590371
ENSGALG00010029827	0.298598643	0.256694648
ENSGALG00010029828	0.500406649	0.182616804
ENSGALG00010029834	0.242704765	0.126598106
ENSGALG00010029835	0.907340973	0.44214869
ENSGALG00010029836	0.739378944	0.011752061
ENSGALG00010029837	0.982429247	0.330425634
ENSGALG00010029839	0.349898801	0.27603925
ENSGALG00010029841	0.475537496	0.378349006
ENSGALG00010029844	0.581247037	0.200158436
ENSGALG00010029845	0.470810233	0.309523015
ENSGALG00010029847	0.586553086	0.37973285
ENSGALG00010029850	0.626542268	0.06388599
ENSGALG00010029854	0.277566769	0.107857127
ENSGALG00010029856	0.963807909	0.241124139
ENSGALG00010029857	0.906300766	0.333228107
ENSGALG00010029858	0.785725497	0.516767646
ENSGALG00010029859	0.83546448	0.295804857
ENSGALG00010029860	0.288784432	0.238149581
ENSGALG00010029861	0.826596895	0.251948412

ENSGALG00010029862	0.676118961	0.024427399
ENSGALG00010029863	0.692681928	0.026920455
ENSGALG00010029864	0.911316249	0.389108763
ENSGALG00010029865	0.212856342	0.113526745
ENSGALG00010029866	0.034122929	0.043157565
ENSGALG00010029867	0.075864242	0.032286247
ENSGALG00010029868	0.028716272	0.17724602
ENSGALG00010029869	0.633118691	0.162596501
ENSGALG00010029871	0.208550923	0.676301294
ENSGALG00010029873	0.271162407	0.118740615
ENSGALG00010029876	0.508627651	0.218138437
ENSGALG00010029878	0.604595787	0.268337389
ENSGALG00010029879	0.777330867	0.371494779
ENSGALG00010029880	0.010534108	0.108510093
ENSGALG00010029881	0.41596524	0.187579552
ENSGALG00010029882	0.219386918	0.079388121
ENSGALG00010029883	0.528736459	0.61536648
ENSGALG00010029884	0.72105419	0.416249342
ENSGALG00010029885	0.230386193	0.091602208
ENSGALG00010029886	0.181275072	0.251143049
ENSGALG00010029887	0.786213248	0.327410698
ENSGALG00010029888	0.249675719	0.319212827
ENSGALG00010029889	0.275125424	0.126154882
ENSGALG00010029892	0.476636619	0.28042731
ENSGALG00010029893	0.559440921	0.176551736
ENSGALG00010029894	0.377096693	0.134016806
ENSGALG00010029895	0.975242098	0.294908674
ENSGALG00010029897	0.206911318	0.115213763
ENSGALG00010029898	0.270451009	0.090541667
ENSGALG00010029899	0.382448095	0.094032633
ENSGALG00010029901	0.285272007	0.109169955
ENSGALG00010029903	0.833852274	0.187553682
ENSGALG00010029904	0.25153872	0.017020562
ENSGALG00010029907	0.905687956	0.266725071
ENSGALG00010029909	0.63529269	0.198965251
ENSGALG00010029914	0.390466756	0.106163828
ENSGALG00010029916	0.81081909	0.300721096
ENSGALG00010029920	0.266137114	0.107784983
ENSGALG00010029921	0.785102578	0.187893556
ENSGALG00010029923	0.459194924	0.139014987
ENSGALG00010029924	0.187555343	0.194535423
ENSGALG00010029925	0.104345636	0.078743762
ENSGALG00010029926	0.730431408	0.075227096
ENSGALG00010029927	0.054717168	0.37877297
ENSGALG00010029928	0.686766021	0.408688922
ENSGALG00010029929	0.093350038	0.109304197
ENSGALG00010029930	0.109168363	0.225682899
ENSGALG00010029932	0.239260536	0.186256372
ENSGALG00010029933	0.929350179	0.243010289
ENSGALG00010029934	0.088797653	0.357122107
ENSGALG00010029935	0.154958561	0.342899367
ENSGALG00010029936	0.992920323	0.305973123
ENSGALG00010029937	0.953807656	0.305115733
ENSGALG00010029938	0.983207766	0.281005046
ENSGALG00010029939	0.043367435	0.54588566
ENSGALG00010029941	0.816544072	0.288573728

ENSGALG00010029943	0.419258983	0.210904094
ENSGALG00010029944	0.144515293	0.311748346
ENSGALG00010029945	0.492239349	0.30102198
ENSGALG00010029946	0.821476518	0.432083936
ENSGALG00010029947	0.796672625	0.363427116
ENSGALG00010029948	0.987355338	0.354485627
ENSGALG00010029950	0.966089682	0.308533708
ENSGALG00010029951	0.944073113	0.367531113
ENSGALG00010029952	0.099921756	0.261213653
ENSGALG00010029953	0.790518263	0.410172502
ENSGALG00010029956	0.98483516	0.337671089
ENSGALG00010029957	0.985608097	0.293505707
ENSGALG00010029958	0.981814006	0.28174133
ENSGALG00010029959	0.981874325	0.330401523
ENSGALG00010029960	0.019712204	0.418846563
ENSGALG00010029962	0.430182133	0.052554495
ENSGALG00010029966	0.418583343	0.253436761
ENSGALG00010029967	0.39190877	0.131701825
ENSGALG00010029968	0.285272007	0.109169955
ENSGALG00010029978	0.375157217	0.295541278
ENSGALG00010029983	0.483999928	0.154434277
ENSGALG00010029984	0.888721302	0.31904481
ENSGALG00010029987	0.418023482	0.260249685
ENSGALG00010029988	0.643939703	0.305192965
ENSGALG00010029990	0.265842936	0.058628392
ENSGALG00010029991	0.538752987	0.320278859
ENSGALG00010029995	0.945645706	0.162554554
ENSGALG00010029997	0.94280447	0.311098534
ENSGALG00010030001	0.544232447	0.025189088
ENSGALG00010030002	0.56977859	0.067115863
ENSGALG00010030003	0.53813553	0.329636751
ENSGALG00010030004	0.949544811	0.393962156
ENSGALG00010030005	0.117150563	0.27518886
ENSGALG00010030006	0.398498509	0.49303674
ENSGALG00010030007	0.255492709	0.101879462
ENSGALG00010030008	0.908686375	0.33897134
ENSGALG00010030012	0.712184303	0.273427504
ENSGALG00010030013	0.303768277	0.18293581
ENSGALG00010030014	0.893464387	0.341746497
ENSGALG00010030016	0.981356994	0.293312669
ENSGALG00010030017	0.148948063	0.268126765
ENSGALG00010030019	0.339444113	0.045741665
ENSGALG00010030021	0.278434714	0.327665487
ENSGALG00010030026	0.121386485	0.19735015
ENSGALG00010030027	0.775227756	0.149829211
ENSGALG00010030029	0.045230036	0.275555838
ENSGALG00010030041	0.322415465	0.246520801
ENSGALG00010030042	0.662903744	0.415586347
ENSGALG00010030043	0.577744561	0.209142794
ENSGALG00010030044	0.353136475	0.236486821
ENSGALG00010030045	0.266137114	0.107784983
ENSGALG00010030046	0.102206405	0.249375097
ENSGALG00010030047	0.947420611	0.404980986
ENSGALG00010030048	0.640987691	0.138551271
ENSGALG00010030049	0.932167086	0.360781167
ENSGALG00010030052	0.816828571	0.390570912

ENSGALG00010030053	0.886967454	0.406331887
ENSGALG00010030062	0.277566769	0.107857127
ENSGALG00010030063	0.591182869	0.123954105
ENSGALG00010030064	0.379129433	0.232100788
ENSGALG00010030066	0.646258753	0.031483691
ENSGALG00010030068	0.523699346	0.09038894
ENSGALG00010030072	0.86924154	0.232717136
ENSGALG00010030073	0.845446017	0.203979876
ENSGALG00010030074	0.412432723	0.157005434
ENSGALG00010030075	0.032251128	0.066414782
ENSGALG00010030076	0.515522834	0.144283912
ENSGALG00010030080	0.434561311	0.038781454
ENSGALG00010030081	0.863818482	0.276755676
ENSGALG00010030082	0.910008608	0.318614762
ENSGALG00010030084	0.189609775	0.037416632
ENSGALG00010030085	0.089985046	0.429628186
ENSGALG00010030087	0.192559451	0.243801159
ENSGALG00010030088	0.38480368	0.180523942
ENSGALG00010030089	0.16810692	0.168037377
ENSGALG00010030091	0.396365709	0.157137627
ENSGALG00010030100	0.506004162	0.19477771
ENSGALG00010030101	0.95633373	0.368615735
ENSGALG00010030102	0.982482726	0.351681263
ENSGALG00010030103	0.093680825	0.121060027
ENSGALG00010030104	0.991791047	0.311065543
ENSGALG00010030105	0.443955729	0.160904907
ENSGALG00010030106	0.911658811	0.272358657
ENSGALG00010030107	0.79801444	0.269339316
ENSGALG00010030108	0.962719101	0.296716842
ENSGALG00010030109	0.514288074	0.029961593
