# Supplementary material for: Weighted gene co-expression network analysis identifies important modules and hub genes involved in the regulation of breast muscle yield in broilers
Source: Anim Biosci. 2024 Apr 25;37(10):1673–82. doi: 10.5713/ab.23.0548 (PMC11366510; doi:10.5713/ab.23.0548)
Supplement: Supplementary file 8 [file ab-23-0548-Supplementary-Table-8.pdf]

Table S8. Hub genes identified in the skyblue3 module.

Gene ID	gene name
ENSGALG00010028957	ENSGALG00010028957
ENSGALG00010005055	ENSGALG00010005055
ENSGALG00010007379	ENSGALG00010007379
ENSGALG00010019489	ENSGALG00010019489
ENSGALG00010028616	ENSGALG00010028616
ENSGALG00010028948	ENSGALG00010028948
ENSGALG00010011669	ENSGALG00010011669
ENSGALG00010028023	ENSGALG00010028023
ENSGALG00010016628	ENSGALG00010016628
ENSGALG00010021132	CALHM3
ENSGALG00010010930	FAM163A
ENSGALG00010007044	ENSGALG00010007044
ENSGALG00010020904	ENSGALG00010020904
ENSGALG00010011938	ENSGALG00010011938
ENSGALG00010020400	ENSGALG00010020400
ENSGALG00010028945	ENSGALG00010028945
ENSGALG00010015988	ENSGALG00010015988
ENSGALG00010028588	ABHD8
ENSGALG00010015294	ENSGALG00010015294
ENSGALG00010023295	ENSGALG00010023295
ENSGALG00010015293	ENSGALG00010015293
ENSGALG00010020427	ENSGALG00010020427
ENSGALG00010020897	VTG3
ENSGALG00010026116	DLX3
ENSGALG00010022763	ENSGALG00010022763
ENSGALG00010025036	ENSGALG00010025036
ENSGALG00010023037	ENSGALG00010023037
ENSGALG00010023325	ENSGALG00010023325
ENSGALG00010002375	NPY
ENSGALG00010026587	ENSGALG00010026587
ENSGALG00010009783	ENSGALG00010009783
ENSGALG00010022373	AK5
ENSGALG00010018155	ENSGALG00010018155
ENSGALG00010019415	ENSGALG00010019415
ENSGALG00010016621	GRXCR2
ENSGALG00010019992	CHAC2
ENSGALG00010029523	FSCN2
ENSGALG00010018083	PAX1
ENSGALG00010024214	CRABP1
ENSGALG00010023326	ENSGALG00010023326
ENSGALG00010016051	ENSGALG00010016051
ENSGALG00010001372	ENSGALG00010001372
ENSGALG00010005206	gga-mir-6601
ENSGALG00010016627	SH3RF2
ENSGALG00010022611	gga-mir-191
ENSGALG00010017352	GPCPD1
ENSGALG00010012459	ENSGALG00010012459
ENSGALG00010015506	ENSGALG00010015506
ENSGALG00010002531	ENSGALG00010002531
ENSGALG00010014307	ENSGALG00010014307
ENSGALG00010008479	ENSGALG00010008479
ENSGALG00010019144	IFT122
ENSGALG00010020430	ENSGALG00010020430

ENSGALG00010027385	CNTN2
ENSGALG00010023339	ENSGALG00010023339
ENSGALG00010024844	TM4SF19
ENSGALG00010029071	ENSGALG00010029071
ENSGALG00010028152	ENSGALG00010028152
ENSGALG00010024124	DIDO1
ENSGALG00010025238	IGSF9B
ENSGALG00010019299	ENSGALG00010019299
ENSGALG00010013111	RFESD
ENSGALG00010009819	ENSGALG00010009819
ENSGALG00010000811	ENSGALG00010000811
ENSGALG00010004285	SLC18B1
ENSGALG00010021986	ENSGALG00010021986
ENSGALG00010007274	ENSGALG00010007274
ENSGALG00010019838	ALX4
ENSGALG00010009471	ENSGALG00010009471
ENSGALG00010024380	PNMT
ENSGALG00010023333	ENDOU
ENSGALG00010002535	ENSGALG00010002535
ENSGALG00010003684	ENSGALG00010003684
ENSGALG00010007520	ENSGALG00010007520
ENSGALG00010008944	ENSGALG00010008944
ENSGALG00010011109	ENSGALG00010011109
ENSGALG00010011259	ENSGALG00010011259
ENSGALG00010014279	ENSGALG00010014279
ENSGALG00010015106	ENSGALG00010015106
ENSGALG00010016539	ENSGALG00010016539
ENSGALG00010019133	ENSGALG00010019133
ENSGALG00010019512	ENSGALG00010019512
ENSGALG00010022848	SGIP1
ENSGALG00010024015	ENSGALG00010024015
ENSGALG00010024087	ENSGALG00010024087
ENSGALG00010026363	FAM187A
ENSGALG00010026490	ENSGALG00010026490
ENSGALG00010027868	CACNA2D3
ENSGALG00010028940	ENSGALG00010028940
ENSGALG00010018176	ENSGALG00010018176
ENSGALG00010002640	ENSGALG00010002640
ENSGALG00010003163	ENSGALG00010003163
ENSGALG00010003266	ENSGALG00010003266
ENSGALG00010004320	ENSGALG00010004320
ENSGALG00010004813	ENSGALG00010004813
ENSGALG00010005066	ENSGALG00010005066
ENSGALG00010007804	ENSGALG00010007804
ENSGALG00010010040	ENSGALG00010010040
ENSGALG00010010176	ENSGALG00010010176
ENSGALG00010010227	OLIG2
ENSGALG00010010517	STMN4
ENSGALG00010011444	TMEM252
ENSGALG00010014123	ENSGALG00010014123
ENSGALG00010014815	ENSGALG00010014815
ENSGALG00010015178	ENSGALG00010015178
ENSGALG00010015183	SLC5A7
ENSGALG00010016327	IL4
ENSGALG00010018679	ENSGALG00010018679
ENSGALG00010018683	ENSGALG00010018683

ENSGALG00010020482	Metazoa_SRP
ENSGALG00010020488	gga-mir-1676
ENSGALG00010021128	CALHM1
ENSGALG00010021796	VENTX
ENSGALG00010023754	ENSGALG00010023754
ENSGALG00010025110	ENSGALG00010025110
ENSGALG00010026577	ENSGALG00010026577
ENSGALG00010027851	gga-mir-6654
ENSGALG00010029871	ENSGALG00010029871
ENSGALG00010019340	ENSGALG00010019340
ENSGALG00010010369	ENSGALG00010010369
ENSGALG00010001827	PDZRN4
ENSGALG00010009567	NSD1
ENSGALG00010022175	ENSGALG00010022175
ENSGALG00010026780	MYSM1
ENSGALG00010016284	ENSGALG00010016284
ENSGALG00010026117	LZTR1
ENSGALG00010004386	CFAP44
ENSGALG00010022271	SLC18A2
ENSGALG00010028654	ENSGALG00010028654
ENSGALG00010002569	GTPBP6
ENSGALG00010003918	HTR1E
ENSGALG00010013568	ENSGALG00010013568
ENSGALG00010024960	ARFRP1
ENSGALG00010020647	ENSGALG00010020647
ENSGALG00010005682	ENSGALG00010005682
ENSGALG00010012086	CIBAR1
ENSGALG00010012128	ENSGALG00010012128
ENSGALG00010013093	GPATCH3
ENSGALG00010006736	ENSGALG00010006736
ENSGALG00010008346	NR4A3
ENSGALG00010022851	ENSGALG00010022851
ENSGALG00010025012	CRH2
ENSGALG00010023174	KCNQ2
ENSGALG00010018929	ENSGALG00010018929
ENSGALG00010025447	ENSGALG00010025447
ENSGALG00010010667	ENSGALG00010010667
ENSGALG00010013952	ENSGALG00010013952
ENSGALG00010023246	SP5
ENSGALG00010024154	VPS29L
ENSGALG00010024492	NTF3
