# Supplementary material for: Weighted gene co-expression network analysis identifies important modules and hub genes involved in the regulation of breast muscle yield in broilers
Source: Anim Biosci. 2024 Apr 25;37(10):1673–82. doi: 10.5713/ab.23.0548 (PMC11366510; doi:10.5713/ab.23.0548)
Supplement: Supplementary file 9 [file ab-23-0548-Supplementary-Table-9.pdf]

Table S9. Functional enrichment analysis of the hub genes in the skyblue3 module.

Category	Term description	Term ID	Adjusted p value	Genes
GO:MF	monoamine:proton antiporter activity	GO:0015311	0.02079708	ENSGALG00010004285,ENSGALG00010022271
GO:MF	monoamine transmembrane transporter activity	GO:0008504	0.0480393	ENSGALG00010004285,ENSGALG00010022271
