# Supplementary material for: Weighted gene co-expression network analysis identifies important modules and hub genes involved in the regulation of breast muscle yield in broilers
Source: Anim Biosci. 2024 Apr 25;37(10):1673–82. doi: 10.5713/ab.23.0548 (PMC11366510; doi:10.5713/ab.23.0548)
Supplement: Supplementary file 10 [file ab-23-0548-Supplementary-Table-10.pdf]

Table S10. The hub genes identified in the turquoise module.

Gene ID	gene name
ENSGALG00010028598	TMOD4
ENSGALG00010029655	PHKG1
ENSGALG00010029215	ENSGALG00010029215
ENSGALG00010024570	TPM1
ENSGALG00010024181	FOXD3
ENSGALG00010019451	CAV3
ENSGALG00010016292	PHKA1
ENSGALG00010000153	NF2
ENSGALG00010002079	ARX
ENSGALG00010012253	MYF6
ENSGALG00010018340	DNPEP
ENSGALG00010029786	ENSGALG00010029786
ENSGALG00010017826	PABPC4
ENSGALG00010019045	LBX1
ENSGALG00010023392	TPI1
ENSGALG00010013012	TMEM182
ENSGALG00010023957	YBX3
ENSGALG00010022774	A1CF
ENSGALG00010021110	PKLR
ENSGALG00010013846	YIPF7
ENSGALG00010009385	GPI
ENSGALG00010006069	TTR
ENSGALG00010001844	HOXA10
ENSGALG00010029785	ENSGALG00010029785
ENSGALG00010004358	GC
ENSGALG00010017501	SPIA5
ENSGALG00010006653	CFL2
ENSGALG00010003750	CPB2
ENSGALG00010023605	INSYN1
ENSGALG00010000081	ALDOA
ENSGALG00010013965	SLC2A2
ENSGALG00010021670	BLCAP
ENSGALG00010009379	RYR1
ENSGALG00010019065	CAND2
ENSGALG00010023099	DNAJC22
ENSGALG00010007226	PLG
ENSGALG00010026635	C8A
ENSGALG00010017481	SPIA1
ENSGALG00010005199	ENSGALG00010005199
ENSGALG00010029213	APOH
ENSGALG00010016781	CDIP1
ENSGALG00010004990	ENSGALG00010004990
ENSGALG00010018306	DES
ENSGALG00010022038	GAPDH
ENSGALG00010024051	KRT8
ENSGALG00010006570	SULT6B1L
ENSGALG00010018322	NT5C1A
ENSGALG00010027351	TMEM38A
ENSGALG00010029336	SYNGR2
ENSGALG00010027409	ENSGALG00010027409
ENSGALG00010021228	ESRP2
ENSGALG00010001427	ASB11
ENSGALG00010011715	SLCO1B3

ENSGALG00010014778	TMPRSS6
ENSGALG00010016699	SYNPO
ENSGALG00010022972	A2M
ENSGALG00010004888	FOXA1
ENSGALG00010017512	SPIA9
ENSGALG00010027714	SYPL2
ENSGALG00010026700	C8B
ENSGALG00010024440	PKNOX2
ENSGALG00010008084	CYP2AC1
ENSGALG00010007977	APOB
ENSGALG00010014972	FGA
ENSGALG00010002953	WDFY2
ENSGALG00010008664	TM4SF1a
ENSGALG00010018732	FYTTD1L
ENSGALG00010028849	CCDC180
ENSGALG00010017466	SPIA4
ENSGALG00010017460	SERPINA10
ENSGALG00010001351	CAPN1
ENSGALG00010004338	ENSGALG00010004338
ENSGALG00010003206	ENSGALG00010003206
ENSGALG00010008168	CAVIN4
ENSGALG00010018783	ENSGALG00010018783
ENSGALG00010013767	PGK2
ENSGALG00010018076	FOXA2
ENSGALG00010000476	ENSGALG00010000476
ENSGALG00010014696	SPINK5
ENSGALG00010002582	C10orf90
ENSGALG00010016263	HGFAC
ENSGALG00010013994	CHRM2
ENSGALG00010027528	APOA2
ENSGALG00010005255	BZW2
ENSGALG00010020714	PGAM1
ENSGALG00010020327	MTTPL
ENSGALG00010005926	NUDT5
ENSGALG00010024018	KRT18
ENSGALG00010012264	MYF5
ENSGALG00010007957	CDH15
ENSGALG00010025499	STAC3
ENSGALG00010017656	FSD2
ENSGALG00010001514	MET
ENSGALG00010025996	CUL3
ENSGALG00010028082	AK1
ENSGALG00010005269	KIN
ENSGALG00010016400	PRPS1L1
ENSGALG00010009676	ACO1
ENSGALG00010019621	ENSGALG00010019621
ENSGALG00010016229	ENSGALG00010016229
ENSGALG00010015188	SULT1C3
ENSGALG00010020302	HAAO
ENSGALG00010000571	ASPDH
ENSGALG00010004921	RRAGD
ENSGALG00010012171	CDH17
ENSGALG00010017562	SLC30A10
ENSGALG00010021871	ENSGALG00010021871
ENSGALG00010004973	SLN
ENSGALG00010022987	F2

ENSGALG00010001971	MLIP
ENSGALG00010004995	PIT54
ENSGALG00010003811	PGLYRP2
ENSGALG00010006487	ENSGALG00010006487
ENSGALG00010013428	BPGM
ENSGALG00010014540	FTCD
ENSGALG00010005556	IYD
ENSGALG00010011455	AvBD8
ENSGALG00010010115	NEB
ENSGALG00010013029	MYLK2
ENSGALG00010028053	ORM1
ENSGALG00010024650	PRKAG1
ENSGALG00010020734	SIGIRR
ENSGALG00010021381	DNAJB2
ENSGALG00010006279	HACD1
ENSGALG00010013451	PRKAB2
ENSGALG00010023762	BBS5
ENSGALG00010016916	ACYP2
ENSGALG00010022236	SHF
ENSGALG00010016177	HRG
ENSGALG00010020730	RRP12
ENSGALG00010027059	ASB14
ENSGALG00010015075	MUL1L
ENSGALG00010022568	SCRN3
ENSGALG00010012730	CAP2
ENSGALG00010005264	ITIH2
ENSGALG00010024546	KY
ENSGALG00010017724	FLII
ENSGALG00010019764	ENSGALG00010019764
ENSGALG00010020070	ARL6IP5
ENSGALG00010025504	AMPD1
ENSGALG00010020142	SLC35E2B
ENSGALG00010017515	SPIA3
ENSGALG00010028479	CASQ2
ENSGALG00010000565	ENSGALG00010000565
ENSGALG00010027153	ACTR3B
ENSGALG00010021619	FHOD1
ENSGALG00010015583	PRDM4
ENSGALG00010027225	CREB3L3
ENSGALG00010013429	PDE4DIP
ENSGALG00010029628	UNC45B
