# Supplementary material for: Weighted gene co-expression network analysis identifies important modules and hub genes involved in the regulation of breast muscle yield in broilers
Source: Anim Biosci. 2024 Apr 25;37(10):1673–82. doi: 10.5713/ab.23.0548 (PMC11366510; doi:10.5713/ab.23.0548)
Supplement: Supplementary file 11 [file ab-23-0548-Supplementary-Table-11.pdf]

Table S11. Functional enrichment analysis of the top 150 hub genes in the turquoise module.

Category	Term description	Term ID	Adjusted p value	Genes
GO:MF	endopeptidase inhibitor activity	GO:0004866	9.55E-06	ENSGALG00010017501,ENSGALG00010017481,ENSGALG00010022038,ENSGALG00010022972,ENSGALG00010017512,ENSGALG00010017466,ENSGALG00010017460,ENSGALG00010016177,ENSGALG00010005264,ENSGALG00010017515
GO:MF	peptidase inhibitor activity	GO:0030414	9.55E-06	ENSGALG00010017501,ENSGALG00010017481,ENSGALG00010022038,ENSGALG00010022972,ENSGALG00010017512,ENSGALG00010017466,ENSGALG00010017460,ENSGALG00010016177,ENSGALG00010005264,ENSGALG00010017515
GO:MF	endopeptidase regulator activity	GO:0061135	9.55E-06	ENSGALG00010017501,ENSGALG00010017481,ENSGALG00010022038,ENSGALG00010022972,ENSGALG00010017512,ENSGALG00010017466,ENSGALG00010017460,ENSGALG00010016177,ENSGALG00010005264,ENSGALG00010017515
GO:MF	peptidase regulator activity	GO:0061134	3.49E-05	ENSGALG00010017501,ENSGALG00010017481,ENSGALG00010022038,ENSGALG00010022972,ENSGALG00010017512,ENSGALG00010017466,ENSGALG00010017460,ENSGALG00010016177,ENSGALG00010005264,ENSGALG00010017515
GO:MF	serine-type endopeptidase inhibitor activity	GO:0004867	4.67E-05	ENSGALG00010017501,ENSGALG00010017481,ENSGALG00010017512,ENSGALG00010017466,ENSGALG00010017460,ENSGALG00010005264,ENSGALG00010017515
GO:MF	enzyme inhibitor activity	GO:0004857	0.002536	ENSGALG00010017501,ENSGALG00010017481,ENSGALG00010022038,ENSGALG00010022972,ENSGALG00010017512,ENSGALG00010017466,ENSGALG00010017460,ENSGALG00010016177,ENSGALG00010005264,ENSGALG00010017515

GO:MF	phosphoglycerate mutase activity	GO:0004619	0.004217	ENSGALG00010020714,ENSGALG00010013428
GO:MF	actin binding	GO:0003779	0.007441	ENSGALG00010028598,ENSGALG00010024570,ENSGALG00010000153,ENSGALG00010004358,ENSGALG00010006653,ENSGALG00010016699,ENSGALG00010024440,ENSGALG00010010115,ENSGALG00010012730,ENSGALG00010017724
GO:MF	intramolecular oxidoreductase activity, interconverting aldoses and ketoses	GO:0016861	0.017502	ENSGALG00010023392,ENSGALG00010009385
GO:MF	fructose-bisphosphate aldolase activity	GO:0004332	0.017502	ENSGALG00010000081,ENSGALG00010000476
GO:BP	glycolytic process	GO:0006096	3.60E-06	ENSGALG00010023392,ENSGALG00010021110,ENSGALG00010009385,ENSGALG00010000081,ENSGALG00010022038,ENSGALG00010013767,ENSGALG00010000476,ENSGALG00010020714,ENSGALG00010013428
GO:BP	carbohydrate catabolic process	GO:0016052	5.59E-06	ENSGALG00010023392,ENSGALG00010021110,ENSGALG00010009385,ENSGALG00010000081,ENSGALG00010022038,ENSGALG00010013767,ENSGALG00010000476,ENSGALG00010020714,ENSGALG00010005926,ENSGALG00010013428
GO:BP	pyruvate metabolic process	GO:0006090	1.41E-05	ENSGALG00010023392,ENSGALG00010021110,ENSGALG00010009385,ENSGALG00010000081,ENSGALG00010022038,ENSGALG00010013767,ENSGALG00010000476,ENSGALG00010020714,ENSGALG00010013428

GO:BP	muscle cell development	GO:0055001	1.85E-05	ENSGALG00010024570,ENSGALG00010019451,ENSGALG00010012253,ENSGALG00010013012,ENSGALG00010006653,ENSGALG00010027714,ENSGALG00010012264,ENSGALG00010025499,ENSGALG00010023762,ENSGALG00010028479
GO:BP	small molecule metabolic process	GO:0044281	0.000705	ENSGALG00010023392,ENSGALG00010021110,ENSGALG00010009385,ENSGALG00010006069,ENSGALG00010004358,ENSGALG00010000081,ENSGALG00010022038,ENSGALG00010018322,ENSGALG00010008084,ENSGALG00010007977,ENSGALG00010013767,ENSGALG00010000476,ENSGALG00010020714,ENSGALG00010005926,ENSGALG00010028082,ENSGALG00010016400,ENSGALG00010009676,ENSGALG00010015188,ENSGALG00010020302,ENSGALG00010000571,ENSGALG00010013428,ENSGALG00010005556,ENSGALG00010024650,ENSGALG00010006279,ENSGALG00010013451,ENSGALG00010025504
GO:BP	actomyosin structure organization	GO:0031032	0.000705	ENSGALG00010024570,ENSGALG00010019451,ENSGALG00010000153,ENSGALG00010006653,ENSGALG00010001514,ENSGALG00010025996,ENSGALG00010023762,ENSGALG00010028479,ENSGALG00010021619
GO:BP	sarcomere organization	GO:0045214	0.001206	ENSGALG00010024570,ENSGALG00010019451,ENSGALG00010006653,ENSGALG00010023762,ENSGALG00010028479
GO:BP	organic acid metabolic process	GO:0006082	0.00174	ENSGALG00010023392,ENSGALG00010021110,ENSGALG00010009385,ENSGALG00010000081,ENSGALG00010022038,ENSGALG00010008084,ENSGALG00010013767,ENSGALG00010000476,ENSGALG00010020714,ENSGALG00010009676,ENSGALG00010015188,ENSGALG00010020302,ENSGALG00010013428,ENSGALG00010005556,ENSGALG00010024650,ENSGALG00010006279,ENSGALG00010013451

GO:BP	blood coagulation	GO:0007596	0.001814	ENSGALG00010003750,ENSGALG00010007226,ENSGALG00010029213,ENSGALG00010014972,ENSGALG00010017460,ENSGALG00010018076,ENSGALG00010016263,ENSGALG00010022987
GO:BP	myotube cell development	GO:0014904	0.001814	ENSGALG00010012253,ENSGALG00010013012,ENSGALG00010012264,ENSGALG00010025499,ENSGALG00010023762
GO:BP	hemostasis	GO:0007599	0.001823	ENSGALG00010003750,ENSGALG00010007226,ENSGALG00010029213,ENSGALG00010014972,ENSGALG00010017460,ENSGALG00010018076,ENSGALG00010016263,ENSGALG00010022987
GO:BP	coagulation	GO:0050817	0.001823	ENSGALG00010003750,ENSGALG00010007226,ENSGALG00010029213,ENSGALG00010014972,ENSGALG00010017460,ENSGALG00010018076,ENSGALG00010016263,ENSGALG00010022987
GO:BP	striated muscle cell differentiation	GO:0051146	0.001852	ENSGALG00010024570,ENSGALG00010019451,ENSGALG00010012253,ENSGALG00010013012,ENSGALG00010006653,ENSGALG00010012264,ENSGALG00010025499,ENSGALG00010023762,ENSGALG00010028479
GO:BP	myofibril assembly	GO:0030239	0.00327	ENSGALG00010024570,ENSGALG00010019451,ENSGALG00010006653,ENSGALG00010023762,ENSGALG00010028479
GO:BP	muscle cell differentiation	GO:0042692	0.003292	ENSGALG00010024570,ENSGALG00010019451,ENSGALG00010012253,ENSGALG00010013012,ENSGALG00010006653,ENSGALG00010027714,ENSGALG00010012264,ENSGALG00010025499,ENSGALG00010023762,ENSGALG00010028479

GO:BP	monocarboxylic acid metabolic process	GO:0032787	0.003292	ENSGALG00010023392,ENSGALG00010021110,ENSGALG00010009385,ENSGALG00010000081,ENSGALG00010022038,ENSGALG00010013767,ENSGALG00010000476,ENSGALG00010020714,ENSGALG00010013428,ENSGALG00010024650,ENSGALG00010006279,ENSGALG00010013451
GO:BP	oxoacid metabolic process	GO:0043436	0.003292	ENSGALG00010023392,ENSGALG00010021110,ENSGALG00010009385,ENSGALG00010000081,ENSGALG00010022038,ENSGALG00010013767,ENSGALG00010000476,ENSGALG00010020714,ENSGALG00010009676,ENSGALG00010015188,ENSGALG00010020302,ENSGALG00010013428,ENSGALG00010005556,ENSGALG00010024650,ENSGALG00010006279,ENSGALG00010013451
GO:BP	muscle structure development	GO:0061061	0.003292	ENSGALG00010024570,ENSGALG00010019451,ENSGALG00010012253,ENSGALG00010013012,ENSGALG00010006653,ENSGALG00010027714,ENSGALG00010012264,ENSGALG00010025499,ENSGALG00010013029,ENSGALG00010023762,ENSGALG00010024546,ENSGALG00010028479,ENSGALG00010029628
GO:BP	striated muscle cell development	GO:0055002	0.003292	ENSGALG00010024570,ENSGALG00010019451,ENSGALG00010006653,ENSGALG00010023762,ENSGALG00010028479
GO:BP	myotube differentiation	GO:0014902	0.004148	ENSGALG00010019451,ENSGALG00010012253,ENSGALG00010013012,ENSGALG00010012264,ENSGALG00010025499,ENSGALG00010023762
GO:BP	fibrinolysis	GO:0042730	0.005309	ENSGALG00010003750,ENSGALG00010007226,ENSGALG00010029213

GO:BP	muscle organ development	GO:0007517	0.005451	ENSGALG00010024570,ENSGALG00010012253,ENSGALG00010006653,ENSGALG00010012264,ENSGALG00010025499,ENSGALG00010013029,ENSGALG00010023762,ENSGALG00010024546,ENSGALG00010029628
GO:BP	carbohydrate metabolic process	GO:0005975	0.007004	ENSGALG00010029655,ENSGALG00010016292,ENSGALG00010023392,ENSGALG00010021110,ENSGALG00010009385,ENSGALG00010000081,ENSGALG00010022038,ENSGALG00010013767,ENSGALG00010000476,ENSGALG00010020714,ENSGALG00010005926,ENSGALG00010013428
GO:BP	carboxylic acid metabolic process	GO:0019752	0.007117	ENSGALG00010023392,ENSGALG00010021110,ENSGALG00010009385,ENSGALG00010000081,ENSGALG00010022038,ENSGALG00010013767,ENSGALG00010000476,ENSGALG00010020714,ENSGALG00010009676,ENSGALG00010020302,ENSGALG00010013428,ENSGALG00010005556,ENSGALG00010024650,ENSGALG00010006279,ENSGALG00010013451
GO:BP	skeletal muscle fiber development	GO:0048741	0.007117	ENSGALG00010012253,ENSGALG00010012264,ENSGALG00010025499,ENSGALG00010023762
GO:BP	actin filament-based process	GO:0030029	0.009573	ENSGALG00010028598,ENSGALG00010024570,ENSGALG00010019451,ENSGALG00010000153,ENSGALG00010006653,ENSGALG00010016699,ENSGALG00010001514,ENSGALG00010025996,ENSGALG00010013029,ENSGALG00010023762,ENSGALG00010012730,ENSGALG00010017724,ENSGALG00010028479,ENSGALG00010021619

GO:BP	actin cytoskeleton organization	GO:0030036	0.010374	ENSGALG00010028598,ENSGALG00010024570,ENSGALG00010019451,ENSGALG00010000153,ENSGALG00010006653,ENSGALG00010016699,ENSGALG00010001514,ENSGALG00010025996,ENSGALG00010023762,ENSGALG00010012730,ENSGALG00010017724,ENSGALG00010028479,ENSGALG00010021619
GO:BP	wound healing	GO:0042060	0.010374	ENSGALG00010019451,ENSGALG00010003750,ENSGALG00010007226,ENSGALG00010029213,ENSGALG00010014972,ENSGALG00010017460,ENSGALG00010018076,ENSGALG00010016263,ENSGALG00010022987
GO:BP	regulation of body fluid levels	GO:0050878	0.012533	ENSGALG00010003750,ENSGALG00010007226,ENSGALG00010029213,ENSGALG00010014972,ENSGALG00010017460,ENSGALG00010018076,ENSGALG00010016263,ENSGALG00010022987
GO:BP	striated muscle contraction	GO:0006941	0.013053	ENSGALG00010024570,ENSGALG00010019451,ENSGALG00010027351,ENSGALG00010025499,ENSGALG00010013029,ENSGALG00010028479
GO:BP	regulation of myoblast differentiation	GO:0045661	0.025211	ENSGALG00010012253,ENSGALG00010013012,ENSGALG00010012264,ENSGALG00010023762
GO:BP	cellular component assembly involved in morphogenesis	GO:0010927	0.025211	ENSGALG00010024570,ENSGALG00010019451,ENSGALG00010006653,ENSGALG00010023762,ENSGALG00010028479

GO:BP	regulation of blood coagulation	GO:0030193	0.025211	ENSGALG00010003750,ENSGALG00010007226,ENSGALG00010029213,ENSGALG00010018076
GO:BP	skeletal muscle contraction	GO:0003009	0.025211	ENSGALG00010019451,ENSGALG00010025499,ENSGALG00010028479
GO:BP	regulation of hemostasis	GO:1900046	0.026673	ENSGALG00010003750,ENSGALG00010007226,ENSGALG00010029213,ENSGALG00010018076
GO:BP	skeletal muscle tissue development	GO:0007519	0.029153	ENSGALG00010012253,ENSGALG00010006653,ENSGALG00010012264,ENSGALG00010025499,ENSGALG00010013029,ENSGALG00010023762
GO:BP	regulation of coagulation	GO:0050818	0.030334	ENSGALG00010003750,ENSGALG00010007226,ENSGALG00010029213,ENSGALG00010018076
GO:BP	ribonucleoside monophosphate metabolic process	GO:0009161	0.03227	ENSGALG00010018322,ENSGALG00010028082,ENSGALG00010016400,ENSGALG00010025504
GO:BP	muscle contraction	GO:0006936	0.032596	ENSGALG00010024570,ENSGALG00010019451,ENSGALG00010027351,ENSGALG00010013994,ENSGALG00010025499,ENSGALG00010013029,ENSGALG00010028479

GO:BP	monosaccharide catabolic process	GO:0046365	0.032795	ENSGALG00010023392,ENSGALG00010021110,ENSGALG00010005926
GO:BP	supramolecular fiber organization	GO:0097435	0.032795	ENSGALG00010028598,ENSGALG00010024570,ENSGALG00010019451,ENSGALG00010000153,ENSGALG00010006653,ENSGALG00010018306,ENSGALG00010016699,ENSGALG00010001514,ENSGALG00010025996,ENSGALG00010023762,ENSGALG00010028479,ENSGALG00010021619
GO:BP	skeletal muscle organ development	GO:0060538	0.032795	ENSGALG00010012253,ENSGALG00010006653,ENSGALG00010012264,ENSGALG00010025499,ENSGALG00010013029,ENSGALG00010023762
GO:BP	nucleobase-containing small molecule metabolic process	GO:0055086	0.032795	ENSGALG00010006069,ENSGALG00010018322,ENSGALG00010005926,ENSGALG00010028082,ENSGALG00010016400,ENSGALG00010015188,ENSGALG00010020302,ENSGALG00010000571,ENSGALG00010013428,ENSGALG00010025504
GO:BP	response to wounding	GO:0009611	0.033153	ENSGALG00010019451,ENSGALG00010003750,ENSGALG00010007226,ENSGALG00010029213,ENSGALG00010014972,ENSGALG00010017460,ENSGALG00010018076,ENSGALG00010016263,ENSGALG00010022987
GO:BP	response to caffeine	GO:0031000	0.033591	ENSGALG00010009379,ENSGALG00010027351

GO:BP	cellular response to caffeine	GO:0071313	0.033591	ENSGALG00010009379,ENSGALG00010027351
GO:BP	gluconeogenesis	GO:0006094	0.033591	ENSGALG00010023392,ENSGALG00010009385,ENSGALG00010022038,ENSGALG00010020714
GO:BP	regulation of muscle contraction	GO:0006937	0.035345	ENSGALG00010019451,ENSGALG00010027351,ENSGALG00010013994,ENSGALG00010013029,ENSGALG00010028479
GO:BP	hexose biosynthetic process	GO:0019319	0.037759	ENSGALG00010023392,ENSGALG00010009385,ENSGALG00010022038,ENSGALG00010020714
GO:BP	muscle tissue morphogenesis	GO:0060415	0.03995	ENSGALG00010024570,ENSGALG00010012253,ENSGALG00010012264,ENSGALG00010013029
GO:BP	nucleoside phosphate metabolic process	GO:0006753	0.045037	ENSGALG00010018322,ENSGALG00010005926,ENSGALG00010028082,ENSGALG00010016400,ENSGALG00010015188,ENSGALG00010020302,ENSGALG00010000571,ENSGALG00010013428,ENSGALG00010025504
GO:BP	positive regulation of skeletal muscle fiber development	GO:0048743	0.045037	ENSGALG00010012253,ENSGALG00010012264

GO:CC	extracellular space	GO:0005615	0.004333	ENSGALG00010009385,ENSGALG00010006069,ENSGALG00010004358,ENSGALG00010017501,ENSGALG00010007226,ENSGALG00010017481,ENSGALG00010029213,ENSGALG00010011715,ENSGALG00010014778,ENSGALG00010022972,ENSGALG00010017512,ENSGALG00010007977,ENSGALG00010014972,ENSGALG00010017466,ENSGALG00010017460,ENSGALG00010028053,ENSGALG00010017515
GO:CC	contractile fiber	GO:0043292	0.004333	ENSGALG00010028598,ENSGALG00010006653,ENSGALG00010009379,ENSGALG00010018306,ENSGALG00010010115,ENSGALG00010023762,ENSGALG00010029628
GO:CC	extracellular region	GO:0005576	0.004333	ENSGALG00010009385,ENSGALG00010006069,ENSGALG00010004358,ENSGALG00010017501,ENSGALG00010007226,ENSGALG00010017481,ENSGALG00010029213,ENSGALG00010011715,ENSGALG00010014778,ENSGALG00010022972,ENSGALG00010017512,ENSGALG00010007977,ENSGALG00010014972,ENSGALG00010017466,ENSGALG00010017460,ENSGALG00010016263,ENSGALG00010027528,ENSGALG00010022987,ENSGALG00010011455,ENSGALG00010028053,ENSGALG00010005264,ENSGALG00010017515
GO:CC	sarcomere	GO:0030017	0.004333	ENSGALG00010028598,ENSGALG00010006653,ENSGALG00010009379,ENSGALG00010018306,ENSGALG00010010115,ENSGALG00010023762,ENSGALG00010029628
GO:CC	myofibril	GO:0030016	0.004333	ENSGALG00010028598,ENSGALG00010006653,ENSGALG00010009379,ENSGALG00010018306,ENSGALG00010010115,ENSGALG00010023762,ENSGALG00010029628

GO:CC	sarcoplasmic reticulum	GO:0016529	0.004333	ENSGALG00010009379,ENSGALG00010027351,ENSGALG00010023762,ENSGALG00010028479
GO:CC	pore complex	GO:0046930	0.012445	ENSGALG00010026635,ENSGALG00010026700,ENSGALG00010004338
GO:CC	I band	GO:0031674	0.012445	ENSGALG00010006653,ENSGALG00010009379,ENSGALG00010018306,ENSGALG00010010115,ENSGALG00010029628
GO:CC	sarcoplasm	GO:0016528	0.012445	ENSGALG00010009379,ENSGALG00010027351,ENSGALG00010023762,ENSGALG00010028479
GO:CC	membrane attack complex	GO:0005579	0.019091	ENSGALG00010026635,ENSGALG00010026700
GO:CC	chylomicron	GO:0042627	0.036008	ENSGALG00010029213,ENSGALG00010007977
GO:CC	Z disc	GO:0030018	0.040642	ENSGALG00010006653,ENSGALG00010018306,ENSGALG00010010115,ENSGALG00010029628

GO:CC	triglyceride-rich plasma lipoprotein particle	GO:0034385	0.047918	ENSGALG00010029213,ENSGALG00010007977
GO:CC	very-low-density lipoprotein particle	GO:0034361	0.047918	ENSGALG00010029213,ENSGALG00010007977
KEGG	Glycolysis / Gluconeogenesis	KEGG:00010	1.57E-05	ENSGALG00010023392,ENSGALG00010021110,ENSGALG00010009385,ENSGALG00010022038,ENSGALG00010013767,ENSGALG00010020714,ENSGALG00010013428
KEGG	Biosynthesis of amino acids	KEGG:01230	2.28E-05	ENSGALG00010023392,ENSGALG00010021110,ENSGALG00010022038,ENSGALG00010013767,ENSGALG00010020714,ENSGALG00010016400,ENSGALG00010009676
KEGG	Carbon metabolism	KEGG:01200	5.10E-05	ENSGALG00010023392,ENSGALG00010021110,ENSGALG00010009385,ENSGALG00010022038,ENSGALG00010013767,ENSGALG00010020714,ENSGALG00010016400,ENSGALG00010009676
KEGG	Tight junction	KEGG:04530	0.028562	ENSGALG00010000153,ENSGALG00010023957,ENSGALG00010016699,ENSGALG00010024650,ENSGALG00010013451,ENSGALG00010027153
KEGG	Regulation of actin cytoskeleton	KEGG:04810	0.028562	ENSGALG00010006653,ENSGALG00010026635,ENSGALG00010026700,ENSGALG00010013994,ENSGALG00010022987,ENSGALG00010013029,ENSGALG00010027153

KEGG	Purine metabolism	KEGG:00230	0.049899	ENSGALG00010018322,ENSGALG00010005926,ENSGALG00010028082,ENSGALG00010016400,ENSGALG00010025504
