# Supplementary material for: Weighted gene co-expression network analysis identifies important modules and hub genes involved in the regulation of breast muscle yield in broilers
Source: Anim Biosci. 2024 Apr 25;37(10):1673–82. doi: 10.5713/ab.23.0548 (PMC11366510; doi:10.5713/ab.23.0548)
Supplement: Supplementary file 12 [file ab-23-0548-Supplementary-Table-12.pdf]

Table S12. Top 30 hub genes identified in the turquoise module.

Gene ID	gene name
ENSGALG00010028598	TMOD4
ENSGALG00010029655	PHKG1
ENSGALG00010029215	ENSGALG00010029215
ENSGALG00010024570	TPM1
ENSGALG00010024181	FOXD3
ENSGALG00010019451	CAV3
ENSGALG00010016292	PHKA1
ENSGALG00010000153	NF2
ENSGALG00010002079	ARX
ENSGALG00010012253	MYF6
ENSGALG00010018340	DNPEP
ENSGALG00010029786	ENSGALG00010029786
ENSGALG00010017826	PABPC4
ENSGALG00010019045	LBX1
ENSGALG00010023392	TPI1
ENSGALG00010013012	TMEM182
ENSGALG00010023957	YBX3
ENSGALG00010022774	A1CF
ENSGALG00010021110	PKLR
ENSGALG00010013846	YIPF7
ENSGALG00010009385	GPI
ENSGALG00010006069	TTR
ENSGALG00010001844	HOXA10
ENSGALG00010029785	ENSGALG00010029785
ENSGALG00010004358	GC
ENSGALG00010017501	SPIA5
ENSGALG00010006653	CFL2
ENSGALG00010003750	CPB2
ENSGALG00010023605	INSYN1
ENSGALG00010000081	ALDOA
