# Supplementary material for: Weighted gene co-expression network analysis identifies important modules and hub genes involved in the regulation of breast muscle yield in broilers
Source: Anim Biosci. 2024 Apr 25;37(10):1673–82. doi: 10.5713/ab.23.0548 (PMC11366510; doi:10.5713/ab.23.0548)
Supplement: Supplementary file 13 [file ab-23-0548-Supplementary-Table-13.pdf]

Table S13. Functional enrichment analysis of the top 30 hub genes in the turquoise module.

Category	Term description	Term ID	Adjusted p value	Genes
GO:BP	purine nucleoside diphosphate metabolic process	GO:0009135	5.9E-05	ENSGALG00010023392,ENSGALG00010021110,ENSGALG00010009385,ENSGALG00010000081
GO:BP	glycolytic process	GO:0006096	5.9E-05	ENSGALG00010023392,ENSGALG00010021110,ENSGALG00010009385,ENSGALG00010000081
GO:BP	ADP metabolic process	GO:0046031	5.9E-05	ENSGALG00010023392,ENSGALG00010021110,ENSGALG00010009385,ENSGALG00010000081
GO:BP	nucleoside diphosphate phosphorylation	GO:0006165	5.9E-05	ENSGALG00010023392,ENSGALG00010021110,ENSGALG00010009385,ENSGALG00010000081
GO:BP	nucleotide phosphorylation	GO:0046939	5.9E-05	ENSGALG00010023392,ENSGALG00010021110,ENSGALG00010009385,ENSGALG00010000081
GO:BP	ATP generation from ADP	GO:0006757	5.9E-05	ENSGALG00010023392,ENSGALG00010021110,ENSGALG00010009385,ENSGALG00010000081
GO:BP	purine ribonucleoside diphosphate metabolic process	GO:0009179	5.9E-05	ENSGALG00010023392,ENSGALG00010021110,ENSGALG00010009385,ENSGALG00010000081
GO:BP	ribonucleoside diphosphate metabolic process	GO:0009185	5.9E-05	ENSGALG00010023392,ENSGALG00010021110,ENSGALG00010009385,ENSGALG00010000081
GO:BP	striated muscle cell differentiation	GO:0051146	5.9E-05	ENSGALG00010024570,ENSGALG00010019451,ENSGALG00010012253,ENSGALG00010013012,ENSGALG00010006653
GO:BP	muscle cell development	GO:0055001	5.9E-05	ENSGALG00010024570,ENSGALG00010019451,ENSGALG00010012253,ENSGALG00010013012,ENSGALG00010006653
GO:BP	generation of precursor metabolites and energy	GO:0006091	5.9E-05	ENSGALG00010029655,ENSGALG00010016292,ENSGALG00010023392,ENSGALG00010021110,ENSGALG00010009385,ENSGALG00010000081

GO:BP	pyruvate metabolic process	GO:0006090	6.42E-05	ENSGALG00010023392,ENSGALG00010021110,ENSGALG00010009385,ENSGALG00010000081
GO:BP	nucleoside diphosphate metabolic process	GO:0009132	0.000113	ENSGALG00010023392,ENSGALG00010021110,ENSGALG00010009385,ENSGALG00010000081
GO:BP	carbohydrate metabolic process	GO:0005975	0.000113	ENSGALG00010029655,ENSGALG00010016292,ENSGALG00010023392,ENSGALG00010021110,ENSGALG00010009385,ENSGALG00010000081
GO:BP	carbohydrate catabolic process	GO:0016052	0.000121	ENSGALG00010023392,ENSGALG00010021110,ENSGALG00010009385,ENSGALG00010000081
GO:BP	muscle cell differentiation	GO:0042692	0.000141	ENSGALG00010024570,ENSGALG00010019451,ENSGALG00010012253,ENSGALG00010013012,ENSGALG00010006653
GO:BP	anatomical structure morphogenesis	GO:0009653	0.000141	ENSGALG00010024570,ENSGALG00010019451,ENSGALG00010000153,ENSGALG00010002079,ENSGALG00010012253,ENSGALG00010019045,ENSGALG00010013012,ENSGALG00010021110,ENSGALG00010001844,ENSGALG00010006653
GO:BP	sarcomere organization	GO:0045214	0.000157	ENSGALG00010024570,ENSGALG00010019451,ENSGALG00010006653
GO:BP	ATP metabolic process	GO:0046034	0.000177	ENSGALG00010023392,ENSGALG00010021110,ENSGALG00010009385,ENSGALG00010000081
GO:BP	anatomical structure formation involved in morphogenesis	GO:0048646	0.000225	ENSGALG00010024570,ENSGALG00010019451,ENSGALG00010000153,ENSGALG00010012253,ENSGALG00010013012,ENSGALG00010021110,ENSGALG00010006653
GO:BP	anatomical structure development	GO:0048856	0.000284	ENSGALG00010024570,ENSGALG00010019451,ENSGALG00010000153,ENSGALG00010002079,ENSGALG00010012253,ENSGALG00010017826,ENSGALG00010019045,ENSGALG00010013012,ENSGALG00010022774,ENSGALG00010021110,ENSGALG00010006069,ENSGALG00010001844,ENSGALG00010006653

GO:BP	purine ribonucleoside triphosphate metabolic process	GO:0009205	0.000289	ENSGALG00010023392,ENSGALG00010021110,ENSGALG00010009385,ENSGALG00010000081
GO:BP	actomyosin structure organization	GO:0031032	0.000295	ENSGALG00010024570,ENSGALG00010019451,ENSGALG00010000153,ENSGALG00010006653
GO:BP	myofibril assembly	GO:0030239	0.0003	ENSGALG00010024570,ENSGALG00010019451,ENSGALG00010006653
GO:BP	ribonucleoside triphosphate metabolic process	GO:0009199	0.0003	ENSGALG00010023392,ENSGALG00010021110,ENSGALG00010009385,ENSGALG00010000081
GO:BP	purine nucleoside triphosphate metabolic process	GO:0009144	0.0003	ENSGALG00010023392,ENSGALG00010021110,ENSGALG00010009385,ENSGALG00010000081
GO:BP	striated muscle cell development	GO:0055002	0.000313	ENSGALG00010024570,ENSGALG00010019451,ENSGALG00010006653
GO:BP	purine-containing compound metabolic process	GO:0072521	0.000387	ENSGALG00010023392,ENSGALG00010021110,ENSGALG00010009385,ENSGALG00010006069,ENSGALG00010000081
GO:BP	nucleoside triphosphate metabolic process	GO:0009141	0.000403	ENSGALG00010023392,ENSGALG00010021110,ENSGALG00010009385,ENSGALG00010000081
GO:BP	developmental process	GO:0032502	0.000403	ENSGALG00010024570,ENSGALG00010019451,ENSGALG00010000153,ENSGALG00010002079,ENSGALG00010012253,ENSGALG00010017826,ENSGALG00010019045,ENSGALG00010013012,ENSGALG00010022774,ENSGALG00010021110,ENSGALG00010006069,ENSGALG00010001844,ENSGALG00010006653
GO:BP	cell development	GO:0048468	0.000426	ENSGALG00010024570,ENSGALG00010019451,ENSGALG00010000153,ENSGALG00010002079,ENSGALG00010012253,ENSGALG00010017826,ENSGALG00010013012,ENSGALG00010006069,ENSGALG00010006653

GO:BP	primary metabolic process	GO:0044238	0.000451	ENSGALG00010029655,ENSGALG00010019451,ENSGALG00010016292,ENSGALG00010000153,ENSGALG00010002079,ENSGALG00010012253,ENSGALG00010018340,ENSGALG00010017826,ENSGALG00010019045,ENSGALG00010023392,ENSGALG00010022774,ENSGALG00010021110,ENSGALG00010009385,ENSGALG00010006069,ENSGALG00010001844,ENSGALG00010004358,ENSGALG00010003750,ENSGALG00010000081
GO:BP	biological_process	GO:0008150	0.000476	ENSGALG00010028598,ENSGALG00010029655,ENSGALG00010024570,ENSGALG00010019451,ENSGALG00010016292,ENSGALG00010000153,ENSGALG00010002079,ENSGALG00010012253,ENSGALG00010018340,ENSGALG00010017826,ENSGALG00010019045,ENSGALG00010023392,ENSGALG00010013012,ENSGALG00010022774,ENSGALG00010021110,ENSGALG00010013846,ENSGALG00010009385,ENSGALG00010006069,ENSGALG00010001844,ENSGALG00010004358,ENSGALG00010006653,ENSGALG00010003750,ENSGALG00010023605,ENSGALG00010000081
GO:BP	NADH regeneration	GO:0006735	0.00073	ENSGALG00010023392,ENSGALG00010021110
GO:BP	canonical glycolysis	GO:0061621	0.00073	ENSGALG00010023392,ENSGALG00010021110
GO:BP	glucose catabolic process to pyruvate	GO:0061718	0.00073	ENSGALG00010023392,ENSGALG00010021110
GO:BP	glucose catabolic process	GO:0006007	0.000844	ENSGALG00010023392,ENSGALG00010021110
GO:BP	glycolytic process through glucose-6-phosphate	GO:0061620	0.000844	ENSGALG00010023392,ENSGALG00010021110
GO:BP	muscle structure development	GO:0061061	0.000844	ENSGALG00010024570,ENSGALG00010019451,ENSGALG00010012253,ENSGALG00010013012,ENSGALG00010006653
GO:BP	nucleobase-containing small molecule metabolic process	GO:0055086	0.000844	ENSGALG00010023392,ENSGALG00010021110,ENSGALG00010009385,ENSGALG00010006069,ENSGALG00010000081

GO:BP	cellular component assembly involved in morphogenesis	GO:0010927	0.000937	ENSGALG00010024570,ENSGALG00010019451,ENSGALG00010006653
GO:BP	organic substance metabolic process	GO:0071704	0.000967	ENSGALG00010029655,ENSGALG00010019451,ENSGALG00010016292,ENSGALG00010000153,ENSGALG00010002079,ENSGALG00010012253,ENSGALG00010018340,ENSGALG00010017826,ENSGALG00010019045,ENSGALG00010023392,ENSGALG00010022774,ENSGALG00010021110,ENSGALG00010009385,ENSGALG00010006069,ENSGALG00010001844,ENSGALG00010004358,ENSGALG00010003750,ENSGALG00010000081
GO:BP	glycolytic process through fructose-6-phosphate	GO:0061615	0.00098	ENSGALG00010023392,ENSGALG00010021110
GO:BP	cell differentiation	GO:0030154	0.001005	ENSGALG00010024570,ENSGALG00010019451,ENSGALG00010000153,ENSGALG00010002079,ENSGALG00010012253,ENSGALG00010017826,ENSGALG00010019045,ENSGALG00010013012,ENSGALG00010006069,ENSGALG00010006653
GO:BP	cellular developmental process	GO:0048869	0.001051	ENSGALG00010024570,ENSGALG00010019451,ENSGALG00010000153,ENSGALG00010002079,ENSGALG00010012253,ENSGALG00010017826,ENSGALG00010019045,ENSGALG00010013012,ENSGALG00010006069,ENSGALG00010006653
GO:BP	myotube differentiation	GO:0014902	0.001084	ENSGALG00010019451,ENSGALG00010012253,ENSGALG00010013012
GO:BP	regulation of developmental process	GO:0050793	0.001105	ENSGALG00010024570,ENSGALG00010019451,ENSGALG00010000153,ENSGALG00010002079,ENSGALG00010012253,ENSGALG00010019045,ENSGALG00010013012,ENSGALG00010021110
GO:BP	tissue morphogenesis	GO:0048729	0.001152	ENSGALG00010024570,ENSGALG00010019451,ENSGALG00010000153,ENSGALG00010012253,ENSGALG00010019045
GO:BP	regulation of actin cytoskeleton organization	GO:0032956	0.001154	ENSGALG00010028598,ENSGALG00010019451,ENSGALG00010000153,ENSGALG00010006653
GO:BP	regulation of supramolecular fiber organization	GO:1902903	0.001154	ENSGALG00010028598,ENSGALG00010019451,ENSGALG00010000153,ENSGALG00010006653

GO:BP	actin cytoskeleton organization	GO:0030036	0.001185	ENSGALG00010028598,ENSGALG00010024570,ENSGALG00010019451,ENSGALG00010000153,ENSGALG00010006653
GO:BP	supramolecular fiber organization	GO:0097435	0.001241	ENSGALG00010028598,ENSGALG00010024570,ENSGALG00010019451,ENSGALG00010000153,ENSGALG00010006653
GO:BP	metabolic process	GO:0008152	0.001406	ENSGALG00010029655,ENSGALG00010019451,ENSGALG00010016292,ENSGALG00010000153,ENSGALG00010002079,ENSGALG00010012253,ENSGALG00010018340,ENSGALG00010017826,ENSGALG00010019045,ENSGALG00010023392,ENSGALG00010022774,ENSGALG00010021110,ENSGALG00010009385,ENSGALG00010006069,ENSGALG00010001844,ENSGALG00010004358,ENSGALG00010003750,ENSGALG00010000081
GO:BP	regulation of biological quality	GO:0065008	0.00148	ENSGALG00010028598,ENSGALG00010019451,ENSGALG00010000153,ENSGALG00010017826,ENSGALG00010006069,ENSGALG00010006653,ENSGALG00010003750,ENSGALG00010023605
GO:BP	purine ribonucleotide metabolic process	GO:0009150	0.00153	ENSGALG00010023392,ENSGALG00010021110,ENSGALG00010009385,ENSGALG00010000081
GO:BP	regulation of actin filament-based process	GO:0032970	0.00153	ENSGALG00010028598,ENSGALG00010019451,ENSGALG00010000153,ENSGALG00010006653
GO:BP	hexose catabolic process	GO:0019320	0.00172	ENSGALG00010023392,ENSGALG00010021110
GO:BP	actin filament-based process	GO:0030029	0.001784	ENSGALG00010028598,ENSGALG00010024570,ENSGALG00010019451,ENSGALG00010000153,ENSGALG00010006653
GO:BP	ribose phosphate metabolic process	GO:0019693	0.001805	ENSGALG00010023392,ENSGALG00010021110,ENSGALG00010009385,ENSGALG00010000081
GO:BP	ribonucleotide metabolic process	GO:0009259	0.001805	ENSGALG00010023392,ENSGALG00010021110,ENSGALG00010009385,ENSGALG00010000081

GO:BP	muscle tissue development	GO:0060537	0.001805	ENSGALG00010024570,ENSGALG00010019451,ENSGALG00010012253,ENSGALG00010006653
GO:BP	regulation of biological process	GO:0050789	0.001805	ENSGALG00010028598,ENSGALG00010024570,ENSGALG00010019451,ENSGALG00010000153,ENSGALG00010002079,ENSGALG00010012253,ENSGALG00010017826,ENSGALG00010019045,ENSGALG00010013012,ENSGALG00010022774,ENSGALG00010021110,ENSGALG00010009385,ENSGALG00010006069,ENSGALG00010001844,ENSGALG00010006653,ENSGALG00010003750,ENSGALG00010023605
GO:BP	tissue development	GO:0009888	0.001982	ENSGALG00010024570,ENSGALG00010019451,ENSGALG00010000153,ENSGALG00010002079,ENSGALG00010012253,ENSGALG00010019045,ENSGALG00010006653
GO:BP	monosaccharide catabolic process	GO:0046365	0.001982	ENSGALG00010023392,ENSGALG00010021110
GO:BP	actin filament organization	GO:0007015	0.002025	ENSGALG00010028598,ENSGALG00010019451,ENSGALG00010000153,ENSGALG00010006653
GO:BP	phosphorylation	GO:0016310	0.002025	ENSGALG00010029655,ENSGALG00010019451,ENSGALG00010000153,ENSGALG00010023392,ENSGALG00010021110,ENSGALG00010009385,ENSGALG00010000081
GO:BP	glucose metabolic process	GO:0006006	0.0021	ENSGALG00010023392,ENSGALG00010021110,ENSGALG00010009385
GO:BP	nitrogen compound metabolic process	GO:0006807	0.002124	ENSGALG00010029655,ENSGALG00010019451,ENSGALG00010000153,ENSGALG00010002079,ENSGALG00010012253,ENSGALG00010018340,ENSGALG00010017826,ENSGALG00010019045,ENSGALG00010023392,ENSGALG00010022774,ENSGALG00010021110,ENSGALG00010009385,ENSGALG00010006069,ENSGALG00010001844,ENSGALG00010003750,ENSGALG00010000081
GO:BP	myoblast fusion	GO:0007520	0.002309	ENSGALG00010019451,ENSGALG00010013012
GO:BP	muscle cell cellular homeostasis	GO:0046716	0.002518	ENSGALG00010019451,ENSGALG00010006653

GO:BP	purine nucleotide metabolic process	GO:0006163	0.002518	ENSGALG00010023392,ENSGALG00010021110,ENSGALG00010009385,ENSGALG00010000081
GO:BP	biological regulation	GO:0065007	0.002682	ENSGALG00010028598,ENSGALG00010024570,ENSGALG00010019451,ENSGALG00010000153,ENSGALG00010002079,ENSGALG00010012253,ENSGALG00010017826,ENSGALG00010019045,ENSGALG00010013012,ENSGALG00010022774,ENSGALG00010021110,ENSGALG00010009385,ENSGALG00010006069,ENSGALG00010001844,ENSGALG00010006653,ENSGALG00010003750,ENSGALG00010023605
GO:BP	multicellular organismal process	GO:0032501	0.002745	ENSGALG00010024570,ENSGALG00010019451,ENSGALG00010000153,ENSGALG00010002079,ENSGALG00010012253,ENSGALG00010019045,ENSGALG00010022774,ENSGALG00010021110,ENSGALG00010006069,ENSGALG00010001844,ENSGALG00010003750,ENSGALG00010023605
GO:BP	animal organ development	GO:0048513	0.002986	ENSGALG00010024570,ENSGALG00010019451,ENSGALG00010000153,ENSGALG00010002079,ENSGALG00010012253,ENSGALG00010019045,ENSGALG00010001844,ENSGALG00010006653
GO:BP	organic cyclic compound metabolic process	GO:1901360	0.003003	ENSGALG00010002079,ENSGALG00010012253,ENSGALG00010017826,ENSGALG00010019045,ENSGALG00010023392,ENSGALG00010022774,ENSGALG00010021110,ENSGALG00010009385,ENSGALG00010006069,ENSGALG00010001844,ENSGALG00010004358,ENSGALG00010000081
GO:BP	monocarboxylic acid metabolic process	GO:0032787	0.003004	ENSGALG00010023392,ENSGALG00010021110,ENSGALG00010009385,ENSGALG00010000081
GO:BP	regulation of cytoskeleton organization	GO:0051493	0.003193	ENSGALG00010028598,ENSGALG00010019451,ENSGALG00010000153,ENSGALG00010006653
GO:BP	carbohydrate biosynthetic process	GO:0016051	0.00321	ENSGALG00010029655,ENSGALG00010023392,ENSGALG00010009385
GO:BP	hexose metabolic process	GO:0019318	0.003322	ENSGALG00010023392,ENSGALG00010021110,ENSGALG00010009385

GO:BP	cellular process	GO:0009987	0.003539	ENSGALG00010028598,ENSGALG00010029655,ENSGALG00010024570,ENSGALG00010019451,ENSGALG00010016292,ENSGALG00010000153,ENSGALG00010002079,ENSGALG00010012253,ENSGALG00010017826,ENSGALG00010019045,ENSGALG00010023392,ENSGALG00010013012,ENSGALG00010022774,ENSGALG00010021110,ENSGALG00010013846,ENSGALG00010009385,ENSGALG00010006069,ENSGALG00010001844,ENSGALG00010006653,ENSGALG00010023605,ENSGALG00010000081
GO:BP	regulation of myotube differentiation	GO:0010830	0.003722	ENSGALG00010019451,ENSGALG00010012253
GO:BP	positive regulation of striated muscle cell differentiation	GO:0051155	0.003924	ENSGALG00010019451,ENSGALG00010012253
GO:BP	nucleotide metabolic process	GO:0009117	0.003924	ENSGALG00010023392,ENSGALG00010021110,ENSGALG00010009385,ENSGALG00010000081
GO:BP	nucleoside phosphate metabolic process	GO:0006753	0.0041	ENSGALG00010023392,ENSGALG00010021110,ENSGALG00010009385,ENSGALG00010000081
GO:BP	monosaccharide metabolic process	GO:0005996	0.004205	ENSGALG00010023392,ENSGALG00010021110,ENSGALG00010009385
GO:BP	syncytium formation	GO:0006949	0.004272	ENSGALG00010019451,ENSGALG00010013012
GO:BP	syncytium formation by plasma membrane fusion	GO:0000768	0.004272	ENSGALG00010019451,ENSGALG00010013012
GO:BP	cell-cell fusion	GO:0140253	0.004272	ENSGALG00010019451,ENSGALG00010013012
GO:BP	negative regulation of cell differentiation	GO:0045596	0.005002	ENSGALG00010019451,ENSGALG00010000153,ENSGALG00010019045,ENSGALG00010013012
GO:BP	myotube cell development	GO:0014904	0.005093	ENSGALG00010012253,ENSGALG00010013012
GO:BP	animal organ morphogenesis	GO:0009887	0.005278	ENSGALG00010024570,ENSGALG00010019451,ENSGALG00010000153,ENSGALG00010012253,ENSGALG00010019045

GO:BP	small molecule metabolic process	GO:0044281	0.005525	ENSGALG00010023392,ENSGALG00010021110,ENSGALG00010009385,ENSGALG00010006069,ENSGALG00010004358,ENSGALG00010000081
GO:BP	cellular metabolic process	GO:0044237	0.005525	ENSGALG00010029655,ENSGALG00010019451,ENSGALG00010016292,ENSGALG00010000153,ENSGALG00010002079,ENSGALG00010012253,ENSGALG00010017826,ENSGALG00010019045,ENSGALG00010023392,ENSGALG00010022774,ENSGALG00010021110,ENSGALG00010009385,ENSGALG00010006069,ENSGALG00010001844,ENSGALG00010000081
GO:BP	nucleobase-containing compound metabolic process	GO:0006139	0.005855	ENSGALG00010002079,ENSGALG00010012253,ENSGALG00010017826,ENSGALG00010019045,ENSGALG00010023392,ENSGALG00010022774,ENSGALG00010021110,ENSGALG00010009385,ENSGALG00010006069,ENSGALG00010001844,ENSGALG00010000081
GO:BP	regulation of actin filament depolymerization	GO:0030834	0.006058	ENSGALG00010028598,ENSGALG00010006653
GO:BP	regulation of actin filament organization	GO:0110053	0.006058	ENSGALG00010028598,ENSGALG00010000153,ENSGALG00010006653
GO:BP	cell fate commitment	GO:0045165	0.006058	ENSGALG00010002079,ENSGALG00010012253,ENSGALG00010019045
GO:BP	cellular carbohydrate metabolic process	GO:0044262	0.006101	ENSGALG00010029655,ENSGALG00010016292,ENSGALG00010023392
GO:BP	heart morphogenesis	GO:0003007	0.006145	ENSGALG00010024570,ENSGALG00010019451,ENSGALG00010019045
GO:BP	positive regulation of muscle cell differentiation	GO:0051149	0.006409	ENSGALG00010019451,ENSGALG00010012253
GO:BP	actin filament depolymerization	GO:0030042	0.006409	ENSGALG00010028598,ENSGALG00010006653
GO:BP	negative regulation of biological process	GO:0048519	0.006431	ENSGALG00010028598,ENSGALG00010024570,ENSGALG00010019451,ENSGALG00010000153,ENSGALG00010002079,ENSGALG00010012253,ENSGALG00010017826,ENSGALG00010019045,ENSGALG00010013012,ENSGALG00010003750

GO:BP	heterocycle metabolic process	GO:0046483	0.006644	ENSGALG00010002079,ENSGALG00010012253,ENSGALG00010017826,ENSGALG00010019045,ENSGALG00010023392,ENSGALG00010022774,ENSGALG00010021110,ENSGALG00010009385,ENSGALG00010006069,ENSGALG00010001844,ENSGALG00010000081
GO:BP	epithelium development	GO:0060429	0.00675	ENSGALG00010019451,ENSGALG00010000153,ENSGALG00010002079,ENSGALG00010012253,ENSGALG00010019045
GO:BP	regulation of cellular process	GO:0050794	0.00675	ENSGALG00010028598,ENSGALG00010024570,ENSGALG00010019451,ENSGALG00010000153,ENSGALG00010002079,ENSGALG00010012253,ENSGALG00010017826,ENSGALG00010019045,ENSGALG00010013012,ENSGALG00010021110,ENSGALG00010009385,ENSGALG00010006069,ENSGALG00010001844,ENSGALG00010006653,ENSGALG00010023605
GO:BP	regulation of myoblast differentiation	GO:0045661	0.006767	ENSGALG00010012253,ENSGALG00010013012
GO:BP	cellular aromatic compound metabolic process	GO:0006725	0.006949	ENSGALG00010002079,ENSGALG00010012253,ENSGALG00010017826,ENSGALG00010019045,ENSGALG00010023392,ENSGALG00010022774,ENSGALG00010021110,ENSGALG00010009385,ENSGALG00010006069,ENSGALG00010001844,ENSGALG00010000081
GO:BP	positive regulation of developmental process	GO:0051094	0.007438	ENSGALG00010019451,ENSGALG00010000153,ENSGALG00010002079,ENSGALG00010012253,ENSGALG00010021110
GO:BP	gluconeogenesis	GO:0006094	0.00782	ENSGALG00010023392,ENSGALG00010009385
GO:BP	hexose biosynthetic process	GO:0019319	0.00782	ENSGALG00010023392,ENSGALG00010009385
GO:BP	regulation of striated muscle cell differentiation	GO:0051153	0.00782	ENSGALG00010019451,ENSGALG00010012253
GO:BP	muscle organ development	GO:0007517	0.00782	ENSGALG00010024570,ENSGALG00010012253,ENSGALG00010006653

GO:BP	cellular component morphogenesis	GO:0032989	0.00782	ENSGALG00010024570,ENSGALG00010019451,ENSGALG00010002079,ENSGALG00010006653
GO:BP	multicellular organism development	GO:0007275	0.00782	ENSGALG00010024570,ENSGALG00010019451,ENSGALG00010000153,ENSGALG00010002079,ENSGALG00010012253,ENSGALG00010019045,ENSGALG00010022774,ENSGALG00010021110,ENSGALG00010001844
GO:BP	muscle tissue morphogenesis	GO:0060415	0.008192	ENSGALG00010024570,ENSGALG00010012253
GO:BP	glycogen metabolic process	GO:0005977	0.00821	ENSGALG00010029655,ENSGALG00010016292
GO:BP	glucan metabolic process	GO:0044042	0.00821	ENSGALG00010029655,ENSGALG00010016292
GO:BP	monosaccharide biosynthetic process	GO:0046364	0.00821	ENSGALG00010023392,ENSGALG00010009385
GO:BP	cellular glucan metabolic process	GO:0006073	0.00821	ENSGALG00010029655,ENSGALG00010016292
GO:BP	regulation of cellular component size	GO:0032535	0.00821	ENSGALG00010028598,ENSGALG00010019451,ENSGALG00010006653
GO:BP	regulation of protein depolymerization	GO:1901879	0.00917	ENSGALG00010028598,ENSGALG00010006653
GO:BP	muscle organ morphogenesis	GO:0048644	0.009452	ENSGALG00010024570,ENSGALG00010012253
GO:BP	energy reserve metabolic process	GO:0006112	0.009736	ENSGALG00010029655,ENSGALG00010016292
GO:BP	cellular nitrogen compound metabolic process	GO:0034641	0.009847	ENSGALG00010002079,ENSGALG00010012253,ENSGALG00010017826,ENSGALG00010019045,ENSGALG00010023392,ENSGALG00010022774,ENSGALG00010021110,ENSGALG00010009385,ENSGALG00010006069,ENSGALG00010001844,ENSGALG00010000081
GO:BP	regulation of cell differentiation	GO:0045595	0.010893	ENSGALG00010019451,ENSGALG00010000153,ENSGALG00010012253,ENSGALG00010019045,ENSGALG00010013012

GO:BP	non-membrane-bounded organelle assembly	GO:0140694	0.01126	ENSGALG00010024570,ENSGALG00010019451,ENSGALG00010006653
GO:BP	carboxylic acid metabolic process	GO:0019752	0.011664	ENSGALG00010023392,ENSGALG00010021110,ENSGALG00010009385,ENSGALG00010000081
GO:BP	negative regulation of developmental process	GO:0051093	0.011664	ENSGALG00010019451,ENSGALG00010000153,ENSGALG00010019045,ENSGALG00010013012
GO:BP	phosphate-containing compound metabolic process	GO:0006796	0.011983	ENSGALG00010029655,ENSGALG00010019451,ENSGALG00010000153,ENSGALG00010023392,ENSGALG00010021110,ENSGALG00010009385,ENSGALG00010000081
GO:BP	oxoacid metabolic process	GO:0043436	0.012078	ENSGALG00010023392,ENSGALG00010021110,ENSGALG00010009385,ENSGALG00010000081
GO:BP	organic acid metabolic process	GO:0006082	0.012245	ENSGALG00010023392,ENSGALG00010021110,ENSGALG00010009385,ENSGALG00010000081
GO:BP	phosphorus metabolic process	GO:0006793	0.012301	ENSGALG00010029655,ENSGALG00010019451,ENSGALG00010000153,ENSGALG00010023392,ENSGALG00010021110,ENSGALG00010009385,ENSGALG00010000081
GO:BP	cellular polysaccharide metabolic process	GO:0044264	0.012685	ENSGALG00010029655,ENSGALG00010016292
GO:BP	regulation of actomyosin structure organization	GO:0110020	0.012855	ENSGALG00010019451,ENSGALG00010000153
GO:BP	myoblast differentiation	GO:0045445	0.013069	ENSGALG00010012253,ENSGALG00010013012
GO:BP	protein depolymerization	GO:0051261	0.013284	ENSGALG00010028598,ENSGALG00010006653
GO:BP	cytoskeleton organization	GO:0007010	0.013284	ENSGALG00010028598,ENSGALG00010024570,ENSGALG00010019451,ENSGALG00010000153,ENSGALG00010006653
GO:BP	polysaccharide metabolic process	GO:0005976	0.013584	ENSGALG00010029655,ENSGALG00010016292

GO:BP	regulation of protein-containing complex disassembly	GO:0043244	0.015083	ENSGALG00010028598,ENSGALG00010006653
GO:BP	cardiac muscle contraction	GO:0060048	0.016223	ENSGALG00010024570,ENSGALG00010019451
GO:BP	regulation of muscle cell differentiation	GO:0051147	0.016449	ENSGALG00010019451,ENSGALG00010012253
GO:BP	organic hydroxy compound metabolic process	GO:1901615	0.016449	ENSGALG00010023392,ENSGALG00010006069,ENSGALG00010004358
GO:BP	regulation of anatomical structure size	GO:0090066	0.016934	ENSGALG00010028598,ENSGALG00010019451,ENSGALG00010006653
GO:BP	regionalization	GO:0003002	0.016934	ENSGALG00010012253,ENSGALG00010019045,ENSGALG00010001844
GO:BP	organophosphate metabolic process	GO:0019637	0.018829	ENSGALG00010023392,ENSGALG00010021110,ENSGALG00010009385,ENSGALG00010000081
GO:BP	cardiac chamber morphogenesis	GO:0003206	0.020406	ENSGALG00010024570,ENSGALG00010019451
GO:BP	striated muscle contraction	GO:0006941	0.020569	ENSGALG00010024570,ENSGALG00010019451
GO:BP	negative regulation of cytoskeleton organization	GO:0051494	0.020569	ENSGALG00010028598,ENSGALG00010019451
GO:BP	negative regulation of supramolecular fiber organization	GO:1902904	0.020569	ENSGALG00010028598,ENSGALG00010019451
GO:BP	negative regulation of MAPK cascade	GO:0043409	0.020569	ENSGALG00010019451,ENSGALG00010000153
GO:BP	pattern specification process	GO:0007389	0.020569	ENSGALG00010012253,ENSGALG00010019045,ENSGALG00010001844
GO:BP	negative regulation of cellular process	GO:0048523	0.020569	ENSGALG00010028598,ENSGALG00010024570,ENSGALG00010019451,ENSGALG00010000153,ENSGALG00010002079,ENSGALG00010012253,ENSGALG00010019045,ENSGALG00010013012

GO:BP	carbohydrate derivative metabolic process	GO:1901135	0.021374	ENSGALG00010023392,ENSGALG00010021110,ENSGALG00010009385,ENSGALG00010000081
GO:BP	regulation of actin polymerization or depolymerization	GO:0008064	0.021418	ENSGALG00010028598,ENSGALG00010006653
GO:BP	regulation of actin filament length	GO:0030832	0.021418	ENSGALG00010028598,ENSGALG00010006653
GO:BP	regulation of organelle organization	GO:0033043	0.021951	ENSGALG00010028598,ENSGALG00010019451,ENSGALG00010000153,ENSGALG00010006653
GO:BP	positive regulation of supramolecular fiber organization	GO:1902905	0.022417	ENSGALG00010000153,ENSGALG00010006653
GO:BP	circulatory system development	GO:0072359	0.022417	ENSGALG00010024570,ENSGALG00010019451,ENSGALG00010019045,ENSGALG00010021110
GO:BP	pallium development	GO:0021543	0.024253	ENSGALG00010000153,ENSGALG00010002079
GO:BP	macromolecule metabolic process	GO:0043170	0.024253	ENSGALG00010029655,ENSGALG00010019451,ENSGALG00010016292,ENSGALG00010000153,ENSGALG00010002079,ENSGALG00010012253,ENSGALG00010018340,ENSGALG00010017826,ENSGALG00010019045,ENSGALG00010022774,ENSGALG00010021110,ENSGALG00010001844,ENSGALG00010003750
GO:BP	organonitrogen compound metabolic process	GO:1901564	0.02521	ENSGALG00010029655,ENSGALG00010019451,ENSGALG00010000153,ENSGALG00010018340,ENSGALG00010023392,ENSGALG00010021110,ENSGALG00010009385,ENSGALG00010006069,ENSGALG00010003750,ENSGALG00010000081
GO:BP	neural precursor cell proliferation	GO:0061351	0.026212	ENSGALG00010000153,ENSGALG00010002079
GO:BP	skeletal muscle tissue development	GO:0007519	0.026474	ENSGALG00010012253,ENSGALG00010006653
GO:BP	heart development	GO:0007507	0.026474	ENSGALG00010024570,ENSGALG00010019451,ENSGALG00010019045

GO:BP	positive regulation of cytoskeleton organization	GO:0051495	0.026574	ENSGALG00010000153,ENSGALG00010006653
GO:BP	actin polymerization or depolymerization	GO:0008154	0.026574	ENSGALG00010028598,ENSGALG00010006653
GO:BP	system development	GO:0048731	0.026574	ENSGALG00010024570,ENSGALG00010019451,ENSGALG00010000153,ENSGALG00010002079,ENSGALG00010019045,ENSGALG00010021110,ENSGALG00010001844
GO:BP	skeletal muscle organ development	GO:0060538	0.027297	ENSGALG00010012253,ENSGALG00010006653
GO:BP	negative regulation of cellular component organization	GO:0051129	0.027297	ENSGALG00010028598,ENSGALG00010019451,ENSGALG00010013012
GO:BP	negative regulation of protein kinase activity	GO:0006469	0.027498	ENSGALG00010019451,ENSGALG00010000153
GO:BP	cardiac chamber development	GO:0003205	0.027498	ENSGALG00010024570,ENSGALG00010019451
GO:BP	heart contraction	GO:0060047	0.028909	ENSGALG00010024570,ENSGALG00010019451
GO:BP	cardiac muscle tissue development	GO:0048738	0.028909	ENSGALG00010024570,ENSGALG00010019451
GO:BP	negative regulation of cell population proliferation	GO:0008285	0.028909	ENSGALG00010024570,ENSGALG00010000153,ENSGALG00010019045
GO:BP	embryo development	GO:0009790	0.028909	ENSGALG00010000153,ENSGALG00010012253,ENSGALG00010019045,ENSGALG00010001844
GO:BP	cellular component assembly	GO:0022607	0.029397	ENSGALG00010028598,ENSGALG00010024570,ENSGALG00010019451,ENSGALG00010000153,ENSGALG00010006069,ENSGALG00010006653
GO:BP	organic substance catabolic process	GO:1901575	0.02966	ENSGALG00010017826,ENSGALG00010023392,ENSGALG00010021110,ENSGALG00010009385,ENSGALG00010000081
GO:BP	developmental growth	GO:0048589	0.030136	ENSGALG00010002079,ENSGALG00010013012,ENSGALG00010006069

GO:BP	negative regulation of kinase activity	GO:0033673	0.030393	ENSGALG00010019451,ENSGALG00010000153
GO:BP	heart process	GO:0003015	0.031426	ENSGALG00010024570,ENSGALG00010019451
GO:BP	central nervous system neuron differentiation	GO:0021953	0.031426	ENSGALG00010002079,ENSGALG00010019045
GO:BP	regulation of macromolecule metabolic process	GO:0060255	0.031426	ENSGALG00010019451,ENSGALG00010000153,ENSGALG00010002079,ENSGALG00010012253,ENSGALG00010017826,ENSGALG00010019045,ENSGALG00010022774,ENSGALG00010021110,ENSGALG00010001844
GO:BP	negative regulation of cell development	GO:0010721	0.031503	ENSGALG00010019451,ENSGALG00010000153
GO:BP	striated muscle tissue development	GO:0014706	0.032277	ENSGALG00010024570,ENSGALG00010019451
GO:BP	protein-containing complex disassembly	GO:0032984	0.033057	ENSGALG00010028598,ENSGALG00010006653
GO:BP	regulation of cell development	GO:0060284	0.033845	ENSGALG00010019451,ENSGALG00010000153,ENSGALG00010012253
GO:BP	regulation of cellular component organization	GO:0051128	0.033845	ENSGALG00010028598,ENSGALG00010019451,ENSGALG00010000153,ENSGALG00010013012,ENSGALG00010006653
GO:BP	cellular component organization	GO:0016043	0.033845	ENSGALG00010028598,ENSGALG00010024570,ENSGALG00010019451,ENSGALG00010000153,ENSGALG00010002079,ENSGALG00010013012,ENSGALG00010013846,ENSGALG00010006069,ENSGALG00010006653
GO:BP	embryonic morphogenesis	GO:0048598	0.034141	ENSGALG00010000153,ENSGALG00010019045,ENSGALG00010001844
GO:BP	negative regulation of transferase activity	GO:0051348	0.036477	ENSGALG00010019451,ENSGALG00010000153
GO:BP	post-transcriptional regulation of gene expression	GO:0010608	0.037231	ENSGALG00010017826,ENSGALG00010022774,ENSGALG00010021110

GO:BP	cellular component biogenesis	GO:0044085	0.037277	ENSGALG00010028598,ENSGALG00010024570,ENSGALG00010019451,ENSGALG00010000153,ENSGALG00010006069,ENSGALG00010006653
GO:BP	anterior/posterior pattern specification	GO:0009952	0.037913	ENSGALG00010012253,ENSGALG00010001844
GO:BP	cellular component organization or biogenesis	GO:0071840	0.038467	ENSGALG00010028598,ENSGALG00010024570,ENSGALG00010019451,ENSGALG00010000153,ENSGALG00010002079,ENSGALG00010013012,ENSGALG00010013846,ENSGALG00010006069,ENSGALG00010006653
GO:BP	muscle contraction	GO:0006936	0.038935	ENSGALG00010024570,ENSGALG00010019451
GO:BP	regulation of cell population proliferation	GO:0042127	0.039515	ENSGALG00010024570,ENSGALG00010000153,ENSGALG00010002079,ENSGALG00010019045
GO:BP	telencephalon development	GO:0021537	0.039585	ENSGALG00010000153,ENSGALG00010002079
GO:BP	regulation of anatomical structure morphogenesis	GO:0022603	0.040373	ENSGALG00010024570,ENSGALG00010019451,ENSGALG00010021110
GO:BP	regulation of cellular metabolic process	GO:0031323	0.040373	ENSGALG00010019451,ENSGALG00010000153,ENSGALG00010002079,ENSGALG00010012253,ENSGALG00010017826,ENSGALG00010019045,ENSGALG00010021110,ENSGALG00010001844
GO:BP	alcohol metabolic process	GO:0006066	0.040966	ENSGALG00010023392,ENSGALG00010006069
GO:BP	positive regulation of cell differentiation	GO:0045597	0.040966	ENSGALG00010019451,ENSGALG00010000153,ENSGALG00010012253
GO:BP	regulation of nitrogen compound metabolic process	GO:0051171	0.041447	ENSGALG00010019451,ENSGALG00010000153,ENSGALG00010002079,ENSGALG00010012253,ENSGALG00010017826,ENSGALG00010019045,ENSGALG00010021110,ENSGALG00010001844
GO:BP	regulation of metabolic process	GO:0019222	0.042396	ENSGALG00010019451,ENSGALG00010000153,ENSGALG00010002079,ENSGALG00010012253,ENSGALG00010017826,ENSGALG00010019045,ENSGALG00010022774,ENSGALG00010021110,ENSGALG00010001844

GO:BP	catabolic process	GO:0009056	0.04534	ENSGALG00010017826,ENSGALG00010023392,ENSGALG00010021110,ENSGALG00010009385,ENSGALG00010000081
GO:BP	regulation of primary metabolic process	GO:0080090	0.046352	ENSGALG00010019451,ENSGALG00010000153,ENSGALG00010002079,ENSGALG00010012253,ENSGALG00010017826,ENSGALG00010019045,ENSGALG00010021110,ENSGALG00010001844
GO:BP	negative regulation of protein phosphorylation	GO:0001933	0.047599	ENSGALG00010019451,ENSGALG00010000153
GO:BP	growth	GO:0040007	0.04785	ENSGALG00010002079,ENSGALG00010013012,ENSGALG00010006069
GO:BP	energy derivation by oxidation of organic compounds	GO:0015980	0.048675	ENSGALG00010029655,ENSGALG00010016292
GO:BP	small molecule catabolic process	GO:0044282	0.048929	ENSGALG00010023392,ENSGALG00010021110
GO:BP	muscle system process	GO:0003012	0.049729	ENSGALG00010024570,ENSGALG00010019451
GO:CC	actin cytoskeleton	GO:0015629	0.015768	ENSGALG00010028598,ENSGALG00010024570,ENSGALG00010000153,ENSGALG00010006653
GO:CC	cytoplasm	GO:0005737	0.015768	ENSGALG00010028598,ENSGALG00010029655,ENSGALG00010024570,ENSGALG00010019451,ENSGALG00010000153,ENSGALG00010018340,ENSGALG00010017826,ENSGALG00010023392,ENSGALG00010022774,ENSGALG00010021110,ENSGALG00010013846,ENSGALG00010009385,ENSGALG00010006069,ENSGALG00010001844,ENSGALG00010006653
GO:CC	cellular_component	GO:0005575	0.034358	ENSGALG00010028598,ENSGALG00010029655,ENSGALG00010024570,ENSGALG00010019451,ENSGALG00010000153,ENSGALG00010012253,ENSGALG00010018340,ENSGALG00010017826,ENSGALG00010019045,ENSGALG00010023392,ENSGALG00010022774,ENSGALG00010021110,ENSGALG00010013846,ENSGALG00010009385,ENSGALG00010006069,ENSGALG00010001844,ENSGALG00010004358,ENSGALG00010017501,ENSGALG00010006653,ENSGALG00010023605

GO:CC	sarcomere	GO:0030017	0.040988	ENSGALG00010028598,ENSGALG00010006653
GO:CC	ruffle	GO:0001726	0.040988	ENSGALG00010024570,ENSGALG00010000153
GO:CC	extracellular space	GO:0005615	0.040988	ENSGALG00010009385,ENSGALG00010006069,ENSGALG00010004358,ENSGALG00010017501
GO:CC	cytosol	GO:0005829	0.040988	ENSGALG00010024570,ENSGALG00010000153,ENSGALG00010018340,ENSGALG00010017826,ENSGALG00010023392,ENSGALG00010021110,ENSGALG00010009385
GO:CC	intracellular anatomical structure	GO:0005622	0.040988	ENSGALG00010028598,ENSGALG00010029655,ENSGALG00010024570,ENSGALG00010019451,ENSGALG00010000153,ENSGALG00010012253,ENSGALG00010018340,ENSGALG00010017826,ENSGALG00010023392,ENSGALG00010022774,ENSGALG00010021110,ENSGALG00010013846,ENSGALG00010009385,ENSGALG00010006069,ENSGALG00010001844,ENSGALG00010006653
GO:CC	cellular anatomical entity	GO:0110165	0.040988	ENSGALG00010028598,ENSGALG00010029655,ENSGALG00010024570,ENSGALG00010019451,ENSGALG00010000153,ENSGALG00010012253,ENSGALG00010018340,ENSGALG00010017826,ENSGALG00010023392,ENSGALG00010022774,ENSGALG00010021110,ENSGALG00010013846,ENSGALG00010009385,ENSGALG00010006069,ENSGALG00010001844,ENSGALG00010004358,ENSGALG00010017501,ENSGALG00010006653,ENSGALG00010023605
GO:CC	transcription regulator complex	GO:0005667	0.041257	ENSGALG00010012253,ENSGALG00010019045,ENSGALG00010001844
GO:CC	myofibril	GO:0030016	0.041749	ENSGALG00010028598,ENSGALG00010006653
GO:CC	contractile fiber	GO:0043292	0.041749	ENSGALG00010028598,ENSGALG00010006653
GO:MF	actin binding	GO:0003779	0.000452	ENSGALG00010028598,ENSGALG00010024570,ENSGALG00010000153,ENSGALG00010004358,ENSGALG00010006653

GO:MF	binding	GO:0005488	0.00083	ENSGALG00010028598,ENSGALG00010029655,ENSGALG00010024570,ENSGALG00010019451,ENSGALG00010016292,ENSGALG00010000153,ENSGALG00010002079,ENSGALG00010012253,ENSGALG00010018340,ENSGALG00010017826,ENSGALG00010019045,ENSGALG00010023392,ENSGALG00010023957,ENSGALG00010022774,ENSGALG00010021110,ENSGALG00010009385,ENSGALG00010006069,ENSGALG00010001844,ENSGALG00010004358,ENSGALG00010006653,ENSGALG00010003750
GO:MF	cytoskeletal protein binding	GO:0008092	0.000866	ENSGALG00010028598,ENSGALG00010024570,ENSGALG00010019451,ENSGALG00010000153,ENSGALG00010004358,ENSGALG00010006653
GO:MF	intramolecular oxidoreductase activity	GO:0016860	0.005255	ENSGALG00010023392,ENSGALG00010009385
GO:MF	protein binding	GO:0005515	0.011037	ENSGALG00010028598,ENSGALG00010029655,ENSGALG00010024570,ENSGALG00010019451,ENSGALG00010016292,ENSGALG00010000153,ENSGALG00010012253,ENSGALG00010018340,ENSGALG00010023392,ENSGALG00010009385,ENSGALG00010006069,ENSGALG00010001844,ENSGALG00010004358,ENSGALG00010006653
GO:MF	single-stranded RNA binding	GO:0003727	0.011739	ENSGALG00010017826,ENSGALG00010022774
GO:MF	nucleic acid binding	GO:0003676	0.016043	ENSGALG00010002079,ENSGALG00010012253,ENSGALG00010017826,ENSGALG00010019045,ENSGALG00010023957,ENSGALG00010022774,ENSGALG00010021110,ENSGALG00010001844
GO:MF	exopeptidase activity	GO:0008238	0.019835	ENSGALG00010018340,ENSGALG00010003750
GO:MF	calmodulin binding	GO:0005516	0.019835	ENSGALG00010029655,ENSGALG00010016292

GO:MF	organic cyclic compound binding	GO:0097159	0.022924	ENSGALG00010029655,ENSGALG00010002079,ENSGALG00010012253,ENSGALG00010017826,ENSGALG00010019045,ENSGALG00010023957,ENSGALG00010022774,ENSGALG00010021110,ENSGALG00010001844,ENSGALG00010004358
GO:MF	isomerase activity	GO:0016853	0.028434	ENSGALG00010023392,ENSGALG00010009385
GO:MF	DNA-binding transcription factor activity	GO:0003700	0.028434	ENSGALG00010002079,ENSGALG00010012253,ENSGALG00010019045,ENSGALG00010001844
GO:MF	actin filament binding	GO:0051015	0.028628	ENSGALG00010028598,ENSGALG00010006653
GO:MF	lyase activity	GO:0016829	0.039499	ENSGALG00010023392,ENSGALG00010000081
GO:MF	RNA polymerase II cis-regulatory region sequence-specific DNA binding	GO:0000978	0.044598	ENSGALG00010002079,ENSGALG00010012253,ENSGALG00010001844
GO:MF	heterocyclic compound binding	GO:1901363	0.044598	ENSGALG00010029655,ENSGALG00010002079,ENSGALG00010012253,ENSGALG00010017826,ENSGALG00010019045,ENSGALG00010023957,ENSGALG00010022774,ENSGALG00010021110,ENSGALG00010001844
GO:MF	cis-regulatory region sequence-specific DNA binding	GO:0000987	0.046697	ENSGALG00010002079,ENSGALG00010012253,ENSGALG00010001844
GO:MF	mRNA binding	GO:0003729	0.047635	ENSGALG00010017826,ENSGALG00010021110
KEGG	Glycolysis / Gluconeogenesis	KEGG:00010	0.000351	ENSGALG00010023392,ENSGALG00010021110,ENSGALG00010009385
KEGG	Carbon metabolism	KEGG:01200	0.001359	ENSGALG00010023392,ENSGALG00010021110,ENSGALG00010009385
KEGG	Biosynthesis of amino acids	KEGG:01230	0.010857	ENSGALG00010023392,ENSGALG00010021110
KEGG	Insulin signaling pathway	KEGG:04910	0.02625	ENSGALG00010029655,ENSGALG00010016292

KEGG	KEGG root term	KEGG:00000	0.02625	ENSGALG00010029655,ENSGALG00010024570,ENSGALG00010019451,ENSGALG00010016292,ENSGALG00010000153,ENSGALG00010017826,ENSGALG00010023392,ENSGALG00010023957,ENSGALG00010021110,ENSGALG00010009385,ENSGALG00010006653
KEGG	Tight junction	KEGG:04530	0.03319	ENSGALG00010000153,ENSGALG00010023957
