# Supplementary material for: Weighted gene co-expression network analysis identifies important modules and hub genes involved in the regulation of breast muscle yield in broilers
Source: Anim Biosci. 2024 Apr 25;37(10):1673–82. doi: 10.5713/ab.23.0548 (PMC11366510; doi:10.5713/ab.23.0548)
Supplement: Supplementary file 14 [file ab-23-0548-Supplementary-Table-14.pdf]

Table S14. Comparison of breast muscle yield between the 817 brolier and Wannan chickens at the market age.

Item	817 broliers	Wannan chickens	P values
Breast muscle yield	19.22±0.78	14.88±0.85	0.00016
