# Supplementary material for: Weighted gene co-expression network analysis identifies important modules and hub genes involved in the regulation of breast muscle yield in broilers
Source: Anim Biosci. 2024 Apr 25;37(10):1673–82. doi: 10.5713/ab.23.0548 (PMC11366510; doi:10.5713/ab.23.0548)
Supplement: Supplementary file 15 [file ab-23-0548-Supplementary-Table-15.pdf]

Table S15. Correlation analysis between the expression of the 6 genes and breast muscle yield in the 817 broilers*.

Genes	Breast muscle yield
CAV3	0.731095**
CFL2	0.625810*
MYF6	0.759531**
TPM1	0.698414**
SH3RF2	–0.620048*
DLX3	–0.708843**

*Significant correlation (P<0.05), **extremely significant correlation (P<0.01)
